# Druggable proteins influencing cardiac structure and function: Implications for heart failure therapies and cancer cardiotoxicity

**DOI:** 10.1126/sciadv.add4984

**Published:** 2023-04-26

**Authors:** Amand F. Schmidt, Mimount Bourfiss, Abdulrahman Alasiri, Esther Puyol-Anton, Sandesh Chopade, Marion van Vugt, Sander W. van der Laan, Christian Gross, Chris Clarkson, Albert Henry, Tom R. Lumbers, Pim van der Harst, Nora Franceschini, Joshua C. Bis, Birgitta K. Velthuis, Anneline S. J. M. te Riele, Aroon D. Hingorani, Bram Ruijsink, Folkert W. Asselbergs, Jessica van Setten, Chris Finan

**Affiliations:** ^1^ Institute of Cardiovascular Science, Faculty of Population Health, University College London, London, UK.; ^2^ UCL BHF Research Accelerator Centre, London, UK.; ^3^ Department of Cardiology, Division Heart and Lungs, University Medical Center Utrecht, Utrecht University, Utrecht, Netherlands.; ^4^ Department of Cardiology, Amsterdam Cardiovascular Sciences, Amsterdam University Medical Centre, University of Amsterdam, Amsterdam, Netherlands.; ^5^ Medical Genomics Research Department, King Abdullah International Medical Research Center, King Saud Bin Abdulaziz University for Health Sciences, Ministry of National Guard Health Affairs, Riyadh, Saudi Arabia.; ^6^ Department of Biomedical Engineering, School of Biomedical Engineering and Imaging Sciences, King's College London, King's Health Partners, London, UK.; ^7^ Central Diagnostics Laboratory, Division Laboratory, Pharmacy, and Biomedical Genetics, University Medical Center Utrecht, Utrecht University, Utrecht, Netherlands.; ^8^ Institute of Health Informatics, Faculty of Population Health, University College London, London, UK.; ^9^ Department of Epidemiology, Gillings School of Global Public Health, University of North Carolina, Chapel Hill, NC, USA.; ^10^ Cardiovascular Health Research Unit, Department of Medicine, University of Washington, Seattle, WA, USA.; ^11^ Department of Radiology, University Medical Center Utrecht, Utrecht University, Utrecht, Netherlands.; ^12^ Netherlands Heart Institute, Utrecht, Netherlands.; ^13^ Member of the European Reference Network for rare, low prevalence, and complex diseases of the heart (ERN GUARD HEART; http://guardheart.ern-net.eu).

## Abstract

Dysfunction of either the right or left ventricle can lead to heart failure (HF) and subsequent morbidity and mortality. We performed a genome-wide association study (GWAS) of 16 cardiac magnetic resonance (CMR) imaging measurements of biventricular function and structure. *Cis-*Mendelian randomization (MR) was used to identify plasma proteins associating with CMR traits as well as with any of the following cardiac outcomes: HF, non-ischemic cardiomyopathy, dilated cardiomyopathy (DCM), atrial fibrillation, or coronary heart disease. In total, 33 plasma proteins were prioritized, including repurposing candidates for DCM and/or HF: IL18R (providing indirect evidence for IL18), I17RA, GPC5, LAMC2, PA2GA, CD33, and SLAF7. In addition, 13 of the 25 druggable proteins (52%; 95% confidence interval, 0.31 to 0.72) could be mapped to compounds with known oncological indications or side effects. These findings provide leads to facilitate drug development for cardiac disease and suggest that cardiotoxicities of several cancer treatments might represent mechanism-based adverse effects.

## Teaser

Combining deep-learning of CMR, GWAS, and Mendelian randomization identified 33 plasma proteins affecting cardiac outcomes.

## INTRODUCTION

Dysfunction of the right or left ventricle (RV, LV), arising due to intrinsic heart muscle disease, coronary artery disease, or pulmonary or systemic hypertension, leads to the clinical syndrome of heart failure (HF) (*1*). HF can be accompanied by ventricular hypertrophy or dilation (depending on the cause) and with impairment of either cardiac contraction or relaxation, leading to HF syndromes defined according to impaired or preserved ejection fraction (EF).

Notwithstanding the recent advance offered by SGLT2 inhibitors for the treatment of HF, drug development for cardiac disease has been met with high failure rates, often occurring during costly late-stage clinical testing (*2*–*4*). These late-stage failures are indicative of the poor predictive potential of preclinical experiments for cardiac drug target identification. This is complicated further by the considerable phenotypic heterogeneity that underlies diagnoses such as HF (*5*), potentially resulting in compounds failing for futility that may genuinely benefit a subset of patients. Conversely, several drugs—predominantly for cancer indications—have been found to cause cardiotoxicity, which may confront patients with treatment-induced heart problems (*6*).

Cardiac magnetic resonance (CMR) imaging is the gold standard for quantification of biventricular function and morphology, and has become an integral diagnostic modality for cardiac disease (see table S1). Here, we used CMR images from the UK Biobank (UKB) to extract measures from both LV and RV using a purpose-built deep-learning algorithm (*7*).

Proteins constitute most drug targets (*8*), which are increasingly analyzed through high-throughput assays measuring the levels of hundreds to thousands of (plasma) proteins (*9*). To leverage proteins and CMR measurements for drug target validation, we have developed an analytical framework (*10*) to perform drug target analyses using human genetic data. Specifically, through two-sample Mendelian randomization (MR), one can anticipate the on-target effect a drug target protein will have on disease-relevant traits such as CMR measurements. Previously, this approach has been extensively validated for cardiovascular drug targets (*11*–*19*).

**Fig. 1.**
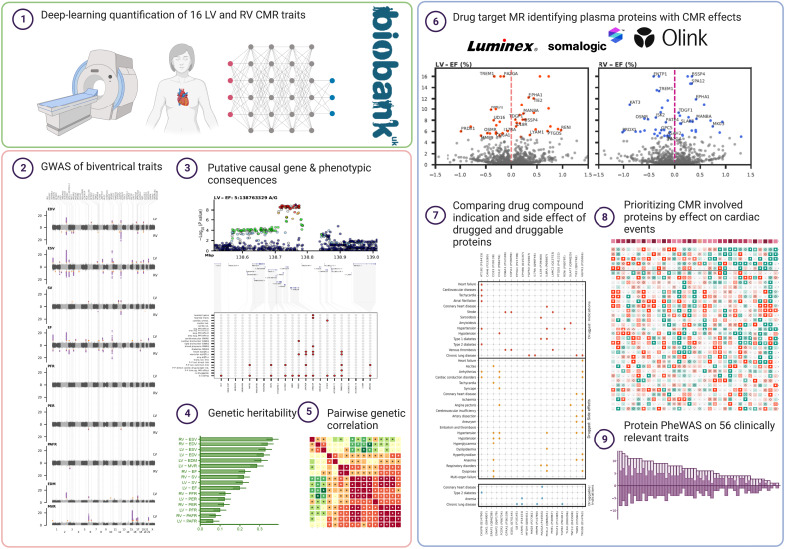
Study infographic leveraging neural network analysis of biventricular CMR data and GWAS to prioritize plasma proteins with an effect on cardiac outcomes. CMR, cardiac magnetic resonance imaging (MRI); GWAS, genome-wide association study.

To prioritize circulating plasma proteins for their involvement with LV and RV traits, we first performed a genome-wide association study (GWAS) on 16 CMR traits measured in up to 36,548 UKB subjects. Subsequently, we format-normalized protein quantitative trait loci (pQTLs) data sourced from three independent GWAS involving cross-platform measurement of plasma protein concentrations using Somalogic (*9*), Olink (*20*), and Luminex (*21*) assays spanning 2900+ plasma proteins. Drug target MR was used to prioritize proteins on their likely causal contribution to CMR traits, as well as cardiac outcomes including HF, dilated cardiomyopathy (DCM), and atrial fibrillation (AF). Repurposing opportunities were identified by extracting cardiovascular indications and side effects from ChEMBL (*22*) and the British National Formulary (BNF). Results were further annotated with tissue-specific mRNA expression data from the Human Protein Atlas (HPA) database (*23*), and with protein-protein interaction data information from IntAct (*24*) (see Fig. 1).

## RESULTS

### UKB participants with LV and RV CMR measurements

CMR measurements were obtained from a sample of up to 36,548 UKB subjects using an extensively validated deep-learning approach (*7*), excluding people with preexisting (cardiac) disease (figs. S1 and S2 and tables S1 and S2). Specifically, the following CMR measurements were extracted: structural measures on end-diastolic, end-systolic, or stroke volumes (EDV, ESV, SV), end-diastolic mass (EDM), and LV mass to EDV ratio (LV-MVR), and functional measures on EF, peak ejection rate (PER), and peak (atrial) filling rates (PAFR, PFR).

On average, subjects were 63.9 (SD, 7.6) years old, and 18,879 (51.8%) were women. Participants had a mean systolic blood pressure (SBP) of 138.2 mmHg (SD, 18.4), a mean diastolic blood pressure (DBP) of 78.6 mmHg (SD, 10.0), and a mean heart rate of 62.5 beats per minute (bpm) (SD, 10.2) (see table S3).

### Genomic loci associated with LV and RV CMR measurements

**Fig. 2.**
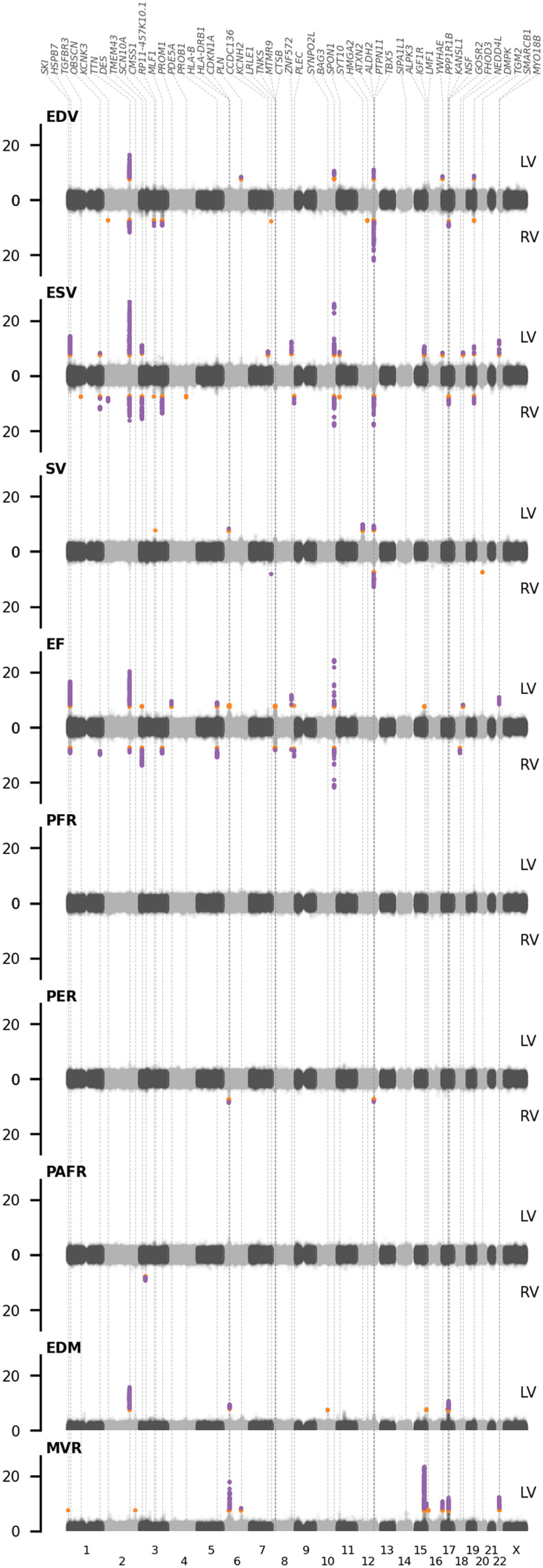
Manhattan plots of genome-wide CMR associations with genomic annotations. Purple dots indicate associations that pass the conservative significant threshold of 7.14 × 10^−9^, with orange dots associating between 5.00 × 10^−8^ and 7.14 × 10^−9^; labels indicate the lead gene in the region. CMR, cardiac MRI; LV, left ventricle; RV, right ventricle; EDV, end-diastolic volume; ESV, end-systolic volume; SV, stroke volume; EF, ejection fraction; PER, peak ejection rate; PAFR/PFR, peak (atrial) filling rate; EDM, end-diastolic mass; MVR, ratio between end diastolic mass and volume. Results are based on an analysis of up to 36,548 subjects.

We performed a GWAS on 16 CMR traits, leveraging genotyped and imputed variants from the Affymetrix BiLEVE and Axiom arrays, and applying BOLT-LMM conditional on age, sex, body surface area (BSA), SBP, genotype measurement batch, 40 principal components (PCs), and assessment center. This resulted in 91 unique lead variants (Fig. 2 and tables S4 and S5), which passed the standard GWAS significance threshold of 5.00 × 10^−8^, and 32 variants passing a more conservative threshold of 7.14 × 10^−9^, accounting for the number of PCs necessary to explain 90% of the CMR trait variance (figs. S3 and S4). This included lead variants in or around genes known to affect cardiac outcomes, such as *BAG3*, *TTN*, *SMARCB1*, *SYNPO2L*, *TBX5*, and *IGF1R.* Please see Supplementary Results, figs. S4 to S8, and tables S5 to S8 for a full description of the GWAS findings.

### Proteins with MR effects on CMR measurements

**Fig. 3.**
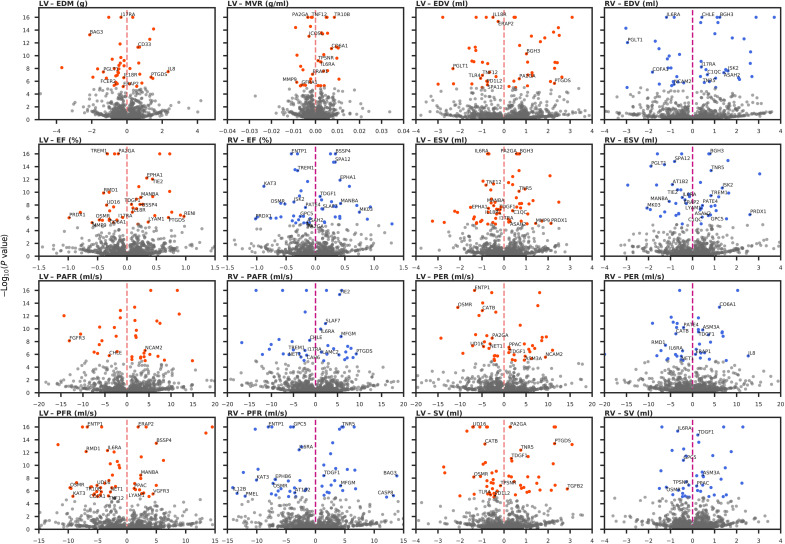
Volcano plots of the plasma protein effect on CMR traits. Proteins were annotated if they were part of the drugged, druggable, directionally concordant, or nearest druggable protein sets. Results were colored by LV and RV if they passed a *P* value threshold of 7.81 × 10^−6^. The MR effects per unit (in SD) change in the protein are plotted on the *x* axis, against the −log_10_(*P* value) on the *y* axis. Nomenclature: Proteins are referred to by their UniProt entry name to differentiate them from the encoding genes.

**Fig. 4.**
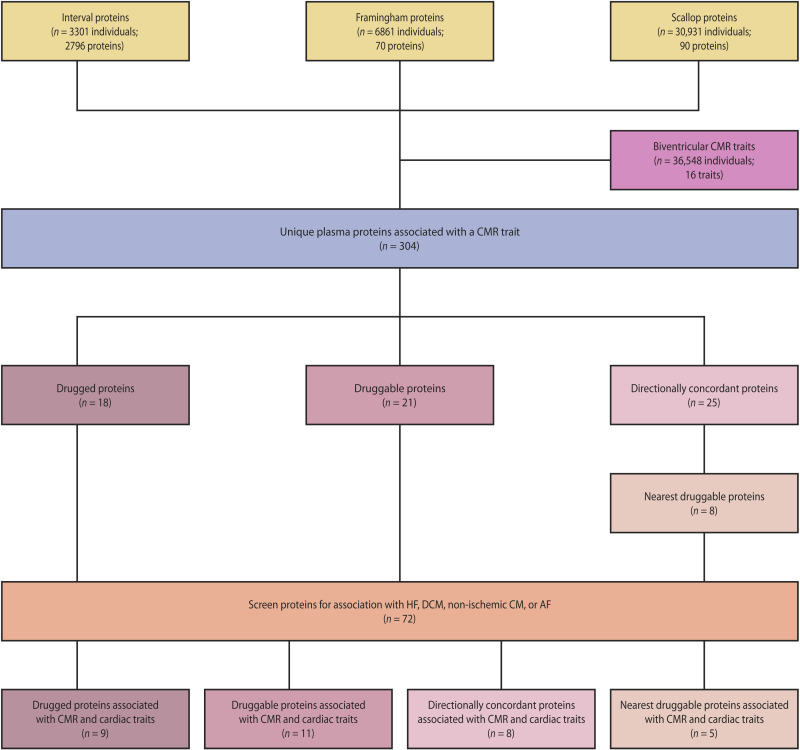
Drug target MR pipeline to identify plasma proteins associating with CMR traits and cardiac outcomes. Plasma proteins were associated with CMR traits through *cis*-MR leveraging genetic variants (pQTLs) associated with the protein of interest. Multiplicity-corrected results were prioritized by identifying 4 sets of proteins: (i) whether the protein was targeted by an approved drug ('drugged proteins' set), (ii) whether the protein was targeted by a developmental drug compound ('druggable proteins' set), (iii) whether the protein showed a directionally concordant (table S1) effect on at least three CMR traits ('concordant proteins' set), and (iv) for the directionally concordant proteins we additionally identified their nearest druggable protein through protein-protein data from IntAct and included proteins with pQTLs available in the three available datasets (SCALLOP, Framingham, INTERVAL) ('nearest druggable proteins' set). These 72 proteins were finally prioritized on an MR association with HF, DCM, CM, and/or AF.

**Fig. 5.**
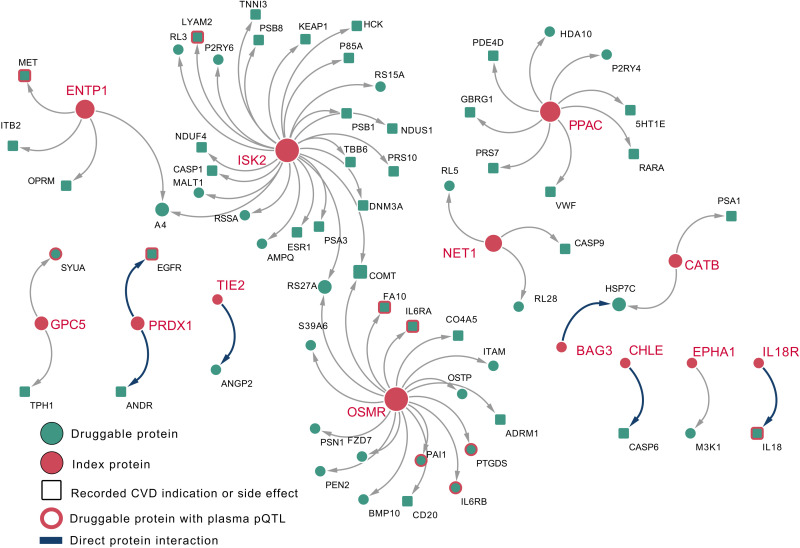
A network of plasma proteins with a directionally concordant CMR effect (index, orange circles) and their nearest druggable protein (green square with record CVD indication or side effect). Directly interacting proteins are presented as a thick blue arrow; the remaining druggable proteins were separated by a single intermediate protein. In the presence of ties, all druggable proteins with the same distance are presented. Druggable proteins with a CVD indication or side effect (based on BNF and ChEMBL) are presented as squares. In addition, nonindexing proteins with available plasma pQTL data are represented by an orange outline. Nomenclature: Proteins are referred to by their UniProt entry name to differentiate them from the encoding genes.

**Fig. 6.**
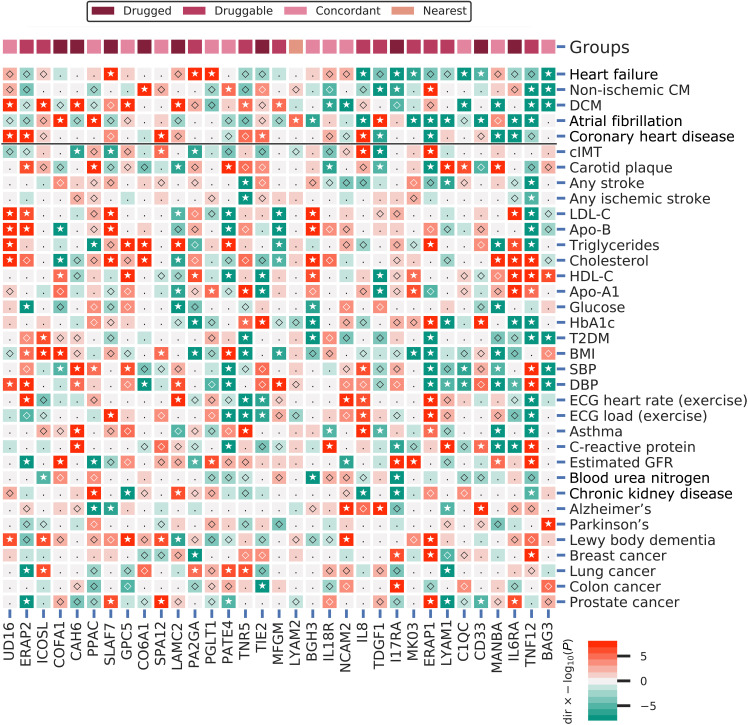
A phenome-wide scan of CMR prioritized proteins associated with one or more cardiac outcomes. Proteins were curated on having a multiplicity-corrected *P* < 1.29 × 10^−5^ with one or more of the following cardiac traits: HF, DCM, non-ischemic CM, AF, or CHD. *P* values passing the 0.05 threshold are indicated by an open diamond, with stars indicating results passing a threshold of 1.29 × 10^−5^. Cells were colored by effect direction multiplied by the −log_10_(*P* value); where *P* values were truncated at 8 for display purposes. The top column indicates whether the CMR-associated proteins were identified as drugged, druggable, directionally concordant, or nearest druggable protein. DCM, dilated cardiomyopathy; cIMT, carotid artery intima media thickness; T2DM, type 2 diabetes; BMI, body mass index; DBP/SBP, diastolic/systolic blood pressure; Estimated GFR, estimated glomerular filtration rate; BUN, blood urea nitrogen; LDL-C, low-density lipoprotein cholesterol; HDL-C, high-density lipoprotein cholesterol; Apo-B, apolipoprotein-B; Apo-A1, apolipoprotein-A1; HbA1c, glycated hemoglobin; ECG, electrocardiography; FVC, forced vital capacity; FEV1, forced expiratory volume during the first second; PEF, peak expiratory flow. Note that all 56 phenome-wide traits are presented in fig. S15. Nomenclature: Proteins are referred to by their UniProt entry name to differentiate them from the encoding genes.

Drug target MR was used to identify plasma proteins affecting CMR traits. Specifically, we identified 304 circulating proteins with a causal effect on RV and/or LV traits by performing a two-sample *cis*-MR (Fig. 3), leveraging aggregated genetic data on protein levels from three sources (SCALLOP, Framingham, and INTERVAL), and associating these with results from the CMR GWAS discovery analysis (Fig. 4 and figs. S8 and S9). Subsequently, we identified CMR-associated proteins that were either drugged or druggable (figs. S10 and S11 and tables S9 to S12). Because of novel ways of drug delivery and developments such as small interfering RNA–based therapeutics, the current definition of druggability will likely change in the future. In anticipation of this, we additionally identified a set of proteins with directionally concordant effects on at least three CMR traits and used protein-protein interaction data to identify “nearest druggable” proteins with sufficient pQTL data to conduct follow-up MR analysis with (Fig. 5, figs. S13 and S14, and tables S13 to S15). This resulted in a set of 72 proteins, which were further prioritized by using MR to identify proteins associated with at least one of the following cardiac outcomes: HF, DCM, non-ischemic cardiomyopathy (CM), AF, and/or coronary heart disease (CHD). This strategy resulted in a final set of 33 prioritized proteins, where 9 proteins affected HF, 13 DCM, 7 non-ischemic CM, 12 AF, and 9 CHD (Fig. 6, fig. S15, and table S16).

The prioritized proteins included 25 targets that were directly or indirectly (where the indexing protein interacted with a drugged or druggable protein) drugged or druggable: IL6RA, IL8, CO6A1, LYAM1, PA2GA, ISK2, CD33, PPAC, COFA1, GPC5, TIE2, IL18R, LAMC2, I17RA, SLAF7 (Table 1). From a level 1 look-up of Anatomical Therapeutic Chemical (ATC) entries, we know that 171 (15%) registered compounds have a cardiovascular indication. Compared to this, we observed significant cardiovascular enrichment, with 15 [60%; 95% confidence interval (CI), 39 to 79] of the 25 drugged or druggable proteins having a known cardiovascular indication or side effect (figs. S13 and S14).

**Table 1.** Summarizing prioritized plasma protein associated with CMR and cardiac outcomes.

Protein (UniProt ID)	Nearest druggable protein (UniProt ID)	Druggability	Ventricle	CMR effect	Cardiac effect	Clinical development phase	No. compounds	Compound action types	CVD indication or side effects	Oncological indication or side effects
MANBA (O00462)		Currently not druggable	Both	Beneficial	Beneficial	0	0			
NCAM2 (O15394)		Currently not druggable	Both	Beneficial	Beneficial	0	0			
TNF12 (O43508)		Directly druggable	Left	Mixed	Beneficial	1	2	Inhibitor		✓
ICOSL (O75144)		Directly druggable	Left	Beneficial	Harmful	1	1	Inhibitor		
BAG3 (O95817)	HSP7C (P11142)	Indirectly druggable	Both	Beneficial	Beneficial	3	1	Inhibitor		
C1QC (P02747)		Currently not druggable	Both	Harmful	Beneficial	0	0			
IL6RA (P08887)		Directly drugged	Both	Mixed	Beneficial	4	4	Antagonist, inhibitor	✓	✓
PATE4 (P0C8F1)		Currently not druggable	Right	Harmful	Harmful	0	0			
IL8 (P10145)		Directly druggable	Both	Mixed	Mixed	2	2	Inhibitor	✓	✓
CO6A1 (P12109)		Directly drugged	Both	Mixed	Harmful	4	2	Hydrolytic enzyme	✓	
TDGF1 (P13385)		Directly druggable	Both	Beneficial	Mixed	1	1	Binding agent		✓
LYAM1 (P14151)		Directly druggable	Both	Mixed	Beneficial	3	3	Antagonist, inhibitor	✓	
PA2GA (P14555)		Directly druggable	Left	Mixed	Harmful	3	2	Inhibitor	✓	
ISK2 (P20155)	LYAM2 (P16581)	Indirectly druggable	None	None	Harmful	3	3	Antagonist, inhibitor	✓	
UD16 (P19224)		Currently not druggable	Left	Harmful	Harmful	0	0			
CD33 (P20138)		Directly drugged	Left	Harmful	Beneficial	4	6	Binding agent, other	✓	✓
CAH6 (P23280)		Directly drugged	Right	Harmful	Harmful	4	1	Inhibitor		
PPAC (P24666)	5HT1E (P28566),HDA10 (Q969S8),PDE4D (Q08499),RARA (P10276),VWF (P04275),P2RY4 (P51582),PRS7 (P35998),GBRG1 (Q8N1C3)	Indirectly drugged	Both	Beneficial	Harmful	4	94	Agonist, allosteric antagonist, antagonist, inhibitor, inverse agonist, modulator, partial agonist, positive allosteric modulator, positive modulator	✓	✓
TNR5 (P25942)		Directly druggable	Both	Mixed	Harmful	2	5	Agonist, antagonist, inhibitor, partial agonist		✓
MK03 (P27361)		Directly druggable	Right	Beneficial	Beneficial	2	3	Inhibitor		✓
COFA1 (P39059)		Directly drugged	Both	Mixed	Harmful	4	2	Hydrolytic enzyme	✓	
GPC5 (P78333)	SYUA (P37840),TPH1 (P17752)	Indirectly drugged	Right	Harmful	Harmful	4	4	Inhibitor	✓	✓
TIE2 (Q02763)		Directly drugged	Both	Beneficial	Harmful	4	8	Inhibitor	✓	✓
MFGM (Q08431)		Directly druggable	Right	Beneficial	Harmful	1	1	Binding agent		
IL18R (Q13478)	IL18 (Q14116)	Indirectly druggable	Left	Beneficial	Beneficial	2	2	Cross-linking agent, inhibitor	✓	
LAMC2 (Q13753)		Directly drugged	Right	Beneficial	Harmful	4	1	Hydrolytic enzyme	✓	
BGH3 (Q15582)		Currently not druggable	Both	Harmful	Beneficial	0	0			
ERAP2 (Q6P179)		Directly druggable	Both	Beneficial	Harmful	2	1	Inhibitor		✓
SPA12 (Q8IW75)		Currently not druggable	Both	Beneficial	Harmful	0	0			
PGLT1 (Q8NBL1)		Currently not druggable	Both	Beneficial	Harmful	0	0			
I17RA (Q96F46)		Directly drugged	Both	Mixed	Beneficial	4	1	Antagonist	✓	
SLAF7 (Q9NQ25)		Directly drugged	Right	Beneficial	Harmful	4	1	Inhibitor	✓	✓
ERAP1 (Q9NZ08)		Directly druggable	Both	Beneficial	Mixed	2	1	Inhibitor		✓

Indirectly drugged or indirectly druggable proteins were mapped to the nearest druggable protein (fig. S8), potentially resulting in tied proteins at the same distance. Tied proteins were both included in further analyses, for example, resulting in eight nearest druggable proteins for PPAC explaining the large number of mapped compounds. For indirectly drugged/druggable proteins, the CMR and cardiac effects represent the indexing protein for which we had plasma pQTL (the first column) and the drug compound data are from the nearest druggable protein(s). The nearest druggable protein of ISK2, LYAM2, was available as pQTL data; hence, here we instead list the LYAM2 effects on CMR and cardiac traits. Clinical development phase refers to the phase of clinical testing (0 no human testing). Nomenclature: Proteins are referred to by their UniProt entry name to differentiate them from the encoding genes.

### Proteins with MR effects on HF, DCM, or non-ischemic CM

Twenty-five proteins associated with HF, DCM, or non-ischemic CM (Figs. 3 and 6 and figs. S10 to S12). These included BAG3, TNF12, C1QC, and IL18R, which affected more than one cardiac trait, as well as proteins targeted by approved compounds: CD33, SLAF7, CO6A1, LAMC2, I17RA, and CAH6 (Figs. 3 and 6 and figs. S10 to S14). Specifically, higher plasma concentrations of BAG3 (BAG family molecular chaperone regulator 3) improved six CMR traits and decreased the risk of HF [odds ratio (OR), 0.75; 95% CI, 0.72 to 0.79], non-ischemic CM (OR, 0.30; 95% CI, 0.25 to 0.36), and DCM (OR, 0.14; 95% CI, 0.11 to 0.17) (Fig. 6, fig. S12, and table S16). BAG3 directly interacts with HSP7C, which is targeted by forigerimod acetate, a phase 3 compound for lupus erythematosus (Fig. 5 and table S14). Higher levels of TNF12 (tumor necrosis factor ligand superfamily member 12) decreased the risk of non-ischemic CM (OR, 0.82; 95% CI, 0.77 to 0.88) and DCM (OR, 0.80; 95% CI, 0.75 to 0.85). TNF12 is targeted by two phase 1 inhibiting compounds indicated for neoplasm and rheumatoid arthritis (tables S11, S12, and S16). Higher levels of C1QC (complement C1q subcomponent subunit C) detrimentally affected three CMR traits but nevertheless decreased the risk of HF (OR, 0.97; 95% CI, 0.96 to 0.98) and DCM (OR, 0.86; 95% CI, 0.82 to 0.90) (Fig. 6, fig. S12, and table S16). Increased levels of CD33 (myeloid cell surface antigen CD33) decreased the risk of HF (OR, 0.96; 95% CI, 0.95 to 0.98) and reduced LV-EDM. CD33 is targeted by monoclonal antibodies (mAbs) such as gemtuzumab, which are indicated in cancer such as acute myeloid leukemia and have documented cardiovascular side effects (fig. S13 and tables S9 and S10). SLAF7 (SLAM family member 7) is targeted by inhibiting compounds indicated for the treatment of cancers, which have known cardiometabolic side effects (fig. S13 and tables S9 and S10). Through MR, we found that SLAF7 improved RV-EF and RV-PAFR, but nevertheless increased the risk of HF (OR, 1.07; 95% CI, 1.05 to 1.08) (Figs. 3 and 6 and fig. S10). Higher plasma levels of IL18R improved four LV CMR traits and decreased the risk of DCM (OR, 0.88; 95% CI, 0.83 to 0.92). IL18R directly interacts with IL18, which is targeted by developmental compounds for diabetes and inflammatory bowel disease (fig. S14 and table S14).

### Proteins with an MR effect on AF

The 12 proteins associated with AF included COFA1, PPAC, LYAM2, BGH3, IL8, TDGF1, MK03, ERAP1, LYAM1, CD33, ILRA, and TNF12 (Figs. 3 and 6 and figs. S10 to S14). Specifically, higher levels of IL8 (interleukin-8) increased LV-EDM, improved RV-PER, and decreased the risk of AF (OR, 0.83; 95% CI, 0.77 to 0.89), as well as HF (OR, 0.74; 95% CI, 0.69 to 0.81). IL8 is targeted by a mAb in development for the treatment of neoplasms and chronic lung disease (Fig. 6, figs. S11 and S13, and tables S11 and S12). TDGF1 (teratocarcinoma-derived growth factor 1) is targeted by BIIB015, which is being developed as an immunoconjugate for the treatment of tumors. Higher levels of TDGF1 improved LV and RV cardiac traits (EF, SV, PER, and RV-PFR) and increased the risk of AF (OR, 1.01; 95% CI, 1.01 to 1.01) while decreasing the risk of non-ischemic CM (OR, 0.93; 95% CI, 0.92 to 0.94). MK03 (mitogen-activated protein kinase 3) is targeted by multiple extracellular signal–regulated kinase 1/2 (ERK1/2) kinase inhibitors for the treatment of neoplasms. Higher levels of MK03 associated with RV-ESV and RV-EF, and decreased the risk of AF (OR, 0.86; 95% CI, 0.82 to 0.91) as well as HF (OR, 0.85; 95% CI, 0.80 to 0.91) (Figs. 3 and 6 and fig. S11). ERAP1 and ERAP2 [endoplasmic reticulum aminopeptidase 1 and 2, which form a protein complex (*25*)], both play a role in peptide trimming for presentation on major histocompatibility complex (MHC) class I, and are both targeted by tosedostat, which is currently in development for the treatment of cancers (fig. S13 and tables S11 and S12). Higher ERAP1 decreased the risk of AF (OR, 0.99; 95% CI, 0.98 to 0.99) and CHD (OR, 0.98; 95% CI, 0.97 to 0.98) and increased the risk of non-ischemic CM (OR, 1.10; 95% CI, 1.07 to 1.13). Higher levels of ERAP2 increased the risk of CHD (OR, 1.03; 95% CI, 1.02 to 1.03).

### Proteins with an MR effect on CHD

The nine proteins associated with CHD included UD16, SPA12, MANBA, IL6RA, ERAP1, ERAP2, TIE2, IL8, and TDGF1 (Figs. 3 and 6 and fig. S10 to S14). Specifically, IL6RA (interleukin-6 receptor subunit α) had a directionally discordant effect on nine LV and RV traits (fig. S10 and tables S9 and S10), with increased levels being associated with decreased risk of CHD (OR, 0.94; 95% CI, 0.93 to 0.94) and AF (OR, 0.95; 95% CI, 0.94 to 0.96) (table S16). Higher UD16 (UDP-glucuronosyltransferase 1-6) worsened four LV CMR traits and increased the risk of DCM (OR, 1.62; 95% CI, 1.46 to 1.80) and CHD (OR, 1.06; 95% CI, 1.04 to 1.08). TIE2 (angiopoietin-1 receptor) is targeted by inhibiting compounds indicated for the treatment of cancers, which have known cardiometabolic side effects (fig. S13). Higher levels of TIE2 affected LV-EF (0.43%; 95% CI, 0.32 to 0.55), RV-ESV (−0.68 ml; 95% CI, −0.89 to −0.48), and RV-PAFR (5.47 ml/s; 95% CI, 4.15 to 6.79) and increased the risk of CHD (OR, 1.10; 95% CI, 1.06 to 1.15). Higher concentrations of MANBA improved five CMR traits (ESV, EF, and LV-PFR) and decreased CHD (OR, 0.93; 95% CI, 0.91 to 0.96) and DCM risk (OR, 0.76; 95% CI, 0.72 to 0.81) (Figs. 3 and 6 and fig. S12).

### Tissue-specific expression and phenome-wide scan

**Fig. 7.**
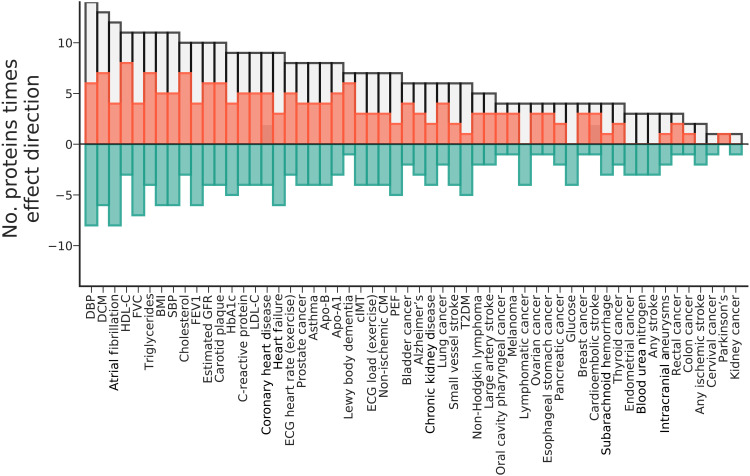
The frequency of a prioritized plasma protein affected the depicted trait. The absolute frequencies (i.e., counts) of associations areprovided as a gray bars, reflecting the number MR estimates that passed a multiplicity-corrected *P* value threshold of 7.81 × 10^−6^. The effect direction of these associations is depicted in orange (counting the number of positive associations) or green (counting the number of negative associations), which sum to the absolute frequency. Plasma proteins were prioritized (Fig. 4) for involvement with CMR traits and cardiac outcomes (AF, CHD, HF, DCM, and non-ischemic CM). Note that the individual protein-trait associations are presented in fig. S15 and a subset in Fig. 6.

To inform possible drug development of these 33 prioritized plasma proteins, we next explored tissue-specific mRNA expression and performed a phenome-wide scan to identify the potential spectrum of effects of on-target perturbation (Figs. 6 and 7, figs. S15 and S16, and table S16). Tissue specificity did not differ between prioritized proteins and nonprioritized proteins (*P* = 0.20). We did observe a significant difference in tissue-specific expression (*P* = 9.01 × 10^−3^), with prioritized plasma proteins more frequently highly expressed in spleen, lymph node, liver, granulocytes, kidney, pancreas, and lung tissues (fig. S16). In addition to the cardiac outcomes these proteins were prioritized for, the *cis-*MR phenome-wide scan showed that these proteins were frequently associated with DBP, SBP, electrocardiography (ECG) measurement during exercise, lipid fractions [e.g. high-density lipoprotein cholesterol (HDL-C), apolipoprotein-A1 (Apo-A1), triglycerides, low-density lipoprotein cholesterol (LDL-C), and apolipoprotein-B (Apo-B)], estimated glomerular filtration rate (eGFR), body mass index (BMI), glycosylated hemoglobin (HbA1c), C-reactive protein, lung function [forced expiratory volume during the first second (FEV1), forced vital capacity (FVC), and peak expiratory flow (PEF)], and carotid intima-media thickness (cIMT) (Figs. 6 and 7, fig. S15, and table S16).

## DISCUSSION

In the current study, we derived 16 LV and RV measurements of structure or function from CMR and used GWAS to identify 91 genetic variants associated with one or more traits. We leveraged drug target MR to identify 33 plasma proteins associated with RV or LV measurements as well as with at least one of the following cardiac outcomes: CHD, AF, HF, DCM, and non-ischemic CM. To further inform drug development, we conducted a phenome-wide scan assessing the on-target effects these 33 proteins may have on 56 clinically relevant traits. We found that 15 (60%; 95% CI, 39 to 79) of the 25 drugged or druggable proteins were targeted by compounds with a cardiovascular indication or side effect (Table 1).

The *cis*-MR analysis leveraged three distinct plasma pQTL resources, distilling a prioritized set of 33 plasma proteins that affect both CMR traits and cardiac outcomes (Table 1). While these proteins were prioritized on their association with CMR traits and cardiac outcomes, the association between a proteins’ CMR effect direction and cardiac outcome effect direction (categorized as “beneficial,” “harmful,” or “mixed,” the latter for multiple directionally discordant protein effects) did not reach significance (*P* = 0.85). This likely reflects imperfect understanding of the relation between CMR traits and disease. Furthermore, given the strong (observational and genetic) correlation among CMR traits, inference might be further improved by considering CMR traits jointly.

Our analyses have highlighted drug targets that affect multiple cardiac outcomes (Fig. 6 and Table 1). Some of these proteins are closely linked, for example, plasma ERAP1 affects CHD, AF, and non-ischemic CM, and is closely related to ERAP2, which showed a directionally opposing effect on CHD. This discordance in effects might be explained by a correspondingly opposing effect on known CHD risk factors/intermediates such as lipoprotein(a), DBP, and carotid plaque. Both ERAP1 and ERAP2 play a major role in peptide trimming for presentation on MHC class I molecules (*26*), which is involved in cardiomyocyte pathogenesis (*27*). Similarly, TNF12 decreases the risk of non-ischemic CM, DCM, and AF and promotes IL8 concentration, which we linked to a lower risk of AF and HF. IL8 concentration has previously been associated with HF and AF outcomes, supporting these observations (*28*, *29*). In agreement with previous loss-of-function studies, we found that higher plasma concentrations of BAG3 affected multiple CMR traits as well as HF, DCM, and non-ischemic CM risk (*30*). BAG3 is indirectly druggable through a protein-protein interaction with HSP7C (heat shock cognate 71 kDa protein), for which BAG3 acts as a co-chaperone (*31*). Now, the BAG3-HSP7C interaction is being explored as a drug target in animal models (*32*, *33*).

Compared to recent CMR GWASs (*34*–*36*), this study uniquely determined LV and RV PER, PFR, and PAFR measurements (tables S17 and S18), where PFR is especially relevant for HF with preserved EF. Through *cis-*MR of plasma pQTLs, we identified seven proteins that affected PFR as well as HF or DCM risk: UD16, MANBA, TNR5, TNF12, MFGM, CPC5, and BAG3, where the last five proteins were (indirectly) drugged or druggable, providing leads for drug development (Table 1).

Through BNF and ChEMBL linkage, we found that 13 (52%; 95% CI, 0.31 to 0.72) of the 25 drugged or druggable proteins were targeted by a compound with an oncological indication (Table 1). For example, CD33 and SLAF7, together with CD38 (which did not have plasma pQTL data), are targeted by mAbs for multiple myeloma (*37*). The high degree of oncological targets suggests that some of the reported cardiotoxicity (*38*) (e.g., by tyrosine kinase inhibitors such as TIE2) may likely be due to on-target effects, which are therefore resistant to compound improvements. Because inhibiting oncological compounds are used to prevent cancer progression, compounds activating these proteins may not necessarily cause novel neoplasms and might be considered for prevention of cardiac events. Activator compounds may nevertheless influence the growth of any existing undiagnosed neoplasms, and hence, a change in action type should be carefully explored. Aside from the oncological targets, we found many additional repurposing opportunities. For example, the PA2GA (phospholipase A2) inhibitor varespladib failed to show a beneficial CHD effect (*39*), whereas we found an effect of PA2GA on CMR traits and HF.

In the current study, we combined GWAS on UKB-derived CMR measurements of RV and LV structure and functions, with cross-platform dataof the plasma proteome, and perform *cis-*MR of protein effect on these CMR measurements, as well as 56 clinically relevant traits. By leveraging orthogonal lines of evidence on mRNA expression, protein interactions, and drug compound indications and side effects, we were able to identify a robust set of proteins affecting CMR and cardiac outcomes. Genetic analyses were conducted using methods such as BOLT-LMM and BOLT-REML, which account for potential population admixture or relatedness (*40*). Drug target MR analyses were guarded against horizontal pleiotropy by removing variants with either high leverage or heterogeneity statistics (as potential outliers), and a model selection framework was used to apply the MR-Egger correction, which is unbiased in the presence of 100% pleiotropic variants (*41*). Furthermore, results were corrected for multiplicity accounting for the correlation between CMR traits through PC analysis. As described above, we attempted to further limit the false-positive rate by integrating orthogonal nongenetic evidence, such as information on cardiovascular indications and/or side effects. The possibility of false positives driving the presented results was further explored through Kolmogorov-Smirnov tests, comparing the observed *P* value distributions with the *P* value distribution expected when all results are false positive, finding considerable differences (fig. S6) suggesting that most of the findings are true positive.

The following potential limitations deserve consideration. We did not exclude individuals with non-European ancestry, and instead, we accounted for potential population stratification bias through linear mixed models (*40*). Nevertheless, most participants were of European decent, and hence, generalizability of our results should be explored. There are some caveats that suggest that drug target MR results may be more useful as a reliable test of effect direction. This is because drugs that inhibit a target usually do so by modifying its function, not its concentration, whereas genetic variants used in MR analysis usually affect protein expression and therefore concentration. MR estimates are considered to reflect a lifelong exposure, but in the absence of serial assessments, possible changes across age are difficult to explore, as are disease induction times. Similarly, possibly dose-specific effects, where an effect only occurs at sufficiently high drug dosage, are impossible to detect through MR. For these reasons, we suggest that drug target MR offers a robust indication of effect direction but may not directly anticipate the effect magnitude of pharmacologically interfering with a protein, and position our findings as a resource to inform ongoing and future drug trials (*42*). We additionally wish to emphasize that while we have identified proteins that affect (multiple) CMR traits and cardiac outcomes, this study does not provide sufficient evidence to rule out an effect, and future studies will likely identify additional signals. To rule out any potential (harmful) cardiac effect(s), confirmatory noninferiority or equivalence (*43*) testing can be considered, which can formally prove that an effect is sufficiently small to be considered clinically insignificant.

In conclusion, through large-scale analyses of the plasma proteome, and linkage to mRNA expression, protein interactions, and drug compound databases, we have identified a prioritized set of 33 proteins with a robust CMR and cardiac outcome fingerprint. Our analyses provide a detailed overview of potential targets for repurposing or de novo drug development for cardiac therapies.

## MATERIALS AND METHODS

### Quantification of LV and RV CMR traits

The current study sourced information from up to 36,548 UKB subjects who had data on both CMR images and genotyping. To minimize influence of preexisting conditions, we excluded subjects with prevalent diseases (e.g., myocardial infarction, HF, and congenital heart diseases) known to affect the LV or RV traits (see table S2).

The deep-learning methodology (AI-CMR^QC^) to extract LV and RV CMR measurements has been previously described and extensively validated (*7*). Briefly, the fully automated and quality-controlled cardiac analysis tool calculates LV and RV traits from cine short axis and two- and four-chamber acquisitions (figs. S1 and S2 and table S1). Automatic quality control steps consisted of preanalysis checks on image quality (e.g., motion artefacts and erroneous image plane planning) and post-analysis checks on accuracy of the image analysis (e.g., coverage of the segmentations, detected abnormalities in volume, and discrepancies between LV and RV parameters), with automatic detection and removal of outlying observations. This was followed by further manual curation described in Supplementary Methods and fig. S2.

### GWAS of CMR traits

We used genotyped and imputed data as provided by UKB (GRCh37 assembly) (*44*). In brief, samples were genotyped on the Affymetrix BiLEVE and Axiom arrays, with untyped variants imputed using the Haplotype Reference Consortium, 1000 Genomes, and UK10K as reference panels. We excluded samples as recommended by UKB (*44*) and, in addition, used the following sample exclusion criteria: discordant self-reported and genetically inferred sex, and genotypical missingness rate above 0.01. Variant quality control included removing variants with minor allele frequency (MAF) below 0.1%, imputation quality below 0.3, and deviation from the Hardy-Weinberg equilibrium (HWE *P* < 1 × 10^−6^).

Genetic associations with the 16 CMR traits were estimated using BOLT-LMM (*40*), using a mixed-effects model to account for possible cryptic relatedness and population stratification. The BOLT-LMM models were run using default setting and conditional on age at CMR, sex, BSA, SBP, genotype measurement batch, 40 PCs, and assessment center. Please see Supplementary Methods and Results for a comparison with recent CMR GWAS (*34*–*36*) and an evaluation on the influence of BSA and SBP adjustment (fig. S7).

### Genetic heritability of CMR variability

BOLT-REML (*40*) (with default settings, excluding variants with a MAF below 0.1%, HWE *P* < 1 × 10^−6^, and over 1% missingness) was used to estimate narrow-sense genetic heritability (i.e., the proportion of phenotypic variance explained by common variants), as well as the pairwise genetic correlation between the CMR traits.

### Functional and phenotypic annotations and identification of likely causal loci

Lead variants were identified through linkage disequilibrium (LD) clumping within a 1-Mb flanking region, applying a pairwise *r*-squared threshold of 0.001. Putative causal genes were identified through manual curation of locus-view plots, where A.F.S., M.B., J.v.S., and C.F. independently determined the most likely causal genes (Supplementary Locus-view plots). Locus-view plots combined variant-specific CMR associations around each respective lead variant [±250-kbp (kilo–base pair) flanking region] with information on regional genes and their exon structure. These plots were enhanced with an incidence (i.e., Boolean) matrix annotating genes on 23 criteria, including whether the gene was coding, encoded a target for drug compounds with known cardiometabolic (side) effects, and had a *cis-*MR association between any of the considered plasma proteins and a CMR trait, previous associations with cardiometabolic traits sourced from GWAS Catalog (*45*), the presence of mRNA expression or splice sites in cardiac or vascular tissues from GTEx, *trans* protein associations with other CMR loci, and protein-protein interactions between CMR-associated proteins (see Supplementary Locus-view plots and Supplementary Excel file).

### Format normalization of cross-platform pQTLs

Genetic associations with plasma protein concentration were available from the following sources: Somalogic measurements on 3301 participants of the INTERVAL cohort (*9*), Luminex assays on 6861 Framingham participants (*21*), and Olink assays on 30,931 individuals from the SCALLOP consortium (*20*). Framingham provided pQTLs from GWAS of common variants, as well as from exome GWAS, which were concatenated here. For the less than 1% variant overlap between the two Framingham arrays, we selected results with the smallest standard error representing the highest degree of precision. Given the difference in proteomics assays, the subsequent MR analyses were conducted on each individual study. Twenty-one proteins had MR results for more than one study, in which case results from the largest pQTL GWAS were selected.

The GWAS files were normalized using a purpose-built normalization pipeline gwas_norm (see the “Data and materials availability” section of Acknowledgments), standardizing file structures, mapping variants against the same genome assembly, assigning UniProt identifiers, and providing annotations with Variant Effect Predictor (VEP), Polymorphism Phenotyping v2 (PolyPhen), and Combined Annotation Dependent Depletion (CADD).

### MR of plasma protein effects on CMR traits

MR was subsequently used to ascertain the likely causal consequences of protein concentration on the 16 CMR traits. To prevent potential influence of study-specific factors, all drug target MRs were conducted per contributing study, performing separate analyses for SCALLOP, Framingham, and INTERVAL. Specifically, drug target MR was conducted by selecting variants from a 100-kbp window around the *cis* gene known to encode the protein, clumping variants to an LD *R*-squared of 0.40, where residual LD was modeled using a generalized least square (GLS) model (*46*) and a 5000 random sample of UKB participants. To reduce the risk of “weak-instrument bias” (*47*), we selected genetic variants with an *F*-statistic of 15 or higher. Furthermore, because of the absence of sample overlap between protein concentration GWAS and CMR GWAS, any potential weak-instrument bias would act toward a null effect, reducing power rather than increasing type 1 errors.

MR analyses were conducted using the GLS implementation of the inverse-variance weighted (IVW) estimator, as well as with an Egger correction protecting against horizontal pleiotropy (*48*). To minimize the potential influence of horizontal pleiotropy, we excluded variants with large leverage statistic (larger than three times the mean leverage) or outlier statistics (chi-square larger than 10.83) and used the *Q*-statistic to identify possible remaining violations (*49*). Last, a model selection framework was applied to select the most appropriate estimator (IVW or MR-Egger) for each individual exposure-outcome analysis (*41*, *49*). The model selection framework [originally developed by G. Rücker (*50*)] uses the difference in heterogeneity between the IVW *Q*-statistic and the Egger *Q*-statistic to decide which methods provide the best model to describe the available data.

### Protein prioritization

After accounting for multiplicity (see below), we identified proteins with a CMR association, prioritizing results on druggability and on directional concordance. Druggability was determined through linkage with ChEMBL and BNF. The BNF draws information from drug medication inserts, scientific literature, regulatory authorities, and professional bodies and is jointly authored by the British Medical Association and the Royal Pharmaceutical Society. ChEMBL (*22*) was extracted for information on clinically used drug targets (from U.S. Food and Drug Association–approved drugs) and information on drug targets that are in early phase consideration [see Finan *et al.* (*8*)]. Proteins targeted by a marketed compound were referred to as “drugged,” with developmental compounds referred to as “druggable.” The drugged and druggable proteins were additionally annotated by extracting cardiovascular-related indications and side effects from the afformentioned databases (see Supplementary Note 1).

Improvements in drug delivery and development will likely change the current druggability classification. In anticipation of this, we set out to identify proteins with a concordant increasing or decreasing effect. Specifically, results were coded toward the cardiac function or structure improving direction by multiplying estimates for EDV, ESV, EDM, and MVR by −1. A concordant set of prioritized proteins was identified by selecting proteins with at least three CMR associations passing multiple testing correction, which were either all in the beneficial positive direction or all in the detrimental negative direction (i.e., without directionally discordant results). Next, we identified the distance between these concordant proteins and the nearest drugged or druggable protein(s) based on the IntAct (*24*) protein-protein interaction database as modeled in Reactome (accessed April 2021) (*51*). Here, distance reflected the number of protein-protein interactions between the “indexing” concordant protein and the next druggable protein, where a distance of 1 represents a direct link.

The above classification of beneficial versus harmful CMR effect direction is imperfect and simplifies the more complex relationship observed in observational studies. For example, LV-EF has been shown to have a u-shaped association with mortality (*52*). Hence, these heuristic orientations were used as a first filtering step, followed by more direct ascertainment of possible effects on clinical cardiac outcomes through MR. The CMR prioritized set of drugged, druggable, concordant, and nearest drugged/druggable proteins were further pruned on an association with HF, non-ischemic CM, DCM, AF, and/or CHD, using the drug target MR pipeline described before (See Fig. 4 for an overview of the prioritization strategy).

### Drug target phenome-wide scan to anticipate effects of prioritized targets

Next, for CMR prioritized proteins with a cardiac trait association, we evaluated their effects on 56 clinically relevant traits, combining drug target MR with a phenome-wide scan to further inform potential on-target protein effects in future drug development programs.

### Assessing tissue specificity of prioritized targets

The set of prioritized CMR-associated plasma proteins with cardiac effects was annotated by exploring their tissue-specific mRNA expression from the HPA (*23*), sourcing the consensus expression obtained by normalizing TPM (transcripts per million) values from three independent transcriptomics datasets: GTEx (*53*), Fantom5 (*54*), and HPA’s own RNA sequencing experiments (*23*). The normalized human expression data were used to determine a protein tissue specificity (*55*), ranging from 0 (ubiquitous expression across all tissues) to 1 (tissue-specific expression). Differentially overexpressed tissues were identified by comparing tissue-specific expression with average expression, testing against a standard normal quantile of 1.96.

### Quality control and multiple testing

LD score regression (*56*) was used to evaluate the possibility of any remaining bias due to population stratification or cryptic relatedness—finding no cause for concern (table S4). Genetic loci were identified using the traditional genome-wide threshold of 5.00 × 10^−8^ and a conservative threshold of 7.14 × 10^−9^. The latter accounts for multiplicity by performing a Bonferroni correction based on the seven PCs necessary to explain over 90% of the CMR trait variance (fig. S3).

On the basis of the described instrument selection criteria, we had sufficient genetic variants to robustly assess 892 unique proteins. Accounting for the same seven PCs described above and the number of proteins, the MR effect estimates with the CMR traits were evaluated using an α of 7.81 × 10^−6^. The phenome-wide scan drug target analysis of CMR prioritized plasma proteins was evaluated using a multiplicity-corrected α of 1.24 × 10^−5^. Under the null hypothesis, the *P* values of a group of tests follow a uniform distribution between zero and one (*57*). Hence, to additionally explore the potential impact of multiple testing, we performed CMR trait–specific “overall” null hypothesis tests comparing the empirical *P* value distribution (using Kolmogorov-Smirnov tests) with the uniform distribution expected under the null hypothesis (*57*).

Unless otherwise specified, any remaining hypothesis tests were evaluated using an α of 0.05, and all point estimates (OR or mean differences) refer to a unit change of the independent variable, typically one SD in plasma protein level (MR results) or an increase in risk allele (GWAS results). To better illustrate concordance, and only where specified, MR results were orientated toward the cardiac beneficial effect direction by multiplying EDV, ESV, EDM, and MVR MR estimates by −1.

## Supplementary Materials

### This PDF file includes:

Figs. S1 to S16 Tables S1 to S19 Supplementary Methods Supplementary Results Locus-view plots Gene assignment table Supplementary Note
