# Supplementary material for: Druggable proteins influencing cardiac structure and function: Implications for heart failure therapies and cancer cardiotoxicity
Source: Sci Adv. 2023 Apr 26;9(17):eadd4984. doi: 10.1126/sciadv.add4984 (PMC10132758; doi:10.1126/sciadv.add4984)
Supplement: Supplementary file 1 — Figs. S1 to S16 Tables S1 to S19 Supplementary Methods Supplementary Results Locus-view plots Gene assignment table Supplementary Note [file sciadv.add4984_sm.pdf]

Science Advances
AAAS

Supplementary Materials for

Druggable proteins influencing cardiac structure and function: Implications for heart failure therapies and cancer cardiotoxicity

Amand F. Schmidt *et al.*

Corresponding author: Amand F. Schmidt, amand.schmidt@ucl.ac.uk

Sci. Adv. **9**, eadd4984 (2023)
DOI: 10.1126/sciadv.add4984

The PDF file includes:

Figs. S1 to S16
Tables S1 to S19
Supplementary Methods
Supplementary Results
Locus-view plots
Gene assignment table
Supplementary Note

Other Supplementary Material for this manuscript includes the following:

Supplementary file 3

Figures

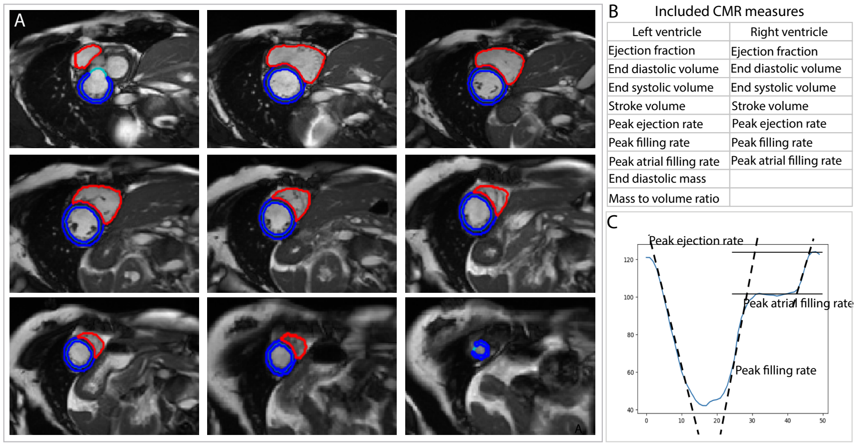

Left ventricle	Right ventricle
Ejection fraction	Ejection fraction
End diastolic volume	End diastolic volume
End systolic volume	End systolic volume
Stroke volume	Stroke volume
Peak ejection rate	Peak ejection rate
Peak filling rate	Peak filling rate
Peak atrial filling rate	Peak atrial filling rate
End diastolic mass	
Mass to volume ratio	

Figure S1: A) Examples of automatic segmentation of the left (blue) and right (red) ventricle from basal to apical short axis cine CMR (base to apex). **B**) The included left and right ventricular CMR measures calculated using a deep-learning algorithm. **C**) A graphical explanation of the calculation of the peak ejection rate, peak filling rate and peak atrial filling rate.

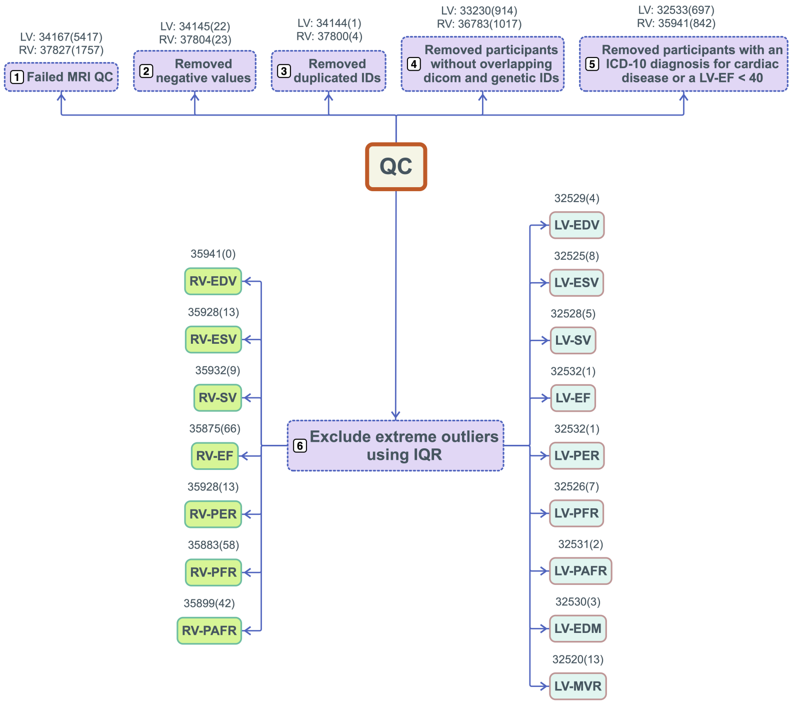

Figure S2: A flowchart of the various quality control steps applied to the available CMR data; see the supplementary methods section for further details.

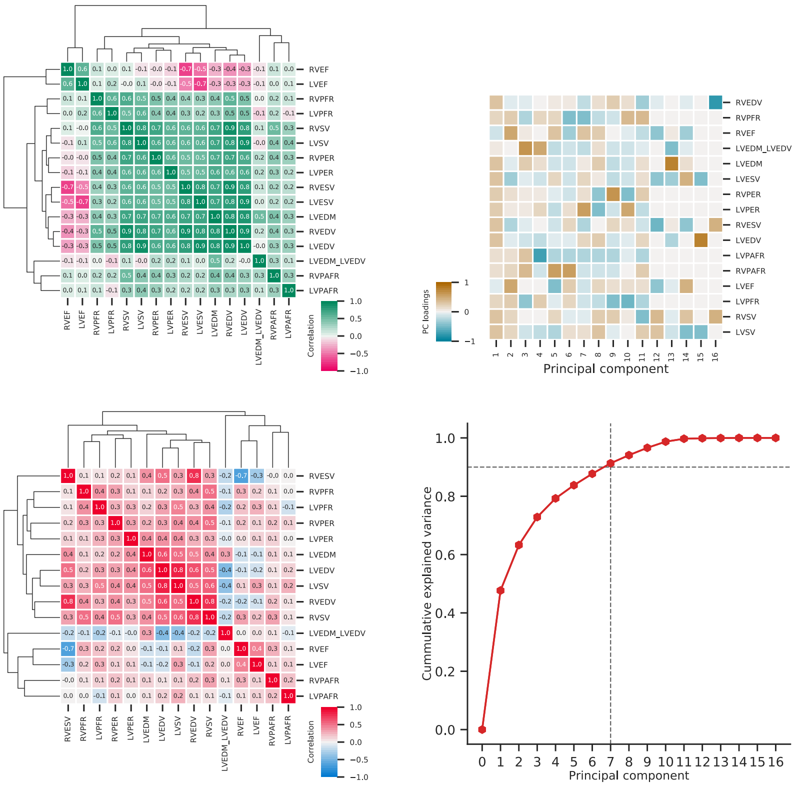

Figure S3: Left column Spearman's pairwise correlation: *top left*, between the phenotypic cardiac MRI measurements (based on a n=36548 UKB sample), and *bottom left*, between the genetic association with these cardiac MRIs. Right column principal components analysis (PCA): *bottom right*, the component loadings, and *top right*, the cumulative explained variance by principal component. The heatmap margins were ordered by hierarchical clustering of the Euclidean distance.

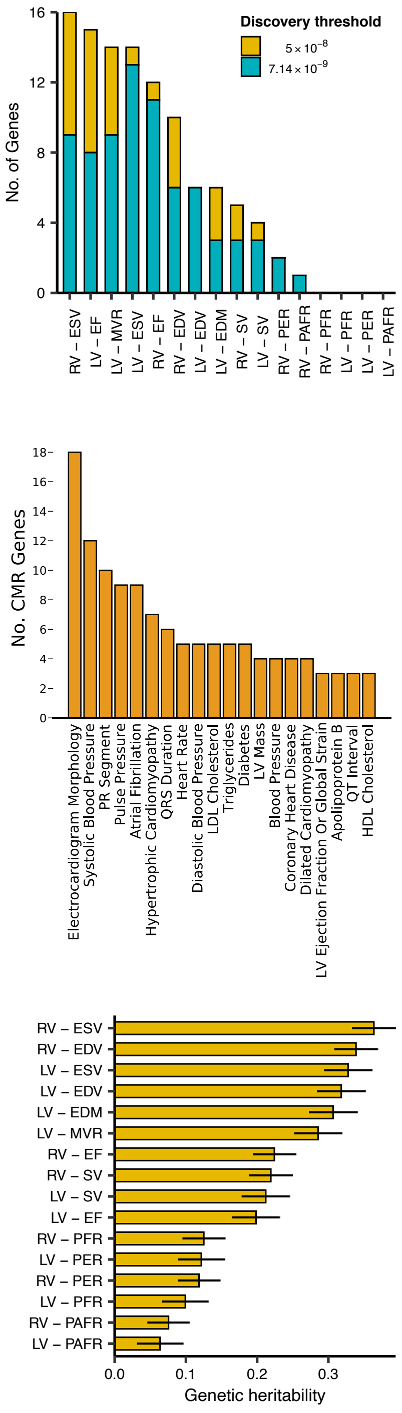

Figure S4: Aggregated GWAS results and genetic heritability estimates. Top panel depicts the number of significant putative causal genes per CMR trait and significance threshold. The middle panel provides the top 20 most frequent trait associations of the discovered CMR genes, sourced from GWAS catalog. The bottom panel provides the genetic heritability estimates with 95% confidence intervals. Results are based on an analysis of 36,548 subjects, a star indicates significant p-value at an alpha of 0.05.

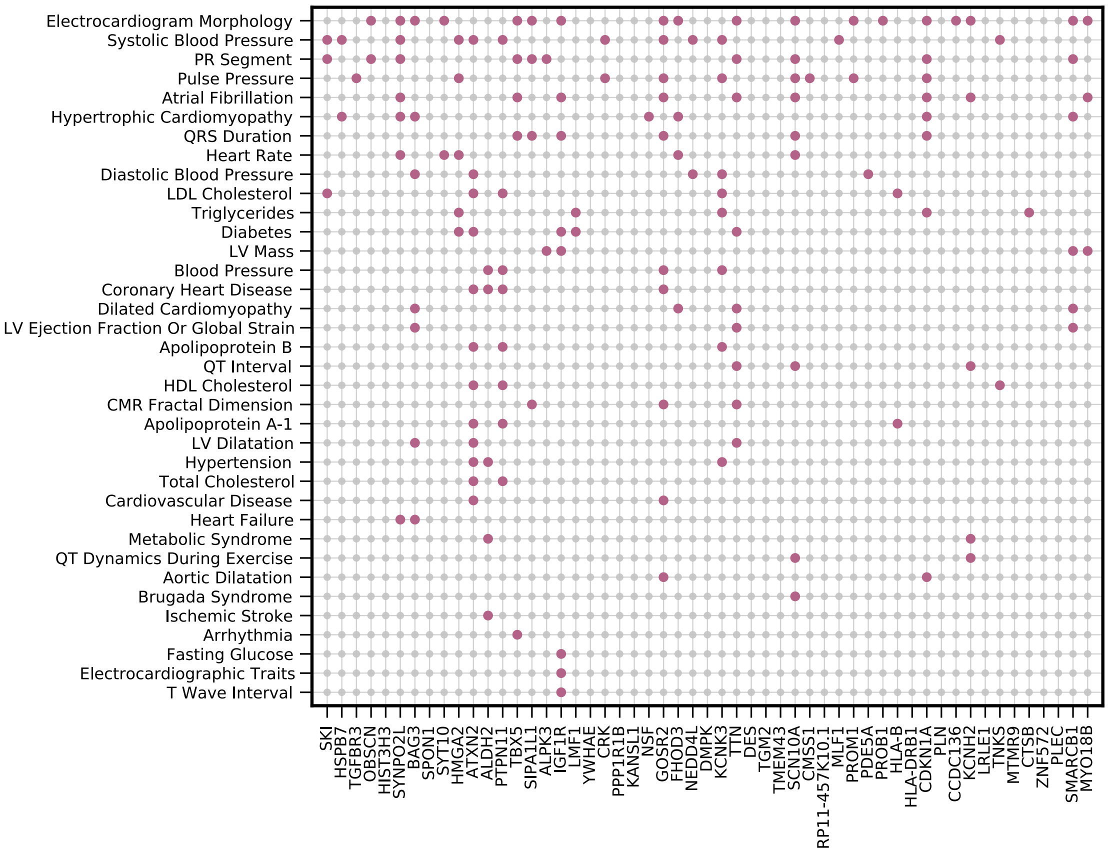

Figure S5: An incidence matrix linking the CMR loci to previous cardio-metabolic GWAS associations from GWAS catalog.

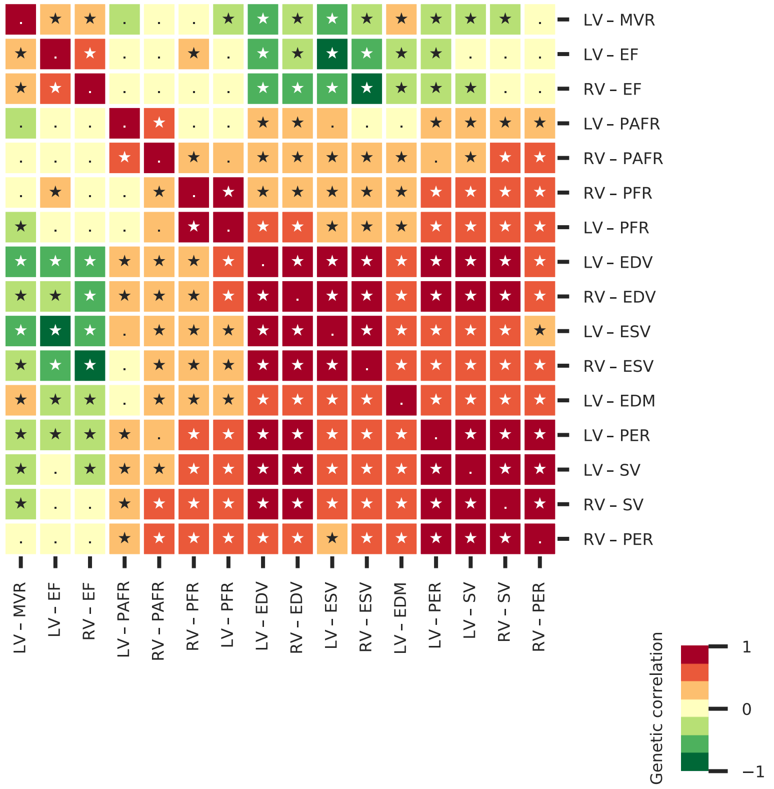

Figure S6: The pairwise genetic correlation between CMR traits. LV, left-ventricle; RV, right-ventricle; EDV, end-diastolic volume; ESV, end-systolic volume; SV, stroke volume; EF, ejection fraction; PER, peak ejection rate; PAFR/PFR, peak (atrial) filling rate; EDM, end-diastolic mass; MVR, ratio between end diastolic mass and volume. Results are based on an analysis of 36,548 subjects. Star-annotated cells indicate significant associations at an alpha of 0.05.

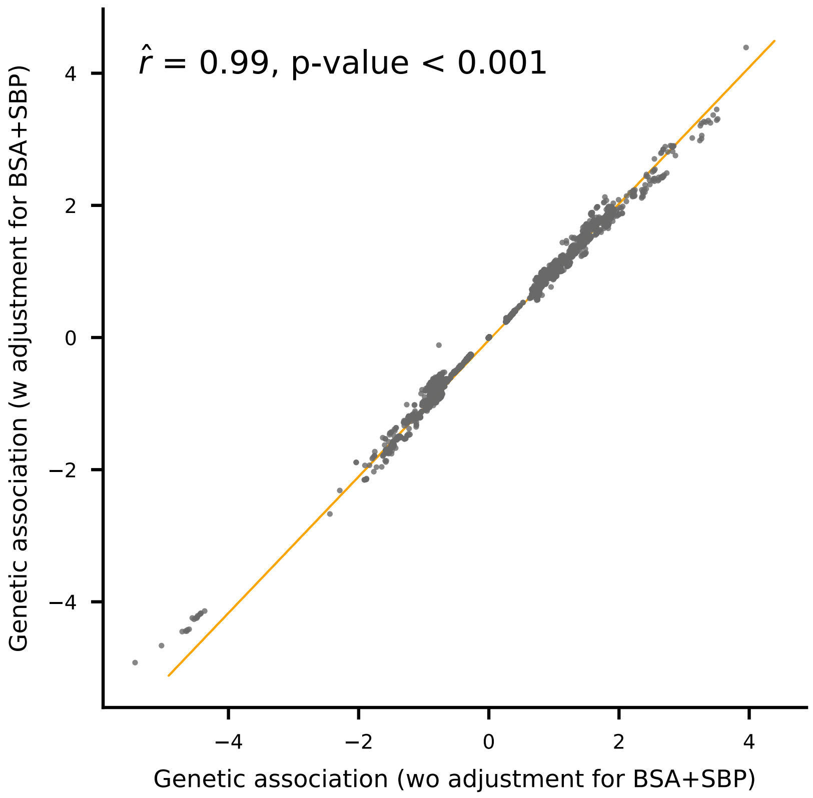

Figure S7: The correlation between genetic associations (mean difference) with and without adjustment for body surface area (BSA) and systolic blood pressure (SBP). $\hat{r}$ represents the Pearson correlation coefficient.

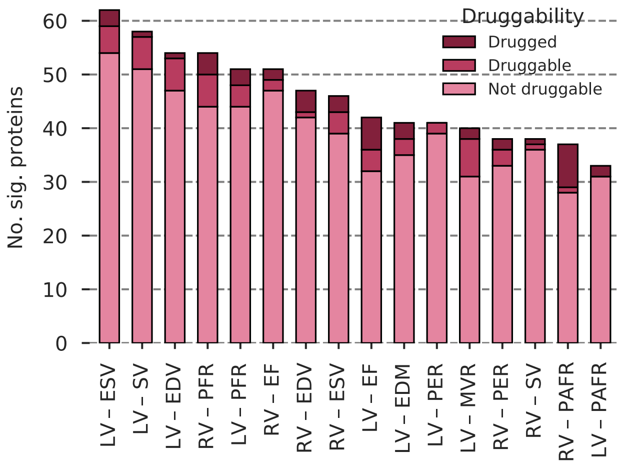

Figure S8: The frequency of plasma protein associations per CMR trait stratified on druggability status. Results are based on *cis* Mendelian randomization analyses and represent estimates that passed a multiplicity corrected p-value threshold of 7.81×10-6. Druggability was based on an update version of Finan *et al* [8]

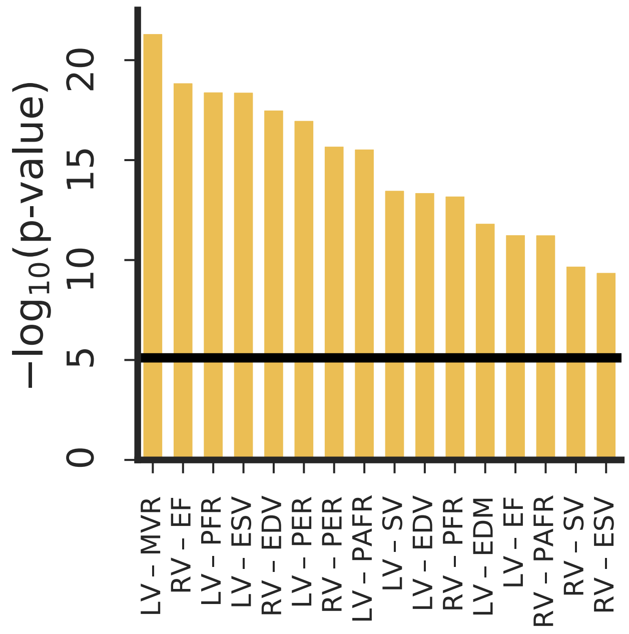

Figure S9: Trait specific Kolmogorov-Smirnoff null-hypothesis tests comparing the empirical p-value distribution to the uniform distribution expected when results are driven by false positive results. The horizontal line indicates the multiplicity corrected alpha of 7.81×10^{-6}.

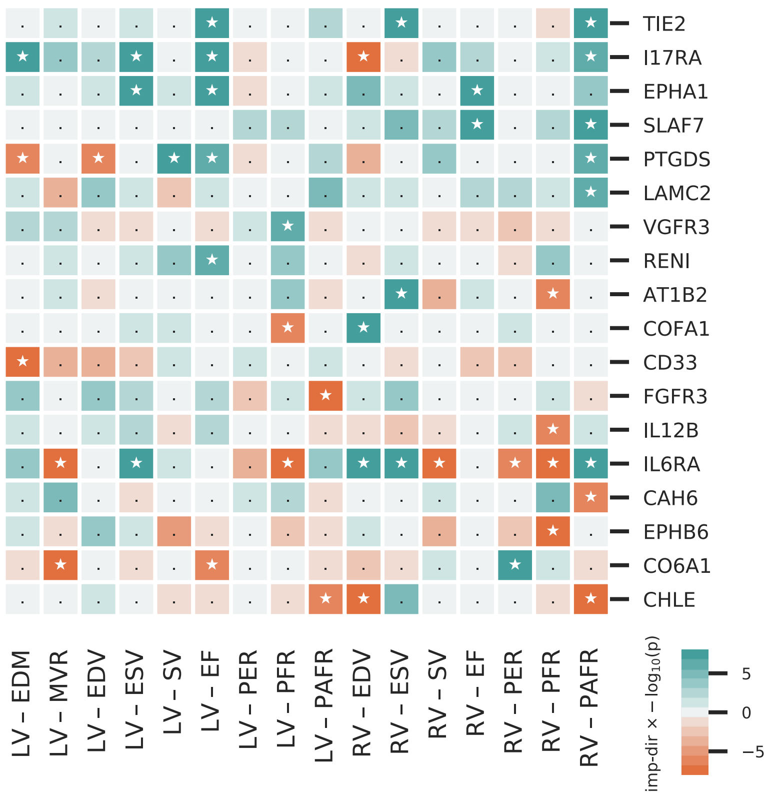

Figure S10: Drug target MR of *drugged* plasma protein concentration (per SD) effects on CMR traits orientated toward the cardiac function improving direction (‘imp-dir‘). Cells are coloured by truncated p-value (max 8) multiplied by effect direction.

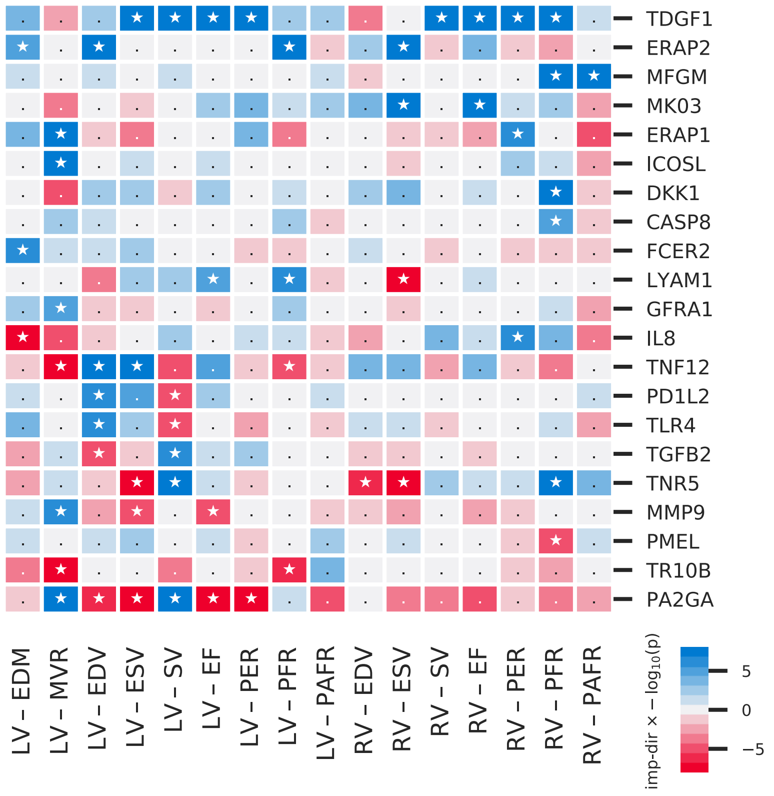

Figure S11: Drug target MR of *druggable* plasma protein concentration (per SD) effects on CMR traits orientated toward the cardiac function improving direction (‘imp-dir‘). Cells are coloured by truncated p-value (max 8) multiplied by effect direction.

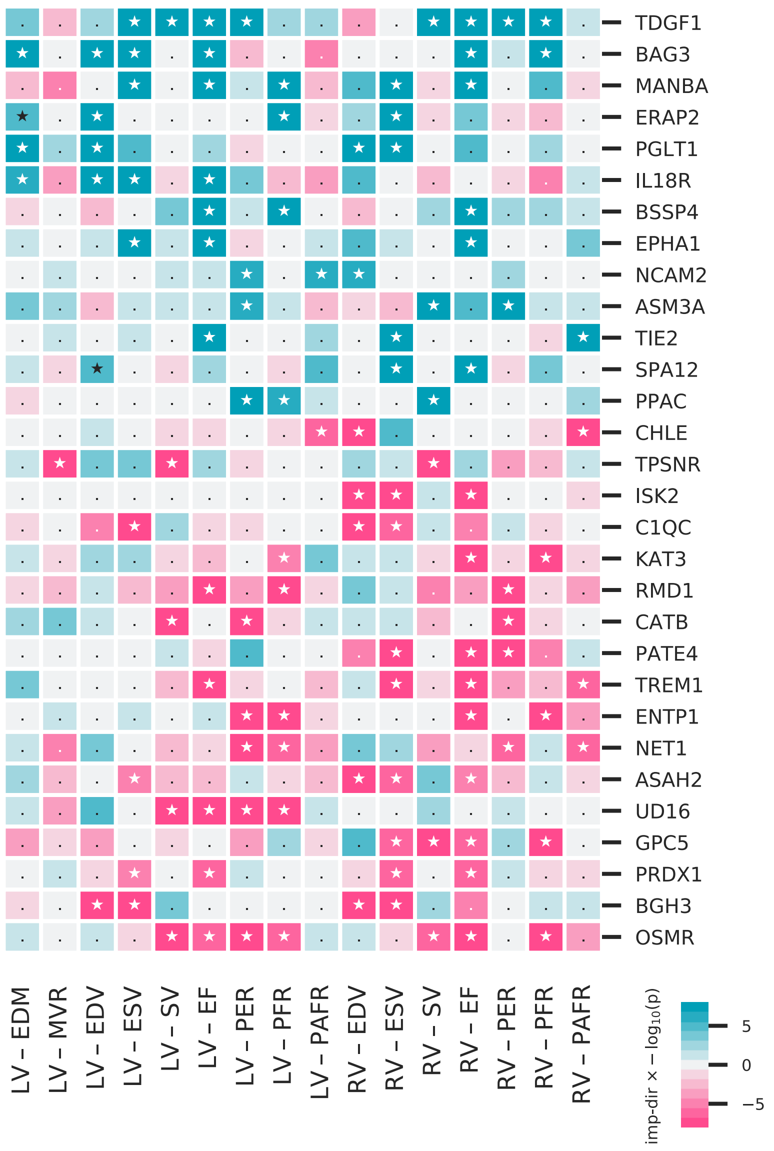

Figure S12: Concordant protein effects on CMR traits orientated toward the cardiac function improving direction (‘imp-dir‘). Depicting proteins affecting three or more CMR traits, in a concordant risk increasing or decreasing direction. Cells are coloured by truncated p-value (max 8) multiplied by effect direction.

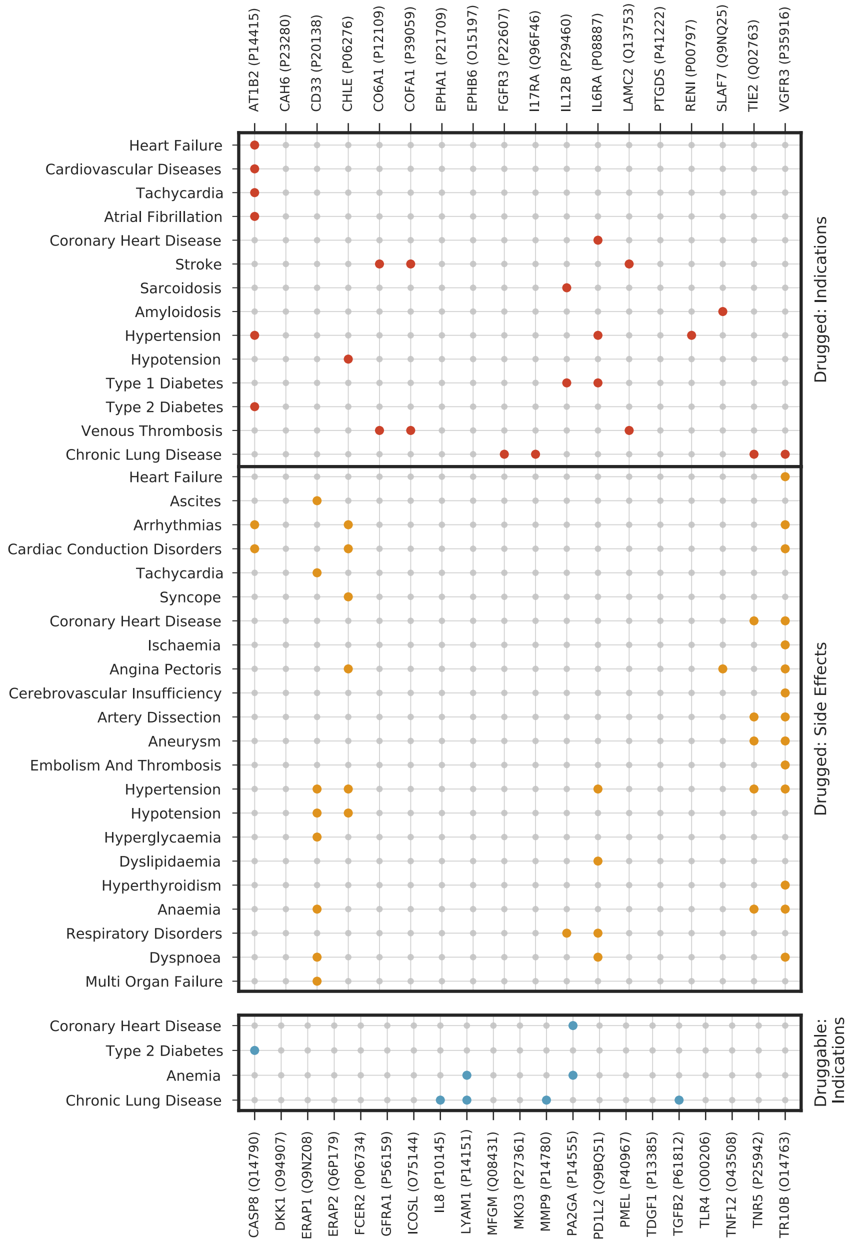

Figure S13: Incidence matrix of cardio-metabolic related indications and side effects of compounds targeting drugged and druggable proteins associated with CMR traits. Coloured dots represents an established link between the compound and trait; data were extracted from BNF and ChEMBL. Nomenclature: proteins are referred to by their uniprot entry name to differentiate them from the encoding genes. Protein names and uniprot ids are provided on the top and bottom x-axis, with indication or side effect trait on the y-axis

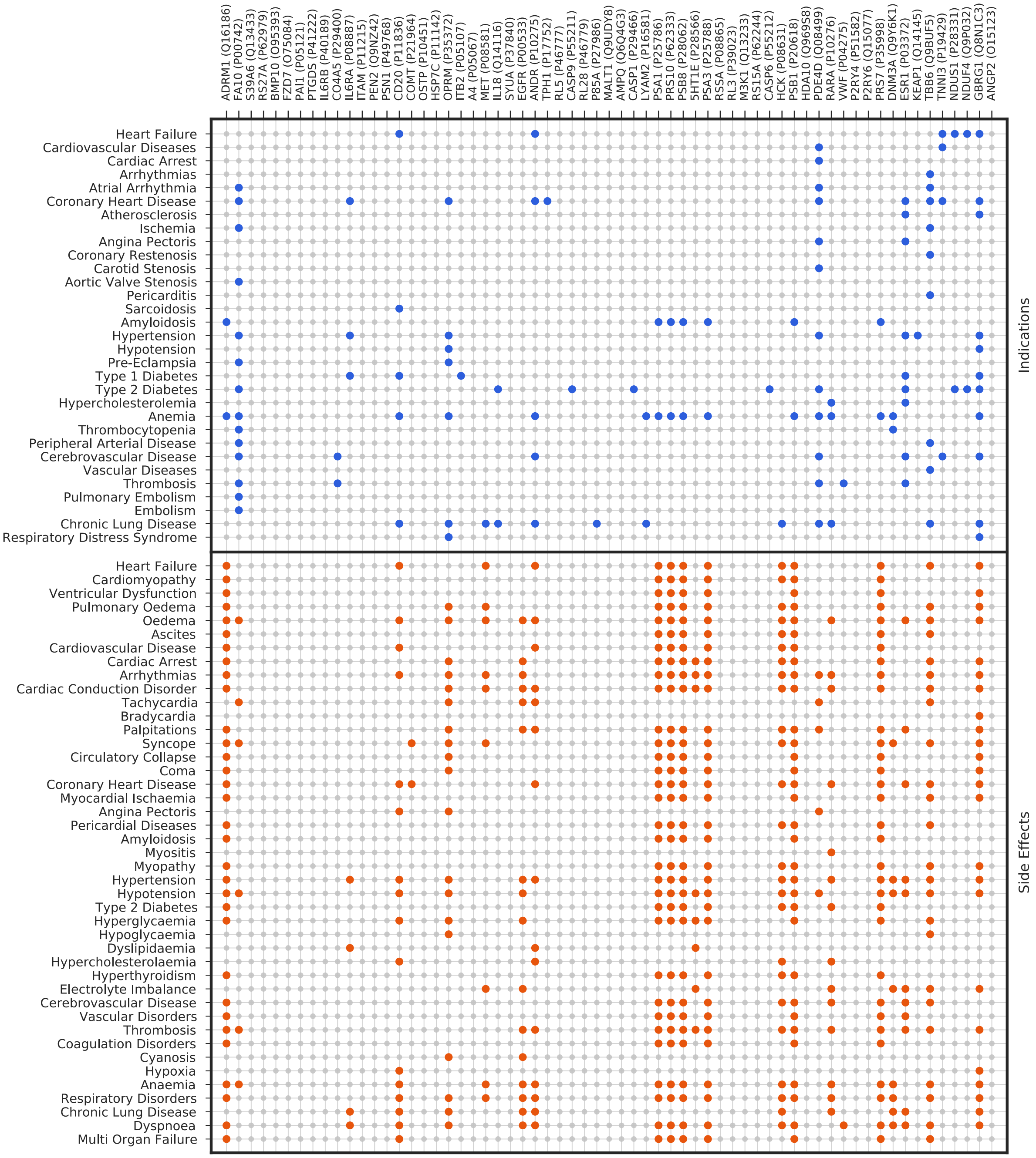

Figure S14: Incidence matrix of cardiovascular related indication and side effect of *indirectly* drugged or druggable protein. These *indirectly* drugged or druggable proteins were identified through a Reactome pathway analysis, identifying druggable proteins that had an protein-protein interaction with a *concordant* CMR associated indexing protein. Coloured dots represents an established link between the compound and trait. Nomenclature: proteins are referred to by their uniprot entry name to differentiate them from the encoding genes. Protein name and uniprot id are provided on the top x-axis, with indication or side effect trait on the y-axis.

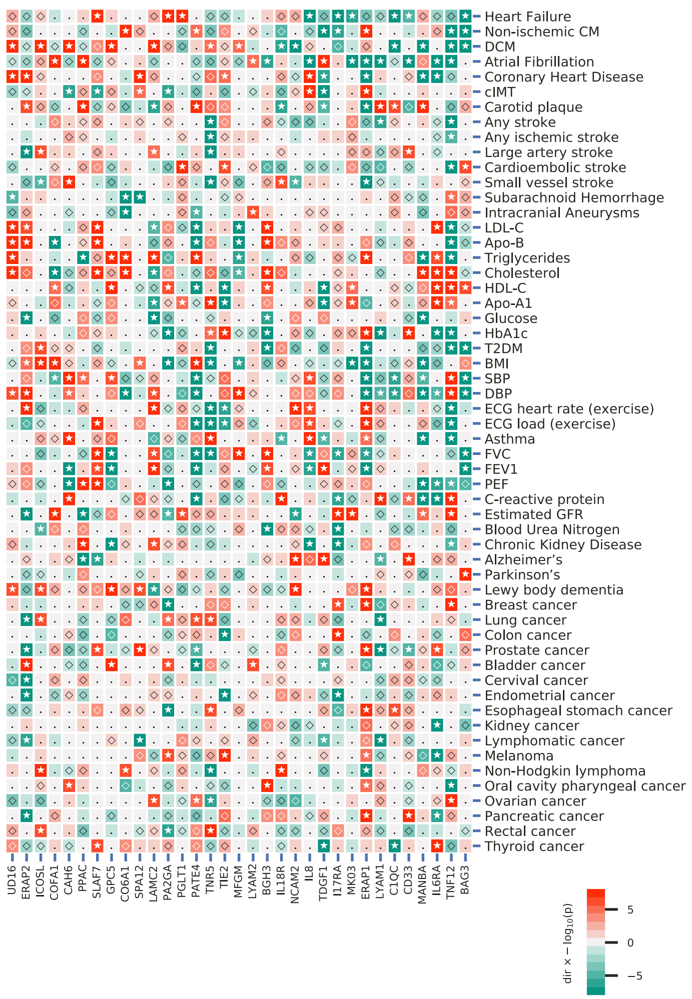

Figure S15: A phenome-wide scan of CMR proteins associated with one or more cardiac outcome. N.b. Proteins were curated on having a multiplicity corrected p-value $> 1.29 \times 10^{-5}$ with one or more of the following cardiac traits: Heart Failure (HR), Dilated Cardiomyopathy (DCM), Non-ischemic CM, Atrial Fibrillation (AF), or Coronary Heart Disease (CHD). P-value passing the 0.05 threshold are indicated by square rotated about 90 degrees, with stars indicating results passing the mentioned multiplicity corrected threshold. Cells were coloured by effect direction times $-\log_{10}$(p-value); where p-values were truncated to 8.

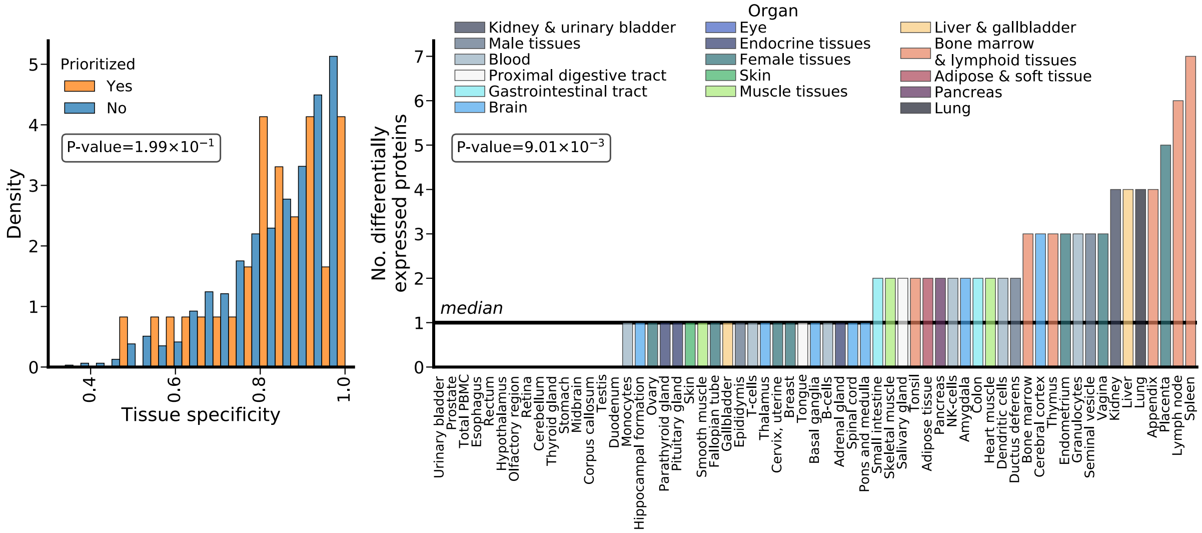

Figure S16: *Left:* The tissue specificity of plasma proteins prioritized on having an robust CMR and cardiac outcome association; The p-value is based on a Mann-Whitney test. *Right:* The number of tissues with differentially more mRNA expression of the CMR and cardiac outcome prioritized plasma proteins; with χ^2-test for equal number differentially expressed proteins per tissue. Data were sourced from the Human Protein Atlas.

Tables

Measurement	Abbreviation	Unit	Pathological consequence of higher values
Stroke volume	SV	ml	Beneficial
Peak ejection rate	PER	ml/s	Beneficial
Peak atrial filling rate	PAFR	ml/s	Beneficial
Peak filling rate	PFR	ml/s	Beneficial
End systolic volume	ESV	ml	Harmful
Ejection fraction	EF	%	Beneficial
End diastolic volume	EDV	ml	Harmful
End diastolic mass	EDM	gram	Harmful
Ratio of EDM and EDV	MVR	gram/ml	Harmful

Table S1: CMR measurements and pathological consequence

Table S2: UK biobank fields used to exclude participants with possible pre-existing cardiac disease.

**Excluded phenotype**	**UKB field ID/ICD-10 code**
Myocardial infarction	42000
Acute transmural myocardial infarction of anterior wall	I21.0
Acute transmural myocardial infarction of inferior wall	I21.1
Acute transmural myocardial infarction of other sites	I21.2
Acute transmural myocardial infarction of unspecified site	I21.3
Acute subendocardial myocardial infarction	I21.4
Acute myocardial infarction	I21.9
Subsequent myocardial infarction of anterior wall	I22.0
Subsequent myocardial infarction of inferior wall	I22.1
Subsequent myocardial infarction of other sites	I22.8
Subsequent myocardial infarction of unspecified site	I22.9
Acute ischaemic heart disease	I24.9
Old myocardial infarction	125.2
Pulmonary embolism with mention of acute cor pulmonale	I26.0
Primary pulmonary hypertension	I27.0
Kyphoscoliotic heart disease	I27.1
Other secondary pulmonary hypertension	I27.2
Nonrheumatic tricuspid (valve) stenosis	I36.0
Nonrheumatic tricuspid (valve) insufficiency	I36.1
Other nonrheumatic tricuspid valve disorders	I36.8
Nonrheumatic tricuspid valve disorder	I36.9
Pulmonary valve stenosis	I37.0
Pulmonary valve insufficiency	I37.1
Pulmonary valve stenosis with insufficiency	I37.2
Other pulmonary valve disorders	I37.8
Pulmonary valve disorder	I37.9
Congestive heart failure	I50.0
Left ventricular failure	I50.1
Heart failure	I50.9
Hypertensive heart disease with (congestive) heart failure	I11.0
Dilated cardiomyopathy	I42.0
Obstructive hypertrophic cardiomyopathy	I42.1
Other hypertrophic cardiomyopathy	I42.2
Endocardial fibroelastosis	I42.4
Other restrictive cardiomyopathy	I42.5
Alcoholic cardiomyopathy	I42.6
Cardiomyopathy due to drugs and other external agents	I42.7
Other cardiomyopathies	I42.8
Congenital malformation of cardiac chambers and connexions	Q20.9
Common arterial trunk	Q20.0
Discordant ventriculoarterial connexion	Q20.3
Ventricular septal defect	Q21.0
Atrial septal defect	Q21.1
Atrioventricular septal defect	Q21.2
Tetralogy of Fallot	Q21.3
Aortopulmonary septal defect	Q21.4
Other congenital malformations of cardiac septa	Q21.8
Congenital malformation of cardiac septum	Q21.9
Congenital pulmonary valve stenosis	Q22.1
Congenital pulmonary valve insufficiency	Q22.2
’Ebstein”s anomaly’	Q22.5
Other congenital malformations of tricuspid valve	Q22.8
Congenital stenosis of aortic valve	Q23.0
Congenital insufficiency of aortic valve	Q23.1
Congenital mitral insufficiency	Q23.3
Other congenital malformations of aortic and mitral valves	Q23.8
Congenital malformation of aortic and mitral valves	Q23.9

Table S2: UK biobank fields used to exclude participants with possible pre-existing cardiac disease. *(continued)*

**Excluded phenotype**	**UKB field ID/ICD-10 code**
Chronic obstructive pulmonary disease with acute lower respiratory infection	J44.0
Chronic obstructive pulmonary disease with acute exacerbation	J44.1
Other specified chronic obstructive pulmonary disease	J44.8
’MacLeod”s syndrome’	J43.0
Panlobular emphysema	J43.1
Centrilobular emphysema	J43.2
Other emphysema	J43.8
Emphysema	J43.9
LVEF <40%	Automated CMR data used

Table S3: Characteristics of UK biobank subjects used in the CMR GWAS.

Characteristics	Total (n=36548)	Female (n=18879)	Male (n=17093)	Missing
Age at time of CMR (years)	63.9 (7.6)	63.3 (7.4)	64.5 (7.7)	0
Ethnicity				679
Caucasian	34872 (97.0%)	18336 (97.1%)	16536 (96.8%)	
Black	209 (0.6%)	111 (0.6%)	98 (0.6%)	
Asian	351 (1.0%)	122 (0.6%)	229 (1.3%)	
Other	437 (1.3%)	267 (1.5%)	170 (1.0%)	
Height (cm)	169.2 (89.2)	162.8 (6.2)	176.1 (6.6)	0
Weight (kg)	76.0 (15.1)	69.1 (13.1)	83.7 (13.3)	0
**Cardiovacular risk factors**				
BMI (kg/m2)	26.5 (4.4)	26.1 (4.7)	26.9 (3.9)	0
Systolic bloodpressure (mmHg)	138.2 (18.4)	135.1 (19.0)	141.6 (17.1)	3525
Diastolic bloodpressure (mmHg)	78.6 (10.0)	76.8 (9.9)	80.5 (9.7)	2525
Heart rate (bpm)	62.5 (10.2)	63.5 (9.9)	61.5 (10.4)	577
Cholesterol (mmol/l)	5.7 (1.1)	5.9 (1.1)	5.6 (1.1)	2340
Diabetes	1835 (5.1%)	669 (3.5%)	1166 (6.8%)	248
Smoking				248
Past	12227 (33.7%)	5891 (30.9%)	6336 (36.7)	
Current	1254 (3.5%)	541 (2.8%)	713 (4.1%)	
Alcohol intake- daily/almost daily	6104 (16.8%)	2531 (13.3%)	3573 (20.7%)	248
Moderate activity (MET minutes pw)	826.7 (1120.3)	832.7 (1108.8)	820.6 (1133.9	5191
CMR parameters				
LV–EDV (ml)	143.2 (33.2)	125.1 (22.2)	163.5 (31.7)	4078
LV–ESV (ml)	58.8 (18.5)	48.9 (12.2)	69.8 (18.1)	4082
LV–EDM (g)	80.9 (22.4)	65.9 (12.6)	97.6 (18.9)	4077
LV–MVR (g/ml)	0.6 (0.1)	0.5 (0.1)	0.6 (0.1)	4087
LV–SV (ml)	84.4 (18.9)	76.2 (14.5)	93.7 (19.1)	4079
LV–EF (%)	59.4 (6.2)	61.0 (5.8)	57.5 (5.9)	4075
LV–PFR (ml/s)	321.1 (98.3)	303.4 (86.6)	341.0 (106.5)	4081
LV–PER (ml/s)	377.8 (111.9)	323.5 (84.8)	438.4 (106.5)	4075
LV–PAFR (ml/s)	251.8 (106.5)	225.6 (94.1)	280.9 (111.9)	4076
RV–EDV (ml)	153.7 (38)	131.0 (24.6)	178.7 (34.3)	676
RV–ESV (ml)	64.8 (22.0)	52.2 (14.1)	78.6 (20.9)	689
RV–SV (ml)	88.9 (20.5)	78.8 (15.2)	100.1 (19.8)	685
RV–EF (%)	58.5 (6.8)	60.4 (6.4)	56.3 (6.5)	742
RV–PFR (ml/s)	308.4 (96.3)	282.9 (82.8)	336.6 (102.1)	734
RV–PER (ml/s)	396.3 (111.2)	341.7 (83.1)	456.5 (107.1)	689
RV–PAFR (ml/s)	293.7 (108.6)	260.4 (92.3)	163.9 (31.4)	718

General:
Number represents mean/counts with between brackets the standard deviation or percentage. BMI= body mass index; CMR= cardiac magnetic resonance; EDV= end-diastolic volume; EDM= end-diastolic mass; EF= ejection fraction; ESV= end-systolic volume; LV= left ventricle; MET= metabolic equivalent of task; MVR= mass to volume ratio; PAFR= peak atrial filling rate; PER= peak ejection rate; PFR= peak filling rate; RV= right ventricle; SV= stroke volume.

Table S4: Assessing the potential for bias due to cryptic relatedness and population stratification.

**CMR trait**	**LD-Score intercept (SE)**
RV - EDV	1.02 (0.01)
RV - EF	1.00 (0.01)
RV - ESV	1.02 (0.01)
RV - PAFR	1.00 (0.01)
RV - PER	0.99 (0.01)
RV - PFR	1.01 (0.01)
RV - SV	1.00 (0.01)
LV - EDM	1.01 (0.01)
LV - MVR	1.01 (0.01)
LV - EDV	1.01 (0.01)
LV - EF	0.99 (0.01)
LV - ESV	1.00 (0.01)
LV - PAFR	1.00 (0.01)
LV - PER	1.01 (0.01)
LV - PFR	1.01 (0.01)
LV - SV	1.00 (0.01)

General:
In the absence of bias the intercept is expected to be 1.00. SE: standard error.

Table S5: Top hits from a cardiac MRI (CMR) GWAS on sixteen left and right-ventricle (LV, RV) traits.

Marker name	Chr	BP (build 37)	Nearest gene	Putative causal gene	CMR trait	EF/NEA	MD	p-value
rs2503715	1	2144107	C1orf86 (ENSG00000162585)	SKI (ENSG00000157933)	LV – MVR	A/G	-0.005	3.08×10^{-8}
rs28579893	1	16347534	HSPB7 (ENSG00000173641)	HSPB7 (ENSG00000173641)	LV – EF	A/G	0.410	2.54×10^{-17}
rs28579893	1	16347534	HSPB7 (ENSG00000173641)	HSPB7 (ENSG00000173641)	LV – ESV	A/G	-0.924	4.58×10^{-15}
rs945425	1	16348412	HSPB7 (ENSG00000173641)	HSPB7 (ENSG00000173641)	RV – EF	T/C	0.301	3.62×10^{-10}
rs6604061	1	92328613	RN7SL653P (ENSG00000239794)	TGFBR3 (ENSG00000069702)	RV – ESV	T/A	0.689	2.18×10^{-8}
rs3738685	1	228556788	OBSCN (ENSG00000154358)	OBSCN (ENSG00000154358)	LV – ESV	C/T	-0.676	6.30×10^{-9}
rs3738685	1	228556788	OBSCN (ENSG00000154358)	OBSCN (ENSG00000154358)	RV – EF	C/T	0.306	9.29×10^{-11}
rs12126782	1	228613648	HIST3H3 (ENSG00000168148)	HIST3H3 (ENSG00000168148)	RV – ESV	T/G	-0.910	7.04×10^{-13}
rs1314982	2	26922062	KCNK3 (ENSG00000171303)	KCNK3 (ENSG00000171303)	RV – EDV	G/A	1.224	3.50×10^{-8}
rs13394970	2	26929282	KCNK3 (ENSG00000171303)	KCNK3 (ENSG00000171303)	RV – ESV	T/G	0.761	5.06×10^{-10}
rs746173377	2	179442292	RP11-171I2.5 (ENSG00000271011)	TTN (ENSG00000155657)	RV – ESV	TA/T	-1.090	1.43×10^{-8}
rs12988307	2	179490478	RP11-171I2.4 (ENSG00000271141)	TTN (ENSG00000155657)	LV – ESV	T/C	1.522	1.58×10^{-27}
rs2562845	2	179514433	RP11-171I2.3 (ENSG00000271401)	TTN (ENSG00000155657)	LV – EF	T/C	-0.539	4.98×10^{-21}
rs2042995	2	179558366	RP11-171I2.1 (ENSG00000267784)	TTN (ENSG00000155657)	RV – EDV	T/C	1.620	1.77×10^{-12}
rs2042995	2	179558366	RP11-171I2.1 (ENSG00000267784)	TTN (ENSG00000155657)	RV – EF	T/C	-0.331	7.78×10^{-10}
rs2042995	2	179558366	RP11-171I2.1 (ENSG00000267784)	TTN (ENSG00000155657)	RV – ESV	T/C	1.190	4.76×10^{-17}
rs111692972	2	179669931	CCDC141 (ENSG00000163492)	TTN (ENSG00000155657)	LV – EDM	C/T	1.809	1.81×10^{-16}
rs111692972	2	179669931	CCDC141 (ENSG00000163492)	TTN (ENSG00000155657)	LV – EDV	C/T	3.371	4.88×10^{-17}
rs6747402	2	179759434	RNU7-104P (ENSG00000238542)	TTN (ENSG00000155657)	RV – EDV	G/A	-1.299	2.29×10^{-11}
rs6747402	2	179759434	RNU7-104P (ENSG00000238542)	TTN (ENSG00000155657)	RV – ESV	G/A	-0.762	2.05×10^{-10}
rs55844607	2	179770998	RNU7-104P (ENSG00000238542)	TTN (ENSG00000155657)	RV – SV	A/G	1.147	4.65×10^{-8}
rs35009641	2	220432957	INHA (ENSG00000123999)	DES (ENSG00000175084)	LV – MVR	G/A	-0.008	3.36×10^{-8}
rs200775399	3	14291003	RP11-536I6.2 (ENSG00000255021)	TMEM43 (ENSG00000170876)	LV – EF	CTTTT/C	-0.271	2.18×10^{-8}
rs11710541	3	14291679	RP11-536I6.2 (ENSG00000255021)	TMEM43 (ENSG00000170876)	LV – ESV	T/C	0.804	9.02×10^{-12}
rs2170454	3	14294549	RP11-536I6.2 (ENSG00000255021)	TMEM43 (ENSG00000170876)	RV – ESV	T/C	-0.990	1.49×10^{-16}
rs10865722	3	14306782	RP11-536I6.2 (ENSG00000255021)	TMEM43 (ENSG00000170876)	RV – EF	G/T	-0.375	1.15×10^{-14}
rs34234056	3	14418444	RNA5SP124 (ENSG00000199609)	TMEM43 (ENSG00000170876)	RV – ESV	A/G	0.655	4.52×10^{-8}
rs6795970	3	38766675	SCN10A (ENSG00000185313)	SCN10A (ENSG00000185313)	RV – PAFR	A/G	-4.711	4.44×10^{-10}
rs57848867	3	99779984	FILIP1L (ENSG00000168386)	CMSS1 (ENSG00000184220)	RV – EDV	A/T	-1.223	4.03×10^{-10}
rs57848867	3	99779984	FILIP1L (ENSG00000168386)	CMSS1 (ENSG00000184220)	RV – ESV	A/T	-0.674	2.29×10^{-8}
rs111487301	3	109560677	RP11-457K10.1 (ENSG00000242029)	RP11-457K10.1 (ENSG00000242029)	LV – SV	ATG/A	0.735	2.28×10^{-8}
rs11710570	3	158056654	RP11-113A11.1 (ENSG00000241723)	MLF1 (ENSG00000178053)	RV – EDV	T/C	-1.206	4.82×10^{-10}
rs9844502	3	158298615	MLF1 (ENSG00000178053)	MLF1 (ENSG00000178053)	RV – ESV	C/T	-0.915	1.97×10^{-14}

Table S5: Top hits from a cardiac MRI (CMR) GWAS on sixteen left and right-ventricle (LV, RV) traits. *(continued)*

Marker name	Chr	BP (build 37)	Nearest gene	Putative causal gene	CMR trait	EF/NEA	MD	p-value
rs9864508	3	158298703	MLF1 (ENSG00000178053)	MLF1 (ENSG00000178053)	RV – EF	T/C	0.284	3.44×10^{-10}
rs10939649	4	16034589	RNU6-350P (ENSG00000251758)	PROM1 (ENSG00000007062)	LV – EF	C/T	0.330	3.00×10^{-10}
rs34463475	4	120438654	PDE5A (ENSG00000138735)	PDE5A (ENSG00000138735)	RV – ESV	CT/C	-0.764	1.17×10^{-8}
rs12514667	5	138718825	SLC23A1 (ENSG00000170482)	PROB1 (ENSG00000228672)	RV – ESV	C/G	-0.695	4.61×10^{-8}
rs11242465	5	138762305	DNAJC18 (ENSG00000170464)	PROB1 (ENSG00000228672)	LV – ESV	T/G	-0.681	4.92×10^{-8}
rs4835729	5	138763329	DNAJC18 (ENSG00000170464)	PROB1 (ENSG00000228672)	LV – EF	A/G	0.299	9.11×10^{-10}
rs4835730	5	138763807	DNAJC18 (ENSG00000170464)	PROB1 (ENSG00000228672)	RV – EF	T/C	0.328	1.27×10^{-11}
rs34504162	6	31310626	HLA-B (ENSG00000234745)	HLA-B (ENSG00000234745)	LV – MVR	A/G	-0.004	3.09×10^{-8}
rs9265867	6	31312790	HLA-B (ENSG00000234745)	HLA-B (ENSG00000234745)	RV – PER	A/T	-5.435	1.95×10^{-9}
rs111721712	6	31315407	HLA-B (ENSG00000234745)	HLA-B (ENSG00000234745)	LV – SV	C/CT	0.702	5.36×10^{-9}
rs281874821	6	32631426	XXbac-BPG254F23.6 (ENSG00000241287)	HLA-DRB1 (ENSG00000196126)	LV – EF	G/A	-0.327	9.09×10^{-9}
rs7774130	6	36623756	RNU1-88P (ENSG00000238554)	CDKN1A (ENSG00000124762)	LV – EDM	C/T	-0.760	4.24×10^{-10}
rs730506	6	36645968	CDKN1A (ENSG00000124762)	CDKN1A (ENSG00000124762)	LV – MVR	G/C	-0.007	1.51×10^{-18}
rs147436240	6	36648064	CDKN1A (ENSG00000124762)	CDKN1A (ENSG00000124762)	LV – EF	CGCGT/C	-0.339	7.97×10^{-9}
rs11153730	6	118667522	SLC35F1 (ENSG00000196376)	PLN (ENSG00000198523)	LV – EDV	T/C	-1.114	4.57×10^{-9}
rs74640693	6	118684824	SLC35F1 (ENSG00000196376)	PLN (ENSG00000198523)	LV – MVR	A/T	-0.008	5.46×10^{-9}
rs73238147	7	128469917	FLNC (ENSG00000128591)	CCDC136 (ENSG00000128596)	LV – ESV	T/C	0.976	1.63×10^{-9}
rs3918226	7	150690176	NOS3 (ENSG00000164867)	KCNH2 (ENSG00000055118)	RV – EDV	C/T	2.051	1.65×10^{-8}
rs3918226	7	150690176	NOS3 (ENSG00000164867)	KCNH2 (ENSG00000055118)	RV – SV	C/T	1.272	5.93×10^{-9}
rs2980441	8	8095129	ALG1L13P (ENSG00000253981)	LRLE1 (ENSG00000268955)	RV – EF	G/C	-0.261	9.87×10^{-9}
rs66645639	8	9591977	MIR597 (ENSG00000207701)	TNKS (ENSG00000173273)	LV – EF	TTACTC/T	0.267	1.56×10^{-8}
rs7823349	8	10998630	AF131215.4 (ENSG00000254556)	MTMR9 (ENSG00000104643)	RV – EF	G/T	0.266	5.44×10^{-9}
rs13268810	8	11797430	OR7E161P (ENSG00000206014)	CTSB (ENSG00000164733)	LV – EF	A/T	-0.265	1.53×10^{-8}
rs12541595	8	125857359	LINC00964 (ENSG00000249816)	ZNF572 (ENSG00000180938)	LV – EF	G/T	-0.348	2.66×10^{-12}
rs200712209	8	125858538	LINC00964 (ENSG00000249816)	ZNF572 (ENSG00000180938)	LV – ESV	GA/G	0.879	4.63×10^{-13}
rs34866937	8	125859850	LINC00964 (ENSG00000249816)	ZNF572 (ENSG00000180938)	RV – EF	G/A	-0.284	6.42×10^{-9}
rs11784619	8	145013775	MIR661 (ENSG00000207574)	PLEC (ENSG00000178209)	LV – EF	G/A	-0.563	1.61×10^{-8}
rs11786896	8	145018354	MIR661 (ENSG00000207574)	PLEC (ENSG00000178209)	RV – EF	C/T	-0.702	2.67×10^{-11}
rs11786896	8	145018354	MIR661 (ENSG00000207574)	PLEC (ENSG00000178209)	RV – ESV	C/T	1.805	8.29×10^{-11}
rs3812629	10	75407290	SYNPO2L (ENSG00000166317)	SYNPO2L (ENSG00000166317)	LV – EDM	G/A	-0.823	3.36×10^{-8}
rs10886511	10	121307823	RGS10 (ENSG00000148908)	BAG3 (ENSG00000151929)	RV – ESV	G/A	0.793	2.39×10^{-9}
rs72840788	10	121415685	BAG3 (ENSG00000151929)	BAG3 (ENSG00000151929)	LV – EDV	G/A	1.551	2.83×10^{-11}
rs72840788	10	121415685	BAG3 (ENSG00000151929)	BAG3 (ENSG00000151929)	LV – EF	G/A	-0.581	3.98×10^{-25}

Table S5: Top hits from a cardiac MRI (CMR) GWAS on sixteen left and right-ventricle (LV, RV) traits. *(continued)*

Marker name	Chr	BP (build 37)	Nearest gene	Putative causal gene	CMR trait	EF/NEA	MD	p-value
rs72840788	10	121415685	BAG3 (ENSG00000151929)	BAG3 (ENSG00000151929)	LV – ESV	G/A	1.463	1.12×10^{-26}
rs72840788	10	121415685	BAG3 (ENSG00000151929)	BAG3 (ENSG00000151929)	RV – EF	G/A	-0.544	1.21×10^{-22}
rs72840788	10	121415685	BAG3 (ENSG00000151929)	BAG3 (ENSG00000151929)	RV – ESV	G/A	1.303	6.41×10^{-19}
rs1609342	11	14022662	SPON1 (ENSG00000152268)	SPON1 (ENSG00000152268)	RV – ESV	C/T	-0.684	1.32×10^{-8}
rs61884835	11	14052348	SPON1 (ENSG00000152268)	SPON1 (ENSG00000152268)	LV – ESV	T/C	-0.667	2.42×10^{-9}
rs6488162	12	33593127	SYT10 (ENSG00000110975)	SYT10 (ENSG00000110975)	LV – SV	T/C	-0.782	1.61×10^{-10}
rs1585897	12	66383320	HMGA2 (ENSG00000149948)	HMGA2 (ENSG00000149948)	RV – EDV	C/A	1.104	1.79×10^{-8}
rs4766578	12	111904371	ATXN2 (ENSG00000204842)	ATXN2 (ENSG00000204842)	RV – EDV	T/A	-1.913	9.14×10^{-23}
rs4766578	12	111904371	ATXN2 (ENSG00000204842)	ATXN2 (ENSG00000204842)	RV – SV	T/A	-0.869	1.32×10^{-13}
rs597808	12	111973358	U7 (ENSG00000272215)	ATXN2 (ENSG00000204842)	LV – SV	A/G	-0.750	4.57×10^{-10}
rs653178	12	112007756	U7 (ENSG00000272215)	ATXN2 (ENSG00000204842)	LV – EDV	C/T	-1.295	1.20×10^{-11}
rs653178	12	112007756	U7 (ENSG00000272215)	ATXN2 (ENSG00000204842)	RV – ESV	C/T	-1.062	8.93×10^{-19}
rs11513729	12	112273499	MAPKAPK5-AS1 (ENSG00000234608)	ALDH2 (ENSG00000111275)	RV – PER	C/T	3.952	4.85×10^{-9}
rs11066320	12	112906415	PTPN11 (ENSG00000179295)	PTPN11 (ENSG00000179295)	RV – EDV	A/G	-1.593	9.96×10^{-16}
rs11066320	12	112906415	PTPN11 (ENSG00000179295)	PTPN11 (ENSG00000179295)	RV – SV	A/G	-0.777	8.12×10^{-11}
rs1895606	12	114833384	TBX5-AS1 (ENSG00000255399)	TBX5 (ENSG00000089225)	RV – ESV	C/T	-0.680	1.76×10^{-8}
rs34647020	14	71796137	RP1-261D10.1 (ENSG00000259079)	SIPA1L1 (ENSG00000197555)	LV – MVR	G/GT	-0.004	4.86×10^{-8}
rs56864281	15	85357649	ALPK3 (ENSG00000136383)	ALPK3 (ENSG00000136383)	LV – MVR	C/A	0.007	4.71×10^{-24}
rs7164817	15	85379544	SNORA25 (ENSG00000200991)	ALPK3 (ENSG00000136383)	LV – EF	T/C	-0.274	2.08×10^{-8}
rs12907646	15	85403496	SNORA25 (ENSG00000200991)	ALPK3 (ENSG00000136383)	LV – ESV	G/A	-0.897	2.36×10^{-11}
rs6598541	15	99271135	MIR4714 (ENSG00000264480)	IGF1R (ENSG00000140443)	LV – EDM	A/G	-0.611	1.88×10^{-8}
rs7166287	15	99273075	MIR4714 (ENSG00000264480)	IGF1R (ENSG00000140443)	LV – MVR	C/T	-0.004	1.12×10^{-10}
rs11350493	16	951372	LA16c-306A4.2 (ENSG00000260316)	LMF1 (ENSG00000103227)	LV – MVR	TG/T	-0.004	2.29×10^{-8}
rs12452627	17	1302472	YWHAE (ENSG00000108953)	YWHAE (ENSG00000108953)	LV – MVR	G/A	-0.007	2.05×10^{-11}
rs2302455	17	1374195	MYO1C (ENSG00000197879)	CRK (ENSG00000167193)	LV – EDV	G/A	1.783	3.09×10^{-9}
rs2302455	17	1374195	MYO1C (ENSG00000197879)	CRK (ENSG00000167193)	LV – ESV	G/A	1.031	5.52×10^{-9}
rs11869286	17	37813856	STARD3 (ENSG00000131748)	PPP1R1B (ENSG00000131771)	LV – EDM	G/C	-0.619	1.74×10^{-8}
rs2696421	17	43667635	DND1P1 (ENSG00000264070)	KANSL1 (ENSG00000120071)	LV – MVR	A/G	-0.005	5.82×10^{-12}
rs567642046	17	44358811	ARL17B (ENSG00000228696)	KANSL1 (ENSG00000120071)	LV – EDM	G/A	-0.941	1.87×10^{-11}
rs17692129	17	44793283	RPS7P11 (ENSG00000213326)	NSF (ENSG00000073969)	LV – MVR	C/T	0.004	2.50×10^{-10}
rs17608766	17	45013271	RP11-156P1.2 (ENSG00000262633)	GOSR2 (ENSG00000108433)	RV – EDV	T/C	-1.752	2.14×10^{-10}
rs17608766	17	45013271	RP11-156P1.2 (ENSG00000262633)	GOSR2 (ENSG00000108433)	RV – ESV	T/C	-1.129	3.33×10^{-11}
rs772756888	18	34219777	SNORD112 (ENSG00000252078)	FHOD3 (ENSG00000134775)	RV – EF	G/GTT	-0.331	4.03×10^{-10}

Table S5: Top hits from a cardiac MRI (CMR) GWAS on sixteen left and right-ventricle (LV, RV) traits. *(continued)*

Marker name	Chr	BP (build 37)	Nearest gene	Putative causal gene	CMR trait	EF/NEA	MD	p-value
rs7230600	18	55938204	RP11-718I15.1 (ENSG00000267743)	NEDD4L (ENSG00000049759)	LV – ESV	G/A	-0.693	3.66×10^{-9}
rs10871753	18	55956865	RP11-845C23.2 (ENSG00000267504)	NEDD4L (ENSG00000049759)	LV – EF	G/T	-0.267	6.77×10^{-9}
rs35226705	19	46301456	RSPH6A (ENSG00000104941)	DMPK (ENSG00000104936)	RV – EDV	A/C	1.086	2.04×10^{-8}
rs56096557	19	46310895	RSPH6A (ENSG00000104941)	DMPK (ENSG00000104936)	RV – ESV	A/C	-0.813	8.08×10^{-11}
rs10421891	19	46315809	RSPH6A (ENSG00000104941)	DMPK (ENSG00000104936)	LV – EDV	A/G	-1.186	2.09×10^{-9}
rs10421891	19	46315809	RSPH6A (ENSG00000104941)	DMPK (ENSG00000104936)	LV – ESV	A/G	-0.775	2.60×10^{-11}
rs4811602	20	36849088	KIAA1755 (ENSG00000149633)	TGM2 (ENSG00000198959)	RV – SV	G/A	-0.658	2.52×10^{-8}
rs5760054	22	24161717	DERL3 (ENSG00000099958)	SMARCB1 (ENSG00000099956)	LV – ESV	C/T	-1.027	2.07×10^{-13}
rs5760054	22	24161717	DERL3 (ENSG00000099958)	SMARCB1 (ENSG00000099956)	LV – MVR	C/T	0.006	8.45×10^{-13}
rs5760054	22	24161717	DERL3 (ENSG00000099958)	SMARCB1 (ENSG00000099956)	LV – EF	C/T	0.387	1.34×10^{-11}
rs133890	22	26160161	MYO18B (ENSG00000133454)	MYO18B (ENSG00000133454)	LV – MVR	C/G	-0.004	2.33×10^{-9}

General:
Putative causal genes were identified through consensus based on extensive annotations, see online methods. CMR: cardiac MRI; Chr: chromosome; BP: base pair location; EA: effect allele; NEA: non-effect allele; EAF: effect allele frequence; MD: mean difference coded towards the EA; SE: standard error of the MD.

Table S6: CMR genes encoding druggable and drugged proteins

Putative causal gene	Protein	Target type	Druggability	Compound	Molecule type	Drug mechanism
CDKN1A (ENSG00000124762)	CDN1A (P38936)		Druggable			
CTSB (ENSG00000164733)	CATB (P07858)		Druggable			
DMPK (ENSG00000104936)	DMPK (Q09013)		Druggable			
HLA-B (ENSG00000234745)	1B48|1B81|1B67|1B42|1B07|1B73 (P30486)		Druggable			
OBSCN (ENSG00000154358)	OBSCN (Q5VST9)		Druggable			
PROM1 (ENSG00000007062)	PROM1 (O43490)		Druggable			
PTPN11 (ENSG00000179295)	PTN11 (Q06124)		Druggable	Tno-155	Small molecule	Inhibitor
TGFBR3 (ENSG00000069702)	TGBR3 (Q03167)		Druggable			
TGM2 (ENSG00000198959)	TGM2 (P21980)		Druggable			
TTN (ENSG00000155657)	TITIN (Q8WZ42)		Druggable			
ALDH2 (ENSG00000111275)	ALDH2 (P05091)	Single Protein	Drugged	Disulfiram	Small molecule	Inhibitor
HLA-DRB1 (ENSG00000196126)	2B1F (P01911)	Single Protein	Drugged	Lym-1	Antibody	Other
	2B11 (P04229)	Protein Family	Drugged	Lym-1	Antibody	Other
	2B1G (Q29974)	Protein Family	Drugged	Lym-1	Antibody	Other
	2B1E (Q9GIY3)	Protein Family	Drugged	Lym-1	Antibody	Other
				Plovamer Acetate	Small molecule	Modulator
				Plovamer Acetate	Small molecule	Modulator
				Plovamer Acetate	Small molecule	Modulator
				Plovamer Acetate	Small molecule	Modulator
				Apolizumab	Antibody	Inhibitor
				Apolizumab	Antibody	Inhibitor
				Apolizumab	Antibody	Inhibitor
				Apolizumab	Antibody	Inhibitor
IGF1R (ENSG00000140443)	IGF1R (P08069)	Single Protein	Drugged	Mecasermin Rinfabate	Protein	Agonist
				Mecasermin	Protein	Agonist
				Aew-541	Small molecule	Inhibitor
				Pl-225B	Small molecule	Inhibitor
				Xl-228	Small molecule	Inhibitor
				Axl-1717	Small molecule	Inhibitor
				Bms-754807	Small molecule	Inhibitor
				Insm-18	Small molecule	Inhibitor
				Kw-2450	Small molecule	Inhibitor
				Linsitinib	Small molecule	Inhibitor
				Figitumumab	Antibody	Antagonist
				Ganitumab	Antibody	Antagonist
				Teprotumumab	Antibody	Antagonist
				Cixutumumab	Antibody	Antagonist
				Dalotuzumab	Antibody	Antagonist
				Robatumumab	Antibody	Antagonist
				Ave-1642	Antibody	Antagonist
				Biib-022	Antibody	Inhibitor
				Istiratumab	Antibody	Inhibitor
				Conteltinib	Small molecule	Inhibitor
KCNH2 (ENSG00000055118)	KCNH2 (Q12809)	Protein Family	Drugged	Dofetilide	Small molecule	Blocker
				Ibutilide Fumarate	Small molecule	Blocker
				Amiodarone Hydrochloride	Small molecule	Blocker
				Sotalol Hydrochloride	Small molecule	Blocker
				Azd7009	Small molecule	Blocker
				Azd1305	Small molecule	Blocker

Table S6: CMR genes encoding druggable and drugged proteins *(continued)*

Putative causal gene	Protein	Target type	Druggability	Compound	Molecule type	Drug mechanism
				Vernakalant Hydrochloride	Small molecule	Blocker
				Dalfampridine	Small molecule	Blocker
				Guanidine Hydrochloride	Small molecule	Blocker
				Nerispirdine	Small molecule	Blocker
				Tedisamil	Small molecule	Blocker
				Amifampridine	Small molecule	Blocker
				Amifampridine Phosphate	Small molecule	Blocker
KCNK3 (ENSG00000171303)	KCNK3 (O14649)	Single Protein	Drugged	Desflurane	Small molecule	Opener
				Doxapram Hydrochloride	Small molecule	Blocker
				Sevoflurane	Small molecule	Opener
				Halothane	Small molecule	Opener
				Isoflurane	Small molecule	Opener
				Enflurane	Small molecule	Opener
PDE5A (ENSG00000138735)	PDE5A (O76074)	Single Protein	Drugged	Tadalafil	Small molecule	Inhibitor
				Vardenafil Hydrochloride	Small molecule	Inhibitor
				Sildenafil Citrate	Small molecule	Inhibitor
				Avanafil	Small molecule	Inhibitor
				Udenafil	Small molecule	Inhibitor
				Ibudilast	Small molecule	Inhibitor
				Gisadenafil	Small molecule	Inhibitor
				Pf-00489791	Small molecule	Inhibitor
				Pentoxifylline	Small molecule	Inhibitor
				Dipyridamole	Small molecule	Inhibitor
SCN10A (ENSG00000185313)	SCNAA (Q9Y5Y9)	Protein Family	Drugged	Dibucaine Hydrochloride	Small molecule	Blocker
				Dsp-2230	Small molecule	Blocker
				Afacifenacin	Small molecule	Blocker
				Pf-04531083	Small molecule	Blocker
				Quinidine Polygalacturonate	Small molecule	Blocker
				Ropivacaine Hydrochloride	Small molecule	Blocker
				Phenacemide	Small molecule	Blocker
				Benoxinate Hydrochloride	Small molecule	Blocker
				Chloroprocaine Hydrochloride	Small molecule	Blocker
				Mexiletine Hydrochloride	Small molecule	Blocker
				Riluzole	Small molecule	Blocker
				Hexylcaine Hydrochloride	Small molecule	Blocker
				Phenazopyridine Hydrochloride	Small molecule	Blocker
				Quinidine Gluconate	Small molecule	Blocker
				Primidone	Small molecule	Blocker
				Ethotoin	Small molecule	Blocker
				Disopyramide Phosphate	Small molecule	Blocker
				Dronedarone Hydrochloride	Small molecule	Blocker
				Dyclonine Hydrochloride	Small molecule	Blocker
				Quinidine Sulfate	Small molecule	Blocker
				Prilocaine	Small molecule	Blocker
				Phenytoin Sodium	Small molecule	Blocker
				Topiramate	Small molecule	Blocker
				Merethoxylline Procaine	Small molecule	Blocker
				Procaine Hydrochloride	Small molecule	Blocker
				Mephenytoin	Small molecule	Blocker
				Carbamazepine	Small molecule	Blocker
				Articaine Hydrochloride	Small molecule	Blocker
				Tetracaine	Small molecule	Blocker
				Erlosamide	Small molecule	Blocker
				Mepivacaine Hydrochloride	Small molecule	Blocker
				Moricizine Hydrochloride	Small molecule	Blocker
				Etidocaine Hydrochloride	Small molecule	Blocker
				Orphenadrine Hydrochloride	Small molecule	Blocker
				Orphenadrine Citrate	Small molecule	Blocker
				Procainamide Hydrochloride	Small molecule	Blocker
				Propafenone Hydrochloride	Small molecule	Blocker

Table S6: CMR genes encoding druggable and drugged proteins *(continued)*

Putative causal gene	Protein	Target type	Druggability	Compound	Molecule type	Drug mechanism
				Proparacaine Hydrochloride	Small molecule	Blocker
				Propoxycaine Hydrochloride	Small molecule	Blocker
				Rufinamide	Small molecule	Blocker
				Oxcarbazepine	Small molecule	Blocker
				Phenytoin	Small molecule	Blocker
				Prilocaine Hydrochloride	Small molecule	Blocker
				Fosphenytoin Sodium	Small molecule	Blocker
				Indecainide Hydrochloride	Small molecule	Blocker
				Lamotrigine	Small molecule	Blocker
				Lidocaine	Small molecule	Blocker
				Lidocaine Hydrochloride	Small molecule	Blocker
				Tocainide Hydrochloride	Small molecule	Blocker
				Zonisamide	Small molecule	Blocker
				Eslicarbazepine Acetate	Small molecule	Blocker
				Evenamide	Small molecule	Blocker
				Nktr-171	Small molecule	Blocker
				Irampanel	Small molecule	Blocker
				Nerispirdine	Small molecule	Blocker
				Ralfinamide	Small molecule	Blocker
				Tetracaine Hydrochloride	Small molecule	Blocker
				Eslicarbazepine	Small molecule	Blocker
				Encainide Hydrochloride	Small molecule	Blocker
				Dichlorobenzyl Alcohol	Small molecule	Blocker
				Cenobamate	Small molecule	Inhibitor
TNKS (ENSG00000173273)	TNKS1 (O95271)		Drugged	2X-121	Small molecule	Inhibitor

General:
Data were extracted from British National Formulary, and ChEMBL. Genes are provided with ensemlb id, and proteins with uniprot id.

Table S7: Cardiometabolic drug indications of compounds targeting proteins encoded by CMR genes

Putative causal gene	Protein	Target type	Compound	Drug mechanism	Drug indication
IGF1R (ENSG00000140443)	IGF1R (P08069)	Single Protein	Mecasermin	Agonist	Diabetes Mellitus
KCNH2 (ENSG00000055118)	KCNH2 (Q12809)	Protein Family	Dofetilide	Blocker	Atrial Fibrillation
				Blocker	Heart Failure
					Arrhythmias, Cardiac
			Amiodarone Hydrochloride	Blocker	Amyloidosis
					Atrial Fibrillation
					Heart Failure
			Sotalol Hydrochloride	Blocker	Atrial Fibrillation
					Heart Failure
			Azd7009	Blocker	Atrial Fibrillation
			Azd1305	Blocker	Atrial Fibrillation
					Atrial Flutter
			Vernakalant Hydrochloride	Blocker	Atrial Fibrillation
					Atrial Flutter
			Dalfampridine	Blocker	Stroke
			Tedisamil	Blocker	Arrhythmias, Cardiac
KCNK3 (ENSG00000171303)	KCNK3 (O14649)	Single Protein	Sevoflurane	Opener	Heart Diseases
					Aneurysm
					Coronary Disease
			Isoflurane	Opener	Heart Diseases
PDE5A (ENSG00000138735)	PDE5A (O76074)	Single Protein	Tadalafil	Inhibitor	Heart Failure
					Essential Hypertension
					Diabetes Mellitus
					Hypertension
					Stroke
					Lung Diseases, Interstitial
			Sildenafil Citrate	Inhibitor	Stroke
					Ischemia
					Emphysema
					Hypertension
					Pre-Eclampsia
					Heart Failure
			Udenafil	Inhibitor	Heart Diseases
					Hypertension
					Heart Failure
			Ibudilast	Inhibitor	Lung Diseases, Obstructive
					Pneumonia
			Gisadenafil	Inhibitor	Pulmonary Disease, Chronic Obstructive
			Pf-00489791	Inhibitor	Hypertension
			Pentoxifylline	Inhibitor	Anemia
					Coronary Disease
					Fibrosis
					Sarcoidosis
					Acute Coronary Syndrome
					Cardiovascular Diseases
			Dipyridamole	Inhibitor	Hypertension
					Anemia
					Coronary Disease
					Heart Diseases
					Carotid Stenosis
					Ischemia
					Angina, Stable
					Coronary Artery Disease
					Thrombosis
					Stroke
					Adjunct to oral anticoagulation for prophylaxis of thromboembolism associated with prosthetic heart valves

Table S7: Cardiometabolic drug indications of compounds targeting proteins encoded by CMR genes *(continued)*

Putative causal gene	Protein	Target type	Compound	Drug mechanism	Drug indication
					Secondary prevention of ischaemic stroke and transient ischaemic attacks
SCN10A (ENSG00000185313)	SCNAA (Q9Y5Y9)	Protein Family	Quinidine Polygalacturonate	Blocker	Arrhythmias, Cardiac
			Primidone	Blocker	Stroke
			Disopyramide Phosphate	Blocker	Atrial Fibrillation
					Heart Failure
			Dronedarone Hydrochloride	Blocker	Atrial Fibrillation
			Topiramate	Blocker	Ischemia
					Diabetes Mellitus
					Hypertension
			Procainamide Hydrochloride	Blocker	Atrial Fibrillation
					Heart Failure
			Propafenone Hydrochloride	Blocker	Atrial Fibrillation
					Heart Failure
			Phenytoin	Blocker	Anemia
			Lidocaine	Blocker	Hypertension
					Thromboembolism
					Heart Diseases
					Heart Arrest
					Pre-Eclampsia
					Stroke
					Arrhythmias, Cardiac
			Lidocaine Hydrochloride	Blocker	Hypertension
					Diabetes Mellitus

General:
Data were extracted from British National Formulary, and ChEMBL. Genes are provided with ensemlb id, and proteins with uniprot id.

Table S8: Cardiometabolic drug side-effects of compounds targeting proteins encoded by CMR genes

Putative causal gene	Protein	Target type	Compound	Drug mechanism	Drug side-effects
KCNH2 (ENSG00000055118)	KCNH2 (Q12809)	Protein Family	Amiodarone Hydrochloride	Blocker	Arrhythmias
				Blocker	hyperthyroidism
					hypotension
					respiratory disorders
					oedema
					anaemia
					hypertension
					haemorrhage
					cardiac conduction disorder
					myopathy
			Sotalol Hydrochloride	Blocker	arrhythmia
					bradycardia
					chest pain
					dyspnoea
					oedema
					palpitations
					syncope
			Amifampridine	Blocker	arrhythmia
					asthma
					palpitations
KCNK3 (ENSG00000171303)	KCNK3 (O14649)	Single Protein	Desflurane	Opener	arrhythmia
					hypertension
					hypotension
					respiratory disorders
					QT interval prolongation
					cardiac arrest
					haemorrhage
					hypokalaemia
					ventricular dysfunction
					myocardial infarction
					ischaemia
			Doxapram Hydrochloride	Blocker	Arrhythmias
					chest discomfort
					dyspnoea
					respiratory disorders
			Isoflurane	Opener	arrhythmia
					hypertension
					hypotension
					respiratory disorders
					QT interval prolongation
					cardiac arrest
					chest discomfort
					dyspnoea
					haemorrhage
			Sevoflurane	Opener	arrhythmia
					hypertension
					hypotension
					respiratory disorders
					QT interval prolongation
					cardiac arrest
					haemorrhage
					oedema
					Asthma
					atrioventricular block
PDE5A (ENSG00000138735)	PDE5A (O76074)	Single Protein	Avanafil	Inhibitor	angina pectoris
					chest pain
					hypertension
					oedema
					tachycardia
					dyspnoea

Table S8: Cardiometabolic drug side-effects of compounds targeting proteins encoded by CMR genes *(continued)*

Putative causal gene	Protein	Target type	Compound	Drug mechanism	Drug side-effects
					palpitations
					respiratory disorders
			Tadalafil	Inhibitor	Acute coronary syndrome
					oedema
					cerebrovascular insufficiency
					syncope
					Arrhythmias
					chest pain
					dyspnoea
					haemorrhage
					hypertension
					hypotension
					palpitations
			Dipyridamole	Inhibitor	Angina pectoris
					oedema
					haemorrhage
					hypotension
					tachycardia
			Pentoxifylline	Inhibitor	angina pectoris
					oedema
					arrhythmia
					haemorrhage
					hypotension
SCN10A (ENSG00000185313)	SCNAA (Q9Y5Y9)	Protein Family	Articaine Hydrochloride	Blocker	oedema
			Carbamazepine	Blocker	hyponatraemia
					oedema
					anaemia
					arrhythmia
					cardiac conduction disorder
					circulatory collapse
					congestive heart failure
					dyspnoea
					embolism and thrombosis
					coma
					hypertension
					hypotension
					syncope
			Chloroprocaine Hydrochloride	Blocker	hypotension
					Cardiac arrest
					dyspnoea
					respiratory disorders
					Arrhythmias
					hypertension
			Eslicarbazepine Acetate	Blocker	oedema
					anaemia
					bradycardia
					chest pain
					haemorrhage
					hypertension
					hypotension
					palpitations
			Fosphenytoin Sodium	Blocker	hypotension
					atrioventricular block
					bradycardia
					cerebrovascular insufficiency
					circulatory collapse
					hyperglycaemia
					respiratory disorders
					Cardiac arrest
			Lamotrigine	Blocker	oedema
					multi organ failure
			Lidocaine Hydrochloride	Blocker	arrhythmia

Table S8: Cardiometabolic drug side-effects of compounds targeting proteins encoded by CMR genes *(continued)*

Putative causal gene	Protein	Target type	Compound	Drug mechanism	Drug side-effects
					atrioventricular block
					cardiac arrest
					circulatory collapse
					hypotension
					respiratory disorders
			Mepivacaine Hydrochloride	Blocker	Arrhythmias
					hypertension
					hypotension
					cardiac arrest
			Orphenadrine Hydrochloride	Blocker	tachycardia
			Oxcarbazepine	Blocker	hyponatraemia
					hypertension
					oedema
					arrhythmia
					atrioventricular block
			Phenytoin	Blocker	arrhythmia
					cardiac arrest
					cerebrovascular insufficiency
					hypotension
					anaemia
					respiratory disorders
			Prilocaine Hydrochloride	Blocker	Arrhythmias
					hypertension
					hypotension
					Cardiac arrest
			Prilocaine	Blocker	cardiac arrest
					hypotension
			Primidone	Blocker	anaemia
			Propafenone Hydrochloride	Blocker	arrhythmia
					cardiac conduction disorder
					chest pain
					dyspnoea
					palpitations
					hypotension
					syncope
			Riluzole	Blocker	tachycardia
					Anaemia
					oedema
					interstitial lung disease
			Ropivacaine Hydrochloride	Blocker	Arrhythmias
					hypertension
					hypotension
					Cardiac arrest
					dyspnoea
					syncope
			Tetracaine	Blocker	oedema
			Topiramate	Blocker	anaemia
					dyspnoea
					haemorrhage
					oedema
					coma
					arrhythmia
					hypokalaemia
					hypotension
					palpitations
					syncope
			Lidocaine	Blocker	hypertension
			Zonisamide	Blocker	oedema
					coma
					dyspnoea
					stroke
					hypokalaemia

Table S8: Cardiometabolic drug side-effects of compounds targeting proteins encoded by CMR genes *(continued)*

Putative causal gene	Protein	Target type	Compound	Drug mechanism	Drug side-effects
					respiratory disorders

General:
Data were extracted from British National Formulary, and ChEMBL. Genes are provided with ensemlb id, and proteins with uniprot id.

Table S9: Drugged CMR proteins: BNF, ChEMBL and HPA annotations.

Protein (uniprot)	Compound	Drug type	Target type	Drug action	Curated indications	Curated side effects	mRNA Tissue specificity	Above average mRNA expression
AT1B2 (P14415)	Deslanoside, Digitoxin, Digoxin, Acetyldigitoxin	Small_Mol	Protein Complex Group	Inhibitor	Heart failure (for patients in sinus rhythm); Maintenance, for atrial fibrillation or flutter; Angioedemas, Hereditary; Atrial Fibrillation; Cardiovascular Diseases; Diabetes Mellitus; Diabetes Mellitus, Type 2; Essential Hypertension; Heart Failure; Tachycardia, Supraventricular	Arrhythmias; cardiac conduction disorder	0.914	Cerebral cortex, Pons and medulla
CAH6 (P23280)	Ethoxzolamide	Bio_Mol, Small_Mol	Protein Family	Inhibitor			0.999	Salivary gland
CD33 (P20138)	Gemtuzumab Ozogamicin, Lintuzumab, M195, Oncolysin M, Gemtuzumab, Ave-9633	Bio_Mol	Single Protein	Binding Agent, Other		Anaemia; ascites; dyspnoea; haemorrhage; hyperglycaemia; hypertension; hypotension; multi organ failure; oedema; tachycardia	0.880	Dendritic cells, Lymph node, Monocytes, Spleen
CHLE (P06276)	Propanidid	Bio_Mol, Small_Mol	Single Protein	Inhibitor			0.965	Liver
CHLE (P06276)	Rivastigmine Tartrate, Rivastigmine, Tacrine Hydrochloride	Bio_Mol, Small_Mol	Selectivity Group	Inhibitor	Hypotension, Orthostatic	angina pectoris; arrhythmias; atrioventricular block; hypertension; hypotension; syncope	0.965	Liver
CO6A1 (P12109)	Collagenase Clostridium Histolyticum, Ocriplasmin	Bio_Mol	Protein Complex Group	Hydrolytic Enzyme	Stroke; Venous Thrombosis	haemorrhage	0.801	Cervix, uterine, Colon, Endometrium, Smooth muscle
COFA1 (P39059)	Collagenase Clostridium Histolyticum, Ocriplasmin	Bio_Mol	Protein Complex Group	Hydrolytic Enzyme	Stroke; Venous Thrombosis	haemorrhage	0.842	Endometrium, Heart muscle, Placenta
EPHA1 (P21709)	Vandetanib	Small_Mol	Protein Family	Inhibitor			0.967	Parathyroid gland
EPHB6 (O15197)	Vandetanib	Bio_Mol, Small_Mol	Protein Family	Inhibitor			0.852	Basal ganglia, Cerebral cortex, Skin, Thymus
FGFR3 (P22607)	Pazopanib Hydrochloride, Enmd-2076, Masitinib, Dovitinib, Rg-7444	Bio_Mol, Small_Mol	Single Protein	Inhibitor	Asthma		0.946	Skin
FGFR3 (P22607)	Nintedanib Esylate, Enmd-981693, Arq-087, Bay-1163877, Cp-459632, Ly-2874455, Azd-4547, Xl-999, Bgj-398, Brivanib, Brivanib Alaninate, Orantinib	Bio_Mol, Small_Mol	Protein Family	Inhibitor			0.946	Skin
I17RA (Q96F46)	Brodalumab	Bio_Mol	Single Protein	Antagonist	Asthma		0.816	Bone marrow, Lymph node, Thymus
IL12B (P29460)	Ustekinumab, Briakinumab	Bio_Mol	Protein Complex	Inhibitor	Diabetes Mellitus, Type 1; Sarcoidosis	Respiratory disorders	0.952	Thymus
IL12B (P29460)	Briakinumab	Bio_Mol	Single Protein	Inhibitor			0.952	Thymus
IL6RA (P08887)	Satralizumab	Bio_Mol	Protein-Protein Interaction	Antagonist			0.873	Granulocytes, Liver, Skeletal muscle
IL6RA (P08887)	Tocilizumab, Sarilumab, Vobarilizumab	Bio_Mol	Single Protein	Inhibitor, Antagonist	Diabetes Mellitus, Type 1; Hypertension, Pulmonary; Non-ST Elevated Myocardial Infarction; Pneumonia; Pneumonia, Viral	Dyslipidaemia; dyslipidaemia; dyspnoea; hypertension; interstitial lung disease	0.873	Granulocytes, Liver, Skeletal muscle
LAMC2 (Q13753)	Ocriplasmin	Bio_Mol	Protein Complex Group	Hydrolytic Enzyme	Stroke; Venous Thrombosis	haemorrhage	0.776	Appendix, Fallopian tube, Lung, Placenta
PTGDS (P41222)	Antrafenine	Bio_Mol	Single Protein	Inhibitor			0.806	Cervix, uterine, Heart muscle, Retina

Table S9: Drugged CMR proteins: BNF, ChEMBL and HPA annotations. *(continued)*

Protein (uniprot)	Compound	Drug type	Target type	Drug action	Curated indications	Curated side effects	mRNA Tissue specificity	Above average mRNA expression
RENI (P00797)	Aliskiren Fumarate	Bio_Mol, Small_Mol	Single Protein	Inhibitor	Hypertension		0.983	Kidney
SLAF7 (Q9NQ25)	Elotuzumab	Bio_Mol	Single Protein	Inhibitor	Amyloidosis, Familial	Chest pain	0.859	Appendix, Dendritic cells, Spleen, T-cells, Tonsil
TIE2 (Q02763)	Regorafenib, Vandetanib, Pexmetinib, Ce-245677, Cep-11981, Altiratinib, Foretinib, Mgcd-265	Bio_Mol, Small_Mol	Single Protein	Inhibitor	Pulmonary Disease, Chronic Obstructive	,; Aneurysm; anaemia; artery dissection; hypertension; myocardial infarction; myocardial ischaemia; pain	0.808	Adipose tissue, Kidney, Lung, Placenta, Spleen
VGFR3 (P35916)	Jnj-26483327, Anlotinib, Telatinib, Famitinib, Imc-3C5	Bio_Mol, Small_Mol	Single Protein	Inhibitor			0.903	Breast, Lymph node, Placenta
VGFR3 (P35916)	Sunitinib Malate, Axitinib, Vandetanib, Sorafenib Tosylate, Pazopanib Hydrochloride, Regorafenib, Nintedanib Esylate, Lenvatinib Mesylate, Su-14813, Su-014813, 4Sc-203, Ilorasertib, Cep-11981, Cep-5214, Cep-7055, Cp-459632, Chiauranib, Sulfatinib, Krn-633, L-21649, Osi-930, Tak-593, Rg-1530, Ag-13958, Bms-690514, Lucitanib, Foretinib, Fruquintinib, Mgcd-265, X-82, Xl-820, Xl-999, Linifanib, Cediranib, Brivanib, Brivanib Alaninate, Tivozanib, Vatalanib, Semaxanib, Dovitinib, Motesanib	Bio_Mol, Small_Mol	Protein Family	Inhibitor	Pulmonary Disease, Chronic Obstructive	,; Aneurysm; Coronary artery insufficiency; QT interval prolongation; anaemia; angina pectoris; arrhythmias; artery dissection; cerebrovascular insufficiency; chest pain; dyspnoea; embolism and thrombosis; haemorrhage; heart failure; hypertension; hyperthyroidism; ischaemia; myocardial infarction; myocardial ischaemia; pain; pulmonary oedema	0.903	Breast, Lymph node, Placenta

General:
Data were extracted from British National Formulary, ChEMBL, Human Protein Atlas. Indicates and side effects were curated for cardiovascular relevant traits. Tissues with above average mRNA expression were selected based on a z-statistic of at least 1.96. Tissue specificity ranges between 0 (ubiquitous expressed) and 1 (specifically expressed).

Table S10: CMR drugged proteins: MR effect estimates of plasma protein effects on sixteen CMR traits.

Protein (uniprot)	Gene (ensembl)	CMR trait	MD (95%CI)	P-value	Multiple testing threshold	Q p-value	No. variants	MR model	pQTL source
IL6RA (P08887)	IL6R (ENSG00000160712)	RV – SV (ml)	-0.68 (-0.84; -0.51)	4.4×10^{-16}	7.81×10^{-6}	0.091	72	MR Egger	Scallop
		RV – PFR (ml/s)	-2.79 (-3.55; -2.04)	3.8×10^{-13}		0.017	74	MR Egger	
		RV – PER (ml/s)	-2.26 (-3.12; -1.40)	2.8×10^{-7}		0.029	70	MR Egger	
		RV – PAFR (ml/s)	1.18 (0.82; 1.53)	1.1×10^{-10}		0.043	77	IVW	
		RV – ESV (ml)	-0.47 (-0.62; -0.32)	6.7×10^{-10}		0.008	76	MR Egger	
		RV – EF (%)	0.01 (-0.04; 0.07)	6.1×10^{-1}		0.018	77	MR Egger	
		RV – EDV (ml)	-1.19 (-1.43; -0.94)	1.0×10^{-100}		0.039	73	MR Egger	
		LV – SV (ml)	0.05 (-0.02; 0.11)	1.4×10^{-1}		0.027	67	IVW	
		LV – PFR (ml/s)	-3.32 (-4.22; -2.42)	4.9×10^{-13}		0.024	73	MR Egger	
		LV – PER (ml/s)	-1.50 (-2.37; -0.62)	7.6×10^{-4}		0.045	74	MR Egger	
		LV – PAFR (ml/s)	0.80 (0.40; 1.20)	9.2×10^{-5}		0.407	73	IVW	
		LV – MVR (g/ml)	0.00 (0.00; 0.00)	9.5×10^{-10}		0.003	72	MR Egger	
		LV – ESV (ml)	-0.71 (-0.85; -0.57)	1.0×10^{-100}		0.009	72	MR Egger	
		LV – EF (%)	0.01 (-0.01; 0.04)	3.5×10^{-1}		0.264	75	IVW	
		LV – EDV (ml)	0.03 (-0.06; 0.13)	5.1×10^{-1}		0.002	68	IVW	
		LV – EDM (g)	-0.31 (-0.49; -0.14)	4.6×10^{-4}		<0.001	72	MR Egger	
PTGDS (P41222)	PTGDS (ENSG00000107317)	RV – SV (ml)	1.18 (0.60; 1.76)	6.8×10^{-5}	7.81×10^{-6}	0.424	6	IVW	Interval
		RV – PFR (ml/s)	-0.41 (-3.52; 2.71)	8.0×10^{-1}		0.744	6	IVW	
		RV – PER (ml/s)	1.92 (-2.34; 6.17)	3.8×10^{-1}		0.125	6	IVW	
		RV – PAFR (ml/s)	9.24 (5.56; 12.92)	8.5×10^{-7}		0.988	6	IVW	
		RV – ESV (ml)	-2.00 (-5.39; 1.39)	2.5×10^{-1}		0.943	6	MR Egger	
		RV – EF (%)	0.10 (-0.12; 0.33)	3.7×10^{-1}		0.550	6	IVW	
		RV – EDV (ml)	1.93 (0.92; 2.94)	1.9×10^{-4}		0.358	6	IVW	
		LV – SV (ml)	2.29 (1.70; 2.89)	4.0×10^{-14}		0.792	6	IVW	
		LV – PFR (ml/s)	-5.25 (-25.53; 15.02)	6.1×10^{-1}		0.743	6	MR Egger	
		LV – PER (ml/s)	-12.44 (-32.84; 7.96)	2.3×10^{-1}		0.898	5	MR Egger	
		LV – PAFR (ml/s)	7.22 (2.56; 11.88)	2.4×10^{-3}		0.751	5	IVW	
		LV – MVR (g/ml)	0.00 (-0.00; 0.00)	5.3×10^{-1}		0.858	6	IVW	
		LV – ESV (ml)	0.10 (-0.45; 0.66)	7.2×10^{-1}		0.490	6	IVW	
		LV – EF (%)	0.71 (0.42; 0.99)	9.0×10^{-7}		0.177	6	IVW	
		LV – EDV (ml)	2.29 (1.34; 3.23)	2.1×10^{-6}		0.674	6	IVW	
		LV – EDM (g)	1.36 (0.85; 1.88)	2.3×10^{-7}		0.511	6	IVW	
I17RA (Q96F46)	IL17RA (ENSG00000177663)	RV – SV (ml)	0.17 (0.07; 0.26)	8.2×10^{-4}	7.81×10^{-6}	0.007	45	IVW	Interval
		RV – PFR (ml/s)	0.53 (0.04; 1.02)	3.4×10^{-2}		<0.001	47	IVW	
		RV – PER (ml/s)	0.21 (-0.25; 0.68)	3.6×10^{-1}		<0.001	43	IVW	
		RV – PAFR (ml/s)	1.32 (0.81; 1.84)	4.4×10^{-7}		0.014	45	IVW	
		RV – ESV (ml)	0.11 (0.02; 0.20)	1.7×10^{-2}		0.135	47	IVW	
		RV – EF (%)	0.05 (0.01; 0.08)	1.2×10^{-2}		0.081	48	IVW	
		RV – EDV (ml)	0.40 (0.26; 0.53)	5.8×10^{-9}		<0.001	47	IVW	
		LV – SV (ml)	0.02 (-0.06; 0.11)	5.6×10^{-1}		<0.001	46	IVW	
		LV – PFR (ml/s)	0.20 (-0.73; 1.13)	6.8×10^{-1}		0.035	48	MR Egger	
		LV – PER (ml/s)	-0.49 (-0.97; -0.01)	4.8×10^{-2}		<0.001	47	IVW	
		LV – PAFR (ml/s)	0.38 (-0.27; 1.03)	2.5×10^{-1}		<0.001	44	IVW	
		LV – MVR (g/ml)	-0.00 (-0.00; -0.00)	2.1×10^{-4}		0.033	47	MR Egger	
		LV – ESV (ml)	-0.21 (-0.28; -0.13)	1.8×10^{-7}		0.610	46	IVW	
		LV – EF (%)	0.09 (0.06; 0.13)	1.3×10^{-7}		0.203	47	IVW	
		LV – EDV (ml)	-0.17 (-0.30; -0.03)	1.4×10^{-2}		0.011	47	IVW	
		LV – EDM (g)	-0.34 (-0.41; -0.27)	1.0×10^{-100}		0.032	45	IVW	
TIE2 (Q02763)	TEK (ENSG00000120156)	RV – SV (ml)	0.02 (-0.35; 0.39)	9.1×10^{-1}	7.81×10^{-6}	<0.001	52	MR Egger	Scallop
		RV – PFR (ml/s)	-2.80 (-5.15; -0.46)	1.9×10^{-2}		0.018	49	MR Egger	
		RV – PER (ml/s)	0.22 (-2.02; 2.46)	8.5×10^{-1}		<0.001	47	MR Egger	
		RV – PAFR (ml/s)	5.47 (4.15; 6.79)	4.4×10^{-16}		<0.001	54	IVW	
		RV – ESV (ml)	-0.68 (-0.89; -0.48)	4.1×10^{-11}		<0.001	48	IVW	
		RV – EF (%)	-0.03 (-0.15; 0.10)	6.8×10^{-1}		0.015	53	MR Egger	
		RV – EDV (ml)	-0.02 (-0.56; 0.52)	9.4×10^{-1}		<0.001	55	MR Egger	
		LV – SV (ml)	0.23 (-0.16; 0.62)	2.4×10^{-1}		<0.001	48	MR Egger	
		LV – PFR (ml/s)	-0.48 (-2.45; 1.49)	6.3×10^{-1}		<0.001	52	MR Egger	
		LV – PER (ml/s)	-0.12 (-2.54; 2.30)	9.2×10^{-1}		<0.001	48	MR Egger	
		LV – PAFR (ml/s)	1.81 (0.57; 3.05)	4.2×10^{-3}		<0.001	55	IVW	
		LV – MVR (g/ml)	-0.00 (-0.00; -0.00)	4.6×10^{-2}		0.011	52	MR Egger	
		LV – ESV (ml)	-0.34 (-0.74; 0.06)	9.4×10^{-2}		<0.001	44	MR Egger	
		LV – EF (%)	0.43 (0.32; 0.55)	8.9×10^{-13}		<0.001	49	MR Egger	
		LV – EDV (ml)	0.20 (-0.50; 0.90)	5.7×10^{-1}		0.013	50	MR Egger	

Table S10: CMR drugged proteins: MR effect estimates of plasma protein effects on sixteen CMR traits. *(continued)*

Protein (uniprot)	Gene (ensembl)	CMR trait	MD (95%CI)	P-value	Multiple testing threshold	Q p-value	No. variants	MR model	pQTL source
		LV – EDM (g)	-0.14 (-0.53; 0.25)	4.7×10^{-1}		<0.001	46	MR Egger	
EPHA1 (P21709)	EPHA1 (ENSG00000146904)	RV – SV (ml)	-0.04 (-0.66; 0.57)	8.9×10^{-1}	7.81×10^{-6}	0.616	16	MR Egger	Interval
		RV – PFR (ml/s)	0.58 (-2.72; 3.89)	7.3×10^{-1}		0.591	16	MR Egger	
		RV – PER (ml/s)	-1.26 (-4.70; 2.18)	4.7×10^{-1}		0.553	16	MR Egger	
		RV – PAFR (ml/s)	3.38 (1.59; 5.18)	2.3×10^{-4}		0.099	17	IVW	
		RV – ESV (ml)	-0.71 (-1.42; 0.00)	5.1×10^{-2}		0.239	14	MR Egger	
		RV – EF (%)	0.42 (0.30; 0.53)	1.3×10^{-12}		0.167	16	IVW	
		RV – EDV (ml)	-0.82 (-1.20; -0.43)	3.7×10^{-5}		0.543	17	IVW	
		LV – SV (ml)	0.23 (-0.01; 0.47)	5.8×10^{-2}		0.039	17	IVW	
		LV – PFR (ml/s)	-1.46 (-6.24; 3.33)	5.5×10^{-1}		0.069	16	MR Egger	
		LV – PER (ml/s)	-2.31 (-6.02; 1.40)	2.2×10^{-1}		0.027	16	MR Egger	
		LV – PAFR (ml/s)	1.00 (-0.56; 2.56)	2.1×10^{-1}		0.451	17	IVW	
		LV – MVR (g/ml)	0.00 (-0.00; 0.00)	5.1×10^{-1}		0.005	17	IVW	
		LV – ESV (ml)	-0.71 (-0.96; -0.45)	3.3×10^{-8}		0.217	17	IVW	
		LV – EF (%)	0.34 (0.24; 0.43)	6.0×10^{-13}		0.042	17	IVW	
		LV – EDV (ml)	-0.49 (-0.89; -0.09)	1.7×10^{-2}		0.335	17	IVW	
		LV – EDM (g)	-0.16 (-0.41; 0.08)	1.9×10^{-1}		0.148	17	IVW	
CO6A1 (P12109)	COL6A1 (ENSG00000142156)	RV – SV (ml)	1.29 (0.06; 2.53)	4.0×10^{-2}	7.81×10^{-6}	0.344	20	MR Egger	Interval
		RV – PFR (ml/s)	6.19 (-0.35; 12.73)	6.4×10^{-2}		0.364	21	MR Egger	
		RV – PER (ml/s)	6.10 (4.51; 7.68)	4.2×10^{-14}		0.003	19	IVW	
		RV – PAFR (ml/s)	-6.11 (-13.82; 1.60)	1.2×10^{-1}		0.379	20	MR Egger	
		RV – ESV (ml)	1.30 (-0.14; 2.74)	7.6×10^{-2}		0.104	21	MR Egger	
		RV – EF (%)	-0.07 (-0.56; 0.41)	7.7×10^{-1}		0.329	20	MR Egger	
		RV – EDV (ml)	2.95 (1.00; 4.90)	3.1×10^{-3}		0.759	21	MR Egger	
		LV – SV (ml)	-0.08 (-1.28; 1.12)	9.0×10^{-1}		0.019	21	MR Egger	
		LV – PFR (ml/s)	-1.20 (-10.61; 8.21)	8.0×10^{-1}		0.097	19	MR Egger	
		LV – PER (ml/s)	-0.45 (-7.84; 6.93)	9.0×10^{-1}		0.641	19	MR Egger	
		LV – PAFR (ml/s)	-8.60 (-16.66; -0.54)	3.7×10^{-2}		0.012	19	MR Egger	
		LV – MVR (g/ml)	0.01 (0.01; 0.01)	7.9×10^{-12}		0.056	19	IVW	
		LV – ESV (ml)	1.13 (-0.02; 2.29)	5.5×10^{-2}		0.390	21	MR Egger	
		LV – EF (%)	-0.30 (-0.42; -0.18)	1.6×10^{-6}		0.002	18	IVW	
		LV – EDV (ml)	0.95 (-0.97; 2.86)	3.3×10^{-1}		0.872	21	MR Egger	
		LV – EDM (g)	1.10 (-0.07; 2.27)	6.6×10^{-2}		0.211	20	MR Egger	
CHLE (P06276)	BCHE (ENSG00000114200)	RV – SV (ml)	-0.16 (-0.69; 0.36)	5.4×10^{-1}	7.81×10^{-6}	0.883	17	MR Egger	Framingham
		RV – PFR (ml/s)	-0.25 (-0.57; 0.06)	1.1×10^{-1}		0.038	24	IVW	
		RV – PER (ml/s)	1.67 (-2.65; 5.98)	4.5×10^{-1}		0.218	12	MR Egger	
		RV – PAFR (ml/s)	-1.33 (-1.78; -0.88)	6.3×10^{-9}		0.519	24	IVW	
		RV – ESV (ml)	-0.80 (-1.18; -0.42)	4.1×10^{-5}		0.002	20	MR Egger	
		RV – EF (%)	0.04 (-0.03; 0.11)	3.0×10^{-1}		0.024	20	IVW	
		RV – EDV (ml)	0.42 (0.33; 0.52)	1.0×10^{-100}		<0.001	23	IVW	
		LV – SV (ml)	-0.72 (-1.53; 0.10)	8.6×10^{-2}		0.150	15	MR Egger	
		LV – PFR (ml/s)	-0.37 (-0.72; -0.03)	3.6×10^{-2}		0.677	26	IVW	
		LV – PER (ml/s)	-2.42 (-8.59; 3.74)	4.4×10^{-1}		0.990	8	MR Egger	
		LV – PAFR (ml/s)	-4.03 (-5.67; -2.38)	1.6×10^{-6}		0.347	17	IVW	
		LV – MVR (g/ml)	0.00 (-0.00; 0.00)	5.3×10^{-1}		0.278	26	IVW	
		LV – ESV (ml)	-0.47 (-1.28; 0.33)	2.5×10^{-1}		0.495	11	MR Egger	
		LV – EF (%)	-0.20 (-0.37; -0.03)	2.2×10^{-2}		0.025	20	MR Egger	
		LV – EDV (ml)	-1.19 (-2.78; 0.40)	1.4×10^{-1}		0.853	10	MR Egger	
		LV – EDM (g)	-0.58 (-1.63; 0.47)	2.8×10^{-1}		0.907	7	MR Egger	
SLAF7 (Q9NQ25)	SLAMF7 (ENSG00000026751)	RV – SV (ml)	0.29 (0.11; 0.47)	1.9×10^{-3}	7.81×10^{-6}	0.888	30	MR Egger	Interval
		RV – PFR (ml/s)	1.87 (0.71; 3.04)	1.6×10^{-3}		0.084	30	MR Egger	
		RV – PER (ml/s)	0.61 (-0.53; 1.75)	2.9×10^{-1}		0.225	29	MR Egger	
		RV – PAFR (ml/s)	2.26 (1.60; 2.91)	1.5×10^{-11}		0.858	29	IVW	
		RV – ESV (ml)	-0.22 (-0.32; -0.12)	1.6×10^{-5}		0.035	29	IVW	
		RV – EF (%)	0.12 (0.08; 0.17)	4.3×10^{-8}		0.089	30	IVW	
		RV – EDV (ml)	-0.19 (-0.37; 0.00)	5.5×10^{-2}		0.124	28	IVW	
		LV – SV (ml)	0.07 (-0.13; 0.26)	4.8×10^{-1}		0.368	30	MR Egger	
		LV – PFR (ml/s)	1.59 (0.45; 2.74)	6.5×10^{-3}		0.833	30	MR Egger	
		LV – PER (ml/s)	1.23 (0.41; 2.05)	3.2×10^{-3}		0.128	27	IVW	
		LV – PAFR (ml/s)	0.34 (-0.91; 1.60)	5.9×10^{-1}		0.785	29	MR Egger	
		LV – MVR (g/ml)	0.00 (-0.00; 0.00)	4.0×10^{-1}		0.244	28	IVW	
		LV – ESV (ml)	-0.00 (-0.19; 0.19)	1.0×10^{0}		0.309	30	MR Egger	

Table S10: CMR drugged proteins: MR effect estimates of plasma protein effects on sixteen CMR traits. *(continued)*

Protein (uniprot)	Gene (ensembl)	CMR trait	MD (95%CI)	P-value	Multiple testing threshold	Q p-value	No. variants	MR model	pQTL source
		LV – EF (%)	0.01 (-0.03; 0.05)	5.8×10^{-1}		0.809	30	IVW	
		LV – EDV (ml)	0.06 (-0.27; 0.39)	7.4×10^{-1}		0.248	29	MR Egger	
		LV – EDM (g)	0.03 (-0.14; 0.19)	7.7×10^{-1}		0.039	26	MR Egger	
COFA1 (P39059)	COL15A1 (ENSG00000204291)	RV – SV (ml)	0.68 (-0.76; 2.13)	3.5×10^{-1}	7.81×10^{-6}	0.350	18	MR Egger	Interval
		RV – PFR (ml/s)	-0.06 (-2.05; 1.93)	9.5×10^{-1}		0.645	18	IVW	
		RV – PER (ml/s)	5.06 (-2.63; 12.75)	2.0×10^{-1}		0.535	18	MR Egger	
		RV – PAFR (ml/s)	-2.24 (-10.98; 6.50)	6.2×10^{-1}		0.877	18	MR Egger	
		RV – ESV (ml)	0.65 (-0.77; 2.06)	3.7×10^{-1}		0.619	18	MR Egger	
		RV – EF (%)	0.01 (-0.13; 0.16)	8.8×10^{-1}		0.872	18	IVW	
		RV – EDV (ml)	-1.82 (-2.47; -1.17)	3.7×10^{-8}		0.595	17	IVW	
		LV – SV (ml)	0.26 (-0.14; 0.67)	2.0×10^{-1}		0.439	17	IVW	
		LV – PFR (ml/s)	-5.55 (-7.88; -3.22)	3.0×10^{-6}		0.405	18	IVW	
		LV – PER (ml/s)	4.61 (-3.68; 12.91)	2.8×10^{-1}		0.516	18	MR Egger	
		LV – PAFR (ml/s)	-4.25 (-13.47; 4.97)	3.7×10^{-1}		0.545	18	MR Egger	
		LV – MVR (g/ml)	0.00 (-0.00; 0.01)	4.5×10^{-1}		0.628	18	MR Egger	
		LV – ESV (ml)	-0.44 (-0.86; -0.02)	4.0×10^{-2}		0.124	18	IVW	
		LV – EF (%)	-0.01 (-0.55; 0.53)	9.7×10^{-1}		0.617	18	MR Egger	
		LV – EDV (ml)	0.17 (-0.56; 0.89)	6.5×10^{-1}		0.111	18	IVW	
		LV – EDM (g)	0.17 (-0.16; 0.50)	3.2×10^{-1}		0.801	18	IVW	
AT1B2 (P14415)	ATP1B2 (ENSG00000129244)	RV – SV (ml)	-0.50 (-0.79; -0.21)	8.1×10^{-4}	7.81×10^{-6}	<0.001	16	IVW	Interval
		RV – PFR (ml/s)	-3.53 (-4.99; -2.08)	1.9×10^{-6}		0.012	17	IVW	
		RV – PER (ml/s)	-0.35 (-1.95; 1.26)	6.7×10^{-1}		<0.001	16	IVW	
		RV – PAFR (ml/s)	0.89 (-3.84; 5.61)	7.1×10^{-1}		0.331	21	MR Egger	
		RV – ESV (ml)	-0.92 (-1.18; -0.66)	6.4×10^{-12}		0.003	19	IVW	
		RV – EF (%)	0.28 (0.00; 0.56)	4.8×10^{-2}		0.046	20	MR Egger	
		RV – EDV (ml)	-0.68 (-2.00; 0.65)	3.2×10^{-1}		<0.001	18	MR Egger	
		LV – SV (ml)	0.12 (-0.20; 0.44)	4.6×10^{-1}		<0.001	15	IVW	
		LV – PFR (ml/s)	3.83 (1.65; 6.02)	5.7×10^{-4}		0.091	16	IVW	
		LV – PER (ml/s)	0.70 (-1.00; 2.40)	4.2×10^{-1}		0.028	18	IVW	
		LV – PAFR (ml/s)	-6.29 (-11.51; -1.08)	1.8×10^{-2}		0.141	21	MR Egger	
		LV – MVR (g/ml)	-0.00 (-0.00; -0.00)	4.5×10^{-2}		0.267	16	IVW	
		LV – ESV (ml)	-0.32 (-1.06; 0.43)	4.0×10^{-1}		0.023	19	MR Egger	
		LV – EF (%)	0.05 (-0.06; 0.16)	3.4×10^{-1}		0.433	18	IVW	
		LV – EDV (ml)	0.51 (-0.06; 1.09)	8.2×10^{-2}		0.002	14	IVW	
		LV – EDM (g)	0.03 (-0.78; 0.83)	9.5×10^{-1}		0.102	21	MR Egger	
VGFR3 (P35916)	FLT4 (ENSG00000037280)	RV – SV (ml)	-1.47 (-2.96; 0.02)	5.3×10^{-2}	7.81×10^{-6}	0.552	22	MR Egger	Interval
		RV – PFR (ml/s)	-1.43 (-3.01; 0.15)	7.5×10^{-2}		0.901	23	IVW	
		RV – PER (ml/s)	-11.76 (-20.13; -3.39)	5.9×10^{-3}		0.006	20	MR Egger	
		RV – PAFR (ml/s)	0.45 (-1.43; 2.32)	6.4×10^{-1}		0.006	22	IVW	
		RV – ESV (ml)	-0.14 (-0.45; 0.17)	3.8×10^{-1}		0.373	23	IVW	
		RV – EF (%)	-0.51 (-1.09; 0.07)	8.5×10^{-2}		0.009	22	MR Egger	
		RV – EDV (ml)	-0.10 (-0.59; 0.39)	6.8×10^{-1}		0.677	23	IVW	
		LV – SV (ml)	0.27 (-1.29; 1.83)	7.4×10^{-1}		0.417	22	MR Egger	
		LV – PFR (ml/s)	4.44 (2.57; 6.30)	3.0×10^{-6}		0.602	21	IVW	
		LV – PER (ml/s)	2.04 (-0.12; 4.20)	6.4×10^{-2}		0.006	21	IVW	
		LV – PAFR (ml/s)	-10.41 (-22.03; 1.21)	7.9×10^{-2}		0.137	22	MR Egger	
		LV – MVR (g/ml)	-0.00 (-0.00; -0.00)	1.5×10^{-2}		0.035	23	IVW	
		LV – ESV (ml)	0.24 (-0.04; 0.53)	9.0×10^{-2}		0.889	23	IVW	
		LV – EF (%)	-0.65 (-1.23; -0.06)	3.1×10^{-2}		0.464	22	MR Egger	
		LV – EDV (ml)	1.57 (-0.87; 4.01)	2.1×10^{-1}		0.871	22	MR Egger	
		LV – EDM (g)	-0.36 (-0.63; -0.10)	6.5×10^{-3}		0.006	23	IVW	
RENI (P00797)	REN (ENSG00000143839)	RV – SV (ml)	-0.01 (-1.12; 1.10)	9.9×10^{-1}	7.81×10^{-6}	0.666	11	IVW	Scallop
		RV – PFR (ml/s)	7.60 (3.50; 11.70)	2.8×10^{-4}		0.816	12	IVW	
		RV – PER (ml/s)	-14.59 (-27.63; -1.54)	2.8×10^{-2}		0.729	11	MR Egger	
		RV – PAFR (ml/s)	-2.12 (-6.97; 2.73)	3.9×10^{-1}		0.043	12	IVW	
		RV – ESV (ml)	-1.08 (-1.98; -0.18)	1.8×10^{-2}		0.210	12	IVW	
		RV – EF (%)	-0.07 (-0.64; 0.51)	8.2×10^{-1}		0.059	11	IVW	
		RV – EDV (ml)	0.80 (-0.47; 2.07)	2.2×10^{-1}		0.782	12	IVW	
		LV – SV (ml)	1.40 (0.62; 2.18)	4.5×10^{-4}		0.491	12	IVW	
		LV – PFR (ml/s)	9.64 (4.94; 14.34)	5.8×10^{-5}		0.436	12	IVW	
		LV – PER (ml/s)	-1.88 (-16.68; 12.92)	8.0×10^{-1}		0.351	11	MR Egger	
		LV – PAFR (ml/s)	0.64 (-4.75; 6.03)	8.2×10^{-1}		0.343	12	IVW	

Table S10: CMR drugged proteins: MR effect estimates of plasma protein effects on sixteen CMR traits. *(continued)*

Protein (uniprot)	Gene (ensembl)	CMR trait	MD (95%CI)	P-value	Multiple testing threshold	Q p-value	No. variants	MR model	pQTL source
		LV – MVR (g/ml)	-0.00 (-0.01; 0.00)	1.4×10^{-1}		0.354	12	IVW	
		LV – ESV (ml)	-0.82 (-1.55; -0.09)	2.7×10^{-2}		0.972	12	IVW	
		LV – EF (%)	0.97 (0.59; 1.35)	6.0×10^{-7}		0.086	12	IVW	
		LV – EDV (ml)	0.56 (-0.68; 1.80)	3.8×10^{-1}		0.984	12	IVW	
		LV – EDM (g)	0.04 (-1.86; 1.93)	9.7×10^{-1}		0.740	12	MR Egger	
LAMC2 (Q13753)	LAMC2 (ENSG00000058085)	RV – SV (ml)	-0.07 (-0.38; 0.24)	6.7×10^{-1}	7.81×10^{-6}	0.033	34	MR Egger	Interval
		RV – PFR (ml/s)	1.98 (0.30; 3.65)	2.1×10^{-2}		0.007	35	MR Egger	
		RV – PER (ml/s)	2.83 (0.91; 4.75)	3.9×10^{-3}		0.184	35	MR Egger	
		RV – PAFR (ml/s)	5.33 (3.18; 7.48)	1.2×10^{-6}		0.216	35	MR Egger	
		RV – ESV (ml)	-0.28 (-0.67; 0.11)	1.6×10^{-1}		0.057	34	MR Egger	
		RV – EF (%)	0.17 (0.05; 0.30)	7.2×10^{-3}		0.009	33	MR Egger	
		RV – EDV (ml)	-0.42 (-0.93; 0.10)	1.2×10^{-1}		0.004	34	MR Egger	
		LV – SV (ml)	-0.50 (-0.88; -0.12)	9.4×10^{-3}		0.068	35	MR Egger	
		LV – PFR (ml/s)	-0.23 (-2.15; 1.68)	8.1×10^{-1}		0.034	35	MR Egger	
		LV – PER (ml/s)	-0.32 (-1.41; 0.77)	5.7×10^{-1}		0.026	35	IVW	
		LV – PAFR (ml/s)	2.82 (1.52; 4.12)	2.1×10^{-5}		0.044	34	IVW	
		LV – MVR (g/ml)	0.00 (0.00; 0.00)	2.3×10^{-4}		0.007	34	MR Egger	
		LV – ESV (ml)	-0.17 (-0.35; -0.00)	5.0×10^{-2}		0.006	35	IVW	
		LV – EF (%)	0.08 (-0.06; 0.23)	2.4×10^{-1}		0.105	35	MR Egger	
		LV – EDV (ml)	-1.00 (-1.51; -0.49)	1.2×10^{-4}		0.003	34	MR Egger	
		LV – EDM (g)	-0.13 (-0.30; 0.05)	1.6×10^{-1}		0.196	35	IVW	
IL12B (P29460)	IL12B (ENSG00000113302)	RV – SV (ml)	-1.02 (-1.99; -0.06)	3.8×10^{-2}	7.81×10^{-6}	0.015	15	MR Egger	Interval
		RV – PFR (ml/s)	-13.36 (-18.91; -7.80)	2.4×10^{-6}		0.314	15	MR Egger	
		RV – PER (ml/s)	2.12 (0.38; 3.86)	1.7×10^{-2}		0.777	16	IVW	
		RV – PAFR (ml/s)	2.64 (0.46; 4.81)	1.8×10^{-2}		<0.001	14	IVW	
		RV – ESV (ml)	0.47 (0.12; 0.82)	9.0×10^{-3}		0.042	14	IVW	
		RV – EF (%)	0.13 (-0.26; 0.52)	5.2×10^{-1}		0.361	15	MR Egger	
		RV – EDV (ml)	1.45 (-0.75; 3.65)	2.0×10^{-1}		0.002	13	MR Egger	
		LV – SV (ml)	-0.66 (-1.65; 0.33)	1.9×10^{-1}		0.953	15	MR Egger	
		LV – PFR (ml/s)	-2.65 (-8.58; 3.27)	3.8×10^{-1}		0.571	15	MR Egger	
		LV – PER (ml/s)	-0.19 (-2.36; 1.99)	8.7×10^{-1}		0.168	16	IVW	
		LV – PAFR (ml/s)	-1.90 (-3.99; 0.19)	7.4×10^{-2}		0.495	16	IVW	
		LV – MVR (g/ml)	0.00 (-0.00; 0.01)	3.0×10^{-1}		0.994	15	MR Egger	
		LV – ESV (ml)	-0.43 (-0.77; -0.09)	1.2×10^{-2}		0.209	16	IVW	
		LV – EF (%)	0.18 (0.06; 0.30)	3.8×10^{-3}		0.726	16	IVW	
		LV – EDV (ml)	-1.64 (-3.36; 0.08)	6.2×10^{-2}		0.278	15	MR Egger	
		LV – EDM (g)	-0.69 (-1.69; 0.31)	1.7×10^{-1}		0.176	15	MR Egger	
FGFR3 (P22607)	FGFR3 (ENSG00000068078)	RV – SV (ml)	0.22 (-0.36; 0.80)	4.5×10^{-1}	7.81×10^{-6}	0.327	9	IVW	Interval
		RV – PFR (ml/s)	2.24 (-0.89; 5.37)	1.6×10^{-1}		0.888	8	IVW	
		RV – PER (ml/s)	-0.83 (-3.85; 2.18)	5.9×10^{-1}		0.505	9	IVW	
		RV – PAFR (ml/s)	-2.92 (-6.35; 0.51)	9.5×10^{-2}		0.843	9	IVW	
		RV – ESV (ml)	-1.31 (-2.00; -0.62)	1.9×10^{-4}		0.136	9	IVW	
		RV – EF (%)	-0.64 (-2.09; 0.82)	3.9×10^{-1}		0.324	9	MR Egger	
		RV – EDV (ml)	-1.02 (-1.91; -0.12)	2.7×10^{-2}		0.714	9	IVW	
		LV – SV (ml)	-0.09 (-0.64; 0.47)	7.6×10^{-1}		0.906	9	IVW	
		LV – PFR (ml/s)	4.15 (0.45; 7.85)	2.8×10^{-2}		0.380	8	IVW	
		LV – PER (ml/s)	-4.81 (-8.07; -1.56)	3.7×10^{-3}		0.498	9	IVW	
		LV – PAFR (ml/s)	-13.09 (-17.53; -8.65)	7.6×10^{-9}		0.148	9	IVW	
		LV – MVR (g/ml)	-0.00 (-0.00; 0.00)	5.0×10^{-1}		0.446	9	IVW	
		LV – ESV (ml)	-1.04 (-1.69; -0.38)	1.8×10^{-3}		0.215	8	IVW	
		LV – EF (%)	0.36 (0.08; 0.65)	1.2×10^{-2}		0.146	8	IVW	
		LV – EDV (ml)	-1.52 (-2.40; -0.64)	6.9×10^{-4}		0.486	9	IVW	
		LV – EDM (g)	-1.13 (-1.70; -0.57)	8.6×10^{-5}		0.199	9	IVW	
EPHB6 (O15197)	EPHB6 (ENSG00000106123)	RV – SV (ml)	-0.90 (-1.37; -0.43)	1.6×10^{-4}	7.81×10^{-6}	0.354	13	IVW	Interval
		RV – PFR (ml/s)	-6.88 (-9.27; -4.50)	1.6×10^{-8}		0.007	13	IVW	
		RV – PER (ml/s)	-9.20 (-15.76; -2.63)	6.0×10^{-3}		0.320	15	MR Egger	
		RV – PAFR (ml/s)	0.51 (-2.46; 3.49)	7.4×10^{-1}		0.848	12	IVW	
		RV – ESV (ml)	0.05 (-0.48; 0.57)	8.7×10^{-1}		0.196	13	IVW	
		RV – EF (%)	0.18 (-0.25; 0.61)	4.2×10^{-1}		0.870	15	MR Egger	
		RV – EDV (ml)	-0.96 (-1.85; -0.07)	3.4×10^{-2}		0.139	13	IVW	
		LV – SV (ml)	-0.95 (-1.41; -0.50)	4.1×10^{-5}		0.706	13	IVW	
		LV – PFR (ml/s)	-5.11 (-8.38; -1.83)	2.2×10^{-3}		0.139	13	IVW	

Table S10: CMR drugged proteins: MR effect estimates of plasma protein effects on sixteen CMR traits. *(continued)*

Protein (uniprot)	Gene (ensembl)	CMR trait	MD (95%CI)	P-value	Multiple testing threshold	Q p-value	No. variants	MR model	pQTL source
		LV – PER (ml/s)	-0.32 (-2.99; 2.36)	8.2×10^{-1}		0.680	13	IVW	
		LV – PAFR (ml/s)	-2.65 (-6.26; 0.96)	1.5×10^{-1}		0.125	13	IVW	
		LV – MVR (g/ml)	0.00 (0.00; 0.01)	2.1×10^{-2}		0.851	13	IVW	
		LV – ESV (ml)	-0.47 (-0.91; -0.03)	3.5×10^{-2}		0.381	13	IVW	
		LV – EF (%)	-0.12 (-0.30; 0.05)	1.7×10^{-1}		0.491	13	IVW	
		LV – EDV (ml)	-1.48 (-2.20; -0.75)	6.3×10^{-5}		0.639	13	IVW	
		LV – EDM (g)	-0.49 (-0.88; -0.09)	1.6×10^{-2}		0.515	13	IVW	
CD33 (P20138)	CD33 (ENSG00000105383)	RV – SV (ml)	0.00 (-0.11; 0.11)	9.8×10^{-1}	7.81×10^{-6}	<0.001	48	IVW	Interval
		RV – PFR (ml/s)	-0.17 (-1.29; 0.95)	7.7×10^{-1}		0.851	51	MR Egger	
		RV – PER (ml/s)	-1.39 (-2.48; -0.31)	1.2×10^{-2}		<0.001	53	MR Egger	
		RV – PAFR (ml/s)	0.51 (-0.88; 1.89)	4.7×10^{-1}		0.101	53	MR Egger	
		RV – ESV (ml)	0.21 (-0.01; 0.44)	6.3×10^{-2}		0.286	51	MR Egger	
		RV – EF (%)	-0.11 (-0.20; -0.03)	7.6×10^{-3}		0.341	51	MR Egger	
		RV – EDV (ml)	0.06 (-0.27; 0.39)	7.4×10^{-1}		0.002	52	MR Egger	
		LV – SV (ml)	0.14 (-0.08; 0.35)	2.1×10^{-1}		0.027	50	MR Egger	
		LV – PFR (ml/s)	-0.46 (-1.75; 0.82)	4.8×10^{-1}		0.002	51	MR Egger	
		LV – PER (ml/s)	0.53 (-0.06; 1.11)	7.8×10^{-2}		0.687	53	IVW	
		LV – PAFR (ml/s)	0.65 (-0.14; 1.44)	1.1×10^{-1}		0.129	48	IVW	
		LV – MVR (g/ml)	0.00 (0.00; 0.00)	5.9×10^{-5}		0.038	51	MR Egger	
		LV – ESV (ml)	0.25 (0.05; 0.45)	1.3×10^{-2}		<0.001	51	MR Egger	
		LV – EF (%)	-0.03 (-0.12; 0.06)	4.8×10^{-1}		<0.001	50	MR Egger	
		LV – EDV (ml)	0.34 (0.16; 0.52)	2.0×10^{-4}		<0.001	47	IVW	
		LV – EDM (g)	0.62 (0.45; 0.80)	4.8×10^{-12}		<0.001	52	MR Egger	
CAH6 (P23280)	CA6 (ENSG00000131686)	RV – SV (ml)	0.12 (0.00; 0.24)	4.6×10^{-2}	7.81×10^{-6}	<0.001	50	IVW	Interval
		RV – PFR (ml/s)	1.54 (0.84; 2.24)	1.5×10^{-5}		<0.001	44	IVW	
		RV – PER (ml/s)	-0.38 (-1.07; 0.31)	2.8×10^{-1}		<0.001	45	IVW	
		RV – PAFR (ml/s)	-2.01 (-2.82; -1.19)	1.3×10^{-6}		0.137	50	IVW	
		RV – ESV (ml)	0.14 (-0.23; 0.51)	4.5×10^{-1}		0.056	49	MR Egger	
		RV – EF (%)	0.03 (-0.09; 0.16)	6.2×10^{-1}		0.032	47	MR Egger	
		RV – EDV (ml)	0.19 (-0.40; 0.78)	5.3×10^{-1}		0.059	50	MR Egger	
		LV – SV (ml)	0.05 (-0.31; 0.40)	8.0×10^{-1}		0.067	51	MR Egger	
		LV – PFR (ml/s)	1.06 (0.34; 1.77)	3.7×10^{-3}		0.003	50	IVW	
		LV – PER (ml/s)	0.65 (-0.05; 1.35)	6.8×10^{-2}		0.006	50	IVW	
		LV – PAFR (ml/s)	-1.87 (-4.00; 0.25)	8.4×10^{-2}		0.377	50	MR Egger	
		LV – MVR (g/ml)	-0.00 (-0.00; -0.00)	4.0×10^{-5}		0.002	49	IVW	
		LV – ESV (ml)	0.26 (-0.07; 0.59)	1.2×10^{-1}		0.061	51	MR Egger	
		LV – EF (%)	-0.03 (-0.08; 0.02)	3.0×10^{-1}		0.082	49	IVW	
		LV – EDV (ml)	0.31 (-0.23; 0.84)	2.6×10^{-1}		0.153	51	MR Egger	
		LV – EDM (g)	-0.07 (-0.18; 0.04)	2.3×10^{-1}		0.543	48	IVW	

General:
CMR: Cardiac MRI, MR: Mendelian randomization, MD: mean difference, CI: confidence interval. Effect estimates are coded towards protein and CMR increasing directions.

Table S11: CMR druggable proteins: ChEMBL and HPA annotations.

Protein (uniprot)	Compound	Drug type	Target type	Drug action	Clinical development phase	Curated indications	mRNA Tissue specificity	Above average mRNA expression
CASP8 (Q14790)	Nivocasan	Small_Mol	Single Protein	Inhibitor	2		0.776	Granulocytes, Spleen, Thymus, Tonsil
CASP8 (Q14790)	Emricasan	Small_Mol	Protein Family	Inhibitor	2	Diabetes Mellitus	0.776	Granulocytes, Spleen, Thymus, Tonsil
DKK1 (O94907)	Bhq-880	Bio_Mol, Small_Mol	Single Protein	Inhibitor	2		0.986	Placenta
ERAP1 (Q9NZ08)	Tosedostat	Bio_Mol	Protein Family	Inhibitor	2		0.704	Pituitary gland, Small intestine, Thymus
ERAP2 (Q6P179)	Tosedostat	Small_Mol	Protein Family	Inhibitor	2		0.727	Colon, Lymph node, Spleen, Thymus
FCER2 (P06734)	Lumiliximab	Bio_Mol, Small_Mol	Single Protein	Antagonist	2		0.968	B-cells, Spleen, Tonsil
GFRA1 (P56159)	Liatermin	Bio_Mol	Single Protein	Agonist	1		0.747	Adipose tissue, Breast, Epididymis, Liver
ICOSL (O75144)	Amg-557	Bio_Mol	Single Protein	Inhibitor	1		0.783	Bone marrow, Hippocampal formation, Spinal cord
IL8 (P10145)	Abx-Il8, Humax-Il8	Bio_Mol, Small_Mol	Single Protein	Inhibitor	2	Bronchitis, Chronic	0.976	Bone marrow, Lung
LYAM1 (P14151)	Rivipansel, Aselizumab	Bio_Mol, Small_Mol	Single Protein	Antagonist, Inhibitor	3	Anemia, Sickle Cell	0.935	Granulocytes, NK-cells
LYAM1 (P14151)	Bimosiamose	Bio_Mol, Small_Mol	Protein Family	Inhibitor	2	Pulmonary Disease, Chronic Obstructive	0.935	Granulocytes, NK-cells
MFGM (Q08431)	Bre-3 90Y	Bio_Mol	Single Protein	Binding Agent	1		0.823	Breast, Endometrium, Heart muscle, Vagina
MK03 (P27361)	Ravoxertinib, Mk-8353, Ulixertinib	Small_Mol	Single Protein	Inhibitor	2		0.650	Amygdala, Cerebral cortex, Small intestine
MMP9 (P14780)	Marimastat, Andecaliximab	Bio_Mol, Small_Mol	Single Protein	Inhibitor	3	Pulmonary Disease, Chronic Obstructive	0.916	Adipose tissue, Bone marrow, Granulocytes, Lymph node, Spleen
PA2GA (P14555)	Varespladib, Varespladib Methyl	Bio_Mol, Small_Mol	Single Protein	Inhibitor	3	Acute Coronary Syndrome; Anemia, Sickle Cell; Coronary Artery Disease	0.962	Liver
PD1L2 (Q9BQ51)	Rhigm12B7	Bio_Mol	Single Protein	Cross-Linking Agent	1		0.806	Adipose tissue, Lung, Placenta, Spleen
PMEL (P40967)	Imc-Gp100	Bio_Mol	Single Protein	Cross-Linking Agent	0		0.990	Retina
TDGF1 (P13385)	Biib-015	Bio_Mol	Single Protein	Binding Agent	1		0.925	Kidney, Ovary, Spleen
TGFB2 (P61812)	Lerdelimumab, Fresolimumab	Bio_Mol	Single Protein	Inhibitor	3	Idiopathic Pulmonary Fibrosis	0.774	Placenta, Prostate, Seminal vesicle, Spinal cord
TLR4 (O00206)	Eritoran Tetrasodium	Bio_Mol, Small_Mol	Protein Complex	Antagonist	3		0.753	Adipose tissue, Granulocytes, Lymph node, Spleen
TNF12 (O43508)	Ro-5458640, Biib-023	Bio_Mol	Single Protein	Inhibitor	1		0.592	Adipose tissue, Vagina
TNR5 (P25942)	Dacetuzumab, Teneliximab, Pg-102, Lucatumumab, Cp-870893	Bio_Mol	Single Protein	Inhibitor, Partial Agonist, Antagonist, Agonist	2		0.781	Appendix, B-cells, Lymph node, Spleen, Tonsil
TR10B (O14763)	Tigatuzumab, Dulanermin, Conatumumab, Drozitumab, Apomab, Lexatumumab, Lby-135	Bio_Mol	Single Protein	Agonist	3		0.676	Adipose tissue, Liver, Small intestine

General:
Data were extracted from ChEMBL, and Human Protein Atlas. Indicates were curated for cardiovascular relevant traits. Tissues with above average mRNA expression were selected based on a z-statistic of at least 1.96. Tissue specificity ranges between 0 (ubiquitous expressed) and 1 (specifically expressed).

Table S12: CMR druggable proteins: MR effect estimates of plasma protein effects on sixteen CMR traits.

Protein (uniprot)	Gene (ensembl)	CMR trait	MD (95%CI)	P-value	Multiple testing threshold	Q p-value	No. variants	MR model	pQTL source
TDGF1 (P13385)	TDGF1 (ENSG00000241186)	RV – SV (ml)	0.24 (0.18; 0.30)	1.8×10^{-15}	7.81×10^{-6}	<0.001	56	IVW	Interval
		RV – PFR (ml/s)	1.41 (0.94; 1.89)	4.1×10^{-9}		0.002	58	MR Egger	
		RV – PER (ml/s)	1.34 (0.93; 1.76)	3.0×10^{-10}		<0.001	55	IVW	
		RV – PAFR (ml/s)	0.61 (0.12; 1.11)	1.6×10^{-2}		<0.001	48	IVW	
		RV – ESV (ml)	-0.01 (-0.08; 0.06)	7.7×10^{-1}		0.019	46	IVW	
		RV – EF (%)	0.08 (0.05; 0.10)	4.6×10^{-10}		0.011	51	IVW	
		RV – EDV (ml)	0.20 (0.10; 0.30)	5.7×10^{-5}		<0.001	54	IVW	
		LV – SV (ml)	0.33 (0.24; 0.43)	8.6×10^{-12}		<0.001	57	MR Egger	
		LV – PFR (ml/s)	0.61 (0.25; 0.98)	9.4×10^{-4}		0.030	56	IVW	
		LV – PER (ml/s)	1.22 (0.77; 1.68)	1.6×10^{-7}		<0.001	60	MR Egger	
		LV – PAFR (ml/s)	0.76 (0.26; 1.26)	2.7×10^{-3}		<0.001	58	MR Egger	
		LV – MVR (g/ml)	0.00 (0.00; 0.00)	1.6×10^{-3}		0.016	50	IVW	
		LV – ESV (ml)	-0.18 (-0.25; -0.12)	5.4×10^{-8}		0.010	47	IVW	
		LV – EF (%)	0.08 (0.05; 0.10)	8.0×10^{-9}		<0.001	48	IVW	
		LV – EDV (ml)	-0.17 (-0.27; -0.07)	9.3×10^{-4}		0.003	50	IVW	
		LV – EDM (g)	-0.11 (-0.16; -0.05)	2.2×10^{-4}		0.013	49	IVW	
PA2GA (P14555)	PLA2G2A (ENSG00000188257)	RV – SV (ml)	-0.30 (-0.45; -0.15)	8.0×10^{-5}	7.81×10^{-6}	0.032	52	MR Egger	Interval
		RV – PFR (ml/s)	-1.47 (-2.33; -0.61)	7.8×10^{-4}		<0.001	51	MR Egger	
		RV – PER (ml/s)	-0.73 (-1.77; 0.32)	1.7×10^{-1}		0.019	46	MR Egger	
		RV – PAFR (ml/s)	-1.43 (-2.50; -0.37)	8.4×10^{-3}		<0.001	50	MR Egger	
		RV – ESV (ml)	0.40 (0.20; 0.60)	9.0×10^{-5}		0.339	48	MR Egger	
		RV – EF (%)	-0.14 (-0.20; -0.08)	9.4×10^{-6}		0.036	51	MR Egger	
		RV – EDV (ml)	0.13 (-0.14; 0.40)	3.5×10^{-1}		0.029	53	MR Egger	
		LV – SV (ml)	0.28 (0.21; 0.34)	1.0×10^{-100}		0.015	55	IVW	
		LV – PFR (ml/s)	0.35 (-0.18; 0.88)	2.0×10^{-1}		<0.001	52	IVW	
		LV – PER (ml/s)	-2.70 (-3.60; -1.81)	2.9×10^{-9}		0.013	49	MR Egger	
		LV – PAFR (ml/s)	-2.65 (-3.89; -1.40)	3.0×10^{-5}		0.052	53	MR Egger	
		LV – MVR (g/ml)	-0.00 (-0.00; -0.00)	1.0×10^{-100}		0.003	50	MR Egger	
		LV – ESV (ml)	0.57 (0.49; 0.64)	1.0×10^{-100}		<0.001	51	IVW	
		LV – EF (%)	-0.15 (-0.18; -0.12)	1.0×10^{-100}		0.403	55	IVW	
		LV – EDV (ml)	0.69 (0.42; 0.96)	4.1×10^{-7}		<0.001	54	MR Egger	
		LV – EDM (g)	0.06 (-0.02; 0.13)	1.3×10^{-1}		<0.001	48	IVW	
TNR5 (P25942)	CD40 (ENSG00000101017)	RV – SV (ml)	0.67 (0.22; 1.13)	3.8×10^{-3}	7.81×10^{-6}	0.063	47	MR Egger	Scallop
		RV – PFR (ml/s)	4.52 (3.54; 5.49)	1.0×10^{-100}		0.046	49	IVW	
		RV – PER (ml/s)	2.71 (0.11; 5.31)	4.1×10^{-2}		0.081	48	MR Egger	
		RV – PAFR (ml/s)	2.79 (1.39; 4.18)	8.9×10^{-5}		<0.001	44	IVW	
		RV – ESV (ml)	0.84 (0.62; 1.06)	4.1×10^{-14}		<0.001	45	IVW	
		RV – EF (%)	0.16 (-0.01; 0.33)	7.1×10^{-2}		0.011	45	MR Egger	
		RV – EDV (ml)	1.05 (0.64; 1.45)	3.3×10^{-7}		0.093	49	IVW	
		LV – SV (ml)	0.76 (0.55; 0.96)	4.2×10^{-13}		<0.001	49	IVW	
		LV – PFR (ml/s)	0.90 (-1.79; 3.60)	5.1×10^{-1}		0.071	51	MR Egger	
		LV – PER (ml/s)	-1.70 (-4.09; 0.69)	1.6×10^{-1}		<0.001	47	MR Egger	
		LV – PAFR (ml/s)	-0.98 (-3.66; 1.70)	4.7×10^{-1}		<0.001	49	MR Egger	
		LV – MVR (g/ml)	-0.00 (-0.00; 0.00)	6.5×10^{-2}		0.005	50	MR Egger	
		LV – ESV (ml)	0.68 (0.47; 0.88)	7.0×10^{-11}		0.025	45	IVW	
		LV – EF (%)	0.17 (0.01; 0.34)	4.1×10^{-2}		<0.001	46	MR Egger	
		LV – EDV (ml)	0.60 (-0.28; 1.48)	1.8×10^{-1}		<0.001	42	MR Egger	
		LV – EDM (g)	0.23 (0.05; 0.40)	1.1×10^{-2}		0.032	47	IVW	
TNF12 (O43508)	TNFSF12 (ENSG00000239697)	RV – SV (ml)	-1.22 (-2.14; -0.30)	9.3×10^{-3}	7.81×10^{-6}	0.052	15	MR Egger	Interval
		RV – PFR (ml/s)	-7.51 (-11.28; -3.73)	9.6×10^{-5}		0.006	15	MR Egger	
		RV – PER (ml/s)	-1.21 (-2.49; 0.06)	6.2×10^{-2}		0.005	14	IVW	
		RV – PAFR (ml/s)	-0.29 (-1.70; 1.12)	6.9×10^{-1}		0.929	17	IVW	
		RV – ESV (ml)	-1.54 (-2.35; -0.73)	1.9×10^{-4}		0.145	16	MR Egger	
		RV – EF (%)	0.21 (0.11; 0.32)	1.0×10^{-4}		0.076	17	IVW	
		RV – EDV (ml)	-2.70 (-4.14; -1.26)	2.4×10^{-4}		0.102	15	MR Egger	
		LV – SV (ml)	-1.57 (-2.29; -0.85)	2.0×10^{-5}		<0.001	15	MR Egger	
		LV – PFR (ml/s)	-3.16 (-4.53; -1.79)	5.9×10^{-6}		0.010	17	IVW	
		LV – PER (ml/s)	-3.48 (-7.41; 0.45)	8.3×10^{-2}		0.031	15	MR Egger	
		LV – PAFR (ml/s)	-1.83 (-3.32; -0.34)	1.6×10^{-2}		0.587	17	IVW	
		LV – MVR (g/ml)	0.01 (0.00; 0.01)	1.0×10^{-100}		0.042	17	IVW	
		LV – ESV (ml)	-0.82 (-1.05; -0.58)	7.7×10^{-12}		0.256	16	IVW	
		LV – EF (%)	0.20 (0.11; 0.28)	1.1×10^{-5}		0.567	17	IVW	
		LV – EDV (ml)	-0.98 (-1.35; -0.62)	1.2×10^{-7}		0.002	16	IVW	

Table S12: CMR druggable proteins: MR effect estimates of plasma protein effects on sixteen CMR traits. *(continued)*

Protein (uniprot)	Gene (ensembl)	CMR trait	MD (95%CI)	P-value	Multiple testing threshold	Q p-value	No. variants	MR model	pQTL source
		LV – EDM (g)	0.20 (0.01; 0.40)	4.3×10^{-2}		0.050	17	IVW	
ERAP2 (Q6P179)	ERAP2 (ENSG00000164308)	RV – SV (ml)	-0.17 (-0.35; 0.01)	6.7×10^{-2}	7.81×10^{-6}	0.002	45	MR Egger	Interval
		RV – PFR (ml/s)	-1.35 (-2.41; -0.28)	1.3×10^{-2}		<0.001	42	MR Egger	
		RV – PER (ml/s)	-0.23 (-0.46; 0.01)	6.1×10^{-2}		<0.001	57	IVW	
		RV – PAFR (ml/s)	0.13 (-0.65; 0.90)	7.5×10^{-1}		<0.001	55	MR Egger	
		RV – ESV (ml)	-0.34 (-0.46; -0.23)	1.5×10^{-9}		0.039	54	MR Egger	
		RV – EF (%)	0.10 (0.05; 0.15)	8.1×10^{-5}		<0.001	54	MR Egger	
		RV – EDV (ml)	-0.35 (-0.58; -0.12)	2.7×10^{-3}		<0.001	47	MR Egger	
		LV – SV (ml)	-0.01 (-0.14; 0.12)	8.6×10^{-1}		0.084	54	MR Egger	
		LV – PFR (ml/s)	1.90 (1.66; 2.14)	1.0×10^{-100}		<0.001	54	IVW	
		LV – PER (ml/s)	-0.10 (-0.37; 0.17)	4.7×10^{-1}		<0.001	53	IVW	
		LV – PAFR (ml/s)	-0.97 (-1.88; -0.05)	3.9×10^{-2}		0.198	54	MR Egger	
		LV – MVR (g/ml)	0.00 (-0.00; 0.00)	7.0×10^{-1}		0.031	56	MR Egger	
		LV – ESV (ml)	-0.00 (-0.12; 0.11)	9.4×10^{-1}		0.104	57	MR Egger	
		LV – EF (%)	-0.00 (-0.02; 0.02)	9.3×10^{-1}		0.650	54	IVW	
		LV – EDV (ml)	-0.27 (-0.33; -0.20)	4.4×10^{-16}		0.003	56	IVW	
		LV – EDM (g)	-0.22 (-0.31; -0.12)	6.7×10^{-6}		<0.001	57	MR Egger	
MMP9 (P14780)	MMP9 (ENSG00000100985)	RV – SV (ml)	0.09 (-0.57; 0.74)	8.0×10^{-1}	7.81×10^{-6}	0.583	6	IVW	Framingham
		RV – PFR (ml/s)	2.13 (-1.45; 5.71)	2.4×10^{-1}		0.394	6	IVW	
		RV – PER (ml/s)	-3.96 (-7.62; -0.30)	3.4×10^{-2}		0.470	6	IVW	
		RV – PAFR (ml/s)	-2.59 (-7.83; 2.66)	3.3×10^{-1}		0.157	6	IVW	
		RV – ESV (ml)	0.98 (0.21; 1.75)	1.3×10^{-2}		0.256	6	IVW	
		RV – EF (%)	-0.35 (-0.60; -0.09)	7.5×10^{-3}		0.890	6	IVW	
		RV – EDV (ml)	1.08 (-0.18; 2.35)	9.3×10^{-2}		0.242	6	IVW	
		LV – SV (ml)	0.28 (-0.39; 0.96)	4.1×10^{-1}		0.724	6	IVW	
		LV – PFR (ml/s)	-0.84 (-4.86; 3.18)	6.8×10^{-1}		0.894	6	IVW	
		LV – PER (ml/s)	1.33 (-2.61; 5.28)	5.1×10^{-1}		0.971	6	IVW	
		LV – PAFR (ml/s)	-2.92 (-7.31; 1.46)	1.9×10^{-1}		0.667	6	IVW	
		LV – MVR (g/ml)	-0.01 (-0.01; -0.01)	1.4×10^{-6}		0.974	6	IVW	
		LV – ESV (ml)	1.43 (0.80; 2.06)	7.7×10^{-6}		0.832	6	IVW	
		LV – EF (%)	-0.59 (-0.85; -0.34)	5.8×10^{-6}		0.859	6	IVW	
		LV – EDV (ml)	1.67 (0.60; 2.73)	2.2×10^{-3}		0.725	6	IVW	
		LV – EDM (g)	-0.40 (-0.99; 0.18)	1.8×10^{-1}		0.593	6	IVW	
LYAM1 (P14151)	SELL (ENSG00000188404)	RV – SV (ml)	0.08 (-0.28; 0.44)	6.5×10^{-1}	7.81×10^{-6}	0.014	35	MR Egger	Interval
		RV – PFR (ml/s)	-0.77 (-3.02; 1.48)	5.0×10^{-1}		0.086	35	MR Egger	
		RV – PER (ml/s)	0.86 (-1.62; 3.34)	5.0×10^{-1}		0.059	34	MR Egger	
		RV – PAFR (ml/s)	1.55 (-1.84; 4.95)	3.7×10^{-1}		0.102	32	MR Egger	
		RV – ESV (ml)	0.40 (0.26; 0.55)	3.2×10^{-8}		0.542	34	IVW	
		RV – EF (%)	0.10 (-0.04; 0.24)	1.7×10^{-1}		0.679	34	MR Egger	
		RV – EDV (ml)	-0.10 (-0.80; 0.61)	7.9×10^{-1}		0.089	34	MR Egger	
		LV – SV (ml)	0.51 (0.14; 0.88)	7.3×10^{-3}		0.482	35	MR Egger	
		LV – PFR (ml/s)	2.85 (1.67; 4.02)	2.1×10^{-6}		0.050	32	IVW	
		LV – PER (ml/s)	-0.78 (-2.96; 1.39)	4.8×10^{-1}		0.467	35	MR Egger	
		LV – PAFR (ml/s)	-2.40 (-5.95; 1.15)	1.9×10^{-1}		0.014	32	MR Egger	
		LV – MVR (g/ml)	0.00 (-0.00; 0.00)	3.3×10^{-1}		0.442	34	MR Egger	
		LV – ESV (ml)	-0.81 (-1.32; -0.31)	1.6×10^{-3}		0.065	33	MR Egger	
		LV – EF (%)	0.36 (0.21; 0.52)	4.6×10^{-6}		0.253	34	MR Egger	
		LV – EDV (ml)	0.47 (0.19; 0.74)	8.2×10^{-4}		0.005	31	IVW	
		LV – EDM (g)	-0.16 (-0.55; 0.23)	4.2×10^{-1}		0.043	34	MR Egger	
TR10B (O14763)	TNFRSF10B (ENSG00000120889)	RV – SV (ml)	0.17 (-0.12; 0.46)	2.5×10^{-1}	7.81×10^{-6}	0.648	39	IVW	Scallop
		RV – PFR (ml/s)	-4.54 (-7.87; -1.21)	7.6×10^{-3}		0.531	42	MR Egger	
		RV – PER (ml/s)	-1.42 (-3.36; 0.51)	1.5×10^{-1}		<0.001	36	IVW	
		RV – PAFR (ml/s)	0.63 (-1.20; 2.46)	5.0×10^{-1}		0.006	38	IVW	
		RV – ESV (ml)	0.29 (-0.45; 1.02)	4.4×10^{-1}		0.196	39	MR Egger	
		RV – EF (%)	-0.03 (-0.17; 0.11)	6.7×10^{-1}		0.172	34	IVW	
		RV – EDV (ml)	-0.17 (-1.37; 1.03)	7.8×10^{-1}		0.661	40	MR Egger	
		LV – SV (ml)	-0.71 (-1.05; -0.36)	5.9×10^{-5}		0.070	39	IVW	
		LV – PFR (ml/s)	-4.36 (-6.13; -2.59)	1.4×10^{-6}		0.008	39	IVW	
		LV – PER (ml/s)	-2.98 (-6.93; 0.98)	1.4×10^{-1}		0.278	42	MR Egger	
		LV – PAFR (ml/s)	4.05 (2.09; 6.02)	5.1×10^{-5}		0.025	38	IVW	
		LV – MVR (g/ml)	0.01 (0.01; 0.01)	1.0×10^{-100}		0.002	36	IVW	
		LV – ESV (ml)	-0.31 (-0.99; 0.37)	3.8×10^{-1}		0.083	42	MR Egger	

Table S12: CMR druggable proteins: MR effect estimates of plasma protein effects on sixteen CMR traits. *(continued)*

Protein (uniprot)	Gene (ensembl)	CMR trait	MD (95%CI)	P-value	Multiple testing threshold	Q p-value	No. variants	MR model	pQTL source
		LV – EF (%)	-0.00 (-0.12; 0.11)	9.6×10^{-1}		0.007	38	IVW	
		LV – EDV (ml)	-0.57 (-1.75; 0.62)	3.5×10^{-1}		0.083	41	MR Egger	
		LV – EDM (g)	1.32 (0.65; 1.99)	1.1×10^{-4}		0.085	41	MR Egger	
TLR4 (O00206)	TLR4 (ENSG00000136869)	RV – SV (ml)	-0.44 (-1.07; 0.19)	1.7×10^{-1}	7.81×10^{-6}	0.692	16	MR Egger	Interval
		RV – PFR (ml/s)	1.51 (0.04; 2.98)	4.5×10^{-2}		0.240	19	IVW	
		RV – PER (ml/s)	-2.09 (-6.51; 2.33)	3.5×10^{-1}		0.185	16	MR Egger	
		RV – PAFR (ml/s)	-2.48 (-4.08; -0.88)	2.4×10^{-3}		0.678	18	IVW	
		RV – ESV (ml)	-0.91 (-1.74; -0.07)	3.3×10^{-2}		0.281	14	MR Egger	
		RV – EF (%)	-0.01 (-0.13; 0.10)	7.9×10^{-1}		0.150	19	IVW	
		RV – EDV (ml)	-1.84 (-3.35; -0.32)	1.8×10^{-2}		0.103	15	MR Egger	
		LV – SV (ml)	-0.61 (-0.87; -0.35)	4.0×10^{-6}		0.920	18	IVW	
		LV – PFR (ml/s)	-0.28 (-1.81; 1.26)	7.2×10^{-1}		0.447	19	IVW	
		LV – PER (ml/s)	-5.44 (-9.37; -1.51)	6.7×10^{-3}		0.009	16	MR Egger	
		LV – PAFR (ml/s)	-2.82 (-7.30; 1.66)	2.2×10^{-1}		0.285	16	MR Egger	
		LV – MVR (g/ml)	0.00 (-0.00; 0.00)	2.4×10^{-1}		0.065	18	IVW	
		LV – ESV (ml)	-0.42 (-0.71; -0.14)	3.8×10^{-3}		0.104	19	IVW	
		LV – EF (%)	0.02 (-0.08; 0.11)	7.4×10^{-1}		0.015	19	IVW	
		LV – EDV (ml)	-1.06 (-1.47; -0.65)	3.4×10^{-7}		0.445	19	IVW	
		LV – EDM (g)	-0.49 (-0.75; -0.22)	3.1×10^{-4}		0.111	19	IVW	
TGFB2 (P61812)	TGFB2 (ENSG00000092969)	RV – SV (ml)	0.58 (-0.51; 1.67)	3.0×10^{-1}	7.81×10^{-6}	0.892	2	IVW	Interval
		RV – PFR (ml/s)	-0.87 (-6.72; 4.98)	7.7×10^{-1}		0.479	2	IVW	
		RV – PER (ml/s)	0.76 (-5.33; 6.85)	8.1×10^{-1}		0.419	2	IVW	
		RV – PAFR (ml/s)	3.74 (-3.19; 10.66)	2.9×10^{-1}		0.777	2	IVW	
		RV – ESV (ml)	0.79 (-0.33; 1.91)	1.6×10^{-1}		0.774	2	IVW	
		RV – EF (%)	-0.30 (-0.72; 0.12)	1.7×10^{-1}		0.829	2	IVW	
		RV – EDV (ml)	1.44 (-0.37; 3.25)	1.2×10^{-1}		0.856	2	IVW	
		LV – SV (ml)	2.87 (1.75; 3.99)	4.9×10^{-7}		0.859	2	IVW	
		LV – PFR (ml/s)	2.67 (-4.03; 9.37)	4.3×10^{-1}		0.450	2	IVW	
		LV – PER (ml/s)	10.98 (2.74; 19.21)	9.0×10^{-3}		0.210	2	IVW	
		LV – PAFR (ml/s)	-2.27 (-9.57; 5.04)	5.4×10^{-1}		0.958	2	IVW	
		LV – MVR (g/ml)	-0.01 (-0.01; 0.00)	7.0×10^{-2}		0.647	2	IVW	
		LV – ESV (ml)	1.19 (0.15; 2.24)	2.5×10^{-2}		0.963	2	IVW	
		LV – EF (%)	0.46 (0.04; 0.89)	3.4×10^{-2}		0.759	2	IVW	
		LV – EDV (ml)	4.06 (2.28; 5.84)	7.6×10^{-6}		0.879	2	IVW	
		LV – EDM (g)	1.61 (0.63; 2.58)	1.2×10^{-3}		0.548	2	IVW	
PD1L2 (Q9BQ51)	PDCD1LG2 (ENSG00000197646)	RV – SV (ml)	-0.16 (-0.83; 0.51)	6.3×10^{-1}	7.81×10^{-6}	0.556	33	MR Egger	Interval
		RV – PFR (ml/s)	-1.65 (-5.25; 1.95)	3.7×10^{-1}		0.747	33	MR Egger	
		RV – PER (ml/s)	-1.49 (-5.82; 2.85)	5.0×10^{-1}		0.102	32	MR Egger	
		RV – PAFR (ml/s)	4.74 (0.29; 9.19)	3.7×10^{-2}		0.327	33	MR Egger	
		RV – ESV (ml)	-0.10 (-0.33; 0.13)	3.8×10^{-1}		0.083	30	IVW	
		RV – EF (%)	-0.13 (-0.42; 0.15)	3.7×10^{-1}		0.206	33	MR Egger	
		RV – EDV (ml)	0.08 (-1.18; 1.34)	9.0×10^{-1}		0.139	33	MR Egger	
		LV – SV (ml)	-0.43 (-0.62; -0.24)	6.6×10^{-6}		0.748	32	IVW	
		LV – PFR (ml/s)	-2.09 (-6.20; 2.02)	3.2×10^{-1}		0.917	33	MR Egger	
		LV – PER (ml/s)	1.20 (-2.84; 5.23)	5.6×10^{-1}		0.812	33	MR Egger	
		LV – PAFR (ml/s)	3.21 (-1.67; 8.09)	2.0×10^{-1}		0.220	33	MR Egger	
		LV – MVR (g/ml)	0.00 (-0.00; 0.01)	3.2×10^{-1}		0.891	33	MR Egger	
		LV – ESV (ml)	-0.45 (-0.66; -0.23)	3.8×10^{-5}		0.087	30	IVW	
		LV – EF (%)	0.12 (0.03; 0.21)	5.8×10^{-3}		0.091	30	IVW	
		LV – EDV (ml)	-0.78 (-1.10; -0.47)	9.6×10^{-7}		0.298	32	IVW	
		LV – EDM (g)	-0.17 (-0.34; -0.01)	4.1×10^{-2}		0.531	30	IVW	
MK03 (P27361)	MAPK3 (ENSG00000102882)	RV – SV (ml)	-0.48 (-1.43; 0.46)	3.2×10^{-1}	7.81×10^{-6}	0.136	5	IVW	Interval
		RV – PFR (ml/s)	5.06 (1.23; 8.88)	9.6×10^{-3}		0.510	5	IVW	
		RV – PER (ml/s)	2.72 (-1.27; 6.70)	1.8×10^{-1}		0.597	5	IVW	
		RV – PAFR (ml/s)	-6.10 (-10.63; -1.58)	8.2×10^{-3}		0.945	5	IVW	
		RV – ESV (ml)	-2.07 (-2.80; -1.34)	3.0×10^{-8}		0.563	5	IVW	
		RV – EF (%)	0.75 (0.47; 1.02)	1.3×10^{-7}		0.027	5	IVW	
		RV – EDV (ml)	-2.43 (-3.61; -1.24)	6.0×10^{-5}		0.960	5	IVW	
		LV – SV (ml)	-0.46 (-1.25; 0.34)	2.6×10^{-1}		0.320	5	IVW	
		LV – PFR (ml/s)	3.59 (-0.78; 7.96)	1.1×10^{-1}		0.460	5	IVW	
		LV – PER (ml/s)	46.63 (19.37; 73.89)	8.0×10^{-4}		0.773	5	MR Egger	
		LV – PAFR (ml/s)	6.31 (1.54; 11.08)	9.5×10^{-3}		0.430	5	IVW	

Table S12: CMR druggable proteins: MR effect estimates of plasma protein effects on sixteen CMR traits. *(continued)*

Protein (uniprot)	Gene (ensembl)	CMR trait	MD (95%CI)	P-value	Multiple testing threshold	Q p-value	No. variants	MR model	pQTL source
		LV – MVR (g/ml)	0.01 (0.00; 0.01)	3.6×10^{-4}		0.287	5	IVW	
		LV – ESV (ml)	2.77 (-1.56; 7.10)	2.1×10^{-1}		0.846	5	MR Egger	
		LV – EF (%)	0.43 (0.15; 0.71)	2.5×10^{-3}		0.709	5	IVW	
		LV – EDV (ml)	3.66 (-3.71; 11.04)	3.3×10^{-1}		0.791	5	MR Egger	
		LV – EDM (g)	-0.05 (-0.68; 0.59)	8.9×10^{-1}		0.689	5	IVW	
MFGM (Q08431)	MFGE8 (ENSG00000140545)	RV – SV (ml)	0.11 (-0.20; 0.42)	4.9×10^{-1}	7.81×10^{-6}	0.359	12	IVW	Interval
		RV – PFR (ml/s)	4.27 (2.67; 5.86)	1.6×10^{-7}		0.934	12	IVW	
		RV – PER (ml/s)	-0.54 (-2.60; 1.53)	6.1×10^{-1}		0.106	12	IVW	
		RV – PAFR (ml/s)	5.80 (3.91; 7.68)	1.7×10^{-9}		0.563	12	IVW	
		RV – ESV (ml)	0.56 (-1.69; 2.82)	6.2×10^{-1}		0.706	12	MR Egger	
		RV – EF (%)	-0.04 (-0.17; 0.10)	6.0×10^{-1}		0.223	12	IVW	
		RV – EDV (ml)	0.34 (-0.15; 0.84)	1.7×10^{-1}		0.615	12	IVW	
		LV – SV (ml)	0.28 (-0.02; 0.59)	7.1×10^{-2}		0.662	12	IVW	
		LV – PFR (ml/s)	-6.32 (-19.79; 7.15)	3.6×10^{-1}		0.468	12	MR Egger	
		LV – PER (ml/s)	-7.16 (-20.39; 6.06)	2.9×10^{-1}		0.868	12	MR Egger	
		LV – PAFR (ml/s)	1.56 (-0.61; 3.73)	1.6×10^{-1}		0.284	12	IVW	
		LV – MVR (g/ml)	0.00 (-0.00; 0.00)	8.3×10^{-1}		0.879	12	IVW	
		LV – ESV (ml)	-0.75 (-2.85; 1.36)	4.9×10^{-1}		0.797	12	MR Egger	
		LV – EF (%)	-0.28 (-1.25; 0.70)	5.8×10^{-1}		0.238	12	MR Egger	
		LV – EDV (ml)	-2.24 (-5.82; 1.34)	2.2×10^{-1}		0.870	12	MR Egger	
		LV – EDM (g)	-2.24 (-4.20; -0.28)	2.5×10^{-2}		0.884	12	MR Egger	
IL8 (P10145)	IL8 (ENSG00000169429)	RV – SV (ml)	1.99 (0.93; 3.05)	2.4×10^{-4}	7.81×10^{-6}	0.271	4	IVW	Scallop
		RV – PFR (ml/s)	9.25 (4.27; 14.23)	2.7×10^{-4}		0.581	4	IVW	
		RV – PER (ml/s)	12.63 (7.45; 17.81)	1.8×10^{-6}		0.047	4	IVW	
		RV – PAFR (ml/s)	-12.19 (-18.22; -6.16)	7.4×10^{-5}		0.369	4	IVW	
		RV – ESV (ml)	0.35 (-0.60; 1.30)	4.8×10^{-1}		0.923	4	IVW	
		RV – EF (%)	0.29 (-0.18; 0.76)	2.3×10^{-1}		0.161	4	IVW	
		RV – EDV (ml)	2.47 (0.93; 4.01)	1.7×10^{-3}		0.753	4	IVW	
		LV – SV (ml)	1.48 (0.41; 2.56)	6.9×10^{-3}		0.279	4	IVW	
		LV – PFR (ml/s)	3.86 (-1.83; 9.55)	1.8×10^{-1}		0.878	4	IVW	
		LV – PER (ml/s)	3.84 (-1.75; 9.43)	1.8×10^{-1}		0.898	4	IVW	
		LV – PAFR (ml/s)	-5.87 (-12.80; 1.06)	9.7×10^{-2}		0.291	4	IVW	
		LV – MVR (g/ml)	0.01 (0.01; 0.02)	3.3×10^{-5}		0.838	4	IVW	
		LV – ESV (ml)	0.15 (-0.74; 1.04)	7.4×10^{-1}		0.651	4	IVW	
		LV – EF (%)	0.04 (-0.49; 0.58)	8.7×10^{-1}		0.091	4	IVW	
		LV – EDV (ml)	1.32 (-0.19; 2.83)	8.6×10^{-2}		0.886	4	IVW	
		LV – EDM (g)	2.34 (1.51; 3.16)	3.1×10^{-8}		0.768	4	IVW	
ERAP1 (Q9NZ08)	ERAP1 (ENSG00000164307)	RV – SV (ml)	-0.13 (-0.25; -0.00)	4.3×10^{-2}	7.81×10^{-6}	<0.001	50	MR Egger	Interval
		RV – PFR (ml/s)	0.02 (-0.67; 0.70)	9.6×10^{-1}		<0.001	49	MR Egger	
		RV – PER (ml/s)	0.66 (0.40; 0.93)	9.7×10^{-7}		0.233	61	IVW	
		RV – PAFR (ml/s)	-0.65 (-0.95; -0.35)	2.5×10^{-5}		0.050	64	IVW	
		RV – ESV (ml)	0.12 (0.01; 0.23)	3.0×10^{-2}		0.007	62	MR Egger	
		RV – EF (%)	-0.05 (-0.08; -0.02)	3.2×10^{-3}		<0.001	50	IVW	
		RV – EDV (ml)	-0.05 (-0.15; 0.05)	3.6×10^{-1}		<0.001	50	IVW	
		LV – SV (ml)	0.03 (-0.02; 0.08)	2.9×10^{-1}		<0.001	59	IVW	
		LV – PFR (ml/s)	-1.61 (-2.39; -0.83)	4.9×10^{-5}		<0.001	49	MR Egger	
		LV – PER (ml/s)	0.48 (0.21; 0.75)	4.0×10^{-4}		<0.001	61	IVW	
		LV – PAFR (ml/s)	0.18 (-0.56; 0.92)	6.3×10^{-1}		<0.001	58	MR Egger	
		LV – MVR (g/ml)	-0.00 (-0.00; -0.00)	8.0×10^{-8}		0.011	64	MR Egger	
		LV – ESV (ml)	0.09 (0.04; 0.13)	8.5×10^{-5}		0.003	61	IVW	
		LV – EF (%)	0.01 (-0.03; 0.04)	5.9×10^{-1}		0.008	65	MR Egger	
		LV – EDV (ml)	0.09 (0.01; 0.17)	1.9×10^{-2}		0.023	58	IVW	
		LV – EDM (g)	-0.07 (-0.11; -0.03)	2.8×10^{-4}		0.027	61	IVW	
PMEL (P40967)	PMEL (ENSG00000185664)	RV – SV (ml)	-0.09 (-1.06; 0.88)	8.5×10^{-1}	7.81×10^{-6}	0.716	2	IVW	Interval
		RV – PFR (ml/s)	-11.98 (-17.19; -6.77)	6.6×10^{-6}		0.890	2	IVW	
		RV – PER (ml/s)	-5.36 (-10.78; 0.07)	5.3×10^{-2}		0.764	2	IVW	
		RV – PAFR (ml/s)	5.74 (-0.42; 11.90)	6.8×10^{-2}		0.747	2	IVW	
		RV – ESV (ml)	-0.67 (-1.67; 0.32)	1.8×10^{-1}		0.654	2	IVW	
		RV – EF (%)	0.14 (-0.24; 0.52)	4.7×10^{-1}		0.633	2	IVW	
		RV – EDV (ml)	-0.56 (-2.17; 1.06)	5.0×10^{-1}		0.855	2	IVW	
		LV – SV (ml)	-0.23 (-1.23; 0.76)	6.5×10^{-1}		0.933	2	IVW	
		LV – PFR (ml/s)	-2.12 (-8.08; 3.84)	4.9×10^{-1}		0.777	2	IVW	

Table S12: CMR druggable proteins: MR effect estimates of plasma protein effects on sixteen CMR traits. *(continued)*

Protein (uniprot)	Gene (ensembl)	CMR trait	MD (95%CI)	P-value	Multiple testing threshold	Q p-value	No. variants	MR model	pQTL source
		LV – PER (ml/s)	-6.92 (-12.77; -1.07)	2.1×10^{-2}		0.891	2	IVW	
		LV – PAFR (ml/s)	10.26 (3.76; 16.77)	2.0×10^{-3}		0.942	2	IVW	
		LV – MVR (g/ml)	0.00 (-0.00; 0.01)	6.5×10^{-1}		0.815	2	IVW	
		LV – ESV (ml)	-1.57 (-2.50; -0.64)	9.4×10^{-4}		0.914	2	IVW	
		LV – EF (%)	0.46 (0.07; 0.84)	1.9×10^{-2}		0.879	2	IVW	
		LV – EDV (ml)	-1.86 (-3.44; -0.27)	2.2×10^{-2}		0.899	2	IVW	
		LV – EDM (g)	-0.80 (-1.67; 0.06)	6.8×10^{-2}		0.695	2	IVW	
ICOSL (O75144)	ICOSLG (ENSG00000160223)	RV – SV (ml)	-0.07 (-0.66; 0.52)	8.2×10^{-1}	7.81×10^{-6}	0.295	37	MR Egger	Interval
		RV – PFR (ml/s)	2.65 (-0.19; 5.49)	6.8×10^{-2}		<0.001	40	MR Egger	
		RV – PER (ml/s)	4.38 (1.27; 7.48)	5.8×10^{-3}		0.028	36	MR Egger	
		RV – PAFR (ml/s)	-4.66 (-8.02; -1.30)	6.5×10^{-3}		0.661	40	MR Egger	
		RV – ESV (ml)	0.34 (-0.22; 0.90)	2.3×10^{-1}		0.047	37	MR Egger	
		RV – EF (%)	0.03 (-0.19; 0.26)	7.7×10^{-1}		0.413	38	MR Egger	
		RV – EDV (ml)	0.31 (-0.60; 1.22)	5.1×10^{-1}		0.452	38	MR Egger	
		LV – SV (ml)	0.31 (-0.29; 0.91)	3.1×10^{-1}		0.170	40	MR Egger	
		LV – PFR (ml/s)	-0.41 (-3.66; 2.84)	8.1×10^{-1}		0.017	40	MR Egger	
		LV – PER (ml/s)	1.13 (-2.06; 4.32)	4.9×10^{-1}		0.532	40	MR Egger	
		LV – PAFR (ml/s)	-1.60 (-5.14; 1.95)	3.8×10^{-1}		0.816	40	MR Egger	
		LV – MVR (g/ml)	-0.00 (-0.00; -0.00)	9.2×10^{-14}		0.222	42	IVW	
		LV – ESV (ml)	-0.45 (-0.97; 0.07)	9.1×10^{-2}		<0.001	36	MR Egger	
		LV – EF (%)	0.24 (0.03; 0.45)	2.8×10^{-2}		0.038	37	MR Egger	
		LV – EDV (ml)	0.06 (-0.82; 0.94)	9.0×10^{-1}		<0.001	39	MR Egger	
		LV – EDM (g)	-0.28 (-0.80; 0.25)	3.0×10^{-1}		0.154	40	MR Egger	
GFRA1 (P56159)	GFRA1 (ENSG00000151892)	RV – SV (ml)	0.19 (-0.28; 0.65)	4.3×10^{-1}	7.81×10^{-6}	0.933	12	IVW	Interval
		RV – PFR (ml/s)	2.06 (-0.42; 4.55)	1.0×10^{-1}		0.451	12	IVW	
		RV – PER (ml/s)	-0.42 (-2.86; 2.03)	7.4×10^{-1}		0.893	12	IVW	
		RV – PAFR (ml/s)	-4.04 (-6.82; -1.27)	4.3×10^{-3}		0.998	13	IVW	
		RV – ESV (ml)	0.47 (-0.06; 1.00)	8.2×10^{-2}		0.020	11	IVW	
		RV – EF (%)	-0.05 (-0.24; 0.13)	5.7×10^{-1}		0.298	12	IVW	
		RV – EDV (ml)	0.61 (-0.42; 1.64)	2.5×10^{-1}		0.155	11	IVW	
		LV – SV (ml)	0.07 (-0.46; 0.60)	7.8×10^{-1}		0.540	11	IVW	
		LV – PFR (ml/s)	4.79 (1.32; 8.26)	6.9×10^{-3}		0.128	12	IVW	
		LV – PER (ml/s)	-0.64 (-3.75; 2.47)	6.9×10^{-1}		0.484	11	IVW	
		LV – PAFR (ml/s)	-1.73 (-4.93; 1.46)	2.9×10^{-1}		0.390	12	IVW	
		LV – MVR (g/ml)	-0.01 (-0.01; -0.00)	4.0×10^{-6}		0.506	10	IVW	
		LV – ESV (ml)	0.50 (-0.00; 0.99)	5.2×10^{-2}		0.420	10	IVW	
		LV – EF (%)	-0.18 (-0.35; -0.01)	3.7×10^{-2}		0.613	13	IVW	
		LV – EDV (ml)	0.71 (-0.13; 1.55)	9.7×10^{-2}		0.824	10	IVW	
		LV – EDM (g)	-0.60 (-1.01; -0.18)	4.6×10^{-3}		0.920	12	IVW	
FCER2 (P06734)	FCER2 (ENSG00000104921)	RV – SV (ml)	-0.70 (-1.43; 0.02)	5.8×10^{-2}	7.81×10^{-6}	0.991	26	MR Egger	Interval
		RV – PFR (ml/s)	-2.49 (-6.40; 1.42)	2.1×10^{-1}		0.814	26	MR Egger	
		RV – PER (ml/s)	-3.19 (-7.26; 0.88)	1.2×10^{-1}		0.679	26	MR Egger	
		RV – PAFR (ml/s)	-3.64 (-8.26; 0.98)	1.2×10^{-1}		0.466	26	MR Egger	
		RV – ESV (ml)	-0.24 (-1.04; 0.57)	5.7×10^{-1}		0.269	26	MR Egger	
		RV – EF (%)	-0.13 (-0.41; 0.15)	3.6×10^{-1}		0.598	26	MR Egger	
		RV – EDV (ml)	-0.89 (-2.10; 0.32)	1.5×10^{-1}		0.708	26	MR Egger	
		LV – SV (ml)	-0.27 (-1.02; 0.48)	4.8×10^{-1}		0.919	26	MR Egger	
		LV – PFR (ml/s)	-3.89 (-8.36; 0.59)	8.8×10^{-2}		0.735	26	MR Egger	
		LV – PER (ml/s)	-3.66 (-8.05; 0.73)	1.0×10^{-1}		0.960	26	MR Egger	
		LV – PAFR (ml/s)	-1.93 (-6.95; 3.08)	4.5×10^{-1}		0.385	26	MR Egger	
		LV – MVR (g/ml)	-0.00 (-0.01; 0.00)	2.1×10^{-1}		0.575	26	MR Egger	
		LV – ESV (ml)	-0.35 (-0.56; -0.14)	1.0×10^{-3}		0.026	24	IVW	
		LV – EF (%)	-0.05 (-0.13; 0.04)	2.8×10^{-1}		0.332	24	IVW	
		LV – EDV (ml)	-0.93 (-2.12; 0.26)	1.2×10^{-1}		0.656	26	MR Egger	
		LV – EDM (g)	-0.62 (-0.89; -0.36)	2.8×10^{-6}		0.217	20	IVW	
DKK1 (O94907)	DKK1 (ENSG00000107984)	RV – SV (ml)	-0.20 (-1.31; 0.91)	7.3×10^{-1}	7.81×10^{-6}	0.484	9	IVW	Scallop
		RV – PFR (ml/s)	16.29 (10.33; 22.24)	8.3×10^{-8}		0.600	9	IVW	
		RV – PER (ml/s)	-3.57 (-9.77; 2.63)	2.6×10^{-1}		0.961	9	IVW	
		RV – PAFR (ml/s)	-6.24 (-13.72; 1.24)	1.0×10^{-1}		0.340	9	IVW	
		RV – ESV (ml)	-2.08 (-3.22; -0.94)	3.4×10^{-4}		0.736	9	IVW	
		RV – EF (%)	0.58 (0.09; 1.06)	1.9×10^{-2}		0.265	9	IVW	
		RV – EDV (ml)	-2.35 (-4.19; -0.50)	1.3×10^{-2}		0.755	9	IVW	

Table S12: CMR druggable proteins: MR effect estimates of plasma protein effects on sixteen CMR traits. *(continued)*

Protein (uniprot)	Gene (ensembl)	CMR trait	MD (95%CI)	P-value	Multiple testing threshold	Q p-value	No. variants	MR model	pQTL source
		LV – SV (ml)	-1.26 (-2.47; -0.05)	4.2×10^{-2}		0.332	9	IVW	
		LV – PFR (ml/s)	7.62 (-0.04; 15.29)	5.1×10^{-2}		0.254	9	IVW	
		LV – PER (ml/s)	4.16 (-4.18; 12.51)	3.3×10^{-1}		0.131	9	IVW	
		LV – PAFR (ml/s)	-0.25 (-7.67; 7.18)	9.5×10^{-1}		0.793	9	IVW	
		LV – MVR (g/ml)	0.01 (0.01; 0.02)	3.8×10^{-5}		0.433	9	IVW	
		LV – ESV (ml)	-1.98 (-3.21; -0.75)	1.6×10^{-3}		0.217	9	IVW	
		LV – EF (%)	0.63 (0.13; 1.12)	1.3×10^{-2}		0.248	9	IVW	
		LV – EDV (ml)	-3.23 (-5.15; -1.31)	9.8×10^{-4}		0.339	9	IVW	
		LV – EDM (g)	-0.22 (-1.21; 0.77)	6.6×10^{-1}		0.586	9	IVW	
CASP8 (Q14790)	CASP8 (ENSG00000064012)	RV – SV (ml)	0.15 (-2.84; 3.14)	9.2×10^{-1}	7.81×10^{-6}	0.416	9	MR Egger	Scallop
		RV – PFR (ml/s)	13.28 (7.55; 19.01)	5.5×10^{-6}		0.824	9	IVW	
		RV – PER (ml/s)	-3.51 (-9.47; 2.45)	2.5×10^{-1}		0.670	9	IVW	
		RV – PAFR (ml/s)	-6.99 (-13.76; -0.21)	4.3×10^{-2}		0.908	9	IVW	
		RV – ESV (ml)	-0.48 (-1.86; 0.91)	5.0×10^{-1}		0.120	9	IVW	
		RV – EF (%)	0.11 (-0.36; 0.58)	6.5×10^{-1}		0.688	8	IVW	
		RV – EDV (ml)	0.36 (-1.45; 2.18)	6.9×10^{-1}		0.398	9	IVW	
		LV – SV (ml)	0.12 (-1.02; 1.27)	8.3×10^{-1}		0.361	9	IVW	
		LV – PFR (ml/s)	9.46 (2.52; 16.40)	7.6×10^{-3}		0.345	9	IVW	
		LV – PER (ml/s)	2.09 (-4.35; 8.53)	5.2×10^{-1}		0.789	9	IVW	
		LV – PAFR (ml/s)	-5.94 (-13.09; 1.22)	1.0×10^{-1}		0.684	9	IVW	
		LV – MVR (g/ml)	-0.01 (-0.01; -0.00)	1.3×10^{-2}		0.839	9	IVW	
		LV – ESV (ml)	1.40 (-1.53; 4.33)	3.5×10^{-1}		0.384	9	MR Egger	
		LV – EF (%)	-0.45 (-1.62; 0.71)	4.5×10^{-1}		0.897	9	MR Egger	
		LV – EDV (ml)	-1.43 (-3.55; 0.68)	1.8×10^{-1}		0.159	9	IVW	
		LV – EDM (g)	0.55 (-2.24; 3.35)	7.0×10^{-1}		0.351	9	MR Egger	

General:
CMR: Cardiac MRI, MR: Mendelian randomization, MD: mean difference, CI: confidence interval. Effect estimates are coded towards protein and CMR increasing directions.

Table S13: Annotating the set of directionally concordant CMR proteins.

Protein (uniprot)	Gene (ensembl)	mRNA Tissue specificity	Above average mRNA expression	Druggability	Nearest druggable prot.	Prot. distance
TDGF1 (P13385)	TDGF1 (ENSG00000241186)	0.925	Kidney, Ovary, Spleen	Druggable		
OSMR (Q99650)	OSMR (ENSG00000145623)	0.693	Adipose tissue, Placenta, Urinary bladder, Vagina	Not druggable	ADRM1 (Q16186)	2
					FA10 (P00742)	2
					S39A6 (Q13433)	2
					RS27A (P62979)	2
					BMP10 (O95393)	2
					FZD7 (O75084)	2
					PAI1 (P05121)	2
					PTGDS (P41222)	2
					IL6RB (P40189)	2
					CO4A5 (P29400)	2
					IL6RA (P08887)	2
					ITAM (P11215)	2
					PEN2 (Q9NZ42)	2
					PSN1 (P49768)	2
					CD20 (P11836)	2
					COMT (P21964)	2
					OSTP (P10451)	2
BAG3 (O95817)	BAG3 (ENSG00000151929)	0.906	Skeletal muscle, Vagina	Not druggable	HSP7C (P11142)	1
						1
MANBA (O00462)	MANBA (ENSG00000109323)	0.544	Granulocytes, Parathyroid gland, Placenta, Salivary gland	Not druggable		
ENTP1 (P49961)	ENTPD1 (ENSG00000138185)	0.808	Endometrium, Granulocytes, Smooth muscle	Not druggable	OPRM (P35372)	2
					ITB2 (P05107)	2
					A4 (P05067)	2
					MET (P08581)	2
PGLT1 (Q8NBL1)	POGLUT1 (ENSG00000163389)	0.620	Ductus deferens, Pancreas, Seminal vesicle	Not druggable		
					OPRM (P35372)	2
IL18R (Q13478)	IL18R1 (ENSG00000115604)	0.834	Lung, Lymph node, NK-cells, Seminal vesicle	Not druggable	IL18 (Q14116)	1
GPC5 (P78333)	GPC5 (ENSG00000179399)	0.844	Basal ganglia, Cerebral cortex, Kidney, Pons and medulla	Not druggable	SYUA (P37840)	2
PRDX1 (Q06830)	PRDX1 (ENSG00000117450)	0.559	Esophagus, Kidney, Liver, Thyroid gland	Not druggable	EGFR (P00533)	1
					EGFR (P00533)	1
					ANDR (P10275)	1
UD16 (P19224)	UGT1A6 (ENSG00000167165)	0.978	Kidney, Liver	Not druggable		
BGH3 (Q15582)	TGFBI (ENSG00000120708)	0.751	Epididymis, Gallbladder, Placenta	Not druggable		
ASAH2 (Q9NR71)	ASAH2 (ENSG00000188611)	0.970	Duodenum, Small intestine	Not druggable		
ERAP2 (Q6P179)	ERAP2 (ENSG00000164308)	0.727	Colon, Lymph node, Spleen, Thymus	Druggable		
					TPH1 (P17752)	2
NET1 (O95631)	NTN1 (ENSG00000065320)	0.823	Esophagus, Gallbladder, Heart muscle, Retina	Not druggable	RL5 (P46777)	2
TREM1 (Q9NP99)	TREM1 (ENSG00000124731)	0.957	Bone marrow, Granulocytes, Lung	Not druggable		
					CASP9 (P55211)	2
					RL28 (P46779)	2
ISK2 (P20155)	SPINK2 (ENSG00000128040)	0.987	Epididymis	Not druggable	A4 (P05067)	2
					P85A (P27986)	2
					MALT1 (Q9UDY8)	2
					AMPQ (Q6Q4G3)	2
					CASP1 (P29466)	2
					LYAM2 (P16581)	2
CATB (P07858)	CTSB (ENSG00000164733)	0.825	Adipose tissue, Liver, Lymph node, Thyroid gland	Not druggable	PSA1 (P25786)	2
					PSA1 (P25786)	2
					PRS10 (P62333)	2
					HSP7C (P11142)	2

Table S13: Annotating the set of directionally concordant CMR proteins. *(continued)*

Protein (uniprot)	Gene (ensembl)	mRNA Tissue specificity	Above average mRNA expression	Druggability	Nearest druggable prot.	Prot. distance
					PSB8 (P28062)	2
PPAC (P24666)	ACP1 (ENSG00000143727)	0.472	Adrenal gland, Liver, Pancreas	Not druggable	5HT1E (P28566)	2
					PSA3 (P25788)	2
					RSSA (P08865)	2
TPSNR (Q9BX59)	TAPBPL (ENSG00000139192)	0.668	Monocytes, NK-cells, Small intestine, Total PBMC	Not druggable		
					RL3 (P39023)	2
BSSP4 (Q9GZN4)	PRSS22 (ENSG00000005001)	0.921	Esophagus, Tongue, Tonsil	Not druggable		
EPHA1 (P21709)	EPHA1 (ENSG00000146904)	0.967	Parathyroid gland	Drugged	M3K1 (Q13233)	2
					RS15A (P62244)	2
KAT3 (Q6YP21)	CCBL2 (ENSG00000137944)	0.340	NA	Not druggable		
NCAM2 (O15394)	NCAM2 (ENSG00000154654)	0.903	Amygdala, Cerebral cortex, Thalamus	Not druggable		
RMD1 (Q96DB5)	RMDN1 (ENSG00000176623)	0.589	Granulocytes, Heart muscle, Kidney, Skeletal muscle	Not druggable		
ASM3A (Q92484)	SMPDL3A (ENSG00000172594)	0.785	Colon, Skeletal muscle, Small intestine	Not druggable		
CHLE (P06276)	BCHE (ENSG00000114200)	0.965	Liver	Drugged	CASP6 (P55212)	1
					HCK (P08631)	2
					PSB1 (P20618)	2
					HDA10 (Q969S8)	2
					PDE4D (Q08499)	2
					RARA (P10276)	2
					VWF (P04275)	2
PATE4 (P0C8F1)	PATE4 (ENSG00000237353)	0.984	Ductus deferens, Seminal vesicle	Not druggable		
					P2RY4 (P51582)	2
					P2RY6 (Q15077)	2
					PRS7 (P35998)	2
SPA12 (Q8IW75)	SERPINA12 (ENSG00000165953)	0.998	Skin	Not druggable		
C1QC (P02747)	C1QC (ENSG00000159189)	0.907	Lymph node, Spleen	Not druggable		
					DNM3A (Q9Y6K1)	2
					ESR1 (P03372)	2
					KEAP1 (Q14145)	2
					TBB6 (Q9BUF5)	2
					TNNI3 (P19429)	2
					NDUS1 (P28331)	2
					NDUF4 (Q9P032)	2
					GBRG1 (Q8N1C3)	2
TIE2 (Q02763)	TEK (ENSG00000120156)	0.808	Adipose tissue, Kidney, Lung, Placenta, Spleen	Drugged	ANGP2 (O15123)	1

General:
Proteins were prioritized by selecting proteins who were associated with at least three CMR traits, and without directionally discordant effects. Data were extracted from Reactome, ChEMBL, and Human Protein Atlas. Tissues with above average mRNA expression were selected based on a z-statistic of at least 1.96. Tissue specificity ranges between 0 (ubiquitous expressed) and 1 (specifically expressed). The nearest druggable protein was identified by linking with Reactome and extracting the nearest proteins that met our definition of druggable (see main text). Distance reflect how many protein interactions (represented by edges in a graph, with one an adjacent protein) these a druggable protein was from the index protein.

Table S14: Directionally concordant CMR proteins and their (next) nearest druggable protein.

Protein (uniprot)	Nearest druggable prot.	Prot. distance	Compound	Drug type	Drug action	Clinical development phase	Curated indications	Curated side effects
OSMR (Q99650)	ADRM1 (Q16186)	2	Carfilzomib, Bortezomib, Ixazomib Citrate, Oprozomib	Small_Mol, Bio_Mol	Inhibitor	4	Amyloidosis; Amyloidosis, Familial; Anemia, Refractory, with Excess of Blasts	Anaemia; Cardiac arrest; QT interval prolongation; acute coronary syndrome; amyloidosis; arrhythmias; ascites; atrioventricular block; cardiac arrest; cardiomyopathy; cardiovascular disorder; cerebrovascular insufficiency; chest discomfort; chest pain; circulatory collapse; coagulation disorders; coma; coronary artery insufficiency; diabetes mellitus; dyspnoea; embolism and thrombosis; haemorrhage; heart failure; hyperglycaemia; hypertension; hyperthyroidism; hypotension; inflammation; ischaemic heart disease; multi organ failure; myocardial infarction; myocardial ischaemia; myopathy; oedema; pain; palpitations; pericardial disorders; pericardial effusion; pericarditis; pulmonary hypertension; pulmonary oedema; respiratory disorders; stroke; syncope; vascular disorders; ventricular dysfunction
	FA10 (P00742)	2	Emicizumab	Bio_Mol, Small_Mol	Other	4		embolism and thrombosis
	S39A6 (Q13433)	2		Bio_Mol				
	RS27A (P62979)	2	Dorlimomab Aritox	Bio_Mol	Inhibitor	1		
	BMP10 (O95393)	2	Dalantercept	Bio_Mol	Inhibitor	3		
	FZD7 (O75084)	2	Vantictumab	Bio_Mol	Antagonist	1		
	PAI1 (P05121)	2	Aleplasinin	Bio_Mol, Small_Mol	Inhibitor	1		
	PTGDS (P41222)	2	Antrafenine	Bio_Mol	Inhibitor	4		
	IL6RB (P40189)	2	Satralizumab	Bio_Mol	Antagonist	3		
	CO4A5 (P29400)	2	Collagenase Clostridium Histolyticum, Ocriplasmin	Bio_Mol	Hydrolytic Enzyme	4	Stroke; Venous Thrombosis	haemorrhage
	IL6RA (P08887)	2	Satralizumab	Bio_Mol	Antagonist	3		
	ITAM (P11215)	2	Rovelizumab	Bio_Mol	Antagonist	2		
	PEN2 (Q9NZ42)	2	Tarenflurbil, Semagacestat, Avagacestat, Begacestat	Small_Mol	Modulator, Inhibitor	3		
	PSN1 (P49768)	2	Tarenflurbil, Semagacestat, Avagacestat, Begacestat	Small_Mol	Modulator, Inhibitor	3		
	CD20 (P11836)	2	Yttrium Y 90 Ibritumomab Tiuxetan, Rituximab, Ofatumumab, Tositumomab, Obinutuzumab, Ocrelizumab, Ublituximab, Veltuzumab, Ibritumomab Tiuxetan, Tositumomab 131I, Ocaratuzumab, Lfb-R603, Fbt-A05	Bio_Mol	Binding Agent, Inhibitor, Other, Cross-Linking Agent	4	Anemia, Aplastic; Anemia, Hemolytic, Autoimmune; Anemia, Sickle Cell; Diabetes Mellitus, Type 1; Heart Failure; Idiopathic Pulmonary Fibrosis; Lung Diseases, Interstitial; Sarcoidosis	Acute coronary syndrome; Arrhythmias; Asthma; anaemia; angina pectoris; arrhythmias; cardiac disorder; chest pain; coagulation disorder; dyspnoea; heart failure; hypercholesterolaemia; hyperglycaemia; hypertension; hypotension; hypoxia; ischaemic heart disease; multi organ failure; myocardial infarction; oedema; pain; respiratory disorders
	COMT (P21964)	2	Tolcapone, Entacapone	Small_Mol	Inhibitor	4		Myocardial infarction; chest pain; ischaemic heart disease; syncope
	OSTP (P10451)	2	Ask-8007	Bio_Mol	Inhibitor	2		
BAG3 (O95817)	HSP7C (P11142)	1	Forigerimod Acetate	Bio_Mol	Inhibitor	3		

Table S14: Directionally concordant CMR proteins and their (next) nearest druggable protein. *(continued)*

Protein (uniprot)	Nearest druggable prot.	Prot. distance	Compound	Drug type	Drug action	Clinical development phase	Curated indications	Curated side effects
ENTP1 (P49961)	OPRM (P35372)	2	Hydromorphone Hydrochloride, Oxycodone Terephthalate, Loperamide Hydrochloride, Tramadol Hydrochloride, Alfentanil Hydrochloride, Difenoxin Hydrochloride, Diphenoxylate Hydrochloride, Fentanyl Hydrochloride, Fentanyl Citrate, Oxycodone Hydrochloride, Morphine Sulfate, Propoxyphene Napsylate, Alvimopan, Meperidine Hydrochloride, Methadone Hydrochloride, Propoxyphene Hydrochloride, Levorphanol Tartrate, Dihydrocodeine Bitartrate, Remifentanil Hydrochloride, Methylnaltrexone Bromide, Sufentanil Citrate, Fentanyl, Levomethadyl Acetate Hydrochloride, Buprenorphine Hydrochloride, Anileridine Hydrochloride, Dezocine, Anileridine Phosphate, Tapentadol Hydrochloride, Butorphanol Tartrate, Buprenorphine, Levallorphan Tartrate, Naloxegol Oxalate, Eluxadoline, Methylsamidorphan, Axelopran, Samidorphan, Faxeladol, Axomadol, Naltalimide, Frakefamide, Naldemedine, Bevenopran, Oliceridine, Naldemedine Tosylate, Oxycodone	Small_Mol	Agonist, Antagonist, Partial Agonist	4	Anemia, Sickle Cell; Hypertension, Pulmonary; Hypotension; Lung Diseases, Interstitial; Myocardial Infarction; Pre-Eclampsia; Respiratory Distress Syndrome, Adult	Arrhythmias; Dyspnoea; QT interval prolongation; angina pectoris; asthma; atrioventricular block; cardiac arrest; chest pain; circulatory collapse; coma; cyanosis; dyspnoea; haemorrhage; hypertension; hypoglycaemia; hypotension; oedema; pain; palpitations; respiratory disorders; syncope
	ITB2 (P05107)	2	Efalizumab, Lifitegrast, Rovelizumab	Bio_Mol, Small_Mol	Inhibitor, Antagonist	4	Diabetes Mellitus, Type 1	
	A4 (P05067)	2	Gantenerumab, Solanezumab, Bapineuzumab, Aducanumab, Crenezumab, Ponezumab, Gsk933776, Ban2401	Bio_Mol, Small_Mol	Other, Inhibitor	3		
	MET (P08581)	2	Cabozantinib S-Malate, Crizotinib, Bms-794833, Telisotuzumab, Amg-208, Arry-300, Bms-698769, Bms-817378, Altiratinib, Emd-1204831, Jnj-38877605, Merestinib, Mk-8033, Pf-04217903, Sar-125844, Sgx-523, Tas-115, Bpi-9016, Amg-337, Savolitinib, Bms-777607, Golvatinib, Foretinib, Mgcd-265, Mk-2461, Amuvatinib, Tepotinib, Capmatinib, Tivantinib, Onartuzumab, Emibetuzumab	Bio_Mol, Small_Mol	Inhibitor, Antagonist	4	Pulmonary Disease, Chronic Obstructive	Anaemia; QT interval prolongation; arrhythmias; heart failure; hypophosphataemia; oedema; pulmonary oedema; respiratory disorders; syncope
	OPRM (P35372)	2	Hydrocodone Polistirex, Codeine Polistirex, Oxymorphone Hydrochloride, Naltrexone, Codeine Phosphate, Codeine Sulfate, Naltrexone Hydrochloride, Nalbuphine Hydrochloride, Nalmefene Hydrochloride, Naloxone Hydrochloride, Hydrocodone Bitartrate, Odelepran, Cebranopadol, Dextromoramide	Small_Mol	Agonist, Antagonist	4	Respiratory Distress Syndrome, Adult	Arrhythmias; Cardiac arrest; chest pain; dyspnoea; hyperglycaemia; hypertension; hypotension; pain; palpitations; pulmonary oedema; tachycardia

Table S14: Directionally concordant CMR proteins and their (next) nearest druggable protein. *(continued)*

Protein (uniprot)	Nearest druggable prot.	Prot. distance	Compound	Drug type	Drug action	Clinical development phase	Curated indications	Curated side effects
IL18R (Q13478)	IL18 (Q14116)	1	Gsk-1070806, Medi-2338	Bio_Mol	Inhibitor, Cross-Linking Agent	2	Diabetes Mellitus; Pulmonary Disease, Chronic Obstructive	
GPC5 (P78333)	SYUA (P37840)	2	Biib054	Bio_Mol, Small_Mol	Inhibitor	2		
PRDX1 (Q06830)	EGFR (P00533)	1	Vandetanib, Osimertinib Mesylate, Dacomitinib, Poziotinib, Neratinib Maleate	Bio_Mol, Small_Mol	Inhibitor	4		
	EGFR (P00533)	1	Panitumumab, Cetuximab, Erlotinib Hydrochloride, Gefitinib, Lapatinib Ditosylate, Afatinib Dimaleate, Necitumumab, Ac-480, Allitinib, Azd-4769, Falnidamol, Cep-32496, Cudc-101, Olmutinib, Theliatinib, Epitinib, Jnj-26483327, Mp-412, Pd-0166285, Pki-166, S-222611, Tak-285, Puquitinib, Pyrotinib, Aee-788, Brigatinib, Varlitinib, Sapitinib, Bms-690514, Rociletinib, Pelitinib, Tesevatinib, Egf816, Osimertinib, Canertinib Dihydrochloride, Neratinib, Dacomitinib, Simotinib, Zalutumumab, Matuzumab, Mab-425, Mdx-447, Rg-7160, Nimotuzumab, Futuximab, Depatuxizumab Mafodotin, Duligotuzumab, Imgatuzumab, Ro-5083945, Zatuximab	Bio_Mol, Small_Mol	Inhibitor, Antagonist, Other, Binding Agent	4		Anaemia; Cardiac arrest; Embolism and thrombosis; QT interval prolongation; anaemia; arrhythmias; chest discomfort; chest pain; cyanosis; dyspnoea; embolism and thrombosis; haemorrhage; hyperglycaemia; hypertension; hypokalaemia; hypomagnesaemia; hypotension; interstitial lung disease; oedema; pain; palpitations; respiratory disorders; tachycardia
	ANDR (P10275)	1	Oxymetholone, Flutamide, Nilutamide, Dromostanolone Propionate, Bicalutamide, Methyltestosterone, Ethylestrenol, Stanozolol, Testosterone Propionate, Testosterone Enanthate, Nandrolone Decanoate, Oxandrolone, Testosterone Cypionate, Testosterone, Fluoxymesterone, Enzalutamide, Nandrolone Phenpropionate, Danazol, Testosterone Undecanoate, He3235, Gsk2881078, Gsk2849466, Vk5211, Apc-100, Glpg0492, Azd3514, Lgd-2941, Ly2452473, Mk-0773, Cb-03-01, Galeterone, Cr 1447, Odm-201, Enobosarm, Apalutamide, Methandrostenolone, Cyproterone Acetate, Abiraterone	Bio_Mol, Small_Mol	Agonist, Antagonist, Modulator	4	Hereditary angioedema; Anemia, Aplastic; Cerebral Arterial Diseases; Coronary Artery Disease; Coronary Disease; Heart Failure; Pulmonary Disease, Chronic Obstructive	Acute coronary syndrome; Anaemia; QT interval prolongation; anaemia; cardiovascular disorder; chest pain; dyslipidaemia; dyspnoea; embolism and thrombosis; heart failure; hypercholesterolaemia; hypertension; interstitial lung disease; ischaemic heart disease; myocardial infarction; oedema; pain; palpitations; respiratory disorders; tachycardia; thromboembolism
	TPH1 (P17752)	2	Telotristat, Telotristat Ethyl, Telotristat Etiprate	Small_Mol	Inhibitor	4	Carcinoid Heart Disease	
NET1 (O95631)	RL5 (P46777)	2	Dorlimomab Aritox	Bio_Mol	Inhibitor	1		
	CASP9 (P55211)	2	Emricasan	Small_Mol	Inhibitor	2	Diabetes Mellitus	
	RL28 (P46779)	2	Dorlimomab Aritox	Bio_Mol	Inhibitor	1		
ISK2 (P20155)	A4 (P05067)	2	Gantenerumab, Solanezumab, Bapineuzumab, Aducanumab, Crenezumab, Ponezumab, Gsk933776, Ban2401	Bio_Mol, Small_Mol	Other, Inhibitor	3		

Table S14: Directionally concordant CMR proteins and their (next) nearest druggable protein. *(continued)*

Protein (uniprot)	Nearest druggable prot.	Prot. distance	Compound	Drug type	Drug action	Clinical development phase	Curated indications	Curated side effects
	P85A (P27986)	2	Azd-6482, Ds-7423, Gsk-1059615, Omipalisib, Recilisib, Panulisib, Pa-799, Pwt-33587, Rg-7666, Sf-1126, Vs-5584, Wx-037, Puquitinib, Copanlisib, Dactolisib, Bgt-226, Buparlisib, Pf-04691502, Gedatolisib, Sonolisib, Pictilisib, Apitolisib, Taselisib, Pilaralisib, Voxtalisib, Zstk-474, Ly-3023414	Small_Mol	Inhibitor	4	Idiopathic Pulmonary Fibrosis	
	MALT1 (Q9UDY8)	2	Mepazine Acetate	Small_Mol	Inhibitor	4		
	AMPQ (Q6Q4G3)	2	Tosedostat	Small_Mol	Inhibitor	2		
	CASP1 (P29466)	2	Nivocasan	Small_Mol	Inhibitor	2		
	LYAM2 (P16581)	2	Rivipansel, Cdp-850	Bio_Mol, Small_Mol	Antagonist, Inhibitor	3	Anemia, Sickle Cell	
CATB (P07858)	PSA1 (P25786)	2	Marizomib	Small_Mol	Inhibitor	3		
	PSA1 (P25786)	2	Carfilzomib, Bortezomib, Ixazomib Citrate, Oprozomib	Small_Mol	Inhibitor	4	Amyloidosis; Amyloidosis, Familial; Anemia, Refractory, with Excess of Blasts	Anaemia; Cardiac arrest; QT interval prolongation; acute coronary syndrome; amyloidosis; arrhythmias; ascites; atrioventricular block; cardiac arrest; cardiomyopathy; cardiovascular disorder; cerebrovascular insufficiency; chest discomfort; chest pain; circulatory collapse; coagulation disorders; coma; coronary artery insufficiency; diabetes mellitus; dyspnoea; embolism and thrombosis; haemorrhage; heart failure; hyperglycaemia; hypertension; hyperthyroidism; hypotension; inflammation; ischaemic heart disease; multi organ failure; myocardial infarction; myocardial ischaemia; myopathy; oedema; pain; palpitations; pericardial disorders; pericardial effusion; pericarditis; pulmonary hypertension; pulmonary oedema; respiratory disorders; stroke; syncope; vascular disorders; ventricular dysfunction

Table S14: Directionally concordant CMR proteins and their (next) nearest druggable protein. *(continued)*

Protein (uniprot)	Nearest druggable prot.	Prot. distance	Compound	Drug type	Drug action	Clinical development phase	Curated indications	Curated side effects
	PRS10 (P62333)	2	Carfilzomib, Bortezomib, Ixazomib Citrate, Oprozomib	Small_Mol, Bio_Mol	Inhibitor	4	Amyloidosis; Amyloidosis, Familial; Anemia, Refractory, with Excess of Blasts	Anaemia; Cardiac arrest; QT interval prolongation; acute coronary syndrome; amyloidosis; arrhythmias; ascites; atrioventricular block; cardiac arrest; cardiomyopathy; cardiovascular disorder; cerebrovascular insufficiency; chest discomfort; chest pain; circulatory collapse; coagulation disorders; coma; coronary artery insufficiency; diabetes mellitus; dyspnoea; embolism and thrombosis; haemorrhage; heart failure; hyperglycaemia; hypertension; hyperthyroidism; hypotension; inflammation; ischaemic heart disease; multi organ failure; myocardial infarction; myocardial ischaemia; myopathy; oedema; pain; palpitations; pericardial disorders; pericardial effusion; pericarditis; pulmonary hypertension; pulmonary oedema; respiratory disorders; stroke; syncope; vascular disorders; ventricular dysfunction
	HSP7C (P11142)	2	Forigerimod Acetate	Bio_Mol	Inhibitor	3		
	PSB8 (P28062)	2	Marizomib	Small_Mol	Inhibitor	3		
PPAC (P24666)	5HT1E (P28566)	2	Dexfenfluramine Hydrochloride, Zimeldine Hydrochloride, Amisulpride	Bio_Mol, Small_Mol	Agonist, Antagonist	4		Hyperglycaemia; QT interval prolongation; arrhythmias; cardiac arrest; dyslipidaemia; embolism and thrombosis; hyponatraemia; hypotension
	PSA3 (P25788)	2	Carfilzomib, Bortezomib, Ixazomib Citrate, Oprozomib	Small_Mol	Inhibitor	4	Amyloidosis; Amyloidosis, Familial; Anemia, Refractory, with Excess of Blasts	Anaemia; Cardiac arrest; QT interval prolongation; acute coronary syndrome; amyloidosis; arrhythmias; ascites; atrioventricular block; cardiac arrest; cardiomyopathy; cardiovascular disorder; cerebrovascular insufficiency; chest discomfort; chest pain; circulatory collapse; coagulation disorders; coma; coronary artery insufficiency; diabetes mellitus; dyspnoea; embolism and thrombosis; haemorrhage; heart failure; hyperglycaemia; hypertension; hyperthyroidism; hypotension; inflammation; ischaemic heart disease; multi organ failure; myocardial infarction; myocardial ischaemia; myopathy; oedema; pain; palpitations; pericardial disorders; pericardial effusion; pericarditis; pulmonary hypertension; pulmonary oedema; respiratory disorders; stroke; syncope; vascular disorders; ventricular dysfunction
	RSSA (P08865)	2	Dorlimomab Aritox	Bio_Mol	Inhibitor	1		
	RL3 (P39023)	2	Dorlimomab Aritox	Bio_Mol	Inhibitor	1		
EPHA1 (P21709)	M3K1 (Q13233)	2	E-6201	Small_Mol	Inhibitor	2		
	RS15A (P62244)	2	Dorlimomab Aritox	Bio_Mol	Inhibitor	1		
CHLE (P06276)	CASP6 (P55212)	1	Emricasan	Small_Mol	Inhibitor	2	Diabetes Mellitus	

Table S14: Directionally concordant CMR proteins and their (next) nearest druggable protein. *(continued)*

Protein (uniprot)	Nearest druggable prot.	Prot. distance	Compound	Drug type	Drug action	Clinical development phase	Curated indications	Curated side effects
	HCK (P08631)	2	Bosutinib	Small_Mol	Inhibitor	4		Anaemia; QT interval prolongation; chest discomfort; dyspnoea; haemorrhage; hypertension; oedema; pain; pericardial effusion; pericarditis; pulmonary hypertension; pulmonary oedema; respiratory disorders
	PSB1 (P20618)	2	Marizomib	Small_Mol	Inhibitor	3		
	HDA10 (Q969S8)	2	Romidepsin, Belinostat, Panobinostat Lactate, Cudc-101, Fimepinostat, Tacedinaline, Entinostat	Small_Mol	Inhibitor	4		
	PDE4D (Q08499)	2	Pentoxifylline, Dipyridamole	Small_Mol	Inhibitor	4	Adjunct to oral anticoagulation for prophylaxis of thromboembolism associated with prosthetic heart valves; Secondary prevention of ischaemic stroke (not associated with atrial fibrillation) and transient ischaemic attacks (used alone or with aspirin); Secondary prevention of ischaemic stroke and transient ischaemic attacks; Acute Coronary Syndrome; Anemia; Anemia, Sickle Cell; Angina, Stable; Cardiovascular Diseases; Carotid Stenosis; Coronary Artery Disease; Coronary Disease; Fibrosis; Heart Diseases; Hypertension; Myocardial Ischemia; Stroke; Thrombosis	Angina pectoris; angina pectoris; arrhythmias; haemorrhage; hypotension; tachycardia
	RARA (P10276)	2	Alitretinoin, Acitretin, Etretinate, Mofarotene	Small_Mol	Agonist, Modulator	4		anaemia; haemorrhage; hypercholesterolaemia; hypertension
	VWF (P04275)	2	Caplacizumab	Bio_Mol	Inhibitor	3	Thrombosis	dyspnoea; haemorrhage
	P2RY4 (P51582)	2		Bio_Mol, Small_Mol				
	P2RY6 (Q15077)	2		Bio_Mol, Small_Mol				
	PRS7 (P35998)	2	Carfilzomib, Bortezomib, Ixazomib Citrate, Oprozomib	Small_Mol, Bio_Mol	Inhibitor	4	Amyloidosis; Amyloidosis, Familial; Anemia, Refractory, with Excess of Blasts	Anaemia; Cardiac arrest; QT interval prolongation; acute coronary syndrome; amyloidosis; arrhythmias; ascites; atrioventricular block; cardiac arrest; cardiomyopathy; cardiovascular disorder; cerebrovascular insufficiency; chest discomfort; chest pain; circulatory collapse; coagulation disorders; coma; coronary artery insufficiency; diabetes mellitus; dyspnoea; embolism and thrombosis; haemorrhage; heart failure; hyperglycaemia; hypertension; hyperthyroidism; hypotension; inflammation; ischaemic heart disease; multi organ failure; myocardial infarction; myocardial ischaemia; myopathy; oedema; pain; palpitations; pericardial disorders; pericardial effusion; pericarditis; pulmonary hypertension; pulmonary oedema; respiratory disorders; stroke; syncope; vascular disorders; ventricular dysfunction
	DNM3A (Q9Y6K1)	2	Azacitidine, Decitabine	Small_Mol	Inhibitor	4	Anemia, Refractory, with Excess of Blasts; Anemia, Sickle Cell; Thrombocytopenia	Anaemia; anaemia; chest pain; dyspnoea; haemorrhage; hypertension; hypokalaemia; hypotension; inflammation; interstitial lung disease; pain; respiratory disorders; syncope

Table S14: Directionally concordant CMR proteins and their (next) nearest druggable protein. *(continued)*

Protein (uniprot)	Nearest druggable prot.	Prot. distance	Compound	Drug type	Drug action	Clinical development phase	Curated indications	Curated side effects
	ESR1 (P03372)	2	Polyestradiol Phosphate, Tamoxifen Citrate, Estrone, Estradiol, Estradiol Acetate, Estradiol Valerate, Clomiphene Citrate, Ethinyl Estradiol, Dienestrol, Estradiol Cypionate, Estropipate, Mestranol, Diethylstilbestrol, Sr16234, Rad1901, Brilanestrant, Gtx-758	Small_Mol	Agonist, Modulator, Antagonist, Degrader	4	Atherosclerosis; Cerebral Arterial Diseases; Coronary Artery Disease; Diabetes Mellitus, Type 1; Diabetes Mellitus, Type 2; Fibrosis; Hypertension; Myocardial Ischemia	Cerebrovascular insufficiency; Hypertension; Venous thromboembolism; arterial thromboembolism; asthma; cerebrovascular insufficiency; chest pain; dyspnoea; embolism and thrombosis; haemorrhage; hypercalcaemia; hypertension; hypotension; myocardial infarction; oedema; pain; palpitations; vascular disorders; venous thromboembolism
	KEAP1 (Q14145)	2	Dimethyl Fumarate	Small_Mol	Inhibitor	4		
	TBB6 (Q9BUF5)	2	Trastuzumab Emtansine, Brentuximab Vedotin, Paclitaxel, Docetaxel, Eribulin Mesylate, Cabazitaxel, Colchicine, Ixabepilone, Vincristine Sulfate, Vinorelbine Tartrate, Vinblastine Sulfate, Paclitaxel Poliglumex, Indibulin, Davunetide, Crolibulin, Lexibulin, Vinflunine, Fosbretabulin Disodium, Fosbretabulin Tromethamine, Sagopilone, Plinabulin, Verubulin, Mirvetuximab Soravtansine	Bio_Mol, Small_Mol	Inhibitor, Stabiliser, Disrupting Agent	4	Acute Coronary Syndrome; Arrhythmias, Cardiac; Asthma; Atrial Fibrillation; Coronary Artery Disease; Coronary Restenosis; Heart Diseases; Ischemia; Lung Diseases; Myocardial Infarction; Pericarditis; Peripheral Arterial Disease; Pneumonia, Viral; ST Elevation Myocardial Infarction; Vascular Diseases	Anaemia; Ascites; Atrioventricular block; Respiratory disorders; anaemia; arrhythmia; arrhythmias; cardiac arrest; chest discomfort; chest pain; chest tightness; congestive heart failure; coronary artery disease; dyspnoea; embolism and thrombosis; haemorrhage; heart failure; hyperglycaemia; hypertension; hypoglycaemia; hypokalaemia; hyponatraemia; hypotension; multi organ failure; myocardial infarction; myocardial ischaemia; myopathy; oedema; pain; pericardial effusion; pulmonary oedema; respiratory disorders; stroke; syncope; tachycardia; venous thromboembolism; venous thrombosis
	TNNI3 (P19429)	2	Levosimendan	Small_Mol	Positive Modulator	3	Cardiovascular Diseases; Coronary Disease; Heart Failure; Ischemic Attack, Transient; ST Elevation Myocardial Infarction; Stroke	
	NDUS1 (P28331)	2	Metformin Hydrochloride, Me-344, Nv-128	Small_Mol	Inhibitor	4	Type 2 diabetes mellitus [monotherapy or in combination with other antidiabetic drugs (including insulin)]; Type 2 diabetes mellitus [reduction in risk or delay of onset]; Diabetes Mellitus; Diabetes Mellitus, Type 2; Heart Failure	
	NDUF4 (Q9P032)	2	Metformin Hydrochloride, Me-344, Nv-128	Small_Mol	Inhibitor	4	Type 2 diabetes mellitus [monotherapy or in combination with other antidiabetic drugs (including insulin)]; Type 2 diabetes mellitus [reduction in risk or delay of onset]; Diabetes Mellitus; Diabetes Mellitus, Type 2; Heart Failure	

Table S14: Directionally concordant CMR proteins and their (next) nearest druggable protein. *(continued)*

Protein (uniprot)	Nearest druggable prot.	Prot. distance	Compound	Drug type	Drug action	Clinical development phase	Curated indications	Curated side effects
	GBRG1 (Q8N1C3)	2	Methoxyflurane, Flumazenil, Clorazepate Dipotassium, Chlordiazepoxide, Chlordiazepoxide Hydrochloride, Diazepam, Eszopiclone, Oxazepam, Meprobamate, Clobazam, Alprazolam, Triazolam, Butalbital, Clonazepam, Desflurane, Estazolam, Talbutal, Butabarbital Sodium, Glutethimide, Acamprosate Calcium, Lorazepam, Enflurane, Ethchlorvynol, Metharbital, Methohexital Sodium, Methyprylon, Midazolam Hydrochloride, Prazepam, Primidone, Propofol, Quazepam, Pentobarbital, Pentobarbital Sodium, Secobarbital Sodium, Sevoflurane, Temazepam, Thiamylal Sodium, Thiopental Sodium, Etomidate, Flurazepam Hydrochloride, Halazepam, Halothane, Isoflurane, Topiramate, Triclofos Sodium, Adipiplon, Lorediplon, Resequinil, Pf-06372865, Ganaxolone, Pagoclone, Flunitrazepam, Methaqualone, Methaqualone Hydrochloride, Tetrazepam, Clomethiazole, Clotiazepam	Bio_Mol, Small_Mol	Positive Modulator, Allosteric Antagonist, Positive Allosteric Modulator, Agonist, Partial Agonist, Inverse Agonist	4	Anemia; Atherosclerosis; Coronary Artery Disease; Diabetes Mellitus; Diabetes Mellitus, Type 1; Diabetes Mellitus, Type 2; Heart Diseases; Heart Failure; Hypertension; Hypotension; Pulmonary Disease, Chronic Obstructive; Respiratory Distress Syndrome, Adult; Stroke	Arrhythmia; Arrhythmias; Asthma; Bradycardia; Coagulation disorder; Hypoxia; QT interval prolongation; Thrombosis; anaemia; arrhythmias; atrioventricular block; bradycardia; cardiac arrest; chest discomfort; chest pain; circulatory collapse; coma; dyspnoea; embolism and thrombosis; haemorrhage; heart failure; hypertension; hypokalaemia; hyponatraemia; hypotension; hypoxia; myocardial infarction; myocardial ischaemia; myopathy; oedema; pain; palpitations; pulmonary oedema; respiratory disorder; respiratory disorders; syncope; thrombosis; ventricular dysfunction
TIE2 (Q02763)	ANGP2 (O15123)	1	Trebananib, Nesvacumab, Vanucizumab, Amg-780, Medi-3617	Bio_Mol	Inhibitor	3		

General:
Proteins were prioritized by selecting proteins who were associated with at least three CMR traits, and without pathological discordant effects. Data were extracted from Reactome, ChEMBL, and Human Protein Atlas. The nearest druggable protein was identified by linking with Reactome and extracting the nearest proteins that met our definition of druggable (see main text). Distance reflect how many protein interactions (represented by edges in a graph, with one an adjacent protein) these a druggable protein was from the index protein.

Table S15: Directionally concordant CMR proteins: MR effect estimates of plasma protein effects on sixteen CMR traits.

Protein (uniprot)	Gene (ensembl)	CMR trait	No. hits/Path. concordance	MD (95%CI)	P-value	Multiple testing threshold	Q p-value	No. variants	MR model	pQTL source
TDGF1 (P13385)	TDGF1 (ENSG00000241186)	RV – SV (ml)	8.0/8.0	0.24 (0.18; 0.30)	1.8×10^{-15}	7.81×10^{-6}	<0.001	56	IVW	Interval
		RV – PFR (ml/s)		1.41 (0.94; 1.89)	4.1×10^{-9}		0.002	58	MR Egger	
		RV – PER (ml/s)		1.34 (0.93; 1.76)	3.0×10^{-10}		<0.001	55	IVW	
		RV – PAFR (ml/s)		0.61 (0.12; 1.11)	1.6×10^{-2}		<0.001	48	IVW	
		RV – ESV (ml)		-0.01 (-0.08; 0.06)	7.7×10^{-1}		0.019	46	IVW	
		RV – EF (%)		0.08 (0.05; 0.10)	4.6×10^{-10}		0.011	51	IVW	
		RV – EDV (ml)		0.20 (0.10; 0.30)	5.7×10^{-5}		<0.001	54	IVW	
		LV – SV (ml)		0.33 (0.24; 0.43)	8.6×10^{-12}		<0.001	57	MR Egger	
		LV – PFR (ml/s)		0.61 (0.25; 0.98)	9.4×10^{-4}		0.030	56	IVW	
		LV – PER (ml/s)		1.22 (0.77; 1.68)	1.6×10^{-7}		<0.001	60	MR Egger	
		LV – PAFR (ml/s)		0.76 (0.26; 1.26)	2.7×10^{-3}		<0.001	58	MR Egger	
		LV – MVR (g/ml)		0.00 (0.00; 0.00)	1.6×10^{-3}		0.016	50	IVW	
		LV – ESV (ml)		-0.18 (-0.25; -0.12)	5.4×10^{-8}		0.010	47	IVW	
		LV – EF (%)		0.08 (0.05; 0.10)	8.0×10^{-9}		<0.001	48	IVW	
		LV – EDV (ml)		-0.17 (-0.27; -0.07)	9.3×10^{-4}		0.003	50	IVW	
		LV – EDM (g)		-0.11 (-0.16; -0.05)	2.2×10^{-4}		0.013	49	IVW	
OSMR (Q99650)	OSMR (ENSG00000145623)	RV – SV (ml)	7.0/7.0	-1.19 (-1.64; -0.74)	2.6×10^{-7}	7.81×10^{-6}	0.986	9	IVW	Interval
		RV – PFR (ml/s)		-7.23 (-9.85; -4.61)	6.5×10^{-8}		0.318	9	IVW	
		RV – PER (ml/s)		-1.34 (-3.88; 1.19)	3.0×10^{-1}		0.531	9	IVW	
		RV – PAFR (ml/s)		-5.07 (-8.03; -2.10)	8.2×10^{-4}		0.384	9	IVW	
		RV – ESV (ml)		0.57 (0.10; 1.03)	1.7×10^{-2}		0.551	9	IVW	
		RV – EF (%)		-0.52 (-0.70; -0.34)	6.9×10^{-9}		0.793	9	IVW	
		RV – EDV (ml)		-0.55 (-1.30; 0.20)	1.5×10^{-1}		0.840	9	IVW	
		LV – SV (ml)		-1.37 (-1.84; -0.91)	6.7×10^{-9}		0.030	9	IVW	
		LV – PFR (ml/s)		-9.70 (-13.41; -5.98)	3.1×10^{-7}		0.074	9	IVW	
		LV – PER (ml/s)		-10.50 (-13.23; -7.77)	4.6×10^{-14}		0.482	9	IVW	
		LV – PAFR (ml/s)		3.35 (0.31; 6.38)	3.1×10^{-2}		0.612	9	IVW	
		LV – MVR (g/ml)		-0.00 (-0.00; 0.00)	9.1×10^{-1}		0.807	9	IVW	
		LV – ESV (ml)		0.46 (0.03; 0.90)	3.7×10^{-2}		0.740	9	IVW	
		LV – EF (%)		-0.47 (-0.67; -0.28)	2.2×10^{-6}		0.292	9	IVW	
		LV – EDV (ml)		-0.85 (-1.69; 0.00)	5.1×10^{-2}		0.229	9	IVW	
		LV – EDM (g)		-0.41 (-0.81; -0.00)	4.7×10^{-2}		0.695	9	IVW	
BAG3 (O95817)	BAG3 (ENSG00000151929)	RV – SV (ml)	6.0/6.0	-0.52 (-5.25; 4.22)	8.3×10^{-1}	7.81×10^{-6}	0.162	5	MR Egger	Interval
		RV – PFR (ml/s)		13.85 (9.22; 18.48)	4.4×10^{-9}		0.100	5	IVW	
		RV – PER (ml/s)		4.14 (0.46; 7.82)	2.7×10^{-2}		0.337	5	IVW	
		RV – PAFR (ml/s)		-0.96 (-4.88; 2.95)	6.3×10^{-1}		0.674	5	IVW	
		RV – ESV (ml)		-0.68 (-4.85; 3.48)	7.5×10^{-1}		0.286	5	MR Egger	
		RV – EF (%)		2.96 (2.67; 3.26)	1.0×10^{-100}		0.003	4	IVW	
		RV – EDV (ml)		-0.93 (-6.93; 5.08)	7.6×10^{-1}		0.777	5	MR Egger	
		LV – SV (ml)		-0.50 (-1.34; 0.35)	2.5×10^{-1}		0.132	5	IVW	
		LV – PFR (ml/s)		-5.41 (-27.60; 16.78)	6.3×10^{-1}		0.015	5	MR Egger	
		LV – PER (ml/s)		-5.56 (-9.71; -1.41)	8.7×10^{-3}		0.290	5	IVW	
		LV – PAFR (ml/s)		-9.28 (-13.42; -5.14)	1.1×10^{-5}		0.726	5	IVW	
		LV – MVR (g/ml)		0.00 (-0.02; 0.02)	7.7×10^{-1}		0.773	5	MR Egger	
		LV – ESV (ml)		-7.94 (-9.04; -6.84)	1.0×10^{-100}		0.075	4	IVW	
		LV – EF (%)		3.11 (2.81; 3.40)	1.0×10^{-100}		<0.001	4	IVW	
		LV – EDV (ml)		-7.80 (-8.81; -6.80)	1.0×10^{-100}		0.689	5	IVW	
		LV – EDM (g)		-2.11 (-2.67; -1.56)	5.5×10^{-14}		0.772	5	IVW	
MANBA (O00462)	MANBA (ENSG00000109323)	RV – SV (ml)	5.0/5.0	-0.23 (-0.59; 0.13)	2.1×10^{-1}	7.81×10^{-6}	0.364	26	MR Egger	Interval
		RV – PFR (ml/s)		4.23 (2.25; 6.22)	2.8×10^{-5}		0.004	25	MR Egger	
		RV – PER (ml/s)		-0.08 (-1.99; 1.82)	9.3×10^{-1}		0.926	27	MR Egger	
		RV – PAFR (ml/s)		-1.05 (-2.63; 0.52)	1.9×10^{-1}		0.059	20	IVW	
		RV – ESV (ml)		-1.06 (-1.41; -0.71)	2.7×10^{-9}		0.008	28	MR Egger	
		RV – EF (%)		0.43 (0.28; 0.57)	5.8×10^{-9}		0.005	25	MR Egger	
		RV – EDV (ml)		-1.30 (-1.91; -0.68)	3.4×10^{-5}		0.252	27	MR Egger	
		LV – SV (ml)		0.13 (-0.22; 0.48)	4.8×10^{-1}		0.967	27	MR Egger	
		LV – PFR (ml/s)		2.33 (1.56; 3.11)	3.5×10^{-9}		0.357	26	IVW	
		LV – PER (ml/s)		0.91 (-0.19; 2.01)	1.0×10^{-1}		0.267	23	IVW	
		LV – PAFR (ml/s)		-3.02 (-5.31; -0.73)	9.7×10^{-3}		0.997	26	MR Egger	
		LV – MVR (g/ml)		0.00 (0.00; 0.01)	1.2×10^{-5}		0.003	27	MR Egger	
		LV – ESV (ml)		-0.47 (-0.63; -0.31)	1.3×10^{-8}		0.033	24	IVW	
		LV – EF (%)		0.23 (0.16; 0.31)	6.5×10^{-10}		0.193	23	IVW	

Table S15: Directionally concordant CMR proteins: MR effect estimates of plasma protein effects on sixteen CMR traits. *(continued)*

Protein (uniprot)	Gene (ensembl)	CMR trait	No. hits/Path. concordance	MD (95%CI)	P-value	Multiple testing threshold	Q p-value	No. variants	MR model	pQTL source
		LV – EDV (ml)		0.07 (-0.49; 0.62)	8.1×10^{-1}		0.858	28	MR Egger	
		LV – EDM (g)		0.54 (0.22; 0.86)	1.0×10^{-3}		0.297	28	MR Egger	
UD16 (P19224)	UGT1A6 (ENSG00000167165)	RV – SV (ml)	4.0/4.0	1.18 (0.46; 1.90)	1.4×10^{-3}	7.81×10^{-6}	0.047	19	MR Egger	Interval
		RV – PFR (ml/s)		-0.31 (-1.93; 1.31)	7.1×10^{-1}		0.218	17	IVW	
		RV – PER (ml/s)		5.31 (0.63; 10.00)	2.6×10^{-2}		0.148	19	MR Egger	
		RV – PAFR (ml/s)		-1.02 (-3.15; 1.11)	3.5×10^{-1}		0.133	16	IVW	
		RV – ESV (ml)		0.13 (-0.15; 0.40)	3.7×10^{-1}		<0.001	17	IVW	
		RV – EF (%)		0.03 (-0.11; 0.16)	7.0×10^{-1}		0.128	15	IVW	
		RV – EDV (ml)		0.05 (-0.40; 0.50)	8.2×10^{-1}		0.005	17	IVW	
		LV – SV (ml)		-1.42 (-1.71; -1.12)	1.0×10^{-100}		0.012	15	IVW	
		LV – PFR (ml/s)		-5.10 (-7.00; -3.20)	1.4×10^{-7}		0.178	17	IVW	
		LV – PER (ml/s)		-4.64 (-6.32; -2.96)	6.0×10^{-8}		0.617	16	IVW	
		LV – PAFR (ml/s)		2.21 (0.41; 4.02)	1.6×10^{-2}		0.004	17	IVW	
		LV – MVR (g/ml)		0.00 (0.00; 0.00)	7.6×10^{-5}		0.038	15	IVW	
		LV – ESV (ml)		0.10 (-0.16; 0.36)	4.4×10^{-1}		0.945	17	IVW	
		LV – EF (%)		-0.35 (-0.47; -0.23)	1.0×10^{-8}		0.212	17	IVW	
		LV – EDV (ml)		-1.25 (-1.82; -0.69)	1.3×10^{-5}		0.098	16	IVW	
		LV – EDM (g)		-0.20 (-0.45; 0.04)	1.1×10^{-1}		0.022	16	IVW	
TREM1 (Q9NP99)	TREM1 (ENSG00000124731)	RV – SV (ml)	4.0/4.0	-0.11 (-0.24; 0.02)	9.2×10^{-2}	7.81×10^{-6}	0.249	31	IVW	Interval
		RV – PFR (ml/s)		-1.56 (-2.62; -0.51)	3.7×10^{-3}		0.607	32	MR Egger	
		RV – PER (ml/s)		-2.32 (-3.60; -1.05)	3.6×10^{-4}		0.091	33	MR Egger	
		RV – PAFR (ml/s)		-2.39 (-3.30; -1.48)	2.4×10^{-7}		0.101	30	IVW	
		RV – ESV (ml)		0.83 (0.57; 1.09)	3.5×10^{-10}		0.087	32	MR Egger	
		RV – EF (%)		-0.30 (-0.38; -0.23)	4.2×10^{-14}		0.360	32	MR Egger	
		RV – EDV (ml)		-0.22 (-0.45; 0.01)	5.5×10^{-2}		0.124	32	IVW	
		LV – SV (ml)		-0.32 (-0.53; -0.12)	1.6×10^{-3}		0.046	34	MR Egger	
		LV – PFR (ml/s)		0.03 (-0.82; 0.89)	9.4×10^{-1}		0.052	32	IVW	
		LV – PER (ml/s)		-0.90 (-1.71; -0.09)	3.0×10^{-2}		0.138	32	IVW	
		LV – PAFR (ml/s)		-2.13 (-3.59; -0.66)	4.4×10^{-3}		0.224	31	MR Egger	
		LV – MVR (g/ml)		-0.00 (-0.00; 0.00)	9.7×10^{-1}		0.577	34	MR Egger	
		LV – ESV (ml)		0.07 (-0.05; 0.19)	2.5×10^{-1}		0.243	32	IVW	
		LV – EF (%)		-0.33 (-0.41; -0.26)	1.0×10^{-100}		0.901	34	MR Egger	
		LV – EDV (ml)		-0.00 (-0.23; 0.22)	9.8×10^{-1}		0.075	31	IVW	
		LV – EDM (g)		-0.21 (-0.32; -0.10)	2.9×10^{-4}		0.003	30	IVW	
PRDX1 (Q06830)	PRDX1 (ENSG00000117450)	RV – SV (ml)	4.0/4.0	-0.09 (-1.06; 0.89)	8.6×10^{-1}	7.81×10^{-6}	0.661	3	IVW	Interval
		RV – PFR (ml/s)		-5.15 (-10.38; 0.08)	5.3×10^{-2}		0.741	3	IVW	
		RV – PER (ml/s)		4.94 (-2.74; 12.63)	2.1×10^{-1}		0.136	3	IVW	
		RV – PAFR (ml/s)		-3.93 (-10.11; 2.25)	2.1×10^{-1}		0.784	3	IVW	
		RV – ESV (ml)		2.60 (1.60; 3.60)	3.2×10^{-7}		0.035	3	IVW	
		RV – EF (%)		-1.02 (-1.44; -0.61)	1.4×10^{-6}		0.300	3	IVW	
		RV – EDV (ml)		2.25 (0.01; 4.50)	4.9×10^{-2}		0.145	3	IVW	
		LV – SV (ml)		-0.08 (-1.08; 0.92)	8.8×10^{-1}		0.396	3	IVW	
		LV – PFR (ml/s)		2.71 (-3.27; 8.70)	3.7×10^{-1}		0.496	3	IVW	
		LV – PER (ml/s)		4.32 (-2.12; 10.77)	1.9×10^{-1}		0.300	3	IVW	
		LV – PAFR (ml/s)		-0.98 (-7.50; 5.55)	7.7×10^{-1}		0.741	3	IVW	
		LV – MVR (g/ml)		-0.01 (-0.01; 0.00)	1.1×10^{-1}		0.199	3	IVW	
		LV – ESV (ml)		2.13 (1.20; 3.06)	7.4×10^{-6}		0.033	3	IVW	
		LV – EF (%)		-0.99 (-1.38; -0.59)	9.9×10^{-7}		0.345	3	IVW	
		LV – EDV (ml)		2.04 (-0.61; 4.69)	1.3×10^{-1}		0.062	3	IVW	
		LV – EDM (g)		0.46 (-0.41; 1.32)	3.0×10^{-1}		0.447	3	IVW	
PGLT1 (Q8NBL1)	POGLUT1 (ENSG00000163389)	RV – SV (ml)	4.0/4.0	0.08 (-1.24; 1.40)	9.0×10^{-1}	7.81×10^{-6}	0.119	11	MR Egger	Interval
		RV – PFR (ml/s)		3.32 (0.83; 5.81)	9.0×10^{-3}		<0.001	11	IVW	
		RV – PER (ml/s)		-4.01 (-11.19; 3.16)	2.7×10^{-1}		0.147	11	MR Egger	
		RV – PAFR (ml/s)		1.36 (-1.95; 4.67)	4.2×10^{-1}		<0.001	10	IVW	
		RV – ESV (ml)		-1.89 (-2.37; -1.41)	8.4×10^{-15}		0.434	11	IVW	
		RV – EF (%)		0.40 (0.22; 0.58)	1.2×10^{-5}		0.600	11	IVW	
		RV – EDV (ml)		-2.96 (-3.77; -2.14)	9.0×10^{-13}		0.596	10	IVW	
		LV – SV (ml)		-0.55 (-1.67; 0.57)	3.4×10^{-1}		0.375	11	MR Egger	
		LV – PFR (ml/s)		-0.01 (-6.48; 6.46)	1.0×10^{0}		0.582	11	MR Egger	
		LV – PER (ml/s)		-9.92 (-18.30; -1.53)	2.0×10^{-2}		0.073	11	MR Egger	
		LV – PAFR (ml/s)		0.19 (-2.92; 3.30)	9.0×10^{-1}		0.848	11	IVW	
		LV – MVR (g/ml)		-0.01 (-0.02; -0.00)	4.0×10^{-3}		0.056	11	MR Egger	

Table S15: Directionally concordant CMR proteins: MR effect estimates of plasma protein effects on sixteen CMR traits. *(continued)*

Protein (uniprot)	Gene (ensembl)	CMR trait	No. hits/Path. concordance	MD (95%CI)	P-value	Multiple testing threshold	Q p-value	No. variants	MR model	pQTL source
		LV – ESV (ml)		-1.25 (-1.85; -0.65)	4.3×10^{-5}		0.052	11	IVW	
		LV – EF (%)		0.31 (0.12; 0.49)	1.0×10^{-3}		0.570	11	IVW	
		LV – EDV (ml)		-2.32 (-3.12; -1.52)	1.1×10^{-8}		0.626	10	IVW	
		LV – EDM (g)		-1.34 (-1.82; -0.86)	3.5×10^{-8}		0.748	8	IVW	
NET1 (O95631)	NTN1 (ENSG00000065320)	RV – SV (ml)	4.0/4.0	-0.93 (-1.47; -0.39)	6.7×10^{-4}	7.81×10^{-6}	0.005	28	MR Egger	Interval
		RV – PFR (ml/s)		1.01 (-0.23; 2.24)	1.1×10^{-1}		<0.001	28	IVW	
		RV – PER (ml/s)		-2.56 (-3.62; -1.51)	1.9×10^{-6}		0.216	31	IVW	
		RV – PAFR (ml/s)		-3.29 (-4.67; -1.92)	2.6×10^{-6}		0.074	31	IVW	
		RV – ESV (ml)		-0.30 (-0.51; -0.09)	4.8×10^{-3}		0.095	31	IVW	
		RV – EF (%)		-0.08 (-0.15; -0.00)	3.8×10^{-2}		0.006	28	IVW	
		RV – EDV (ml)		-1.60 (-2.39; -0.81)	7.9×10^{-5}		<0.001	31	MR Egger	
		LV – SV (ml)		-0.68 (-1.16; -0.21)	5.0×10^{-3}		0.020	30	MR Egger	
		LV – PFR (ml/s)		-2.83 (-3.98; -1.68)	1.5×10^{-6}		<0.001	29	IVW	
		LV – PER (ml/s)		-3.18 (-4.29; -2.06)	2.3×10^{-8}		0.029	30	IVW	
		LV – PAFR (ml/s)		-2.14 (-3.36; -0.92)	5.9×10^{-4}		0.019	30	IVW	
		LV – MVR (g/ml)		0.00 (0.00; 0.00)	3.0×10^{-5}		0.157	31	IVW	
		LV – ESV (ml)		-0.11 (-0.31; 0.09)	2.8×10^{-1}		0.003	29	IVW	
		LV – EF (%)		-0.07 (-0.14; 0.00)	5.9×10^{-2}		0.045	30	IVW	
		LV – EDV (ml)		-0.68 (-1.04; -0.32)	2.5×10^{-4}		0.066	29	IVW	
		LV – EDM (g)		-0.14 (-0.33; 0.05)	1.5×10^{-1}		0.012	28	IVW	
IL18R (Q13478)	IL18R1 (ENSG00000115604)	RV – SV (ml)	4.0/4.0	-0.33 (-0.54; -0.12)	1.7×10^{-3}	7.81×10^{-6}	0.131	46	MR Egger	Interval
		RV – PFR (ml/s)		-2.28 (-3.29; -1.27)	1.0×10^{-5}		<0.001	45	MR Egger	
		RV – PER (ml/s)		-1.32 (-2.60; -0.03)	4.4×10^{-2}		0.213	44	MR Egger	
		RV – PAFR (ml/s)		1.10 (-0.09; 2.29)	7.0×10^{-2}		0.006	45	MR Egger	
		RV – ESV (ml)		-0.05 (-0.16; 0.06)	3.8×10^{-1}		<0.001	33	IVW	
		RV – EF (%)		-0.01 (-0.09; 0.08)	8.5×10^{-1}		0.052	45	MR Egger	
		RV – EDV (ml)		-0.65 (-0.96; -0.34)	3.6×10^{-5}		0.008	46	MR Egger	
		LV – SV (ml)		-0.16 (-0.38; 0.06)	1.5×10^{-1}		0.088	46	MR Egger	
		LV – PFR (ml/s)		-1.81 (-2.98; -0.64)	2.3×10^{-3}		<0.001	44	MR Egger	
		LV – PER (ml/s)		2.50 (1.20; 3.80)	1.6×10^{-4}		0.076	45	MR Egger	
		LV – PAFR (ml/s)		-2.48 (-3.77; -1.20)	1.6×10^{-4}		<0.001	43	MR Egger	
		LV – MVR (g/ml)		0.00 (0.00; 0.00)	4.3×10^{-4}		0.005	45	MR Egger	
		LV – ESV (ml)		-0.26 (-0.35; -0.17)	5.0×10^{-8}		0.091	32	IVW	
		LV – EF (%)		0.10 (0.07; 0.14)	3.0×10^{-8}		<0.001	35	IVW	
		LV – EDV (ml)		-0.50 (-0.62; -0.39)	1.0×10^{-100}		<0.001	39	IVW	
		LV – EDM (g)		-0.18 (-0.25; -0.11)	2.8×10^{-7}		<0.001	40	IVW	
GPC5 (P78333)	GPC5 (ENSG00000179399)	RV – SV (ml)	4.0/4.0	-0.42 (-0.54; -0.30)	1.6×10^{-11}	7.81×10^{-6}	0.003	78	IVW	Interval
		RV – PFR (ml/s)		-3.77 (-4.36; -3.18)	1.0×10^{-100}		<0.001	77	IVW	
		RV – PER (ml/s)		2.00 (0.41; 3.59)	1.4×10^{-2}		0.002	83	MR Egger	
		RV – PAFR (ml/s)		0.25 (-0.41; 0.92)	4.6×10^{-1}		<0.001	83	IVW	
		RV – ESV (ml)		0.72 (0.44; 1.00)	4.5×10^{-7}		<0.001	83	MR Egger	
		RV – EF (%)		-0.26 (-0.36; -0.16)	6.1×10^{-7}		0.026	88	MR Egger	
		RV – EDV (ml)		-0.43 (-0.63; -0.23)	3.3×10^{-5}		<0.001	74	IVW	
		LV – SV (ml)		-0.11 (-0.23; 0.01)	7.6×10^{-2}		<0.001	77	IVW	
		LV – PFR (ml/s)		3.00 (1.10; 4.90)	2.0×10^{-3}		<0.001	78	MR Egger	
		LV – PER (ml/s)		-1.28 (-1.93; -0.64)	9.8×10^{-5}		0.004	83	IVW	
		LV – PAFR (ml/s)		-0.79 (-1.53; -0.06)	3.5×10^{-2}		<0.001	82	IVW	
		LV – MVR (g/ml)		0.00 (-0.00; 0.00)	1.7×10^{-1}		<0.001	84	MR Egger	
		LV – ESV (ml)		-0.05 (-0.15; 0.05)	3.5×10^{-1}		<0.001	83	IVW	
		LV – EF (%)		0.00 (-0.11; 0.12)	9.4×10^{-1}		0.138	89	MR Egger	
		LV – EDV (ml)		0.92 (0.38; 1.46)	8.0×10^{-4}		<0.001	75	MR Egger	
		LV – EDM (g)		0.54 (0.28; 0.80)	6.1×10^{-5}		<0.001	83	MR Egger	
ERAP2 (Q6P179)	ERAP2 (ENSG00000164308)	RV – SV (ml)	4.0/4.0	-0.17 (-0.35; 0.01)	6.7×10^{-2}	7.81×10^{-6}	0.002	45	MR Egger	Interval
		RV – PFR (ml/s)		-1.35 (-2.41; -0.28)	1.3×10^{-2}		<0.001	42	MR Egger	
		RV – PER (ml/s)		-0.23 (-0.46; 0.01)	6.1×10^{-2}		<0.001	57	IVW	
		RV – PAFR (ml/s)		0.13 (-0.65; 0.90)	7.5×10^{-1}		<0.001	55	MR Egger	
		RV – ESV (ml)		-0.34 (-0.46; -0.23)	1.5×10^{-9}		0.039	54	MR Egger	
		RV – EF (%)		0.10 (0.05; 0.15)	8.1×10^{-5}		<0.001	54	MR Egger	
		RV – EDV (ml)		-0.35 (-0.58; -0.12)	2.7×10^{-3}		<0.001	47	MR Egger	
		LV – SV (ml)		-0.01 (-0.14; 0.12)	8.6×10^{-1}		0.084	54	MR Egger	
		LV – PFR (ml/s)		1.90 (1.66; 2.14)	1.0×10^{-100}		<0.001	54	IVW	
		LV – PER (ml/s)		-0.10 (-0.37; 0.17)	4.7×10^{-1}		<0.001	53	IVW	

Table S15: Directionally concordant CMR proteins: MR effect estimates of plasma protein effects on sixteen CMR traits. *(continued)*

Protein (uniprot)	Gene (ensembl)	CMR trait	No. hits/Path. concordance	MD (95%CI)	P-value	Multiple testing threshold	Q p-value	No. variants	MR model	pQTL source
		LV – PAFR (ml/s)		-0.97 (-1.88; -0.05)	3.9×10^{-2}		0.198	54	MR Egger	
		LV – MVR (g/ml)		0.00 (-0.00; 0.00)	7.0×10^{-1}		0.031	56	MR Egger	
		LV – ESV (ml)		-0.00 (-0.12; 0.11)	9.4×10^{-1}		0.104	57	MR Egger	
		LV – EF (%)		-0.00 (-0.02; 0.02)	9.3×10^{-1}		0.650	54	IVW	
		LV – EDV (ml)		-0.27 (-0.33; -0.20)	4.4×10^{-16}		0.003	56	IVW	
		LV – EDM (g)		-0.22 (-0.31; -0.12)	6.7×10^{-6}		<0.001	57	MR Egger	
ENTP1 (P49961)	ENTPD1 (ENSG00000138185)	RV – SV (ml)	4.0/4.0	-0.89 (-3.25; 1.48)	4.6×10^{-1}	7.81×10^{-6}	0.471	9	MR Egger	Interval
		RV – PFR (ml/s)		-8.04 (-9.21; -6.88)	1.0×10^{-100}		0.980	11	IVW	
		RV – PER (ml/s)		-1.55 (-14.43; 11.33)	8.1×10^{-1}		0.857	11	MR Egger	
		RV – PAFR (ml/s)		-2.64 (-4.02; -1.26)	1.7×10^{-4}		0.903	11	IVW	
		RV – ESV (ml)		-1.15 (-3.92; 1.62)	4.2×10^{-1}		0.583	8	MR Egger	
		RV – EF (%)		-0.41 (-0.50; -0.33)	1.0×10^{-100}		0.576	11	IVW	
		RV – EDV (ml)		-1.21 (-5.71; 3.28)	6.0×10^{-1}		0.612	8	MR Egger	
		LV – SV (ml)		0.03 (-2.39; 2.45)	9.8×10^{-1}		0.877	9	MR Egger	
		LV – PFR (ml/s)		-6.76 (-8.09; -5.43)	1.0×10^{-100}		0.756	11	IVW	
		LV – PER (ml/s)		-6.70 (-8.01; -5.39)	1.0×10^{-100}		0.844	11	IVW	
		LV – PAFR (ml/s)		-18.41 (-34.22; -2.60)	2.3×10^{-2}		0.978	9	MR Egger	
		LV – MVR (g/ml)		-0.00 (-0.00; 0.00)	1.3×10^{-1}		0.918	11	IVW	
		LV – ESV (ml)		-3.77 (-7.65; 0.10)	5.6×10^{-2}		0.032	4	MR Egger	
		LV – EF (%)		1.11 (0.14; 2.08)	2.5×10^{-2}		0.361	9	MR Egger	
		LV – EDV (ml)		-1.27 (-7.19; 4.65)	6.7×10^{-1}		0.149	6	MR Egger	
		LV – EDM (g)		0.04 (-2.06; 2.15)	9.7×10^{-1}		0.618	9	MR Egger	
BGH3 (Q15582)	TGFBI (ENSG00000120708)	RV – SV (ml)	4.0/4.0	1.20 (0.27; 2.13)	1.2×10^{-2}	7.81×10^{-6}	0.831	20	MR Egger	Interval
		RV – PFR (ml/s)		0.67 (-0.19; 1.53)	1.3×10^{-1}		0.793	19	IVW	
		RV – PER (ml/s)		-0.31 (-1.20; 0.59)	5.1×10^{-1}		0.898	19	IVW	
		RV – PAFR (ml/s)		5.45 (-0.53; 11.43)	7.4×10^{-2}		0.420	20	MR Egger	
		RV – ESV (ml)		0.79 (0.62; 0.95)	1.0×10^{-100}		0.589	19	IVW	
		RV – EF (%)		-0.13 (-0.20; -0.07)	3.3×10^{-5}		0.727	19	IVW	
		RV – EDV (ml)		1.23 (0.97; 1.50)	1.0×10^{-100}		0.646	19	IVW	
		LV – SV (ml)		0.33 (0.17; 0.50)	7.0×10^{-5}		0.472	19	IVW	
		LV – PFR (ml/s)		-0.39 (-1.61; 0.83)	5.3×10^{-1}		0.198	18	IVW	
		LV – PER (ml/s)		0.57 (-0.51; 1.64)	3.0×10^{-1}		0.422	17	IVW	
		LV – PAFR (ml/s)		0.83 (-5.39; 7.04)	7.9×10^{-1}		0.557	20	MR Egger	
		LV – MVR (g/ml)		-0.00 (-0.00; 0.00)	5.1×10^{-1}		0.597	19	IVW	
		LV – ESV (ml)		0.70 (0.55; 0.85)	1.0×10^{-100}		0.009	19	IVW	
		LV – EF (%)		-0.01 (-0.40; 0.38)	9.7×10^{-1}		0.303	20	MR Egger	
		LV – EDV (ml)		1.02 (0.71; 1.32)	5.1×10^{-11}		0.152	19	IVW	
		LV – EDM (g)		0.63 (-0.26; 1.53)	1.7×10^{-1}		0.270	20	MR Egger	
ASAH2 (Q9NR71)	ASAH2 (ENSG00000188611)	RV – SV (ml)	4.0/4.0	0.63 (0.30; 0.95)	1.6×10^{-4}	7.81×10^{-6}	0.092	23	MR Egger	Interval
		RV – PFR (ml/s)		2.19 (-0.19; 4.57)	7.2×10^{-2}		0.228	20	MR Egger	
		RV – PER (ml/s)		-1.66 (-2.70; -0.62)	1.8×10^{-3}		<0.001	23	IVW	
		RV – PAFR (ml/s)		-2.40 (-4.44; -0.36)	2.1×10^{-2}		0.034	22	MR Egger	
		RV – ESV (ml)		0.81 (0.50; 1.13)	3.6×10^{-7}		0.216	21	MR Egger	
		RV – EF (%)		-0.14 (-0.21; -0.08)	6.3×10^{-6}		0.140	24	IVW	
		RV – EDV (ml)		1.42 (0.91; 1.93)	4.6×10^{-8}		0.178	23	MR Egger	
		LV – SV (ml)		-0.38 (-0.66; -0.10)	8.2×10^{-3}		<0.001	24	MR Egger	
		LV – PFR (ml/s)		-1.03 (-2.67; 0.61)	2.2×10^{-1}		0.652	25	MR Egger	
		LV – PER (ml/s)		1.98 (0.35; 3.61)	1.7×10^{-2}		0.023	24	MR Egger	
		LV – PAFR (ml/s)		-4.91 (-7.81; -2.01)	9.0×10^{-4}		0.510	16	MR Egger	
		LV – MVR (g/ml)		0.00 (0.00; 0.00)	1.1×10^{-2}		0.008	23	IVW	
		LV – ESV (ml)		0.26 (0.15; 0.38)	4.0×10^{-6}		<0.001	25	IVW	
		LV – EF (%)		-0.08 (-0.14; -0.03)	3.5×10^{-3}		0.055	26	IVW	
		LV – EDV (ml)		-0.25 (-0.70; 0.20)	2.7×10^{-1}		<0.001	24	MR Egger	
		LV – EDM (g)		-0.39 (-0.68; -0.10)	8.1×10^{-3}		0.098	24	MR Egger	
TPSNR (Q9BX59)	TAPBPL (ENSG00000139192)	RV – SV (ml)	3.0/3.0	-0.17 (-0.23; -0.11)	1.2×10^{-7}	7.81×10^{-6}	0.016	60	IVW	Interval
		RV – PFR (ml/s)		-0.53 (-0.87; -0.20)	1.5×10^{-3}		0.035	59	IVW	
		RV – PER (ml/s)		-0.81 (-1.21; -0.42)	5.2×10^{-5}		0.038	53	IVW	
		RV – PAFR (ml/s)		1.29 (0.22; 2.35)	1.8×10^{-2}		0.037	53	MR Egger	
		RV – ESV (ml)		-0.13 (-0.30; 0.04)	1.4×10^{-1}		0.185	55	MR Egger	
		RV – EF (%)		0.07 (0.02; 0.12)	8.3×10^{-3}		0.015	59	MR Egger	
		RV – EDV (ml)		-0.35 (-0.62; -0.08)	1.2×10^{-2}		0.014	55	MR Egger	
		LV – SV (ml)		-0.16 (-0.22; -0.10)	1.5×10^{-7}		0.002	62	IVW	

Table S15: Directionally concordant CMR proteins: MR effect estimates of plasma protein effects on sixteen CMR traits. *(continued)*

Protein (uniprot)	Gene (ensembl)	CMR trait	No. hits/Path. concordance	MD (95%CI)	P-value	Multiple testing threshold	Q p-value	No. variants	MR model	pQTL source
		LV – PFR (ml/s)		-0.46 (-1.28; 0.37)	2.8×10^{-1}		0.023	57	MR Egger	
		LV – PER (ml/s)		-1.04 (-1.98; -0.11)	2.8×10^{-2}		0.092	57	MR Egger	
		LV – PAFR (ml/s)		-0.00 (-0.49; 0.48)	9.9×10^{-1}		0.022	53	IVW	
		LV – MVR (g/ml)		0.00 (0.00; 0.00)	6.1×10^{-10}		0.011	57	IVW	
		LV – ESV (ml)		-0.12 (-0.18; -0.05)	3.0×10^{-4}		<0.001	58	IVW	
		LV – EF (%)		0.09 (0.04; 0.15)	1.0×10^{-3}		0.019	56	MR Egger	
		LV – EDV (ml)		-0.46 (-0.71; -0.21)	3.3×10^{-4}		0.074	55	MR Egger	
		LV – EDM (g)		-0.06 (-0.12; 0.01)	8.2×10^{-2}		0.451	55	IVW	
TIE2 (Q02763)	TEK (ENSG00000120156)	RV – SV (ml)	3.0/3.0	0.02 (-0.35; 0.39)	9.1×10^{-1}	7.81×10^{-6}	<0.001	52	MR Egger	Scallop
		RV – PFR (ml/s)		-2.80 (-5.15; -0.46)	1.9×10^{-2}		0.018	49	MR Egger	
		RV – PER (ml/s)		0.22 (-2.02; 2.46)	8.5×10^{-1}		<0.001	47	MR Egger	
		RV – PAFR (ml/s)		5.47 (4.15; 6.79)	4.4×10^{-16}		<0.001	54	IVW	
		RV – ESV (ml)		-0.68 (-0.89; -0.48)	4.1×10^{-11}		<0.001	48	IVW	
		RV – EF (%)		-0.03 (-0.15; 0.10)	6.8×10^{-1}		0.015	53	MR Egger	
		RV – EDV (ml)		-0.02 (-0.56; 0.52)	9.4×10^{-1}		<0.001	55	MR Egger	
		LV – SV (ml)		0.23 (-0.16; 0.62)	2.4×10^{-1}		<0.001	48	MR Egger	
		LV – PFR (ml/s)		-0.48 (-2.45; 1.49)	6.3×10^{-1}		<0.001	52	MR Egger	
		LV – PER (ml/s)		-0.12 (-2.54; 2.30)	9.2×10^{-1}		<0.001	48	MR Egger	
		LV – PAFR (ml/s)		1.81 (0.57; 3.05)	4.2×10^{-3}		<0.001	55	IVW	
		LV – MVR (g/ml)		-0.00 (-0.00; -0.00)	4.6×10^{-2}		0.011	52	MR Egger	
		LV – ESV (ml)		-0.34 (-0.74; 0.06)	9.4×10^{-2}		<0.001	44	MR Egger	
		LV – EF (%)		0.43 (0.32; 0.55)	8.9×10^{-13}		<0.001	49	MR Egger	
		LV – EDV (ml)		0.20 (-0.50; 0.90)	5.7×10^{-1}		0.013	50	MR Egger	
		LV – EDM (g)		-0.14 (-0.53; 0.25)	4.7×10^{-1}		<0.001	46	MR Egger	
SPA12 (Q8IW75)	SERPINA12 (ENSG00000165953)	RV – SV (ml)	3.0/3.0	0.55 (-0.58; 1.67)	3.4×10^{-1}	7.81×10^{-6}	0.068	20	MR Egger	Interval
		RV – PFR (ml/s)		2.74 (1.41; 4.08)	5.5×10^{-5}		0.120	19	IVW	
		RV – PER (ml/s)		-1.10 (-2.26; 0.07)	6.5×10^{-2}		<0.001	18	IVW	
		RV – PAFR (ml/s)		0.42 (-5.36; 6.19)	8.9×10^{-1}		0.048	20	MR Egger	
		RV – ESV (ml)		-0.82 (-1.02; -0.62)	1.6×10^{-15}		0.954	20	IVW	
		RV – EF (%)		0.33 (0.25; 0.42)	2.0×10^{-15}		0.270	20	IVW	
		RV – EDV (ml)		0.16 (-1.36; 1.67)	8.4×10^{-1}		0.572	20	MR Egger	
		LV – SV (ml)		-0.24 (-0.48; 0.01)	5.6×10^{-2}		0.092	20	IVW	
		LV – PFR (ml/s)		-1.48 (-2.68; -0.27)	1.6×10^{-2}		<0.001	20	IVW	
		LV – PER (ml/s)		-0.31 (-1.49; 0.87)	6.0×10^{-1}		0.035	20	IVW	
		LV – PAFR (ml/s)		3.36 (1.86; 4.86)	1.2×10^{-5}		0.161	20	IVW	
		LV – MVR (g/ml)		0.00 (-0.00; 0.01)	9.2×10^{-2}		0.596	20	MR Egger	
		LV – ESV (ml)		-0.49 (-1.37; 0.39)	2.7×10^{-1}		0.728	19	MR Egger	
		LV – EF (%)		0.12 (0.03; 0.22)	8.3×10^{-3}		0.110	19	IVW	
		LV – EDV (ml)		-0.76 (-1.07; -0.44)	3.6×10^{-6}		0.712	20	IVW	
		LV – EDM (g)		-0.19 (-0.39; 0.02)	8.2×10^{-2}		0.099	20	IVW	
RMD1 (Q96DB5)	RMDN1 (ENSG00000176623)	RV – SV (ml)	3.0/3.0	-3.53 (-5.13; -1.94)	1.4×10^{-5}	7.81×10^{-6}	0.350	8	MR Egger	Interval
		RV – PFR (ml/s)		-1.94 (-3.60; -0.28)	2.2×10^{-2}		0.962	8	IVW	
		RV – PER (ml/s)		-6.15 (-8.35; -3.96)	3.7×10^{-8}		0.128	8	IVW	
		RV – PAFR (ml/s)		-21.81 (-34.60; -9.02)	8.3×10^{-4}		0.096	8	MR Egger	
		RV – ESV (ml)		-0.25 (-0.57; 0.06)	1.2×10^{-1}		0.932	8	IVW	
		RV – EF (%)		-0.22 (-0.35; -0.09)	7.5×10^{-4}		0.346	8	IVW	
		RV – EDV (ml)		-4.91 (-7.41; -2.40)	1.2×10^{-4}		0.906	8	MR Egger	
		LV – SV (ml)		-2.89 (-4.43; -1.34)	2.5×10^{-4}		0.696	8	MR Egger	
		LV – PFR (ml/s)		-6.96 (-8.86; -5.06)	7.0×10^{-13}		0.575	8	IVW	
		LV – PER (ml/s)		-16.30 (-25.38; -7.22)	4.3×10^{-4}		0.874	8	MR Egger	
		LV – PAFR (ml/s)		-1.57 (-3.64; 0.51)	1.4×10^{-1}		0.685	8	IVW	
		LV – MVR (g/ml)		0.00 (0.00; 0.00)	1.2×10^{-3}		0.783	8	IVW	
		LV – ESV (ml)		0.45 (0.15; 0.74)	3.1×10^{-3}		0.995	8	IVW	
		LV – EF (%)		-0.40 (-0.52; -0.28)	1.3×10^{-10}		0.786	8	IVW	
		LV – EDV (ml)		-2.89 (-5.34; -0.43)	2.1×10^{-2}		0.826	8	MR Egger	
		LV – EDM (g)		0.21 (-0.07; 0.48)	1.4×10^{-1}		0.490	8	IVW	
PPAC (P24666)	ACP1 (ENSG00000143727)	RV – SV (ml)	3.0/3.0	0.19 (0.12; 0.25)	1.7×10^{-7}	7.81×10^{-6}	0.009	51	IVW	Interval
		RV – PFR (ml/s)		0.20 (-0.55; 0.95)	6.1×10^{-1}		0.012	53	MR Egger	
		RV – PER (ml/s)		0.22 (-0.55; 0.99)	5.7×10^{-1}		0.651	55	MR Egger	
		RV – PAFR (ml/s)		1.50 (0.58; 2.41)	1.3×10^{-3}		0.288	56	MR Egger	
		RV – ESV (ml)		0.04 (-0.03; 0.11)	2.9×10^{-1}		<0.001	53	IVW	
		RV – EF (%)		-0.01 (-0.03; 0.02)	6.4×10^{-1}		0.002	51	IVW	

Table S15: Directionally concordant CMR proteins: MR effect estimates of plasma protein effects on sixteen CMR traits. *(continued)*

Protein (uniprot)	Gene (ensembl)	CMR trait	No. hits/Path. concordance	MD (95%CI)	P-value	Multiple testing threshold	Q p-value	No. variants	MR model	pQTL source
		RV – EDV (ml)		-0.06 (-0.29; 0.17)	5.9×10^{-1}		0.010	55	MR Egger	
		LV – SV (ml)		0.05 (-0.12; 0.21)	5.8×10^{-1}		0.086	55	MR Egger	
		LV – PFR (ml/s)		1.22 (0.75; 1.70)	4.1×10^{-7}		0.106	52	IVW	
		LV – PER (ml/s)		1.11 (0.71; 1.52)	7.8×10^{-8}		0.008	53	IVW	
		LV – PAFR (ml/s)		1.01 (0.05; 1.97)	3.8×10^{-2}		<0.001	53	MR Egger	
		LV – MVR (g/ml)		-0.00 (-0.00; 0.00)	9.6×10^{-1}		0.002	50	IVW	
		LV – ESV (ml)		-0.03 (-0.16; 0.10)	6.8×10^{-1}		0.716	56	MR Egger	
		LV – EF (%)		-0.01 (-0.07; 0.04)	6.8×10^{-1}		0.852	56	MR Egger	
		LV – EDV (ml)		0.01 (-0.24; 0.25)	9.6×10^{-1}		0.137	56	MR Egger	
		LV – EDM (g)		0.14 (0.02; 0.26)	2.6×10^{-2}		0.498	56	MR Egger	
PATE4 (P0C8F1)	PATE4 (ENSG00000237353)	RV – SV (ml)	3.0/3.0	-0.07 (-0.20; 0.07)	3.2×10^{-1}	7.81×10^{-6}	0.002	28	IVW	Interval
		RV – PFR (ml/s)		-1.57 (-2.30; -0.84)	2.3×10^{-5}		<0.001	29	IVW	
		RV – PER (ml/s)		-2.06 (-2.67; -1.44)	6.4×10^{-11}		0.029	35	IVW	
		RV – PAFR (ml/s)		0.61 (-0.30; 1.52)	1.9×10^{-1}		0.159	30	IVW	
		RV – ESV (ml)		0.45 (0.29; 0.60)	1.2×10^{-8}		0.006	28	IVW	
		RV – EF (%)		-0.17 (-0.23; -0.11)	2.4×10^{-8}		<0.001	28	IVW	
		RV – EDV (ml)		0.40 (0.21; 0.59)	3.8×10^{-5}		<0.001	30	IVW	
		LV – SV (ml)		0.11 (-0.01; 0.23)	7.0×10^{-2}		0.009	34	IVW	
		LV – PFR (ml/s)		-1.24 (-3.84; 1.36)	3.5×10^{-1}		<0.001	31	MR Egger	
		LV – PER (ml/s)		1.70 (0.92; 2.48)	1.9×10^{-5}		<0.001	31	IVW	
		LV – PAFR (ml/s)		-0.31 (-3.18; 2.57)	8.3×10^{-1}		<0.001	28	MR Egger	
		LV – MVR (g/ml)		-0.00 (-0.00; 0.00)	3.1×10^{-1}		0.169	32	MR Egger	
		LV – ESV (ml)		0.04 (-0.39; 0.48)	8.5×10^{-1}		<0.001	31	MR Egger	
		LV – EF (%)		-0.17 (-0.35; 0.00)	5.6×10^{-2}		0.276	31	MR Egger	
		LV – EDV (ml)		-0.26 (-1.00; 0.49)	5.0×10^{-1}		0.002	31	MR Egger	
		LV – EDM (g)		-0.10 (-0.48; 0.27)	5.9×10^{-1}		0.003	31	MR Egger	
NCAM2 (O15394)	NCAM2 (ENSG00000154654)	RV – SV (ml)	3.0/3.0	-0.03 (-0.24; 0.17)	7.5×10^{-1}	7.81×10^{-6}	0.467	31	IVW	Interval
		RV – PFR (ml/s)		-1.61 (-5.44; 2.23)	4.1×10^{-1}		0.098	31	MR Egger	
		RV – PER (ml/s)		5.24 (1.80; 8.67)	2.8×10^{-3}		0.011	30	MR Egger	
		RV – PAFR (ml/s)		-1.26 (-5.13; 2.61)	5.2×10^{-1}		0.045	32	MR Egger	
		RV – ESV (ml)		0.25 (-0.43; 0.94)	4.7×10^{-1}		0.644	30	MR Egger	
		RV – EF (%)		-0.06 (-0.37; 0.24)	6.8×10^{-1}		0.054	31	MR Egger	
		RV – EDV (ml)		-0.84 (-1.20; -0.49)	3.0×10^{-6}		0.055	33	IVW	
		LV – SV (ml)		0.86 (0.09; 1.63)	2.8×10^{-2}		0.174	30	MR Egger	
		LV – PFR (ml/s)		-2.00 (-6.89; 2.89)	4.2×10^{-1}		0.069	30	MR Egger	
		LV – PER (ml/s)		9.39 (5.46; 13.31)	2.7×10^{-6}		0.277	32	MR Egger	
		LV – PAFR (ml/s)		4.10 (2.54; 5.66)	2.5×10^{-7}		0.024	30	IVW	
		LV – MVR (g/ml)		-0.00 (-0.01; 0.00)	2.2×10^{-1}		<0.001	31	MR Egger	
		LV – ESV (ml)		-0.00 (-0.17; 0.17)	9.8×10^{-1}		0.468	34	IVW	
		LV – EF (%)		0.29 (0.05; 0.53)	1.9×10^{-2}		0.859	32	MR Egger	
		LV – EDV (ml)		0.61 (-0.53; 1.76)	2.9×10^{-1}		0.316	30	MR Egger	
		LV – EDM (g)		-0.25 (-0.88; 0.38)	4.4×10^{-1}		0.402	29	MR Egger	
KAT3 (Q6YP21)	CCBL2 (ENSG00000137944)	RV – SV (ml)	3.0/3.0	-0.69 (-1.52; 0.13)	9.8×10^{-2}	7.81×10^{-6}	0.178	5	IVW	Interval
		RV – PFR (ml/s)		-10.05 (-13.56; -6.54)	2.0×10^{-8}		0.705	5	IVW	
		RV – PER (ml/s)		-25.14 (-66.70; 16.42)	2.4×10^{-1}		0.462	5	MR Egger	
		RV – PAFR (ml/s)		-4.65 (-10.13; 0.83)	9.7×10^{-2}		0.137	5	IVW	
		RV – ESV (ml)		-5.33 (-12.95; 2.29)	1.7×10^{-1}		0.932	5	MR Egger	
		RV – EF (%)		-0.88 (-1.13; -0.63)	1.2×10^{-11}		0.573	5	IVW	
		RV – EDV (ml)		-8.07 (-20.43; 4.28)	2.0×10^{-1}		0.851	5	MR Egger	
		LV – SV (ml)		-7.32 (-17.15; 2.51)	1.4×10^{-1}		0.172	5	MR Egger	
		LV – PFR (ml/s)		-9.20 (-13.21; -5.19)	6.9×10^{-6}		0.942	5	IVW	
		LV – PER (ml/s)		-0.65 (-4.59; 3.29)	7.5×10^{-1}		0.709	5	IVW	
		LV – PAFR (ml/s)		10.19 (4.68; 15.69)	2.9×10^{-4}		0.175	5	IVW	
		LV – MVR (g/ml)		0.05 (0.01; 0.09)	2.0×10^{-2}		0.587	5	MR Egger	
		LV – ESV (ml)		-9.97 (-17.07; -2.86)	6.0×10^{-3}		0.882	5	MR Egger	
		LV – EF (%)		-0.44 (-0.77; -0.12)	7.5×10^{-3}		0.172	5	IVW	
		LV – EDV (ml)		-17.00 (-29.11; -4.90)	5.9×10^{-3}		0.670	5	MR Egger	
		LV – EDM (g)		-0.45 (-1.03; 0.13)	1.3×10^{-1}		0.528	5	IVW	
ISK2 (P20155)	SPINK2 (ENSG00000128040)	RV – SV (ml)	3.0/3.0	0.23 (-0.08; 0.55)	1.5×10^{-1}	7.81×10^{-6}	0.785	21	IVW	Interval
		RV – PFR (ml/s)		0.30 (-1.56; 2.16)	7.5×10^{-1}		0.826	20	IVW	
		RV – PER (ml/s)		-0.71 (-8.29; 6.86)	8.5×10^{-1}		0.326	22	MR Egger	
		RV – PAFR (ml/s)		-1.48 (-3.48; 0.51)	1.4×10^{-1}		0.859	21	IVW	

Table S15: Directionally concordant CMR proteins: MR effect estimates of plasma protein effects on sixteen CMR traits. *(continued)*

Protein (uniprot)	Gene (ensembl)	CMR trait	No. hits/Path. concordance	MD (95%CI)	P-value	Multiple testing threshold	Q p-value	No. variants	MR model	pQTL source
		RV – ESV (ml)		1.35 (0.95; 1.74)	2.2×10^{-11}		0.069	21	IVW	
		RV – EF (%)		-0.37 (-0.49; -0.25)	3.4×10^{-9}		0.792	21	IVW	
		RV – EDV (ml)		1.61 (1.04; 2.17)	2.4×10^{-8}		0.270	21	IVW	
		LV – SV (ml)		-0.10 (-1.43; 1.24)	8.9×10^{-1}		0.422	22	MR Egger	
		LV – PFR (ml/s)		3.44 (-4.44; 11.32)	3.9×10^{-1}		0.881	22	MR Egger	
		LV – PER (ml/s)		3.08 (-4.66; 10.82)	4.4×10^{-1}		0.553	22	MR Egger	
		LV – PAFR (ml/s)		4.64 (-4.16; 13.44)	3.0×10^{-1}		0.400	22	MR Egger	
		LV – MVR (g/ml)		0.00 (-0.01; 0.01)	9.8×10^{-1}		0.816	22	MR Egger	
		LV – ESV (ml)		-0.11 (-1.34; 1.12)	8.6×10^{-1}		0.469	22	MR Egger	
		LV – EF (%)		0.14 (-0.38; 0.65)	6.0×10^{-1}		0.407	22	MR Egger	
		LV – EDV (ml)		-0.28 (-2.38; 1.81)	7.9×10^{-1}		0.457	22	MR Egger	
		LV – EDM (g)		-0.04 (-0.35; 0.28)	8.1×10^{-1}		0.923	20	IVW	
EPHA1 (P21709)	EPHA1 (ENSG00000146904)	RV – SV (ml)	3.0/3.0	-0.04 (-0.66; 0.57)	8.9×10^{-1}	7.81×10^{-6}	0.616	16	MR Egger	Interval
		RV – PFR (ml/s)		0.58 (-2.72; 3.89)	7.3×10^{-1}		0.591	16	MR Egger	
		RV – PER (ml/s)		-1.26 (-4.70; 2.18)	4.7×10^{-1}		0.553	16	MR Egger	
		RV – PAFR (ml/s)		3.38 (1.59; 5.18)	2.3×10^{-4}		0.099	17	IVW	
		RV – ESV (ml)		-0.71 (-1.42; 0.00)	5.1×10^{-2}		0.239	14	MR Egger	
		RV – EF (%)		0.42 (0.30; 0.53)	1.3×10^{-12}		0.167	16	IVW	
		RV – EDV (ml)		-0.82 (-1.20; -0.43)	3.7×10^{-5}		0.543	17	IVW	
		LV – SV (ml)		0.23 (-0.01; 0.47)	5.8×10^{-2}		0.039	17	IVW	
		LV – PFR (ml/s)		-1.46 (-6.24; 3.33)	5.5×10^{-1}		0.069	16	MR Egger	
		LV – PER (ml/s)		-2.31 (-6.02; 1.40)	2.2×10^{-1}		0.027	16	MR Egger	
		LV – PAFR (ml/s)		1.00 (-0.56; 2.56)	2.1×10^{-1}		0.451	17	IVW	
		LV – MVR (g/ml)		0.00 (-0.00; 0.00)	5.1×10^{-1}		0.005	17	IVW	
		LV – ESV (ml)		-0.71 (-0.96; -0.45)	3.3×10^{-8}		0.217	17	IVW	
		LV – EF (%)		0.34 (0.24; 0.43)	6.0×10^{-13}		0.042	17	IVW	
		LV – EDV (ml)		-0.49 (-0.89; -0.09)	1.7×10^{-2}		0.335	17	IVW	
		LV – EDM (g)		-0.16 (-0.41; 0.08)	1.9×10^{-1}		0.148	17	IVW	
CHLE (P06276)	BCHE (ENSG00000114200)	RV – SV (ml)	3.0/3.0	-0.16 (-0.69; 0.36)	5.4×10^{-1}	7.81×10^{-6}	0.883	17	MR Egger	Framingham
		RV – PFR (ml/s)		-0.25 (-0.57; 0.06)	1.1×10^{-1}		0.038	24	IVW	
		RV – PER (ml/s)		1.67 (-2.65; 5.98)	4.5×10^{-1}		0.218	12	MR Egger	
		RV – PAFR (ml/s)		-1.33 (-1.78; -0.88)	6.3×10^{-9}		0.519	24	IVW	
		RV – ESV (ml)		-0.80 (-1.18; -0.42)	4.1×10^{-5}		0.002	20	MR Egger	
		RV – EF (%)		0.04 (-0.03; 0.11)	3.0×10^{-1}		0.024	20	IVW	
		RV – EDV (ml)		0.42 (0.33; 0.52)	1.0×10^{-100}		<0.001	23	IVW	
		LV – SV (ml)		-0.72 (-1.53; 0.10)	8.6×10^{-2}		0.150	15	MR Egger	
		LV – PFR (ml/s)		-0.37 (-0.72; -0.03)	3.6×10^{-2}		0.677	26	IVW	
		LV – PER (ml/s)		-2.42 (-8.59; 3.74)	4.4×10^{-1}		0.990	8	MR Egger	
		LV – PAFR (ml/s)		-4.03 (-5.67; -2.38)	1.6×10^{-6}		0.347	17	IVW	
		LV – MVR (g/ml)		0.00 (-0.00; 0.00)	5.3×10^{-1}		0.278	26	IVW	
		LV – ESV (ml)		-0.47 (-1.28; 0.33)	2.5×10^{-1}		0.495	11	MR Egger	
		LV – EF (%)		-0.20 (-0.37; -0.03)	2.2×10^{-2}		0.025	20	MR Egger	
		LV – EDV (ml)		-1.19 (-2.78; 0.40)	1.4×10^{-1}		0.853	10	MR Egger	
		LV – EDM (g)		-0.58 (-1.63; 0.47)	2.8×10^{-1}		0.907	7	MR Egger	
CATB (P07858)	CTSB (ENSG00000164733)	RV – SV (ml)	3.0/3.0	-0.32 (-0.53; -0.11)	3.0×10^{-3}	7.81×10^{-6}	0.009	25	IVW	Interval
		RV – PFR (ml/s)		-1.12 (-2.65; 0.40)	1.5×10^{-1}		0.626	19	IVW	
		RV – PER (ml/s)		-3.79 (-4.99; -2.59)	6.1×10^{-10}		0.003	24	IVW	
		RV – PAFR (ml/s)		-0.83 (-2.23; 0.56)	2.4×10^{-1}		0.408	24	IVW	
		RV – ESV (ml)		-0.74 (-1.68; 0.20)	1.2×10^{-1}		<0.001	16	MR Egger	
		RV – EF (%)		0.12 (-0.30; 0.53)	5.8×10^{-1}		<0.001	13	MR Egger	
		RV – EDV (ml)		-1.33 (-2.51; -0.16)	2.6×10^{-2}		0.009	23	MR Egger	
		LV – SV (ml)		-0.87 (-1.10; -0.64)	4.7×10^{-14}		0.007	23	IVW	
		LV – PFR (ml/s)		-1.36 (-2.72; -0.01)	4.9×10^{-2}		0.345	25	IVW	
		LV – PER (ml/s)		-4.93 (-6.24; -3.62)	1.4×10^{-13}		0.438	24	IVW	
		LV – PAFR (ml/s)		4.21 (-0.75; 9.17)	9.7×10^{-2}		0.189	23	MR Egger	
		LV – MVR (g/ml)		-0.00 (-0.00; -0.00)	2.7×10^{-4}		<0.001	22	IVW	
		LV – ESV (ml)		0.12 (-0.10; 0.33)	2.9×10^{-1}		<0.001	22	IVW	
		LV – EF (%)		0.01 (-0.36; 0.37)	9.8×10^{-1}		<0.001	17	MR Egger	
		LV – EDV (ml)		-1.45 (-2.68; -0.23)	2.0×10^{-2}		0.147	24	MR Egger	
		LV – EDM (g)		-0.97 (-1.74; -0.21)	1.3×10^{-2}		0.056	21	MR Egger	
C1QC (P02747)	C1QC (ENSG00000159189)	RV – SV (ml)	3.0/3.0	0.27 (-0.12; 0.66)	1.8×10^{-1}	7.81×10^{-6}	0.091	44	MR Egger	Interval
		RV – PFR (ml/s)		-0.61 (-1.36; 0.15)	1.2×10^{-1}		<0.001	42	IVW	

Table S15: Directionally concordant CMR proteins: MR effect estimates of plasma protein effects on sixteen CMR traits. *(continued)*

Protein (uniprot)	Gene (ensembl)	CMR trait	No. hits/Path. concordance	MD (95%CI)	P-value	Multiple testing threshold	Q p-value	No. variants	MR model	pQTL source
		RV – PER (ml/s)		1.70 (-0.30; 3.69)	9.5×10^{-2}		0.003	40	MR Egger	
		RV – PAFR (ml/s)		1.37 (-0.94; 3.69)	2.4×10^{-1}		0.254	44	MR Egger	
		RV – ESV (ml)		0.44 (0.26; 0.61)	7.7×10^{-7}		0.095	42	IVW	
		RV – EF (%)		-0.13 (-0.18; -0.07)	1.3×10^{-5}		0.657	42	IVW	
		RV – EDV (ml)		0.67 (0.42; 0.92)	9.5×10^{-8}		0.018	42	IVW	
		LV – SV (ml)		0.61 (0.23; 0.99)	1.7×10^{-3}		0.218	44	MR Egger	
		LV – PFR (ml/s)		-0.41 (-1.26; 0.44)	3.4×10^{-1}		0.961	44	IVW	
		LV – PER (ml/s)		-1.04 (-1.90; -0.18)	1.8×10^{-2}		0.039	41	IVW	
		LV – PAFR (ml/s)		0.89 (-1.58; 3.36)	4.8×10^{-1}		0.272	44	MR Egger	
		LV – MVR (g/ml)		-0.00 (-0.00; 0.00)	4.2×10^{-1}		0.003	42	MR Egger	
		LV – ESV (ml)		0.39 (0.25; 0.53)	7.7×10^{-8}		<0.001	39	IVW	
		LV – EF (%)		-0.15 (-0.29; -0.00)	4.4×10^{-2}		0.007	41	MR Egger	
		LV – EDV (ml)		0.49 (0.27; 0.72)	2.0×10^{-5}		0.015	43	IVW	
		LV – EDM (g)		0.28 (-0.04; 0.60)	9.1×10^{-2}		0.013	43	MR Egger	
BSSP4 (Q9GZN4)	PRSS22 (ENSG00000005001)	RV – SV (ml)	3.0/3.0	1.11 (0.43; 1.79)	1.3×10^{-3}	7.81×10^{-6}	0.444	19	MR Egger	Interval
		RV – PFR (ml/s)		4.53 (0.91; 8.15)	1.4×10^{-2}		0.810	19	MR Egger	
		RV – PER (ml/s)		1.97 (0.57; 3.37)	5.9×10^{-3}		0.074	16	IVW	
		RV – PAFR (ml/s)		3.31 (-0.97; 7.59)	1.3×10^{-1}		0.927	19	MR Egger	
		RV – ESV (ml)		-0.01 (-0.20; 0.19)	9.5×10^{-1}		0.039	18	IVW	
		RV – EF (%)		0.34 (0.26; 0.42)	1.0×10^{-100}		0.303	18	IVW	
		RV – EDV (ml)		1.65 (0.53; 2.77)	3.9×10^{-3}		0.048	19	MR Egger	
		LV – SV (ml)		1.29 (0.60; 1.98)	2.6×10^{-4}		0.501	19	MR Egger	
		LV – PFR (ml/s)		4.99 (3.70; 6.28)	3.5×10^{-14}		0.209	18	IVW	
		LV – PER (ml/s)		3.07 (-0.99; 7.13)	1.4×10^{-1}		0.686	19	MR Egger	
		LV – PAFR (ml/s)		2.47 (-2.04; 6.98)	2.8×10^{-1}		0.535	19	MR Egger	
		LV – MVR (g/ml)		-0.00 (-0.00; 0.00)	7.9×10^{-1}		0.505	18	IVW	
		LV – ESV (ml)		-0.04 (-0.22; 0.14)	6.5×10^{-1}		0.510	18	IVW	
		LV – EF (%)		0.22 (0.15; 0.30)	8.0×10^{-9}		0.487	17	IVW	
		LV – EDV (ml)		1.45 (0.35; 2.55)	9.8×10^{-3}		0.449	19	MR Egger	
		LV – EDM (g)		0.66 (0.05; 1.27)	3.4×10^{-2}		0.426	19	MR Egger	
ASM3A (Q92484)	SMPDL3A (ENSG00000172594)	RV – SV (ml)	3.0/3.0	0.47 (0.31; 0.62)	4.1×10^{-9}	7.81×10^{-6}	0.051	36	IVW	Interval
		RV – PFR (ml/s)		0.68 (-0.02; 1.38)	5.8×10^{-2}		<0.001	35	IVW	
		RV – PER (ml/s)		2.30 (1.60; 3.01)	1.5×10^{-10}		<0.001	37	IVW	
		RV – PAFR (ml/s)		1.26 (-0.83; 3.35)	2.4×10^{-1}		0.717	38	MR Egger	
		RV – ESV (ml)		0.24 (0.08; 0.39)	2.6×10^{-3}		0.151	36	IVW	
		RV – EF (%)		0.28 (0.16; 0.41)	1.0×10^{-5}		<0.001	38	MR Egger	
		RV – EDV (ml)		0.60 (-0.01; 1.22)	5.4×10^{-2}		0.086	40	MR Egger	
		LV – SV (ml)		0.38 (0.03; 0.74)	3.5×10^{-2}		0.417	34	MR Egger	
		LV – PFR (ml/s)		2.00 (-0.24; 4.23)	8.0×10^{-2}		0.031	36	MR Egger	
		LV – PER (ml/s)		4.78 (2.81; 6.76)	1.9×10^{-6}		<0.001	38	MR Egger	
		LV – PAFR (ml/s)		-1.16 (-2.07; -0.24)	1.3×10^{-2}		0.058	40	IVW	
		LV – MVR (g/ml)		-0.00 (-0.00; -0.00)	1.8×10^{-3}		0.007	39	MR Egger	
		LV – ESV (ml)		-0.35 (-0.71; 0.01)	5.8×10^{-2}		0.120	37	MR Egger	
		LV – EF (%)		0.16 (-0.00; 0.33)	5.1×10^{-2}		0.269	34	MR Egger	
		LV – EDV (ml)		0.26 (0.05; 0.47)	1.5×10^{-2}		0.356	38	IVW	
		LV – EDM (g)		-0.51 (-0.80; -0.22)	5.5×10^{-4}		0.010	39	MR Egger	

General:
CMR: Cardiac MRI, MR: Mendelian randomization, MD: mean difference, CI: confidence interval. Effect estimates are coded towards protein and CMR increasing directions.

Table S16: Phenome-wide drug target MR scan of proteins with a prioritized CMR association.

Protein (uniprot)	Protein set	Trait	Estimate (95%CI)	P-value	Q p-value	No. variants	MR model	pQTL source
AT1B2 (P14415)	Drugged	HF (OR)	0.99 (0.97; 1.01)	1.7×10^{-1}	<0.001	14	IVW	Interval
		Non-ischemic CM (OR)	0.98 (0.76; 1.26)	8.5×10^{-1}	0.105	13	MR Egger	
		DCM (OR)	1.22 (1.10; 1.35)	9.5×10^{-5}	0.525	16	IVW	
		AF (OR)	1.00 (0.88; 1.13)	9.9×10^{-1}	0.091	12	MR Egger	
		CHD (OR)	1.09 (1.01; 1.19)	3.7×10^{-2}	0.253	13	MR Egger	
		cIMT (mm)	0.00 (-0.00; 0.01)	6.5×10^{-1}	0.002	11	MR Egger	
		Carotid plaque (OR)	0.94 (0.85; 1.04)	2.4×10^{-1}	<0.001	13	MR Egger	
		Any stroke (OR)	1.04 (1.02; 1.06)	2.2×10^{-5}	0.049	15	IVW	
		Any ischemic stroke (OR)	1.05 (1.02; 1.08)	2.7×10^{-4}	0.083	15	IVW	
		LDL cholesterol (mmol/l)	0.04 (0.04; 0.05)	1.0×10^{-100}	<0.001	13	IVW	
		Apoliprotein B (g/l)	0.01 (0.01; 0.01)	1.0×10^{-100}	<0.001	14	IVW	
		Triglycerides (mmol/l)	0.02 (0.00; 0.04)	2.2×10^{-2}	<0.001	15	MR Egger	
		Cholesterol (mmol/l)	0.04 (0.03; 0.05)	1.0×10^{-100}	<0.001	11	IVW	
		HDL cholesterol (mmol/l)	-0.02 (-0.02; -0.01)	1.0×10^{-100}	<0.001	9	IVW	
		Apoliprotein A1 (g/l)	-0.01 (-0.02; -0.00)	6.8×10^{-3}	<0.001	9	MR Egger	
		Glucose (mmol/l)	-0.03 (-0.05; -0.01)	2.0×10^{-3}	<0.001	17	MR Egger	
		Glycated haemoglobin (mmol/mol)	0.42 (0.33; 0.51)	1.0×10^{-100}	0.117	11	IVW	
		T2DM (OR)	0.96 (0.92; 1.00)	6.8×10^{-2}	0.454	11	MR Egger	
		BMI (SD)	-0.02 (-0.03; -0.01)	3.7×10^{-3}	<0.001	7	IVW	
		SBP (mmHg)	0.16 (-0.11; 0.43)	2.4×10^{-1}	0.116	8	MR Egger	
		DBP (mmHg)	0.25 (-0.03; 0.53)	8.1×10^{-2}	0.954	5	MR Egger	
		ECG heart rate (exercise) (BPM)	0.17 (-0.54; 0.88)	6.4×10^{-1}	0.153	18	MR Egger	
		ECG load (exercise) (Watts)	-0.22 (-0.94; 0.49)	5.4×10^{-1}	0.369	18	MR Egger	
		Asthma (OR)	1.01 (1.00; 1.03)	1.5×10^{-1}	0.015	16	IVW	
		CRP (log(mg/L))	-0.02 (-0.04; -0.00)	1.4×10^{-2}	0.130	12	IVW	
		eGFR (SD of log(eGFR))	-0.01 (-0.01; -0.01)	5.7×10^{-11}	<0.001	13	MR Egger	
		BUN (mg/dl)	0.01 (0.00; 0.01)	3.5×10^{-4}	0.093	10	IVW	
		CKD (OR)	1.05 (0.99; 1.11)	9.5×10^{-2}	<0.001	15	MR Egger	
		Alzheimer’s (OR)	0.99 (0.97; 1.01)	3.0×10^{-1}	0.260	16	MR Egger	
		Parkinson’s (OR)	1.06 (0.94; 1.20)	3.1×10^{-1}	0.006	13	MR Egger	
		Lewy body dementia (OR)	1.29 (0.93; 1.80)	1.3×10^{-1}	0.203	15	MR Egger	
		Breast cancer (OR)	1.05 (1.03; 1.08)	3.8×10^{-5}	0.007	16	IVW	
		Lung cancer (OR)	0.95 (0.90; 1.00)	5.8×10^{-2}	0.676	17	IVW	
		Colon cancer (OR)	1.49 (1.19; 1.86)	4.6×10^{-4}	0.694	17	MR Egger	
		Prostate cancer (OR)	1.05 (1.02; 1.09)	1.7×10^{-3}	0.043	13	IVW	
CAH6 (P23280)	Drugged	HF (OR)	1.01 (0.99; 1.03)	2.8×10^{-1}	0.027	39	MR Egger	Interval
		Non-ischemic CM (OR)	1.00 (0.96; 1.04)	9.5×10^{-1}	<0.001	39	IVW	
		DCM (OR)	1.18 (1.13; 1.23)	6.2×10^{-15}	0.101	43	IVW	
		AF (OR)	0.97 (0.95; 0.99)	1.2×10^{-3}	0.014	46	MR Egger	
		CHD (OR)	1.00 (0.97; 1.03)	9.0×10^{-1}	0.048	42	MR Egger	
		cIMT (mm)	-0.00 (-0.00; -0.00)	5.5×10^{-7}	<0.001	40	IVW	
		Carotid plaque (OR)	1.00 (0.96; 1.05)	9.0×10^{-1}	0.256	40	MR Egger	
		Any stroke (OR)	1.02 (0.99; 1.04)	1.5×10^{-1}	0.012	42	MR Egger	
		Any ischemic stroke (OR)	1.05 (1.02; 1.09)	1.1×10^{-3}	0.564	39	MR Egger	
		LDL cholesterol (mmol/l)	-0.00 (-0.01; 0.00)	2.1×10^{-1}	0.524	50	MR Egger	
		Apoliprotein B (g/l)	-0.00 (-0.00; 0.00)	8.8×10^{-1}	0.099	47	IVW	
		Triglycerides (mmol/l)	0.00 (-0.00; 0.00)	9.2×10^{-1}	0.004	43	IVW	
		Cholesterol (mmol/l)	-0.01 (-0.02; -0.00)	1.0×10^{-2}	0.145	50	MR Egger	
		HDL cholesterol (mmol/l)	-0.00 (-0.01; -0.00)	8.6×10^{-4}	0.081	49	MR Egger	
		Apoliprotein A1 (g/l)	-0.00 (-0.00; 0.00)	5.3×10^{-2}	0.121	48	MR Egger	
		Glucose (mmol/l)	-0.00 (-0.01; 0.01)	6.8×10^{-1}	0.019	50	MR Egger	
		Glycated haemoglobin (mmol/mol)	-0.03 (-0.08; 0.01)	1.4×10^{-1}	0.571	43	MR Egger	
		T2DM (OR)	1.01 (1.00; 1.02)	4.3×10^{-2}	<0.001	39	IVW	
		BMI (SD)	0.00 (0.00; 0.01)	3.1×10^{-2}	0.021	33	IVW	
		SBP (mmHg)	0.12 (0.08; 0.16)	1.8×10^{-8}	0.003	32	IVW	
		DBP (mmHg)	0.05 (0.03; 0.08)	6.6×10^{-6}	0.098	38	IVW	
		ECG heart rate (exercise) (BPM)	-0.06 (-0.15; 0.03)	2.1×10^{-1}	0.042	46	IVW	
		ECG load (exercise) (Watts)	-0.11 (-0.36; 0.13)	3.6×10^{-1}	0.312	50	MR Egger	
		Asthma (OR)	1.04 (1.03; 1.04)	1.0×10^{-100}	<0.001	34	IVW	
		CRP (log(mg/L))	0.01 (0.01; 0.02)	3.7×10^{-9}	0.182	38	IVW	
		eGFR (SD of log(eGFR))	0.00 (-0.00; 0.00)	8.5×10^{-1}	<0.001	39	IVW	
		BUN (mg/dl)	0.00 (-0.00; 0.00)	4.1×10^{-1}	0.121	40	MR Egger	
		CKD (OR)	1.02 (1.00; 1.05)	8.4×10^{-2}	<0.001	41	MR Egger	
		Alzheimer’s (OR)	1.00 (1.00; 1.00)	3.7×10^{-2}	<0.001	40	IVW	
		Parkinson’s (OR)	0.95 (0.89; 1.02)	1.4×10^{-1}	0.621	38	MR Egger	
		Lewy body dementia (OR)	0.98 (0.94; 1.03)	4.4×10^{-1}	0.307	39	IVW	

Table S16: Phenome-wide drug target MR scan of proteins with a prioritized CMR association. *(continued)*

Protein (uniprot)	Protein set	Trait	Estimate (95%CI)	P-value	Q p-value	No. variants	MR model	pQTL source
		Breast cancer (OR)	1.00 (0.95; 1.04)	9.6×10^{-1}	<0.001	37	MR Egger	
		Lung cancer (OR)	0.99 (0.95; 1.02)	4.3×10^{-1}	0.013	41	IVW	
		Colon cancer (OR)	1.03 (1.00; 1.07)	4.7×10^{-2}	0.181	41	IVW	
		Prostate cancer (OR)	0.97 (0.95; 0.99)	6.3×10^{-4}	0.014	38	IVW	
CD33 (P20138)	Drugged	HF (OR)	0.96 (0.95; 0.98)	9.6×10^{-6}	0.038	40	MR Egger	Interval
		Non-ischemic CM (OR)	0.96 (0.89; 1.03)	2.1×10^{-1}	0.079	45	MR Egger	
		DCM (OR)	0.95 (0.85; 1.06)	3.5×10^{-1}	0.594	36	MR Egger	
		AF (OR)	0.95 (0.94; 0.96)	5.3×10^{-15}	<0.001	46	MR Egger	
		CHD (OR)	1.01 (1.00; 1.02)	1.7×10^{-2}	0.736	41	IVW	
		cIMT (mm)	0.00 (-0.00; 0.00)	7.2×10^{-1}	0.060	40	MR Egger	
		Carotid plaque (OR)	0.90 (0.85; 0.94)	3.1×10^{-5}	0.665	37	MR Egger	
		Any stroke (OR)	1.00 (0.98; 1.02)	6.7×10^{-1}	0.026	43	MR Egger	
		Any ischemic stroke (OR)	0.99 (0.97; 1.02)	5.8×10^{-1}	0.031	42	MR Egger	
		LDL cholesterol (mmol/l)	0.00 (-0.00; 0.01)	9.6×10^{-2}	0.257	51	MR Egger	
		Apoliprotein B (g/l)	0.00 (0.00; 0.00)	7.1×10^{-3}	0.156	52	MR Egger	
		Triglycerides (mmol/l)	0.00 (0.00; 0.01)	7.0×10^{-5}	0.161	52	IVW	
		Cholesterol (mmol/l)	0.00 (-0.00; 0.01)	6.4×10^{-1}	0.084	52	MR Egger	
		HDL cholesterol (mmol/l)	-0.00 (-0.00; -0.00)	1.6×10^{-2}	0.053	52	MR Egger	
		Apoliprotein A1 (g/l)	-0.00 (-0.00; 0.00)	8.2×10^{-2}	<0.001	53	MR Egger	
		Glucose (mmol/l)	-0.00 (-0.01; -0.00)	1.0×10^{-3}	0.003	51	IVW	
		Glycated haemoglobin (mmol/mol)	0.13 (0.11; 0.16)	1.0×10^{-100}	0.003	52	MR Egger	
		T2DM (OR)	1.00 (0.98; 1.01)	8.1×10^{-1}	0.029	43	MR Egger	
		BMI (SD)	0.01 (0.00; 0.01)	2.3×10^{-2}	<0.001	38	MR Egger	
		SBP (mmHg)	0.11 (0.03; 0.19)	6.4×10^{-3}	0.019	31	MR Egger	
		DBP (mmHg)	0.04 (0.02; 0.06)	3.8×10^{-5}	0.022	31	IVW	
		ECG heart rate (exercise) (BPM)	-0.16 (-0.35; 0.04)	1.2×10^{-1}	0.159	50	MR Egger	
		ECG load (exercise) (Watts)	0.00 (-0.20; 0.20)	9.7×10^{-1}	0.002	50	MR Egger	
		Asthma (OR)	1.01 (1.00; 1.02)	2.9×10^{-1}	0.013	52	MR Egger	
		CRP (log(mg/L))	0.03 (0.02; 0.04)	4.3×10^{-6}	<0.001	32	MR Egger	
		eGFR (SD of log(eGFR))	-0.00 (-0.00; 0.00)	1.1×10^{-1}	0.034	40	MR Egger	
		BUN (mg/dl)	-0.00 (-0.01; -0.00)	7.3×10^{-3}	0.751	40	MR Egger	
		CKD (OR)	0.99 (0.97; 1.01)	3.2×10^{-1}	0.459	44	MR Egger	
		Alzheimer's (OR)	1.02 (1.02; 1.03)	1.0×10^{-100}	0.004	44	MR Egger	
		Parkinson's (OR)	1.05 (1.01; 1.08)	1.9×10^{-2}	0.020	43	MR Egger	
		Lewy body dementia (OR)	0.96 (0.89; 1.03)	2.2×10^{-1}	<0.001	43	MR Egger	
		Breast cancer (OR)	1.01 (1.00; 1.02)	1.5×10^{-1}	0.024	49	IVW	
		Lung cancer (OR)	1.00 (0.94; 1.07)	9.6×10^{-1}	0.235	46	MR Egger	
		Colon cancer (OR)	1.03 (0.98; 1.07)	2.8×10^{-1}	0.012	46	MR Egger	
		Prostate cancer (OR)	0.93 (0.91; 0.96)	4.9×10^{-6}	0.065	49	MR Egger	
CO6A1 (P12109)	Drugged	HF (OR)	1.07 (0.95; 1.19)	2.7×10^{-1}	0.550	15	MR Egger	Interval
		Non-ischemic CM (OR)	1.53 (1.35; 1.74)	2.9×10^{-11}	0.744	11	IVW	
		DCM (OR)	1.02 (0.57; 1.82)	9.6×10^{-1}	0.247	18	MR Egger	
		AF (OR)	1.02 (0.98; 1.06)	4.4×10^{-1}	0.131	12	IVW	
		CHD (OR)	0.98 (0.96; 1.01)	1.7×10^{-1}	0.413	15	IVW	
		cIMT (mm)	-0.01 (-0.02; 0.00)	1.7×10^{-1}	0.368	15	MR Egger	
		Carotid plaque (OR)	0.89 (0.83; 0.95)	5.2×10^{-4}	0.111	13	IVW	
		Any stroke (OR)	1.03 (0.99; 1.07)	1.4×10^{-1}	0.434	14	IVW	
		Any ischemic stroke (OR)	1.01 (0.97; 1.05)	6.3×10^{-1}	0.590	14	IVW	
		LDL cholesterol (mmol/l)	-0.01 (-0.03; 0.02)	6.9×10^{-1}	0.674	16	MR Egger	
		Apoliprotein B (g/l)	0.00 (-0.00; 0.01)	2.2×10^{-1}	0.142	17	MR Egger	
		Triglycerides (mmol/l)	0.02 (0.01; 0.02)	1.0×10^{-9}	0.484	17	IVW	
		Cholesterol (mmol/l)	0.03 (0.02; 0.03)	4.6×10^{-13}	0.187	16	IVW	
		HDL cholesterol (mmol/l)	-0.00 (-0.01; 0.01)	6.3×10^{-1}	0.265	16	MR Egger	
		Apoliprotein A1 (g/l)	-0.00 (-0.00; 0.00)	9.5×10^{-1}	0.174	17	IVW	
		Glucose (mmol/l)	-0.01 (-0.04; 0.03)	6.7×10^{-1}	0.002	17	MR Egger	
		Glycated haemoglobin (mmol/mol)	-0.01 (-0.19; 0.16)	8.8×10^{-1}	0.909	17	MR Egger	
		T2DM (OR)	0.94 (0.86; 1.02)	1.5×10^{-1}	0.040	13	MR Egger	
		BMI (SD)	-0.00 (-0.01; 0.01)	8.1×10^{-1}	0.700	13	IVW	
		SBP (mmHg)	-0.30 (-0.46; -0.14)	2.2×10^{-4}	0.350	8	IVW	
		DBP (mmHg)	-0.33 (-0.41; -0.25)	2.2×10^{-16}	0.014	10	IVW	
		ECG heart rate (exercise) (BPM)	-0.33 (-1.31; 0.66)	5.1×10^{-1}	0.558	17	MR Egger	
		ECG load (exercise) (Watts)	0.31 (-0.79; 1.42)	5.8×10^{-1}	0.596	17	MR Egger	
		Asthma (OR)	1.00 (0.93; 1.08)	9.0×10^{-1}	0.261	17	MR Egger	
		CRP (log(mg/L))	-0.01 (-0.03; -0.00)	3.8×10^{-2}	0.633	14	IVW	
		eGFR (SD of log(eGFR))	-0.00 (-0.00; 0.00)	6.3×10^{-1}	0.004	11	IVW	
		BUN (mg/dl)	0.00 (-0.00; 0.01)	5.3×10^{-2}	<0.001	12	IVW	

Table S16: Phenome-wide drug target MR scan of proteins with a prioritized CMR association. *(continued)*

Protein (uniprot)	Protein set	Trait	Estimate (95%CI)	P-value	Q p-value	No. variants	MR model	pQTL source
		CKD (OR)	1.05 (1.01; 1.09)	2.7×10^{-2}	0.433	11	IVW	
		Alzheimer's (OR)	1.00 (0.99; 1.01)	4.7×10^{-1}	0.014	15	IVW	
		Parkinson's (OR)	1.01 (0.93; 1.10)	8.2×10^{-1}	0.302	11	IVW	
		Lewy body dementia (OR)	1.35 (1.12; 1.62)	1.4×10^{-3}	0.125	11	IVW	
		Breast cancer (OR)	0.94 (0.90; 0.98)	8.0×10^{-3}	0.829	11	IVW	
		Lung cancer (OR)	1.30 (1.15; 1.46)	3.1×10^{-5}	0.198	12	IVW	
		Colon cancer (OR)	1.00 (0.63; 1.58)	1.0×10^{0}	0.745	10	MR Egger	
		Prostate cancer (OR)	0.94 (0.89; 0.99)	3.0×10^{-2}	0.007	12	IVW	
COFA1 (P39059)	Drugged	HF (OR)	0.91 (0.82; 1.02)	1.1×10^{-1}	0.916	15	MR Egger	Interval
		Non-ischemic CM (OR)	1.03 (0.94; 1.11)	5.6×10^{-1}	0.964	16	IVW	
		DCM (OR)	0.59 (0.36; 0.98)	4.2×10^{-2}	0.260	16	MR Egger	
		AF (OR)	1.12 (1.09; 1.15)	2.2×10^{-16}	0.577	15	IVW	
		CHD (OR)	0.99 (0.97; 1.01)	3.3×10^{-1}	0.850	15	IVW	
		cIMT (mm)	-0.00 (-0.00; 0.00)	2.9×10^{-1}	0.664	15	IVW	
		Carotid plaque (OR)	0.93 (0.88; 0.98)	1.2×10^{-2}	0.741	13	IVW	
		Any stroke (OR)	1.05 (1.02; 1.08)	4.9×10^{-4}	0.766	15	IVW	
		Any ischemic stroke (OR)	1.01 (0.91; 1.11)	9.2×10^{-1}	0.738	17	MR Egger	
		LDL cholesterol (mmol/l)	-0.01 (-0.03; 0.02)	5.9×10^{-1}	0.936	18	MR Egger	
		Apoliprotein B (g/l)	-0.01 (-0.01; -0.00)	1.5×10^{-10}	0.987	18	IVW	
		Triglycerides (mmol/l)	-0.02 (-0.05; 0.00)	8.6×10^{-2}	0.854	18	MR Egger	
		Cholesterol (mmol/l)	-0.03 (-0.04; -0.02)	1.6×10^{-10}	0.804	18	IVW	
		HDL cholesterol (mmol/l)	0.01 (0.00; 0.01)	9.7×10^{-6}	0.702	18	IVW	
		Apoliprotein A1 (g/l)	0.00 (0.00; 0.01)	3.9×10^{-5}	0.732	18	IVW	
		Glucose (mmol/l)	-0.02 (-0.03; -0.01)	3.4×10^{-4}	0.978	18	IVW	
		Glycated haemoglobin (mmol/mol)	-0.06 (-0.25; 0.13)	5.2×10^{-1}	0.868	17	MR Egger	
		T2DM (OR)	1.02 (1.00; 1.04)	4.5×10^{-2}	0.822	15	IVW	
		BMI (SD)	0.02 (0.02; 0.03)	2.2×10^{-9}	0.033	12	IVW	
		SBP (mmHg)	-0.29 (-0.42; -0.16)	8.4×10^{-6}	<0.001	10	IVW	
		DBP (mmHg)	-0.16 (-0.37; 0.06)	1.5×10^{-1}	0.052	17	MR Egger	
		ECG heart rate (exercise) (BPM)	-0.25 (-0.53; 0.02)	7.3×10^{-2}	0.850	18	IVW	
		ECG load (exercise) (Watts)	0.07 (-1.38; 1.52)	9.2×10^{-1}	0.063	18	MR Egger	
		Asthma (OR)	1.10 (1.02; 1.18)	1.7×10^{-2}	0.033	17	MR Egger	
		CRP (log(mg/L))	0.00 (-0.01; 0.02)	7.1×10^{-1}	0.691	14	IVW	
		eGFR (SD of log(eGFR))	0.00 (0.00; 0.00)	3.2×10^{-10}	0.445	15	IVW	
		BUN (mg/dl)	0.01 (0.00; 0.01)	3.8×10^{-4}	0.898	15	IVW	
		CKD (OR)	1.00 (0.90; 1.10)	9.3×10^{-1}	0.259	17	MR Egger	
		Alzheimer's (OR)	1.01 (1.00; 1.02)	5.1×10^{-2}	0.300	15	IVW	
		Parkinson's (OR)	1.10 (0.89; 1.36)	3.6×10^{-1}	0.758	16	MR Egger	
		Lewy body dementia (OR)	1.69 (1.18; 2.41)	3.9×10^{-3}	0.408	17	MR Egger	
		Breast cancer (OR)	0.97 (0.88; 1.08)	6.4×10^{-1}	0.401	17	MR Egger	
		Lung cancer (OR)	0.91 (0.69; 1.20)	4.9×10^{-1}	0.824	17	MR Egger	
		Colon cancer (OR)	1.11 (0.89; 1.38)	3.7×10^{-1}	0.438	17	MR Egger	
		Prostate cancer (OR)	1.21 (1.05; 1.38)	6.7×10^{-3}	0.444	17	MR Egger	
EPHA1 (P21709)	Drugged	HF (OR)	1.01 (0.98; 1.03)	7.1×10^{-1}	0.601	17	MR Egger	Interval
		Non-ischemic CM (OR)	0.97 (0.90; 1.03)	3.0×10^{-1}	0.264	16	IVW	
		DCM (OR)	1.14 (1.05; 1.23)	1.0×10^{-3}	0.092	17	IVW	
		AF (OR)	1.01 (0.99; 1.04)	3.1×10^{-1}	0.394	16	MR Egger	
		CHD (OR)	1.00 (0.96; 1.04)	9.6×10^{-1}	0.151	16	MR Egger	
		cIMT (mm)	0.00 (-0.00; 0.00)	1.6×10^{-1}	0.265	17	IVW	
		Carotid plaque (OR)	0.97 (0.92; 1.03)	3.3×10^{-1}	0.437	17	MR Egger	
		Any stroke (OR)	1.01 (0.97; 1.04)	7.9×10^{-1}	0.210	17	MR Egger	
		Any ischemic stroke (OR)	1.01 (0.97; 1.06)	5.5×10^{-1}	0.055	17	MR Egger	
		LDL cholesterol (mmol/l)	-0.02 (-0.03; -0.00)	8.5×10^{-3}	0.281	15	MR Egger	
		Apoliprotein B (g/l)	-0.00 (-0.01; -0.00)	2.6×10^{-2}	0.274	15	MR Egger	
		Triglycerides (mmol/l)	0.00 (-0.01; 0.01)	9.1×10^{-1}	0.826	16	MR Egger	
		Cholesterol (mmol/l)	-0.02 (-0.04; -0.01)	6.7×10^{-3}	0.198	15	MR Egger	
		HDL cholesterol (mmol/l)	-0.00 (-0.01; -0.00)	6.1×10^{-5}	0.331	17	IVW	
		Apoliprotein A1 (g/l)	-0.01 (-0.01; -0.00)	5.4×10^{-3}	0.249	16	MR Egger	
		Glucose (mmol/l)	0.03 (0.01; 0.04)	1.8×10^{-3}	0.512	15	MR Egger	
		Glycated haemoglobin (mmol/mol)	-0.05 (-0.08; -0.02)	8.2×10^{-4}	0.021	17	IVW	
		T2DM (OR)	1.01 (0.98; 1.03)	5.6×10^{-1}	0.666	16	MR Egger	
		BMI (SD)	0.00 (-0.01; 0.01)	6.5×10^{-1}	0.265	16	MR Egger	
		SBP (mmHg)	-0.05 (-0.16; 0.07)	4.2×10^{-1}	0.009	16	MR Egger	
		DBP (mmHg)	0.06 (0.02; 0.11)	6.2×10^{-3}	0.048	13	IVW	
		ECG heart rate (exercise) (BPM)	0.32 (0.08; 0.55)	8.2×10^{-3}	0.107	15	IVW	
		ECG load (exercise) (Watts)	-0.43 (-0.63; -0.24)	1.3×10^{-5}	0.909	17	IVW	

Table S16: Phenome-wide drug target MR scan of proteins with a prioritized CMR association. *(continued)*

Protein (uniprot)	Protein set	Trait	Estimate (95%CI)	P-value	Q p-value	No. variants	MR model	pQTL source
		Asthma (OR)	1.03 (1.02; 1.05)	1.5×10^{-6}	0.602	16	IVW	
		CRP (log(mg/L))	-0.00 (-0.02; 0.01)	5.0×10^{-1}	0.501	14	IVW	
		eGFR (SD of log(eGFR))	-0.00 (-0.00; -0.00)	4.4×10^{-3}	0.497	15	IVW	
		BUN (mg/dl)	-0.00 (-0.01; -0.00)	1.4×10^{-2}	0.431	16	MR Egger	
		CKD (OR)	1.02 (1.01; 1.04)	4.1×10^{-3}	0.566	16	IVW	
		Alzheimer's (OR)	0.98 (0.96; 1.00)	2.4×10^{-2}	0.053	8	IVW	
		Parkinson's (OR)	1.04 (0.99; 1.09)	8.9×10^{-2}	0.058	15	IVW	
		Lewy body dementia (OR)	1.04 (0.96; 1.12)	3.7×10^{-1}	0.201	14	IVW	
		Breast cancer (OR)	1.00 (0.94; 1.05)	9.0×10^{-1}	0.053	16	MR Egger	
		Lung cancer (OR)	0.93 (0.83; 1.04)	2.0×10^{-1}	0.867	15	MR Egger	
		Colon cancer (OR)	0.90 (0.80; 1.02)	9.8×10^{-2}	0.014	16	MR Egger	
		Prostate cancer (OR)	0.97 (0.92; 1.03)	3.5×10^{-1}	0.978	16	MR Egger	
EPHB6 (O15197)	Drugged	HF (OR)	0.96 (0.87; 1.06)	3.8×10^{-1}	0.664	10	MR Egger	Interval
		Non-ischemic CM (OR)	1.02 (0.83; 1.24)	8.8×10^{-1}	0.113	11	IVW	
		DCM (OR)	1.07 (0.95; 1.20)	2.8×10^{-1}	0.031	10	IVW	
		AF (OR)	1.05 (1.02; 1.09)	6.5×10^{-4}	0.532	12	IVW	
		CHD (OR)	1.12 (1.00; 1.25)	5.5×10^{-2}	0.720	11	MR Egger	
		cIMT (mm)	-0.00 (-0.01; 0.00)	5.5×10^{-2}	0.139	13	IVW	
		Carotid plaque (OR)	1.05 (1.00; 1.10)	5.3×10^{-2}	0.497	12	IVW	
		Any stroke (OR)	0.99 (0.96; 1.02)	4.1×10^{-1}	0.328	13	IVW	
		Any ischemic stroke (OR)	1.01 (0.98; 1.04)	5.1×10^{-1}	0.530	13	IVW	
		LDL cholesterol (mmol/l)	-0.00 (-0.01; 0.00)	4.3×10^{-1}	0.888	13	IVW	
		Apoliprotein B (g/l)	-0.00 (-0.00; 0.00)	4.2×10^{-1}	0.737	13	IVW	
		Triglycerides (mmol/l)	-0.00 (-0.01; 0.01)	6.0×10^{-1}	0.142	13	IVW	
		Cholesterol (mmol/l)	-0.01 (-0.02; 0.00)	1.7×10^{-1}	0.944	13	IVW	
		HDL cholesterol (mmol/l)	-0.00 (-0.00; 0.00)	3.6×10^{-1}	0.752	13	IVW	
		Apoliprotein A1 (g/l)	-0.00 (-0.00; 0.00)	5.2×10^{-2}	0.855	13	IVW	
		Glucose (mmol/l)	0.01 (-0.00; 0.02)	7.7×10^{-2}	0.857	12	IVW	
		Glycated haemoglobin (mmol/mol)	-0.06 (-0.14; 0.01)	7.5×10^{-2}	0.110	13	IVW	
		T2DM (OR)	0.99 (0.96; 1.02)	5.5×10^{-1}	0.689	11	IVW	
		BMI (SD)	0.01 (-0.01; 0.03)	3.9×10^{-1}	0.191	11	MR Egger	
		SBP (mmHg)	0.31 (-0.42; 1.04)	4.0×10^{-1}	0.072	7	MR Egger	
		DBP (mmHg)	0.02 (-0.10; 0.14)	7.3×10^{-1}	0.339	8	IVW	
		ECG heart rate (exercise) (BPM)	-0.29 (-0.62; 0.04)	8.0×10^{-2}	0.670	12	IVW	
		ECG load (exercise) (Watts)	-0.66 (-1.11; -0.21)	4.0×10^{-3}	0.114	13	IVW	
		Asthma (OR)	0.98 (0.96; 1.02)	3.3×10^{-1}	0.052	13	IVW	
		CRP (log(mg/L))	-0.00 (-0.02; 0.01)	6.6×10^{-1}	0.630	11	IVW	
		eGFR (SD of log(eGFR))	-0.00 (-0.01; 0.00)	2.3×10^{-1}	0.091	11	MR Egger	
		BUN (mg/dl)	-0.00 (-0.01; 0.00)	7.0×10^{-1}	0.089	11	IVW	
		CKD (OR)	0.98 (0.93; 1.03)	3.6×10^{-1}	0.500	10	IVW	
		Alzheimer's (OR)	1.01 (1.00; 1.02)	1.5×10^{-2}	0.203	12	IVW	
		Parkinson's (OR)	1.21 (0.95; 1.55)	1.2×10^{-1}	0.462	12	MR Egger	
		Lewy body dementia (OR)	1.02 (0.86; 1.20)	8.5×10^{-1}	0.755	10	IVW	
		Breast cancer (OR)	1.04 (0.99; 1.08)	8.9×10^{-2}	0.399	12	IVW	
		Lung cancer (OR)	0.85 (0.76; 0.95)	3.3×10^{-3}	0.035	11	IVW	
		Colon cancer (OR)	1.01 (0.74; 1.38)	9.5×10^{-1}	0.713	11	MR Egger	
		Prostate cancer (OR)	1.00 (0.82; 1.21)	9.8×10^{-1}	0.400	11	MR Egger	
FGFR3 (P22607)	Drugged	HF (OR)	0.92 (0.86; 0.98)	1.4×10^{-2}	0.174	7	IVW	Interval
		Non-ischemic CM (OR)	1.09 (0.85; 1.39)	4.9×10^{-1}	0.587	5	IVW	
		DCM (OR)	1.49 (1.22; 1.83)	1.2×10^{-4}	0.799	6	IVW	
		AF (OR)	0.97 (0.92; 1.01)	1.6×10^{-1}	0.314	8	IVW	
		CHD (OR)	0.98 (0.91; 1.06)	6.1×10^{-1}	0.074	8	IVW	
		cIMT (mm)	-0.00 (-0.01; 0.01)	9.4×10^{-1}	0.509	7	IVW	
		Carotid plaque (OR)	0.98 (0.81; 1.19)	8.4×10^{-1}	0.091	7	IVW	
		Any stroke (OR)	0.93 (0.84; 1.02)	1.2×10^{-1}	0.101	7	IVW	
		Any ischemic stroke (OR)	0.91 (0.82; 1.01)	8.1×10^{-2}	0.074	7	IVW	
		LDL cholesterol (mmol/l)	0.01 (0.00; 0.02)	6.3×10^{-3}	0.300	9	IVW	
		Apoliprotein B (g/l)	0.00 (0.00; 0.01)	4.4×10^{-2}	0.722	9	IVW	
		Triglycerides (mmol/l)	-0.00 (-0.02; 0.01)	8.8×10^{-1}	0.124	8	IVW	
		Cholesterol (mmol/l)	0.01 (-0.00; 0.02)	1.0×10^{-1}	0.337	9	IVW	
		HDL cholesterol (mmol/l)	-0.00 (-0.01; 0.00)	1.7×10^{-1}	0.039	9	IVW	
		Apoliprotein A1 (g/l)	-0.00 (-0.00; 0.00)	7.6×10^{-1}	0.323	9	IVW	
		Glucose (mmol/l)	-0.01 (-0.02; 0.00)	1.3×10^{-1}	0.431	9	IVW	
		Glycated haemoglobin (mmol/mol)	-0.05 (-0.12; 0.03)	2.3×10^{-1}	0.345	9	IVW	
		T2DM (OR)	1.19 (1.13; 1.25)	5.0×10^{-13}	0.392	5	IVW	
		BMI (SD)	-0.05 (-0.12; 0.02)	2.0×10^{-1}	0.226	7	MR Egger	

Table S16: Phenome-wide drug target MR scan of proteins with a prioritized CMR association. *(continued)*

Protein (uniprot)	Protein set	Trait	Estimate (95%CI)	P-value	Q p-value	No. variants	MR model	pQTL source
		SBP (mmHg)	0.35 (-0.01; 0.71)	5.7×10^{-2}	0.041	3	IVW	
		DBP (mmHg)	0.04 (-0.27; 0.35)	8.0×10^{-1}	0.110	3	IVW	
		ECG heart rate (exercise) (BPM)	-0.19 (-0.59; 0.21)	3.6×10^{-1}	0.478	9	IVW	
		ECG load (exercise) (Watts)	0.86 (0.41; 1.31)	2.0×10^{-4}	0.946	9	IVW	
		Asthma (OR)	0.91 (0.76; 1.09)	3.0×10^{-1}	0.003	8	MR Egger	
		CRP (log(mg/L))	-0.02 (-0.05; 0.02)	3.5×10^{-1}	0.871	6	IVW	
		eGFR (SD of log(eGFR))	-0.00 (-0.01; -0.00)	1.6×10^{-2}	0.079	6	IVW	
		BUN (mg/dl)	0.01 (0.00; 0.02)	3.0×10^{-2}	0.055	6	IVW	
		CKD (OR)	1.06 (0.98; 1.14)	1.3×10^{-1}	0.290	6	IVW	
		Alzheimer’s (OR)	1.00 (0.98; 1.01)	4.7×10^{-1}	0.809	8	IVW	
		Parkinson’s (OR)	1.33 (1.16; 1.51)	2.6×10^{-5}	0.573	6	IVW	
		Lewy body dementia (OR)	1.03 (0.76; 1.39)	8.7×10^{-1}	0.950	4	IVW	
		Breast cancer (OR)	0.74 (0.50; 1.10)	1.4×10^{-1}	0.775	7	MR Egger	
		Lung cancer (OR)	2.18 (0.80; 5.92)	1.3×10^{-1}	0.430	7	MR Egger	
		Colon cancer (OR)	0.99 (0.85; 1.16)	9.3×10^{-1}	0.695	7	IVW	
		Prostate cancer (OR)	1.08 (0.98; 1.20)	1.3×10^{-1}	0.927	7	IVW	
I17RA (Q96F46)	Drugged	HF (OR)	0.97 (0.96; 0.98)	5.1×10^{-8}	0.003	33	MR Egger	Interval
		Non-ischemic CM (OR)	0.94 (0.92; 0.96)	5.0×10^{-7}	0.239	44	IVW	
		DCM (OR)	0.93 (0.89; 0.96)	4.3×10^{-5}	<0.001	35	IVW	
		AF (OR)	1.00 (1.00; 1.01)	2.1×10^{-1}	<0.001	41	IVW	
		CHD (OR)	1.00 (0.99; 1.00)	4.3×10^{-1}	0.010	38	IVW	
		cIMT (mm)	-0.00 (-0.00; 0.00)	2.5×10^{-1}	0.011	37	IVW	
		Carotid plaque (OR)	1.02 (0.96; 1.07)	5.9×10^{-1}	0.035	23	MR Egger	
		Any stroke (OR)	1.00 (0.99; 1.01)	9.0×10^{-1}	0.076	38	IVW	
		Any ischemic stroke (OR)	1.01 (1.00; 1.01)	8.9×10^{-2}	0.031	39	IVW	
		LDL cholesterol (mmol/l)	0.00 (0.00; 0.01)	3.6×10^{-3}	0.360	50	MR Egger	
		Apoliprotein B (g/l)	0.00 (-0.00; 0.00)	5.9×10^{-2}	0.052	48	MR Egger	
		Triglycerides (mmol/l)	0.01 (0.00; 0.01)	3.4×10^{-3}	<0.001	38	MR Egger	
		Cholesterol (mmol/l)	0.00 (0.00; 0.00)	3.7×10^{-2}	0.075	48	IVW	
		HDL cholesterol (mmol/l)	0.00 (0.00; 0.00)	3.0×10^{-2}	<0.001	45	MR Egger	
		Apoliprotein A1 (g/l)	0.00 (0.00; 0.00)	1.8×10^{-2}	0.003	47	IVW	
		Glucose (mmol/l)	0.00 (-0.00; 0.01)	7.9×10^{-1}	0.194	44	MR Egger	
		Glycated haemoglobin (mmol/mol)	0.02 (0.01; 0.03)	2.4×10^{-3}	<0.001	41	IVW	
		T2DM (OR)	1.00 (1.00; 1.01)	1.2×10^{-1}	0.305	36	IVW	
		BMI (SD)	-0.00 (-0.00; -0.00)	2.2×10^{-2}	<0.001	42	MR Egger	
		SBP (mmHg)	0.06 (0.02; 0.10)	5.6×10^{-3}	0.015	29	MR Egger	
		DBP (mmHg)	0.03 (0.02; 0.04)	6.5×10^{-5}	0.010	33	IVW	
		ECG heart rate (exercise) (BPM)	-0.06 (-0.19; 0.06)	3.4×10^{-1}	0.140	49	MR Egger	
		ECG load (exercise) (Watts)	-0.17 (-0.31; -0.03)	1.6×10^{-2}	0.224	47	MR Egger	
		Asthma (OR)	1.01 (1.00; 1.01)	6.1×10^{-2}	<0.001	40	IVW	
		CRP (log(mg/L))	-0.01 (-0.01; -0.00)	1.4×10^{-6}	0.006	38	IVW	
		eGFR (SD of log(eGFR))	0.00 (0.00; 0.00)	1.6×10^{-8}	0.102	44	MR Egger	
		BUN (mg/dl)	-0.00 (-0.00; -0.00)	5.6×10^{-8}	0.044	41	IVW	
		CKD (OR)	0.96 (0.95; 0.97)	1.2×10^{-10}	<0.001	40	MR Egger	
		Alzheimer’s (OR)	1.00 (0.99; 1.00)	4.5×10^{-4}	0.003	44	MR Egger	
		Parkinson’s (OR)	0.99 (0.97; 1.00)	6.8×10^{-2}	0.081	43	IVW	
		Lewy body dementia (OR)	None	None	None	None	None	
		Breast cancer (OR)	1.03 (1.02; 1.04)	7.0×10^{-7}	<0.001	35	IVW	
		Lung cancer (OR)	0.93 (0.90; 0.97)	4.5×10^{-4}	<0.001	38	MR Egger	
		Colon cancer (OR)	1.06 (1.04; 1.08)	2.8×10^{-9}	<0.001	41	IVW	
		Prostate cancer (OR)	0.99 (0.98; 1.00)	7.0×10^{-3}	0.644	40	IVW	
IL12B (P29460)	Drugged	HF (OR)	0.96 (0.91; 1.01)	1.2×10^{-1}	0.166	14	MR Egger	Interval
		Non-ischemic CM (OR)	1.01 (0.77; 1.33)	9.4×10^{-1}	0.011	15	MR Egger	
		DCM (OR)	0.82 (0.74; 0.90)	5.8×10^{-5}	0.709	15	IVW	
		AF (OR)	0.95 (0.90; 1.01)	1.1×10^{-1}	0.438	15	MR Egger	
		CHD (OR)	0.99 (0.97; 1.01)	4.3×10^{-1}	0.326	14	IVW	
		cIMT (mm)	-0.00 (-0.01; 0.00)	1.6×10^{-1}	0.376	15	MR Egger	
		Carotid plaque (OR)	0.86 (0.82; 0.91)	3.5×10^{-8}	0.047	12	IVW	
		Any stroke (OR)	0.97 (0.92; 1.02)	2.8×10^{-1}	0.431	15	MR Egger	
		Any ischemic stroke (OR)	0.94 (0.88; 1.00)	4.1×10^{-2}	0.260	15	MR Egger	
		LDL cholesterol (mmol/l)	-0.00 (-0.01; 0.00)	1.3×10^{-1}	0.013	14	IVW	
		Apoliprotein B (g/l)	-0.00 (-0.00; -0.00)	4.5×10^{-2}	0.007	14	IVW	
		Triglycerides (mmol/l)	-0.03 (-0.05; -0.01)	2.9×10^{-4}	0.019	16	MR Egger	
		Cholesterol (mmol/l)	0.00 (-0.01; 0.01)	5.5×10^{-1}	<0.001	15	IVW	
		HDL cholesterol (mmol/l)	0.00 (0.00; 0.01)	4.0×10^{-2}	0.385	12	IVW	
		Apoliprotein A1 (g/l)	0.00 (-0.00; 0.00)	6.1×10^{-1}	0.310	12	IVW	

Table S16: Phenome-wide drug target MR scan of proteins with a prioritized CMR association. *(continued)*

Protein (uniprot)	Protein set	Trait	Estimate (95%CI)	P-value	Q p-value	No. variants	MR model	pQTL source
		Glucose (mmol/l)	-0.00 (-0.02; 0.02)	8.5×10^{-1}	0.430	16	MR Egger	
		Glycated haemoglobin (mmol/mol)	0.01 (-0.03; 0.05)	6.7×10^{-1}	0.839	14	IVW	
		T2DM (OR)	0.97 (0.95; 0.99)	1.2×10^{-3}	0.676	15	IVW	
		BMI (SD)	-0.04 (-0.05; -0.03)	1.0×10^{-100}	0.004	11	IVW	
		SBP (mmHg)	0.04 (-0.16; 0.24)	7.1×10^{-1}	0.022	13	MR Egger	
		DBP (mmHg)	0.08 (-0.03; 0.18)	1.4×10^{-1}	0.005	14	MR Egger	
		ECG heart rate (exercise) (BPM)	-0.39 (-0.70; -0.08)	1.3×10^{-2}	0.100	14	IVW	
		ECG load (exercise) (Watts)	0.42 (0.16; 0.69)	1.8×10^{-3}	0.398	16	IVW	
		Asthma (OR)	None	None	None	None	None	
		CRP (log(mg/L))	0.05 (0.02; 0.07)	4.3×10^{-4}	0.262	15	MR Egger	
		eGFR (SD of log(eGFR))	-0.01 (-0.01; -0.00)	1.2×10^{-3}	0.134	15	MR Egger	
		BUN (mg/dl)	0.02 (0.01; 0.03)	4.5×10^{-5}	0.785	14	MR Egger	
		CKD (OR)	1.03 (0.97; 1.08)	3.4×10^{-1}	0.617	15	MR Egger	
		Alzheimer's (OR)	1.00 (0.99; 1.00)	3.8×10^{-1}	0.014	14	IVW	
		Parkinson's (OR)	0.92 (0.77; 1.09)	3.2×10^{-1}	0.991	14	MR Egger	
		Lewy body dementia (OR)	0.88 (0.70; 1.10)	2.7×10^{-1}	0.307	15	MR Egger	
		Breast cancer (OR)	1.02 (0.98; 1.05)	3.1×10^{-1}	0.238	14	IVW	
		Lung cancer (OR)	0.95 (0.88; 1.02)	1.3×10^{-1}	0.551	15	IVW	
		Colon cancer (OR)	1.18 (1.10; 1.26)	2.1×10^{-6}	0.170	15	IVW	
		Prostate cancer (OR)	0.97 (0.89; 1.05)	4.6×10^{-1}	0.739	15	MR Egger	
IL6RA (P08887)	Drugged	HF (OR)	0.98 (0.97; 0.99)	6.0×10^{-3}	<0.001	47	MR Egger	Scallop
		Non-ischemic CM (OR)	1.06 (1.03; 1.10)	1.0×10^{-3}	0.006	54	IVW	
		DCM (OR)	1.01 (0.95; 1.07)	8.0×10^{-1}	<0.001	62	MR Egger	
		AF (OR)	0.95 (0.94; 0.96)	1.0×10^{-100}	0.003	60	MR Egger	
		CHD (OR)	0.94 (0.93; 0.94)	1.0×10^{-100}	<0.001	52	IVW	
		cIMT (mm)	-0.00 (-0.00; 0.00)	6.6×10^{-1}	0.197	53	IVW	
		Carotid plaque (OR)	1.01 (0.96; 1.05)	8.1×10^{-1}	0.194	42	MR Egger	
		Any stroke (OR)	0.98 (0.96; 1.00)	2.9×10^{-2}	0.007	54	MR Egger	
		Any ischemic stroke (OR)	0.97 (0.94; 0.99)	1.7×10^{-2}	0.159	52	MR Egger	
		LDL cholesterol (mmol/l)	0.01 (0.00; 0.01)	1.6×10^{-7}	0.033	62	MR Egger	
		Apoliprotein B (g/l)	0.00 (-0.00; 0.00)	2.4×10^{-1}	<0.001	64	IVW	
		Triglycerides (mmol/l)	0.01 (0.00; 0.01)	1.1×10^{-6}	0.039	63	MR Egger	
		Cholesterol (mmol/l)	0.01 (0.01; 0.02)	3.5×10^{-11}	0.073	60	MR Egger	
		HDL cholesterol (mmol/l)	0.00 (0.00; 0.00)	2.8×10^{-14}	<0.001	58	IVW	
		Apoliprotein A1 (g/l)	0.00 (0.00; 0.00)	4.6×10^{-9}	<0.001	52	MR Egger	
		Glucose (mmol/l)	-0.00 (-0.01; 0.00)	2.9×10^{-1}	0.003	62	MR Egger	
		Glycated haemoglobin (mmol/mol)	-0.08 (-0.10; -0.07)	1.0×10^{-100}	<0.001	53	IVW	
		T2DM (OR)	0.92 (0.87; 0.98)	8.9×10^{-3}	0.216	17	MR Egger	
		BMI (SD)	-0.03 (-0.05; -0.02)	8.4×10^{-5}	0.445	17	MR Egger	
		SBP (mmHg)	-0.03 (-0.14; 0.09)	6.7×10^{-1}	0.067	34	MR Egger	
		DBP (mmHg)	-0.09 (-0.14; -0.05)	1.1×10^{-5}	0.095	28	IVW	
		ECG heart rate (exercise) (BPM)	-0.14 (-0.27; -0.02)	2.1×10^{-2}	<0.001	67	MR Egger	
		ECG load (exercise) (Watts)	-0.26 (-0.40; -0.11)	5.1×10^{-4}	0.125	67	MR Egger	
		Asthma (OR)	None	None	None	None	None	
		CRP (log(mg/L))	-0.15 (-0.20; -0.11)	6.4×10^{-12}	<0.001	11	MR Egger	
		eGFR (SD of log(eGFR))	0.00 (-0.00; 0.00)	1.1×10^{-1}	0.011	35	IVW	
		BUN (mg/dl)	0.00 (-0.00; 0.00)	1.9×10^{-1}	0.382	49	MR Egger	
		CKD (OR)	1.00 (0.98; 1.01)	6.6×10^{-1}	<0.001	55	MR Egger	
		Alzheimer's (OR)	1.01 (1.00; 1.01)	3.6×10^{-3}	0.015	43	MR Egger	
		Parkinson's (OR)	0.95 (0.90; 1.00)	5.2×10^{-2}	0.005	51	MR Egger	
		Lewy body dementia (OR)	1.09 (1.03; 1.14)	1.8×10^{-3}	0.266	63	MR Egger	
		Breast cancer (OR)	1.01 (0.98; 1.04)	5.0×10^{-1}	0.134	58	MR Egger	
		Lung cancer (OR)	1.01 (0.99; 1.03)	2.3×10^{-1}	0.007	61	IVW	
		Colon cancer (OR)	0.97 (0.96; 0.99)	1.4×10^{-3}	0.008	64	IVW	
		Prostate cancer (OR)	1.07 (1.04; 1.09)	4.6×10^{-7}	0.002	62	MR Egger	
LAMC2 (Q13753)	Drugged	HF (OR)	1.02 (1.01; 1.04)	4.2×10^{-3}	0.443	27	IVW	Interval
		Non-ischemic CM (OR)	0.95 (0.84; 1.07)	4.0×10^{-1}	0.064	28	MR Egger	
		DCM (OR)	1.20 (1.13; 1.28)	1.4×10^{-8}	0.387	27	IVW	
		AF (OR)	0.96 (0.94; 0.99)	3.2×10^{-3}	0.060	27	MR Egger	
		CHD (OR)	1.05 (1.02; 1.08)	1.1×10^{-3}	0.445	21	MR Egger	
		cIMT (mm)	0.00 (-0.00; 0.00)	6.6×10^{-1}	0.010	27	IVW	
		Carotid plaque (OR)	0.93 (0.90; 0.96)	4.8×10^{-7}	0.039	24	IVW	
		Any stroke (OR)	1.00 (0.97; 1.03)	9.9×10^{-1}	<0.001	25	MR Egger	
		Any ischemic stroke (OR)	1.01 (0.98; 1.04)	5.5×10^{-1}	0.002	27	MR Egger	
		LDL cholesterol (mmol/l)	-0.01 (-0.02; -0.01)	1.0×10^{-5}	0.002	34	MR Egger	
		Apoliprotein B (g/l)	-0.00 (-0.00; -0.00)	6.7×10^{-4}	0.127	34	MR Egger	

Table S16: Phenome-wide drug target MR scan of proteins with a prioritized CMR association. *(continued)*

Protein (uniprot)	Protein set	Trait	Estimate (95%CI)	P-value	Q p-value	No. variants	MR model	pQTL source
		Triglycerides (mmol/l)	0.02 (0.01; 0.02)	3.8×10^{-15}	0.008	31	IVW	
		Cholesterol (mmol/l)	-0.02 (-0.03; -0.01)	5.5×10^{-7}	<0.001	33	MR Egger	
		HDL cholesterol (mmol/l)	-0.00 (-0.01; -0.00)	1.2×10^{-3}	<0.001	23	IVW	
		Apoliprotein A1 (g/l)	-0.00 (-0.01; -0.00)	3.0×10^{-7}	<0.001	23	IVW	
		Glucose (mmol/l)	-0.03 (-0.04; -0.02)	7.5×10^{-8}	0.139	34	MR Egger	
		Glycated haemoglobin (mmol/mol)	-0.04 (-0.09; -0.00)	4.2×10^{-2}	0.003	33	MR Egger	
		T2DM (OR)	1.01 (0.99; 1.02)	4.7×10^{-1}	0.072	24	IVW	
		BMI (SD)	-0.00 (-0.00; 0.00)	4.6×10^{-1}	0.002	25	IVW	
		SBP (mmHg)	0.17 (0.07; 0.27)	1.0×10^{-3}	0.035	23	MR Egger	
		DBP (mmHg)	0.14 (0.09; 0.20)	5.3×10^{-7}	0.460	24	MR Egger	
		ECG heart rate (exercise) (BPM)	0.41 (0.26; 0.55)	5.3×10^{-8}	0.046	32	IVW	
		ECG load (exercise) (Watts)	-0.06 (-0.41; 0.28)	7.2×10^{-1}	0.064	32	MR Egger	
		Asthma (OR)	0.98 (0.97; 0.99)	4.4×10^{-5}	0.026	31	IVW	
		CRP (log(mg/L))	0.01 (0.00; 0.02)	2.7×10^{-2}	0.143	25	IVW	
		eGFR (SD of log(eGFR))	0.00 (0.00; 0.00)	1.2×10^{-2}	0.013	25	MR Egger	
		BUN (mg/dl)	0.00 (-0.00; 0.00)	3.9×10^{-1}	0.159	27	MR Egger	
		CKD (OR)	1.05 (1.03; 1.07)	2.0×10^{-7}	0.167	27	IVW	
		Alzheimer's (OR)	1.00 (0.99; 1.00)	3.2×10^{-1}	0.110	27	IVW	
		Parkinson's (OR)	1.03 (0.99; 1.06)	1.2×10^{-1}	<0.001	22	IVW	
		Lewy body dementia (OR)	0.82 (0.77; 0.89)	3.6×10^{-7}	0.101	27	IVW	
		Breast cancer (OR)	1.05 (1.02; 1.09)	3.4×10^{-3}	<0.001	19	IVW	
		Lung cancer (OR)	0.96 (0.91; 1.02)	1.9×10^{-1}	<0.001	27	IVW	
		Colon cancer (OR)	0.98 (0.91; 1.06)	6.7×10^{-1}	0.046	28	MR Egger	
		Prostate cancer (OR)	1.05 (1.01; 1.10)	2.5×10^{-2}	0.517	29	MR Egger	
PTGDS (P41222)	Drugged	HF (OR)	1.09 (1.02; 1.17)	1.6×10^{-2}	0.816	4	IVW	Interval
		Non-ischemic CM (OR)	1.30 (1.09; 1.55)	4.2×10^{-3}	0.691	5	IVW	
		DCM (OR)	0.96 (0.75; 1.24)	7.8×10^{-1}	0.681	5	IVW	
		AF (OR)	0.96 (0.90; 1.02)	1.5×10^{-1}	0.370	5	IVW	
		CHD (OR)	1.07 (1.01; 1.12)	1.3×10^{-2}	0.648	5	IVW	
		cIMT (mm)	-0.00 (-0.01; 0.00)	5.2×10^{-1}	0.886	5	IVW	
		Carotid plaque (OR)	0.98 (0.86; 1.11)	7.2×10^{-1}	0.827	5	IVW	
		Any stroke (OR)	0.92 (0.86; 0.99)	2.0×10^{-2}	0.739	5	IVW	
		Any ischemic stroke (OR)	0.96 (0.89; 1.03)	2.7×10^{-1}	0.310	5	IVW	
		LDL cholesterol (mmol/l)	-0.01 (-0.02; 0.01)	4.8×10^{-1}	0.588	4	IVW	
		Apoliprotein B (g/l)	-0.00 (-0.00; 0.00)	8.7×10^{-1}	0.790	4	IVW	
		Triglycerides (mmol/l)	-0.01 (-0.03; 0.01)	1.9×10^{-1}	0.555	4	IVW	
		Cholesterol (mmol/l)	-0.00 (-0.02; 0.02)	8.8×10^{-1}	0.403	4	IVW	
		HDL cholesterol (mmol/l)	0.01 (0.00; 0.02)	3.3×10^{-3}	0.558	4	IVW	
		Apoliprotein A1 (g/l)	0.01 (0.00; 0.01)	4.9×10^{-4}	0.940	4	IVW	
		Glucose (mmol/l)	-0.00 (-0.02; 0.02)	8.3×10^{-1}	0.756	4	IVW	
		Glycated haemoglobin (mmol/mol)	0.08 (-0.03; 0.19)	1.5×10^{-1}	0.841	4	IVW	
		T2DM (OR)	1.02 (0.99; 1.06)	1.8×10^{-1}	0.646	5	IVW	
		BMI (SD)	0.00 (-0.01; 0.02)	5.4×10^{-1}	0.070	5	IVW	
		SBP (mmHg)	1.29 (0.61; 1.97)	2.0×10^{-4}	0.055	2	IVW	
		DBP (mmHg)	0.61 (0.24; 0.99)	1.4×10^{-3}	0.064	2	IVW	
		ECG heart rate (exercise) (BPM)	-3.18 (-6.68; 0.32)	7.5×10^{-2}	0.104	5	MR Egger	
		ECG load (exercise) (Watts)	0.23 (-0.48; 0.93)	5.3×10^{-1}	0.613	4	IVW	
		Asthma (OR)	1.01 (0.98; 1.03)	6.3×10^{-1}	0.367	6	IVW	
		CRP (log(mg/L))	-0.02 (-0.04; 0.01)	2.1×10^{-1}	0.863	5	IVW	
		eGFR (SD of log(eGFR))	0.01 (0.00; 0.02)	9.6×10^{-3}	0.309	5	MR Egger	
		BUN (mg/dl)	0.01 (0.01; 0.02)	2.9×10^{-5}	0.317	5	IVW	
		CKD (OR)	0.73 (0.57; 0.95)	1.7×10^{-2}	0.514	5	MR Egger	
		Alzheimer's (OR)	0.99 (0.97; 1.01)	4.0×10^{-1}	0.774	5	IVW	
		Parkinson's (OR)	1.10 (1.00; 1.22)	4.5×10^{-2}	0.469	5	IVW	
		Lewy body dementia (OR)	1.19 (0.98; 1.45)	8.1×10^{-2}	0.890	5	IVW	
		Breast cancer (OR)	1.01 (0.94; 1.08)	8.6×10^{-1}	0.606	4	IVW	
		Lung cancer (OR)	1.04 (0.86; 1.26)	6.6×10^{-1}	0.594	4	IVW	
		Colon cancer (OR)	0.82 (0.71; 0.95)	8.1×10^{-3}	0.030	4	IVW	
		Prostate cancer (OR)	0.95 (0.87; 1.05)	3.2×10^{-1}	0.978	4	IVW	
RENI (P00797)	Drugged	HF (OR)	0.97 (0.91; 1.04)	4.4×10^{-1}	0.306	10	IVW	Scallop
		Non-ischemic CM (OR)	0.84 (0.65; 1.07)	1.6×10^{-1}	0.961	11	IVW	
		DCM (OR)	0.54 (0.36; 0.79)	1.6×10^{-3}	0.735	10	IVW	
		AF (OR)	0.90 (0.75; 1.07)	2.3×10^{-1}	0.251	12	MR Egger	
		CHD (OR)	1.06 (1.00; 1.11)	4.2×10^{-2}	0.791	13	IVW	
		cIMT (mm)	-0.00 (-0.01; 0.00)	7.7×10^{-1}	0.953	11	IVW	
		Carotid plaque (OR)	0.88 (0.74; 1.04)	1.4×10^{-1}	0.864	10	IVW	

Table S16: Phenome-wide drug target MR scan of proteins with a prioritized CMR association. *(continued)*

Protein (uniprot)	Protein set	Trait	Estimate (95%CI)	P-value	Q p-value	No. variants	MR model	pQTL source
		Any stroke (OR)	1.15 (1.05; 1.25)	3.1×10^{-3}	0.127	11	IVW	
		Any ischemic stroke (OR)	1.12 (1.03; 1.22)	5.6×10^{-3}	0.706	11	IVW	
		LDL cholesterol (mmol/l)	-0.01 (-0.05; 0.04)	8.0×10^{-1}	0.009	11	MR Egger	
		Apoliprotein B (g/l)	-0.00 (-0.01; 0.01)	5.8×10^{-1}	0.006	11	MR Egger	
		Triglycerides (mmol/l)	-0.01 (-0.03; 0.00)	1.7×10^{-1}	0.372	12	IVW	
		Cholesterol (mmol/l)	-0.01 (-0.06; 0.04)	7.2×10^{-1}	0.004	11	MR Egger	
		HDL cholesterol (mmol/l)	-0.00 (-0.02; 0.01)	6.1×10^{-1}	0.312	11	MR Egger	
		Apoliprotein A1 (g/l)	0.00 (-0.00; 0.01)	7.4×10^{-1}	0.117	12	IVW	
		Glucose (mmol/l)	-0.04 (-0.11; 0.02)	1.7×10^{-1}	0.244	12	MR Egger	
		Glycated haemoglobin (mmol/mol)	-0.00 (-0.12; 0.11)	9.4×10^{-1}	0.275	12	IVW	
		T2DM (OR)	0.97 (0.92; 1.03)	3.1×10^{-1}	0.192	11	IVW	
		BMI (SD)	0.01 (-0.03; 0.06)	5.7×10^{-1}	0.236	10	MR Egger	
		SBP (mmHg)	-0.43 (-0.73; -0.13)	4.5×10^{-3}	<0.001	8	IVW	
		DBP (mmHg)	-0.05 (-0.23; 0.12)	5.3×10^{-1}	<0.001	6	IVW	
		ECG heart rate (exercise) (BPM)	0.50 (-0.07; 1.08)	8.8×10^{-2}	0.624	12	IVW	
		ECG load (exercise) (Watts)	1.30 (0.65; 1.94)	8.6×10^{-5}	0.622	12	IVW	
		Asthma (OR)	0.96 (0.85; 1.07)	4.4×10^{-1}	0.561	12	MR Egger	
		CRP (log(mg/L))	0.05 (0.02; 0.09)	1.9×10^{-3}	0.483	11	IVW	
		eGFR (SD of log(eGFR))	-0.00 (-0.01; 0.00)	3.4×10^{-1}	0.188	11	MR Egger	
		BUN (mg/dl)	0.03 (0.02; 0.04)	5.8×10^{-12}	0.133	10	IVW	
		CKD (OR)	1.12 (1.04; 1.20)	1.9×10^{-3}	0.331	11	IVW	
		Alzheimer's (OR)	1.03 (1.01; 1.04)	3.9×10^{-4}	0.905	11	IVW	
		Parkinson's (OR)	0.90 (0.76; 1.06)	2.1×10^{-1}	0.563	11	IVW	
		Lewy body dementia (OR)	0.66 (0.47; 0.92)	1.5×10^{-2}	0.976	11	IVW	
		Breast cancer (OR)	1.06 (0.97; 1.15)	1.9×10^{-1}	0.315	12	IVW	
		Lung cancer (OR)	1.01 (0.55; 1.83)	9.8×10^{-1}	0.736	12	MR Egger	
		Colon cancer (OR)	0.84 (0.68; 1.04)	1.1×10^{-1}	0.038	11	IVW	
		Prostate cancer (OR)	0.95 (0.85; 1.06)	3.5×10^{-1}	0.335	12	IVW	
SLAF7 (Q9NQ25)	Drugged	HF (OR)	1.07 (1.05; 1.08)	2.2×10^{-13}	0.215	23	MR Egger	Interval
		Non-ischemic CM (OR)	1.01 (0.96; 1.07)	6.6×10^{-1}	0.065	21	IVW	
		DCM (OR)	1.12 (1.05; 1.19)	3.8×10^{-4}	0.063	20	IVW	
		AF (OR)	0.99 (0.99; 1.00)	5.9×10^{-3}	0.026	27	IVW	
		CHD (OR)	1.02 (1.01; 1.03)	7.0×10^{-4}	0.024	23	IVW	
		cIMT (mm)	-0.00 (-0.00; -0.00)	2.1×10^{-7}	0.013	23	IVW	
		Carotid plaque (OR)	0.95 (0.92; 0.99)	1.2×10^{-2}	0.030	23	MR Egger	
		Any stroke (OR)	1.04 (1.01; 1.06)	1.7×10^{-3}	0.038	19	MR Egger	
		Any ischemic stroke (OR)	1.00 (0.99; 1.01)	6.7×10^{-1}	0.004	21	IVW	
		LDL cholesterol (mmol/l)	0.01 (0.00; 0.01)	1.5×10^{-9}	0.675	25	IVW	
		Apoliprotein B (g/l)	0.00 (0.00; 0.00)	1.4×10^{-11}	0.201	24	IVW	
		Triglycerides (mmol/l)	0.01 (0.00; 0.01)	5.3×10^{-3}	0.010	26	MR Egger	
		Cholesterol (mmol/l)	0.01 (0.00; 0.01)	7.4×10^{-8}	0.082	27	IVW	
		HDL cholesterol (mmol/l)	-0.00 (-0.00; 0.00)	9.1×10^{-2}	0.124	28	MR Egger	
		Apoliprotein A1 (g/l)	-0.00 (-0.00; -0.00)	1.9×10^{-2}	0.145	26	MR Egger	
		Glucose (mmol/l)	-0.01 (-0.01; -0.00)	2.2×10^{-3}	0.025	25	IVW	
		Glycated haemoglobin (mmol/mol)	0.02 (0.00; 0.03)	3.2×10^{-2}	0.208	26	IVW	
		T2DM (OR)	0.99 (0.99; 1.00)	1.3×10^{-2}	0.047	22	IVW	
		BMI (SD)	0.00 (0.00; 0.00)	1.5×10^{-2}	0.043	21	IVW	
		SBP (mmHg)	-0.02 (-0.08; 0.04)	4.3×10^{-1}	0.554	20	MR Egger	
		DBP (mmHg)	0.01 (-0.01; 0.04)	3.8×10^{-1}	0.052	17	IVW	
		ECG heart rate (exercise) (BPM)	0.00 (-0.09; 0.09)	9.8×10^{-1}	<0.001	25	IVW	
		ECG load (exercise) (Watts)	0.51 (0.41; 0.60)	1.0×10^{-100}	0.002	27	IVW	
		Asthma (OR)	1.01 (1.00; 1.01)	9.7×10^{-3}	0.008	27	IVW	
		CRP (log(mg/L))	0.01 (-0.01; 0.02)	2.6×10^{-1}	0.008	15	MR Egger	
		eGFR (SD of log(eGFR))	-0.00 (-0.00; -0.00)	3.5×10^{-3}	0.342	19	MR Egger	
		BUN (mg/dl)	-0.00 (-0.00; 0.00)	7.2×10^{-1}	0.122	20	IVW	
		CKD (OR)	1.02 (0.99; 1.05)	2.6×10^{-1}	0.332	21	MR Egger	
		Alzheimer's (OR)	0.99 (0.99; 1.00)	9.0×10^{-7}	0.150	24	IVW	
		Parkinson's (OR)	1.02 (0.98; 1.07)	3.0×10^{-1}	0.474	23	MR Egger	
		Lewy body dementia (OR)	1.20 (1.09; 1.33)	2.7×10^{-4}	0.216	25	MR Egger	
		Breast cancer (OR)	1.02 (0.99; 1.05)	1.2×10^{-1}	0.005	27	MR Egger	
		Lung cancer (OR)	0.99 (0.89; 1.10)	8.1×10^{-1}	0.003	25	MR Egger	
		Colon cancer (OR)	0.99 (0.97; 1.02)	6.5×10^{-1}	<0.001	24	IVW	
		Prostate cancer (OR)	1.03 (1.02; 1.04)	2.8×10^{-6}	0.036	28	IVW	
TIE2 (Q02763)	Drugged	HF (OR)	0.98 (0.96; 0.99)	2.4×10^{-3}	0.002	55	IVW	Scallop
		Non-ischemic CM (OR)	1.28 (1.12; 1.46)	2.4×10^{-4}	<0.001	50	MR Egger	
		DCM (OR)	1.16 (1.01; 1.33)	3.3×10^{-2}	0.019	55	MR Egger	

Table S16: Phenome-wide drug target MR scan of proteins with a prioritized CMR association. *(continued)*

Protein (uniprot)	Protein set	Trait	Estimate (95%CI)	P-value	Q p-value	No. variants	MR model	pQTL source
		AF (OR)	1.03 (1.00; 1.05)	2.7×10^{-2}	<0.001	53	MR Egger	
		CHD (OR)	1.10 (1.06; 1.15)	9.9×10^{-6}	<0.001	40	MR Egger	
		cIMT (mm)	-0.01 (-0.01; -0.00)	9.0×10^{-5}	<0.001	48	MR Egger	
		Carotid plaque (OR)	1.14 (1.07; 1.21)	8.5×10^{-5}	<0.001	52	MR Egger	
		Any stroke (OR)	1.04 (1.02; 1.07)	5.0×10^{-4}	0.052	49	IVW	
		Any ischemic stroke (OR)	1.03 (1.01; 1.05)	1.5×10^{-2}	<0.001	45	IVW	
		LDL cholesterol (mmol/l)	0.00 (-0.00; 0.01)	1.6×10^{-1}	<0.001	52	IVW	
		Apoliprotein B (g/l)	-0.00 (-0.00; 0.00)	1.3×10^{-1}	0.004	51	MR Egger	
		Triglycerides (mmol/l)	-0.00 (-0.01; 0.01)	6.6×10^{-1}	<0.001	44	MR Egger	
		Cholesterol (mmol/l)	-0.01 (-0.02; -0.01)	5.3×10^{-5}	<0.001	53	MR Egger	
		HDL cholesterol (mmol/l)	-0.01 (-0.01; -0.01)	1.0×10^{-100}	0.017	48	MR Egger	
		Apoliprotein A1 (g/l)	-0.01 (-0.01; -0.00)	3.1×10^{-11}	<0.001	49	MR Egger	
		Glucose (mmol/l)	0.00 (-0.00; 0.01)	1.3×10^{-1}	<0.001	43	IVW	
		Glycated haemoglobin (mmol/mol)	0.13 (0.09; 0.18)	1.6×10^{-8}	<0.001	48	MR Egger	
		T2DM (OR)	1.01 (0.99; 1.02)	4.4×10^{-1}	<0.001	47	IVW	
		BMI (SD)	0.00 (-0.01; 0.01)	5.4×10^{-1}	<0.001	47	MR Egger	
		SBP (mmHg)	0.16 (0.09; 0.24)	3.3×10^{-5}	<0.001	48	IVW	
		DBP (mmHg)	0.15 (0.08; 0.21)	1.6×10^{-5}	0.069	41	IVW	
		ECG heart rate (exercise) (BPM)	-0.37 (-0.51; -0.23)	1.9×10^{-7}	<0.001	51	IVW	
		ECG load (exercise) (Watts)	-0.39 (-0.55; -0.23)	2.3×10^{-6}	0.108	55	IVW	
		Asthma (OR)	None	None	None	None	None	
		CRP (log(mg/L))	-0.02 (-0.03; -0.01)	6.9×10^{-4}	0.196	54	IVW	
		eGFR (SD of log(eGFR))	0.00 (-0.00; 0.00)	2.0×10^{-1}	0.015	52	IVW	
		BUN (mg/dl)	-0.00 (-0.01; 0.00)	3.0×10^{-1}	0.153	51	MR Egger	
		CKD (OR)	0.97 (0.94; 1.01)	1.2×10^{-1}	<0.001	50	MR Egger	
		Alzheimer's (OR)	1.00 (0.99; 1.01)	8.1×10^{-1}	<0.001	44	MR Egger	
		Parkinson's (OR)	1.05 (0.99; 1.11)	8.8×10^{-2}	0.007	45	IVW	
		Lewy body dementia (OR)	0.89 (0.81; 0.97)	9.9×10^{-3}	<0.001	41	IVW	
		Breast cancer (OR)	1.08 (1.03; 1.12)	3.9×10^{-4}	0.376	50	MR Egger	
		Lung cancer (OR)	0.89 (0.83; 0.95)	6.5×10^{-4}	0.336	49	IVW	
		Colon cancer (OR)	0.82 (0.79; 0.86)	1.0×10^{-100}	0.069	49	IVW	
		Prostate cancer (OR)	0.99 (0.97; 1.02)	6.4×10^{-1}	0.033	50	IVW	
VGFR3 (P35916)	Drugged	HF (OR)	1.03 (0.91; 1.17)	6.0×10^{-1}	0.078	18	MR Egger	Interval
		Non-ischemic CM (OR)	1.34 (0.88; 2.04)	1.8×10^{-1}	0.621	18	MR Egger	
		DCM (OR)	0.91 (0.78; 1.06)	2.1×10^{-1}	0.374	18	IVW	
		AF (OR)	1.04 (1.01; 1.06)	3.0×10^{-3}	0.824	18	IVW	
		CHD (OR)	1.00 (0.96; 1.03)	8.2×10^{-1}	0.804	16	IVW	
		cIMT (mm)	-0.01 (-0.03; -0.00)	1.1×10^{-2}	0.956	18	MR Egger	
		Carotid plaque (OR)	0.88 (0.80; 0.96)	3.9×10^{-3}	0.234	14	IVW	
		Any stroke (OR)	1.00 (0.94; 1.05)	9.5×10^{-1}	0.083	15	IVW	
		Any ischemic stroke (OR)	1.02 (0.97; 1.07)	4.3×10^{-1}	0.497	15	IVW	
		LDL cholesterol (mmol/l)	0.00 (-0.01; 0.01)	9.3×10^{-1}	0.008	17	IVW	
		Apoliprotein B (g/l)	0.00 (-0.00; 0.00)	9.2×10^{-1}	0.009	17	IVW	
		Triglycerides (mmol/l)	0.00 (-0.01; 0.01)	6.8×10^{-1}	0.157	18	IVW	
		Cholesterol (mmol/l)	0.01 (-0.00; 0.02)	1.1×10^{-1}	0.005	18	IVW	
		HDL cholesterol (mmol/l)	0.01 (0.00; 0.01)	3.4×10^{-5}	0.320	17	IVW	
		Apoliprotein A1 (g/l)	0.01 (0.00; 0.01)	3.1×10^{-8}	0.048	17	IVW	
		Glucose (mmol/l)	0.00 (-0.01; 0.01)	4.5×10^{-1}	0.015	18	IVW	
		Glycated haemoglobin (mmol/mol)	0.00 (-0.05; 0.05)	9.2×10^{-1}	0.014	18	IVW	
		T2DM (OR)	0.99 (0.97; 1.02)	4.9×10^{-1}	0.445	16	IVW	
		BMI (SD)	0.01 (0.00; 0.02)	2.5×10^{-2}	0.022	15	IVW	
		SBP (mmHg)	0.03 (-0.55; 0.60)	9.2×10^{-1}	0.018	14	MR Egger	
		DBP (mmHg)	-0.24 (-0.32; -0.17)	9.9×10^{-10}	0.003	12	IVW	
		ECG heart rate (exercise) (BPM)	0.16 (-0.14; 0.45)	2.9×10^{-1}	0.580	18	IVW	
		ECG load (exercise) (Watts)	-0.66 (-0.99; -0.33)	8.3×10^{-5}	0.586	18	IVW	
		Asthma (OR)	0.95 (0.88; 1.03)	2.1×10^{-1}	0.559	20	MR Egger	
		CRP (log(mg/L))	-0.02 (-0.04; 0.00)	5.9×10^{-2}	0.443	16	IVW	
		eGFR (SD of log(eGFR))	-0.00 (-0.01; 0.01)	8.0×10^{-1}	0.108	17	MR Egger	
		BUN (mg/dl)	0.00 (-0.00; 0.00)	1.0×10^{0}	0.045	15	IVW	
		CKD (OR)	0.87 (0.84; 0.91)	2.0×10^{-11}	0.042	15	IVW	
		Alzheimer's (OR)	1.00 (0.99; 1.01)	6.2×10^{-1}	0.599	17	IVW	
		Parkinson's (OR)	0.63 (0.48; 0.83)	9.7×10^{-4}	0.733	17	MR Egger	
		Lewy body dementia (OR)	1.03 (0.88; 1.20)	7.2×10^{-1}	0.392	16	IVW	
		Breast cancer (OR)	1.03 (0.98; 1.08)	2.8×10^{-1}	0.763	16	IVW	
		Lung cancer (OR)	1.12 (0.97; 1.30)	1.3×10^{-1}	0.170	16	IVW	
		Colon cancer (OR)	1.06 (0.94; 1.19)	3.8×10^{-1}	0.140	16	IVW	

Table S16: Phenome-wide drug target MR scan of proteins with a prioritized CMR association. *(continued)*

Protein (uniprot)	Protein set	Trait	Estimate (95%CI)	P-value	Q p-value	No. variants	MR model	pQTL source
		Prostate cancer (OR)	0.99 (0.93; 1.06)	7.8×10^{-1}	0.034	16	IVW	
CASP8 (Q14790)	Druggable	HF (OR)	1.14 (1.01; 1.28)	3.1×10^{-2}	0.347	7	IVW	Scallop
		Non-ischemic CM (OR)	0.92 (0.67; 1.25)	5.9×10^{-1}	0.969	7	IVW	
		DCM (OR)	1.05 (0.64; 1.73)	8.5×10^{-1}	0.769	7	IVW	
		AF (OR)	1.02 (0.92; 1.12)	7.5×10^{-1}	0.103	8	IVW	
		CHD (OR)	1.13 (1.03; 1.24)	1.3×10^{-2}	0.550	8	IVW	
		cIMT (mm)	-0.00 (-0.01; 0.00)	3.6×10^{-1}	0.412	7	IVW	
		Carotid plaque (OR)	0.84 (0.68; 1.04)	1.0×10^{-1}	0.373	7	IVW	
		Any stroke (OR)	1.02 (0.89; 1.16)	8.3×10^{-1}	0.255	7	IVW	
		Any ischemic stroke (OR)	1.05 (0.90; 1.22)	5.2×10^{-1}	0.243	7	IVW	
		LDL cholesterol (mmol/l)	0.00 (-0.02; 0.02)	9.5×10^{-1}	0.506	8	IVW	
		Apoliprotein B (g/l)	-0.02 (-0.04; -0.00)	2.4×10^{-2}	0.373	8	MR Egger	
		Triglycerides (mmol/l)	-0.03 (-0.06; 0.01)	1.5×10^{-1}	0.440	5	IVW	
		Cholesterol (mmol/l)	-0.08 (-0.15; -0.00)	4.1×10^{-2}	0.442	8	MR Egger	
		HDL cholesterol (mmol/l)	-0.00 (-0.01; 0.01)	5.6×10^{-1}	0.367	8	IVW	
		Apoliprotein A1 (g/l)	0.00 (-0.00; 0.01)	2.2×10^{-1}	0.653	8	IVW	
		Glucose (mmol/l)	-0.04 (-0.07; -0.01)	1.3×10^{-2}	0.528	8	IVW	
		Glycated haemoglobin (mmol/mol)	0.37 (-0.19; 0.93)	2.0×10^{-1}	0.138	8	MR Egger	
		T2DM (OR)	1.00 (0.93; 1.07)	9.7×10^{-1}	0.216	7	IVW	
		BMI (SD)	0.03 (0.01; 0.04)	2.3×10^{-3}	0.655	7	IVW	
		SBP (mmHg)	-0.42 (-0.70; -0.13)	4.1×10^{-3}	0.973	7	IVW	
		DBP (mmHg)	-0.11 (-0.27; 0.05)	1.9×10^{-1}	0.947	7	IVW	
		ECG heart rate (exercise) (BPM)	0.58 (-0.32; 1.48)	2.1×10^{-1}	0.828	8	IVW	
		ECG load (exercise) (Watts)	-1.08 (-4.17; 2.01)	4.9×10^{-1}	0.295	8	MR Egger	
		Asthma (OR)	1.15 (1.08; 1.22)	5.4×10^{-6}	0.305	8	IVW	
		CRP (log(mg/L))	-0.04 (-0.08; 0.01)	1.4×10^{-1}	0.155	7	IVW	
		eGFR (SD of log(eGFR))	-0.00 (-0.00; 0.00)	3.9×10^{-1}	0.773	8	IVW	
		BUN (mg/dl)	0.01 (-0.00; 0.01)	2.1×10^{-1}	0.685	7	IVW	
		CKD (OR)	1.39 (1.06; 1.83)	1.6×10^{-2}	0.400	8	MR Egger	
		Alzheimer's (OR)	0.96 (0.89; 1.04)	3.2×10^{-1}	0.991	7	MR Egger	
		Parkinson's (OR)	1.03 (0.87; 1.23)	7.2×10^{-1}	0.389	7	IVW	
		Lewy body dementia (OR)	1.05 (0.60; 1.84)	8.5×10^{-1}	0.187	6	IVW	
		Breast cancer (OR)	0.61 (0.53; 0.70)	1.9×10^{-11}	0.789	6	IVW	
		Lung cancer (OR)	0.70 (0.51; 0.95)	2.3×10^{-2}	0.341	9	IVW	
		Colon cancer (OR)	0.81 (0.64; 1.03)	8.1×10^{-2}	0.022	9	IVW	
		Prostate cancer (OR)	0.57 (0.37; 0.86)	7.6×10^{-3}	0.501	9	MR Egger	
DKK1 (O94907)	Druggable	HF (OR)	0.89 (0.83; 0.96)	2.6×10^{-3}	0.100	10	IVW	Scallop
		Non-ischemic CM (OR)	0.79 (0.56; 1.12)	1.8×10^{-1}	0.758	8	IVW	
		DCM (OR)	0.44 (0.30; 0.65)	4.7×10^{-5}	0.974	8	IVW	
		AF (OR)	0.86 (0.80; 0.92)	2.9×10^{-5}	0.160	9	IVW	
		CHD (OR)	0.92 (0.84; 1.01)	7.4×10^{-2}	0.315	8	IVW	
		cIMT (mm)	-0.01 (-0.02; -0.00)	2.6×10^{-2}	0.898	9	IVW	
		Carotid plaque (OR)	1.17 (0.98; 1.39)	7.8×10^{-2}	0.292	9	IVW	
		Any stroke (OR)	0.92 (0.82; 1.02)	1.2×10^{-1}	0.171	9	IVW	
		Any ischemic stroke (OR)	0.95 (0.85; 1.06)	3.5×10^{-1}	0.302	9	IVW	
		LDL cholesterol (mmol/l)	-0.02 (-0.05; -0.00)	3.9×10^{-2}	0.989	7	IVW	
		Apoliprotein B (g/l)	-0.01 (-0.01; -0.00)	4.2×10^{-2}	0.955	7	IVW	
		Triglycerides (mmol/l)	-0.05 (-0.07; -0.02)	4.4×10^{-4}	0.438	7	IVW	
		Cholesterol (mmol/l)	-0.03 (-0.06; -0.00)	3.1×10^{-2}	0.965	7	IVW	
		HDL cholesterol (mmol/l)	0.01 (0.00; 0.02)	2.3×10^{-2}	0.003	7	IVW	
		Apoliprotein A1 (g/l)	0.03 (0.01; 0.04)	6.4×10^{-3}	0.032	8	MR Egger	
		Glucose (mmol/l)	0.01 (-0.03; 0.04)	6.7×10^{-1}	0.850	7	IVW	
		Glycated haemoglobin (mmol/mol)	0.03 (-0.15; 0.22)	7.3×10^{-1}	0.282	7	IVW	
		T2DM (OR)	1.03 (0.96; 1.11)	4.5×10^{-1}	0.297	8	IVW	
		BMI (SD)	-0.03 (-0.05; -0.01)	3.1×10^{-3}	0.674	8	IVW	
		SBP (mmHg)	-0.14 (-0.46; 0.19)	4.1×10^{-1}	0.782	8	IVW	
		DBP (mmHg)	0.17 (-0.38; 0.72)	5.5×10^{-1}	0.550	8	MR Egger	
		ECG heart rate (exercise) (BPM)	-1.53 (-2.47; -0.58)	1.6×10^{-3}	0.999	7	IVW	
		ECG load (exercise) (Watts)	0.09 (-0.98; 1.15)	8.7×10^{-1}	0.752	7	IVW	
		Asthma (OR)	0.98 (0.92; 1.04)	4.9×10^{-1}	0.967	8	IVW	
		CRP (log(mg/L))	0.02 (-0.03; 0.06)	4.6×10^{-1}	0.651	9	IVW	
		eGFR (SD of log(eGFR))	-0.00 (-0.00; 0.00)	4.3×10^{-1}	0.918	8	IVW	
		BUN (mg/dl)	-0.01 (-0.02; 0.01)	3.8×10^{-1}	0.197	8	IVW	
		CKD (OR)	0.96 (0.86; 1.08)	5.2×10^{-1}	0.312	8	IVW	
		Alzheimer's (OR)	0.98 (0.95; 1.01)	1.1×10^{-1}	0.282	9	IVW	
		Parkinson's (OR)	1.04 (0.76; 1.43)	8.1×10^{-1}	0.111	8	IVW	

Table S16: Phenome-wide drug target MR scan of proteins with a prioritized CMR association. *(continued)*

Protein (uniprot)	Protein set	Trait	Estimate (95%CI)	P-value	Q p-value	No. variants	MR model	pQTL source
		Lewy body dementia (OR)	1.26 (0.83; 1.91)	2.9×10^{-1}	0.020	6	IVW	
		Breast cancer (OR)	1.14 (1.03; 1.27)	1.5×10^{-2}	0.806	8	IVW	
		Lung cancer (OR)	0.29 (0.11; 0.75)	1.1×10^{-2}	0.531	8	MR Egger	
		Colon cancer (OR)	1.93 (1.56; 2.41)	3.1×10^{-9}	0.604	8	IVW	
		Prostate cancer (OR)	1.01 (0.83; 1.24)	8.9×10^{-1}	0.058	7	IVW	
ERAP1 (Q9NZ08)	Druggable	HF (OR)	0.99 (0.99; 0.99)	5.7×10^{-5}	<0.001	33	IVW	Interval
		Non-ischemic CM (OR)	1.10 (1.07; 1.13)	3.1×10^{-12}	<0.001	35	IVW	
		DCM (OR)	1.03 (1.01; 1.05)	1.0×10^{-2}	0.066	48	IVW	
		AF (OR)	0.99 (0.98; 0.99)	1.5×10^{-11}	<0.001	46	IVW	
		CHD (OR)	0.98 (0.97; 0.98)	4.4×10^{-16}	0.007	39	IVW	
		cIMT (mm)	0.00 (0.00; 0.00)	1.7×10^{-11}	0.023	38	IVW	
		Carotid plaque (OR)	0.95 (0.94; 0.96)	3.8×10^{-11}	0.007	42	MR Egger	
		Any stroke (OR)	0.97 (0.96; 0.99)	2.0×10^{-4}	0.170	32	MR Egger	
		Any ischemic stroke (OR)	0.99 (0.97; 1.00)	7.9×10^{-2}	0.248	33	MR Egger	
		LDL cholesterol (mmol/l)	0.00 (-0.00; 0.00)	5.6×10^{-1}	<0.001	49	MR Egger	
		Apoliprotein B (g/l)	0.00 (0.00; 0.00)	6.9×10^{-5}	<0.001	48	IVW	
		Triglycerides (mmol/l)	0.00 (0.00; 0.01)	9.9×10^{-9}	<0.001	41	IVW	
		Cholesterol (mmol/l)	0.00 (-0.00; 0.00)	6.6×10^{-1}	<0.001	49	MR Egger	
		HDL cholesterol (mmol/l)	0.00 (-0.00; 0.00)	7.3×10^{-1}	<0.001	40	MR Egger	
		Apoliprotein A1 (g/l)	-0.00 (-0.00; -0.00)	4.3×10^{-5}	<0.001	43	IVW	
		Glucose (mmol/l)	-0.00 (-0.00; 0.00)	6.9×10^{-1}	<0.001	48	IVW	
		Glycated haemoglobin (mmol/mol)	0.04 (0.03; 0.05)	2.3×10^{-12}	0.073	46	IVW	
		T2DM (OR)	0.97 (0.96; 0.98)	1.0×10^{-100}	0.004	39	MR Egger	
		BMI (SD)	-0.01 (-0.01; -0.00)	1.2×10^{-10}	<0.001	35	MR Egger	
		SBP (mmHg)	-0.17 (-0.20; -0.15)	1.0×10^{-100}	<0.001	25	IVW	
		DBP (mmHg)	-0.15 (-0.17; -0.13)	1.0×10^{-100}	<0.001	27	IVW	
		ECG heart rate (exercise) (BPM)	0.27 (0.17; 0.36)	7.4×10^{-8}	0.008	52	MR Egger	
		ECG load (exercise) (Watts)	0.27 (0.21; 0.32)	1.0×10^{-100}	<0.001	48	IVW	
		Asthma (OR)	1.01 (1.01; 1.02)	4.5×10^{-6}	0.011	57	MR Egger	
		CRP (log(mg/L))	0.00 (-0.00; 0.01)	7.4×10^{-2}	<0.001	47	MR Egger	
		eGFR (SD of log(eGFR))	0.00 (-0.00; 0.00)	3.3×10^{-1}	0.053	34	IVW	
		BUN (mg/dl)	-0.00 (-0.00; 0.00)	2.3×10^{-1}	<0.001	38	MR Egger	
		CKD (OR)	1.02 (1.01; 1.04)	3.3×10^{-4}	0.011	40	MR Egger	
		Alzheimer's (OR)	1.00 (1.00; 1.00)	4.0×10^{-1}	0.068	43	MR Egger	
		Parkinson's (OR)	1.00 (0.99; 1.01)	6.5×10^{-1}	<0.001	40	IVW	
		Lewy body dementia (OR)	1.09 (1.06; 1.11)	7.8×10^{-13}	0.014	44	IVW	
		Breast cancer (OR)	1.05 (1.03; 1.07)	9.8×10^{-9}	<0.001	44	MR Egger	
		Lung cancer (OR)	1.00 (0.96; 1.04)	9.6×10^{-1}	0.091	44	MR Egger	
		Colon cancer (OR)	0.96 (0.94; 0.99)	3.6×10^{-3}	<0.001	48	MR Egger	
		Prostate cancer (OR)	1.04 (1.03; 1.05)	1.0×10^{-100}	<0.001	46	IVW	
ERAP2 (Q6P179)	Druggable	HF (OR)	0.99 (0.99; 1.00)	1.0×10^{-2}	<0.001	32	IVW	Interval
		Non-ischemic CM (OR)	0.95 (0.92; 0.97)	2.4×10^{-4}	0.092	39	IVW	
		DCM (OR)	0.95 (0.90; 0.99)	2.9×10^{-2}	0.129	46	MR Egger	
		AF (OR)	0.98 (0.97; 0.99)	1.5×10^{-3}	0.008	40	MR Egger	
		CHD (OR)	1.03 (1.02; 1.03)	1.0×10^{-100}	<0.001	39	IVW	
		cIMT (mm)	-0.00 (-0.00; -0.00)	3.8×10^{-2}	<0.001	38	MR Egger	
		Carotid plaque (OR)	1.04 (1.03; 1.06)	8.0×10^{-7}	0.050	39	MR Egger	
		Any stroke (OR)	1.00 (0.99; 1.01)	8.2×10^{-1}	0.348	44	MR Egger	
		Any ischemic stroke (OR)	0.99 (0.98; 1.00)	1.2×10^{-1}	0.254	44	MR Egger	
		LDL cholesterol (mmol/l)	0.01 (0.00; 0.01)	6.6×10^{-7}	0.541	46	MR Egger	
		Apoliprotein B (g/l)	0.00 (0.00; 0.00)	3.2×10^{-8}	0.051	46	MR Egger	
		Triglycerides (mmol/l)	0.00 (-0.00; 0.01)	9.3×10^{-2}	<0.001	48	MR Egger	
		Cholesterol (mmol/l)	0.01 (0.00; 0.01)	3.8×10^{-5}	0.211	47	MR Egger	
		HDL cholesterol (mmol/l)	-0.00 (-0.00; 0.00)	4.7×10^{-1}	<0.001	37	IVW	
		Apoliprotein A1 (g/l)	-0.00 (-0.00; 0.00)	8.0×10^{-1}	<0.001	39	IVW	
		Glucose (mmol/l)	-0.01 (-0.01; -0.01)	1.0×10^{-100}	<0.001	45	IVW	
		Glycated haemoglobin (mmol/mol)	-0.00 (-0.01; 0.01)	7.2×10^{-1}	<0.001	45	IVW	
		T2DM (OR)	1.01 (1.01; 1.02)	7.5×10^{-5}	0.066	40	IVW	
		BMI (SD)	0.01 (0.01; 0.01)	6.1×10^{-6}	0.081	30	MR Egger	
		SBP (mmHg)	0.15 (0.07; 0.22)	1.5×10^{-4}	<0.001	21	MR Egger	
		DBP (mmHg)	0.13 (0.10; 0.17)	1.6×10^{-14}	<0.001	26	MR Egger	
		ECG heart rate (exercise) (BPM)	0.31 (0.22; 0.40)	1.2×10^{-12}	<0.001	49	MR Egger	
		ECG load (exercise) (Watts)	-0.09 (-0.14; -0.05)	1.6×10^{-5}	<0.001	51	IVW	
		Asthma (OR)	1.00 (0.99; 1.00)	2.8×10^{-1}	0.032	43	MR Egger	
		CRP (log(mg/L))	-0.00 (-0.01; 0.01)	9.4×10^{-1}	0.002	39	MR Egger	
		eGFR (SD of log(eGFR))	-0.00 (-0.00; -0.00)	5.7×10^{-9}	0.024	40	IVW	

Table S16: Phenome-wide drug target MR scan of proteins with a prioritized CMR association. *(continued)*

Protein (uniprot)	Protein set	Trait	Estimate (95%CI)	P-value	Q p-value	No. variants	MR model	pQTL source
		BUN (mg/dl)	-0.00 (-0.00; 0.00)	8.9×10^{-1}	0.476	42	MR Egger	
		CKD (OR)	0.99 (0.98; 1.00)	1.5×10^{-1}	0.070	43	MR Egger	
		Alzheimer’s (OR)	1.00 (1.00; 1.00)	2.9×10^{-2}	<0.001	45	IVW	
		Parkinson’s (OR)	0.96 (0.94; 0.99)	1.7×10^{-3}	0.010	45	MR Egger	
		Lewy body dementia (OR)	0.97 (0.96; 0.99)	5.7×10^{-4}	0.008	40	IVW	
		Breast cancer (OR)	0.99 (0.99; 1.00)	9.6×10^{-2}	<0.001	39	IVW	
		Lung cancer (OR)	0.94 (0.93; 0.95)	1.0×10^{-100}	0.026	41	IVW	
		Colon cancer (OR)	1.02 (0.98; 1.06)	2.4×10^{-1}	0.756	42	MR Egger	
		Prostate cancer (OR)	0.95 (0.95; 0.96)	1.0×10^{-100}	<0.001	36	IVW	
FCER2 (P06734)	Druggable	HF (OR)	0.94 (0.90; 1.00)	3.5×10^{-2}	0.523	22	MR Egger	Interval
		Non-ischemic CM (OR)	0.93 (0.73; 1.19)	5.7×10^{-1}	0.043	21	MR Egger	
		DCM (OR)	1.17 (0.88; 1.55)	2.9×10^{-1}	0.779	21	MR Egger	
		AF (OR)	1.03 (1.01; 1.05)	8.5×10^{-3}	0.207	23	IVW	
		CHD (OR)	1.01 (0.96; 1.06)	7.1×10^{-1}	0.265	23	MR Egger	
		cIMT (mm)	0.00 (-0.00; 0.01)	6.9×10^{-1}	0.154	22	MR Egger	
		Carotid plaque (OR)	0.96 (0.91; 1.00)	5.6×10^{-2}	0.007	20	IVW	
		Any stroke (OR)	0.96 (0.91; 1.02)	1.5×10^{-1}	0.710	22	MR Egger	
		Any ischemic stroke (OR)	0.95 (0.88; 1.02)	1.6×10^{-1}	0.114	23	MR Egger	
		LDL cholesterol (mmol/l)	-0.00 (-0.02; 0.01)	9.3×10^{-1}	0.300	25	MR Egger	
		Apoliprotein B (g/l)	0.00 (-0.00; 0.00)	9.5×10^{-1}	0.394	25	MR Egger	
		Triglycerides (mmol/l)	-0.00 (-0.02; 0.02)	9.8×10^{-1}	0.098	25	MR Egger	
		Cholesterol (mmol/l)	0.00 (-0.02; 0.02)	9.6×10^{-1}	0.332	25	MR Egger	
		HDL cholesterol (mmol/l)	0.00 (-0.01; 0.01)	7.4×10^{-1}	0.038	25	MR Egger	
		Apoliprotein A1 (g/l)	0.00 (-0.00; 0.01)	2.7×10^{-1}	0.250	24	MR Egger	
		Glucose (mmol/l)	0.01 (-0.01; 0.03)	2.8×10^{-1}	0.776	25	MR Egger	
		Glycated haemoglobin (mmol/mol)	-0.06 (-0.17; 0.05)	3.1×10^{-1}	0.344	25	MR Egger	
		T2DM (OR)	0.99 (0.96; 1.02)	5.6×10^{-1}	0.075	17	IVW	
		BMI (SD)	0.00 (-0.01; 0.01)	7.1×10^{-1}	0.068	20	IVW	
		SBP (mmHg)	0.16 (0.06; 0.25)	9.5×10^{-4}	0.644	19	IVW	
		DBP (mmHg)	0.11 (-0.02; 0.24)	1.1×10^{-1}	0.221	20	MR Egger	
		ECG heart rate (exercise) (BPM)	-0.10 (-0.65; 0.44)	7.1×10^{-1}	0.437	26	MR Egger	
		ECG load (exercise) (Watts)	-0.03 (-0.63; 0.58)	9.4×10^{-1}	0.480	26	MR Egger	
		Asthma (OR)	0.99 (0.95; 1.04)	7.6×10^{-1}	0.110	25	MR Egger	
		CRP (log(mg/L))	-0.01 (-0.06; 0.03)	5.0×10^{-1}	0.548	20	MR Egger	
		eGFR (SD of log(eGFR))	0.00 (-0.00; 0.00)	8.2×10^{-1}	0.346	21	MR Egger	
		BUN (mg/dl)	-0.01 (-0.01; 0.00)	1.8×10^{-1}	0.301	21	MR Egger	
		CKD (OR)	0.94 (0.86; 1.03)	2.2×10^{-1}	0.104	21	MR Egger	
		Alzheimer’s (OR)	0.99 (0.97; 1.00)	1.1×10^{-1}	0.580	22	MR Egger	
		Parkinson’s (OR)	0.84 (0.70; 1.00)	4.5×10^{-2}	0.736	21	MR Egger	
		Lewy body dementia (OR)	1.00 (0.79; 1.26)	9.9×10^{-1}	0.797	22	MR Egger	
		Breast cancer (OR)	0.95 (0.92; 0.97)	2.6×10^{-4}	0.669	19	IVW	
		Lung cancer (OR)	1.22 (0.90; 1.64)	2.0×10^{-1}	0.092	19	MR Egger	
		Colon cancer (OR)	1.21 (0.98; 1.48)	7.2×10^{-2}	0.424	19	MR Egger	
		Prostate cancer (OR)	1.03 (0.97; 1.09)	4.1×10^{-1}	0.119	16	IVW	
GFRA1 (P56159)	Druggable	HF (OR)	1.03 (0.94; 1.13)	5.2×10^{-1}	0.618	10	MR Egger	Interval
		Non-ischemic CM (OR)	0.90 (0.55; 1.48)	6.7×10^{-1}	0.136	10	MR Egger	
		DCM (OR)	0.99 (0.86; 1.15)	9.2×10^{-1}	<0.001	9	IVW	
		AF (OR)	0.96 (0.93; 0.98)	9.4×10^{-4}	0.622	12	IVW	
		CHD (OR)	1.00 (0.91; 1.10)	9.6×10^{-1}	0.880	12	MR Egger	
		cIMT (mm)	-0.00 (-0.01; 0.00)	1.9×10^{-1}	0.903	11	MR Egger	
		Carotid plaque (OR)	0.86 (0.80; 0.91)	8.2×10^{-7}	<0.001	11	IVW	
		Any stroke (OR)	1.02 (0.90; 1.15)	7.8×10^{-1}	0.268	10	MR Egger	
		Any ischemic stroke (OR)	1.00 (0.96; 1.04)	9.0×10^{-1}	0.339	10	IVW	
		LDL cholesterol (mmol/l)	-0.00 (-0.01; 0.00)	3.3×10^{-1}	0.571	10	IVW	
		Apoliprotein B (g/l)	-0.00 (-0.00; 0.00)	4.2×10^{-1}	0.477	11	IVW	
		Triglycerides (mmol/l)	-0.01 (-0.02; 0.00)	9.1×10^{-2}	0.300	11	IVW	
		Cholesterol (mmol/l)	-0.01 (-0.02; 0.00)	6.2×10^{-2}	0.427	11	IVW	
		HDL cholesterol (mmol/l)	-0.01 (-0.02; 0.00)	1.9×10^{-1}	0.586	10	MR Egger	
		Apoliprotein A1 (g/l)	-0.00 (-0.00; 0.00)	8.0×10^{-1}	0.741	10	IVW	
		Glucose (mmol/l)	0.02 (0.01; 0.03)	1.4×10^{-3}	0.037	10	IVW	
		Glycated haemoglobin (mmol/mol)	0.04 (-0.02; 0.10)	1.7×10^{-1}	0.941	11	IVW	
		T2DM (OR)	1.01 (0.99; 1.03)	4.9×10^{-1}	0.467	10	IVW	
		BMI (SD)	-0.01 (-0.02; -0.00)	2.3×10^{-3}	0.257	10	IVW	
		SBP (mmHg)	0.27 (0.16; 0.39)	3.4×10^{-6}	0.811	9	IVW	
		DBP (mmHg)	0.17 (0.11; 0.23)	1.4×10^{-7}	0.963	10	IVW	
		ECG heart rate (exercise) (BPM)	-0.27 (-0.61; 0.07)	1.2×10^{-1}	0.424	11	IVW	

Table S16: Phenome-wide drug target MR scan of proteins with a prioritized CMR association. *(continued)*

Protein (uniprot)	Protein set	Trait	Estimate (95%CI)	P-value	Q p-value	No. variants	MR model	pQTL source
		ECG load (exercise) (Watts)	-0.75 (-1.14; -0.35)	1.9×10^{-4}	0.391	10	IVW	
		Asthma (OR)	0.96 (0.93; 0.98)	2.2×10^{-4}	0.489	11	IVW	
		CRP (log(mg/L))	-0.01 (-0.09; 0.08)	8.7×10^{-1}	0.047	7	MR Egger	
		eGFR (SD of log(eGFR))	-0.00 (-0.01; 0.00)	5.4×10^{-1}	0.993	10	MR Egger	
		BUN (mg/dl)	-0.00 (-0.01; -0.00)	9.5×10^{-3}	0.511	10	IVW	
		CKD (OR)	1.02 (0.99; 1.06)	2.4×10^{-1}	0.627	10	IVW	
		Alzheimer's (OR)	1.01 (1.00; 1.02)	2.2×10^{-1}	0.820	9	IVW	
		Parkinson's (OR)	1.33 (0.96; 1.85)	9.0×10^{-2}	0.138	10	MR Egger	
		Lewy body dementia (OR)	1.01 (0.66; 1.53)	9.8×10^{-1}	0.760	11	MR Egger	
		Breast cancer (OR)	0.97 (0.84; 1.11)	6.3×10^{-1}	0.261	12	MR Egger	
		Lung cancer (OR)	0.93 (0.83; 1.04)	2.1×10^{-1}	0.897	11	IVW	
		Colon cancer (OR)	2.01 (1.42; 2.84)	9.0×10^{-5}	0.891	10	MR Egger	
		Prostate cancer (OR)	0.87 (0.82; 0.92)	2.0×10^{-6}	0.803	11	IVW	
ICOSL (O75144)	Druggable	HF (OR)	1.05 (1.00; 1.09)	2.8×10^{-2}	0.004	34	MR Egger	Interval
		Non-ischemic CM (OR)	1.09 (0.91; 1.30)	3.5×10^{-1}	0.487	31	MR Egger	
		DCM (OR)	1.26 (1.20; 1.33)	1.0×10^{-100}	0.049	36	IVW	
		AF (OR)	1.08 (1.03; 1.12)	1.1×10^{-3}	0.473	36	MR Egger	
		CHD (OR)	1.05 (1.00; 1.09)	4.8×10^{-2}	0.981	33	MR Egger	
		cIMT (mm)	0.00 (-0.00; 0.00)	1.5×10^{-1}	0.034	33	IVW	
		Carotid plaque (OR)	1.12 (1.02; 1.23)	1.3×10^{-2}	0.886	33	MR Egger	
		Any stroke (OR)	0.95 (0.90; 1.01)	1.0×10^{-1}	0.119	34	MR Egger	
		Any ischemic stroke (OR)	0.97 (0.92; 1.03)	3.2×10^{-1}	0.631	33	MR Egger	
		LDL cholesterol (mmol/l)	0.00 (-0.01; 0.01)	9.3×10^{-1}	0.015	40	MR Egger	
		Apoliprotein B (g/l)	-0.00 (-0.00; 0.00)	4.2×10^{-1}	<0.001	40	MR Egger	
		Triglycerides (mmol/l)	-0.00 (-0.01; 0.01)	8.4×10^{-1}	0.043	40	MR Egger	
		Cholesterol (mmol/l)	-0.00 (-0.02; 0.01)	7.2×10^{-1}	0.052	40	MR Egger	
		HDL cholesterol (mmol/l)	0.00 (-0.00; 0.00)	9.9×10^{-1}	0.033	37	MR Egger	
		Apoliprotein A1 (g/l)	-0.00 (-0.01; 0.00)	1.4×10^{-1}	0.017	38	MR Egger	
		Glucose (mmol/l)	0.00 (-0.02; 0.02)	9.4×10^{-1}	0.095	40	MR Egger	
		Glycated haemoglobin (mmol/mol)	0.07 (-0.01; 0.15)	9.1×10^{-2}	0.005	33	MR Egger	
		T2DM (OR)	1.03 (1.02; 1.04)	2.6×10^{-7}	0.244	29	IVW	
		BMI (SD)	0.01 (0.01; 0.01)	1.6×10^{-11}	0.040	30	IVW	
		SBP (mmHg)	-0.14 (-0.30; 0.03)	9.9×10^{-2}	0.474	30	MR Egger	
		DBP (mmHg)	-0.02 (-0.12; 0.07)	6.2×10^{-1}	0.042	30	MR Egger	
		ECG heart rate (exercise) (BPM)	-0.76 (-1.16; -0.36)	1.8×10^{-4}	0.002	39	MR Egger	
		ECG load (exercise) (Watts)	-0.53 (-0.97; -0.08)	2.0×10^{-2}	0.024	38	MR Egger	
		Asthma (OR)	1.02 (1.01; 1.03)	1.5×10^{-3}	0.205	33	IVW	
		CRP (log(mg/L))	-0.03 (-0.06; 0.00)	8.1×10^{-2}	0.208	32	MR Egger	
		eGFR (SD of log(eGFR))	-0.00 (-0.00; 0.00)	7.1×10^{-1}	0.131	29	MR Egger	
		BUN (mg/dl)	-0.00 (-0.01; -0.00)	3.5×10^{-6}	0.426	29	IVW	
		CKD (OR)	0.99 (0.97; 1.01)	4.5×10^{-1}	0.002	25	IVW	
		Alzheimer's (OR)	0.99 (0.98; 1.01)	4.1×10^{-1}	0.060	30	MR Egger	
		Parkinson's (OR)	0.96 (0.93; 0.99)	2.1×10^{-2}	0.046	25	IVW	
		Lewy body dementia (OR)	1.69 (1.36; 2.11)	3.0×10^{-6}	0.088	9	IVW	
		Breast cancer (OR)	0.96 (0.91; 1.02)	1.7×10^{-1}	0.012	36	MR Egger	
		Lung cancer (OR)	1.15 (1.09; 1.22)	7.6×10^{-7}	0.026	34	IVW	
		Colon cancer (OR)	0.93 (0.82; 1.05)	2.3×10^{-1}	0.932	36	MR Egger	
		Prostate cancer (OR)	0.95 (0.87; 1.03)	2.3×10^{-1}	0.098	36	MR Egger	
IL8 (P10145)	Druggable	HF (OR)	0.74 (0.69; 0.81)	4.8×10^{-13}	0.576	3	IVW	Scallop
		Non-ischemic CM (OR)	0.69 (0.51; 0.91)	1.0×10^{-2}	0.732	3	IVW	
		DCM (OR)	1.46 (1.04; 2.04)	2.9×10^{-2}	0.708	3	IVW	
		AF (OR)	0.83 (0.77; 0.89)	9.3×10^{-8}	0.314	3	IVW	
		CHD (OR)	1.18 (1.11; 1.25)	1.2×10^{-8}	0.909	5	IVW	
		cIMT (mm)	0.02 (0.02; 0.03)	7.8×10^{-10}	0.557	3	IVW	
		Carotid plaque (OR)	1.35 (1.13; 1.61)	8.6×10^{-4}	0.396	3	IVW	
		Any stroke (OR)	0.84 (0.76; 0.92)	3.2×10^{-4}	0.958	3	IVW	
		Any ischemic stroke (OR)	0.94 (0.85; 1.05)	2.7×10^{-1}	0.983	3	IVW	
		LDL cholesterol (mmol/l)	0.01 (-0.01; 0.03)	2.7×10^{-1}	0.873	3	IVW	
		Apoliprotein B (g/l)	-0.00 (-0.01; 0.00)	7.6×10^{-1}	0.953	3	IVW	
		Triglycerides (mmol/l)	-0.03 (-0.05; -0.01)	2.2×10^{-3}	0.507	3	IVW	
		Cholesterol (mmol/l)	0.01 (-0.01; 0.03)	3.5×10^{-1}	0.774	3	IVW	
		HDL cholesterol (mmol/l)	0.01 (-0.00; 0.01)	7.9×10^{-2}	0.446	3	IVW	
		Apoliprotein A1 (g/l)	0.01 (0.00; 0.01)	4.7×10^{-3}	0.487	3	IVW	
		Glucose (mmol/l)	0.00 (-0.03; 0.03)	9.1×10^{-1}	0.627	3	IVW	
		Glycated haemoglobin (mmol/mol)	-0.23 (-0.36; -0.09)	1.0×10^{-3}	0.900	3	IVW	
		T2DM (OR)	1.02 (0.96; 1.08)	5.7×10^{-1}	0.806	3	IVW	

Table S16: Phenome-wide drug target MR scan of proteins with a prioritized CMR association. *(continued)*

Protein (uniprot)	Protein set	Trait	Estimate (95%CI)	P-value	Q p-value	No. variants	MR model	pQTL source
		BMI (SD)	-0.02 (-0.04; -0.00)	3.7×10^{-2}	0.238	3	IVW	
		SBP (mmHg)	0.67 (0.39; 0.95)	2.5×10^{-6}	0.385	3	IVW	
		DBP (mmHg)	0.32 (0.16; 0.48)	7.0×10^{-5}	0.897	3	IVW	
		ECG heart rate (exercise) (BPM)	1.89 (1.13; 2.65)	1.2×10^{-6}	0.628	3	IVW	
		ECG load (exercise) (Watts)	2.26 (1.40; 3.11)	2.2×10^{-7}	0.725	3	IVW	
		Asthma (OR)	1.16 (1.11; 1.22)	2.2×10^{-9}	0.974	3	IVW	
		CRP (log(mg/L))	0.03 (-0.01; 0.08)	1.8×10^{-1}	0.610	3	IVW	
		eGFR (SD of log(eGFR))	-0.00 (-0.00; 0.00)	9.9×10^{-1}	0.641	3	IVW	
		BUN (mg/dl)	-0.01 (-0.02; -0.00)	1.8×10^{-2}	0.867	3	IVW	
		CKD (OR)	0.65 (0.59; 0.71)	1.0×10^{-100}	0.932	3	IVW	
		Alzheimer's (OR)	1.04 (1.02; 1.05)	3.3×10^{-5}	0.411	3	IVW	
		Parkinson's (OR)	1.01 (0.84; 1.22)	8.8×10^{-1}	0.863	3	IVW	
		Lewy body dementia (OR)	0.61 (0.16; 2.31)	4.7×10^{-1}	None	1	Wald	
		Breast cancer (OR)	0.89 (0.71; 1.12)	3.2×10^{-1}	0.971	3	IVW	
		Lung cancer (OR)	0.63 (0.37; 1.09)	1.0×10^{-1}	0.847	3	IVW	
		Colon cancer (OR)	1.25 (0.76; 2.04)	3.8×10^{-1}	0.748	3	IVW	
		Prostate cancer (OR)	1.03 (0.74; 1.41)	8.8×10^{-1}	0.307	3	IVW	
LYAM1 (P14151)	Druggable	HF (OR)	0.96 (0.92; 0.99)	1.3×10^{-2}	0.140	27	MR Egger	Interval
		Non-ischemic CM (OR)	0.93 (0.81; 1.06)	2.7×10^{-1}	0.250	31	MR Egger	
		DCM (OR)	1.01 (0.85; 1.21)	9.0×10^{-1}	0.004	30	MR Egger	
		AF (OR)	0.88 (0.85; 0.91)	1.0×10^{-100}	<0.001	28	MR Egger	
		CHD (OR)	1.01 (1.00; 1.02)	1.8×10^{-1}	0.002	31	IVW	
		cIMT (mm)	-0.00 (-0.00; 0.00)	8.2×10^{-2}	<0.001	32	IVW	
		Carotid plaque (OR)	1.11 (1.08; 1.14)	1.0×10^{-13}	<0.001	29	IVW	
		Any stroke (OR)	0.92 (0.89; 0.95)	7.9×10^{-7}	0.290	32	MR Egger	
		Any ischemic stroke (OR)	1.00 (0.98; 1.01)	5.7×10^{-1}	0.101	29	IVW	
		LDL cholesterol (mmol/l)	-0.00 (-0.00; 0.00)	1.0×10^{-1}	0.269	33	IVW	
		Apoliprotein B (g/l)	-0.00 (-0.00; -0.00)	6.3×10^{-3}	0.844	33	IVW	
		Triglycerides (mmol/l)	-0.00 (-0.00; 0.00)	3.2×10^{-1}	0.011	32	IVW	
		Cholesterol (mmol/l)	-0.01 (-0.01; -0.00)	2.1×10^{-3}	0.126	33	IVW	
		HDL cholesterol (mmol/l)	0.00 (-0.00; 0.00)	3.7×10^{-1}	0.007	31	MR Egger	
		Apoliprotein A1 (g/l)	-0.00 (-0.00; 0.00)	8.3×10^{-1}	0.343	30	MR Egger	
		Glucose (mmol/l)	0.01 (-0.00; 0.03)	9.2×10^{-2}	0.126	31	MR Egger	
		Glycated haemoglobin (mmol/mol)	-0.08 (-0.10; -0.06)	1.2×10^{-13}	0.123	31	IVW	
		T2DM (OR)	1.01 (0.99; 1.04)	2.9×10^{-1}	0.878	31	MR Egger	
		BMI (SD)	-0.01 (-0.01; 0.00)	1.9×10^{-1}	0.173	31	MR Egger	
		SBP (mmHg)	-0.32 (-0.49; -0.16)	1.1×10^{-4}	<0.001	26	MR Egger	
		DBP (mmHg)	-0.16 (-0.23; -0.09)	5.5×10^{-6}	0.021	28	MR Egger	
		ECG heart rate (exercise) (BPM)	0.43 (0.09; 0.76)	1.3×10^{-2}	0.287	31	MR Egger	
		ECG load (exercise) (Watts)	0.52 (0.15; 0.89)	5.4×10^{-3}	0.092	31	MR Egger	
		Asthma (OR)	0.97 (0.94; 0.99)	5.7×10^{-3}	0.005	29	MR Egger	
		CRP (log(mg/L))	0.02 (0.02; 0.03)	2.1×10^{-13}	0.012	31	IVW	
		eGFR (SD of log(eGFR))	-0.00 (-0.00; -0.00)	7.9×10^{-3}	0.011	31	MR Egger	
		BUN (mg/dl)	0.00 (-0.00; 0.00)	8.6×10^{-1}	0.200	28	MR Egger	
		CKD (OR)	1.00 (0.99; 1.02)	5.2×10^{-1}	0.362	32	IVW	
		Alzheimer's (OR)	0.99 (0.99; 1.00)	1.0×10^{-5}	0.103	33	IVW	
		Parkinson's (OR)	1.10 (1.01; 1.21)	3.6×10^{-2}	0.516	30	MR Egger	
		Lewy body dementia (OR)	1.05 (0.99; 1.13)	1.1×10^{-1}	0.470	29	IVW	
		Breast cancer (OR)	0.95 (0.93; 0.98)	3.8×10^{-5}	0.027	29	IVW	
		Lung cancer (OR)	0.74 (0.67; 0.82)	1.2×10^{-8}	0.213	32	MR Egger	
		Colon cancer (OR)	1.00 (0.90; 1.10)	9.4×10^{-1}	0.170	31	MR Egger	
		Prostate cancer (OR)	0.91 (0.89; 0.93)	8.9×10^{-16}	0.014	31	IVW	
MFGM (Q08431)	Druggable	HF (OR)	0.98 (0.96; 1.00)	1.1×10^{-1}	0.031	8	IVW	Interval
		Non-ischemic CM (OR)	1.02 (0.92; 1.13)	7.3×10^{-1}	0.684	8	IVW	
		DCM (OR)	1.43 (1.22; 1.68)	1.0×10^{-5}	0.022	8	IVW	
		AF (OR)	0.93 (0.89; 0.97)	1.4×10^{-3}	0.475	8	IVW	
		CHD (OR)	0.97 (0.91; 1.04)	3.9×10^{-1}	0.790	8	IVW	
		cIMT (mm)	0.00 (-0.00; 0.00)	5.2×10^{-1}	0.035	9	IVW	
		Carotid plaque (OR)	1.04 (0.81; 1.32)	7.7×10^{-1}	0.510	9	MR Egger	
		Any stroke (OR)	1.02 (0.99; 1.05)	2.7×10^{-1}	0.316	9	IVW	
		Any ischemic stroke (OR)	1.03 (0.99; 1.07)	1.3×10^{-1}	0.248	9	IVW	
		LDL cholesterol (mmol/l)	-0.02 (-0.02; -0.01)	9.0×10^{-9}	0.731	9	IVW	
		Apoliprotein B (g/l)	-0.00 (-0.01; -0.00)	9.6×10^{-11}	0.550	9	IVW	
		Triglycerides (mmol/l)	-0.02 (-0.03; -0.01)	1.2×10^{-6}	0.116	9	IVW	
		Cholesterol (mmol/l)	-0.02 (-0.02; -0.01)	6.3×10^{-6}	0.862	9	IVW	
		HDL cholesterol (mmol/l)	0.00 (-0.00; 0.01)	6.3×10^{-2}	0.094	8	IVW	

Table S16: Phenome-wide drug target MR scan of proteins with a prioritized CMR association. *(continued)*

Protein (uniprot)	Protein set	Trait	Estimate (95%CI)	P-value	Q p-value	No. variants	MR model	pQTL source
		Apoliprotein A1 (g/l)	0.02 (-0.00; 0.03)	1.4×10^{-1}	0.113	8	MR Egger	
		Glucose (mmol/l)	-0.02 (-0.09; 0.04)	4.9×10^{-1}	0.376	9	MR Egger	
		Glycated haemoglobin (mmol/mol)	-0.05 (-0.09; -0.01)	2.1×10^{-2}	0.562	9	IVW	
		T2DM (OR)	1.00 (0.98; 1.02)	9.2×10^{-1}	0.403	8	IVW	
		BMI (SD)	-0.02 (-0.02; -0.01)	2.8×10^{-7}	0.021	8	IVW	
		SBP (mmHg)	0.05 (-0.07; 0.18)	4.0×10^{-1}	0.111	8	IVW	
		DBP (mmHg)	0.27 (0.19; 0.34)	5.4×10^{-13}	0.111	8	IVW	
		ECG heart rate (exercise) (BPM)	2.09 (0.13; 4.05)	3.6×10^{-2}	0.368	9	MR Egger	
		ECG load (exercise) (Watts)	0.39 (0.13; 0.64)	3.0×10^{-3}	0.665	9	IVW	
		Asthma (OR)	1.00 (0.99; 1.02)	9.5×10^{-1}	0.502	12	IVW	
		CRP (log(mg/L))	-0.01 (-0.03; -0.00)	3.1×10^{-2}	0.824	9	IVW	
		eGFR (SD of log(eGFR))	0.00 (-0.00; 0.00)	6.0×10^{-2}	0.910	8	IVW	
		BUN (mg/dl)	0.00 (0.00; 0.01)	6.1×10^{-3}	0.967	8	IVW	
		CKD (OR)	0.99 (0.96; 1.02)	6.0×10^{-1}	0.494	8	IVW	
		Alzheimer's (OR)	1.00 (0.99; 1.02)	5.9×10^{-1}	0.374	8	IVW	
		Parkinson's (OR)	0.90 (0.84; 0.95)	6.6×10^{-4}	0.447	8	IVW	
		Lewy body dementia (OR)	0.92 (0.67; 1.26)	5.9×10^{-1}	0.388	7	IVW	
		Breast cancer (OR)	0.97 (0.89; 1.06)	5.4×10^{-1}	0.719	8	IVW	
		Lung cancer (OR)	0.98 (0.77; 1.23)	8.5×10^{-1}	0.406	8	IVW	
		Colon cancer (OR)	1.15 (0.93; 1.43)	2.1×10^{-1}	0.232	8	IVW	
		Prostate cancer (OR)	0.99 (0.88; 1.11)	8.1×10^{-1}	0.831	8	IVW	
MK03 (P27361)	Druggable	HF (OR)	0.85 (0.80; 0.91)	3.9×10^{-7}	0.768	3	IVW	Interval
		Non-ischemic CM (OR)	0.77 (0.57; 1.02)	7.0×10^{-2}	0.499	3	IVW	
		DCM (OR)	0.82 (0.61; 1.10)	1.9×10^{-1}	0.503	3	IVW	
		AF (OR)	0.86 (0.82; 0.91)	6.2×10^{-8}	0.663	3	IVW	
		CHD (OR)	1.00 (0.94; 1.07)	8.9×10^{-1}	0.909	3	IVW	
		cIMT (mm)	0.00 (-0.00; 0.01)	1.1×10^{-1}	0.807	3	IVW	
		Carotid plaque (OR)	1.13 (0.98; 1.30)	8.1×10^{-2}	0.702	3	IVW	
		Any stroke (OR)	1.16 (1.08; 1.25)	6.3×10^{-5}	0.846	3	IVW	
		Any ischemic stroke (OR)	1.14 (1.05; 1.23)	1.0×10^{-3}	0.932	3	IVW	
		LDL cholesterol (mmol/l)	0.01 (-0.01; 0.04)	3.3×10^{-1}	0.613	3	IVW	
		Apoliprotein B (g/l)	0.00 (-0.01; 0.01)	8.0×10^{-1}	0.736	3	IVW	
		Triglycerides (mmol/l)	-0.04 (-0.07; -0.01)	1.9×10^{-2}	0.581	3	IVW	
		Cholesterol (mmol/l)	0.03 (-0.00; 0.07)	7.0×10^{-2}	0.709	3	IVW	
		HDL cholesterol (mmol/l)	0.03 (0.02; 0.04)	4.8×10^{-6}	0.798	3	IVW	
		Apoliprotein A1 (g/l)	0.02 (0.01; 0.03)	3.0×10^{-6}	0.576	3	IVW	
		Glucose (mmol/l)	0.08 (-0.10; 0.26)	3.9×10^{-1}	0.306	3	MR Egger	
		Glycated haemoglobin (mmol/mol)	1.18 (0.26; 2.09)	1.1×10^{-2}	0.710	3	MR Egger	
		T2DM (OR)	0.82 (0.53; 1.25)	3.5×10^{-1}	0.143	3	MR Egger	
		BMI (SD)	-0.15 (-0.17; -0.14)	1.0×10^{-100}	0.361	3	IVW	
		SBP (mmHg)	-0.22 (-1.87; 1.43)	7.9×10^{-1}	0.230	3	MR Egger	
		DBP (mmHg)	0.13 (-1.14; 1.40)	8.4×10^{-1}	0.110	3	MR Egger	
		ECG heart rate (exercise) (BPM)	0.10 (-1.36; 1.56)	8.9×10^{-1}	0.205	3	IVW	
		ECG load (exercise) (Watts)	-0.37 (-2.25; 1.51)	7.0×10^{-1}	0.124	3	IVW	
		Asthma (OR)	0.93 (0.87; 1.00)	6.5×10^{-2}	0.438	4	IVW	
		CRP (log(mg/L))	-0.05 (-0.08; -0.02)	2.1×10^{-3}	0.919	3	IVW	
		eGFR (SD of log(eGFR))	0.01 (0.01; 0.01)	9.6×10^{-10}	0.736	3	IVW	
		BUN (mg/dl)	0.01 (-0.04; 0.05)	8.1×10^{-1}	0.217	3	MR Egger	
		CKD (OR)	0.94 (0.86; 1.02)	1.2×10^{-1}	0.287	3	IVW	
		Alzheimer's (OR)	1.30 (0.82; 2.08)	2.7×10^{-1}	0.039	3	MR Egger	
		Parkinson's (OR)	1.01 (0.79; 1.29)	9.4×10^{-1}	0.101	3	IVW	
		Lewy body dementia (OR)	2.02 (1.43; 2.86)	7.5×10^{-5}	0.212	3	IVW	
		Breast cancer (OR)	0.87 (0.70; 1.09)	2.3×10^{-1}	0.832	2	IVW	
		Lung cancer (OR)	0.84 (0.46; 1.53)	5.6×10^{-1}	0.834	2	IVW	
		Colon cancer (OR)	0.90 (0.56; 1.44)	6.6×10^{-1}	0.767	2	IVW	
		Prostate cancer (OR)	1.17 (0.86; 1.58)	3.2×10^{-1}	0.784	2	IVW	
PA2GA (P14555)	Druggable	HF (OR)	1.05 (1.03; 1.06)	2.1×10^{-8}	<0.001	42	MR Egger	Interval
		Non-ischemic CM (OR)	1.01 (0.92; 1.11)	8.8×10^{-1}	0.121	43	MR Egger	
		DCM (OR)	1.13 (1.03; 1.23)	8.6×10^{-3}	0.082	44	MR Egger	
		AF (OR)	1.00 (1.00; 1.00)	8.6×10^{-1}	0.002	46	IVW	
		CHD (OR)	0.99 (0.97; 1.01)	4.9×10^{-1}	<0.001	39	MR Egger	
		cIMT (mm)	-0.00 (-0.00; -0.00)	1.1×10^{-6}	0.175	43	IVW	
		Carotid plaque (OR)	1.02 (1.00; 1.03)	7.7×10^{-3}	0.415	42	IVW	
		Any stroke (OR)	1.03 (1.00; 1.05)	2.5×10^{-2}	0.073	40	MR Egger	
		Any ischemic stroke (OR)	1.01 (0.99; 1.04)	3.4×10^{-1}	0.142	39	MR Egger	
		LDL cholesterol (mmol/l)	0.00 (0.00; 0.01)	3.0×10^{-4}	<0.001	50	MR Egger	

Table S16: Phenome-wide drug target MR scan of proteins with a prioritized CMR association. *(continued)*

Protein (uniprot)	Protein set	Trait	Estimate (95%CI)	P-value	Q p-value	No. variants	MR model	pQTL source
		Apoliprotein B (g/l)	0.00 (0.00; 0.00)	6.3×10^{-3}	<0.001	51	MR Egger	
		Triglycerides (mmol/l)	-0.01 (-0.01; -0.00)	4.5×10^{-5}	0.072	52	MR Egger	
		Cholesterol (mmol/l)	0.01 (0.00; 0.01)	7.2×10^{-4}	<0.001	53	MR Egger	
		HDL cholesterol (mmol/l)	0.00 (0.00; 0.00)	1.0×10^{-6}	<0.001	45	MR Egger	
		Apoliprotein A1 (g/l)	0.00 (0.00; 0.00)	3.0×10^{-2}	<0.001	46	MR Egger	
		Glucose (mmol/l)	-0.00 (-0.00; -0.00)	2.2×10^{-4}	<0.001	51	IVW	
		Glycated haemoglobin (mmol/mol)	-0.07 (-0.09; -0.05)	2.9×10^{-10}	0.007	51	MR Egger	
		T2DM (OR)	1.00 (0.99; 1.02)	9.5×10^{-1}	0.182	43	MR Egger	
		BMI (SD)	-0.01 (-0.01; -0.00)	1.4×10^{-13}	0.005	42	IVW	
		SBP (mmHg)	-0.04 (-0.13; 0.06)	4.5×10^{-1}	0.017	33	MR Egger	
		DBP (mmHg)	0.00 (-0.04; 0.04)	9.9×10^{-1}	0.005	40	MR Egger	
		ECG heart rate (exercise) (BPM)	-0.05 (-0.11; -0.00)	3.6×10^{-2}	0.004	47	IVW	
		ECG load (exercise) (Watts)	-0.02 (-0.08; 0.03)	4.5×10^{-1}	<0.001	50	IVW	
		Asthma (OR)	1.01 (1.00; 1.02)	4.6×10^{-2}	0.056	51	MR Egger	
		CRP (log(mg/L))	-0.01 (-0.02; -0.00)	3.9×10^{-2}	0.041	43	MR Egger	
		eGFR (SD of log(eGFR))	-0.00 (-0.00; -0.00)	8.2×10^{-6}	0.012	41	MR Egger	
		BUN (mg/dl)	-0.00 (-0.00; 0.00)	6.6×10^{-1}	<0.001	43	MR Egger	
		CKD (OR)	1.03 (1.00; 1.05)	3.9×10^{-2}	<0.001	39	MR Egger	
		Alzheimer’s (OR)	1.00 (0.99; 1.00)	7.5×10^{-2}	0.015	41	MR Egger	
		Parkinson’s (OR)	0.98 (0.93; 1.04)	5.8×10^{-1}	0.004	43	MR Egger	
		Lewy body dementia (OR)	1.04 (1.00; 1.08)	3.4×10^{-2}	0.051	36	IVW	
		Breast cancer (OR)	0.93 (0.91; 0.95)	1.6×10^{-9}	0.278	41	MR Egger	
		Lung cancer (OR)	1.13 (1.07; 1.19)	6.0×10^{-6}	0.014	45	MR Egger	
		Colon cancer (OR)	0.91 (0.86; 0.96)	1.4×10^{-3}	0.003	42	MR Egger	
		Prostate cancer (OR)	1.01 (0.99; 1.04)	3.4×10^{-1}	0.003	44	MR Egger	
PD1L2 (Q9BQ51)	Druggable	HF (OR)	1.04 (0.99; 1.09)	1.5×10^{-1}	0.020	29	MR Egger	Interval
		Non-ischemic CM (OR)	1.08 (0.86; 1.35)	5.2×10^{-1}	0.120	31	MR Egger	
		DCM (OR)	0.98 (0.77; 1.24)	8.5×10^{-1}	0.571	30	MR Egger	
		AF (OR)	1.03 (0.99; 1.07)	2.2×10^{-1}	0.020	31	MR Egger	
		CHD (OR)	1.00 (0.98; 1.01)	7.2×10^{-1}	0.794	26	IVW	
		cIMT (mm)	0.00 (-0.00; 0.00)	9.3×10^{-1}	0.440	31	MR Egger	
		Carotid plaque (OR)	0.99 (0.96; 1.02)	3.7×10^{-1}	0.175	26	IVW	
		Any stroke (OR)	1.02 (1.00; 1.04)	3.8×10^{-2}	0.665	24	IVW	
		Any ischemic stroke (OR)	1.03 (0.96; 1.11)	3.9×10^{-1}	0.063	31	MR Egger	
		LDL cholesterol (mmol/l)	-0.01 (-0.03; 0.00)	5.4×10^{-2}	0.174	32	MR Egger	
		Apoliprotein B (g/l)	-0.00 (-0.01; 0.00)	1.0×10^{-1}	0.127	32	MR Egger	
		Triglycerides (mmol/l)	-0.01 (-0.02; 0.01)	4.5×10^{-1}	0.042	33	MR Egger	
		Cholesterol (mmol/l)	-0.01 (-0.03; 0.00)	5.9×10^{-2}	0.591	32	MR Egger	
		HDL cholesterol (mmol/l)	0.00 (0.00; 0.01)	9.1×10^{-11}	0.046	29	IVW	
		Apoliprotein A1 (g/l)	-0.00 (-0.00; 0.00)	9.9×10^{-1}	0.112	33	MR Egger	
		Glucose (mmol/l)	0.01 (-0.01; 0.03)	3.6×10^{-1}	0.844	33	MR Egger	
		Glycated haemoglobin (mmol/mol)	0.04 (-0.05; 0.13)	3.4×10^{-1}	0.391	33	MR Egger	
		T2DM (OR)	0.98 (0.97; 0.99)	9.8×10^{-5}	0.341	27	IVW	
		BMI (SD)	-0.00 (-0.00; 0.00)	7.9×10^{-1}	0.100	28	IVW	
		SBP (mmHg)	-0.02 (-0.08; 0.03)	4.3×10^{-1}	0.088	25	IVW	
		DBP (mmHg)	-0.07 (-0.17; 0.04)	2.3×10^{-1}	0.357	30	MR Egger	
		ECG heart rate (exercise) (BPM)	0.39 (-0.16; 0.93)	1.7×10^{-1}	0.208	33	MR Egger	
		ECG load (exercise) (Watts)	-0.06 (-0.23; 0.11)	4.8×10^{-1}	0.186	32	IVW	
		Asthma (OR)	1.05 (1.04; 1.06)	1.0×10^{-100}	0.002	24	IVW	
		CRP (log(mg/L))	-0.01 (-0.01; -0.00)	3.6×10^{-2}	0.431	27	IVW	
		eGFR (SD of log(eGFR))	0.00 (-0.00; 0.00)	7.2×10^{-1}	0.948	26	IVW	
		BUN (mg/dl)	-0.00 (-0.00; -0.00)	1.4×10^{-2}	0.449	26	IVW	
		CKD (OR)	0.99 (0.97; 1.00)	9.0×10^{-2}	0.031	26	IVW	
		Alzheimer’s (OR)	1.01 (0.99; 1.02)	4.0×10^{-1}	0.137	30	MR Egger	
		Parkinson’s (OR)	0.83 (0.80; 0.86)	1.0×10^{-100}	0.023	26	IVW	
		Lewy body dementia (OR)	1.01 (0.81; 1.26)	9.3×10^{-1}	0.565	31	MR Egger	
		Breast cancer (OR)	1.00 (0.98; 1.01)	7.8×10^{-1}	0.105	27	IVW	
		Lung cancer (OR)	1.02 (0.98; 1.06)	4.5×10^{-1}	0.021	25	IVW	
		Colon cancer (OR)	0.96 (0.92; 1.00)	4.5×10^{-2}	0.002	24	IVW	
		Prostate cancer (OR)	0.94 (0.85; 1.04)	2.3×10^{-1}	0.020	27	MR Egger	
PMEL (P40967)	Druggable	HF (OR)	0.96 (0.82; 1.13)	6.3×10^{-1}	None	1	Wald	Interval
		Non-ischemic CM (OR)	0.68 (0.34; 1.36)	2.8×10^{-1}	None	1	Wald	
		DCM (OR)	0.50 (0.35; 0.71)	1.2×10^{-4}	0.911	2	IVW	
		AF (OR)	0.99 (0.92; 1.06)	6.9×10^{-1}	0.529	2	IVW	
		CHD (OR)	1.00 (0.84; 1.20)	9.6×10^{-1}	None	1	Wald	
		cIMT (mm)	-0.01 (-0.03; 0.00)	6.7×10^{-2}	None	1	Wald	

Table S16: Phenome-wide drug target MR scan of proteins with a prioritized CMR association. *(continued)*

Protein (uniprot)	Protein set	Trait	Estimate (95%CI)	P-value	Q p-value	No. variants	MR model	pQTL source
		Carotid plaque (OR)	0.67 (0.48; 0.92)	1.5×10^{-2}	None	1	Wald	
		Any stroke (OR)	0.87 (0.72; 1.05)	1.4×10^{-1}	None	1	Wald	
		Any ischemic stroke (OR)	0.84 (0.68; 1.03)	8.9×10^{-2}	None	1	Wald	
		LDL cholesterol (mmol/l)	-0.02 (-0.04; -0.00)	1.8×10^{-2}	0.762	2	IVW	
		Apoliprotein B (g/l)	-0.01 (-0.01; -0.00)	4.5×10^{-4}	0.910	2	IVW	
		Triglycerides (mmol/l)	-0.06 (-0.08; -0.04)	2.3×10^{-8}	0.924	2	IVW	
		Cholesterol (mmol/l)	-0.02 (-0.04; 0.01)	1.4×10^{-1}	0.803	2	IVW	
		HDL cholesterol (mmol/l)	0.02 (0.01; 0.03)	5.5×10^{-8}	0.825	2	IVW	
		Apoliprotein A1 (g/l)	0.01 (0.01; 0.02)	2.8×10^{-7}	0.784	2	IVW	
		Glucose (mmol/l)	-0.01 (-0.03; 0.02)	4.6×10^{-1}	0.769	2	IVW	
		Glycated haemoglobin (mmol/mol)	0.11 (-0.02; 0.23)	9.8×10^{-2}	0.980	2	IVW	
		T2DM (OR)	1.00 (0.88; 1.13)	9.9×10^{-1}	None	1	Wald	
		BMI (SD)	0.02 (-0.02; 0.06)	3.5×10^{-1}	None	1	Wald	
		SBP (mmHg)	-0.06 (-0.66; 0.55)	8.6×10^{-1}	None	1	Wald	
		DBP (mmHg)	0.11 (-0.23; 0.46)	5.2×10^{-1}	None	1	Wald	
		ECG heart rate (exercise) (BPM)	-1.16 (-1.89; -0.44)	1.7×10^{-3}	0.501	2	IVW	
		ECG load (exercise) (Watts)	-0.74 (-1.55; 0.07)	7.5×10^{-2}	0.612	2	IVW	
		Asthma (OR)	1.04 (0.99; 1.09)	1.4×10^{-1}	0.415	2	IVW	
		CRP (log(mg/L))	0.07 (-0.01; 0.15)	8.9×10^{-2}	None	1	Wald	
		eGFR (SD of log(eGFR))	0.01 (0.01; 0.02)	2.0×10^{-4}	None	1	Wald	
		BUN (mg/dl)	-0.00 (-0.02; 0.01)	6.2×10^{-1}	None	1	Wald	
		CKD (OR)	0.90 (0.75; 1.09)	2.8×10^{-1}	None	1	Wald	
		Alzheimer's (OR)	1.04 (1.01; 1.08)	7.5×10^{-3}	0.549	2	IVW	
		Parkinson's (OR)	0.94 (0.64; 1.37)	7.4×10^{-1}	None	1	Wald	
		Lewy body dementia (OR)	1.69 (1.22; 2.34)	1.7×10^{-3}	0.976	2	IVW	
		Breast cancer (OR)	1.11 (1.01; 1.21)	3.8×10^{-2}	0.324	2	IVW	
		Lung cancer (OR)	0.84 (0.64; 1.09)	1.9×10^{-1}	0.695	2	IVW	
		Colon cancer (OR)	1.30 (1.06; 1.59)	1.1×10^{-2}	0.824	2	IVW	
		Prostate cancer (OR)	1.23 (1.08; 1.40)	1.4×10^{-3}	0.948	2	IVW	
TDGF1 (P13385)	Druggable	HF (OR)	0.98 (0.98; 0.99)	3.1×10^{-4}	0.019	42	MR Egger	Interval
		Non-ischemic CM (OR)	0.93 (0.92; 0.94)	1.0×10^{-100}	<0.001	47	IVW	
		DCM (OR)	1.01 (0.97; 1.06)	5.6×10^{-1}	0.002	43	MR Egger	
		AF (OR)	1.01 (1.01; 1.01)	2.8×10^{-10}	0.081	49	IVW	
		CHD (OR)	0.99 (0.99; 1.00)	1.1×10^{-6}	0.002	44	IVW	
		cIMT (mm)	-0.00 (-0.00; -0.00)	1.3×10^{-11}	0.007	48	MR Egger	
		Carotid plaque (OR)	0.98 (0.97; 0.99)	7.6×10^{-6}	0.003	41	IVW	
		Any stroke (OR)	0.99 (0.99; 1.00)	7.2×10^{-2}	0.006	34	IVW	
		Any ischemic stroke (OR)	1.01 (1.00; 1.01)	1.2×10^{-1}	0.013	35	IVW	
		LDL cholesterol (mmol/l)	0.00 (0.00; 0.00)	4.7×10^{-2}	0.016	50	MR Egger	
		Apoliprotein B (g/l)	0.00 (-0.00; 0.00)	1.1×10^{-1}	<0.001	55	MR Egger	
		Triglycerides (mmol/l)	0.00 (-0.00; 0.00)	2.9×10^{-1}	<0.001	40	IVW	
		Cholesterol (mmol/l)	0.00 (-0.00; 0.00)	6.2×10^{-1}	<0.001	51	MR Egger	
		HDL cholesterol (mmol/l)	-0.00 (-0.00; -0.00)	2.7×10^{-8}	<0.001	45	IVW	
		Apoliprotein A1 (g/l)	-0.00 (-0.00; -0.00)	1.9×10^{-11}	<0.001	44	IVW	
		Glucose (mmol/l)	0.00 (0.00; 0.01)	2.2×10^{-4}	0.562	40	IVW	
		Glycated haemoglobin (mmol/mol)	0.00 (-0.01; 0.01)	3.8×10^{-1}	<0.001	38	IVW	
		T2DM (OR)	0.99 (0.98; 0.99)	8.5×10^{-4}	<0.001	37	MR Egger	
		BMI (SD)	-0.00 (-0.01; 0.00)	1.9×10^{-1}	<0.001	26	MR Egger	
		SBP (mmHg)	-0.07 (-0.12; -0.02)	2.6×10^{-3}	<0.001	25	MR Egger	
		DBP (mmHg)	-0.04 (-0.06; -0.02)	9.9×10^{-4}	<0.001	29	MR Egger	
		ECG heart rate (exercise) (BPM)	0.02 (-0.04; 0.08)	4.5×10^{-1}	<0.001	48	IVW	
		ECG load (exercise) (Watts)	0.07 (-0.00; 0.13)	5.2×10^{-2}	<0.001	48	IVW	
		Asthma (OR)	0.99 (0.99; 0.99)	8.1×10^{-6}	<0.001	53	MR Egger	
		CRP (log(mg/L))	-0.00 (-0.00; 0.00)	2.0×10^{-1}	0.107	51	IVW	
		eGFR (SD of log(eGFR))	-0.00 (-0.00; 0.00)	9.3×10^{-2}	0.006	48	MR Egger	
		BUN (mg/dl)	0.00 (-0.00; 0.00)	1.7×10^{-1}	0.007	43	MR Egger	
		CKD (OR)	1.00 (1.00; 1.01)	2.7×10^{-1}	<0.001	45	MR Egger	
		Alzheimer's (OR)	1.00 (1.00; 1.00)	1.0×10^{-100}	0.039	48	IVW	
		Parkinson's (OR)	1.00 (0.99; 1.01)	9.8×10^{-1}	0.002	34	IVW	
		Lewy body dementia (OR)	0.99 (0.94; 1.03)	4.8×10^{-1}	0.006	38	MR Egger	
		Breast cancer (OR)	1.01 (0.99; 1.02)	4.6×10^{-1}	<0.001	46	MR Egger	
		Lung cancer (OR)	1.05 (1.01; 1.08)	3.9×10^{-3}	<0.001	52	MR Egger	
		Colon cancer (OR)	1.00 (0.97; 1.04)	8.0×10^{-1}	0.053	49	MR Egger	
		Prostate cancer (OR)	1.01 (1.00; 1.03)	8.5×10^{-3}	<0.001	43	IVW	
TGFB2 (P61812)	Druggable	HF (OR)	1.07 (0.91; 1.25)	4.0×10^{-1}	None	1	Wald	Interval
		Non-ischemic CM (OR)	1.22 (0.63; 2.39)	5.5×10^{-1}	None	1	Wald	

Table S16: Phenome-wide drug target MR scan of proteins with a prioritized CMR association. *(continued)*

Protein (uniprot)	Protein set	Trait	Estimate (95%CI)	P-value	Q p-value	No. variants	MR model	pQTL source
		DCM (OR)	0.57 (0.27; 1.20)	1.4×10^{-1}	None	1	Wald	
		AF (OR)	1.23 (1.07; 1.40)	2.5×10^{-3}	None	1	Wald	
		CHD (OR)	1.00 (0.86; 1.16)	1.0×10^{0}	None	1	Wald	
		cIMT (mm)	0.01 (-0.00; 0.03)	1.0×10^{-1}	None	1	Wald	
		Carotid plaque (OR)	1.04 (0.75; 1.43)	8.2×10^{-1}	None	1	Wald	
		Any stroke (OR)	1.27 (1.06; 1.53)	9.8×10^{-3}	None	1	Wald	
		Any ischemic stroke (OR)	1.26 (1.03; 1.55)	2.3×10^{-2}	None	1	Wald	
		LDL cholesterol (mmol/l)	-0.02 (-0.04; -0.00)	1.4×10^{-2}	0.664	2	IVW	
		Apoliprotein B (g/l)	-0.01 (-0.01; -0.00)	4.8×10^{-3}	0.658	2	IVW	
		Triglycerides (mmol/l)	0.02 (-0.00; 0.04)	9.0×10^{-2}	0.831	2	IVW	
		Cholesterol (mmol/l)	-0.03 (-0.06; -0.00)	2.0×10^{-2}	0.866	2	IVW	
		HDL cholesterol (mmol/l)	-0.01 (-0.02; -0.00)	4.3×10^{-2}	0.337	2	IVW	
		Apoliprotein A1 (g/l)	-0.00 (-0.01; 0.01)	9.4×10^{-1}	0.266	2	IVW	
		Glucose (mmol/l)	0.04 (0.01; 0.07)	7.2×10^{-3}	0.486	2	IVW	
		Glycated haemoglobin (mmol/mol)	0.20 (-0.01; 0.41)	5.8×10^{-2}	0.152	2	IVW	
		T2DM (OR)	1.20 (1.06; 1.37)	4.7×10^{-3}	None	1	Wald	
		BMI (SD)	0.01 (-0.03; 0.04)	6.4×10^{-1}	None	1	Wald	
		SBP (mmHg)	-0.95 (-1.55; -0.34)	2.1×10^{-3}	None	1	Wald	
		DBP (mmHg)	-0.37 (-0.72; -0.03)	3.5×10^{-2}	None	1	Wald	
		ECG heart rate (exercise) (BPM)	-0.09 (-0.92; 0.74)	8.3×10^{-1}	0.380	2	IVW	
		ECG load (exercise) (Watts)	-0.44 (-1.37; 0.49)	3.5×10^{-1}	0.854	2	IVW	
		Asthma (OR)	0.87 (0.83; 0.92)	1.7×10^{-6}	0.872	2	IVW	
		CRP (log(mg/L))	0.06 (-0.02; 0.14)	1.7×10^{-1}	None	1	Wald	
		eGFR (SD of log(eGFR))	0.00 (-0.00; 0.01)	2.1×10^{-1}	None	1	Wald	
		BUN (mg/dl)	-0.00 (-0.02; 0.02)	7.8×10^{-1}	None	1	Wald	
		CKD (OR)	0.87 (0.71; 1.05)	1.5×10^{-1}	None	1	Wald	
		Alzheimer’s (OR)	1.05 (1.01; 1.09)	1.0×10^{-2}	None	1	Wald	
		Parkinson’s (OR)	0.69 (0.45; 1.07)	9.7×10^{-2}	None	1	Wald	
		Lewy body dementia (OR)	1.38 (0.66; 2.89)	3.9×10^{-1}	None	1	Wald	
		Breast cancer (OR)	1.09 (0.97; 1.22)	1.4×10^{-1}	0.801	2	IVW	
		Lung cancer (OR)	0.83 (0.62; 1.13)	2.4×10^{-1}	0.942	2	IVW	
		Colon cancer (OR)	1.42 (1.13; 1.79)	2.7×10^{-3}	0.778	2	IVW	
		Prostate cancer (OR)	0.81 (0.70; 0.94)	6.0×10^{-3}	0.362	2	IVW	
TLR4 (O00206)	Druggable	HF (OR)	0.97 (0.96; 0.99)	2.8×10^{-3}	0.295	14	IVW	Interval
		Non-ischemic CM (OR)	0.95 (0.81; 1.12)	5.5×10^{-1}	0.363	18	MR Egger	
		DCM (OR)	1.12 (1.03; 1.23)	1.0×10^{-2}	0.313	17	IVW	
		AF (OR)	0.97 (0.95; 0.99)	8.6×10^{-4}	0.032	18	IVW	
		CHD (OR)	1.00 (0.96; 1.04)	8.9×10^{-1}	0.241	17	MR Egger	
		cIMT (mm)	-0.00 (-0.00; 0.00)	1.0×10^{-1}	0.608	15	IVW	
		Carotid plaque (OR)	0.99 (0.95; 1.03)	7.1×10^{-1}	0.146	15	IVW	
		Any stroke (OR)	0.96 (0.93; 0.98)	3.8×10^{-4}	0.135	15	IVW	
		Any ischemic stroke (OR)	0.95 (0.93; 0.97)	7.3×10^{-7}	0.667	15	IVW	
		LDL cholesterol (mmol/l)	-0.00 (-0.01; 0.00)	3.4×10^{-1}	<0.001	17	IVW	
		Apoliprotein B (g/l)	-0.00 (-0.00; -0.00)	1.4×10^{-2}	<0.001	19	IVW	
		Triglycerides (mmol/l)	-0.01 (-0.02; -0.01)	1.4×10^{-4}	0.530	17	IVW	
		Cholesterol (mmol/l)	-0.01 (-0.02; -0.01)	9.1×10^{-5}	<0.001	18	IVW	
		HDL cholesterol (mmol/l)	-0.01 (-0.01; -0.00)	1.5×10^{-2}	0.469	18	MR Egger	
		Apoliprotein A1 (g/l)	-0.00 (-0.00; -0.00)	4.3×10^{-4}	0.573	19	IVW	
		Glucose (mmol/l)	-0.02 (-0.03; -0.01)	1.3×10^{-10}	0.031	19	IVW	
		Glycated haemoglobin (mmol/mol)	-0.09 (-0.14; -0.05)	2.9×10^{-5}	0.216	18	IVW	
		T2DM (OR)	0.96 (0.95; 0.97)	2.3×10^{-10}	0.705	16	IVW	
		BMI (SD)	0.01 (-0.00; 0.02)	2.1×10^{-1}	0.011	14	MR Egger	
		SBP (mmHg)	-0.00 (-0.15; 0.14)	9.9×10^{-1}	0.414	17	MR Egger	
		DBP (mmHg)	0.05 (-0.05; 0.15)	3.5×10^{-1}	0.066	17	MR Egger	
		ECG heart rate (exercise) (BPM)	0.10 (-0.35; 0.56)	6.6×10^{-1}	0.484	18	MR Egger	
		ECG load (exercise) (Watts)	1.05 (0.83; 1.27)	1.0×10^{-100}	0.004	18	IVW	
		Asthma (OR)	0.97 (0.94; 1.00)	5.9×10^{-2}	0.978	17	MR Egger	
		CRP (log(mg/L))	0.02 (0.01; 0.03)	2.6×10^{-3}	0.148	14	IVW	
		eGFR (SD of log(eGFR))	-0.00 (-0.00; 0.00)	3.4×10^{-1}	0.562	18	MR Egger	
		BUN (mg/dl)	-0.01 (-0.01; -0.00)	4.5×10^{-11}	0.333	15	IVW	
		CKD (OR)	0.97 (0.95; 0.99)	4.2×10^{-3}	<0.001	16	IVW	
		Alzheimer’s (OR)	1.00 (0.99; 1.00)	6.2×10^{-1}	0.090	17	IVW	
		Parkinson’s (OR)	0.96 (0.91; 1.01)	1.2×10^{-1}	0.136	16	IVW	
		Lewy body dementia (OR)	0.93 (0.73; 1.20)	5.8×10^{-1}	0.361	13	MR Egger	
		Breast cancer (OR)	0.98 (0.91; 1.05)	5.4×10^{-1}	0.094	17	MR Egger	
		Lung cancer (OR)	0.94 (0.88; 1.01)	7.4×10^{-2}	0.663	18	IVW	

Table S16: Phenome-wide drug target MR scan of proteins with a prioritized CMR association. *(continued)*

Protein (uniprot)	Protein set	Trait	Estimate (95%CI)	P-value	Q p-value	No. variants	MR model	pQTL source
		Colon cancer (OR)	1.01 (0.96; 1.08)	6.4×10^{-1}	0.712	14	IVW	
		Prostate cancer (OR)	0.99 (0.91; 1.06)	7.0×10^{-1}	0.045	16	MR Egger	
TNF12 (O43508)	Druggable	HF (OR)	0.87 (0.82; 0.93)	5.6×10^{-5}	0.017	13	MR Egger	Interval
		Non-ischemic CM (OR)	0.82 (0.77; 0.88)	4.4×10^{-8}	0.030	13	IVW	
		DCM (OR)	0.80 (0.75; 0.85)	3.1×10^{-13}	0.976	14	IVW	
		AF (OR)	0.90 (0.89; 0.91)	1.0×10^{-100}	0.006	15	IVW	
		CHD (OR)	0.89 (0.82; 0.95)	1.4×10^{-3}	0.018	14	MR Egger	
		cIMT (mm)	0.00 (-0.00; 0.00)	4.0×10^{-1}	<0.001	14	IVW	
		Carotid plaque (OR)	0.94 (0.92; 0.97)	6.2×10^{-5}	0.190	15	IVW	
		Any stroke (OR)	0.96 (0.95; 0.97)	1.1×10^{-9}	0.003	15	IVW	
		Any ischemic stroke (OR)	0.96 (0.94; 0.98)	3.7×10^{-6}	0.008	13	IVW	
		LDL cholesterol (mmol/l)	-0.01 (-0.01; -0.01)	2.5×10^{-9}	0.026	9	IVW	
		Apoliprotein B (g/l)	-0.00 (-0.01; -0.00)	1.0×10^{-100}	<0.001	9	IVW	
		Triglycerides (mmol/l)	-0.02 (-0.03; -0.02)	1.0×10^{-100}	<0.001	11	IVW	
		Cholesterol (mmol/l)	0.06 (0.04; 0.07)	3.4×10^{-11}	0.031	11	MR Egger	
		HDL cholesterol (mmol/l)	0.01 (0.00; 0.01)	1.9×10^{-10}	<0.001	10	IVW	
		Apoliprotein A1 (g/l)	0.02 (0.01; 0.03)	6.2×10^{-6}	0.183	9	MR Egger	
		Glucose (mmol/l)	-0.01 (-0.03; 0.00)	1.3×10^{-1}	0.007	15	MR Egger	
		Glycated haemoglobin (mmol/mol)	-0.25 (-0.28; -0.22)	1.0×10^{-100}	0.002	11	IVW	
		T2DM (OR)	0.95 (0.94; 0.96)	1.8×10^{-13}	0.017	12	IVW	
		BMI (SD)	0.00 (-0.01; 0.02)	5.6×10^{-1}	0.011	13	MR Egger	
		SBP (mmHg)	1.44 (1.13; 1.74)	1.0×10^{-100}	<0.001	8	MR Egger	
		DBP (mmHg)	0.16 (0.12; 0.19)	1.0×10^{-100}	<0.001	8	IVW	
		ECG heart rate (exercise) (BPM)	-0.81 (-0.94; -0.67)	1.0×10^{-100}	0.255	16	IVW	
		ECG load (exercise) (Watts)	-0.69 (-0.83; -0.55)	1.0×10^{-100}	0.002	16	IVW	
		Asthma (OR)	0.93 (0.92; 0.94)	1.0×10^{-100}	<0.001	13	IVW	
		CRP (log(mg/L))	0.02 (0.01; 0.02)	1.2×10^{-9}	0.024	15	IVW	
		eGFR (SD of log(eGFR))	0.00 (0.00; 0.00)	7.9×10^{-12}	0.012	10	IVW	
		BUN (mg/dl)	-0.00 (-0.01; -0.00)	2.0×10^{-4}	0.840	11	IVW	
		CKD (OR)	0.96 (0.95; 0.98)	3.9×10^{-6}	<0.001	13	IVW	
		Alzheimer’s (OR)	1.00 (1.00; 1.01)	1.5×10^{-2}	0.028	14	IVW	
		Parkinson’s (OR)	0.99 (0.84; 1.17)	9.0×10^{-1}	0.034	12	MR Egger	
		Lewy body dementia (OR)	1.11 (1.06; 1.16)	1.9×10^{-5}	0.844	13	IVW	
		Breast cancer (OR)	1.34 (1.21; 1.48)	3.2×10^{-8}	0.025	13	MR Egger	
		Lung cancer (OR)	1.37 (1.01; 1.87)	4.6×10^{-2}	0.191	13	MR Egger	
		Colon cancer (OR)	1.02 (0.97; 1.07)	4.0×10^{-1}	0.139	13	IVW	
		Prostate cancer (OR)	0.82 (0.62; 1.08)	1.6×10^{-1}	0.115	10	MR Egger	
TNR5 (P25942)	Druggable	HF (OR)	0.98 (0.98; 0.99)	2.0×10^{-3}	<0.001	45	IVW	Scallop
		Non-ischemic CM (OR)	0.92 (0.89; 0.96)	9.1×10^{-5}	0.006	48	IVW	
		DCM (OR)	1.22 (1.12; 1.33)	6.1×10^{-6}	<0.001	48	MR Egger	
		AF (OR)	0.95 (0.93; 0.98)	1.0×10^{-4}	0.004	45	MR Egger	
		CHD (OR)	1.03 (1.02; 1.05)	3.6×10^{-5}	<0.001	35	IVW	
		cIMT (mm)	0.00 (-0.00; 0.00)	1.2×10^{-1}	<0.001	44	IVW	
		Carotid plaque (OR)	1.05 (1.03; 1.07)	4.4×10^{-5}	0.008	42	IVW	
		Any stroke (OR)	0.92 (0.91; 0.93)	1.0×10^{-100}	<0.001	45	IVW	
		Any ischemic stroke (OR)	0.91 (0.90; 0.92)	1.0×10^{-100}	0.081	42	IVW	
		LDL cholesterol (mmol/l)	0.01 (-0.00; 0.01)	1.3×10^{-1}	<0.001	39	MR Egger	
		Apoliprotein B (g/l)	0.01 (0.00; 0.01)	1.9×10^{-4}	<0.001	22	MR Egger	
		Triglycerides (mmol/l)	0.01 (-0.00; 0.03)	1.4×10^{-1}	<0.001	18	MR Egger	
		Cholesterol (mmol/l)	0.02 (0.01; 0.03)	1.5×10^{-5}	<0.001	44	MR Egger	
		HDL cholesterol (mmol/l)	0.01 (-0.00; 0.01)	9.7×10^{-2}	<0.001	22	MR Egger	
		Apoliprotein A1 (g/l)	0.00 (0.00; 0.01)	1.1×10^{-9}	<0.001	33	IVW	
		Glucose (mmol/l)	-0.01 (-0.01; -0.00)	2.9×10^{-2}	<0.001	47	IVW	
		Glycated haemoglobin (mmol/mol)	0.20 (0.11; 0.28)	4.3×10^{-6}	<0.001	36	MR Egger	
		T2DM (OR)	0.96 (0.95; 0.97)	1.0×10^{-100}	<0.001	45	IVW	
		BMI (SD)	-0.01 (-0.02; -0.01)	4.4×10^{-16}	<0.001	39	IVW	
		SBP (mmHg)	-0.09 (-0.18; -0.00)	4.7×10^{-2}	<0.001	37	MR Egger	
		DBP (mmHg)	0.01 (-0.03; 0.06)	6.1×10^{-1}	<0.001	37	MR Egger	
		ECG heart rate (exercise) (BPM)	-0.46 (-0.60; -0.31)	6.0×10^{-10}	<0.001	43	IVW	
		ECG load (exercise) (Watts)	-1.11 (-1.44; -0.77)	7.3×10^{-11}	0.299	46	MR Egger	
		Asthma (OR)	1.06 (1.04; 1.08)	1.1×10^{-7}	<0.001	43	MR Egger	
		CRP (log(mg/L))	0.01 (-0.00; 0.02)	1.7×10^{-1}	<0.001	44	MR Egger	
		eGFR (SD of log(eGFR))	0.00 (0.00; 0.00)	4.5×10^{-3}	<0.001	49	IVW	
		BUN (mg/dl)	-0.00 (-0.00; -0.00)	1.7×10^{-2}	0.003	43	MR Egger	
		CKD (OR)	0.97 (0.95; 0.99)	6.4×10^{-3}	<0.001	47	MR Egger	
		Alzheimer’s (OR)	1.00 (0.99; 1.01)	9.5×10^{-1}	0.016	48	MR Egger	

Table S16: Phenome-wide drug target MR scan of proteins with a prioritized CMR association. *(continued)*

Protein (uniprot)	Protein set	Trait	Estimate (95%CI)	P-value	Q p-value	No. variants	MR model	pQTL source
		Parkinson’s (OR)	0.90 (0.85; 0.96)	1.6×10^{-3}	0.055	46	MR Egger	
		Lewy body dementia (OR)	1.07 (0.98; 1.17)	1.5×10^{-1}	0.121	46	MR Egger	
		Breast cancer (OR)	1.09 (1.04; 1.14)	3.0×10^{-4}	<0.001	39	MR Egger	
		Lung cancer (OR)	1.18 (1.11; 1.26)	2.9×10^{-7}	0.064	39	IVW	
		Colon cancer (OR)	1.05 (0.98; 1.14)	1.8×10^{-1}	<0.001	45	MR Egger	
		Prostate cancer (OR)	1.01 (0.99; 1.03)	4.8×10^{-1}	0.008	46	IVW	
TR10B (O14763)	Druggable	HF (OR)	0.97 (0.93; 1.02)	2.9×10^{-1}	0.015	33	MR Egger	Scallop
		Non-ischemic CM (OR)	1.14 (0.91; 1.45)	2.6×10^{-1}	0.106	33	MR Egger	
		DCM (OR)	1.12 (1.02; 1.24)	1.9×10^{-2}	<0.001	30	IVW	
		AF (OR)	1.01 (0.99; 1.02)	3.4×10^{-1}	0.417	41	IVW	
		CHD (OR)	1.04 (0.99; 1.09)	1.1×10^{-1}	0.321	36	MR Egger	
		cIMT (mm)	0.01 (0.00; 0.01)	5.4×10^{-9}	0.075	32	IVW	
		Carotid plaque (OR)	1.06 (1.03; 1.10)	5.8×10^{-4}	0.382	38	IVW	
		Any stroke (OR)	0.93 (0.89; 0.98)	8.1×10^{-3}	0.679	37	MR Egger	
		Any ischemic stroke (OR)	0.95 (0.90; 1.01)	9.5×10^{-2}	0.849	37	MR Egger	
		LDL cholesterol (mmol/l)	0.00 (-0.00; 0.01)	6.9×10^{-1}	0.308	36	IVW	
		Apoliprotein B (g/l)	-0.00 (-0.00; 0.00)	2.8×10^{-1}	0.127	36	IVW	
		Triglycerides (mmol/l)	0.00 (-0.01; 0.02)	6.6×10^{-1}	<0.001	35	MR Egger	
		Cholesterol (mmol/l)	0.00 (-0.00; 0.01)	2.7×10^{-1}	0.023	35	IVW	
		HDL cholesterol (mmol/l)	0.00 (0.00; 0.01)	6.1×10^{-3}	0.004	35	IVW	
		Apoliprotein A1 (g/l)	-0.00 (-0.01; 0.00)	7.4×10^{-1}	0.107	38	MR Egger	
		Glucose (mmol/l)	-0.02 (-0.03; -0.01)	9.0×10^{-7}	<0.001	34	IVW	
		Glycated haemoglobin (mmol/mol)	-0.16 (-0.20; -0.11)	9.2×10^{-11}	0.162	35	IVW	
		T2DM (OR)	0.95 (0.94; 0.96)	2.5×10^{-13}	<0.001	36	IVW	
		BMI (SD)	-0.01 (-0.01; -0.00)	1.3×10^{-3}	0.008	34	IVW	
		SBP (mmHg)	0.08 (0.00; 0.16)	4.8×10^{-2}	0.052	32	IVW	
		DBP (mmHg)	0.05 (0.00; 0.09)	4.5×10^{-2}	0.086	33	IVW	
		ECG heart rate (exercise) (BPM)	0.23 (0.00; 0.46)	4.9×10^{-2}	0.013	36	IVW	
		ECG load (exercise) (Watts)	0.28 (0.02; 0.53)	3.2×10^{-2}	0.187	40	IVW	
		Asthma (OR)	0.91 (0.88; 0.94)	1.9×10^{-8}	<0.001	38	MR Egger	
		CRP (log(mg/L))	-0.03 (-0.05; -0.01)	1.1×10^{-2}	<0.001	36	MR Egger	
		eGFR (SD of log(eGFR))	0.00 (-0.00; 0.00)	7.7×10^{-1}	0.115	34	MR Egger	
		BUN (mg/dl)	-0.00 (-0.01; 0.00)	6.3×10^{-1}	0.073	33	MR Egger	
		CKD (OR)	1.00 (0.95; 1.05)	9.2×10^{-1}	0.017	36	MR Egger	
		Alzheimer’s (OR)	0.99 (0.98; 1.00)	1.7×10^{-1}	<0.001	33	MR Egger	
		Parkinson’s (OR)	1.04 (0.91; 1.19)	5.4×10^{-1}	0.066	34	MR Egger	
		Lewy body dementia (OR)	0.85 (0.67; 1.09)	2.0×10^{-1}	0.012	32	MR Egger	
		Breast cancer (OR)	1.00 (0.93; 1.06)	9.0×10^{-1}	0.603	38	MR Egger	
		Lung cancer (OR)	0.73 (0.61; 0.86)	2.4×10^{-4}	0.825	38	MR Egger	
		Colon cancer (OR)	1.00 (0.86; 1.15)	9.8×10^{-1}	0.232	36	MR Egger	
		Prostate cancer (OR)	0.89 (0.86; 0.93)	5.6×10^{-10}	<0.001	35	IVW	
ASAH2 (Q9NR71)	Concordant	HF (OR)	1.00 (0.97; 1.03)	9.9×10^{-1}	0.800	19	MR Egger	Interval
		Non-ischemic CM (OR)	0.96 (0.90; 1.01)	1.4×10^{-1}	0.148	21	IVW	
		DCM (OR)	1.08 (0.94; 1.25)	2.6×10^{-1}	0.612	19	MR Egger	
		AF (OR)	1.00 (0.99; 1.02)	8.5×10^{-1}	0.054	22	IVW	
		CHD (OR)	1.00 (0.98; 1.03)	7.4×10^{-1}	0.544	22	MR Egger	
		cIMT (mm)	0.00 (0.00; 0.01)	3.8×10^{-8}	0.218	19	IVW	
		Carotid plaque (OR)	1.05 (1.02; 1.09)	2.3×10^{-3}	0.134	17	IVW	
		Any stroke (OR)	0.97 (0.96; 0.99)	3.2×10^{-4}	0.476	19	IVW	
		Any ischemic stroke (OR)	0.97 (0.95; 0.99)	5.7×10^{-4}	0.023	20	IVW	
		LDL cholesterol (mmol/l)	-0.02 (-0.02; -0.01)	6.3×10^{-10}	0.738	21	MR Egger	
		Apoliprotein B (g/l)	-0.00 (-0.01; -0.00)	4.0×10^{-11}	0.615	21	MR Egger	
		Triglycerides (mmol/l)	-0.01 (-0.02; -0.01)	3.1×10^{-4}	0.041	21	MR Egger	
		Cholesterol (mmol/l)	-0.01 (-0.02; -0.01)	2.1×10^{-5}	0.884	21	MR Egger	
		HDL cholesterol (mmol/l)	-0.00 (-0.00; -0.00)	2.1×10^{-5}	0.013	22	IVW	
		Apoliprotein A1 (g/l)	0.00 (-0.00; 0.00)	8.5×10^{-1}	0.027	23	IVW	
		Glucose (mmol/l)	-0.01 (-0.01; -0.00)	4.7×10^{-5}	0.396	24	IVW	
		Glycated haemoglobin (mmol/mol)	-0.03 (-0.07; 0.01)	1.7×10^{-1}	0.259	22	MR Egger	
		T2DM (OR)	1.00 (0.99; 1.01)	8.7×10^{-1}	0.324	19	IVW	
		BMI (SD)	-0.00 (-0.01; 0.00)	3.1×10^{-1}	0.231	15	MR Egger	
		SBP (mmHg)	0.01 (-0.13; 0.14)	9.4×10^{-1}	0.467	16	MR Egger	
		DBP (mmHg)	0.02 (-0.05; 0.10)	5.8×10^{-1}	0.418	18	MR Egger	
		ECG heart rate (exercise) (BPM)	0.03 (-0.06; 0.12)	5.0×10^{-1}	<0.001	22	IVW	
		ECG load (exercise) (Watts)	0.23 (-0.00; 0.46)	5.2×10^{-2}	0.431	22	MR Egger	
		Asthma (OR)	0.96 (0.95; 0.98)	1.3×10^{-6}	0.265	23	MR Egger	
		CRP (log(mg/L))	-0.00 (-0.03; 0.02)	7.2×10^{-1}	0.107	18	MR Egger	

Table S16: Phenome-wide drug target MR scan of proteins with a prioritized CMR association. *(continued)*

Protein (uniprot)	Protein set	Trait	Estimate (95%CI)	P-value	Q p-value	No. variants	MR model	pQTL source
		eGFR (SD of log(eGFR))	0.00 (-0.00; 0.00)	9.2×10^{-1}	0.138	16	MR Egger	
		BUN (mg/dl)	-0.00 (-0.01; 0.00)	2.2×10^{-1}	0.572	18	MR Egger	
		CKD (OR)	1.03 (1.00; 1.06)	2.2×10^{-2}	0.185	16	IVW	
		Alzheimer's (OR)	1.00 (0.99; 1.00)	8.4×10^{-1}	0.021	17	IVW	
		Parkinson's (OR)	0.92 (0.88; 0.97)	9.0×10^{-4}	0.114	17	IVW	
		Lewy body dementia (OR)	0.93 (0.88; 0.99)	1.6×10^{-2}	0.045	18	IVW	
		Breast cancer (OR)	0.99 (0.97; 1.02)	5.4×10^{-1}	0.012	17	IVW	
		Lung cancer (OR)	1.05 (0.98; 1.13)	1.5×10^{-1}	0.092	20	IVW	
		Colon cancer (OR)	0.95 (0.91; 1.00)	4.2×10^{-2}	0.008	18	IVW	
		Prostate cancer (OR)	0.94 (0.90; 0.97)	4.9×10^{-4}	0.127	18	IVW	
ASM3A (Q92484)	Concordant	HF (OR)	0.98 (0.95; 1.01)	2.0×10^{-1}	0.023	26	MR Egger	Interval
		Non-ischemic CM (OR)	0.94 (0.90; 0.98)	6.3×10^{-3}	0.197	31	IVW	
		DCM (OR)	1.04 (0.99; 1.10)	1.3×10^{-1}	0.422	31	IVW	
		AF (OR)	1.02 (1.01; 1.03)	1.0×10^{-4}	0.040	27	IVW	
		CHD (OR)	0.99 (0.98; 1.00)	5.6×10^{-2}	0.416	27	IVW	
		cIMT (mm)	0.00 (-0.00; 0.00)	1.1×10^{-1}	<0.001	29	IVW	
		Carotid plaque (OR)	1.01 (0.98; 1.03)	5.5×10^{-1}	0.367	29	IVW	
		Any stroke (OR)	0.99 (0.97; 1.01)	4.3×10^{-1}	0.390	26	IVW	
		Any ischemic stroke (OR)	1.01 (0.98; 1.03)	5.1×10^{-1}	0.394	24	IVW	
		LDL cholesterol (mmol/l)	0.00 (-0.00; 0.00)	7.2×10^{-1}	0.104	39	IVW	
		Apoliprotein B (g/l)	-0.00 (-0.00; 0.00)	2.2×10^{-1}	0.350	39	IVW	
		Triglycerides (mmol/l)	0.00 (-0.00; 0.00)	1.2×10^{-1}	0.029	37	IVW	
		Cholesterol (mmol/l)	0.00 (-0.00; 0.00)	1.5×10^{-1}	0.038	39	IVW	
		HDL cholesterol (mmol/l)	0.00 (-0.00; 0.00)	5.5×10^{-1}	0.393	34	MR Egger	
		Apoliprotein A1 (g/l)	-0.00 (-0.00; 0.00)	9.8×10^{-1}	0.311	35	MR Egger	
		Glucose (mmol/l)	-0.00 (-0.01; 0.01)	8.1×10^{-1}	0.002	30	MR Egger	
		Glycated haemoglobin (mmol/mol)	-0.04 (-0.06; -0.02)	3.9×10^{-7}	<0.001	38	IVW	
		T2DM (OR)	0.99 (0.98; 1.00)	1.5×10^{-1}	0.185	31	IVW	
		BMI (SD)	0.01 (0.01; 0.01)	1.2×10^{-11}	0.010	30	IVW	
		SBP (mmHg)	0.04 (-0.01; 0.08)	9.2×10^{-2}	<0.001	28	IVW	
		DBP (mmHg)	0.05 (-0.01; 0.11)	1.3×10^{-1}	0.100	27	MR Egger	
		ECG heart rate (exercise) (BPM)	0.32 (0.07; 0.57)	1.2×10^{-2}	0.362	37	MR Egger	
		ECG load (exercise) (Watts)	0.17 (-0.15; 0.49)	3.0×10^{-1}	0.062	39	MR Egger	
		Asthma (OR)	1.02 (1.01; 1.03)	2.5×10^{-8}	0.059	39	IVW	
		CRP (log(mg/L))	-0.02 (-0.03; 0.00)	1.1×10^{-1}	0.760	30	MR Egger	
		eGFR (SD of log(eGFR))	0.00 (-0.00; 0.00)	4.8×10^{-1}	0.191	32	IVW	
		BUN (mg/dl)	-0.00 (-0.00; 0.00)	2.0×10^{-1}	0.004	29	IVW	
		CKD (OR)	0.97 (0.93; 1.00)	6.5×10^{-2}	0.873	30	MR Egger	
		Alzheimer's (OR)	1.01 (1.00; 1.02)	7.0×10^{-4}	<0.001	33	MR Egger	
		Parkinson's (OR)	0.95 (0.88; 1.01)	1.1×10^{-1}	0.012	30	MR Egger	
		Lewy body dementia (OR)	1.02 (0.91; 1.15)	7.4×10^{-1}	0.119	28	MR Egger	
		Breast cancer (OR)	1.06 (0.99; 1.13)	7.8×10^{-2}	0.018	26	MR Egger	
		Lung cancer (OR)	0.93 (0.79; 1.10)	3.9×10^{-1}	0.571	31	MR Egger	
		Colon cancer (OR)	0.95 (0.93; 0.98)	1.5×10^{-3}	0.029	31	IVW	
		Prostate cancer (OR)	0.99 (0.97; 1.01)	1.9×10^{-1}	0.260	33	IVW	
BAG3 (O95817)	Concordant	HF (OR)	0.75 (0.72; 0.79)	1.0×10^{-100}	0.900	5	IVW	Interval
		Non-ischemic CM (OR)	0.30 (0.25; 0.36)	1.0×10^{-100}	0.481	5	IVW	
		DCM (OR)	0.14 (0.11; 0.17)	1.0×10^{-100}	0.032	4	IVW	
		AF (OR)	1.01 (0.96; 1.07)	6.3×10^{-1}	0.135	5	IVW	
		CHD (OR)	0.97 (0.92; 1.03)	3.4×10^{-1}	0.217	5	IVW	
		cIMT (mm)	0.00 (-0.00; 0.01)	9.5×10^{-2}	0.581	5	IVW	
		Carotid plaque (OR)	1.16 (1.03; 1.30)	1.3×10^{-2}	0.126	5	IVW	
		Any stroke (OR)	1.18 (0.87; 1.62)	2.9×10^{-1}	0.749	5	MR Egger	
		Any ischemic stroke (OR)	1.11 (0.79; 1.56)	5.5×10^{-1}	0.490	5	MR Egger	
		LDL cholesterol (mmol/l)	-0.08 (-0.14; -0.01)	1.6×10^{-2}	0.919	5	MR Egger	
		Apoliprotein B (g/l)	-0.03 (-0.04; -0.01)	4.4×10^{-3}	0.826	5	MR Egger	
		Triglycerides (mmol/l)	-0.01 (-0.03; 0.01)	3.0×10^{-1}	0.122	5	IVW	
		Cholesterol (mmol/l)	-0.09 (-0.17; -0.01)	2.9×10^{-2}	0.736	5	MR Egger	
		HDL cholesterol (mmol/l)	0.02 (0.01; 0.02)	4.2×10^{-7}	0.109	5	IVW	
		Apoliprotein A1 (g/l)	-0.00 (-0.02; 0.02)	9.3×10^{-1}	0.881	5	MR Egger	
		Glucose (mmol/l)	0.01 (-0.00; 0.03)	7.6×10^{-2}	0.921	5	IVW	
		Glycated haemoglobin (mmol/mol)	-0.05 (-0.14; 0.05)	3.3×10^{-1}	0.262	5	IVW	
		T2DM (OR)	0.89 (0.86; 0.92)	9.0×10^{-13}	0.448	5	IVW	
		BMI (SD)	0.02 (0.01; 0.03)	3.3×10^{-4}	0.011	5	IVW	
		SBP (mmHg)	-0.97 (-1.13; -0.82)	1.0×10^{-100}	0.017	5	IVW	
		DBP (mmHg)	-1.12 (-1.23; -1.02)	1.0×10^{-100}	0.017	4	IVW	

Table S16: Phenome-wide drug target MR scan of proteins with a prioritized CMR association. *(continued)*

Protein (uniprot)	Protein set	Trait	Estimate (95%CI)	P-value	Q p-value	No. variants	MR model	pQTL source
		ECG heart rate (exercise) (BPM)	-0.42 (-0.88; 0.04)	7.6×10^{-2}	0.447	5	IVW	
		ECG load (exercise) (Watts)	0.03 (-0.74; 0.81)	9.3×10^{-1}	0.062	5	IVW	
		Asthma (OR)	1.01 (0.98; 1.04)	6.9×10^{-1}	0.466	5	IVW	
		CRP (log(mg/L))	0.01 (-0.01; 0.03)	3.5×10^{-1}	0.334	5	IVW	
		eGFR (SD of log(eGFR))	0.00 (-0.00; 0.00)	5.7×10^{-1}	0.071	5	IVW	
		BUN (mg/dl)	-0.00 (-0.01; 0.00)	7.4×10^{-1}	0.310	5	IVW	
		CKD (OR)	1.06 (1.00; 1.11)	5.3×10^{-2}	0.345	5	IVW	
		Alzheimer’s (OR)	0.99 (0.97; 1.01)	4.6×10^{-1}	0.066	5	IVW	
		Parkinson’s (OR)	1.56 (1.42; 1.70)	1.0×10^{-100}	0.008	5	IVW	
		Lewy body dementia (OR)	1.18 (0.91; 1.54)	2.1×10^{-1}	0.403	4	IVW	
		Breast cancer (OR)	0.96 (0.91; 1.03)	2.5×10^{-1}	0.996	5	IVW	
		Lung cancer (OR)	0.85 (0.73; 1.00)	5.0×10^{-2}	0.485	5	IVW	
		Colon cancer (OR)	1.37 (1.19; 1.58)	1.9×10^{-5}	0.266	5	IVW	
		Prostate cancer (OR)	1.10 (1.02; 1.19)	1.4×10^{-2}	0.768	5	IVW	
BGH3 (Q15582)	Concordant	HF (OR)	1.01 (1.00; 1.02)	7.3×10^{-2}	0.482	16	IVW	Interval
		Non-ischemic CM (OR)	0.96 (0.91; 1.01)	8.0×10^{-2}	0.321	16	IVW	
		DCM (OR)	0.79 (0.53; 1.17)	2.4×10^{-1}	0.946	16	MR Egger	
		AF (OR)	0.98 (0.97; 0.99)	2.7×10^{-6}	0.842	16	IVW	
		CHD (OR)	0.98 (0.90; 1.07)	6.2×10^{-1}	0.703	16	MR Egger	
		cIMT (mm)	0.01 (-0.00; 0.01)	8.4×10^{-2}	0.569	16	MR Egger	
		Carotid plaque (OR)	1.02 (1.00; 1.05)	9.9×10^{-2}	0.586	15	IVW	
		Any stroke (OR)	1.02 (1.00; 1.04)	1.3×10^{-2}	0.085	16	IVW	
		Any ischemic stroke (OR)	1.01 (0.99; 1.02)	5.0×10^{-1}	0.619	16	IVW	
		LDL cholesterol (mmol/l)	0.02 (0.01; 0.02)	1.0×10^{-100}	0.079	18	IVW	
		Apoliprotein B (g/l)	0.00 (0.00; 0.00)	1.0×10^{-100}	0.222	18	IVW	
		Triglycerides (mmol/l)	-0.02 (-0.04; 0.00)	5.7×10^{-2}	0.242	19	MR Egger	
		Cholesterol (mmol/l)	0.02 (0.02; 0.02)	1.0×10^{-100}	0.009	19	IVW	
		HDL cholesterol (mmol/l)	0.00 (0.00; 0.01)	7.2×10^{-7}	0.015	14	IVW	
		Apoliprotein A1 (g/l)	0.00 (0.00; 0.00)	6.8×10^{-4}	0.051	15	IVW	
		Glucose (mmol/l)	-0.01 (-0.01; -0.01)	7.0×10^{-7}	0.321	19	IVW	
		Glycated haemoglobin (mmol/mol)	-0.06 (-0.08; -0.04)	2.8×10^{-8}	0.951	19	IVW	
		T2DM (OR)	0.96 (0.95; 0.97)	1.0×10^{-100}	<0.001	16	IVW	
		BMI (SD)	-0.01 (-0.01; -0.00)	2.1×10^{-4}	0.745	15	IVW	
		SBP (mmHg)	-0.08 (-0.41; 0.26)	6.5×10^{-1}	0.316	15	MR Egger	
		DBP (mmHg)	0.02 (-0.16; 0.20)	8.4×10^{-1}	0.586	15	MR Egger	
		ECG heart rate (exercise) (BPM)	-0.36 (-1.07; 0.35)	3.2×10^{-1}	0.430	19	MR Egger	
		ECG load (exercise) (Watts)	0.33 (-0.46; 1.12)	4.2×10^{-1}	0.562	19	MR Egger	
		Asthma (OR)	1.05 (1.00; 1.10)	5.0×10^{-2}	0.019	18	MR Egger	
		CRP (log(mg/L))	-0.01 (-0.01; 0.00)	7.5×10^{-2}	0.127	16	IVW	
		eGFR (SD of log(eGFR))	0.00 (-0.00; 0.00)	8.6×10^{-1}	0.751	14	IVW	
		BUN (mg/dl)	-0.01 (-0.01; -0.01)	1.0×10^{-100}	0.498	16	IVW	
		CKD (OR)	1.00 (0.99; 1.02)	6.4×10^{-1}	0.846	16	IVW	
		Alzheimer’s (OR)	1.00 (1.00; 1.01)	2.8×10^{-2}	0.668	15	IVW	
		Parkinson’s (OR)	1.08 (0.84; 1.38)	5.7×10^{-1}	0.225	16	MR Egger	
		Lewy body dementia (OR)	0.92 (0.88; 0.97)	2.8×10^{-3}	0.720	14	IVW	
		Breast cancer (OR)	1.01 (0.90; 1.14)	8.6×10^{-1}	0.320	15	MR Egger	
		Lung cancer (OR)	1.00 (0.95; 1.06)	9.7×10^{-1}	0.144	15	IVW	
		Colon cancer (OR)	1.01 (0.80; 1.28)	9.1×10^{-1}	0.772	14	MR Egger	
		Prostate cancer (OR)	1.00 (0.86; 1.17)	9.6×10^{-1}	0.346	15	MR Egger	
BSSP4 (Q9GZN4)	Concordant	HF (OR)	1.01 (0.95; 1.09)	6.9×10^{-1}	0.767	14	MR Egger	Interval
		Non-ischemic CM (OR)	1.01 (0.93; 1.10)	7.6×10^{-1}	0.751	15	IVW	
		DCM (OR)	0.97 (0.71; 1.33)	8.5×10^{-1}	0.789	14	MR Egger	
		AF (OR)	1.04 (0.97; 1.10)	2.6×10^{-1}	0.096	16	MR Egger	
		CHD (OR)	1.00 (0.98; 1.02)	9.4×10^{-1}	0.998	18	IVW	
		cIMT (mm)	-0.00 (-0.01; 0.00)	2.6×10^{-1}	0.599	13	MR Egger	
		Carotid plaque (OR)	1.00 (0.86; 1.15)	9.5×10^{-1}	0.752	13	MR Egger	
		Any stroke (OR)	0.95 (0.92; 0.97)	1.1×10^{-4}	0.012	15	IVW	
		Any ischemic stroke (OR)	0.90 (0.83; 0.98)	2.1×10^{-2}	0.330	14	MR Egger	
		LDL cholesterol (mmol/l)	-0.00 (-0.02; 0.01)	7.1×10^{-1}	0.331	18	MR Egger	
		Apoliprotein B (g/l)	-0.01 (-0.01; -0.00)	4.3×10^{-3}	0.212	19	MR Egger	
		Triglycerides (mmol/l)	-0.00 (-0.02; 0.02)	9.7×10^{-1}	0.423	18	MR Egger	
		Cholesterol (mmol/l)	-0.00 (-0.02; 0.01)	7.1×10^{-1}	0.233	19	MR Egger	
		HDL cholesterol (mmol/l)	0.00 (0.00; 0.01)	3.2×10^{-5}	0.176	17	IVW	
		Apoliprotein A1 (g/l)	0.01 (0.00; 0.01)	3.1×10^{-5}	0.491	19	MR Egger	
		Glucose (mmol/l)	-0.01 (-0.03; 0.01)	1.7×10^{-1}	0.387	19	MR Egger	
		Glycated haemoglobin (mmol/mol)	-0.03 (-0.14; 0.08)	5.7×10^{-1}	0.066	19	MR Egger	

Table S16: Phenome-wide drug target MR scan of proteins with a prioritized CMR association. *(continued)*

Protein (uniprot)	Protein set	Trait	Estimate (95%CI)	P-value	Q p-value	No. variants	MR model	pQTL source
		T2DM (OR)	0.98 (0.93; 1.04)	5.7×10^{-1}	0.045	13	MR Egger	
		BMI (SD)	0.01 (-0.02; 0.03)	6.0×10^{-1}	0.161	12	MR Egger	
		SBP (mmHg)	-0.12 (-0.21; -0.03)	6.2×10^{-3}	0.496	13	IVW	
		DBP (mmHg)	-0.05 (-0.26; 0.15)	6.0×10^{-1}	0.122	11	MR Egger	
		ECG heart rate (exercise) (BPM)	0.82 (0.20; 1.45)	9.9×10^{-3}	0.065	19	MR Egger	
		ECG load (exercise) (Watts)	0.08 (-0.08; 0.24)	3.1×10^{-1}	0.517	18	IVW	
		Asthma (OR)	None	None	None	None	None	
		CRP (log(mg/L))	0.04 (0.02; 0.05)	1.1×10^{-7}	0.627	13	IVW	
		eGFR (SD of log(eGFR))	-0.00 (-0.01; 0.00)	6.9×10^{-1}	0.084	12	MR Egger	
		BUN (mg/dl)	-0.01 (-0.02; -0.00)	5.0×10^{-2}	0.526	13	MR Egger	
		CKD (OR)	0.97 (0.94; 0.99)	1.0×10^{-2}	0.806	13	IVW	
		Alzheimer's (OR)	1.01 (0.99; 1.03)	3.0×10^{-1}	0.839	15	MR Egger	
		Parkinson's (OR)	0.83 (0.67; 1.01)	6.7×10^{-2}	0.010	13	MR Egger	
		Lewy body dementia (OR)	1.23 (0.87; 1.72)	2.4×10^{-1}	0.707	14	MR Egger	
		Breast cancer (OR)	0.99 (0.96; 1.01)	3.5×10^{-1}	0.460	14	IVW	
		Lung cancer (OR)	0.98 (0.91; 1.06)	6.4×10^{-1}	0.476	13	IVW	
		Colon cancer (OR)	0.98 (0.92; 1.03)	3.9×10^{-1}	0.398	15	IVW	
		Prostate cancer (OR)	1.11 (0.99; 1.26)	7.8×10^{-2}	0.013	14	MR Egger	
C1QC (P02747)	Concordant	HF (OR)	0.97 (0.96; 0.98)	1.5×10^{-7}	0.002	36	IVW	Interval
		Non-ischemic CM (OR)	0.95 (0.85; 1.07)	4.3×10^{-1}	0.032	38	MR Egger	
		DCM (OR)	0.86 (0.82; 0.90)	5.2×10^{-11}	0.044	40	IVW	
		AF (OR)	0.97 (0.95; 1.00)	2.6×10^{-2}	0.026	42	MR Egger	
		CHD (OR)	0.98 (0.96; 1.01)	2.7×10^{-1}	0.040	37	MR Egger	
		cIMT (mm)	-0.00 (-0.00; 0.00)	7.6×10^{-1}	0.021	37	MR Egger	
		Carotid plaque (OR)	1.05 (1.03; 1.08)	3.2×10^{-7}	0.584	38	IVW	
		Any stroke (OR)	1.01 (1.00; 1.02)	4.5×10^{-2}	0.459	38	IVW	
		Any ischemic stroke (OR)	0.98 (0.95; 1.02)	3.7×10^{-1}	0.129	41	MR Egger	
		LDL cholesterol (mmol/l)	-0.00 (-0.01; 0.00)	2.7×10^{-1}	0.008	43	MR Egger	
		Apoliprotein B (g/l)	-0.00 (-0.00; 0.00)	1.2×10^{-1}	0.010	43	MR Egger	
		Triglycerides (mmol/l)	-0.00 (-0.01; 0.00)	3.4×10^{-1}	0.530	43	MR Egger	
		Cholesterol (mmol/l)	-0.00 (-0.01; 0.01)	8.0×10^{-1}	0.004	43	MR Egger	
		HDL cholesterol (mmol/l)	0.00 (0.00; 0.01)	4.7×10^{-2}	0.495	39	MR Egger	
		Apoliprotein A1 (g/l)	0.00 (0.00; 0.00)	4.3×10^{-2}	0.269	39	MR Egger	
		Glucose (mmol/l)	-0.00 (-0.01; 0.01)	5.0×10^{-1}	0.023	39	MR Egger	
		Glycated haemoglobin (mmol/mol)	-0.01 (-0.03; 0.00)	1.3×10^{-1}	0.033	41	IVW	
		T2DM (OR)	1.01 (0.99; 1.03)	3.8×10^{-1}	<0.001	41	MR Egger	
		BMI (SD)	-0.01 (-0.02; -0.00)	6.9×10^{-3}	0.216	36	MR Egger	
		SBP (mmHg)	-0.12 (-0.16; -0.07)	2.1×10^{-7}	<0.001	33	IVW	
		DBP (mmHg)	-0.08 (-0.11; -0.05)	4.7×10^{-7}	0.253	28	IVW	
		ECG heart rate (exercise) (BPM)	0.21 (-0.07; 0.50)	1.4×10^{-1}	0.086	43	MR Egger	
		ECG load (exercise) (Watts)	-0.06 (-0.38; 0.26)	7.3×10^{-1}	0.014	41	MR Egger	
		Asthma (OR)	1.00 (0.99; 1.01)	5.4×10^{-1}	<0.001	37	IVW	
		CRP (log(mg/L))	-0.01 (-0.01; -0.00)	1.1×10^{-2}	0.002	40	IVW	
		eGFR (SD of log(eGFR))	0.00 (-0.00; 0.00)	3.0×10^{-1}	0.015	39	IVW	
		BUN (mg/dl)	-0.00 (-0.00; -0.00)	1.1×10^{-2}	0.025	36	IVW	
		CKD (OR)	1.02 (1.01; 1.04)	5.3×10^{-5}	0.045	36	IVW	
		Alzheimer's (OR)	1.00 (1.00; 1.01)	2.5×10^{-1}	0.013	41	MR Egger	
		Parkinson's (OR)	1.01 (0.95; 1.08)	7.3×10^{-1}	0.013	38	MR Egger	
		Lewy body dementia (OR)	1.01 (0.90; 1.14)	8.5×10^{-1}	0.002	38	MR Egger	
		Breast cancer (OR)	1.02 (1.00; 1.04)	2.3×10^{-2}	0.202	37	IVW	
		Lung cancer (OR)	0.99 (0.89; 1.09)	7.7×10^{-1}	0.005	39	MR Egger	
		Colon cancer (OR)	1.07 (1.03; 1.11)	1.4×10^{-4}	0.019	34	IVW	
		Prostate cancer (OR)	0.94 (0.90; 0.99)	1.8×10^{-2}	0.014	40	MR Egger	
CATB (P07858)	Concordant	HF (OR)	1.01 (0.95; 1.06)	8.5×10^{-1}	0.046	21	MR Egger	Interval
		Non-ischemic CM (OR)	1.12 (1.01; 1.23)	2.4×10^{-2}	<0.001	20	IVW	
		DCM (OR)	1.14 (1.03; 1.28)	1.6×10^{-2}	0.077	21	IVW	
		AF (OR)	1.05 (0.99; 1.10)	1.0×10^{-1}	0.013	19	MR Egger	
		CHD (OR)	1.07 (1.01; 1.13)	2.4×10^{-2}	0.337	22	MR Egger	
		cIMT (mm)	-0.00 (-0.00; -0.00)	4.2×10^{-2}	<0.001	16	IVW	
		Carotid plaque (OR)	0.96 (0.91; 1.01)	1.3×10^{-1}	0.050	18	IVW	
		Any stroke (OR)	0.99 (0.96; 1.02)	6.0×10^{-1}	0.233	20	IVW	
		Any ischemic stroke (OR)	0.99 (0.96; 1.02)	5.9×10^{-1}	0.330	20	IVW	
		LDL cholesterol (mmol/l)	0.04 (0.03; 0.05)	2.7×10^{-8}	<0.001	21	MR Egger	
		Apoliprotein B (g/l)	0.01 (0.00; 0.01)	3.9×10^{-5}	<0.001	18	MR Egger	
		Triglycerides (mmol/l)	0.07 (0.06; 0.07)	1.0×10^{-100}	<0.001	13	IVW	
		Cholesterol (mmol/l)	0.03 (0.03; 0.04)	1.0×10^{-100}	0.231	24	IVW	

Table S16: Phenome-wide drug target MR scan of proteins with a prioritized CMR association. *(continued)*

Protein (uniprot)	Protein set	Trait	Estimate (95%CI)	P-value	Q p-value	No. variants	MR model	pQTL source
		HDL cholesterol (mmol/l)	0.00 (0.00; 0.01)	4.4×10^{-7}	0.044	18	IVW	
		Apoliprotein A1 (g/l)	0.01 (0.00; 0.01)	4.4×10^{-3}	<0.001	19	MR Egger	
		Glucose (mmol/l)	-0.01 (-0.01; -0.00)	7.8×10^{-4}	0.188	26	IVW	
		Glycated haemoglobin (mmol/mol)	-0.05 (-0.09; -0.02)	2.6×10^{-3}	<0.001	19	IVW	
		T2DM (OR)	1.01 (0.95; 1.08)	7.6×10^{-1}	0.056	14	MR Egger	
		BMI (SD)	0.00 (-0.01; 0.01)	1.0×10^{0}	0.033	14	MR Egger	
		SBP (mmHg)	-0.41 (-0.66; -0.16)	1.2×10^{-3}	0.002	11	MR Egger	
		DBP (mmHg)	-0.29 (-0.35; -0.24)	1.0×10^{-100}	0.002	12	IVW	
		ECG heart rate (exercise) (BPM)	0.25 (0.10; 0.40)	1.1×10^{-3}	0.149	25	IVW	
		ECG load (exercise) (Watts)	-0.41 (-1.07; 0.24)	2.2×10^{-1}	<0.001	21	MR Egger	
		Asthma (OR)	1.01 (0.98; 1.05)	5.3×10^{-1}	0.339	25	MR Egger	
		CRP (log(mg/L))	-0.03 (-0.07; 0.00)	7.0×10^{-2}	0.002	12	MR Egger	
		eGFR (SD of log(eGFR))	-0.00 (-0.00; -0.00)	7.2×10^{-8}	0.078	17	IVW	
		BUN (mg/dl)	0.01 (-0.00; 0.01)	7.3×10^{-2}	0.087	23	MR Egger	
		CKD (OR)	1.01 (0.95; 1.08)	6.9×10^{-1}	0.009	22	MR Egger	
		Alzheimer's (OR)	0.98 (0.95; 1.00)	4.1×10^{-2}	0.226	17	MR Egger	
		Parkinson's (OR)	0.69 (0.60; 0.80)	6.8×10^{-7}	0.234	20	MR Egger	
		Lewy body dementia (OR)	0.89 (0.77; 1.03)	1.2×10^{-1}	0.125	15	IVW	
		Breast cancer (OR)	0.98 (0.89; 1.08)	7.5×10^{-1}	0.261	18	MR Egger	
		Lung cancer (OR)	1.00 (0.93; 1.09)	9.1×10^{-1}	0.843	20	IVW	
		Colon cancer (OR)	1.17 (1.00; 1.37)	5.3×10^{-2}	<0.001	21	MR Egger	
		Prostate cancer (OR)	0.96 (0.92; 1.00)	5.6×10^{-2}	<0.001	20	IVW	
ENTP1 (P49961)	Concordant	HF (OR)	1.00 (0.96; 1.04)	1.0×10^{0}	0.181	8	IVW	Interval
		Non-ischemic CM (OR)	0.84 (0.72; 0.97)	2.1×10^{-2}	0.370	8	IVW	
		DCM (OR)	1.24 (1.10; 1.40)	7.0×10^{-4}	0.778	9	IVW	
		AF (OR)	0.91 (0.78; 1.07)	2.5×10^{-1}	0.922	9	MR Egger	
		CHD (OR)	0.99 (0.89; 1.09)	8.2×10^{-1}	0.982	9	MR Egger	
		cIMT (mm)	0.00 (0.00; 0.01)	3.2×10^{-2}	0.182	7	IVW	
		Carotid plaque (OR)	0.94 (0.87; 1.00)	5.6×10^{-2}	0.767	8	IVW	
		Any stroke (OR)	1.19 (1.14; 1.23)	1.0×10^{-100}	0.514	8	IVW	
		Any ischemic stroke (OR)	1.18 (1.13; 1.23)	1.6×10^{-14}	0.499	8	IVW	
		LDL cholesterol (mmol/l)	-0.04 (-0.05; -0.04)	1.0×10^{-100}	0.172	10	IVW	
		Apoliprotein B (g/l)	-0.01 (-0.01; -0.01)	1.0×10^{-100}	0.029	10	IVW	
		Triglycerides (mmol/l)	-0.15 (-0.31; 0.00)	5.2×10^{-2}	0.651	6	MR Egger	
		Cholesterol (mmol/l)	-0.05 (-0.06; -0.05)	1.0×10^{-100}	0.221	10	IVW	
		HDL cholesterol (mmol/l)	-0.01 (-0.01; -0.01)	1.5×10^{-13}	0.167	10	IVW	
		Apoliprotein A1 (g/l)	-0.01 (-0.01; -0.01)	1.0×10^{-100}	0.936	10	IVW	
		Glucose (mmol/l)	-0.02 (-0.03; -0.01)	1.2×10^{-6}	0.126	9	IVW	
		Glycated haemoglobin (mmol/mol)	0.25 (-0.35; 0.86)	4.1×10^{-1}	0.074	8	MR Egger	
		T2DM (OR)	0.99 (0.93; 1.06)	8.7×10^{-1}	0.072	5	IVW	
		BMI (SD)	-0.00 (-0.01; 0.01)	4.8×10^{-1}	0.227	8	IVW	
		SBP (mmHg)	1.20 (0.56; 1.85)	2.7×10^{-4}	0.116	7	MR Egger	
		DBP (mmHg)	0.14 (-0.05; 0.34)	1.5×10^{-1}	0.213	4	IVW	
		ECG heart rate (exercise) (BPM)	-0.27 (-0.43; -0.11)	1.3×10^{-3}	0.971	10	IVW	
		ECG load (exercise) (Watts)	-0.27 (-0.45; -0.09)	3.8×10^{-3}	0.486	10	IVW	
		Asthma (OR)	1.10 (0.84; 1.46)	4.9×10^{-1}	0.123	6	MR Egger	
		CRP (log(mg/L))	-0.02 (-0.04; 0.01)	2.5×10^{-1}	0.187	7	IVW	
		eGFR (SD of log(eGFR))	0.00 (-0.00; 0.00)	8.6×10^{-1}	0.564	5	IVW	
		BUN (mg/dl)	0.00 (-0.01; 0.02)	8.4×10^{-1}	0.444	8	MR Egger	
		CKD (OR)	1.05 (0.99; 1.11)	1.3×10^{-1}	0.101	7	IVW	
		Alzheimer's (OR)	1.00 (0.99; 1.01)	9.4×10^{-1}	0.069	9	IVW	
		Parkinson's (OR)	1.04 (0.79; 1.37)	7.5×10^{-1}	0.859	8	MR Egger	
		Lewy body dementia (OR)	1.05 (0.87; 1.27)	6.0×10^{-1}	0.584	7	IVW	
		Breast cancer (OR)	1.01 (0.98; 1.05)	3.7×10^{-1}	0.800	9	IVW	
		Lung cancer (OR)	1.00 (0.92; 1.09)	9.8×10^{-1}	0.348	9	IVW	
		Colon cancer (OR)	1.06 (0.97; 1.16)	1.8×10^{-1}	0.081	9	IVW	
		Prostate cancer (OR)	0.98 (0.94; 1.02)	2.6×10^{-1}	0.454	9	IVW	
GPC5 (P78333)	Concordant	HF (OR)	1.01 (1.00; 1.03)	1.3×10^{-1}	0.336	65	MR Egger	Interval
		Non-ischemic CM (OR)	0.98 (0.95; 1.01)	1.8×10^{-1}	0.003	67	IVW	
		DCM (OR)	1.15 (1.11; 1.20)	5.8×10^{-12}	<0.001	62	IVW	
		AF (OR)	1.02 (1.00; 1.04)	1.8×10^{-2}	0.002	81	MR Egger	
		CHD (OR)	1.01 (0.99; 1.02)	5.2×10^{-1}	0.045	72	MR Egger	
		cIMT (mm)	0.00 (0.00; 0.00)	4.3×10^{-3}	0.696	69	MR Egger	
		Carotid plaque (OR)	1.02 (1.00; 1.04)	3.3×10^{-2}	0.017	62	IVW	
		Any stroke (OR)	1.00 (0.98; 1.02)	8.8×10^{-1}	0.447	66	MR Egger	
		Any ischemic stroke (OR)	0.98 (0.95; 1.00)	6.3×10^{-2}	0.188	67	MR Egger	

Table S16: Phenome-wide drug target MR scan of proteins with a prioritized CMR association. *(continued)*

Protein (uniprot)	Protein set	Trait	Estimate (95%CI)	P-value	Q p-value	No. variants	MR model	pQTL source
		LDL cholesterol (mmol/l)	-0.00 (-0.00; 0.00)	8.3×10^{-1}	0.033	83	MR Egger	
		Apoliprotein B (g/l)	-0.00 (-0.00; 0.00)	3.6×10^{-1}	0.102	82	MR Egger	
		Triglycerides (mmol/l)	0.01 (0.00; 0.01)	1.6×10^{-7}	<0.001	76	IVW	
		Cholesterol (mmol/l)	0.00 (0.00; 0.01)	1.1×10^{-2}	0.222	76	IVW	
		HDL cholesterol (mmol/l)	0.01 (0.01; 0.01)	2.7×10^{-15}	<0.001	77	MR Egger	
		Apoliprotein A1 (g/l)	0.00 (0.00; 0.00)	1.4×10^{-4}	<0.001	74	IVW	
		Glucose (mmol/l)	0.01 (0.01; 0.02)	5.4×10^{-4}	0.147	82	MR Egger	
		Glycated haemoglobin (mmol/mol)	0.03 (-0.01; 0.07)	9.3×10^{-2}	<0.001	79	MR Egger	
		T2DM (OR)	1.01 (0.99; 1.02)	3.6×10^{-1}	0.027	66	MR Egger	
		BMI (SD)	-0.00 (-0.00; -0.00)	2.0×10^{-2}	0.003	61	IVW	
		SBP (mmHg)	0.08 (0.05; 0.11)	9.9×10^{-7}	<0.001	57	IVW	
		DBP (mmHg)	0.08 (0.04; 0.11)	1.1×10^{-4}	0.301	61	MR Egger	
		ECG heart rate (exercise) (BPM)	-0.09 (-0.31; 0.12)	3.9×10^{-1}	0.165	81	MR Egger	
		ECG load (exercise) (Watts)	0.20 (-0.02; 0.42)	7.8×10^{-2}	0.005	83	MR Egger	
		Asthma (OR)	1.03 (1.02; 1.05)	2.3×10^{-5}	<0.001	81	MR Egger	
		CRP (log(mg/L))	0.01 (-0.00; 0.01)	2.8×10^{-1}	0.324	61	MR Egger	
		eGFR (SD of log(eGFR))	0.00 (0.00; 0.00)	4.7×10^{-4}	<0.001	65	IVW	
		BUN (mg/dl)	0.00 (-0.00; 0.00)	5.1×10^{-1}	0.003	63	IVW	
		CKD (OR)	0.97 (0.96; 0.98)	6.0×10^{-11}	0.393	66	IVW	
		Alzheimer's (OR)	1.00 (1.00; 1.01)	5.2×10^{-2}	<0.001	66	IVW	
		Parkinson's (OR)	1.01 (0.98; 1.03)	6.9×10^{-1}	0.053	65	IVW	
		Lewy body dementia (OR)	1.20 (1.13; 1.28)	5.0×10^{-9}	0.112	58	MR Egger	
		Breast cancer (OR)	1.01 (0.99; 1.02)	4.1×10^{-1}	0.009	75	IVW	
		Lung cancer (OR)	0.90 (0.85; 0.96)	1.6×10^{-3}	0.171	82	MR Egger	
		Colon cancer (OR)	0.88 (0.83; 0.93)	3.9×10^{-5}	0.057	79	MR Egger	
		Prostate cancer (OR)	0.98 (0.97; 1.00)	7.2×10^{-2}	<0.001	75	IVW	
IL18R (Q13478)	Concordant	HF (OR)	1.03 (1.01; 1.05)	5.5×10^{-3}	<0.001	32	MR Egger	Interval
		Non-ischemic CM (OR)	0.94 (0.91; 0.97)	1.6×10^{-5}	0.018	31	IVW	
		DCM (OR)	0.88 (0.83; 0.92)	2.1×10^{-7}	<0.001	29	IVW	
		AF (OR)	1.01 (1.00; 1.02)	1.6×10^{-1}	<0.001	33	IVW	
		CHD (OR)	1.03 (1.01; 1.05)	1.6×10^{-3}	0.005	36	MR Egger	
		cIMT (mm)	-0.00 (-0.01; -0.00)	1.1×10^{-4}	0.284	32	MR Egger	
		Carotid plaque (OR)	0.90 (0.86; 0.94)	3.4×10^{-7}	0.546	33	MR Egger	
		Any stroke (OR)	0.96 (0.93; 1.00)	3.3×10^{-2}	0.098	32	MR Egger	
		Any ischemic stroke (OR)	1.01 (0.97; 1.05)	6.7×10^{-1}	0.145	32	MR Egger	
		LDL cholesterol (mmol/l)	0.00 (-0.01; 0.01)	7.5×10^{-1}	0.626	37	MR Egger	
		Apoliprotein B (g/l)	0.00 (0.00; 0.00)	5.2×10^{-5}	<0.001	29	IVW	
		Triglycerides (mmol/l)	0.00 (-0.00; 0.01)	3.4×10^{-1}	<0.001	25	IVW	
		Cholesterol (mmol/l)	0.01 (0.01; 0.02)	4.5×10^{-5}	<0.001	40	MR Egger	
		HDL cholesterol (mmol/l)	0.00 (-0.00; 0.00)	6.8×10^{-1}	<0.001	33	IVW	
		Apoliprotein A1 (g/l)	-0.00 (-0.00; 0.00)	5.8×10^{-1}	<0.001	33	IVW	
		Glucose (mmol/l)	-0.00 (-0.01; 0.01)	7.3×10^{-1}	0.089	38	MR Egger	
		Glycated haemoglobin (mmol/mol)	0.00 (-0.04; 0.05)	8.3×10^{-1}	0.052	40	MR Egger	
		T2DM (OR)	1.03 (1.01; 1.05)	2.3×10^{-4}	0.336	31	MR Egger	
		BMI (SD)	0.01 (0.00; 0.01)	2.4×10^{-3}	0.011	29	MR Egger	
		SBP (mmHg)	-0.09 (-0.17; 0.00)	5.0×10^{-2}	0.176	30	MR Egger	
		DBP (mmHg)	0.02 (-0.02; 0.06)	3.7×10^{-1}	0.019	28	MR Egger	
		ECG heart rate (exercise) (BPM)	0.00 (-0.08; 0.08)	9.9×10^{-1}	0.193	38	IVW	
		ECG load (exercise) (Watts)	0.13 (-0.08; 0.33)	2.3×10^{-1}	0.798	41	MR Egger	
		Asthma (OR)	0.90 (0.86; 0.94)	5.4×10^{-6}	<0.001	15	MR Egger	
		CRP (log(mg/L))	0.04 (0.03; 0.05)	1.3×10^{-14}	0.009	21	IVW	
		eGFR (SD of log(eGFR))	0.00 (-0.00; 0.00)	8.9×10^{-1}	<0.001	18	IVW	
		BUN (mg/dl)	0.00 (0.00; 0.01)	3.5×10^{-3}	0.231	31	MR Egger	
		CKD (OR)	1.01 (0.96; 1.05)	8.0×10^{-1}	0.002	25	MR Egger	
		Alzheimer's (OR)	1.01 (1.00; 1.02)	1.2×10^{-1}	0.155	31	MR Egger	
		Parkinson's (OR)	0.92 (0.84; 1.01)	8.2×10^{-2}	0.112	28	MR Egger	
		Lewy body dementia (OR)	0.90 (0.82; 0.98)	2.1×10^{-2}	0.010	30	MR Egger	
		Breast cancer (OR)	0.99 (0.96; 1.02)	3.3×10^{-1}	0.041	37	MR Egger	
		Lung cancer (OR)	1.04 (1.01; 1.07)	4.0×10^{-3}	0.491	36	IVW	
		Colon cancer (OR)	0.96 (0.93; 0.99)	1.1×10^{-2}	<0.001	28	IVW	
		Prostate cancer (OR)	1.07 (1.03; 1.12)	7.6×10^{-4}	0.311	37	MR Egger	
ISK2 (P20155)	Concordant	HF (OR)	1.00 (0.97; 1.03)	9.3×10^{-1}	0.163	15	IVW	Interval
		Non-ischemic CM (OR)	0.98 (0.87; 1.09)	6.7×10^{-1}	0.756	15	IVW	
		DCM (OR)	0.99 (0.86; 1.14)	9.0×10^{-1}	0.977	15	IVW	
		AF (OR)	1.00 (0.98; 1.03)	8.4×10^{-1}	0.012	16	IVW	
		CHD (OR)	1.04 (1.00; 1.07)	3.5×10^{-2}	0.117	15	IVW	

Table S16: Phenome-wide drug target MR scan of proteins with a prioritized CMR association. *(continued)*

Protein (uniprot)	Protein set	Trait	Estimate (95%CI)	P-value	Q p-value	No. variants	MR model	pQTL source
		cIMT (mm)	0.00 (-0.00; 0.00)	8.1×10^{-1}	0.390	15	IVW	
		Carotid plaque (OR)	1.17 (1.10; 1.24)	2.7×10^{-7}	0.564	14	IVW	
		Any stroke (OR)	0.95 (0.84; 1.08)	4.6×10^{-1}	0.205	16	MR Egger	
		Any ischemic stroke (OR)	1.00 (0.96; 1.05)	9.0×10^{-1}	0.209	14	IVW	
		LDL cholesterol (mmol/l)	-0.01 (-0.02; -0.00)	2.3×10^{-2}	0.029	18	IVW	
		Apoliprotein B (g/l)	-0.00 (-0.01; -0.00)	9.1×10^{-3}	0.129	17	IVW	
		Triglycerides (mmol/l)	-0.01 (-0.02; -0.00)	5.9×10^{-3}	0.127	17	IVW	
		Cholesterol (mmol/l)	-0.01 (-0.02; 0.00)	1.7×10^{-1}	0.201	18	IVW	
		HDL cholesterol (mmol/l)	0.01 (-0.00; 0.02)	3.0×10^{-1}	0.389	20	MR Egger	
		Apoliprotein A1 (g/l)	0.01 (-0.00; 0.01)	8.2×10^{-2}	0.482	20	MR Egger	
		Glucose (mmol/l)	-0.01 (-0.02; -0.00)	7.6×10^{-3}	0.535	19	IVW	
		Glycated haemoglobin (mmol/mol)	0.01 (-0.04; 0.06)	7.4×10^{-1}	0.257	19	IVW	
		T2DM (OR)	1.01 (0.99; 1.04)	2.6×10^{-1}	0.192	15	IVW	
		BMI (SD)	-0.02 (-0.02; -0.01)	3.2×10^{-7}	0.867	15	IVW	
		SBP (mmHg)	-0.06 (-0.17; 0.04)	2.5×10^{-1}	0.363	15	IVW	
		DBP (mmHg)	-0.01 (-0.07; 0.05)	7.2×10^{-1}	0.537	15	IVW	
		ECG heart rate (exercise) (BPM)	-0.53 (-1.52; 0.45)	2.9×10^{-1}	0.500	20	MR Egger	
		ECG load (exercise) (Watts)	0.11 (-1.12; 1.34)	8.6×10^{-1}	0.221	20	MR Egger	
		Asthma (OR)	1.00 (0.98; 1.02)	8.9×10^{-1}	0.079	20	IVW	
		CRP (log(mg/L))	-0.01 (-0.02; 0.01)	4.8×10^{-1}	0.121	15	IVW	
		eGFR (SD of log(eGFR))	-0.00 (-0.00; -0.00)	5.3×10^{-3}	0.968	15	IVW	
		BUN (mg/dl)	0.01 (-0.00; 0.02)	5.7×10^{-2}	0.510	16	MR Egger	
		CKD (OR)	0.98 (0.94; 1.01)	1.4×10^{-1}	0.607	15	IVW	
		Alzheimer's (OR)	1.00 (0.97; 1.03)	9.8×10^{-1}	0.221	15	MR Egger	
		Parkinson's (OR)	0.98 (0.90; 1.06)	5.5×10^{-1}	0.942	15	IVW	
		Lewy body dementia (OR)	1.16 (1.03; 1.31)	1.2×10^{-2}	0.981	17	IVW	
		Breast cancer (OR)	0.98 (0.93; 1.02)	3.2×10^{-1}	0.198	15	IVW	
		Lung cancer (OR)	1.23 (0.87; 1.75)	2.4×10^{-1}	0.286	17	MR Egger	
		Colon cancer (OR)	0.90 (0.82; 0.99)	2.6×10^{-2}	0.128	17	IVW	
		Prostate cancer (OR)	1.02 (0.86; 1.20)	8.5×10^{-1}	0.657	17	MR Egger	
KAT3 (Q6YP21)	Concordant	HF (OR)	0.90 (0.83; 0.98)	1.1×10^{-2}	0.192	3	IVW	Interval
		Non-ischemic CM (OR)	0.99 (0.75; 1.29)	9.2×10^{-1}	0.803	3	IVW	
		DCM (OR)	1.30 (0.95; 1.78)	9.9×10^{-2}	0.791	3	IVW	
		AF (OR)	0.90 (0.85; 0.95)	8.1×10^{-5}	0.599	3	IVW	
		CHD (OR)	0.93 (0.87; 1.00)	4.4×10^{-2}	0.899	3	IVW	
		cIMT (mm)	-0.00 (-0.01; 0.01)	7.0×10^{-1}	0.155	3	IVW	
		Carotid plaque (OR)	0.86 (0.75; 0.99)	4.1×10^{-2}	0.334	3	IVW	
		Any stroke (OR)	0.91 (0.85; 0.98)	1.7×10^{-2}	0.740	3	IVW	
		Any ischemic stroke (OR)	0.89 (0.82; 0.97)	7.2×10^{-3}	0.842	3	IVW	
		LDL cholesterol (mmol/l)	0.03 (0.02; 0.05)	1.0×10^{-5}	0.200	4	IVW	
		Apoliprotein B (g/l)	0.01 (0.01; 0.01)	2.4×10^{-8}	0.432	4	IVW	
		Triglycerides (mmol/l)	-0.00 (-0.02; 0.01)	8.0×10^{-1}	0.883	4	IVW	
		Cholesterol (mmol/l)	0.05 (0.03; 0.06)	6.8×10^{-10}	0.440	4	IVW	
		HDL cholesterol (mmol/l)	0.15 (-0.03; 0.34)	1.1×10^{-1}	0.246	4	MR Egger	
		Apoliprotein A1 (g/l)	0.11 (-0.05; 0.28)	1.7×10^{-1}	0.109	4	MR Egger	
		Glucose (mmol/l)	-0.03 (-0.05; -0.01)	3.9×10^{-4}	0.949	4	IVW	
		Glycated haemoglobin (mmol/mol)	1.90 (-0.82; 4.63)	1.7×10^{-1}	0.761	4	MR Egger	
		T2DM (OR)	0.92 (0.87; 0.98)	7.5×10^{-3}	0.268	3	IVW	
		BMI (SD)	-0.05 (-0.06; -0.03)	7.9×10^{-10}	0.279	3	IVW	
		SBP (mmHg)	-2.18 (-2.57; -1.80)	1.0×10^{-100}	0.129	2	IVW	
		DBP (mmHg)	-0.67 (-0.95; -0.40)	1.8×10^{-6}	0.057	2	IVW	
		ECG heart rate (exercise) (BPM)	0.39 (-0.12; 0.91)	1.3×10^{-1}	0.425	4	IVW	
		ECG load (exercise) (Watts)	1.58 (1.01; 2.16)	6.8×10^{-8}	0.030	4	IVW	
		Asthma (OR)	0.75 (0.51; 1.11)	1.5×10^{-1}	0.975	5	MR Egger	
		CRP (log(mg/L))	-0.03 (-0.06; 0.01)	1.1×10^{-1}	0.572	3	IVW	
		eGFR (SD of log(eGFR))	0.01 (0.01; 0.02)	1.0×10^{-100}	0.365	3	IVW	
		BUN (mg/dl)	-0.02 (-0.03; -0.01)	2.6×10^{-5}	0.148	3	IVW	
		CKD (OR)	0.89 (0.82; 0.96)	2.9×10^{-3}	0.958	3	IVW	
		Alzheimer's (OR)	1.03 (1.01; 1.05)	9.5×10^{-3}	0.191	3	IVW	
		Parkinson's (OR)	1.07 (0.92; 1.24)	3.7×10^{-1}	0.840	3	IVW	
		Lewy body dementia (OR)	0.96 (0.70; 1.30)	7.8×10^{-1}	0.414	2	IVW	
		Breast cancer (OR)	1.08 (0.98; 1.20)	1.2×10^{-1}	0.276	3	IVW	
		Lung cancer (OR)	0.88 (0.70; 1.11)	2.7×10^{-1}	0.585	3	IVW	
		Colon cancer (OR)	1.37 (1.13; 1.65)	1.1×10^{-3}	0.659	3	IVW	
		Prostate cancer (OR)	1.04 (0.92; 1.17)	5.4×10^{-1}	0.715	3	IVW	
MANBA (O00462)	Concordant	HF (OR)	1.02 (1.01; 1.03)	1.8×10^{-3}	0.803	20	IVW	Interval

Table S16: Phenome-wide drug target MR scan of proteins with a prioritized CMR association. *(continued)*

Protein (uniprot)	Protein set	Trait	Estimate (95%CI)	P-value	Q p-value	No. variants	MR model	pQTL source
		Non-ischemic CM (OR)	1.04 (0.98; 1.11)	2.3×10^{-1}	0.306	20	IVW	
		DCM (OR)	0.76 (0.72; 0.81)	1.0×10^{-100}	0.014	20	IVW	
		AF (OR)	1.11 (1.05; 1.17)	2.6×10^{-4}	0.078	22	MR Egger	
		CHD (OR)	0.93 (0.91; 0.96)	7.6×10^{-8}	0.004	22	MR Egger	
		cIMT (mm)	0.00 (-0.00; 0.00)	7.7×10^{-2}	0.343	17	IVW	
		Carotid plaque (OR)	1.11 (1.08; 1.14)	2.2×10^{-16}	0.642	18	IVW	
		Any stroke (OR)	1.02 (0.99; 1.06)	1.9×10^{-1}	0.215	22	MR Egger	
		Any ischemic stroke (OR)	1.01 (0.99; 1.03)	2.8×10^{-1}	0.504	20	IVW	
		LDL cholesterol (mmol/l)	0.00 (-0.01; 0.01)	6.7×10^{-1}	0.560	24	MR Egger	
		Apoliprotein B (g/l)	-0.00 (-0.00; 0.00)	3.9×10^{-1}	0.132	23	MR Egger	
		Triglycerides (mmol/l)	-0.03 (-0.04; -0.02)	6.3×10^{-11}	0.185	24	MR Egger	
		Cholesterol (mmol/l)	0.02 (0.01; 0.02)	1.0×10^{-10}	<0.001	19	IVW	
		HDL cholesterol (mmol/l)	0.00 (0.00; 0.01)	6.3×10^{-5}	<0.001	16	IVW	
		Apoliprotein A1 (g/l)	0.00 (0.00; 0.00)	2.9×10^{-3}	<0.001	17	IVW	
		Glucose (mmol/l)	-0.03 (-0.04; -0.03)	1.0×10^{-100}	0.318	20	IVW	
		Glycated haemoglobin (mmol/mol)	-0.05 (-0.13; 0.04)	2.8×10^{-1}	0.035	19	MR Egger	
		T2DM (OR)	0.93 (0.90; 0.97)	1.3×10^{-4}	0.006	21	MR Egger	
		BMI (SD)	-0.02 (-0.02; -0.01)	1.0×10^{-100}	0.017	17	IVW	
		SBP (mmHg)	-0.15 (-0.22; -0.09)	8.8×10^{-6}	0.006	11	IVW	
		DBP (mmHg)	-0.27 (-0.30; -0.24)	1.0×10^{-100}	0.016	17	IVW	
		ECG heart rate (exercise) (BPM)	0.35 (-0.00; 0.70)	5.1×10^{-2}	0.079	25	MR Egger	
		ECG load (exercise) (Watts)	0.50 (0.20; 0.80)	1.2×10^{-3}	0.394	27	MR Egger	
		Asthma (OR)	0.94 (0.92; 0.95)	1.0×10^{-100}	0.249	18	IVW	
		CRP (log(mg/L))	-0.03 (-0.04; -0.02)	1.6×10^{-12}	0.009	17	IVW	
		eGFR (SD of log(eGFR))	0.00 (0.00; 0.01)	3.2×10^{-6}	0.458	20	MR Egger	
		BUN (mg/dl)	-0.01 (-0.01; 0.00)	8.5×10^{-2}	0.207	21	MR Egger	
		CKD (OR)	1.00 (0.94; 1.07)	9.8×10^{-1}	0.136	21	MR Egger	
		Alzheimer's (OR)	1.00 (0.99; 1.00)	5.0×10^{-1}	0.004	21	IVW	
		Parkinson's (OR)	0.92 (0.89; 0.96)	7.4×10^{-5}	0.462	18	IVW	
		Lewy body dementia (OR)	0.99 (0.95; 1.04)	6.5×10^{-1}	0.835	22	IVW	
		Breast cancer (OR)	0.95 (0.91; 1.00)	6.5×10^{-2}	0.040	24	MR Egger	
		Lung cancer (OR)	1.00 (0.96; 1.04)	8.8×10^{-1}	0.779	24	IVW	
		Colon cancer (OR)	1.09 (0.97; 1.23)	1.6×10^{-1}	0.082	23	MR Egger	
		Prostate cancer (OR)	1.03 (1.00; 1.06)	3.3×10^{-2}	0.648	19	IVW	
NCAM2 (O15394)	Concordant	HF (OR)	1.02 (1.00; 1.03)	1.5×10^{-2}	0.010	28	IVW	Interval
		Non-ischemic CM (OR)	0.92 (0.77; 1.10)	3.6×10^{-1}	0.161	27	MR Egger	
		DCM (OR)	0.44 (0.35; 0.56)	3.2×10^{-11}	0.140	29	MR Egger	
		AF (OR)	0.99 (0.98; 1.00)	5.5×10^{-2}	<0.001	26	IVW	
		CHD (OR)	0.95 (0.90; 1.00)	4.5×10^{-2}	0.830	26	MR Egger	
		cIMT (mm)	0.00 (0.00; 0.01)	2.9×10^{-2}	0.582	28	MR Egger	
		Carotid plaque (OR)	1.01 (0.98; 1.04)	4.5×10^{-1}	0.002	26	IVW	
		Any stroke (OR)	0.97 (0.96; 0.99)	3.6×10^{-4}	0.778	28	IVW	
		Any ischemic stroke (OR)	0.98 (0.96; 1.00)	6.5×10^{-2}	0.103	28	IVW	
		LDL cholesterol (mmol/l)	0.01 (-0.00; 0.02)	2.1×10^{-1}	0.044	29	MR Egger	
		Apoliprotein B (g/l)	0.00 (0.00; 0.01)	5.7×10^{-3}	0.017	30	MR Egger	
		Triglycerides (mmol/l)	0.02 (0.00; 0.03)	7.4×10^{-3}	0.272	31	MR Egger	
		Cholesterol (mmol/l)	-0.00 (-0.01; 0.00)	1.7×10^{-1}	0.151	27	IVW	
		HDL cholesterol (mmol/l)	-0.00 (-0.01; 0.00)	8.2×10^{-2}	0.083	29	MR Egger	
		Apoliprotein A1 (g/l)	0.00 (-0.00; 0.01)	3.0×10^{-1}	0.274	29	MR Egger	
		Glucose (mmol/l)	0.03 (0.01; 0.04)	2.6×10^{-4}	0.034	26	MR Egger	
		Glycated haemoglobin (mmol/mol)	0.04 (0.01; 0.06)	1.6×10^{-3}	0.312	31	IVW	
		T2DM (OR)	0.98 (0.97; 0.99)	4.1×10^{-4}	<0.001	26	IVW	
		BMI (SD)	0.00 (-0.00; 0.00)	2.6×10^{-1}	0.008	27	IVW	
		SBP (mmHg)	0.05 (0.00; 0.10)	4.2×10^{-2}	<0.001	27	IVW	
		DBP (mmHg)	0.05 (0.02; 0.07)	3.9×10^{-4}	0.574	27	IVW	
		ECG heart rate (exercise) (BPM)	1.29 (0.88; 1.71)	8.4×10^{-10}	0.015	31	MR Egger	
		ECG load (exercise) (Watts)	0.81 (0.33; 1.28)	8.2×10^{-4}	0.011	28	MR Egger	
		Asthma (OR)	None	None	None	None	None	
		CRP (log(mg/L))	0.00 (-0.00; 0.01)	4.4×10^{-1}	0.029	27	IVW	
		eGFR (SD of log(eGFR))	-0.00 (-0.00; -0.00)	8.5×10^{-7}	0.007	30	IVW	
		BUN (mg/dl)	0.00 (0.00; 0.00)	1.6×10^{-3}	0.005	27	IVW	
		CKD (OR)	1.06 (1.01; 1.11)	2.1×10^{-2}	0.123	29	MR Egger	
		Alzheimer's (OR)	1.01 (1.01; 1.02)	1.3×10^{-15}	0.005	24	IVW	
		Parkinson's (OR)	1.04 (1.00; 1.08)	6.9×10^{-2}	0.043	23	IVW	
		Lewy body dementia (OR)	1.37 (1.29; 1.46)	1.0×10^{-100}	0.014	21	IVW	
		Breast cancer (OR)	0.93 (0.87; 1.00)	5.7×10^{-2}	0.417	25	MR Egger	

Table S16: Phenome-wide drug target MR scan of proteins with a prioritized CMR association. *(continued)*

Protein (uniprot)	Protein set	Trait	Estimate (95%CI)	P-value	Q p-value	No. variants	MR model	pQTL source
		Lung cancer (OR)	1.23 (1.04; 1.46)	1.4×10^{-2}	0.588	26	MR Egger	
		Colon cancer (OR)	1.10 (1.04; 1.15)	2.5×10^{-4}	0.857	27	IVW	
		Prostate cancer (OR)	0.96 (0.89; 1.04)	3.1×10^{-1}	0.896	28	MR Egger	
NET1 (O95631)	Concordant	HF (OR)	1.02 (1.00; 1.04)	2.5×10^{-2}	0.244	27	IVW	Interval
		Non-ischemic CM (OR)	0.93 (0.83; 1.04)	1.8×10^{-1}	0.024	29	MR Egger	
		DCM (OR)	1.00 (0.88; 1.14)	9.9×10^{-1}	0.524	32	MR Egger	
		AF (OR)	0.99 (0.97; 1.00)	9.4×10^{-2}	0.011	26	IVW	
		CHD (OR)	1.01 (0.98; 1.04)	6.0×10^{-1}	0.274	30	MR Egger	
		cIMT (mm)	0.00 (-0.00; 0.00)	9.3×10^{-1}	0.161	28	IVW	
		Carotid plaque (OR)	1.00 (0.94; 1.06)	9.1×10^{-1}	0.809	30	MR Egger	
		Any stroke (OR)	0.97 (0.95; 1.00)	2.8×10^{-2}	0.003	22	IVW	
		Any ischemic stroke (OR)	0.98 (0.96; 1.01)	1.8×10^{-1}	<0.001	23	IVW	
		LDL cholesterol (mmol/l)	-0.01 (-0.01; -0.00)	2.8×10^{-5}	0.063	31	IVW	
		Apoliprotein B (g/l)	-0.00 (-0.00; -0.00)	1.3×10^{-2}	0.441	32	MR Egger	
		Triglycerides (mmol/l)	-0.00 (-0.00; 0.00)	9.8×10^{-1}	<0.001	26	IVW	
		Cholesterol (mmol/l)	-0.01 (-0.02; 0.00)	1.3×10^{-1}	0.559	33	MR Egger	
		HDL cholesterol (mmol/l)	-0.00 (-0.00; 0.00)	5.1×10^{-1}	<0.001	27	IVW	
		Apoliprotein A1 (g/l)	-0.00 (-0.00; -0.00)	1.7×10^{-2}	0.002	28	IVW	
		Glucose (mmol/l)	0.00 (-0.01; 0.01)	6.3×10^{-1}	0.002	30	MR Egger	
		Glycated haemoglobin (mmol/mol)	-0.02 (-0.09; 0.04)	4.3×10^{-1}	0.226	31	MR Egger	
		T2DM (OR)	1.02 (1.00; 1.03)	1.2×10^{-2}	0.011	28	IVW	
		BMI (SD)	0.00 (-0.00; 0.00)	8.6×10^{-1}	<0.001	29	IVW	
		SBP (mmHg)	0.17 (0.07; 0.27)	5.4×10^{-4}	<0.001	31	MR Egger	
		DBP (mmHg)	0.06 (0.02; 0.11)	9.3×10^{-3}	0.074	24	IVW	
		ECG heart rate (exercise) (BPM)	-0.13 (-0.27; 0.01)	6.1×10^{-2}	0.318	30	IVW	
		ECG load (exercise) (Watts)	-0.25 (-0.62; 0.13)	2.0×10^{-1}	0.267	29	MR Egger	
		Asthma (OR)	None	None	None	None	None	
		CRP (log(mg/L))	0.02 (0.01; 0.03)	1.6×10^{-6}	0.003	27	IVW	
		eGFR (SD of log(eGFR))	-0.00 (-0.00; -0.00)	6.2×10^{-7}	0.289	29	MR Egger	
		BUN (mg/dl)	0.00 (-0.00; 0.00)	1.3×10^{-1}	0.006	27	IVW	
		CKD (OR)	1.03 (0.99; 1.06)	1.1×10^{-1}	0.499	30	MR Egger	
		Alzheimer's (OR)	1.00 (0.99; 1.01)	6.6×10^{-1}	0.002	30	MR Egger	
		Parkinson's (OR)	0.99 (0.92; 1.06)	6.8×10^{-1}	0.003	32	MR Egger	
		Lewy body dementia (OR)	0.96 (0.90; 1.03)	2.4×10^{-1}	0.341	30	IVW	
		Breast cancer (OR)	0.97 (0.95; 0.99)	3.2×10^{-3}	<0.001	28	IVW	
		Lung cancer (OR)	1.04 (0.98; 1.11)	2.3×10^{-1}	0.086	28	IVW	
		Colon cancer (OR)	0.95 (0.91; 1.00)	3.8×10^{-2}	<0.001	26	IVW	
		Prostate cancer (OR)	1.13 (1.07; 1.19)	6.1×10^{-6}	0.323	31	MR Egger	
OSMR (Q99650)	Concordant	HF (OR)	1.02 (0.98; 1.06)	2.5×10^{-1}	0.804	8	IVW	Interval
		Non-ischemic CM (OR)	0.83 (0.67; 1.02)	8.2×10^{-2}	0.253	7	IVW	
		DCM (OR)	1.44 (0.83; 2.49)	1.9×10^{-1}	0.445	9	MR Egger	
		AF (OR)	1.01 (0.97; 1.04)	6.8×10^{-1}	0.923	8	IVW	
		CHD (OR)	1.12 (1.00; 1.25)	4.8×10^{-2}	0.757	9	MR Egger	
		cIMT (mm)	0.00 (-0.00; 0.01)	7.3×10^{-2}	0.670	8	IVW	
		Carotid plaque (OR)	1.14 (1.05; 1.23)	1.8×10^{-3}	0.465	8	IVW	
		Any stroke (OR)	1.01 (0.96; 1.06)	7.7×10^{-1}	0.996	8	IVW	
		Any ischemic stroke (OR)	0.98 (0.93; 1.03)	3.3×10^{-1}	0.981	8	IVW	
		LDL cholesterol (mmol/l)	-0.03 (-0.07; 0.00)	9.1×10^{-2}	0.102	9	MR Egger	
		Apoliprotein B (g/l)	-0.00 (-0.00; 0.00)	1.3×10^{-1}	<0.001	6	IVW	
		Triglycerides (mmol/l)	-0.01 (-0.02; 0.01)	4.2×10^{-1}	0.055	8	IVW	
		Cholesterol (mmol/l)	0.00 (-0.01; 0.02)	4.6×10^{-1}	0.299	8	IVW	
		HDL cholesterol (mmol/l)	-0.02 (-0.03; 0.00)	6.1×10^{-2}	0.329	8	MR Egger	
		Apoliprotein A1 (g/l)	-0.01 (-0.02; 0.00)	1.3×10^{-1}	0.129	8	MR Egger	
		Glucose (mmol/l)	-0.02 (-0.03; -0.00)	2.8×10^{-2}	0.387	8	IVW	
		Glycated haemoglobin (mmol/mol)	0.12 (-0.10; 0.35)	2.8×10^{-1}	0.271	9	MR Egger	
		T2DM (OR)	1.07 (1.03; 1.11)	5.0×10^{-4}	0.297	7	IVW	
		BMI (SD)	0.01 (-0.01; 0.02)	2.5×10^{-1}	0.124	6	IVW	
		SBP (mmHg)	-0.13 (-0.36; 0.11)	2.9×10^{-1}	0.062	7	IVW	
		DBP (mmHg)	-0.08 (-0.18; 0.01)	9.1×10^{-2}	<0.001	7	IVW	
		ECG heart rate (exercise) (BPM)	-0.32 (-0.79; 0.15)	1.8×10^{-1}	0.160	8	IVW	
		ECG load (exercise) (Watts)	-0.56 (-1.08; -0.05)	3.2×10^{-2}	0.181	8	IVW	
		Asthma (OR)	1.02 (0.99; 1.04)	2.1×10^{-1}	0.904	8	IVW	
		CRP (log(mg/L))	-0.01 (-0.04; 0.02)	5.5×10^{-1}	0.061	8	IVW	
		eGFR (SD of log(eGFR))	0.00 (-0.00; 0.00)	1.5×10^{-1}	0.014	7	IVW	
		BUN (mg/dl)	-0.01 (-0.03; 0.01)	3.6×10^{-1}	0.007	6	MR Egger	
		CKD (OR)	0.96 (0.91; 1.01)	9.0×10^{-2}	0.457	7	IVW	

Table S16: Phenome-wide drug target MR scan of proteins with a prioritized CMR association. *(continued)*

Protein (uniprot)	Protein set	Trait	Estimate (95%CI)	P-value	Q p-value	No. variants	MR model	pQTL source
		Alzheimer's (OR)	0.99 (0.98; 1.00)	5.3×10^{-2}	0.699	9	IVW	
		Parkinson's (OR)	0.99 (0.89; 1.10)	7.8×10^{-1}	0.384	7	IVW	
		Lewy body dementia (OR)	1.10 (0.92; 1.32)	2.9×10^{-1}	0.880	9	IVW	
		Breast cancer (OR)	1.09 (1.04; 1.14)	6.8×10^{-4}	0.566	8	IVW	
		Lung cancer (OR)	1.00 (0.87; 1.14)	9.8×10^{-1}	0.335	8	IVW	
		Colon cancer (OR)	0.94 (0.83; 1.07)	3.6×10^{-1}	0.143	8	IVW	
		Prostate cancer (OR)	1.11 (1.05; 1.19)	8.6×10^{-4}	0.003	8	IVW	
PATE4 (P0C8F1)	Concordant	HF (OR)	1.00 (0.98; 1.01)	6.5×10^{-1}	0.439	21	IVW	Interval
		Non-ischemic CM (OR)	1.13 (1.07; 1.19)	4.8×10^{-6}	0.200	21	IVW	
		DCM (OR)	1.05 (0.99; 1.11)	9.6×10^{-2}	0.043	23	IVW	
		AF (OR)	0.95 (0.92; 0.99)	4.1×10^{-3}	0.080	24	MR Egger	
		CHD (OR)	0.93 (0.87; 1.00)	4.0×10^{-2}	0.069	20	MR Egger	
		cIMT (mm)	-0.00 (-0.00; -0.00)	3.0×10^{-4}	0.397	21	IVW	
		Carotid plaque (OR)	1.11 (1.07; 1.15)	3.1×10^{-9}	0.399	18	IVW	
		Any stroke (OR)	1.01 (0.99; 1.02)	4.0×10^{-1}	0.290	22	IVW	
		Any ischemic stroke (OR)	0.99 (0.97; 1.01)	5.4×10^{-1}	0.002	19	IVW	
		LDL cholesterol (mmol/l)	-0.01 (-0.01; -0.01)	1.0×10^{-100}	<0.001	27	IVW	
		Apoliprotein B (g/l)	-0.00 (-0.00; -0.00)	1.2×10^{-13}	<0.001	29	IVW	
		Triglycerides (mmol/l)	0.01 (0.01; 0.02)	1.0×10^{-10}	0.158	25	IVW	
		Cholesterol (mmol/l)	-0.01 (-0.03; -0.00)	1.0×10^{-2}	<0.001	28	MR Egger	
		HDL cholesterol (mmol/l)	-0.01 (-0.02; -0.01)	1.0×10^{-8}	<0.001	19	MR Egger	
		Apoliprotein A1 (g/l)	-0.00 (-0.00; -0.00)	2.8×10^{-2}	<0.001	18	IVW	
		Glucose (mmol/l)	0.01 (-0.01; 0.02)	3.6×10^{-1}	<0.001	21	MR Egger	
		Glycated haemoglobin (mmol/mol)	0.03 (-0.06; 0.12)	5.0×10^{-1}	0.059	26	MR Egger	
		T2DM (OR)	1.01 (1.00; 1.02)	9.0×10^{-2}	0.003	17	IVW	
		BMI (SD)	0.01 (0.01; 0.02)	7.8×10^{-11}	0.352	16	IVW	
		SBP (mmHg)	-0.27 (-0.34; -0.21)	1.0×10^{-100}	<0.001	19	IVW	
		DBP (mmHg)	-0.18 (-0.24; -0.13)	1.1×10^{-11}	0.028	24	MR Egger	
		ECG heart rate (exercise) (BPM)	-0.42 (-0.82; -0.03)	3.6×10^{-2}	0.211	29	MR Egger	
		ECG load (exercise) (Watts)	-0.61 (-0.72; -0.50)	1.0×10^{-100}	<0.001	28	IVW	
		Asthma (OR)	1.01 (1.01; 1.02)	1.3×10^{-4}	0.005	33	IVW	
		CRP (log(mg/L))	-0.02 (-0.02; -0.01)	1.7×10^{-9}	0.818	24	IVW	
		eGFR (SD of log(eGFR))	0.00 (0.00; 0.00)	1.4×10^{-3}	0.002	20	IVW	
		BUN (mg/dl)	-0.01 (-0.01; -0.00)	4.9×10^{-3}	0.555	18	MR Egger	
		CKD (OR)	0.99 (0.97; 1.00)	6.1×10^{-2}	0.032	20	IVW	
		Alzheimer's (OR)	1.00 (0.99; 1.01)	5.5×10^{-1}	0.077	24	MR Egger	
		Parkinson's (OR)	1.05 (1.00; 1.11)	7.4×10^{-2}	0.027	25	MR Egger	
		Lewy body dementia (OR)	0.82 (0.70; 0.97)	1.9×10^{-2}	0.046	22	MR Egger	
		Breast cancer (OR)	0.97 (0.93; 1.02)	2.7×10^{-1}	0.255	22	MR Egger	
		Lung cancer (OR)	1.56 (1.31; 1.86)	4.3×10^{-7}	0.021	19	MR Egger	
		Colon cancer (OR)	1.17 (1.05; 1.29)	3.6×10^{-3}	0.228	22	MR Egger	
		Prostate cancer (OR)	0.86 (0.81; 0.92)	9.6×10^{-6}	0.169	21	MR Egger	
PGLT1 (Q8NBL1)	Concordant	HF (OR)	1.10 (1.06; 1.13)	2.3×10^{-8}	0.387	9	IVW	Interval
		Non-ischemic CM (OR)	0.62 (0.45; 0.85)	3.0×10^{-3}	0.522	10	MR Egger	
		DCM (OR)	0.72 (0.61; 0.85)	8.4×10^{-5}	0.967	10	IVW	
		AF (OR)	1.01 (0.95; 1.08)	6.7×10^{-1}	0.037	10	MR Egger	
		CHD (OR)	1.04 (0.96; 1.12)	3.4×10^{-1}	0.870	10	MR Egger	
		cIMT (mm)	-0.00 (-0.00; 0.00)	2.3×10^{-1}	0.623	9	IVW	
		Carotid plaque (OR)	1.02 (0.95; 1.10)	5.8×10^{-1}	0.041	8	IVW	
		Any stroke (OR)	1.01 (0.96; 1.07)	5.8×10^{-1}	0.054	9	IVW	
		Any ischemic stroke (OR)	1.05 (1.00; 1.11)	3.7×10^{-2}	0.130	9	IVW	
		LDL cholesterol (mmol/l)	-0.01 (-0.02; -0.00)	4.5×10^{-3}	0.021	10	IVW	
		Apoliprotein B (g/l)	-0.00 (-0.00; 0.00)	5.1×10^{-1}	0.297	10	IVW	
		Triglycerides (mmol/l)	0.02 (-0.00; 0.05)	5.6×10^{-2}	0.274	11	MR Egger	
		Cholesterol (mmol/l)	-0.01 (-0.02; 0.00)	1.2×10^{-1}	0.111	11	IVW	
		HDL cholesterol (mmol/l)	-0.01 (-0.02; 0.00)	7.8×10^{-2}	0.078	11	MR Egger	
		Apoliprotein A1 (g/l)	0.01 (0.00; 0.01)	1.1×10^{-5}	0.024	9	IVW	
		Glucose (mmol/l)	0.01 (-0.01; 0.02)	2.9×10^{-1}	0.498	9	IVW	
		Glycated haemoglobin (mmol/mol)	-0.23 (-0.39; -0.08)	3.0×10^{-3}	0.272	11	MR Egger	
		T2DM (OR)	1.04 (1.01; 1.08)	7.5×10^{-3}	0.029	8	IVW	
		BMI (SD)	-0.06 (-0.09; -0.02)	2.2×10^{-3}	0.337	7	MR Egger	
		SBP (mmHg)	-0.11 (-0.23; 0.02)	9.7×10^{-2}	0.444	10	IVW	
		DBP (mmHg)	-0.34 (-0.50; -0.19)	1.7×10^{-5}	0.894	10	MR Egger	
		ECG heart rate (exercise) (BPM)	1.07 (0.28; 1.87)	7.7×10^{-3}	0.994	11	MR Egger	
		ECG load (exercise) (Watts)	1.63 (0.67; 2.59)	8.5×10^{-4}	0.311	11	MR Egger	
		Asthma (OR)	0.96 (0.93; 0.99)	3.1×10^{-3}	0.353	9	IVW	

Table S16: Phenome-wide drug target MR scan of proteins with a prioritized CMR association. *(continued)*

Protein (uniprot)	Protein set	Trait	Estimate (95%CI)	P-value	Q p-value	No. variants	MR model	pQTL source
		CRP (log(mg/L))	0.01 (-0.01; 0.02)	4.6×10^{-1}	0.666	9	IVW	
		eGFR (SD of log(eGFR))	0.00 (0.00; 0.01)	3.1×10^{-7}	0.145	9	IVW	
		BUN (mg/dl)	-0.01 (-0.01; -0.00)	2.8×10^{-3}	0.221	10	IVW	
		CKD (OR)	1.05 (1.02; 1.10)	5.5×10^{-3}	0.604	9	IVW	
		Alzheimer’s (OR)	0.99 (0.97; 1.01)	4.6×10^{-1}	0.314	9	MR Egger	
		Parkinson’s (OR)	1.15 (1.05; 1.25)	1.7×10^{-3}	0.441	9	IVW	
		Lewy body dementia (OR)	0.77 (0.66; 0.89)	6.0×10^{-4}	0.970	10	IVW	
		Breast cancer (OR)	1.03 (0.98; 1.08)	2.7×10^{-1}	0.048	8	IVW	
		Lung cancer (OR)	1.16 (1.03; 1.30)	1.4×10^{-2}	0.564	10	IVW	
		Colon cancer (OR)	1.12 (1.02; 1.23)	2.1×10^{-2}	0.855	10	IVW	
		Prostate cancer (OR)	0.93 (0.87; 0.99)	2.7×10^{-2}	0.281	10	IVW	
PPAC (P24666)	Concordant	HF (OR)	1.00 (1.00; 1.01)	1.9×10^{-1}	0.076	38	IVW	Interval
		Non-ischemic CM (OR)	0.94 (0.89; 0.98)	7.0×10^{-3}	0.603	43	MR Egger	
		DCM (OR)	0.97 (0.95; 1.00)	2.8×10^{-2}	<0.001	29	IVW	
		AF (OR)	1.02 (1.01; 1.02)	1.9×10^{-13}	0.075	45	IVW	
		CHD (OR)	1.00 (1.00; 1.01)	6.1×10^{-1}	0.146	36	IVW	
		cIMT (mm)	0.00 (0.00; 0.00)	6.3×10^{-3}	0.037	41	MR Egger	
		Carotid plaque (OR)	1.04 (1.03; 1.05)	9.8×10^{-11}	0.027	42	IVW	
		Any stroke (OR)	1.01 (1.00; 1.02)	2.9×10^{-2}	0.078	38	IVW	
		Any ischemic stroke (OR)	1.03 (1.00; 1.05)	1.5×10^{-2}	0.071	42	MR Egger	
		LDL cholesterol (mmol/l)	0.00 (0.00; 0.01)	8.6×10^{-3}	0.479	42	MR Egger	
		Apoliprotein B (g/l)	0.00 (0.00; 0.00)	2.5×10^{-2}	0.365	42	MR Egger	
		Triglycerides (mmol/l)	-0.01 (-0.01; -0.01)	1.0×10^{-100}	0.019	37	IVW	
		Cholesterol (mmol/l)	0.01 (0.00; 0.01)	1.8×10^{-3}	0.195	43	MR Egger	
		HDL cholesterol (mmol/l)	0.00 (-0.00; 0.00)	1.1×10^{-1}	0.191	44	MR Egger	
		Apoliprotein A1 (g/l)	0.00 (-0.00; 0.00)	5.9×10^{-2}	0.411	44	MR Egger	
		Glucose (mmol/l)	0.01 (0.00; 0.01)	5.3×10^{-4}	0.030	43	MR Egger	
		Glycated haemoglobin (mmol/mol)	0.05 (0.02; 0.08)	3.4×10^{-4}	0.092	41	MR Egger	
		T2DM (OR)	1.00 (0.99; 1.01)	8.5×10^{-1}	0.095	40	MR Egger	
		BMI (SD)	-0.01 (-0.01; 0.00)	8.3×10^{-2}	<0.001	27	MR Egger	
		SBP (mmHg)	0.10 (0.06; 0.15)	1.0×10^{-5}	0.159	17	IVW	
		DBP (mmHg)	0.02 (-0.01; 0.05)	1.4×10^{-1}	0.285	26	MR Egger	
		ECG heart rate (exercise) (BPM)	0.16 (0.04; 0.29)	9.0×10^{-3}	<0.001	41	MR Egger	
		ECG load (exercise) (Watts)	-0.14 (-0.27; 0.00)	5.0×10^{-2}	<0.001	43	MR Egger	
		Asthma (OR)	None	None	None	None	None	
		CRP (log(mg/L))	0.00 (-0.00; 0.01)	4.3×10^{-1}	0.930	26	MR Egger	
		eGFR (SD of log(eGFR))	-0.00 (-0.00; -0.00)	1.0×10^{-100}	0.172	41	IVW	
		BUN (mg/dl)	0.00 (0.00; 0.00)	6.9×10^{-4}	0.221	39	MR Egger	
		CKD (OR)	1.02 (1.02; 1.03)	1.9×10^{-12}	0.008	39	IVW	
		Alzheimer’s (OR)	0.99 (0.99; 1.00)	1.0×10^{-100}	<0.001	42	IVW	
		Parkinson’s (OR)	1.05 (1.02; 1.09)	4.9×10^{-4}	0.714	43	MR Egger	
		Lewy body dementia (OR)	1.09 (1.03; 1.16)	3.0×10^{-3}	0.005	42	MR Egger	
		Breast cancer (OR)	1.01 (0.99; 1.03)	2.1×10^{-1}	0.006	43	MR Egger	
		Lung cancer (OR)	0.97 (0.95; 0.99)	6.0×10^{-3}	0.454	44	IVW	
		Colon cancer (OR)	0.97 (0.96; 0.99)	3.8×10^{-4}	<0.001	42	IVW	
		Prostate cancer (OR)	0.97 (0.95; 0.99)	7.1×10^{-3}	0.044	45	MR Egger	
PRDX1 (Q06830)	Concordant	HF (OR)	1.07 (0.97; 1.16)	1.6×10^{-1}	0.909	2	IVW	Interval
		Non-ischemic CM (OR)	1.14 (0.71; 1.83)	5.9×10^{-1}	0.196	2	IVW	
		DCM (OR)	1.17 (0.60; 2.31)	6.5×10^{-1}	0.148	2	IVW	
		AF (OR)	0.97 (0.90; 1.05)	4.2×10^{-1}	0.436	2	IVW	
		CHD (OR)	0.98 (0.89; 1.08)	6.8×10^{-1}	0.851	2	IVW	
		cIMT (mm)	0.01 (-0.00; 0.02)	7.6×10^{-2}	0.910	2	IVW	
		Carotid plaque (OR)	0.94 (0.77; 1.16)	5.8×10^{-1}	0.630	2	IVW	
		Any stroke (OR)	1.14 (1.02; 1.27)	2.4×10^{-2}	0.462	2	IVW	
		Any ischemic stroke (OR)	1.06 (0.94; 1.19)	3.5×10^{-1}	0.604	2	IVW	
		LDL cholesterol (mmol/l)	-0.02 (-0.03; 0.00)	8.3×10^{-2}	0.528	3	IVW	
		Apoliprotein B (g/l)	0.00 (-0.01; 0.01)	9.0×10^{-1}	0.254	3	IVW	
		Triglycerides (mmol/l)	0.00 (-0.02; 0.03)	7.4×10^{-1}	0.146	3	IVW	
		Cholesterol (mmol/l)	-0.01 (-0.03; 0.01)	2.9×10^{-1}	0.490	3	IVW	
		HDL cholesterol (mmol/l)	0.01 (0.00; 0.02)	1.9×10^{-2}	0.860	3	IVW	
		Apoliprotein A1 (g/l)	0.01 (0.00; 0.02)	1.5×10^{-4}	0.655	3	IVW	
		Glucose (mmol/l)	-0.02 (-0.04; 0.01)	2.2×10^{-1}	0.387	3	IVW	
		Glycated haemoglobin (mmol/mol)	0.89 (0.38; 1.40)	6.7×10^{-4}	0.007	3	MR Egger	
		T2DM (OR)	1.09 (0.98; 1.21)	1.0×10^{-1}	0.137	2	IVW	
		BMI (SD)	0.02 (-0.00; 0.04)	6.1×10^{-2}	0.912	2	IVW	
		SBP (mmHg)	0.65 (0.31; 1.00)	1.7×10^{-4}	0.449	2	IVW	

Table S16: Phenome-wide drug target MR scan of proteins with a prioritized CMR association. *(continued)*

Protein (uniprot)	Protein set	Trait	Estimate (95%CI)	P-value	Q p-value	No. variants	MR model	pQTL source
		DBP (mmHg)	0.16 (-0.11; 0.42)	2.4×10^{-1}	0.172	2	IVW	
		ECG heart rate (exercise) (BPM)	0.93 (0.20; 1.66)	1.2×10^{-2}	0.889	3	IVW	
		ECG load (exercise) (Watts)	0.43 (-0.68; 1.54)	4.5×10^{-1}	0.156	3	IVW	
		Asthma (OR)	0.97 (0.91; 1.04)	4.5×10^{-1}	0.151	3	IVW	
		CRP (log(mg/L))	0.11 (0.06; 0.16)	4.8×10^{-5}	0.919	2	IVW	
		eGFR (SD of log(eGFR))	0.02 (0.01; 0.03)	5.2×10^{-10}	0.106	2	IVW	
		BUN (mg/dl)	-0.00 (-0.01; 0.01)	6.7×10^{-1}	0.674	2	IVW	
		CKD (OR)	0.83 (0.75; 0.93)	1.1×10^{-3}	0.529	2	IVW	
		Alzheimer’s (OR)	1.02 (1.00; 1.05)	6.2×10^{-2}	0.429	2	IVW	
		Parkinson’s (OR)	0.92 (0.74; 1.14)	4.5×10^{-1}	0.616	2	IVW	
		Lewy body dementia (OR)	0.47 (0.32; 0.71)	3.1×10^{-4}	0.441	2	IVW	
		Breast cancer (OR)	1.39 (1.21; 1.59)	2.2×10^{-6}	0.275	2	IVW	
		Lung cancer (OR)	1.25 (0.90; 1.73)	1.8×10^{-1}	0.750	2	IVW	
		Colon cancer (OR)	1.01 (0.63; 1.62)	9.6×10^{-1}	0.072	2	IVW	
		Prostate cancer (OR)	1.06 (0.90; 1.24)	5.1×10^{-1}	0.413	2	IVW	
RMD1 (Q96DB5)	Concordant	HF (OR)	1.00 (0.99; 1.02)	6.5×10^{-1}	0.799	4	IVW	Interval
		Non-ischemic CM (OR)	1.08 (0.99; 1.17)	7.9×10^{-2}	0.487	4	IVW	
		DCM (OR)	1.01 (0.73; 1.38)	9.7×10^{-1}	0.946	4	IVW	
		AF (OR)	1.01 (0.96; 1.05)	7.9×10^{-1}	0.961	6	IVW	
		CHD (OR)	1.00 (0.98; 1.01)	4.9×10^{-1}	0.525	5	IVW	
		cIMT (mm)	-0.00 (-0.00; -0.00)	9.2×10^{-3}	0.303	4	IVW	
		Carotid plaque (OR)	0.98 (0.94; 1.02)	2.9×10^{-1}	0.947	4	IVW	
		Any stroke (OR)	0.96 (0.94; 0.98)	2.4×10^{-4}	0.773	4	IVW	
		Any ischemic stroke (OR)	0.96 (0.94; 0.99)	3.7×10^{-3}	0.930	4	IVW	
		LDL cholesterol (mmol/l)	0.03 (0.02; 0.03)	6.2×10^{-14}	0.522	6	IVW	
		Apoliprotein B (g/l)	0.01 (0.00; 0.02)	1.6×10^{-2}	0.783	6	MR Egger	
		Triglycerides (mmol/l)	0.11 (0.05; 0.16)	3.9×10^{-4}	0.180	6	MR Egger	
		Cholesterol (mmol/l)	0.03 (0.02; 0.04)	2.3×10^{-11}	0.730	6	IVW	
		HDL cholesterol (mmol/l)	0.00 (-0.00; 0.01)	9.9×10^{-2}	0.397	6	IVW	
		Apoliprotein A1 (g/l)	0.00 (0.00; 0.01)	1.1×10^{-3}	0.741	6	IVW	
		Glucose (mmol/l)	0.15 (0.09; 0.21)	1.0×10^{-6}	0.697	6	MR Egger	
		Glycated haemoglobin (mmol/mol)	0.51 (0.21; 0.81)	8.3×10^{-4}	0.497	6	MR Egger	
		T2DM (OR)	1.19 (0.95; 1.49)	1.3×10^{-1}	0.578	4	MR Egger	
		BMI (SD)	0.01 (-0.08; 0.09)	8.8×10^{-1}	0.181	4	MR Egger	
		SBP (mmHg)	-0.13 (-0.24; -0.02)	1.6×10^{-2}	0.107	4	IVW	
		DBP (mmHg)	-0.02 (-0.06; 0.03)	4.6×10^{-1}	0.351	4	IVW	
		ECG heart rate (exercise) (BPM)	-0.46 (-0.75; -0.17)	2.0×10^{-3}	0.953	6	IVW	
		ECG load (exercise) (Watts)	0.72 (0.40; 1.05)	1.4×10^{-5}	0.773	6	IVW	
		Asthma (OR)	0.97 (0.96; 0.99)	1.6×10^{-3}	0.049	8	IVW	
		CRP (log(mg/L))	-0.00 (-0.01; 0.01)	8.9×10^{-1}	0.899	4	IVW	
		eGFR (SD of log(eGFR))	-0.01 (-0.01; -0.00)	1.0×10^{-100}	0.072	4	IVW	
		BUN (mg/dl)	-0.00 (-0.00; -0.00)	4.9×10^{-2}	0.860	4	IVW	
		CKD (OR)	1.06 (1.02; 1.09)	1.3×10^{-3}	0.095	4	IVW	
		Alzheimer’s (OR)	0.99 (0.95; 1.03)	5.4×10^{-1}	0.273	3	IVW	
		Parkinson’s (OR)	0.94 (0.89; 0.99)	3.1×10^{-2}	0.774	4	IVW	
		Lewy body dementia (OR)	1.08 (1.00; 1.17)	6.0×10^{-2}	0.102	5	IVW	
		Breast cancer (OR)	1.08 (1.04; 1.11)	7.1×10^{-7}	0.870	4	IVW	
		Lung cancer (OR)	1.13 (1.08; 1.18)	4.1×10^{-7}	0.790	5	IVW	
		Colon cancer (OR)	1.21 (1.14; 1.29)	3.5×10^{-10}	0.521	4	IVW	
		Prostate cancer (OR)	1.03 (1.01; 1.06)	1.8×10^{-2}	0.361	5	IVW	
SPA12 (Q8IW75)	Concordant	HF (OR)	1.00 (0.98; 1.03)	7.1×10^{-1}	0.015	16	IVW	Interval
		Non-ischemic CM (OR)	1.32 (1.06; 1.65)	1.5×10^{-2}	0.002	18	MR Egger	
		DCM (OR)	1.02 (0.92; 1.13)	7.3×10^{-1}	0.523	17	IVW	
		AF (OR)	1.03 (0.98; 1.08)	2.1×10^{-1}	0.452	18	MR Egger	
		CHD (OR)	1.05 (1.03; 1.06)	4.0×10^{-9}	0.014	17	IVW	
		cIMT (mm)	0.01 (0.00; 0.01)	9.0×10^{-6}	0.231	17	IVW	
		Carotid plaque (OR)	0.90 (0.81; 1.01)	6.7×10^{-2}	0.713	19	MR Egger	
		Any stroke (OR)	1.03 (1.00; 1.06)	2.7×10^{-2}	0.639	16	IVW	
		Any ischemic stroke (OR)	1.03 (0.99; 1.06)	1.4×10^{-1}	0.142	15	IVW	
		LDL cholesterol (mmol/l)	0.00 (-0.01; 0.02)	6.9×10^{-1}	0.145	20	MR Egger	
		Apoliprotein B (g/l)	0.00 (-0.00; 0.01)	7.0×10^{-1}	0.140	20	MR Egger	
		Triglycerides (mmol/l)	0.01 (-0.01; 0.03)	3.6×10^{-1}	0.290	20	MR Egger	
		Cholesterol (mmol/l)	-0.00 (-0.01; 0.00)	7.1×10^{-1}	0.092	19	IVW	
		HDL cholesterol (mmol/l)	-0.01 (-0.02; -0.00)	1.8×10^{-2}	0.738	20	MR Egger	
		Apoliprotein A1 (g/l)	-0.00 (-0.01; 0.00)	9.0×10^{-2}	0.492	20	MR Egger	
		Glucose (mmol/l)	-0.01 (-0.04; 0.01)	3.5×10^{-1}	0.434	19	MR Egger	

Table S16: Phenome-wide drug target MR scan of proteins with a prioritized CMR association. *(continued)*

Protein (uniprot)	Protein set	Trait	Estimate (95%CI)	P-value	Q p-value	No. variants	MR model	pQTL source
		Glycated haemoglobin (mmol/mol)	-0.03 (-0.15; 0.09)	6.5×10^{-1}	0.625	19	MR Egger	
		T2DM (OR)	1.00 (0.98; 1.03)	6.4×10^{-1}	0.217	16	IVW	
		BMI (SD)	0.01 (0.00; 0.01)	5.9×10^{-6}	0.261	18	IVW	
		SBP (mmHg)	-0.28 (-0.53; -0.04)	2.5×10^{-2}	0.099	19	MR Egger	
		DBP (mmHg)	-0.11 (-0.22; 0.01)	7.7×10^{-2}	0.698	19	MR Egger	
		ECG heart rate (exercise) (BPM)	0.01 (-0.22; 0.24)	9.3×10^{-1}	0.099	18	IVW	
		ECG load (exercise) (Watts)	0.44 (0.23; 0.65)	4.9×10^{-5}	0.033	18	IVW	
		Asthma (OR)	1.00 (0.99; 1.01)	4.6×10^{-1}	0.465	20	IVW	
		CRP (log(mg/L))	0.02 (0.01; 0.03)	2.1×10^{-5}	0.674	17	IVW	
		eGFR (SD of log(eGFR))	0.00 (0.00; 0.01)	5.2×10^{-3}	0.824	18	MR Egger	
		BUN (mg/dl)	0.00 (-0.00; 0.00)	9.8×10^{-1}	0.621	17	IVW	
		CKD (OR)	0.98 (0.91; 1.06)	6.6×10^{-1}	0.152	17	MR Egger	
		Alzheimer's (OR)	1.00 (0.99; 1.00)	1.8×10^{-1}	0.437	17	IVW	
		Parkinson's (OR)	0.86 (0.74; 1.00)	4.4×10^{-2}	0.324	18	MR Egger	
		Lewy body dementia (OR)	1.22 (1.13; 1.32)	2.2×10^{-7}	0.296	16	IVW	
		Breast cancer (OR)	0.96 (0.93; 0.99)	5.2×10^{-3}	0.018	15	IVW	
		Lung cancer (OR)	1.00 (0.93; 1.09)	9.1×10^{-1}	0.034	15	IVW	
		Colon cancer (OR)	0.97 (0.91; 1.04)	3.6×10^{-1}	0.088	18	IVW	
		Prostate cancer (OR)	1.12 (1.08; 1.15)	7.8×10^{-10}	0.347	18	IVW	
TPSNR (Q9BX59)	Concordant	HF (OR)	0.99 (0.98; 1.00)	1.8×10^{-2}	0.002	47	IVW	Interval
		Non-ischemic CM (OR)	0.98 (0.94; 1.01)	1.7×10^{-1}	0.029	44	IVW	
		DCM (OR)	0.96 (0.92; 1.01)	1.5×10^{-1}	0.087	36	IVW	
		AF (OR)	1.00 (0.98; 1.01)	8.3×10^{-1}	0.036	50	MR Egger	
		CHD (OR)	1.00 (0.98; 1.02)	9.5×10^{-1}	0.431	49	MR Egger	
		cIMT (mm)	0.00 (0.00; 0.00)	4.5×10^{-2}	0.002	46	IVW	
		Carotid plaque (OR)	0.98 (0.95; 1.01)	2.3×10^{-1}	0.578	45	MR Egger	
		Any stroke (OR)	1.01 (0.99; 1.02)	5.9×10^{-1}	<0.001	46	MR Egger	
		Any ischemic stroke (OR)	1.02 (1.00; 1.04)	1.3×10^{-1}	<0.001	45	MR Egger	
		LDL cholesterol (mmol/l)	0.00 (0.00; 0.00)	8.7×10^{-4}	0.436	61	IVW	
		Apoliprotein B (g/l)	0.00 (-0.00; 0.00)	8.5×10^{-2}	0.182	61	IVW	
		Triglycerides (mmol/l)	-0.01 (-0.01; -0.01)	1.0×10^{-100}	<0.001	54	IVW	
		Cholesterol (mmol/l)	0.00 (0.00; 0.00)	4.8×10^{-3}	<0.001	59	IVW	
		HDL cholesterol (mmol/l)	0.00 (0.00; 0.00)	1.0×10^{-4}	<0.001	49	MR Egger	
		Apoliprotein A1 (g/l)	0.00 (0.00; 0.00)	3.1×10^{-13}	<0.001	51	MR Egger	
		Glucose (mmol/l)	0.00 (-0.00; 0.01)	4.0×10^{-1}	0.077	53	MR Egger	
		Glycated haemoglobin (mmol/mol)	-0.00 (-0.01; 0.01)	4.8×10^{-1}	<0.001	56	IVW	
		T2DM (OR)	1.02 (1.00; 1.03)	5.4×10^{-2}	<0.001	42	MR Egger	
		BMI (SD)	0.00 (-0.00; 0.00)	3.7×10^{-1}	0.381	42	IVW	
		SBP (mmHg)	-0.00 (-0.03; 0.03)	8.8×10^{-1}	0.004	35	IVW	
		DBP (mmHg)	0.03 (0.01; 0.05)	1.3×10^{-3}	<0.001	34	IVW	
		ECG heart rate (exercise) (BPM)	-0.22 (-0.31; -0.12)	6.0×10^{-6}	0.114	60	MR Egger	
		ECG load (exercise) (Watts)	0.07 (0.01; 0.12)	1.3×10^{-2}	0.438	60	IVW	
		Asthma (OR)	1.01 (1.00; 1.01)	4.0×10^{-4}	0.016	51	IVW	
		CRP (log(mg/L))	0.01 (-0.00; 0.02)	5.7×10^{-2}	0.062	44	MR Egger	
		eGFR (SD of log(eGFR))	-0.00 (-0.00; 0.00)	5.9×10^{-1}	0.210	43	MR Egger	
		BUN (mg/dl)	-0.00 (-0.00; -0.00)	1.9×10^{-4}	0.006	42	IVW	
		CKD (OR)	0.99 (0.97; 1.01)	4.7×10^{-1}	0.035	45	MR Egger	
		Alzheimer's (OR)	1.00 (1.00; 1.01)	7.1×10^{-5}	0.003	47	IVW	
		Parkinson's (OR)	0.96 (0.94; 0.98)	1.1×10^{-3}	0.063	43	IVW	
		Lewy body dementia (OR)	1.00 (0.95; 1.06)	8.7×10^{-1}	0.246	44	MR Egger	
		Breast cancer (OR)	0.97 (0.96; 0.98)	3.0×10^{-6}	0.009	48	IVW	
		Lung cancer (OR)	1.01 (0.98; 1.05)	4.5×10^{-1}	0.017	47	IVW	
		Colon cancer (OR)	1.04 (1.01; 1.07)	1.1×10^{-2}	0.002	48	IVW	
		Prostate cancer (OR)	0.98 (0.96; 1.00)	1.6×10^{-2}	0.371	46	IVW	
TREM1 (Q9NP99)	Concordant	HF (OR)	0.97 (0.93; 1.01)	9.4×10^{-2}	0.099	29	MR Egger	Interval
		Non-ischemic CM (OR)	1.12 (0.98; 1.27)	9.5×10^{-2}	0.050	27	MR Egger	
		DCM (OR)	1.32 (1.11; 1.57)	2.0×10^{-3}	0.002	27	MR Egger	
		AF (OR)	1.03 (1.01; 1.05)	3.6×10^{-3}	0.208	29	MR Egger	
		CHD (OR)	1.01 (1.00; 1.02)	1.7×10^{-1}	0.063	30	IVW	
		cIMT (mm)	-0.00 (-0.00; -0.00)	6.2×10^{-4}	0.234	29	IVW	
		Carotid plaque (OR)	0.97 (0.95; 0.99)	5.9×10^{-4}	0.191	29	IVW	
		Any stroke (OR)	0.96 (0.93; 1.00)	5.7×10^{-2}	0.018	28	MR Egger	
		Any ischemic stroke (OR)	0.99 (0.96; 1.03)	7.6×10^{-1}	<0.001	29	MR Egger	
		LDL cholesterol (mmol/l)	0.01 (0.00; 0.01)	2.3×10^{-4}	0.180	31	MR Egger	
		Apoliprotein B (g/l)	0.00 (0.00; 0.00)	1.2×10^{-2}	0.232	31	MR Egger	
		Triglycerides (mmol/l)	-0.00 (-0.01; -0.00)	4.3×10^{-2}	0.061	27	IVW	

Table S16: Phenome-wide drug target MR scan of proteins with a prioritized CMR association. *(continued)*

Protein (uniprot)	Protein set	Trait	Estimate (95%CI)	P-value	Q p-value	No. variants	MR model	pQTL source
		Cholesterol (mmol/l)	0.01 (0.00; 0.02)	4.9×10^{-4}	0.117	30	MR Egger	
		HDL cholesterol (mmol/l)	-0.00 (-0.01; 0.00)	2.5×10^{-1}	0.549	26	MR Egger	
		Apoliprotein A1 (g/l)	-0.00 (-0.00; 0.00)	5.8×10^{-1}	0.167	27	MR Egger	
		Glucose (mmol/l)	0.00 (-0.00; 0.01)	2.1×10^{-1}	0.007	26	MR Egger	
		Glycated haemoglobin (mmol/mol)	0.05 (0.03; 0.06)	6.4×10^{-7}	0.146	29	IVW	
		T2DM (OR)	1.01 (1.00; 1.02)	8.3×10^{-2}	0.160	28	IVW	
		BMI (SD)	-0.00 (-0.01; 0.00)	2.6×10^{-1}	<0.001	19	IVW	
		SBP (mmHg)	-0.16 (-0.27; -0.06)	1.9×10^{-3}	0.678	28	MR Egger	
		DBP (mmHg)	-0.04 (-0.07; -0.01)	4.5×10^{-3}	<0.001	25	IVW	
		ECG heart rate (exercise) (BPM)	0.34 (0.21; 0.47)	2.0×10^{-7}	0.079	25	IVW	
		ECG load (exercise) (Watts)	0.09 (-0.04; 0.22)	1.9×10^{-1}	0.074	30	IVW	
		Asthma (OR)	None	None	None	None	None	
		CRP (log(mg/L))	-0.01 (-0.03; 0.01)	5.1×10^{-1}	0.102	25	MR Egger	
		eGFR (SD of log(eGFR))	0.00 (-0.00; 0.00)	5.7×10^{-2}	0.032	26	MR Egger	
		BUN (mg/dl)	-0.00 (-0.00; 0.00)	8.8×10^{-1}	0.030	25	IVW	
		CKD (OR)	0.95 (0.91; 0.99)	1.2×10^{-2}	0.248	28	MR Egger	
		Alzheimer's (OR)	1.00 (0.99; 1.01)	4.1×10^{-1}	0.042	28	MR Egger	
		Parkinson's (OR)	1.00 (0.90; 1.10)	9.8×10^{-1}	0.120	21	MR Egger	
		Lewy body dementia (OR)	0.96 (0.92; 1.00)	6.2×10^{-2}	0.040	25	IVW	
		Breast cancer (OR)	1.03 (1.01; 1.05)	5.9×10^{-3}	0.435	30	MR Egger	
		Lung cancer (OR)	1.23 (1.14; 1.33)	2.2×10^{-7}	0.010	29	MR Egger	
		Colon cancer (OR)	1.03 (1.01; 1.06)	1.6×10^{-2}	0.183	30	IVW	
		Prostate cancer (OR)	0.96 (0.95; 0.97)	1.4×10^{-10}	<0.001	32	IVW	
UD16 (P19224)	Concordant	HF (OR)	1.03 (1.01; 1.05)	3.6×10^{-3}	0.047	15	IVW	Interval
		Non-ischemic CM (OR)	1.10 (1.02; 1.18)	8.7×10^{-3}	0.322	17	IVW	
		DCM (OR)	1.62 (1.46; 1.80)	1.0×10^{-100}	0.055	16	IVW	
		AF (OR)	0.95 (0.90; 1.00)	4.8×10^{-2}	0.685	16	MR Egger	
		CHD (OR)	1.06 (1.04; 1.08)	8.5×10^{-8}	0.153	15	IVW	
		cIMT (mm)	-0.00 (-0.00; -0.00)	2.7×10^{-3}	0.464	16	IVW	
		Carotid plaque (OR)	1.01 (0.88; 1.15)	9.3×10^{-1}	0.754	15	MR Egger	
		Any stroke (OR)	1.05 (0.97; 1.13)	2.4×10^{-1}	0.755	15	MR Egger	
		Any ischemic stroke (OR)	1.05 (0.96; 1.14)	2.9×10^{-1}	0.829	15	MR Egger	
		LDL cholesterol (mmol/l)	0.04 (0.03; 0.04)	1.0×10^{-100}	0.013	13	IVW	
		Apoliprotein B (g/l)	0.01 (0.01; 0.01)	1.0×10^{-100}	0.005	11	IVW	
		Triglycerides (mmol/l)	0.03 (0.03; 0.04)	1.0×10^{-100}	0.087	16	IVW	
		Cholesterol (mmol/l)	0.08 (0.07; 0.10)	1.0×10^{-100}	0.006	17	MR Egger	
		HDL cholesterol (mmol/l)	-0.00 (-0.00; 0.00)	3.3×10^{-1}	0.062	15	IVW	
		Apoliprotein A1 (g/l)	0.00 (0.00; 0.01)	4.6×10^{-2}	0.063	18	MR Egger	
		Glucose (mmol/l)	0.01 (-0.00; 0.03)	1.1×10^{-1}	0.625	16	MR Egger	
		Glycated haemoglobin (mmol/mol)	0.07 (-0.04; 0.18)	1.9×10^{-1}	0.161	15	MR Egger	
		T2DM (OR)	1.01 (0.96; 1.06)	7.4×10^{-1}	0.459	14	MR Egger	
		BMI (SD)	-0.02 (-0.03; -0.00)	3.3×10^{-2}	0.168	16	MR Egger	
		SBP (mmHg)	-0.06 (-0.30; 0.18)	6.2×10^{-1}	0.045	15	MR Egger	
		DBP (mmHg)	0.11 (0.07; 0.15)	6.0×10^{-7}	<0.001	15	IVW	
		ECG heart rate (exercise) (BPM)	0.05 (-0.16; 0.25)	6.5×10^{-1}	0.171	16	IVW	
		ECG load (exercise) (Watts)	-0.36 (-0.94; 0.23)	2.3×10^{-1}	0.655	17	MR Egger	
		Asthma (OR)	1.02 (0.98; 1.06)	2.6×10^{-1}	0.735	18	MR Egger	
		CRP (log(mg/L))	-0.01 (-0.02; 0.00)	7.3×10^{-2}	0.968	15	IVW	
		eGFR (SD of log(eGFR))	-0.00 (-0.00; 0.00)	9.8×10^{-1}	0.043	15	MR Egger	
		BUN (mg/dl)	-0.00 (-0.00; 0.00)	2.4×10^{-1}	0.418	15	IVW	
		CKD (OR)	1.14 (1.04; 1.24)	4.6×10^{-3}	0.144	15	MR Egger	
		Alzheimer's (OR)	1.01 (0.98; 1.03)	5.6×10^{-1}	0.070	15	MR Egger	
		Parkinson's (OR)	1.02 (0.97; 1.06)	4.9×10^{-1}	0.003	15	IVW	
		Lewy body dementia (OR)	1.25 (1.14; 1.36)	1.4×10^{-6}	0.034	15	IVW	
		Breast cancer (OR)	1.08 (0.99; 1.19)	9.5×10^{-2}	0.869	14	MR Egger	
		Lung cancer (OR)	0.95 (0.71; 1.27)	7.4×10^{-1}	0.066	16	MR Egger	
		Colon cancer (OR)	0.95 (0.75; 1.19)	6.4×10^{-1}	0.093	16	MR Egger	
		Prostate cancer (OR)	1.03 (0.92; 1.16)	5.8×10^{-1}	0.475	16	MR Egger	
EGFR (P00533)	Nearest	HF (OR)	1.08 (0.48; 2.43)	8.5×10^{-1}	0.328	5	MR Egger	Interval
		Non-ischemic CM (OR)	1.08 (0.87; 1.35)	4.9×10^{-1}	0.299	5	IVW	
		DCM (OR)	10.76 (0.05; 2155.83)	3.8×10^{-1}	0.191	5	MR Egger	
		AF (OR)	0.99 (0.93; 1.05)	6.8×10^{-1}	0.009	6	IVW	
		CHD (OR)	0.89 (0.83; 0.95)	8.8×10^{-4}	0.036	6	IVW	
		cIMT (mm)	0.09 (0.03; 0.16)	7.5×10^{-3}	0.357	5	MR Egger	
		Carotid plaque (OR)	0.93 (0.84; 1.04)	1.9×10^{-1}	0.380	5	IVW	
		Any stroke (OR)	0.97 (0.92; 1.03)	3.5×10^{-1}	0.515	5	IVW	

Table S16: Phenome-wide drug target MR scan of proteins with a prioritized CMR association. *(continued)*

Protein (uniprot)	Protein set	Trait	Estimate (95%CI)	P-value	Q p-value	No. variants	MR model	pQTL source
		Any ischemic stroke (OR)	0.99 (0.93; 1.05)	7.3×10^{-1}	0.769	5	IVW	
		LDL cholesterol (mmol/l)	0.02 (-0.00; 0.03)	5.4×10^{-2}	0.624	6	IVW	
		Apoliprotein B (g/l)	0.00 (-0.00; 0.01)	1.7×10^{-1}	0.296	6	IVW	
		Triglycerides (mmol/l)	-0.01 (-0.03; 0.01)	4.6×10^{-1}	0.007	6	IVW	
		Cholesterol (mmol/l)	0.02 (0.00; 0.04)	4.6×10^{-2}	0.678	6	IVW	
		HDL cholesterol (mmol/l)	0.01 (-0.00; 0.01)	1.1×10^{-1}	0.030	6	IVW	
		Apoliprotein A1 (g/l)	-0.00 (-0.01; 0.00)	8.2×10^{-1}	0.018	6	IVW	
		Glucose (mmol/l)	-0.00 (-0.03; 0.02)	7.0×10^{-1}	0.364	6	IVW	
		Glycated haemoglobin (mmol/mol)	-0.65 (-1.08; -0.23)	2.5×10^{-3}	0.731	6	MR Egger	
		T2DM (OR)	0.93 (0.90; 0.97)	1.6×10^{-4}	0.917	5	IVW	
		BMI (SD)	0.04 (0.03; 0.05)	1.5×10^{-11}	0.802	5	IVW	
		SBP (mmHg)	-0.14 (-0.32; 0.04)	1.2×10^{-1}	0.950	5	IVW	
		DBP (mmHg)	-0.11 (-0.21; -0.01)	4.0×10^{-2}	0.731	5	IVW	
		ECG heart rate (exercise) (BPM)	0.75 (0.01; 1.50)	4.6×10^{-2}	0.354	6	IVW	
		ECG load (exercise) (Watts)	3.06 (-0.00; 6.12)	5.0×10^{-2}	0.258	6	MR Egger	
		Asthma (OR)	1.00 (0.94; 1.06)	9.6×10^{-1}	0.198	5	IVW	
		CRP (log(mg/L))	0.02 (-0.01; 0.04)	1.9×10^{-1}	0.946	5	IVW	
		eGFR (SD of log(eGFR))	0.00 (0.00; 0.01)	4.0×10^{-3}	0.500	5	IVW	
		BUN (mg/dl)	0.01 (0.00; 0.01)	1.0×10^{-2}	0.336	5	IVW	
		CKD (OR)	1.11 (1.05; 1.17)	3.7×10^{-4}	0.944	5	IVW	
		Alzheimer's (OR)	1.02 (1.00; 1.03)	5.0×10^{-2}	0.803	5	IVW	
		Parkinson's (OR)	2.50 (0.11; 57.89)	5.7×10^{-1}	0.079	5	MR Egger	
		Lewy body dementia (OR)	1.45 (1.08; 1.96)	1.4×10^{-2}	0.593	6	IVW	
		Breast cancer (OR)	0.97 (0.86; 1.10)	6.7×10^{-1}	0.110	6	IVW	
		Lung cancer (OR)	1.18 (0.93; 1.49)	1.7×10^{-1}	0.015	6	IVW	
		Colon cancer (OR)	1.15 (0.95; 1.40)	1.4×10^{-1}	0.428	6	IVW	
		Prostate cancer (OR)	0.94 (0.83; 1.07)	3.6×10^{-1}	0.348	6	IVW	
FA10 (P00742)	Nearest	HF (OR)	1.01 (0.96; 1.07)	6.3×10^{-1}	0.050	13	IVW	Interval
		Non-ischemic CM (OR)	0.90 (0.82; 0.99)	2.9×10^{-2}	0.047	18	IVW	
		DCM (OR)	1.10 (0.98; 1.23)	1.0×10^{-1}	0.016	17	IVW	
		AF (OR)	0.99 (0.97; 1.01)	4.2×10^{-1}	0.002	18	IVW	
		CHD (OR)	0.99 (0.97; 1.01)	3.3×10^{-1}	0.447	20	IVW	
		cIMT (mm)	0.01 (0.00; 0.01)	8.6×10^{-6}	0.064	20	IVW	
		Carotid plaque (OR)	1.07 (0.91; 1.27)	3.9×10^{-1}	0.163	20	MR Egger	
		Any stroke (OR)	1.05 (0.97; 1.15)	2.3×10^{-1}	0.128	21	MR Egger	
		Any ischemic stroke (OR)	1.05 (1.03; 1.08)	1.2×10^{-4}	0.002	20	IVW	
		LDL cholesterol (mmol/l)	0.01 (-0.00; 0.01)	6.8×10^{-2}	0.060	19	IVW	
		Apoliprotein B (g/l)	0.00 (-0.00; 0.00)	1.5×10^{-1}	0.045	19	IVW	
		Triglycerides (mmol/l)	-0.01 (-0.02; 0.01)	4.3×10^{-1}	0.804	21	MR Egger	
		Cholesterol (mmol/l)	0.02 (0.01; 0.02)	2.5×10^{-3}	0.088	19	IVW	
		HDL cholesterol (mmol/l)	0.01 (0.01; 0.01)	2.2×10^{-16}	0.057	21	IVW	
		Apoliprotein A1 (g/l)	0.01 (0.01; 0.01)	1.0×10^{-100}	0.490	21	IVW	
		Glucose (mmol/l)	-0.00 (-0.02; 0.01)	6.5×10^{-1}	0.766	22	MR Egger	
		Glycated haemoglobin (mmol/mol)	0.02 (-0.08; 0.11)	7.2×10^{-1}	0.966	22	MR Egger	
		T2DM (OR)	0.99 (0.95; 1.04)	7.5×10^{-1}	0.449	22	MR Egger	
		BMI (SD)	-0.01 (-0.02; -0.01)	4.7×10^{-5}	0.126	17	IVW	
		SBP (mmHg)	0.05 (-0.04; 0.13)	2.6×10^{-1}	0.270	17	IVW	
		DBP (mmHg)	-0.04 (-0.10; 0.02)	2.0×10^{-1}	0.051	16	IVW	
		ECG heart rate (exercise) (BPM)	0.10 (-0.45; 0.65)	7.2×10^{-1}	0.781	22	MR Egger	
		ECG load (exercise) (Watts)	-0.10 (-0.72; 0.51)	7.5×10^{-1}	0.684	22	MR Egger	
		Asthma (OR)	0.98 (0.95; 1.02)	4.3×10^{-1}	0.005	22	MR Egger	
		CRP (log(mg/L))	0.00 (-0.04; 0.04)	9.6×10^{-1}	0.810	18	MR Egger	
		eGFR (SD of log(eGFR))	0.00 (0.00; 0.00)	1.2×10^{-3}	0.864	19	IVW	
		BUN (mg/dl)	-0.00 (-0.01; 0.00)	2.0×10^{-1}	0.147	19	IVW	
		CKD (OR)	1.00 (0.98; 1.03)	9.5×10^{-1}	0.757	20	IVW	
		Alzheimer's (OR)	0.99 (0.99; 1.00)	9.5×10^{-2}	0.014	20	IVW	
		Parkinson's (OR)	0.95 (0.90; 1.01)	9.3×10^{-2}	0.374	21	IVW	
		Lewy body dementia (OR)	1.22 (1.08; 1.37)	1.0×10^{-3}	0.715	17	IVW	
		Breast cancer (OR)	1.04 (0.99; 1.11)	1.4×10^{-1}	0.188	16	IVW	
		Lung cancer (OR)	0.88 (0.78; 0.98)	1.9×10^{-2}	0.954	17	IVW	
		Colon cancer (OR)	0.89 (0.72; 1.11)	3.0×10^{-1}	0.644	19	MR Egger	
		Prostate cancer (OR)	0.96 (0.90; 1.02)	2.1×10^{-1}	0.141	17	IVW	
IL18 (Q14116)	Nearest	HF (OR)	1.02 (0.99; 1.05)	2.4×10^{-1}	0.340	15	IVW	Scallop
		Non-ischemic CM (OR)	1.14 (0.72; 1.81)	5.8×10^{-1}	0.528	15	MR Egger	
		DCM (OR)	1.49 (0.67; 3.31)	3.3×10^{-1}	0.491	13	MR Egger	
		AF (OR)	1.10 (1.04; 1.16)	2.7×10^{-4}	0.120	14	IVW	

Table S16: Phenome-wide drug target MR scan of proteins with a prioritized CMR association. *(continued)*

Protein (uniprot)	Protein set	Trait	Estimate (95%CI)	P-value	Q p-value	No. variants	MR model	pQTL source
		CHD (OR)	1.00 (0.97; 1.04)	8.0×10^{-1}	0.862	15	IVW	
		cIMT (mm)	0.01 (0.00; 0.01)	9.3×10^{-5}	0.947	16	IVW	
		Carotid plaque (OR)	0.87 (0.82; 0.94)	1.0×10^{-4}	0.628	16	IVW	
		Any stroke (OR)	1.10 (0.95; 1.26)	2.0×10^{-1}	0.985	16	MR Egger	
		Any ischemic stroke (OR)	1.09 (0.93; 1.28)	2.9×10^{-1}	0.357	16	MR Egger	
		LDL cholesterol (mmol/l)	-0.01 (-0.04; 0.03)	7.2×10^{-1}	0.467	15	MR Egger	
		Apoliprotein B (g/l)	-0.01 (-0.01; -0.01)	4.3×10^{-12}	0.660	15	IVW	
		Triglycerides (mmol/l)	-0.02 (-0.03; -0.01)	4.0×10^{-7}	0.861	16	IVW	
		Cholesterol (mmol/l)	-0.01 (-0.06; 0.03)	5.9×10^{-1}	0.611	15	MR Egger	
		HDL cholesterol (mmol/l)	-0.01 (-0.02; 0.01)	5.1×10^{-1}	0.355	15	MR Egger	
		Apoliprotein A1 (g/l)	0.00 (0.00; 0.01)	1.3×10^{-2}	0.415	16	IVW	
		Glucose (mmol/l)	-0.02 (-0.07; 0.03)	3.6×10^{-1}	0.488	15	MR Egger	
		Glycated haemoglobin (mmol/mol)	-0.06 (-0.11; -0.00)	4.7×10^{-2}	0.398	16	IVW	
		T2DM (OR)	1.02 (0.93; 1.11)	7.4×10^{-1}	0.495	14	MR Egger	
		BMI (SD)	-0.02 (-0.05; 0.01)	2.4×10^{-1}	0.077	15	MR Egger	
		SBP (mmHg)	-0.03 (-0.16; 0.09)	5.9×10^{-1}	0.077	12	IVW	
		DBP (mmHg)	-0.16 (-0.23; -0.10)	6.6×10^{-7}	0.202	13	IVW	
		ECG heart rate (exercise) (BPM)	-1.13 (-2.93; 0.66)	2.2×10^{-1}	0.113	15	MR Egger	
		ECG load (exercise) (Watts)	-0.21 (-0.55; 0.13)	2.2×10^{-1}	0.978	16	IVW	
		Asthma (OR)	1.01 (0.99; 1.03)	2.0×10^{-1}	0.387	17	IVW	
		CRP (log(mg/L))	0.03 (0.02; 0.05)	5.7×10^{-5}	0.354	13	IVW	
		eGFR (SD of log(eGFR))	-0.00 (-0.01; 0.00)	5.3×10^{-1}	0.078	15	MR Egger	
		BUN (mg/dl)	-0.01 (-0.01; -0.00)	7.0×10^{-3}	0.258	15	IVW	
		CKD (OR)	1.08 (1.04; 1.12)	1.6×10^{-4}	0.542	15	IVW	
		Alzheimer's (OR)	1.02 (0.96; 1.07)	5.5×10^{-1}	0.284	15	MR Egger	
		Parkinson's (OR)	1.08 (1.00; 1.17)	4.4×10^{-2}	0.646	15	IVW	
		Lewy body dementia (OR)	1.11 (1.00; 1.24)	5.1×10^{-2}	0.287	15	IVW	
		Breast cancer (OR)	1.03 (0.94; 1.13)	4.8×10^{-1}	0.075	11	IVW	
		Lung cancer (OR)	0.82 (0.51; 1.30)	4.0×10^{-1}	0.669	13	MR Egger	
		Colon cancer (OR)	0.89 (0.58; 1.36)	5.9×10^{-1}	0.226	13	MR Egger	
		Prostate cancer (OR)	0.99 (0.78; 1.26)	9.4×10^{-1}	0.915	13	MR Egger	
IL6RB (P40189)	Nearest	HF (OR)	1.04 (0.96; 1.13)	2.8×10^{-1}	0.650	2	IVW	Framingham
		Non-ischemic CM (OR)	1.20 (0.78; 1.86)	4.1×10^{-1}	None	1	Wald	
		DCM (OR)	0.69 (0.44; 1.07)	9.9×10^{-2}	0.293	2	IVW	
		AF (OR)	1.09 (1.01; 1.16)	1.7×10^{-2}	0.484	2	IVW	
		CHD (OR)	1.04 (0.91; 1.18)	6.1×10^{-1}	0.145	2	IVW	
		cIMT (mm)	0.00 (-0.00; 0.01)	4.8×10^{-1}	0.680	2	IVW	
		Carotid plaque (OR)	0.92 (0.75; 1.11)	3.8×10^{-1}	0.270	2	IVW	
		Any stroke (OR)	1.04 (0.91; 1.18)	5.8×10^{-1}	0.173	2	IVW	
		Any ischemic stroke (OR)	1.05 (0.90; 1.23)	5.5×10^{-1}	0.132	2	IVW	
		LDL cholesterol (mmol/l)	-0.00 (-0.02; 0.02)	9.2×10^{-1}	0.879	2	IVW	
		Apoliprotein B (g/l)	-0.00 (-0.01; 0.00)	5.6×10^{-1}	0.940	2	IVW	
		Triglycerides (mmol/l)	-0.04 (-0.06; -0.02)	1.2×10^{-3}	0.568	2	IVW	
		Cholesterol (mmol/l)	0.00 (-0.02; 0.03)	8.9×10^{-1}	0.939	2	IVW	
		HDL cholesterol (mmol/l)	0.01 (0.00; 0.02)	2.4×10^{-2}	0.983	2	IVW	
		Apoliprotein A1 (g/l)	0.00 (-0.00; 0.01)	1.8×10^{-1}	0.287	2	IVW	
		Glucose (mmol/l)	0.01 (-0.03; 0.04)	7.4×10^{-1}	0.502	2	IVW	
		Glycated haemoglobin (mmol/mol)	0.89 (0.17; 1.61)	1.6×10^{-2}	None	2	MR Egger	
		T2DM (OR)	1.00 (0.92; 1.09)	9.2×10^{-1}	None	1	Wald	
		BMI (SD)	0.01 (-0.02; 0.03)	6.6×10^{-1}	None	1	Wald	
		SBP (mmHg)	-0.55 (-0.95; -0.15)	7.4×10^{-3}	None	1	Wald	
		DBP (mmHg)	-0.27 (-0.50; -0.04)	2.1×10^{-2}	None	1	Wald	
		ECG heart rate (exercise) (BPM)	0.14 (-0.73; 1.01)	7.5×10^{-1}	0.731	2	IVW	
		ECG load (exercise) (Watts)	0.91 (-0.06; 1.89)	6.7×10^{-2}	0.739	2	IVW	
		Asthma (OR)	1.08 (0.99; 1.18)	9.1×10^{-2}	0.155	2	IVW	
		CRP (log(mg/L))	0.03 (-0.01; 0.08)	1.4×10^{-1}	0.859	2	IVW	
		eGFR (SD of log(eGFR))	0.00 (-0.00; 0.01)	1.1×10^{-1}	0.259	2	IVW	
		BUN (mg/dl)	-0.01 (-0.02; 0.00)	9.6×10^{-2}	None	1	Wald	
		CKD (OR)	0.94 (0.81; 1.08)	3.6×10^{-1}	0.138	2	IVW	
		Alzheimer's (OR)	1.00 (0.98; 1.02)	7.7×10^{-1}	0.481	2	IVW	
		Parkinson's (OR)	1.01 (0.80; 1.27)	9.5×10^{-1}	None	1	Wald	
		Lewy body dementia (OR)	1.16 (0.71; 1.90)	5.6×10^{-1}	None	1	Wald	
		Breast cancer (OR)	0.94 (0.84; 1.05)	2.6×10^{-1}	0.788	2	IVW	
		Lung cancer (OR)	0.97 (0.73; 1.29)	8.2×10^{-1}	0.609	2	IVW	
		Colon cancer (OR)	0.87 (0.68; 1.10)	2.4×10^{-1}	0.965	2	IVW	
		Prostate cancer (OR)	0.94 (0.82; 1.09)	4.5×10^{-1}	0.903	2	IVW	

Table S16: Phenome-wide drug target MR scan of proteins with a prioritized CMR association. *(continued)*

Protein (uniprot)	Protein set	Trait	Estimate (95%CI)	P-value	Q p-value	No. variants	MR model	pQTL source
LYAM2 (P16581)	Nearest	HF (OR)	1.02 (0.92; 1.14)	6.7×10^{-1}	0.478	8	IVW	Scallop
		Non-ischemic CM (OR)	5.73 (1.22; 26.88)	2.7×10^{-2}	0.962	8	MR Egger	
		DCM (OR)	0.51 (0.23; 1.12)	9.4×10^{-2}	0.115	7	IVW	
		AF (OR)	1.27 (1.14; 1.41)	1.1×10^{-5}	0.480	7	IVW	
		CHD (OR)	0.94 (0.70; 1.25)	6.7×10^{-1}	0.617	9	MR Egger	
		cIMT (mm)	-0.01 (-0.02; -0.00)	4.4×10^{-2}	0.327	8	IVW	
		Carotid plaque (OR)	1.05 (0.43; 2.55)	9.1×10^{-1}	0.251	8	MR Egger	
		Any stroke (OR)	1.21 (0.65; 2.23)	5.5×10^{-1}	0.080	8	MR Egger	
		Any ischemic stroke (OR)	1.16 (0.98; 1.37)	8.0×10^{-2}	0.112	8	IVW	
		LDL cholesterol (mmol/l)	0.03 (-0.07; 0.12)	5.6×10^{-1}	0.295	8	MR Egger	
		Apoliprotein B (g/l)	0.00 (-0.02; 0.03)	7.6×10^{-1}	0.365	8	MR Egger	
		Triglycerides (mmol/l)	0.10 (-0.01; 0.20)	7.5×10^{-2}	0.381	8	MR Egger	
		Cholesterol (mmol/l)	0.03 (-0.01; 0.07)	1.5×10^{-1}	0.295	7	IVW	
		HDL cholesterol (mmol/l)	-0.01 (-0.06; 0.04)	7.6×10^{-1}	0.120	8	MR Egger	
		Apoliprotein A1 (g/l)	0.00 (-0.03; 0.03)	9.2×10^{-1}	0.266	8	MR Egger	
		Glucose (mmol/l)	0.01 (-0.02; 0.04)	4.8×10^{-1}	0.371	8	IVW	
		Glycated haemoglobin (mmol/mol)	-0.21 (-0.37; -0.06)	6.9×10^{-3}	0.456	8	IVW	
		T2DM (OR)	1.04 (0.99; 1.09)	1.6×10^{-1}	0.700	9	IVW	
		BMI (SD)	0.01 (-0.00; 0.03)	1.1×10^{-1}	0.384	9	IVW	
		SBP (mmHg)	1.13 (-0.59; 2.86)	2.0×10^{-1}	0.201	8	MR Egger	
		DBP (mmHg)	0.94 (0.05; 1.84)	3.9×10^{-2}	0.319	8	MR Egger	
		ECG heart rate (exercise) (BPM)	-1.70 (-5.38; 1.98)	3.6×10^{-1}	0.625	8	MR Egger	
		ECG load (exercise) (Watts)	-1.53 (-2.52; -0.54)	2.5×10^{-3}	0.912	8	IVW	
		Asthma (OR)	1.05 (0.98; 1.12)	1.7×10^{-1}	0.318	8	IVW	
		CRP (log(mg/L))	0.05 (-0.01; 0.11)	1.2×10^{-1}	0.349	6	IVW	
		eGFR (SD of log(eGFR))	0.00 (-0.00; 0.01)	1.0×10^{-1}	0.784	8	IVW	
		BUN (mg/dl)	0.01 (-0.05; 0.07)	8.1×10^{-1}	0.097	8	MR Egger	
		CKD (OR)	0.99 (0.88; 1.12)	8.9×10^{-1}	0.720	8	IVW	
		Alzheimer's (OR)	0.97 (0.94; 1.00)	5.6×10^{-2}	0.873	9	IVW	
		Parkinson's (OR)	0.97 (0.83; 1.13)	6.9×10^{-1}	0.263	9	IVW	
		Lewy body dementia (OR)	2.01 (1.04; 3.89)	3.7×10^{-2}	0.496	6	IVW	
		Breast cancer (OR)	1.16 (0.99; 1.37)	7.4×10^{-2}	0.611	8	IVW	
		Lung cancer (OR)	2.34 (0.45; 12.11)	3.1×10^{-1}	0.875	9	MR Egger	
		Colon cancer (OR)	1.36 (0.35; 5.25)	6.6×10^{-1}	0.459	9	MR Egger	
		Prostate cancer (OR)	0.72 (0.53; 0.97)	3.1×10^{-2}	0.064	7	IVW	
MET (P08581)	Nearest	HF (OR)	1.05 (0.96; 1.16)	2.7×10^{-1}	0.266	5	IVW	Interval
		Non-ischemic CM (OR)	0.92 (0.73; 1.16)	4.9×10^{-1}	0.621	6	IVW	
		DCM (OR)	1.01 (0.73; 1.39)	9.6×10^{-1}	0.720	6	IVW	
		AF (OR)	0.96 (0.91; 1.02)	1.7×10^{-1}	0.290	6	IVW	
		CHD (OR)	0.97 (0.91; 1.04)	3.6×10^{-1}	0.509	6	IVW	
		cIMT (mm)	-0.00 (-0.01; 0.00)	3.0×10^{-1}	0.403	6	IVW	
		Carotid plaque (OR)	0.98 (0.82; 1.17)	7.9×10^{-1}	0.130	6	IVW	
		Any stroke (OR)	1.14 (1.06; 1.23)	7.7×10^{-4}	0.684	6	IVW	
		Any ischemic stroke (OR)	1.11 (1.02; 1.20)	1.3×10^{-2}	0.845	6	IVW	
		LDL cholesterol (mmol/l)	0.02 (-0.00; 0.04)	5.7×10^{-2}	0.670	6	IVW	
		Apoliprotein B (g/l)	0.00 (-0.00; 0.01)	8.8×10^{-2}	0.422	6	IVW	
		Triglycerides (mmol/l)	0.01 (-0.01; 0.04)	3.4×10^{-1}	0.016	4	IVW	
		Cholesterol (mmol/l)	0.03 (0.01; 0.06)	1.4×10^{-2}	0.856	6	IVW	
		HDL cholesterol (mmol/l)	0.01 (-0.00; 0.02)	8.6×10^{-2}	0.127	6	IVW	
		Apoliprotein A1 (g/l)	0.01 (0.01; 0.02)	3.3×10^{-5}	0.418	6	IVW	
		Glucose (mmol/l)	0.01 (-0.03; 0.04)	7.1×10^{-1}	0.340	6	IVW	
		Glycated haemoglobin (mmol/mol)	-0.01 (-0.17; 0.16)	9.2×10^{-1}	0.321	6	IVW	
		T2DM (OR)	0.95 (0.90; 1.01)	7.8×10^{-2}	0.187	6	IVW	
		BMI (SD)	-0.00 (-0.01; 0.01)	8.2×10^{-1}	0.012	6	IVW	
		SBP (mmHg)	0.55 (0.04; 1.07)	3.3×10^{-2}	0.077	4	IVW	
		DBP (mmHg)	-0.30 (-0.72; 0.13)	1.7×10^{-1}	0.702	7	MR Egger	
		ECG heart rate (exercise) (BPM)	-0.09 (-0.94; 0.77)	8.4×10^{-1}	0.527	6	IVW	
		ECG load (exercise) (Watts)	-0.42 (-1.38; 0.54)	3.9×10^{-1}	0.665	6	IVW	
		Asthma (OR)	0.99 (0.94; 1.05)	8.6×10^{-1}	0.717	6	IVW	
		CRP (log(mg/L))	-0.05 (-0.08; -0.01)	9.5×10^{-3}	0.638	6	IVW	
		eGFR (SD of log(eGFR))	-0.00 (-0.01; 0.00)	5.2×10^{-1}	0.333	5	IVW	
		BUN (mg/dl)	0.01 (-0.00; 0.01)	1.4×10^{-1}	0.681	6	IVW	
		CKD (OR)	1.01 (0.93; 1.09)	8.2×10^{-1}	0.817	6	IVW	
		Alzheimer's (OR)	1.01 (0.99; 1.03)	1.8×10^{-1}	0.284	6	IVW	
		Parkinson's (OR)	1.26 (0.77; 2.05)	3.5×10^{-1}	0.012	7	MR Egger	
		Lewy body dementia (OR)	1.23 (0.78; 1.95)	3.7×10^{-1}	0.163	5	IVW	

Table S16: Phenome-wide drug target MR scan of proteins with a prioritized CMR association. *(continued)*

Protein (uniprot)	Protein set	Trait	Estimate (95%CI)	P-value	Q p-value	No. variants	MR model	pQTL source
		Breast cancer (OR)	1.48 (1.15; 1.92)	2.8×10^{-3}	0.960	7	MR Egger	
		Lung cancer (OR)	1.08 (0.87; 1.34)	4.7×10^{-1}	0.415	6	IVW	
		Colon cancer (OR)	1.03 (0.87; 1.22)	7.2×10^{-1}	0.954	6	IVW	
		Prostate cancer (OR)	0.92 (0.83; 1.02)	9.7×10^{-2}	0.529	6	IVW	
PAI1 (P05121)	Nearest	HF (OR)	1.04 (0.90; 1.20)	6.3×10^{-1}	None	1	Wald	Framingham
		Non-ischemic CM (OR)	1.11 (0.60; 2.06)	7.4×10^{-1}	None	1	Wald	
		DCM (OR)	0.43 (0.21; 0.89)	2.3×10^{-2}	None	1	Wald	
		AF (OR)	1.23 (1.09; 1.39)	1.1×10^{-3}	None	1	Wald	
		CHD (OR)	1.11 (0.96; 1.30)	1.6×10^{-1}	None	1	Wald	
		cIMT (mm)	-0.01 (-0.02; 0.01)	2.1×10^{-1}	None	1	Wald	
		Carotid plaque (OR)	1.00 (0.71; 1.40)	9.9×10^{-1}	None	1	Wald	
		Any stroke (OR)	0.97 (0.81; 1.15)	7.1×10^{-1}	None	1	Wald	
		Any ischemic stroke (OR)	0.98 (0.82; 1.19)	8.6×10^{-1}	None	1	Wald	
		LDL cholesterol (mmol/l)	-0.00 (-0.04; 0.04)	9.8×10^{-1}	None	1	Wald	
		Apoliprotein B (g/l)	-0.00 (-0.01; 0.01)	6.8×10^{-1}	None	1	Wald	
		Triglycerides (mmol/l)	-0.05 (-0.09; -0.00)	3.9×10^{-2}	None	1	Wald	
		Cholesterol (mmol/l)	0.01 (-0.04; 0.06)	6.2×10^{-1}	None	1	Wald	
		HDL cholesterol (mmol/l)	0.02 (0.01; 0.04)	8.7×10^{-3}	None	1	Wald	
		Apoliprotein A1 (g/l)	0.01 (0.00; 0.03)	1.4×10^{-2}	None	1	Wald	
		Glucose (mmol/l)	0.05 (-0.01; 0.10)	1.1×10^{-1}	None	1	Wald	
		Glycated haemoglobin (mmol/mol)	0.10 (-0.18; 0.39)	4.7×10^{-1}	None	1	Wald	
		T2DM (OR)	1.04 (0.92; 1.17)	5.3×10^{-1}	None	1	Wald	
		BMI (SD)	-0.04 (-0.07; -0.01)	1.3×10^{-2}	None	1	Wald	
		SBP (mmHg)	0.51 (-0.05; 1.07)	7.2×10^{-2}	None	1	Wald	
		DBP (mmHg)	-0.24 (-0.56; 0.07)	1.3×10^{-1}	None	1	Wald	
		ECG heart rate (exercise) (BPM)	-1.35 (-2.96; 0.25)	9.9×10^{-2}	None	1	Wald	
		ECG load (exercise) (Watts)	1.01 (-0.79; 2.81)	2.7×10^{-1}	None	1	Wald	
		Asthma (OR)	0.96 (0.85; 1.07)	4.2×10^{-1}	None	1	Wald	
		CRP (log(mg/L))	-0.09 (-0.16; -0.01)	2.4×10^{-2}	None	1	Wald	
		eGFR (SD of log(eGFR))	0.00 (-0.01; 0.01)	7.6×10^{-1}	None	1	Wald	
		BUN (mg/dl)	0.01 (-0.01; 0.03)	2.2×10^{-1}	None	1	Wald	
		CKD (OR)	1.11 (0.94; 1.32)	2.1×10^{-1}	None	1	Wald	
		Alzheimer's (OR)	1.04 (1.00; 1.08)	3.9×10^{-2}	None	1	Wald	
		Parkinson's (OR)	1.18 (0.83; 1.67)	3.6×10^{-1}	None	1	Wald	
		Lewy body dementia (OR)	1.17 (0.59; 2.33)	6.5×10^{-1}	None	1	Wald	
		Breast cancer (OR)	1.03 (0.83; 1.26)	8.1×10^{-1}	None	1	Wald	
		Lung cancer (OR)	0.98 (0.57; 1.66)	9.3×10^{-1}	None	1	Wald	
		Colon cancer (OR)	1.02 (0.67; 1.57)	9.2×10^{-1}	None	1	Wald	
		Prostate cancer (OR)	1.05 (0.80; 1.37)	7.4×10^{-1}	None	1	Wald	
SYUA (P37840)	Nearest	HF (OR)	1.27 (0.88; 1.83)	2.0×10^{-1}	0.061	4	MR Egger	Interval
		Non-ischemic CM (OR)	1.42 (1.05; 1.94)	2.4×10^{-2}	0.811	4	IVW	
		DCM (OR)	2.35 (0.60; 9.13)	2.2×10^{-1}	0.242	4	MR Egger	
		AF (OR)	1.00 (0.94; 1.07)	9.1×10^{-1}	0.400	4	IVW	
		CHD (OR)	1.19 (0.91; 1.55)	2.0×10^{-1}	0.281	4	MR Egger	
		cIMT (mm)	0.00 (-0.00; 0.01)	3.1×10^{-1}	0.320	4	IVW	
		Carotid plaque (OR)	1.09 (0.93; 1.27)	2.8×10^{-1}	0.797	4	IVW	
		Any stroke (OR)	1.08 (0.99; 1.18)	7.3×10^{-2}	0.732	4	IVW	
		Any ischemic stroke (OR)	1.12 (1.02; 1.23)	2.1×10^{-2}	0.382	4	IVW	
		LDL cholesterol (mmol/l)	-0.00 (-0.02; 0.01)	8.4×10^{-1}	0.943	5	IVW	
		Apoliprotein B (g/l)	-0.00 (-0.01; 0.00)	3.6×10^{-1}	0.889	5	IVW	
		Triglycerides (mmol/l)	-0.01 (-0.03; 0.01)	1.8×10^{-1}	0.778	5	IVW	
		Cholesterol (mmol/l)	0.00 (-0.02; 0.02)	6.6×10^{-1}	0.581	5	IVW	
		HDL cholesterol (mmol/l)	-0.00 (-0.01; 0.01)	9.5×10^{-1}	0.011	5	MR Egger	
		Apoliprotein A1 (g/l)	-0.01 (-0.01; 0.00)	9.9×10^{-2}	0.006	5	MR Egger	
		Glucose (mmol/l)	-0.00 (-0.03; 0.02)	6.9×10^{-1}	0.763	5	IVW	
		Glycated haemoglobin (mmol/mol)	-0.25 (-0.40; -0.10)	1.2×10^{-3}	0.129	5	IVW	
		T2DM (OR)	1.07 (0.82; 1.38)	6.2×10^{-1}	0.106	4	MR Egger	
		BMI (SD)	-0.00 (-0.06; 0.05)	8.7×10^{-1}	0.251	4	MR Egger	
		SBP (mmHg)	-0.13 (-0.41; 0.15)	3.7×10^{-1}	0.696	4	IVW	
		DBP (mmHg)	-0.02 (-0.18; 0.14)	7.9×10^{-1}	0.910	4	IVW	
		ECG heart rate (exercise) (BPM)	-0.66 (-1.65; 0.33)	1.9×10^{-1}	0.021	5	MR Egger	
		ECG load (exercise) (Watts)	-0.15 (-1.54; 1.24)	8.3×10^{-1}	0.194	5	MR Egger	
		Asthma (OR)	1.03 (0.98; 1.07)	2.1×10^{-1}	0.953	5	IVW	
		CRP (log(mg/L))	0.01 (-0.03; 0.05)	7.1×10^{-1}	0.796	4	IVW	
		eGFR (SD of log(eGFR))	0.01 (-0.00; 0.02)	5.2×10^{-2}	0.422	4	MR Egger	
		BUN (mg/dl)	-0.01 (-0.01; 0.00)	1.6×10^{-1}	0.377	4	IVW	

Table S16: Phenome-wide drug target MR scan of proteins with a prioritized CMR association. *(continued)*

Protein (uniprot)	Protein set	Trait	Estimate (95%CI)	P-value	Q p-value	No. variants	MR model	pQTL source
		CKD (OR)	0.97 (0.89; 1.06)	4.8×10^{-1}	0.562	4	IVW	
		Alzheimer's (OR)	0.97 (0.92; 1.03)	3.6×10^{-1}	0.043	4	MR Egger	
		Parkinson's (OR)	0.36 (0.28; 0.47)	7.3×10^{-15}	0.494	2	IVW	
		Lewy body dementia (OR)	0.34 (0.20; 0.58)	6.2×10^{-5}	0.683	2	IVW	
		Breast cancer (OR)	0.91 (0.81; 1.01)	8.6×10^{-2}	0.618	4	IVW	
		Lung cancer (OR)	0.46 (0.16; 1.34)	1.5×10^{-1}	0.045	4	MR Egger	
		Colon cancer (OR)	1.00 (0.80; 1.26)	9.7×10^{-1}	0.449	4	IVW	
		Prostate cancer (OR)	0.91 (0.78; 1.04)	1.7×10^{-1}	0.574	4	IVW	

General:
CMR: Cardiac MRI, MR: Mendelian randomization, MD: mean difference, CI: confidence interval. Effect estimates are presented per protein standard deviation increase.

Table S17: Comparing the Schmidt et al. discovered CMR genes to three recent CMR GWAS publications.

Index gene	Nearest gene (Schmidt et al.)	Putative causal gene (Schmidt et al.)	Pirruccello et al. 2020	Pirruccello et al. 2022	Aung et al.	CMR trait
ALDH2 (ENSG00000111275)	MAPKAPK5-AS1 (ENSG00000234608)	ALDH2 (ENSG00000111275)				RV – PER
ALPK3 (ENSG00000136383)	SNORA25 (ENSG00000200991)	ALPK3 (ENSG00000136383)		ALPK3 (ENSG00000136383)		LV – ESV
	SNORA25 (ENSG00000200991)	ALPK3 (ENSG00000136383)		ALPK3 (ENSG00000136383)		LV – EF
	ALPK3 (ENSG00000136383)	ALPK3 (ENSG00000136383)		ALPK3 (ENSG00000136383)		LV – MVR
				ALPK3 (ENSG00000136383)		LV – EDV
ATXN2 (ENSG00000204842)				ATXN2 (ENSG00000204842)		LV – ESV
	U7 (ENSG00000272215)	ATXN2 (ENSG00000204842)	ATXN2 (ENSG00000204842)	ATXN2 (ENSG00000204842)		LV – EDV
	U7 (ENSG00000272215)	ATXN2 (ENSG00000204842)	ATXN2 (ENSG00000204842)	ATXN2 (ENSG00000204842)		LV – SV
	U7 (ENSG00000272215)	ATXN2 (ENSG00000204842)		ATXN2 (ENSG00000204842)	ATXN2 (ENSG00000204842)	RV – ESV
	ATXN2 (ENSG00000204842)	ATXN2 (ENSG00000204842)			ATXN2 (ENSG00000204842)	RV – SV
	ATXN2 (ENSG00000204842)	ATXN2 (ENSG00000204842)		ATXN2 (ENSG00000204842)	ATXN2 (ENSG00000204842)	RV – EDV
BAG3 (ENSG00000151929)					BAG3 (ENSG00000151929)	RV – EDV
	BAG3 (ENSG00000151929)	BAG3 (ENSG00000151929)	BAG3 (ENSG00000151929)			LV – EDV
	RGS10 (ENSG00000148908)	BAG3 (ENSG00000151929)		BAG3 (ENSG00000151929)	BAG3 (ENSG00000151929)	RV – ESV
	BAG3 (ENSG00000151929)	BAG3 (ENSG00000151929)	BAG3 (ENSG00000151929)	BAG3 (ENSG00000151929)		LV – ESV
	BAG3 (ENSG00000151929)	BAG3 (ENSG00000151929)	BAG3 (ENSG00000151929)	BAG3 (ENSG00000151929)		LV – EF
	BAG3 (ENSG00000151929)	BAG3 (ENSG00000151929)		BAG3 (ENSG00000151929)	BAG3 (ENSG00000151929)	RV – EF
	BAG3 (ENSG00000151929)	BAG3 (ENSG00000151929)				RV – ESV
CCDC136 (ENSG00000128596)				FLNC (ENSG00000128591)		LV – ESV
			FLNC (ENSG00000128591)	FLNC (ENSG00000128591)		LV – EF
	FLNC (ENSG00000128591)	CCDC136 (ENSG00000128596)	FLNC (ENSG00000128591)	CCDC136 (ENSG00000128596)		LV – ESV
CDKN1A (ENSG00000124762)			CDKN1A (ENSG00000124762)	CDKN1A (ENSG00000124762)		LV – ESV
	CDKN1A (ENSG00000124762)	CDKN1A (ENSG00000124762)				LV – MVR
	CDKN1A (ENSG00000124762)	CDKN1A (ENSG00000124762)	CDKN1A (ENSG00000124762)	CDKN1A (ENSG00000124762)		LV – EF
	RNU1-88P (ENSG00000238554)	CDKN1A (ENSG00000124762)				LV – EDM
CMSS1 (ENSG00000184220)				FILIP1L (ENSG00000168386)		RV – SV
	FILIP1L (ENSG00000168386)	CMSS1 (ENSG00000184220)		FILIP1L (ENSG00000168386)		RV – ESV
	FILIP1L (ENSG00000168386)	CMSS1 (ENSG00000184220)		FILIP1L (ENSG00000168386)		RV – EDV
CRK (ENSG00000167193)	MYO1C (ENSG00000197879)	CRK (ENSG00000167193)	MYO1C (ENSG00000197879)	MYO1C (ENSG00000197879)		LV – ESV
	MYO1C (ENSG00000197879)	CRK (ENSG00000167193)	MYO1C (ENSG00000197879)			LV – EDV
CTSB (ENSG00000164733)	OR7E161P (ENSG00000206014)	CTSB (ENSG00000164733)				LV – EF
DES (ENSG00000175084)	INHA (ENSG00000123999)	DES (ENSG00000175084)				LV – MVR
DMPK (ENSG00000104936)	RSPH6A (ENSG00000104941)	DMPK (ENSG00000104936)		RSPH6A (ENSG00000104941)		RV – EDV
	RSPH6A (ENSG00000104941)	DMPK (ENSG00000104936)		DMPK (ENSG00000104936)		RV – ESV
	RSPH6A (ENSG00000104941)	DMPK (ENSG00000104936)		RSPH6A (ENSG00000104941)	RSPH6A (ENSG00000104941)	RV – ESV
	RSPH6A (ENSG00000104941)	DMPK (ENSG00000104936)	RSPH6A (ENSG00000104941)	RSPH6A (ENSG00000104941)		LV – EDV
	RSPH6A (ENSG00000104941)	DMPK (ENSG00000104936)	RSPH6A (ENSG00000104941)	RSPH6A (ENSG00000104941)		LV – ESV
FHOD3 (ENSG00000134775)					FHOD3 (ENSG00000134775)	RV – ESV
			FHOD3 (ENSG00000134775)	FHOD3 (ENSG00000134775)		LV – EF
	SNORD112 (ENSG00000252078)	FHOD3 (ENSG00000134775)		FHOD3 (ENSG00000134775)	FHOD3 (ENSG00000134775)	RV – EF
GOSR2 (ENSG00000108433)					GOSR2 (ENSG00000108433)	RV – EF
	RP11-156P1.2 (ENSG00000262633)	GOSR2 (ENSG00000108433)		GOSR2 (ENSG00000108433)	GOSR2 (ENSG00000108433)	RV – ESV
	RP11-156P1.2 (ENSG00000262633)	GOSR2 (ENSG00000108433)		GOSR2 (ENSG00000108433)	GOSR2 (ENSG00000108433)	RV – EDV
HIST3H3 (ENSG00000168148)	HIST3H3 (ENSG00000168148)	HIST3H3 (ENSG00000168148)				RV – ESV
HLA-B (ENSG00000234745)			HLA-B (ENSG00000234745)	HLA-B (ENSG00000234745)		LV – EDV
				HLA-B (ENSG00000234745)		RV – EDV
	HLA-B (ENSG00000234745)	HLA-B (ENSG00000234745)				LV – MVR
	HLA-B (ENSG00000234745)	HLA-B (ENSG00000234745)				RV – PER
	HLA-B (ENSG00000234745)	HLA-B (ENSG00000234745)	HLA-B (ENSG00000234745)	HLA-B (ENSG00000234745)		LV – SV
HLA-DRB1 (ENSG00000196126)	XXbac-BPG254F23.6 (ENSG00000241287)	HLA-DRB1 (ENSG00000196126)				LV – EF
HMGA2 (ENSG00000149948)				HMGA2 (ENSG00000149948)		LV – EDV
				HMGA2 (ENSG00000149948)		LV – ESV
				HMGA2 (ENSG00000149948)		LV – SV
				HMGA2 (ENSG00000149948)		RV – ESV
				HMGA2 (ENSG00000149948)		RV – SV
	HMGA2 (ENSG00000149948)	HMGA2 (ENSG00000149948)		HMGA2 (ENSG00000149948)		RV – EDV
HSPB7 (ENSG00000173641)	HSPB7 (ENSG00000173641)	HSPB7 (ENSG00000173641)				RV – EF
	HSPB7 (ENSG00000173641)	HSPB7 (ENSG00000173641)	HSPB7 (ENSG00000173641)	HSPB7 (ENSG00000173641)		LV – ESV
	HSPB7 (ENSG00000173641)	HSPB7 (ENSG00000173641)	HSPB7 (ENSG00000173641)			LV – EF
IGF1R (ENSG00000140443)	MIR4714 (ENSG00000264480)	IGF1R (ENSG00000140443)				LV – EDM
	MIR4714 (ENSG00000264480)	IGF1R (ENSG00000140443)				LV – MVR
KANSL1 (ENSG00000120071)	DND1P1 (ENSG00000264070)	KANSL1 (ENSG00000120071)				LV – MVR
	ARL17B (ENSG00000228696)	KANSL1 (ENSG00000120071)				LV – EDM
KCNH2 (ENSG00000055118)			NOS3 (ENSG00000164867)			LV – EDV
	NOS3 (ENSG00000164867)	KCNH2 (ENSG00000055118)				RV – EDV
	NOS3 (ENSG00000164867)	KCNH2 (ENSG00000055118)				RV – SV
KCNK3 (ENSG00000171303)	KCNK3 (ENSG00000171303)	KCNK3 (ENSG00000171303)		KCNK3 (ENSG00000171303)		RV – ESV
	KCNK3 (ENSG00000171303)	KCNK3 (ENSG00000171303)				RV – EDV
LMF1 (ENSG00000103227)			LMF1 (ENSG00000103227)	LMF1 (ENSG00000103227)		LV – ESV
			LMF1 (ENSG00000103227)			LV – EF

Table S17: Comparing the Schmidt et al. discovered CMR genes to three recent CMR GWAS publications. *(continued)*

Index gene	Nearest gene (Schmidt et al.)	Putative causal gene (Schmidt et al.)	Pirruccello et al. 2020	Pirruccello et al. 2022	Aung et al.	CMR trait
	LA16c-306A4.2 (ENSG00000260316)	LMF1 (ENSG00000103227)				LV – MVR
LRLE1 (ENSG00000268955)	ALG1L13P (ENSG00000253981)	LRLE1 (ENSG00000268955)				RV – EF
MLF1 (ENSG00000178053)			MLF1 (ENSG00000178053)	MLF1 (ENSG00000178053)		LV – ESV
	RP11-113A11.1 (ENSG00000241723)	MLF1 (ENSG00000178053)				RV – EDV
	MLF1 (ENSG00000178053)	MLF1 (ENSG00000178053)		MLF1 (ENSG00000178053)		RV – ESV
	MLF1 (ENSG00000178053)	MLF1 (ENSG00000178053)		MLF1 (ENSG00000178053)		RV – EF
MTMR9 (ENSG00000104643)	AF131215.4 (ENSG00000254556)	MTMR9 (ENSG00000104643)				RV – EF
MYO18B (ENSG00000133454)	MYO18B (ENSG00000133454)	MYO18B (ENSG00000133454)				LV – MVR
NEDD4L (ENSG00000049759)	RP11-845C23.2 (ENSG00000267504)	NEDD4L (ENSG00000049759)	NEDD4L (ENSG00000049759)			LV – EF
	RP11-718I15.1 (ENSG00000267743)	NEDD4L (ENSG00000049759)	NEDD4L (ENSG00000049759)			LV – ESV
NSF (ENSG00000073969)	RPS7P11 (ENSG00000213326)	NSF (ENSG00000073969)				LV – MVR
OBSCN (ENSG00000154358)					OBSCN (ENSG00000154358)	RV – EDV
				OBSCN (ENSG00000154358)	OBSCN (ENSG00000154358)	RV – ESV
	OBSCN (ENSG00000154358)	OBSCN (ENSG00000154358)			OBSCN (ENSG00000154358)	RV – EF
	OBSCN (ENSG00000154358)	OBSCN (ENSG00000154358)				LV – ESV
PDE5A (ENSG00000138735)	PDE5A (ENSG00000138735)	PDE5A (ENSG00000138735)				RV – ESV
PLEC (ENSG00000178209)	MIR661 (ENSG00000207574)	PLEC (ENSG00000178209)		PLEC (ENSG00000178209)	PLEC (ENSG00000178209)	RV – EF
	MIR661 (ENSG00000207574)	PLEC (ENSG00000178209)		PLEC (ENSG00000178209)		RV – ESV
	MIR661 (ENSG00000207574)	PLEC (ENSG00000178209)				LV – EF
PLN (ENSG00000198523)			PLN (ENSG00000198523)			LV – SV
					SLC35F1 (ENSG00000196376)	RV – SV
			PLN (ENSG00000198523)	SLC35F1 (ENSG00000196376)		LV – ESV
	SLC35F1 (ENSG00000196376)	PLN (ENSG00000198523)				LV – MVR
	SLC35F1 (ENSG00000196376)	PLN (ENSG00000198523)	PLN (ENSG00000198523)	SLC35F1 (ENSG00000196376)		LV – EDV
PPP1R1B (ENSG00000131771)	STARD3 (ENSG00000131748)	PPP1R1B (ENSG00000131771)				LV – EDM
PROB1 (ENSG00000228672)	DNAJC18 (ENSG00000170464)	PROB1 (ENSG00000228672)			SLC23A1 (ENSG00000170482)	RV – EF
	SLC23A1 (ENSG00000170482)	PROB1 (ENSG00000228672)				RV – ESV
	DNAJC18 (ENSG00000170464)	PROB1 (ENSG00000228672)	PROB1 (ENSG00000228672)	PROB1 (ENSG00000228672)		LV – ESV
	DNAJC18 (ENSG00000170464)	PROB1 (ENSG00000228672)	DNAJC18 (ENSG00000170464)	PROB1 (ENSG00000228672)		LV – EF
PROM1 (ENSG00000007062)	RNU6-350P (ENSG00000251758)	PROM1 (ENSG00000007062)				LV – EF
PTPN11 (ENSG00000179295)					PTPN11 (ENSG00000179295)	RV – ESV
	PTPN11 (ENSG00000179295)	PTPN11 (ENSG00000179295)			PTPN11 (ENSG00000179295)	RV – SV
	PTPN11 (ENSG00000179295)	PTPN11 (ENSG00000179295)			PTPN11 (ENSG00000179295)	RV – EDV
RP11-457K10.1 (ENSG00000242029)	RP11-457K10.1 (ENSG00000242029)	RP11-457K10.1 (ENSG00000242029)				LV – SV
SCN10A (ENSG00000185313)	SCN10A (ENSG00000185313)	SCN10A (ENSG00000185313)				RV – PAFR
SIPA1L1 (ENSG00000197555)	RP1-261D10.1 (ENSG00000259079)	SIPA1L1 (ENSG00000197555)				LV – MVR
SKI (ENSG00000157933)			C1orf86 (ENSG00000162585)			LV – EF
	C1orf86 (ENSG00000162585)	SKI (ENSG00000157933)				LV – MVR
SMARCB1 (ENSG00000099956)	DERL3 (ENSG00000099958)	SMARCB1 (ENSG00000099956)				LV – MVR
	DERL3 (ENSG00000099958)	SMARCB1 (ENSG00000099956)	DERL3 (ENSG00000099958)	SMARCB1 (ENSG00000099956)		LV – ESV
	DERL3 (ENSG00000099958)	SMARCB1 (ENSG00000099956)	SMARCB1 (ENSG00000099956)	SMARCB1 (ENSG00000099956)		LV – EF
SPON1 (ENSG00000152268)	SPON1 (ENSG00000152268)	SPON1 (ENSG00000152268)				LV – ESV
	SPON1 (ENSG00000152268)	SPON1 (ENSG00000152268)				RV – ESV
SYNPO2L (ENSG00000166317)	SYNPO2L (ENSG00000166317)	SYNPO2L (ENSG00000166317)				LV – EDM
SYT10 (ENSG00000110975)	SYT10 (ENSG00000110975)	SYT10 (ENSG00000110975)				LV – SV
TBX5 (ENSG00000089225)	TBX5-AS1 (ENSG00000255399)	TBX5 (ENSG00000089225)				RV – ESV
TGFBR3 (ENSG00000069702)	RN7SL653P (ENSG00000239794)	TGFBR3 (ENSG00000069702)				RV – ESV
TGM2 (ENSG00000198959)	KIAA1755 (ENSG00000149633)	TGM2 (ENSG00000198959)				RV – SV
TMEM43 (ENSG00000170876)	RP11-536I6.2 (ENSG00000255021)	TMEM43 (ENSG00000170876)				RV – EF
	RNA5SP124 (ENSG00000199609)	TMEM43 (ENSG00000170876)				RV – ESV
	RP11-536I6.2 (ENSG00000255021)	TMEM43 (ENSG00000170876)				RV – ESV
	RP11-536I6.2 (ENSG00000255021)	TMEM43 (ENSG00000170876)				LV – ESV
	RP11-536I6.2 (ENSG00000255021)	TMEM43 (ENSG00000170876)				LV – EF
TNKS (ENSG00000173273)				TNKS (ENSG00000173273)		RV – ESV
	MIR597 (ENSG00000207701)	TNKS (ENSG00000173273)				LV – EF
TTN (ENSG00000155657)					TTN (ENSG00000155657)	RV – SV
	RP11-171I2.3 (ENSG00000271401)	TTN (ENSG00000155657)	TTN (ENSG00000155657)	TTN (ENSG00000155657)		LV – EF
	RP11-171I2.4 (ENSG00000271141)	TTN (ENSG00000155657)	TTN (ENSG00000155657)	TTN (ENSG00000155657)		LV – ESV
	RP11-171I2.1 (ENSG00000267784)	TTN (ENSG00000155657)		TTN (ENSG00000155657)	TTN (ENSG00000155657)	RV – EDV
	RP11-171I2.1 (ENSG00000267784)	TTN (ENSG00000155657)		TTN (ENSG00000155657)	TTN (ENSG00000155657)	RV – EF
	RP11-171I2.1 (ENSG00000267784)	TTN (ENSG00000155657)		TTN (ENSG00000155657)	TTN (ENSG00000155657)	RV – ESV
				TTN (ENSG00000155657)		LV – SV
			CCDC141 (ENSG00000163492)	CCDC141 (ENSG00000163492)		LV – ESV
				CCDC141 (ENSG00000163492)		LV – SV
				CCDC141 (ENSG00000163492)		RV – EDV
				CCDC141 (ENSG00000163492)		RV – ESV
				CCDC141 (ENSG00000163492)		RV – SV
	CCDC141 (ENSG00000163492)	TTN (ENSG00000155657)				LV – EDM
	CCDC141 (ENSG00000163492)	TTN (ENSG00000155657)	CCDC141 (ENSG00000163492)	CCDC141 (ENSG00000163492)		LV – EDV
	RP11-171I2.5 (ENSG00000271011)	TTN (ENSG00000155657)				RV – ESV
	RNU7-104P (ENSG00000238542)	TTN (ENSG00000155657)				RV – EDV

Table S17: Comparing the Schmidt et al. discovered CMR genes to three recent CMR GWAS publications. *(continued)*

Index gene	Nearest gene (Schmidt et al.)	Putative causal gene (Schmidt et al.)	Pirruccello et al. 2020	Pirruccello et al. 2022	Aung et al.	CMR trait
	RNU7-104P (ENSG00000238542)	TTN (ENSG00000155657)				RV – ESV
	RNU7-104P (ENSG00000238542)	TTN (ENSG00000155657)				RV – SV
YWHAE (ENSG00000108953)				YWHAE (ENSG00000108953)		LV – EF
				YWHAE (ENSG00000108953)		LV – ESV
	YWHAE (ENSG00000108953)	YWHAE (ENSG00000108953)				LV – MVR
ZNF572 (ENSG00000180938)	LINC00964 (ENSG00000249816)	ZNF572 (ENSG00000180938)				LV – ESV
	LINC00964 (ENSG00000249816)	ZNF572 (ENSG00000180938)				LV – EF
	LINC00964 (ENSG00000249816)	ZNF572 (ENSG00000180938)				RV – EF
ACLY (ENSG00000131473)				ACLY (ENSG00000131473)		RV – ESV
ADPRHL1 (ENSG00000153531)				ADPRHL1 (ENSG00000153531)		RV – EF
ARHGEF40 (ENSG00000165801)				ARHGEF40 (ENSG00000165801)		LV – SV
ATP5SL (ENSG00000105341)			ATP5SL (ENSG00000105341)			LV – EDV
			ATP5SL (ENSG00000105341)			LV – ESV
				ATP5SL (ENSG00000105341)		RV – ESV
BRAP (ENSG00000089234)				BRAP (ENSG00000089234)		LV – SV
				BRAP (ENSG00000089234)		RV – SV
BCKDHA (ENSG00000248098)				BCKDHA (ENSG00000248098)		RV – EDV
BLK (ENSG00000136573)				BLK (ENSG00000136573)		RV – EF
BMP6 (ENSG00000153162)				BMP6 (ENSG00000153162)		RV – SV
C1orf64 (ENSG00000183888)				C1orf64 (ENSG00000183888)		RV – EF
CCND2 (ENSG00000118971)				CCND2 (ENSG00000118971)		LV – EDV
				CCND2 (ENSG00000118971)		RV – EDV
CENPW (ENSG00000203760)				CENPW (ENSG00000203760)		LV – EDV
				CENPW (ENSG00000203760)		RV – EDV
				CENPW (ENSG00000203760)		RV – ESV
CEP128 (ENSG00000100629)				CEP128 (ENSG00000100629)		RV – EF
				CEP128 (ENSG00000100629)		RV – ESV
CLCNKA (ENSG00000186510)			CLCNKA (ENSG00000186510)			LV – EDV
					CLCNKA (ENSG00000186510)	RV – EF
				CLCNKA (ENSG00000186510)		LV – EF
				CLCNKA (ENSG00000186510)		RV – ESV
DLEU1 (ENSG00000176124)				DLEU1 (ENSG00000176124)		LV – EDV
				DLEU1 (ENSG00000176124)		LV – ESV
				DLEU1 (ENSG00000176124)		RV – EDV
				DLEU1 (ENSG00000176124)		RV – ESV
EPHB1 (ENSG00000154928)				EPHB1 (ENSG00000154928)		LV – EDV
			EPHB1 (ENSG00000154928)	EPHB1 (ENSG00000154928)		LV – ESV
				EPHB1 (ENSG00000154928)		RV – ESV
ERBB4 (ENSG00000178568)				ERBB4 (ENSG00000178568)		LV – ESV
EXOSC5 (ENSG00000077348)				EXOSC5 (ENSG00000077348)		LV – EDV
FBXO46 (ENSG00000177051)				FBXO46 (ENSG00000177051)		RV – ESV
FDFT1 (ENSG00000079459)				FDFT1 (ENSG00000079459)		RV – ESV
GDF5 (ENSG00000125965)			GDF5 (ENSG00000125965)			
				GDF5 (ENSG00000125965)		LV – EDV
				GDF5 (ENSG00000125965)		RV – SV
GJA1 (ENSG00000152661)				GJA1 (ENSG00000152661)		LV – EF
			GJA1 (ENSG00000152661)	GJA1 (ENSG00000152661)		LV – SV
HLA-C (ENSG00000204525)				HLA-C (ENSG00000204525)		RV – SV
HLA-DQA1 (ENSG00000196735)				HLA-DQA1 (ENSG00000196735)		LV – EF
				HLA-DQA1 (ENSG00000196735)		RV – EF
HLA-DQB1 (ENSG00000179344)			HLA-DQB1 (ENSG00000179344)			LV – SV
			HLA-DQB1 (ENSG00000179344)			LV – EF
			HLA-DQB1 (ENSG00000179344)	HLA-DQB1 (ENSG00000179344)		LV – ESV
HMGA1 (ENSG00000137309)				HMGA1 (ENSG00000137309)		RV – EDV
IGFBP3 (ENSG00000146674)				IGFBP3 (ENSG00000146674)		LV – EDV
				IGFBP3 (ENSG00000146674)		LV – ESV
ITCH (ENSG00000078747)				ITCH (ENSG00000078747)		RV – EDV
L3MBTL3 (ENSG00000198945)				L3MBTL3 (ENSG00000198945)		RV – EDV
LSM3 (ENSG00000170860)				LSM3 (ENSG00000170860)		LV – EDV
				LSM3 (ENSG00000170860)		LV – ESV
				LSM3 (ENSG00000170860)		LV – EF
				LSM3 (ENSG00000170860)		RV – EDV
				LSM3 (ENSG00000170860)		RV – ESV
				LSM3 (ENSG00000170860)		RV – EF
MLIP (ENSG00000146147)				MLIP (ENSG00000146147)		LV – EDV
				MLIP (ENSG00000146147)		LV – EF
				MLIP (ENSG00000146147)		RV – EF
MYH7B (ENSG00000078814)				MYH7B (ENSG00000078814)		RV – ESV
PKD1 (ENSG00000008710)			PKD1 (ENSG00000008710)	PKD1 (ENSG00000008710)		LV – EDV
			PKD1 (ENSG00000008710)	PKD1 (ENSG00000008710)		LV – ESV
PLAG1 (ENSG00000181690)				PLAG1 (ENSG00000181690)		LV – ESV

Table S17: Comparing the Schmidt et al. discovered CMR genes to three recent CMR GWAS publications. *(continued)*

Index gene	Nearest gene (Schmidt et al.)	Putative causal gene (Schmidt et al.)	Pirruccello et al. 2020	Pirruccello et al. 2022	Aung et al.	CMR trait
PRKCA (ENSG00000154229)			PRKCA (ENSG00000154229)	PRKCA (ENSG00000154229)		LV – ESV
				PRKCA (ENSG00000154229)		LV – EF
PRLR (ENSG00000113494)				PRLR (ENSG00000113494)		RV – ESV
PXN (ENSG00000089159)			PXN (ENSG00000089159)	PXN (ENSG00000089159)		LV – ESV
				PXN (ENSG00000089159)		LV – EF
RBM20 (ENSG00000203867)			RBM20 (ENSG00000203867)	RBM20 (ENSG00000203867)		LV – ESV
			RBM20 (ENSG00000203867)	RBM20 (ENSG00000203867)		LV – EF
				RBM20 (ENSG00000203867)		LV – EDV
				RBM20 (ENSG00000203867)		RV – ESV
RHOA (ENSG00000067560)				RHOA (ENSG00000067560)		LV – EDV
				RHOA (ENSG00000067560)		LV – ESV
RRAS2 (ENSG00000133818)			RRAS2 (ENSG00000133818)	RRAS2 (ENSG00000133818)		LV – ESV
				RRAS2 (ENSG00000133818)		LV – EF
RSRC1 (ENSG00000174891)				RSRC1 (ENSG00000174891)		RV – ESV
SH2B3 (ENSG00000111252)			SH2B3 (ENSG00000111252)			LV – EDV
			SH2B3 (ENSG00000111252)			LV – ESV
				SH2B3 (ENSG00000111252)		RV – EDV
				SH2B3 (ENSG00000111252)		RV – ESV
SRL (ENSG00000185739)				SRL (ENSG00000185739)		LV – EDV
TIAL1 (ENSG00000151923)				TIAL1 (ENSG00000151923)		LV – EDV
VEGFA (ENSG00000112715)			VEGFA (ENSG00000112715)			LV – SV
			VEGFA (ENSG00000112715)	VEGFA (ENSG00000112715)		LV – EDV
YIPF5 (ENSG00000145817)				YIPF5 (ENSG00000145817)		LV – EF
ZNF638 (ENSG00000075292)			ZNF638 (ENSG00000075292)	ZNF638 (ENSG00000075292)		LV – EDV
ACHE (ENSG00000087085)				ACHE (ENSG00000087085)		LV – EDV
				ACHE (ENSG00000087085)		RV – EDV
ACTBL2 (ENSG00000169067)				ACTBL2 (ENSG00000169067)		LV – ESV
B3GNT7 (ENSG00000156966)			B3GNT7 (ENSG00000156966)	B3GNT7 (ENSG00000156966)		LV – SV
BEND3 (ENSG00000178409)			BEND3 (ENSG00000178409)			LV – EDV
			BEND3 (ENSG00000178409)	BEND3 (ENSG00000178409)		LV – SV
DAG1 (ENSG00000173402)				DAG1 (ENSG00000173402)		LV – EDV
HCN4 (ENSG00000138622)				HCN4 (ENSG00000138622)		LV – SV
HLF (ENSG00000108924)			HLF (ENSG00000108924)			LV – EF
			HLF (ENSG00000108924)	HLF (ENSG00000108924)		LV – ESV
KIAA1462 (ENSG00000165757)				KIAA1462 (ENSG00000165757)		RV – EDV
MTSS1 (ENSG00000170873)			MTSS1 (ENSG00000170873)			LV – EDV
			MTSS1 (ENSG00000170873)	MTSS1 (ENSG00000170873)		LV – ESV
MYH6 (ENSG00000197616)			MYH6 (ENSG00000197616)	MYH6 (ENSG00000197616)		LV – SV
OR9Q1 (ENSG00000186509)				OR9Q1 (ENSG00000186509)		RV – EDV
				OR9Q1 (ENSG00000186509)		RV – SV
PALLD (ENSG00000129116)				PALLD (ENSG00000129116)	PALLD (ENSG00000129116)	RV – ESV
PIK3CG (ENSG00000105851)				PIK3CG (ENSG00000105851)		LV – SV
RBL2 (ENSG00000103479)				RBL2 (ENSG00000103479)		LV – SV
					RBL2 (ENSG00000103479)	RV – EDV
					RBL2 (ENSG00000103479)	RV – ESV
SPATS2L (ENSG00000196141)			SPATS2L (ENSG00000196141)			LV – ESV
			SPATS2L (ENSG00000196141)	SPATS2L (ENSG00000196141)		LV – EDV
SURF6 (ENSG00000148296)			SURF6 (ENSG00000148296)			LV – EDV
			SURF6 (ENSG00000148296)	SURF6 (ENSG00000148296)		LV – SV
AK097794 (ENSG00000243150)					AK097794 (ENSG00000243150)	RV – ESV
					AK097794 (ENSG00000243150)	RV – EF
					AK097794 (ENSG00000243150)	RV – EDV
ACTN4 (ENSG00000130402)					ACTN4 (ENSG00000130402)	RV – EDV
					ACTN4 (ENSG00000130402)	RV – ESV
AK311445 (ENSG00000226334)					AK311445 (ENSG00000226334)	RV – EF
BC038750 (ENSG00000250772)					BC038750 (ENSG00000250772)	RV – EDV
CAMK2D (ENSG00000145349)					CAMK2D (ENSG00000145349)	RV – ESV
CCDC85C (ENSG00000205476)					CCDC85C (ENSG00000205476)	RV – EF
HSPA4 (ENSG00000170606)					HSPA4 (ENSG00000170606)	RV – EF
LUC7L2 (ENSG00000269955)					LUC7L2 (ENSG00000269955)	RV – EDV
					LUC7L2 (ENSG00000269955)	RV – SV
SLC6A6 (ENSG00000131389)					SLC6A6 (ENSG00000131389)	RV – EDV
					SLC6A6 (ENSG00000131389)	RV – ESV
					SLC6A6 (ENSG00000131389)	RV – EF
SVIL (ENSG00000197321)					SVIL (ENSG00000197321)	RV – EF
TPM2 (ENSG00000198467)					TPM2 (ENSG00000198467)	RV – EF
ACTN2 (ENSG00000077522)			ACTN2 (ENSG00000077522)			LV – EF
AGO2 (ENSG00000123908)			AGO2 (ENSG00000123908)			LV – ESV
AKR1A1 (ENSG00000117448)			AKR1A1 (ENSG00000117448)			LV – EDV
			AKR1A1 (ENSG00000117448)			LV – ESV
CSRP3 (ENSG00000129170)			CSRP3 (ENSG00000129170)			LV – EF

Table S17: Comparing the Schmidt et al. discovered CMR genes to three recent CMR GWAS publications. *(continued)*

Index gene	Nearest gene (Schmidt et al.)	Putative causal gene (Schmidt et al.)	Pirruccello et al. 2020	Pirruccello et al. 2022	Aung et al.	CMR trait
			CSRP3 (ENSG00000129170)			LV – ESV
DEFB136 (ENSG00000205884)			DEFB136 (ENSG00000205884)			LV – EF
FNDC3B (ENSG00000075420)			FNDC3B (ENSG00000075420)			LV – ESV
HAND2 (ENSG00000164107)			HAND2 (ENSG00000164107)			LV – EDV
HECTD4 (ENSG00000173064)			HECTD4 (ENSG00000173064)			LV – EDV
			HECTD4 (ENSG00000173064)			LV – ESV
			HECTD4 (ENSG00000173064)			LV – SV
HLA-DQA2 (ENSG00000237541)			HLA-DQA2 (ENSG00000237541)			LV – EDV
ILF3 (ENSG00000129351)			ILF3 (ENSG00000129351)			LV – ESV
LLPH (ENSG00000139233)			LLPH (ENSG00000139233)			LV – EDV
			LLPH (ENSG00000139233)			LV – SV
MAPT (ENSG00000186868)			MAPT (ENSG00000186868)			LV – ESV
MECOM (ENSG00000085276)			MECOM (ENSG00000085276)			LV – EDV
MITF (ENSG00000187098)			MITF (ENSG00000187098)			LV – EF
			MITF (ENSG00000187098)			LV – ESV
NKX2-5 (ENSG00000183072)			NKX2-5 (ENSG00000183072)			LV – SV
PTK2 (ENSG00000169398)			PTK2 (ENSG00000169398)			LV – EF
RNF207 (ENSG00000158286)			RNF207 (ENSG00000158286)			LV – ESV
RPH3A (ENSG00000089169)			RPH3A (ENSG00000089169)			LV – SV
RPL22 (ENSG00000116251)			RPL22 (ENSG00000116251)			LV – ESV
SESTD1 (ENSG00000187231)			SESTD1 (ENSG00000187231)			LV – ESV
SP3 (ENSG00000172845)			SP3 (ENSG00000172845)			LV – EDV
SPEN (ENSG00000065526)			SPEN (ENSG00000065526)			LV – EDV
SSPN (ENSG00000123096)			SSPN (ENSG00000123096)			LV – EF
			SSPN (ENSG00000123096)			LV – ESV
XPC (ENSG00000154767)			XPC (ENSG00000154767)			LV – EDV
			XPC (ENSG00000154767)			LV – EF
			XPC (ENSG00000154767)			LV – ESV
ZNF592 (ENSG00000166716)			ZNF592 (ENSG00000166716)			LV – EDV
			ZNF592 (ENSG00000166716)			LV – ESV
			ZNF592 (ENSG00000166716)			LV – EF

General:
For Schmidt et al. both the nearest gene as well as the putative causal gene were recorded and matched to any gene from Pirruccello et al. or Aung et al. [34-36]. The associated cardiac MRI (CMR) trait was recorded in the *CMR trait* column. Pirruccello et al. [34] analyzed MRI traits not considered by Schmidt et al., which were excluded from the current comparison. The *Index gene column* is used to group entries, with its label either equal to the putative causal gene column, or if this was empty, based on the Pirruccello or Aung assigned gene.

**Unique CMR genes**
ALDH2 (ENSG00000111275)
CMSS1 (ENSG00000184220)
DES (ENSG00000175084)
HIST3H3 (ENSG00000168148)
HLA-DRB1 (ENSG00000196126)
KANSL1 (ENSG00000120071)
LRLE1 (ENSG00000268955)
NSF (ENSG00000073969)
PPP1R1B (ENSG00000131771)
PROM1 (ENSG00000007062)
RP11-457K10.1 (ENSG00000242029)
SCN10A (ENSG00000185313)
SKI (ENSG00000157933)
SYNPO2L (ENSG00000166317)
TBX5 (ENSG00000089225)
TGFBR3 (ENSG00000069702)
TGM2 (ENSG00000198959)
ZNF572 (ENSG00000180938)

General:
The putative genes were assigned based on the criteria described in Supplementary File 2, and were not discovered by Pirruccello et al. or Aung et al. [34-36], irrespective of the LV/RV trait the variants were associated with.

Table S18: Unique putative CMR genes, discovered by Schmidt et al.

Supplementary methods

Cardiac MRI quality control steps

We had access to 39584 LV and RV CMR scans, derived using a published deep learning algorithm by Ruijsink et al. [7]; Supplementary Figures S1-S2. The first step of the automated quality control assessed image quality. Here an AI algorithm interrogated images on significant artefacts, arrhythmia artefacts, and wrong planning. This was followed by quality control of the output data, identifying improbable values (i.e. SV difference between LV and RV > 25%, unphysiological volume curves).

After passing the automated CMR quality control step 34167 LV and 37827 RV measurements remained. This set of measurements was further curated by excluding subjects with an negative LV or RV CMR measurements, duplicate subjects, or subjects without available genetic data. Furthermore, to minimize influence of pre-existing conditions on LV or RV CMR measurements we excluded subjects with prevalent (cardiac) diseases including pre-existing heart failure (LV-EF $\leq$ 40%); Supplementary Table S2. Lastly, potential outliers where removed by excluding measurements below or above 3 times the interquartile range (Supplementary Table S19).

Table S19: The lower and upper limits beyond which a cardiac MRI measurement was excluded from subsequent analysis.

	Lower limit	Upper limit
RV-EDV	-34.1	337.3
RV-ESV	-39.5	166.5
RV-SV	-9.3	185.0
RV-EF	28.5	88.0
RV-PER	-120.4	904.6
RV-PFR	-117.2	723.4
RV-PAFR	-175.9	756.6
LV-EDV	-16.9	298.7
LV-ESV	-27.5	142.5

LV-SV	-4.6	171.2
LV-EF	31.0	87.8
LV-EDM	-33.0	192.3
LV-PER	-155.3	904.0
LV-PFR	-134.1	768.9
LV-PAFR	263.5	754.6

Supplementary results

Through consensus-based prioritization leveraging 20+ criteria (see Methods in the main text and the Supplementary Locus-view plots) lead variants were mapped to putative causal genes, including 13 cases where leads variants (passing the conservative p-value threshold) in or around these genes affected multiple CMR traits: *TTN, TMEM43, PROB1, DMPK, PLEC, HLA-B, CDKN1A, ALPK3, SPON1, KCNK3, KCNH2, IGF1R, and CMSS1*.

Comparing the CMR GWAS results to three recent publications

Three recent GWAS by Pirruccello *et al.* and Aung *et al.* [34-36] conducted similar analyses using the UK biobank. To compare our results we first excluded any genetic associations with traits that were not considered in our current publication. For example, Aung *et al.* [35] extracted data on atrial measurements not considered by Schmidt *et al.*. Next, we extract data on the assigned gene identified by each study, additionally recording the CMR traits. We subsequently compared these findings to the nearest gene and the putative causal gene discovered in the current manuscript. Given that many of the CMR traits are closely related, genes were compared irrespective of the type of CMR trait the genetic variants associated with.

Compared to these previous CMR GWAS, we rediscovered 36 putative CMR genes, as well as 18 genes which were unique to the current study (Tables S17-S18). The majority of the putative CMR genes could be linked to cardio-metabolic traits (Figure S4) and included 8 genes which encoded a drugged protein with known

cardio-metabolic indications or side effects.

Broader phenotypic effects of CMR variants

Extracting data from GWAS catalogue (Supplementary Figures S4-S5), we found that our putative CMR genes have been previously implicated with CMR traits from previous GWAS (e.g., LV dilatation, LV mass, and fractal dimension), with electrocardiographic traits (e.g., PR segmentation, QRS duration, QT interval), blood pressure and heart rate; as well as with plasma concentration of various apolipoproteins and cholesterol-containing lipoproteins. The following CMR genes were previously associated with cardiac diseases including: AF (*SYNPO2L, TBX5, IGF1R, GOSR2, TTN, SCN10A, CDKN1A, MYO18B, KCNH2*) hypertrophic cardiomyopathy (*HSPB7, SYNPO2L, BAG3, NSF, FHOD3, CDKN1A, SMARCB1*), DCM (*BAG3, FHOD3, TTN, SMARCB1*), HF (*SYNPO2L, BAG3*); and CHD (*ATXN2, ALDH2, PTPN11, GOSR2*); Supplementary Figure S5.

Additionally, 18 of these putative CMR genes were encoded by a drugged or druggable protein, including compounds with an indication and/or side-effect for AF, HF, and CHD; Supplementary Tables S5-S8.

Genetic heritability of CMR traits and pairwise genetic correlation

BOLT-REML was used to estimate the amount of phenotypic variation that could be explained by narrow-sense genetic heritability (Supplementary Figure S4). Heritability estimates ranged between 36% and 31% for both RV and LV measurements of EDV and ESV, as well as LV-EDM. For LV-MVR, EF and SV of both ventricles heritability ranged between 20% and 29%. Despite an absence of GWAS hits for PFR, LV-PER and LV-PAFR, heritability of these traits was between 6% and 12%.

The pairwise genetic correlation (Supplementary Figure S6) indicated that genetic variants for SV and PER measurements (both LV and RV) were highly correlated (correlation coefficient close to 1.0), as were genetic variants associated with EDV and ESV traits from both ventricles, and variants for LV-PFR and RV-PFR. LV-EDM had a moderately strong correlation (around 0.70) with SV, PER, ESV, EDV of both ventricles. Finally, variants for LV-MVR, RV-EF, and LV-EF showed a positive correlation among themselves (maximum 0.68), and negative correlation with EDV, ESV, EDM, and SV traits (maximum -0.86).

While the number of discovered variants, putative genes, and genetic heritability differed considerably across CMR traits (16 putative

genes for RV-ESV, compared to zero for RV-PFR, LV-PER, and LV-PAFR), the genetic contribution was balanced across both ventricles, and variants for LV and RV measurements were often highly correlated, suggesting similar genetic burden between LV and RV traits. Principal component analysis of the CMR measurement themselves further found that 7 PCs explain more than 90% of the phenotypic variation, where typically LV and RV of a specific trait contributed to the same PC (Figure S3).

GWAS analysis without BSA and SBP adjustment

The presented GWAS analysis on CMR traits was repeated without covariate adjustment for body surface area (BSA) and systolic blood pressure (SBP). Potentially these variables might act as intermediates positioned between the genetic variant and the considered CMR traits, which would induced selection bias (i.e., collider-stratification bias).

To empirically evaluate the potential influence of BSA and SBP correction we extracted the mean difference from the original GWAS analysis (with BSA+SBP adjustment) and compared these to an analysis without adjustment for these covariates, finding near perfect correlation; Supplementary Figure S7.

Locus-view plots

Locus view plots for all the lead variants from a GWAS of 16 CMR traits. The scatter plot is a zoomed in view of the associations in the chromosomal region around each respective lead variant (lead variant position +/- 250 kbp flanking region). The points are coloured according to each variant's pairwise r-squared (r^2) with the lead variant. The track below the scatter plot shows the genes overlapping each region with the < or > next to the gene name indicating the strand for the gene. The exon structure for the canonical transcript each gene is shown with vertical bars. The grid at the bottom of each plot is an incidence matrix of annotations where a red dot indicates that a gene has the respective annotation listed on the y-axis. The annotations are as follows:

- *nearest gene* - Is the nearest canonical transcript to the lead variant. Canonical transcripts are defined by Ensembl (release 105). nearest canonical transcript is calculated as the nearest point of the transcript (either start or end) to the lead variant. Where the transcript overlaps the lead variant it is deemed the closest. Where > 1 transcript overlaps the lead variant the tie is broken by the biggest overlap. Note that in some instances the nearest gene and the nearest transcript differ, this is because gene boundaries are based on a union of all transcripts and not just the canonical one.
- *cardiac annot.* - do any of the genes have a cardiac related annotation in the GeneCards database.
- *cardio ind.* - are any of the genes encoding targets for licensed or developmental drugs indicated for a cardiovascular/cardio-metabolic disease.
- *cardio se.* - do any of the genes encoding targets for licensed drugs have a cardiovascular/cardio-metabolic side effect.
- *sig. MR effect* - do any of the genes have a significant *cis*-MR association ($-\log_{10} P > 5.35$) with the respective CMR trait.
- *any sig. MR effect* - do any of the genes in the region have a significant *cis*-MR association with any CMR trait (not necessarily the one in the locus view).

- *sug. MR effect* - do any of the genes in the region have a suggestive *cis*-MR association ($1.3 < -\log_{10} P \leq 5.35$) with the respective CMR trait.
- *any sug. MR effect* - do any of the genes in the region have a suggestive cis-MR association with any CMR trait.
- *cardiovascular GWAS* - have any of the genes been listed in the GWAS catalog 'SNP_GENE_IDS' column for cardiovascular disease.
- *cardiac biomarker GWAS* - have any of the genes been listed in the GWAS catalog 'SNP_GENE_IDS' column for a cardiac biomarker.
- *lipid biomarker GWAS* - have any of the genes been listed in the GWAS catalog 'SNP_GENE_IDS' column for a lipid biomarker.
- *blood pressure GWAS* - have any of the genes been listed in the GWAS catalog 'SNP_GENE_IDS' column for a blood pressure measure.
- *diabetes GWAS* - have any of the genes been listed in the GWAS catalog 'SNP_GENE_IDS' column for a diabetes trait.
- *heart e/sQTLs* - Has the lead variant been identified as a expression (e) or splice (s) quantitative trait loci for any genes GTEX heart tissues.
- *vascular e/sQTLs* - Has the lead variant been identified as an e/sQTL for any genes GTEX artery tissues.
- *any pQTLs* - Has the lead variant been identified as a protein QTL in the plasma for any genes in the region.
- *trans loci link* - Do any of the genes in the loci encode proteins that have trans pQTL associations that occur in other loci (not necessarily for the same CMR phenotype).
- *P-P loci direct link* - Do any of the proteins encoded by genes in the loci have a direct protein-protein interaction with any proteins encoded by genes that overlap any other CMR loci (not necessarily for the same CMR phenotype)
- *P-P loci common link* - Do any of the proteins encoded by genes in the loci have a direct protein-protein interaction with a common protein that is also interacting with proteins encoded by genes that overlap any other CMR loci (not necessarily for the same CMR phenotype)
- *P-P direct cardio drug target ind.* - Do any of the proteins encoded by genes in the loci have a direct protein-protein interaction with a protein targeted by drugs with a cardio-metabolic indication
- *P-P link sig. MR effect* - Do any of the proteins encoded by genes in the loci have a direct protein-protein interaction with a protein that has a significant MR association with a CMR phenotype (not necessarily for the same CMR phenotype)
- *is druggable* - are any of the genes annotated as potentially

druggable or the targets for existing/developmental compounds.

- *is coding* - Is the gene protein coding.

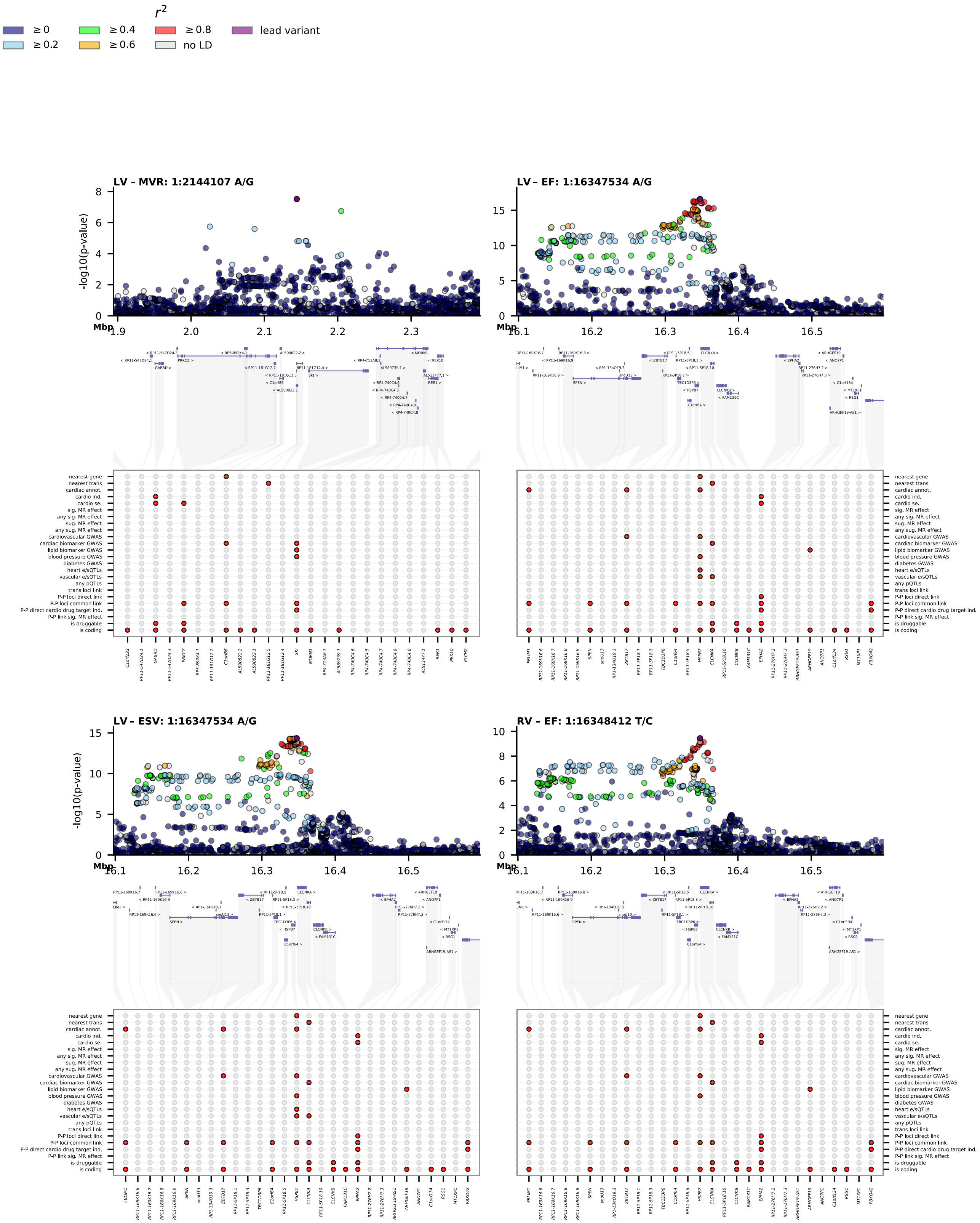

r^2
≥ 0
≥ 0.2
≥ 0.4
≥ 0.6
≥ 0.8
no LD
lead variant
LV - MVR: 1:2144107 A/G
LV – EF: 1:16347534 A/G
LV – ESV: 1:16347534 A/G
RV – EF: 1:16348412 T/C
-log10(p-value)
Mbp
nearest gene
nearest trans
cardiac annot.
cardio ind.
cardio se.
sig. MR effect
any sig. MR effect
sug. MR effect
any sug. MR effect
cardiovascular GWAS
cardiac biomarker GWAS
lipid biomarker GWAS
blood pressure GWAS
diabetes GWAS
heart e/sQTLs
vascular e/sQTLs
any pQTLs
trans loci link
P-P loci direct link
P-P loci common link
P-P direct cardio drug target ind.
P-P link sig. MR effect
is druggable
is coding

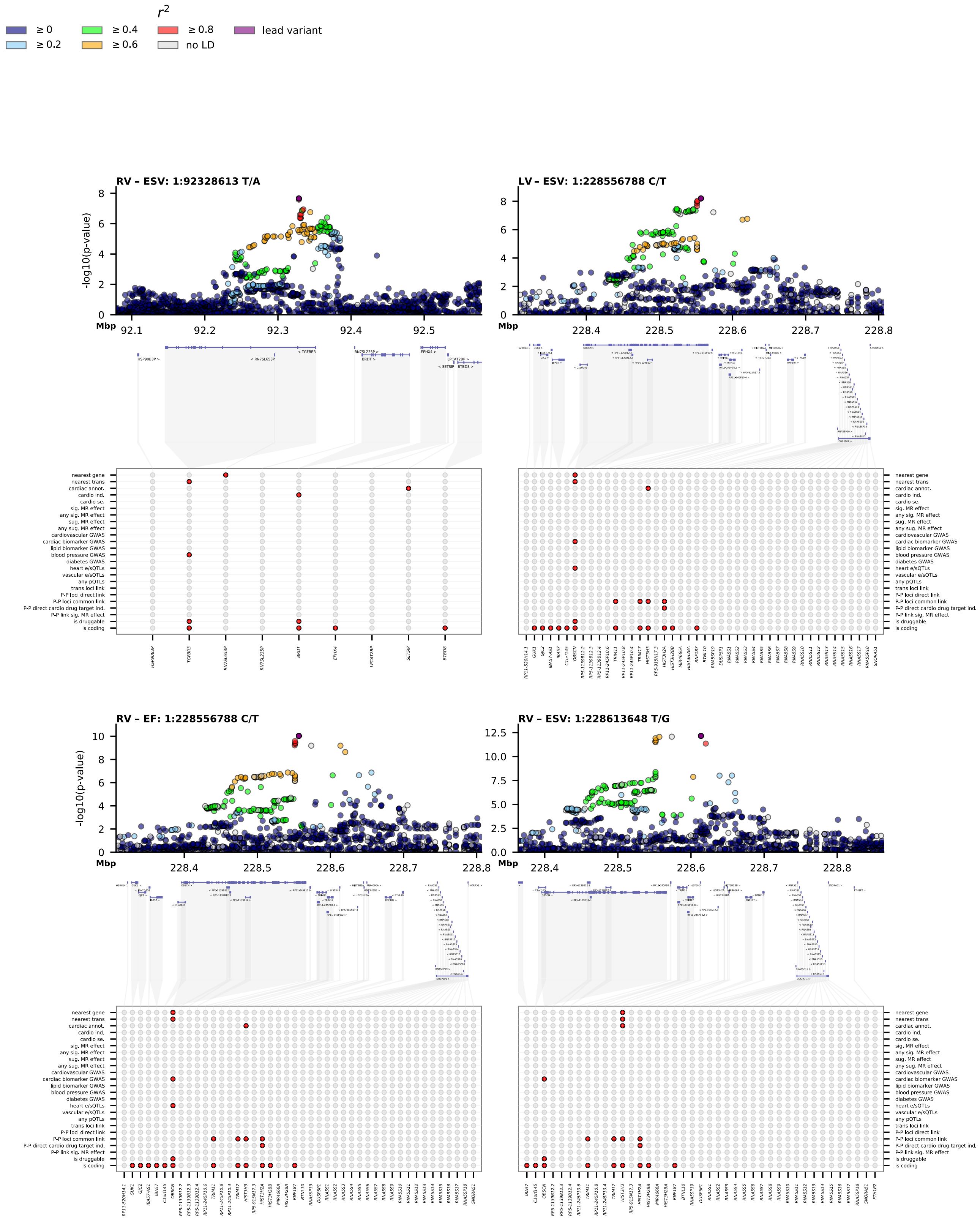

r^2
≥ 0
≥ 0.2
≥ 0.4
≥ 0.6
≥ 0.8
no LD
lead variant
RV – ESV: 1:92328613 T/A
LV – ESV: 1:228556788 C/T
RV – EF: 1:228556788 C/T
RV – ESV: 1:228613648 T/G
-log10(p-value)
Mbp
nearest gene
nearest trans
cardiac annot.
cardio ind.
cardio se.
sig. MR effect
any sig. MR effect
sug. MR effect
any sug. MR effect
cardiovascular GWAS
cardiac biomarker GWAS
lipid biomarker GWAS
blood pressure GWAS
diabetes GWAS
heart e/sQTLs
vascular e/sQTLs
any pQTLs
trans loci link
P-P loci direct link
P-P loci common link
P-P direct cardio drug target ind.
P-P link sig. MR effect
is druggable
is coding

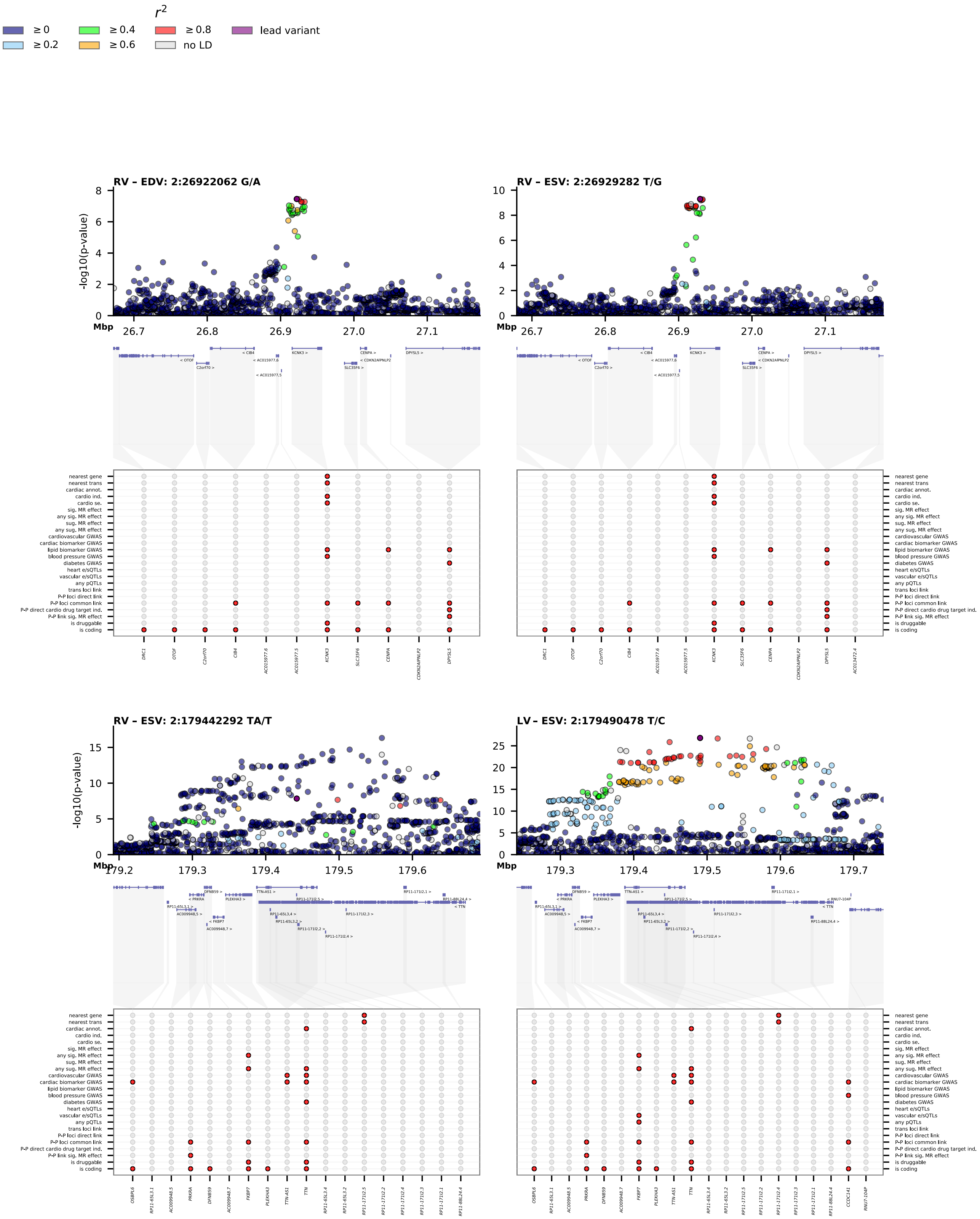

r^2
≥ 0
≥ 0.2
≥ 0.4
≥ 0.6
≥ 0.8
no LD
lead variant
RV – EDV: 2:26922062 G/A
RV – ESV: 2:26929282 T/G
RV – ESV: 2:179442292 TA/T
LV – ESV: 2:179490478 T/C
-log10(p-value)
Mbp
nearest gene
nearest trans
cardiac annot.
cardio ind.
cardio se.
sig. MR effect
any sig. MR effect
sug. MR effect
any sug. MR effect
cardiovascular GWAS
cardiac biomarker GWAS
lipid biomarker GWAS
blood pressure GWAS
diabetes GWAS
heart e/sQTLs
vascular e/sQTLs
any pQTLs
trans loci link
P-P loci direct link
P-P loci common link
P-P direct cardio drug target ind.
P-P link sig. MR effect
is druggable
is coding

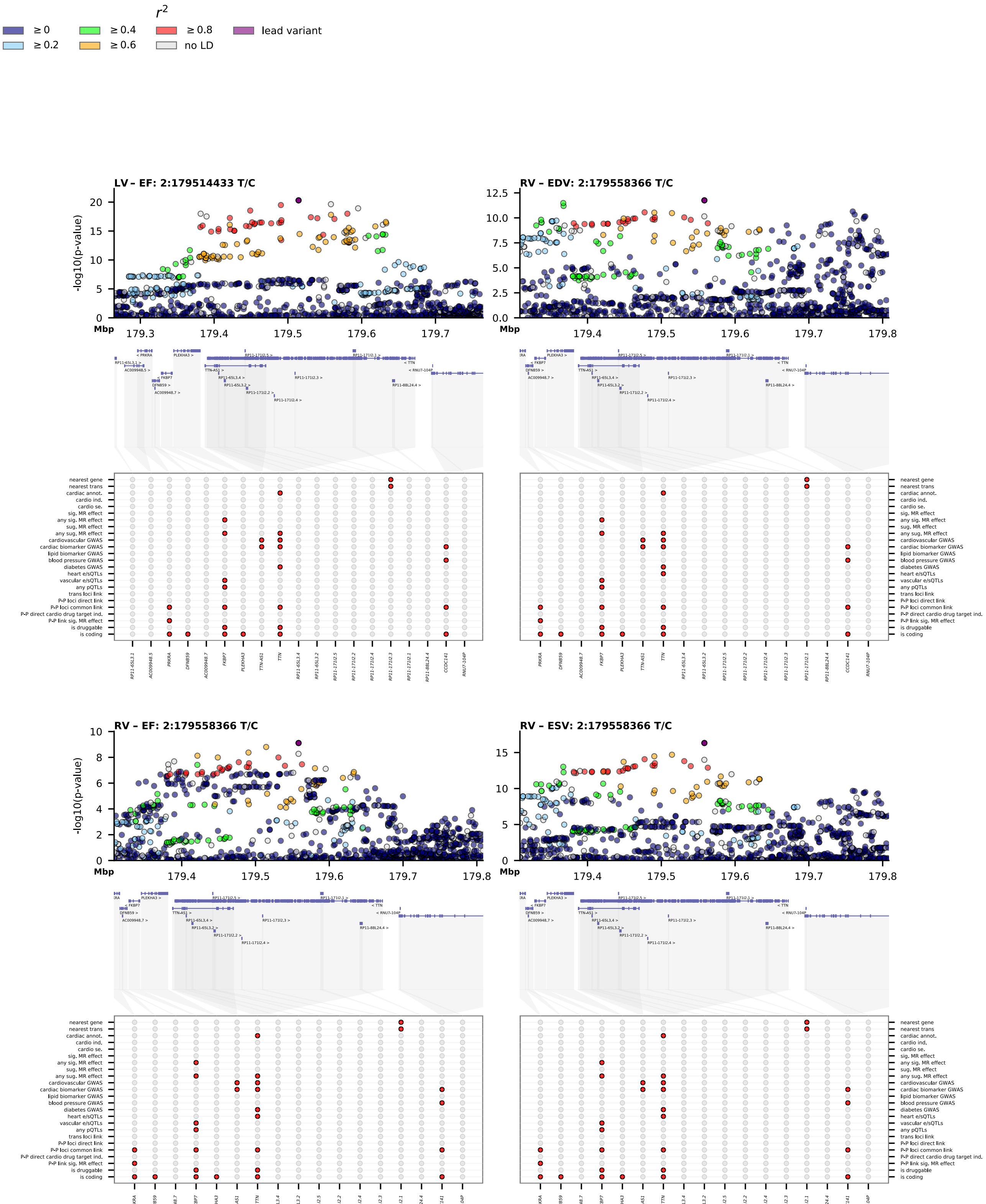

r^2
≥ 0
≥ 0.2
≥ 0.4
≥ 0.6
≥ 0.8
no LD
lead variant
LV – EF: 2:179514433 T/C
RV – EDV: 2:179558366 T/C
RV – EF: 2:179558366 T/C
RV – ESV: 2:179558366 T/C
-log10(p-value)
Mbp
nearest gene
nearest trans
cardiac annot.
cardio ind.
cardio se.
sig. MR effect
any sig. MR effect
sug. MR effect
any sug. MR effect
cardiovascular GWAS
cardiac biomarker GWAS
lipid biomarker GWAS
blood pressure GWAS
diabetes GWAS
heart e/sQTLs
vascular e/sQTLs
any pQTLs
trans loci link
P-P loci direct link
P-P loci common link
P-P direct cardio drug target ind.
P-P link sig. MR effect
is druggable
is coding
PRKRA
DFNB59
AC009948.7
FKBP7
PLEKHA3
TTN-AS1
TTN
CCDC141

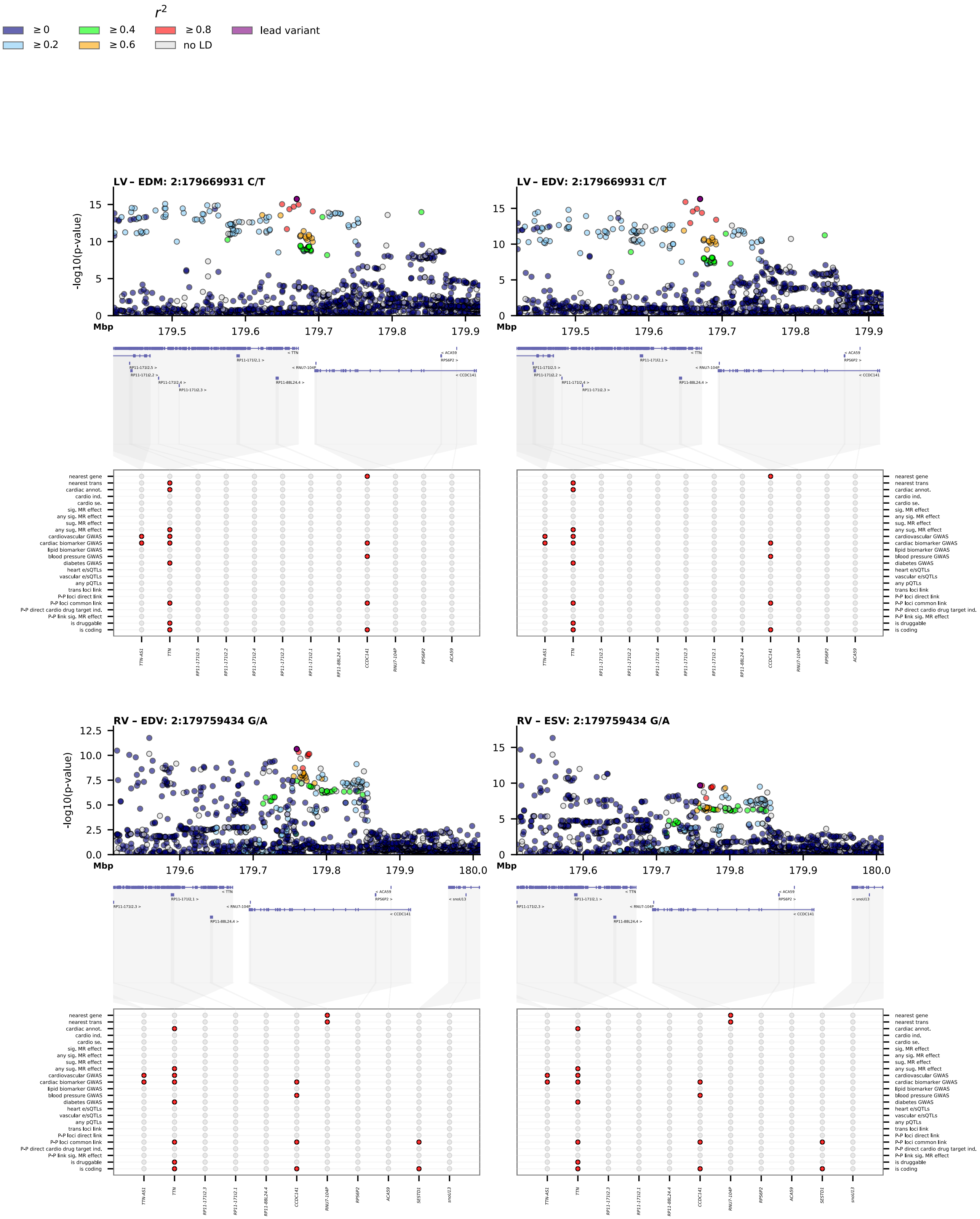

r^2
≥ 0
≥ 0.2
≥ 0.4
≥ 0.6
≥ 0.8
no LD
lead variant
LV – EDM: 2:179669931 C/T
LV – EDV: 2:179669931 C/T
RV – EDV: 2:179759434 G/A
RV – ESV: 2:179759434 G/A
-log10(p-value)
Mbp
nearest gene
nearest trans
cardiac annot.
cardio ind.
cardio se.
sig. MR effect
any sig. MR effect
sug. MR effect
any sug. MR effect
cardiovascular GWAS
cardiac biomarker GWAS
lipid biomarker GWAS
blood pressure GWAS
diabetes GWAS
heart e/sQTLs
vascular e/sQTLs
any pQTLs
trans loci link
P-P loci direct link
P-P loci common link
P-P direct cardio drug target ind.
P-P link sig. MR effect
is druggable
is coding
TTN-AS1
TTN
RP11-171I2.5
RP11-171I2.2
RP11-171I2.4
RP11-171I2.3
RP11-171I2.1
RP11-88L24.4
CCDC141
RNU7-104P
RPS6P2
ACA59
SESTD1
snoU13

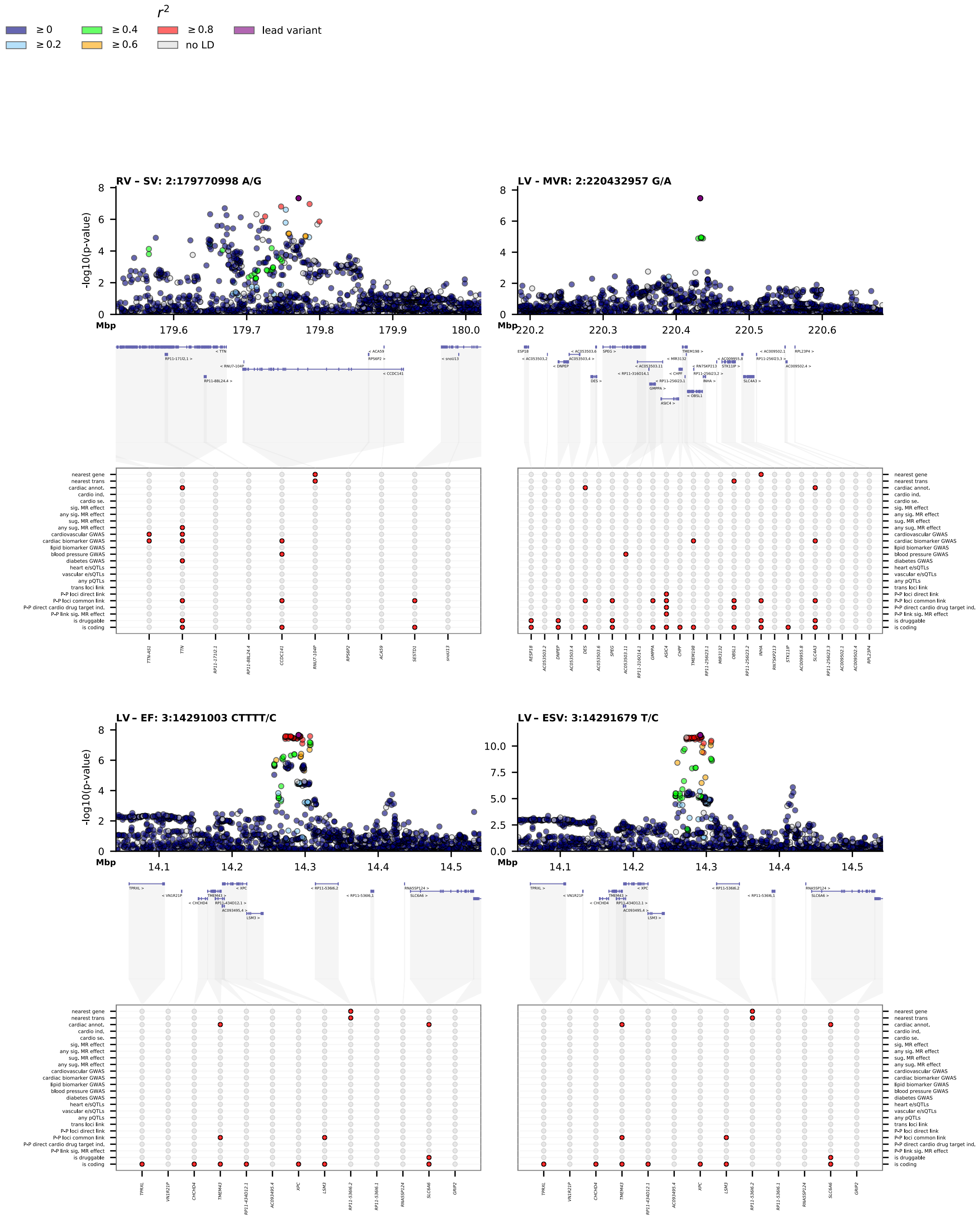

r^2
≥ 0
≥ 0.2
≥ 0.4
≥ 0.6
≥ 0.8
no LD
lead variant
RV – SV: 2:179770998 A/G
LV - MVR: 2:220432957 G/A
LV – EF: 3:14291003 CTTTT/C
LV – ESV: 3:14291679 T/C
-log10(p-value)
Mbp
nearest gene
nearest trans
cardiac annot.
cardio ind.
cardio se.
sig. MR effect
any sig. MR effect
sug. MR effect
any sug. MR effect
cardiovascular GWAS
cardiac biomarker GWAS
lipid biomarker GWAS
blood pressure GWAS
diabetes GWAS
heart e/sQTLs
vascular e/sQTLs
any pQTLs
trans loci link
P-P loci direct link
P-P loci common link
P-P direct cardio drug target ind.
P-P link sig. MR effect
is druggable
is coding

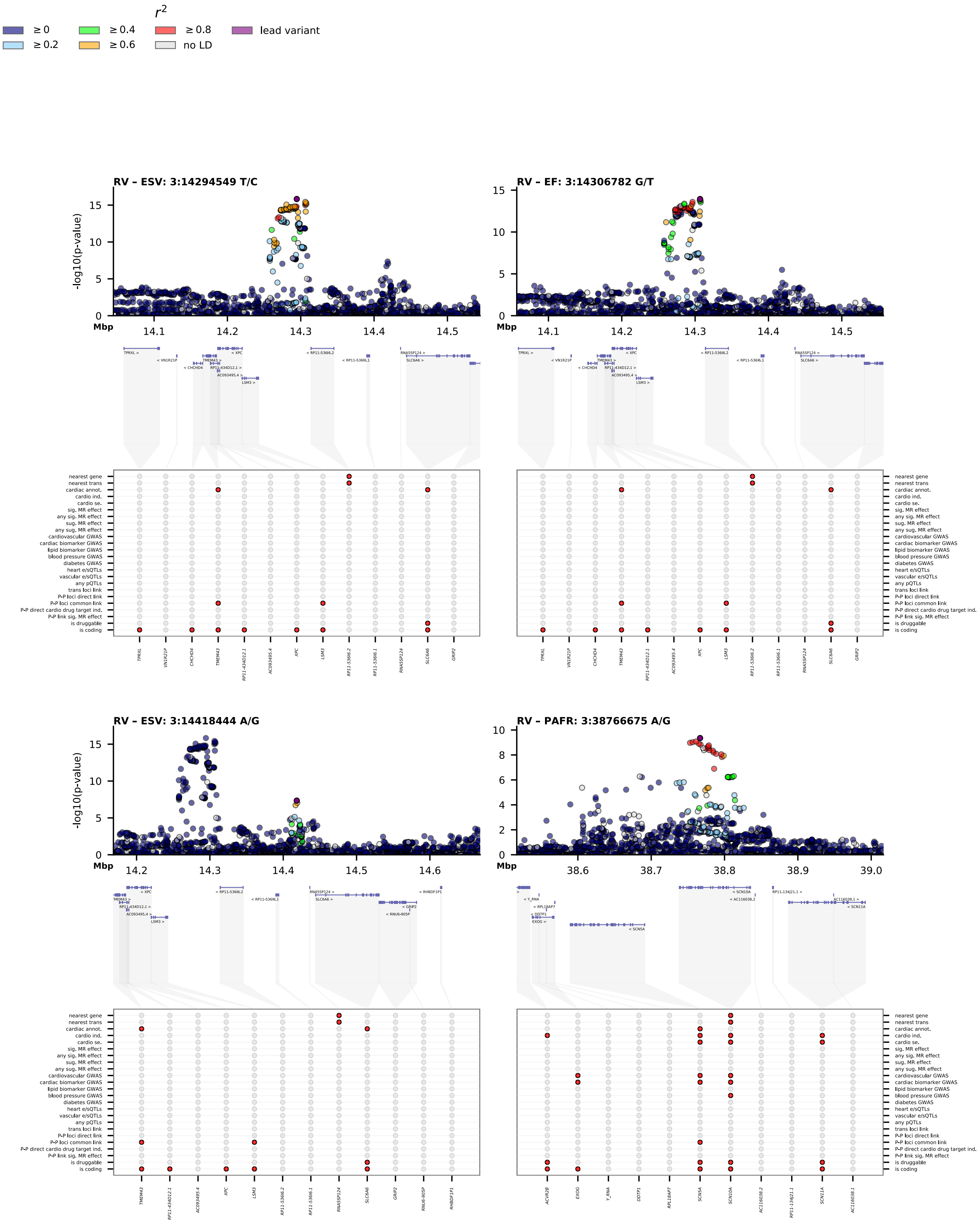

r^2
≥ 0
≥ 0.2
≥ 0.4
≥ 0.6
≥ 0.8
no LD
lead variant
RV – ESV: 3:14294549 T/C
RV – EF: 3:14306782 G/T
RV – ESV: 3:14418444 A/G
RV – PAFR: 3:38766675 A/G
-log10(p-value)
Mbp
nearest gene
nearest trans
cardiac annot.
cardio ind.
cardio se.
sig. MR effect
any sig. MR effect
sug. MR effect
any sug. MR effect
cardiovascular GWAS
cardiac biomarker GWAS
lipid biomarker GWAS
blood pressure GWAS
diabetes GWAS
heart e/sQTLs
vascular e/sQTLs
any pQTLs
trans loci link
P-P loci direct link
P-P loci common link
P-P direct cardio drug target ind.
P-P link sig. MR effect
is druggable
is coding

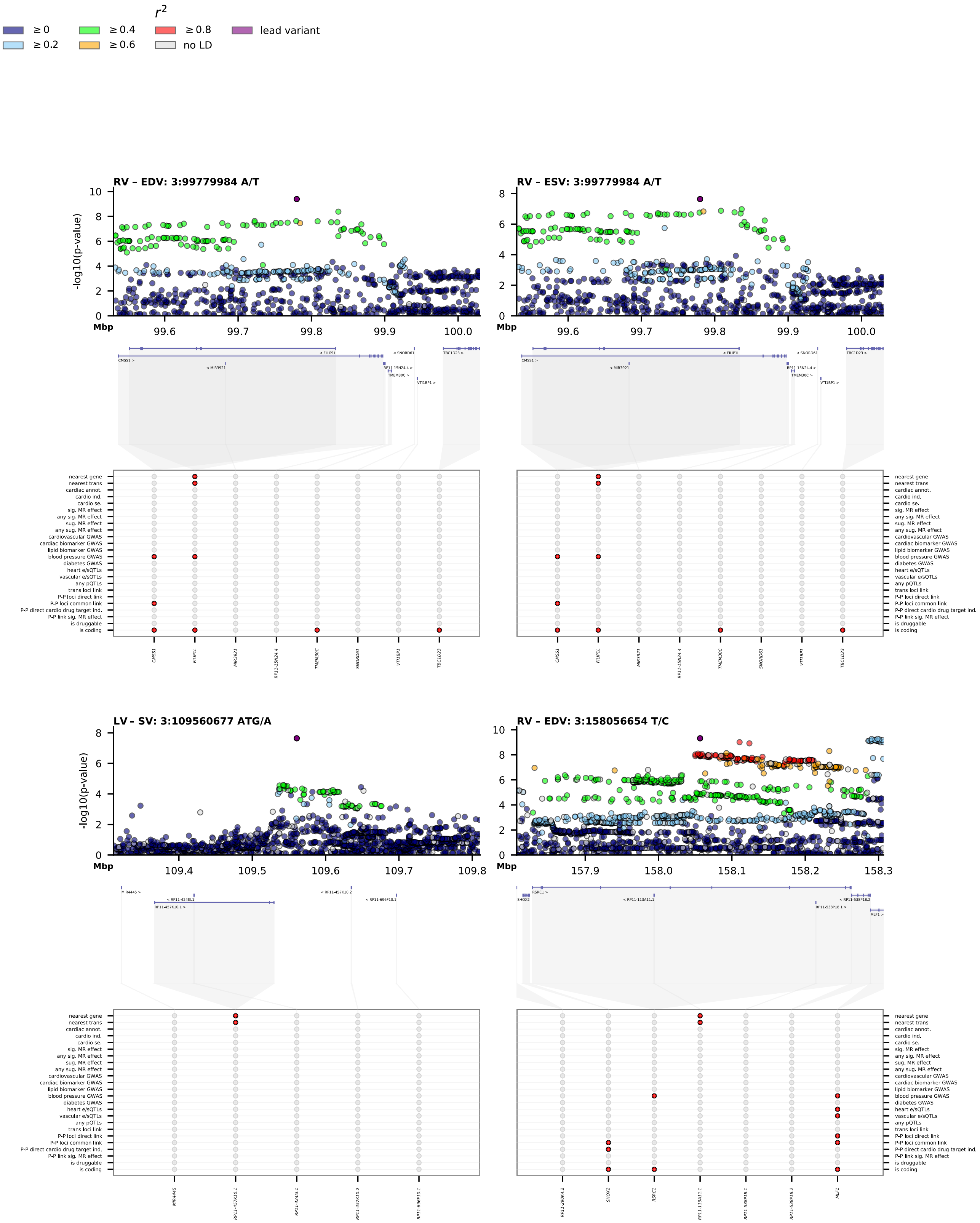

r^2
≥ 0
≥ 0.2
≥ 0.4
≥ 0.6
≥ 0.8
no LD
lead variant
RV – EDV: 3:99779984 A/T
RV – ESV: 3:99779984 A/T
LV – SV: 3:109560677 ATG/A
RV – EDV: 3:158056654 T/C
-log10(p-value)
Mbp
nearest gene
nearest trans
cardiac annot.
cardio ind.
cardio se.
sig. MR effect
any sig. MR effect
sug. MR effect
any sug. MR effect
cardiovascular GWAS
cardiac biomarker GWAS
lipid biomarker GWAS
blood pressure GWAS
diabetes GWAS
heart e/sQTLs
vascular e/sQTLs
any pQTLs
trans loci link
P-P loci direct link
P-P loci common link
P-P direct cardio drug target ind.
P-P link sig. MR effect
is druggable
is coding
CMSS1
FILIP1L
MIR3921
RP11-15N24.4
TMEM30C
SNORD61
VTI1BP1
TBC1D23
MIR4445
RP11-457K10.1
RP11-424I3.1
RP11-457K10.2
RP11-696F10.1
RP11-290K4.2
SHOX2
RSRC1
RP11-113A11.1
RP11-538P18.1
RP11-538P18.2
MLF1

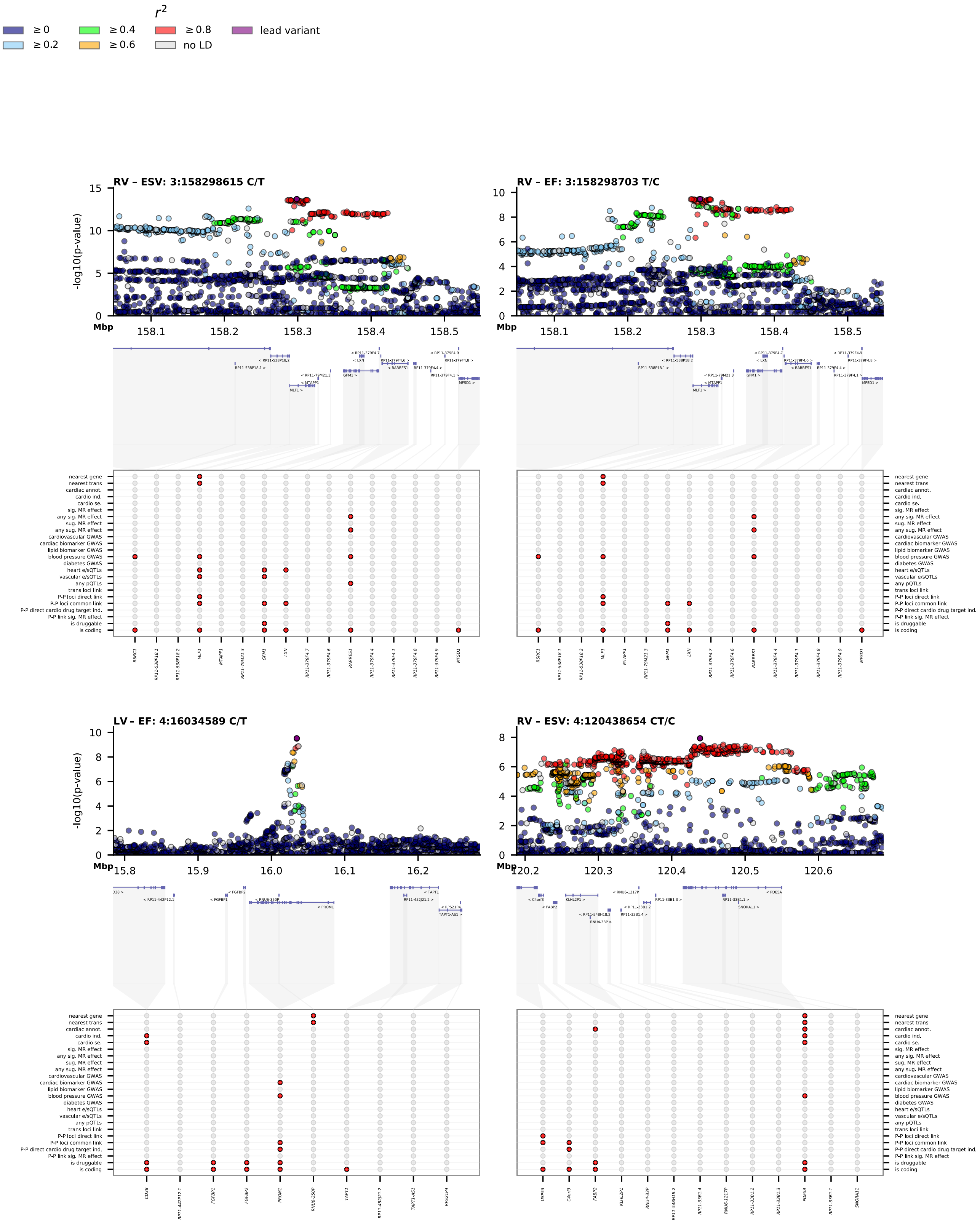
r^2
≥ 0
≥ 0.2
≥ 0.4
≥ 0.6
≥ 0.8
no LD
lead variant
RV – ESV: 3:158298615 C/T
RV – EF: 3:158298703 T/C
LV – EF: 4:16034589 C/T
RV – ESV: 4:120438654 CT/C
-log10(p-value)
Mbp
nearest gene
nearest trans
cardiac annot.
cardio ind.
cardio se.
sig. MR effect
any sig. MR effect
sug. MR effect
any sug. MR effect
cardiovascular GWAS
cardiac biomarker GWAS
lipid biomarker GWAS
blood pressure GWAS
diabetes GWAS
heart e/sQTLs
vascular e/sQTLs
any pQTLs
trans loci link
P-P loci direct link
P-P loci common link
P-P direct cardio drug target ind.
P-P link sig. MR effect
is druggable
is coding

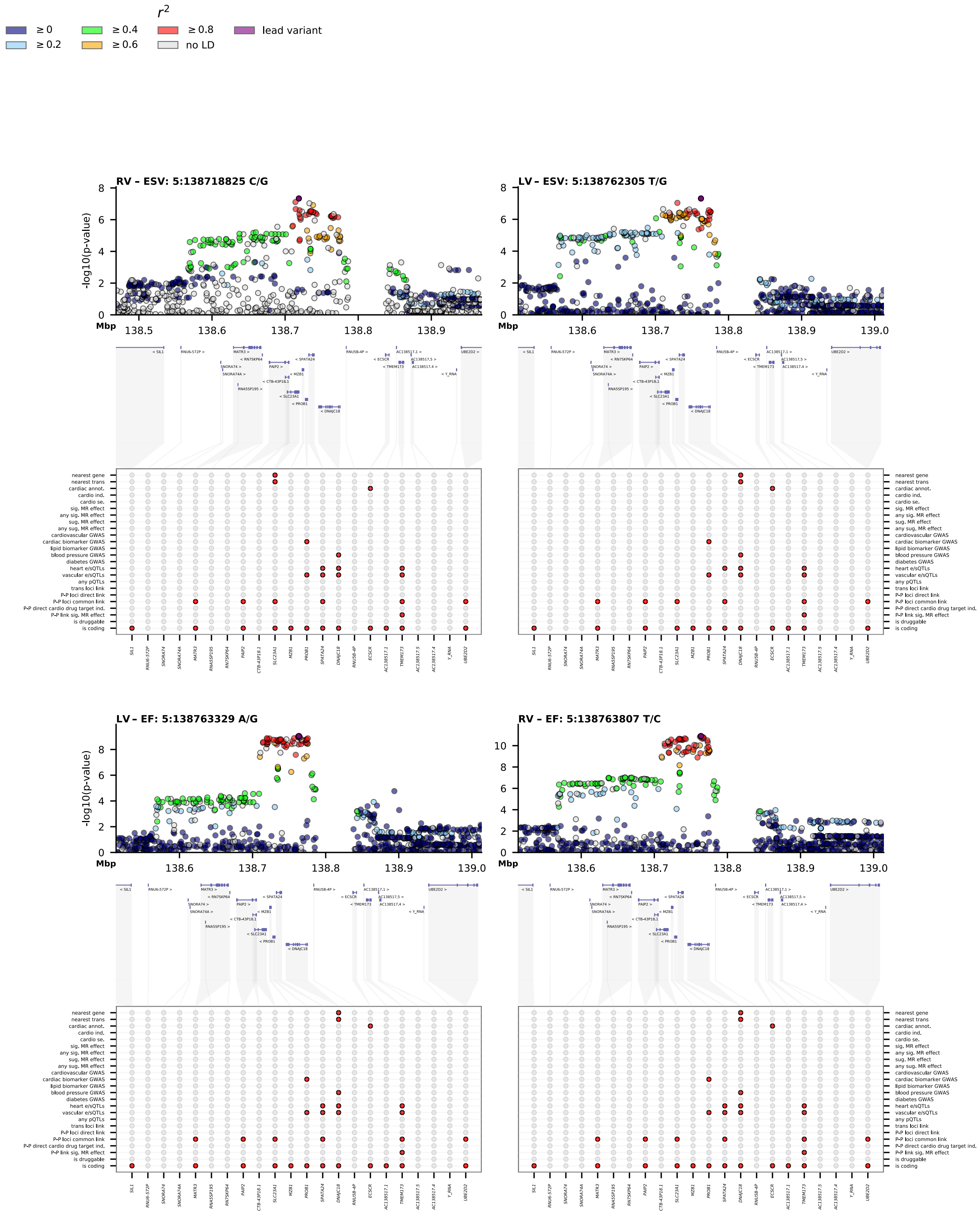

r^2
≥ 0
≥ 0.2
≥ 0.4
≥ 0.6
≥ 0.8
no LD
lead variant
RV – ESV: 5:138718825 C/G
LV – ESV: 5:138762305 T/G
LV – EF: 5:138763329 A/G
RV – EF: 5:138763807 T/C
-log10(p-value)
Mbp
nearest gene
nearest trans
cardiac annot.
cardio ind.
cardio se.
sig. MR effect
any sig. MR effect
sug. MR effect
any sug. MR effect
cardiovascular GWAS
cardiac biomarker GWAS
lipid biomarker GWAS
blood pressure GWAS
diabetes GWAS
heart e/sQTLs
vascular e/sQTLs
any pQTLs
trans loci link
P-P loci direct link
P-P loci common link
P-P direct cardio drug target ind.
P-P link sig. MR effect
is druggable
is coding

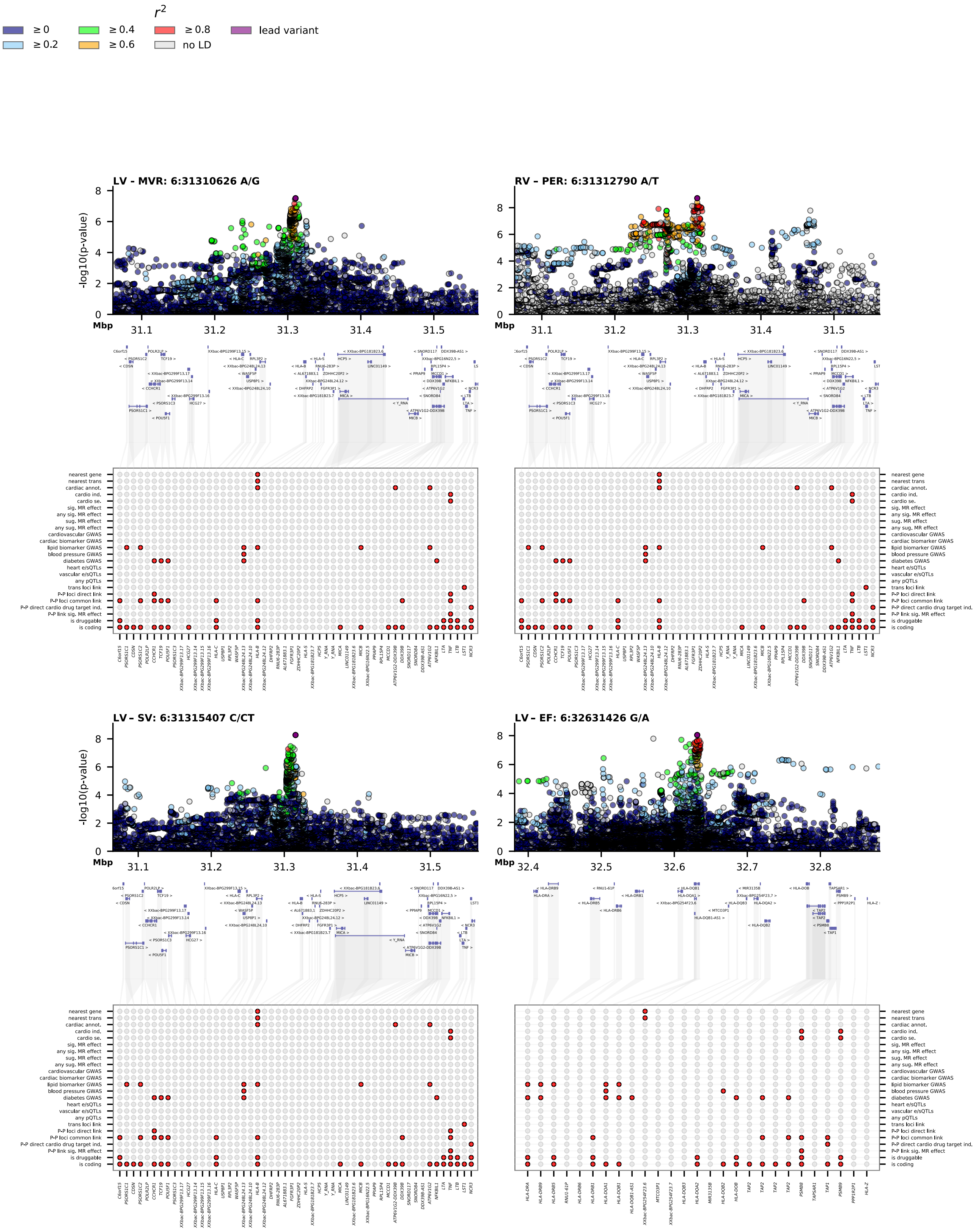

r2
≥ 0
≥ 0.2
≥ 0.4
≥ 0.6
≥ 0.8
no LD
lead variant
LV - MVR: 6:31310626 A/G
RV - PER: 6:31312790 A/T
LV – SV: 6:31315407 C/CT
LV – EF: 6:32631426 G/A
-log10(p-value)
Mbp
nearest gene
nearest trans
cardiac annot.
cardio ind.
cardio se.
sig. MR effect
any sig. MR effect
sug. MR effect
any sug. MR effect
cardiovascular GWAS
cardiac biomarker GWAS
lipid biomarker GWAS
blood pressure GWAS
diabetes GWAS
heart e/sQTLs
vascular e/sQTLs
any pQTLs
trans loci link
P-P loci direct link
P-P loci common link
P-P direct cardio drug target ind.
P-P link sig. MR effect
is druggable
is coding

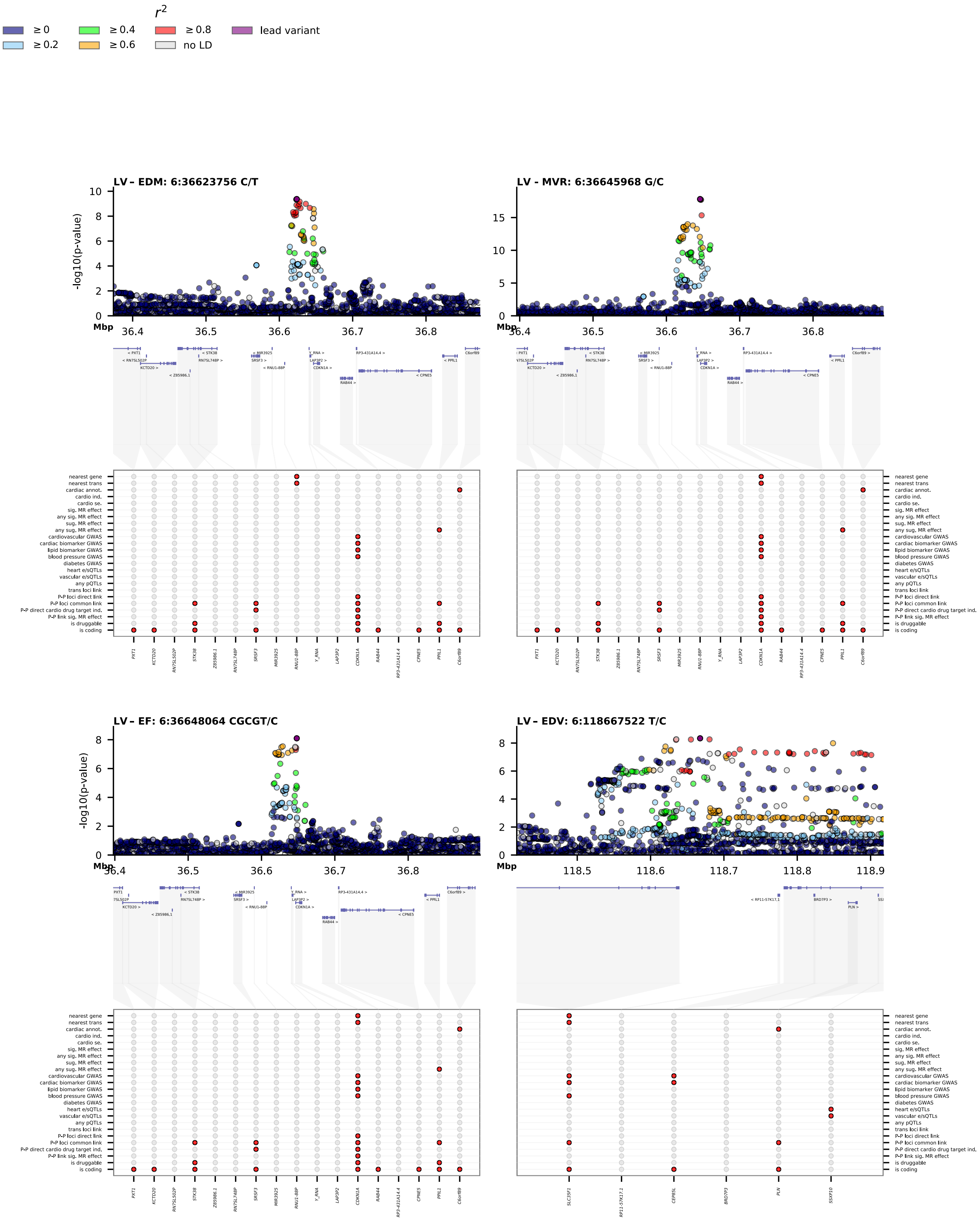

r^2
≥ 0
≥ 0.2
≥ 0.4
≥ 0.6
≥ 0.8
no LD
lead variant
LV – EDM: 6:36623756 C/T
LV – MVR: 6:36645968 G/C
LV – EF: 6:36648064 CGCGT/C
LV – EDV: 6:118667522 T/C
-log10(p-value)
Mbp
nearest gene
nearest trans
cardiac annot.
cardio ind.
cardio se.
sig. MR effect
any sig. MR effect
sug. MR effect
any sug. MR effect
cardiovascular GWAS
cardiac biomarker GWAS
lipid biomarker GWAS
blood pressure GWAS
diabetes GWAS
heart e/sQTLs
vascular e/sQTLs
any pQTLs
trans loci link
P-P loci direct link
P-P loci common link
P-P direct cardio drug target ind.
P-P link sig. MR effect
is druggable
is coding
PXT1
KCTD20
RN7SL502P
STK38
Z85986.1
RN7SL748P
SRSF3
MIR3925
RNU1-88P
Y_RNA
LAP3P2
CDKN1A
RAB44
RP3-431A14.4
CPNE5
PPIL1
C6orf89
SLC35F1
RP11-57K17.1
CEP85L
BRD7P3
PLN
SSXP10

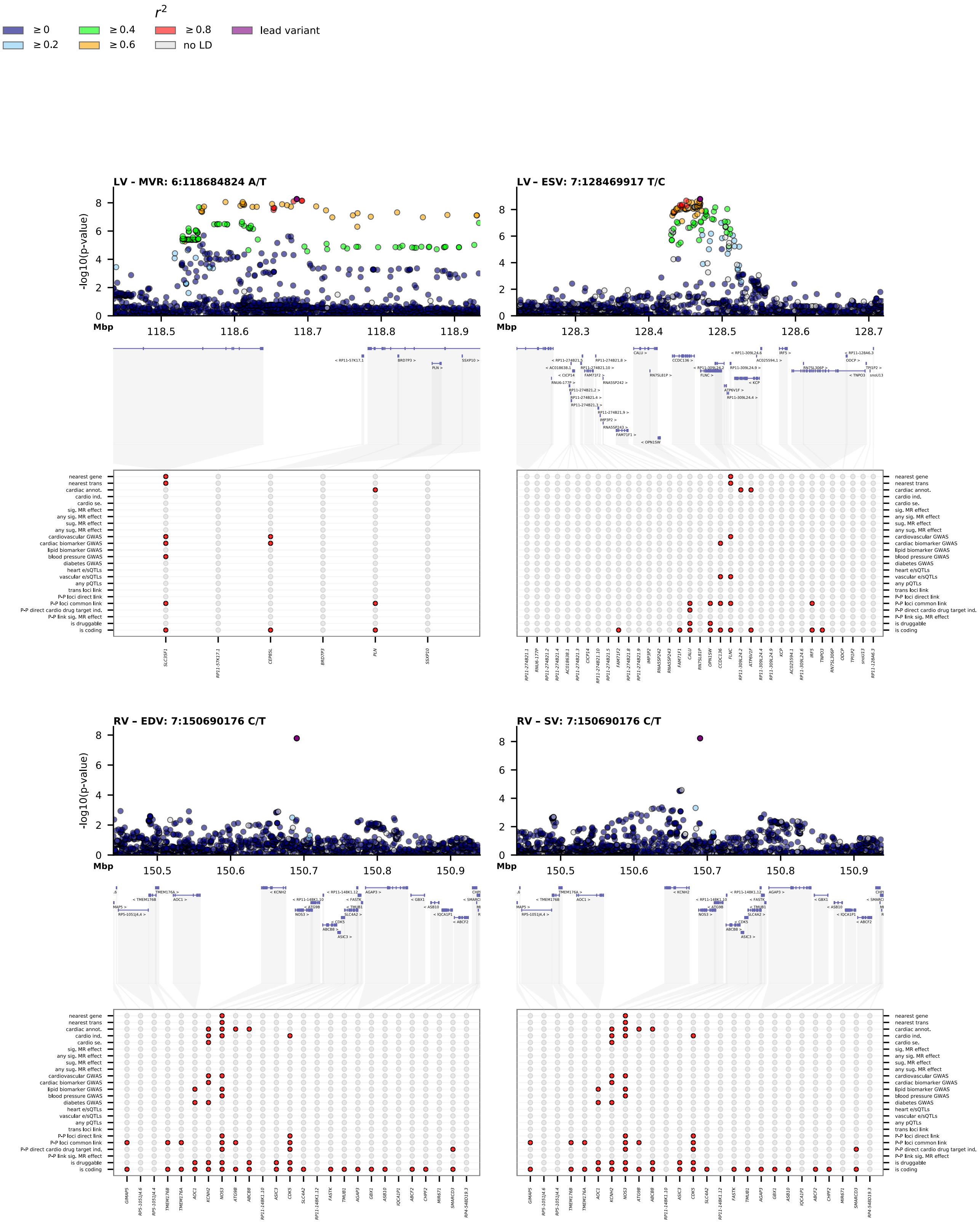

r^2
≥ 0
≥ 0.2
≥ 0.4
≥ 0.6
≥ 0.8
no LD
lead variant
LV - MVR: 6:118684824 A/T
LV – ESV: 7:128469917 T/C
RV – EDV: 7:150690176 C/T
RV – SV: 7:150690176 C/T
-log10(p-value)
Mbp
nearest gene
nearest trans
cardiac annot.
cardio ind.
cardio se.
sig. MR effect
any sig. MR effect
sug. MR effect
any sug. MR effect
cardiovascular GWAS
cardiac biomarker GWAS
lipid biomarker GWAS
blood pressure GWAS
diabetes GWAS
heart e/sQTLs
vascular e/sQTLs
any pQTLs
trans loci link
P-P loci direct link
P-P loci common link
P-P direct cardio drug target ind.
P-P link sig. MR effect
is druggable
is coding
SLC35F1
RP11-57K17.1
CEP85L
BRD7P3
PLN
SSXP10

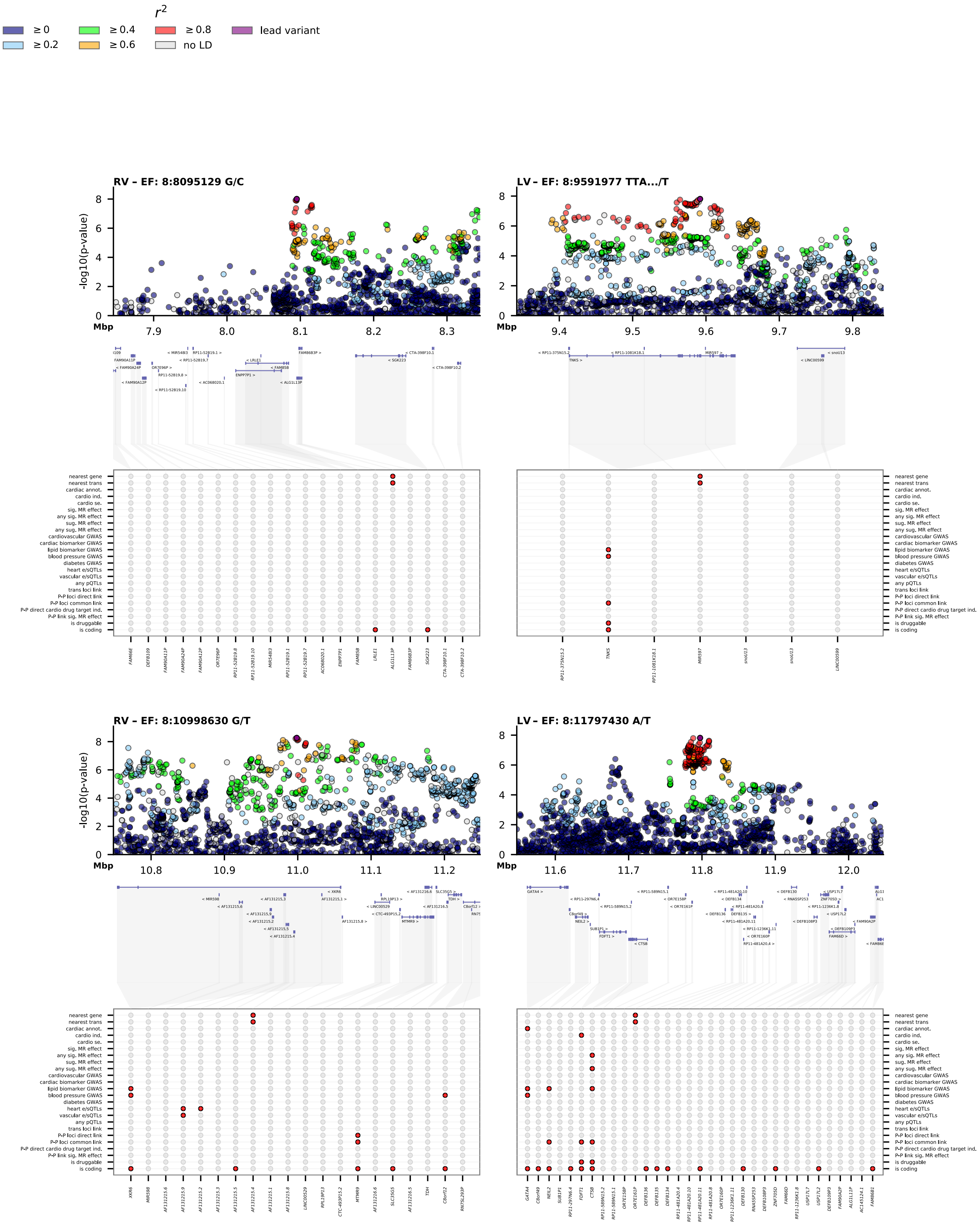

r^2
≥ 0
≥ 0.2
≥ 0.4
≥ 0.6
≥ 0.8
no LD
lead variant
RV – EF: 8:8095129 G/C
LV – EF: 8:9591977 TTA.../T
RV – EF: 8:10998630 G/T
LV – EF: 8:11797430 A/T
-log10(p-value)
Mbp
nearest gene
nearest trans
cardiac annot.
cardio ind.
cardio se.
sig. MR effect
any sig. MR effect
sug. MR effect
any sug. MR effect
cardiovascular GWAS
cardiac biomarker GWAS
lipid biomarker GWAS
blood pressure GWAS
diabetes GWAS
heart e/sQTLs
vascular e/sQTLs
any pQTLs
trans loci link
P-P loci direct link
P-P loci common link
P-P direct cardio drug target ind.
P-P link sig. MR effect
is druggable
is coding

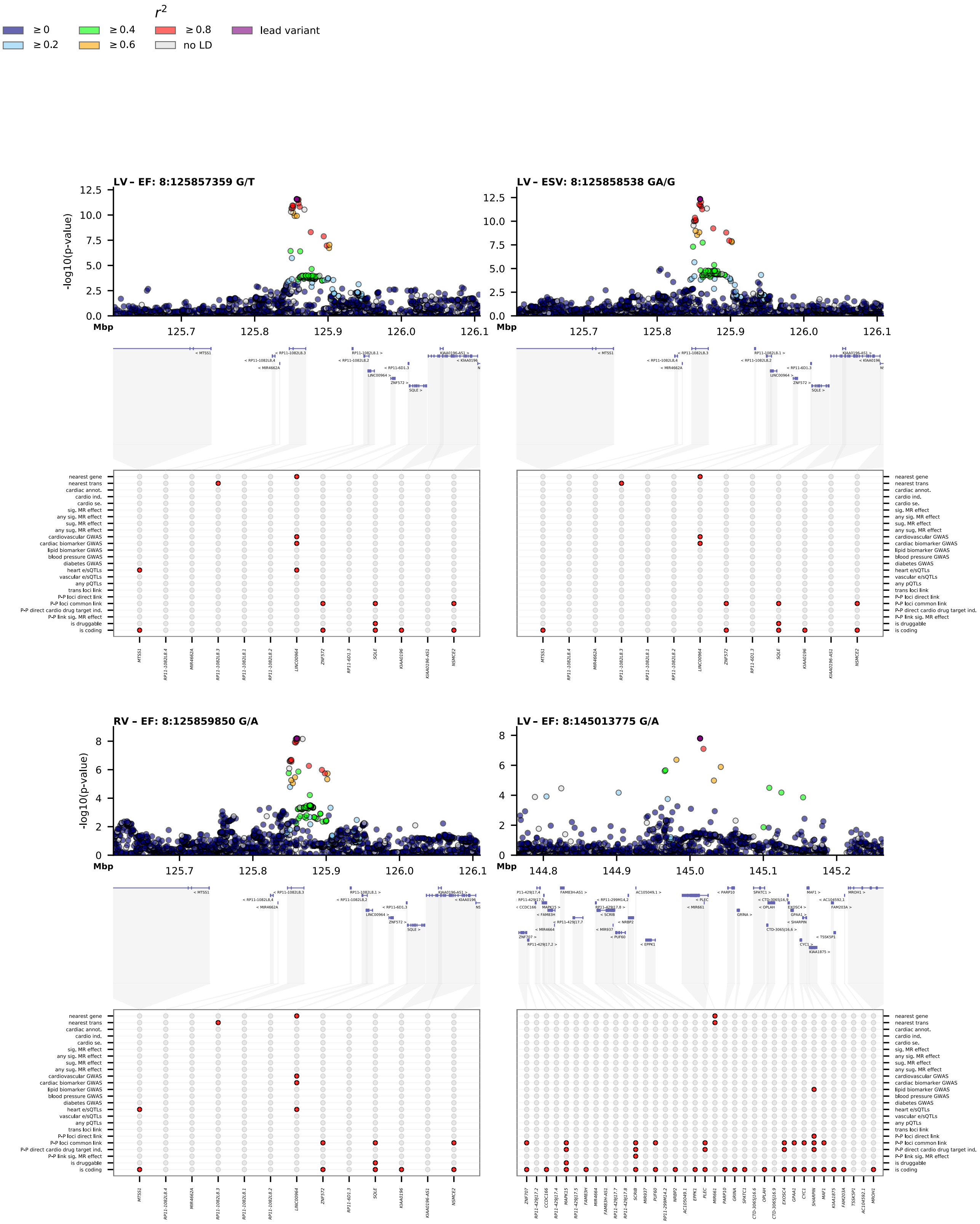

r^2
≥ 0
≥ 0.2
≥ 0.4
≥ 0.6
≥ 0.8
no LD
lead variant
LV – EF: 8:125857359 G/T
LV – ESV: 8:125858538 GA/G
RV – EF: 8:125859850 G/A
LV – EF: 8:145013775 G/A
-log10(p-value)
Mbp
nearest gene
nearest trans
cardiac annot.
cardio ind.
cardio se.
sig. MR effect
any sig. MR effect
sug. MR effect
any sug. MR effect
cardiovascular GWAS
cardiac biomarker GWAS
lipid biomarker GWAS
blood pressure GWAS
diabetes GWAS
heart e/sQTLs
vascular e/sQTLs
any pQTLs
trans loci link
P-P loci direct link
P-P loci common link
P-P direct cardio drug target ind.
P-P link sig. MR effect
is druggable
is coding

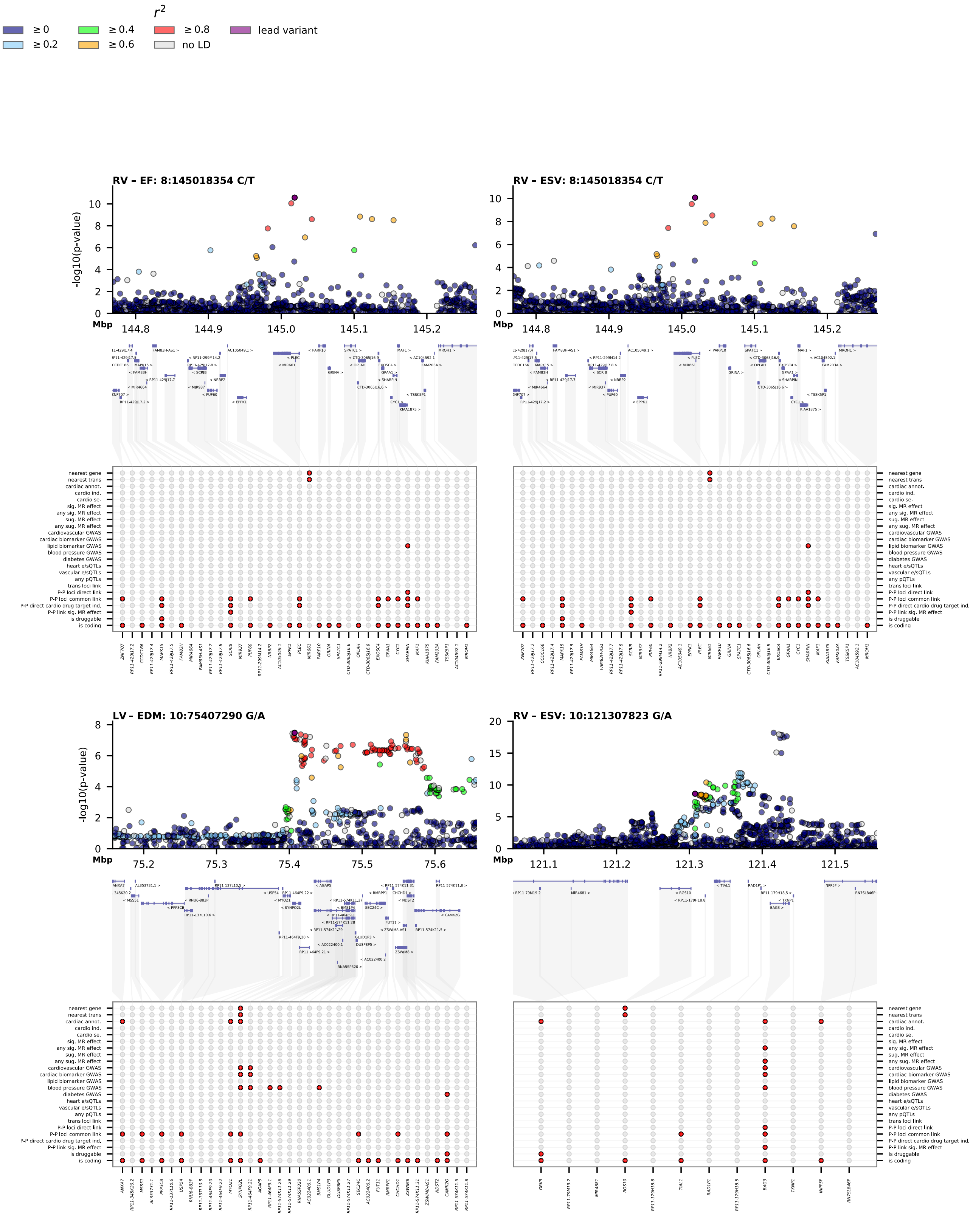

r^2
≥ 0
≥ 0.2
≥ 0.4
≥ 0.6
≥ 0.8
no LD
lead variant
RV – EF: 8:145018354 C/T
RV – ESV: 8:145018354 C/T
LV – EDM: 10:75407290 G/A
RV – ESV: 10:121307823 G/A
-log10(p-value)
Mbp
nearest gene
nearest trans
cardiac annot.
cardio ind.
cardio se.
sig. MR effect
any sig. MR effect
sug. MR effect
any sug. MR effect
cardiovascular GWAS
cardiac biomarker GWAS
lipid biomarker GWAS
blood pressure GWAS
diabetes GWAS
heart e/sQTLs
vascular e/sQTLs
any pQTLs
trans loci link
P-P loci direct link
P-P loci common link
P-P direct cardio drug target ind.
P-P link sig. MR effect
is druggable
is coding

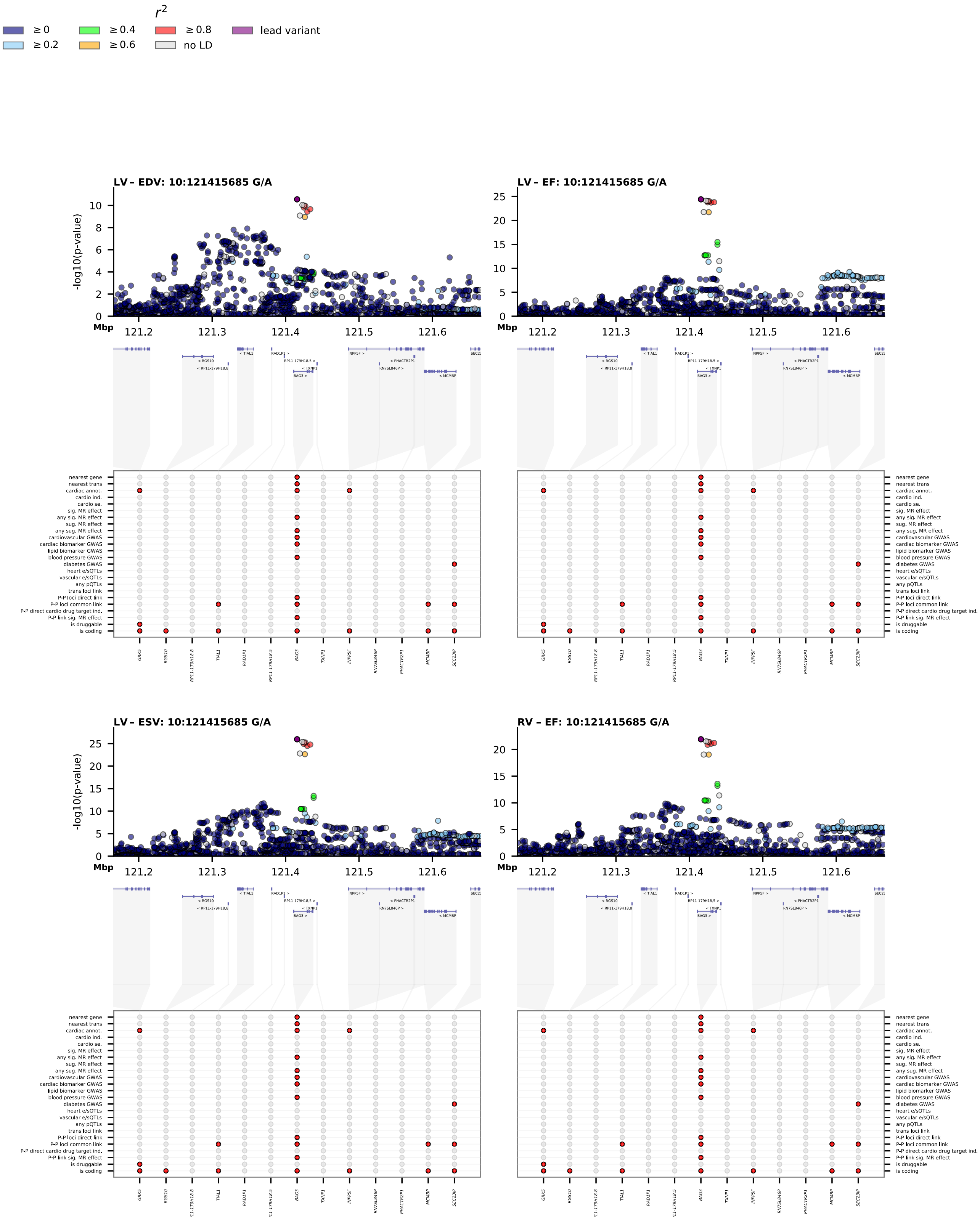

r^2
≥ 0
≥ 0.2
≥ 0.4
≥ 0.6
≥ 0.8
no LD
lead variant
LV – EDV: 10:121415685 G/A
LV – EF: 10:121415685 G/A
LV – ESV: 10:121415685 G/A
RV – EF: 10:121415685 G/A
-log10(p-value)
Mbp
121.2
121.3
121.4
121.5
121.6
nearest gene
nearest trans
cardiac annot.
cardio ind.
cardio se.
sig. MR effect
any sig. MR effect
sug. MR effect
any sug. MR effect
cardiovascular GWAS
cardiac biomarker GWAS
lipid biomarker GWAS
blood pressure GWAS
diabetes GWAS
heart e/sQTLs
vascular e/sQTLs
any pQTLs
trans loci link
P-P loci direct link
P-P loci common link
P-P direct cardio drug target ind.
P-P link sig. MR effect
is druggable
is coding
GRK5
RGS10
RP11-179H18.8
TIAL1
RAD1P1
RP11-179H18.5
BAG3
TXNP1
INPP5F
RN7SL846P
PHACTR2P1
MCMBP
SEC23IP

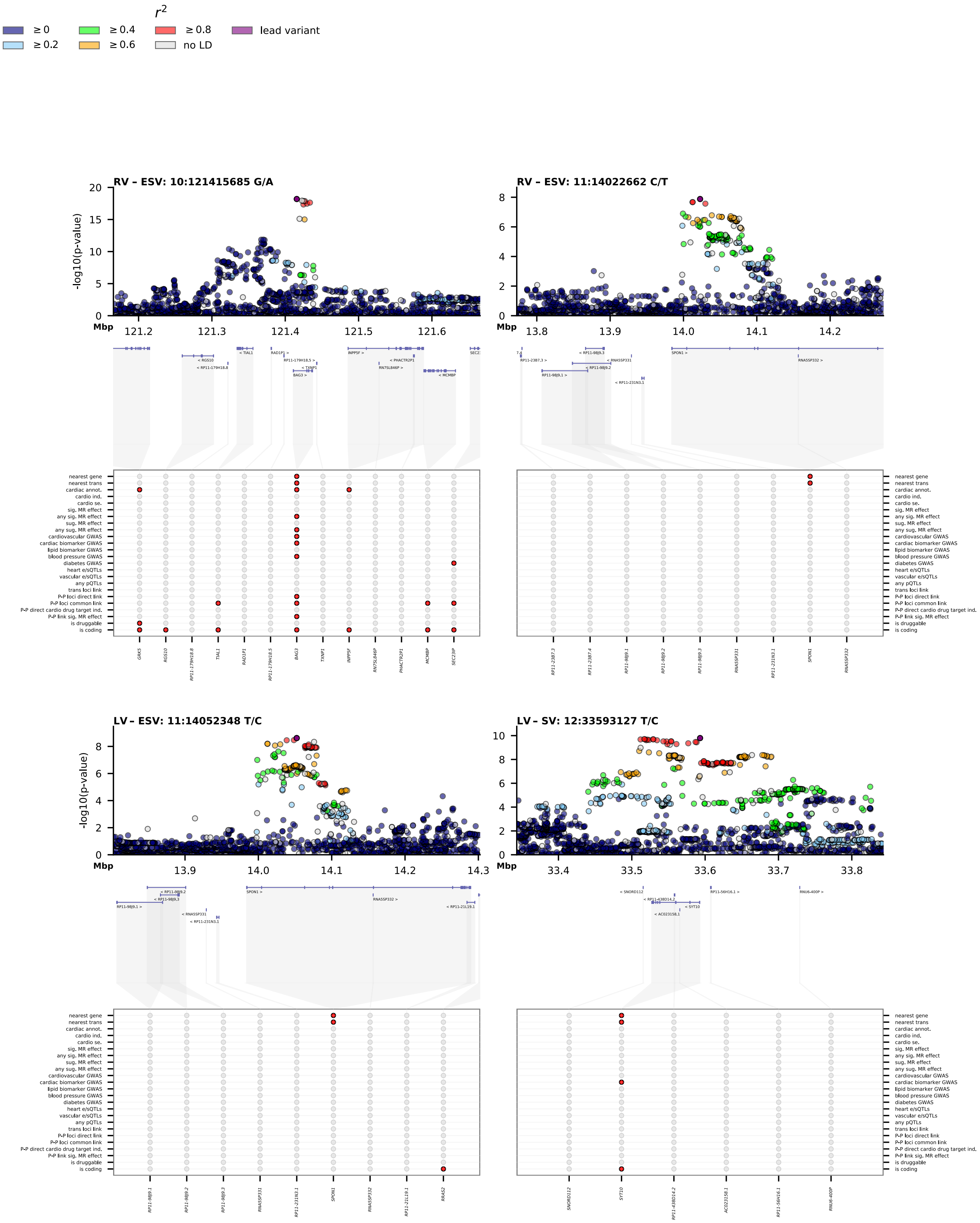

r^2
≥ 0
≥ 0.2
≥ 0.4
≥ 0.6
≥ 0.8
no LD
lead variant
RV – ESV: 10:121415685 G/A
RV – ESV: 11:14022662 C/T
LV – ESV: 11:14052348 T/C
LV – SV: 12:33593127 T/C
-log10(p-value)
Mbp
nearest gene
nearest trans
cardiac annot.
cardio ind.
cardio se.
sig. MR effect
any sig. MR effect
sug. MR effect
any sug. MR effect
cardiovascular GWAS
cardiac biomarker GWAS
lipid biomarker GWAS
blood pressure GWAS
diabetes GWAS
heart e/sQTLs
vascular e/sQTLs
any pQTLs
trans loci link
P-P loci direct link
P-P loci common link
P-P direct cardio drug target ind.
P-P link sig. MR effect
is druggable
is coding
GRK5
RGS10
RP11-179H18.8
TIAL1
RAD1P1
RP11-179H18.5
BAG3
TXNP1
INPP5F
RN7SL846P
PHACTR2P1
MCMBP
SEC23IP
RP11-23B7.3
RP11-23B7.4
RP11-98J9.1
RP11-98J9.2
RP11-98J9.3
RNA5SP331
RP11-231N3.1
SPON1
RNA5SP332
RP11-21L19.1
RRAS2
SNORD112
SYT10
RP11-438D14.2
AC023158.1
RP11-56H16.1
RNU6-400P

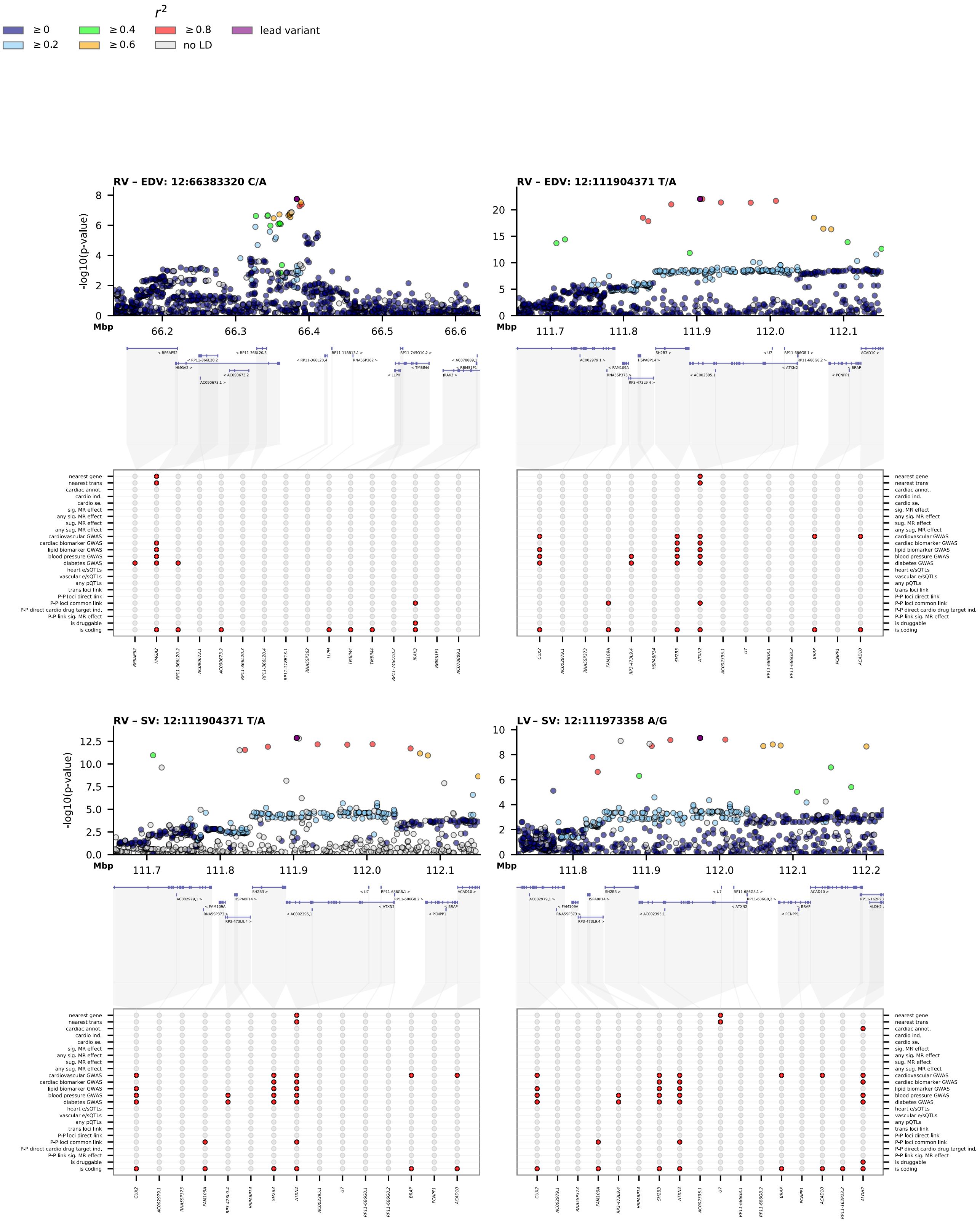

r^2
≥ 0
≥ 0.2
≥ 0.4
≥ 0.6
≥ 0.8
no LD
lead variant
RV – EDV: 12:66383320 C/A
RV – EDV: 12:111904371 T/A
RV – SV: 12:111904371 T/A
LV – SV: 12:111973358 A/G
-log10(p-value)
Mbp
nearest gene
nearest trans
cardiac annot.
cardio ind.
cardio se.
sig. MR effect
any sig. MR effect
sug. MR effect
any sug. MR effect
cardiovascular GWAS
cardiac biomarker GWAS
lipid biomarker GWAS
blood pressure GWAS
diabetes GWAS
heart e/sQTLs
vascular e/sQTLs
any pQTLs
trans loci link
P-P loci direct link
P-P loci common link
P-P direct cardio drug target ind.
P-P link sig. MR effect
is druggable
is coding

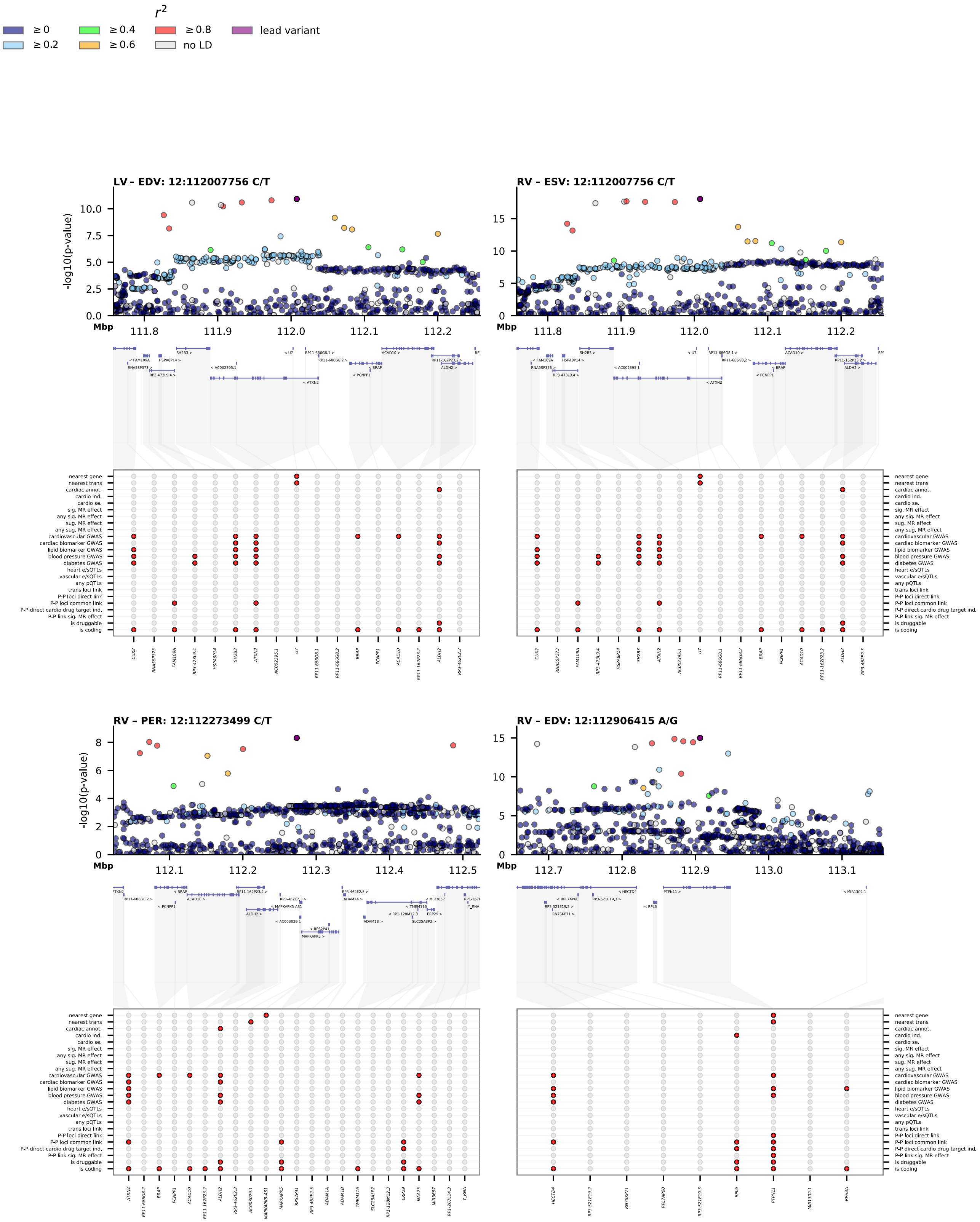

r^2
≥ 0
≥ 0.2
≥ 0.4
≥ 0.6
≥ 0.8
no LD
lead variant
LV – EDV: 12:112007756 C/T
RV – ESV: 12:112007756 C/T
RV – PER: 12:112273499 C/T
RV – EDV: 12:112906415 A/G
-log10(p-value)
Mbp
nearest gene
nearest trans
cardiac annot.
cardio ind.
cardio se.
sig. MR effect
any sig. MR effect
sug. MR effect
any sug. MR effect
cardiovascular GWAS
cardiac biomarker GWAS
lipid biomarker GWAS
blood pressure GWAS
diabetes GWAS
heart e/sQTLs
vascular e/sQTLs
any pQTLs
trans loci link
P-P loci direct link
P-P loci common link
P-P direct cardio drug target ind.
P-P link sig. MR effect
is druggable
is coding

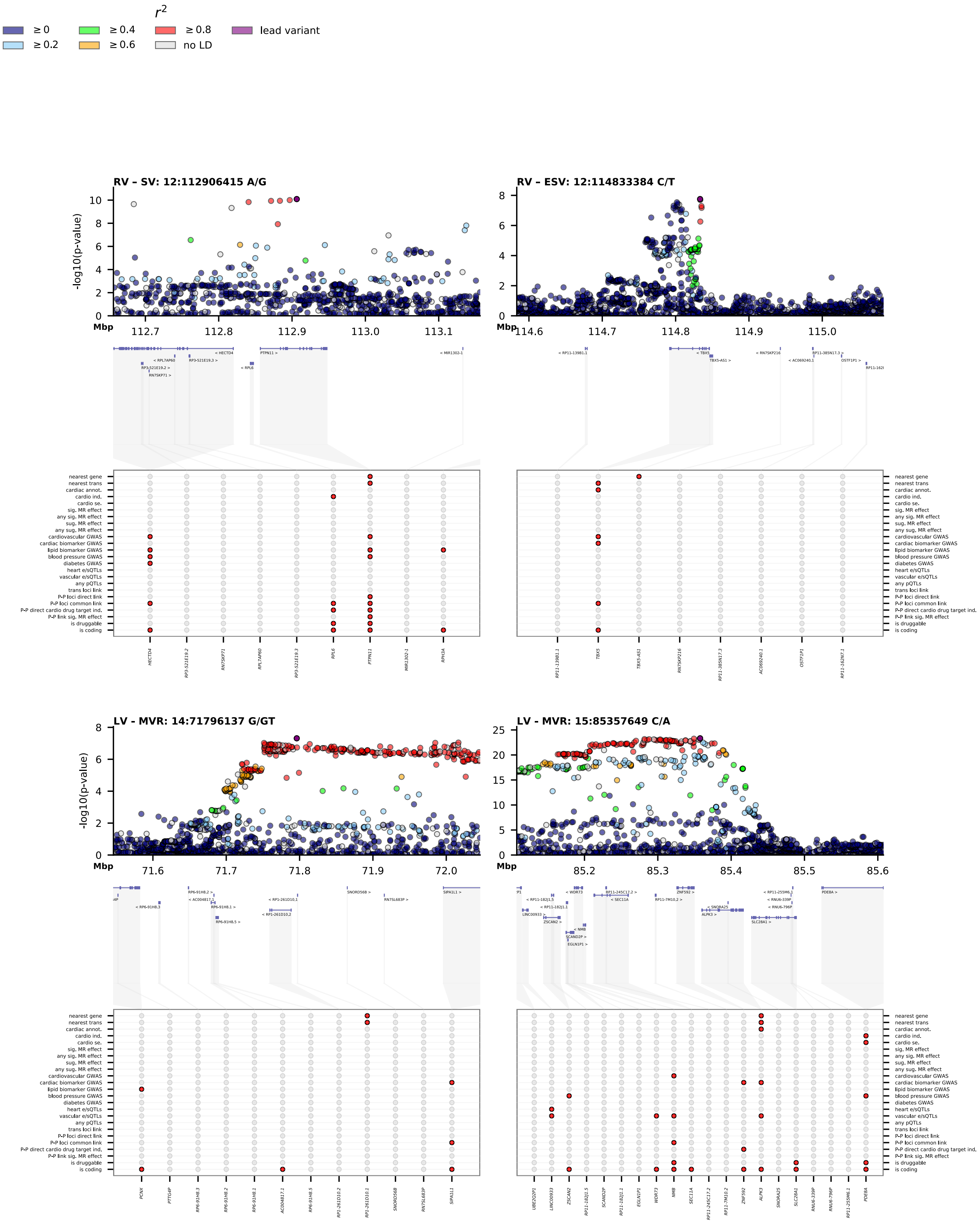

r2
≥ 0
≥ 0.2
≥ 0.4
≥ 0.6
≥ 0.8
no LD
lead variant
RV – SV: 12:112906415 A/G
RV – ESV: 12:114833384 C/T
LV - MVR: 14:71796137 G/GT
LV - MVR: 15:85357649 C/A
-log10(p-value)
Mbp
nearest gene
nearest trans
cardiac annot.
cardio ind.
cardio se.
sig. MR effect
any sig. MR effect
sug. MR effect
any sug. MR effect
cardiovascular GWAS
cardiac biomarker GWAS
lipid biomarker GWAS
blood pressure GWAS
diabetes GWAS
heart e/sQTLs
vascular e/sQTLs
any pQTLs
trans loci link
P-P loci direct link
P-P loci common link
P-P direct cardio drug target ind.
P-P link sig. MR effect
is druggable
is coding
HECTD4
RPL6
PTPN11
TBX5
TBX5-AS1
PCNX
SIPA1L1
ZSCAN2
WDR73
NMB
SEC11A
ZNF592
ALPK3
SLC28A1
PDE8A

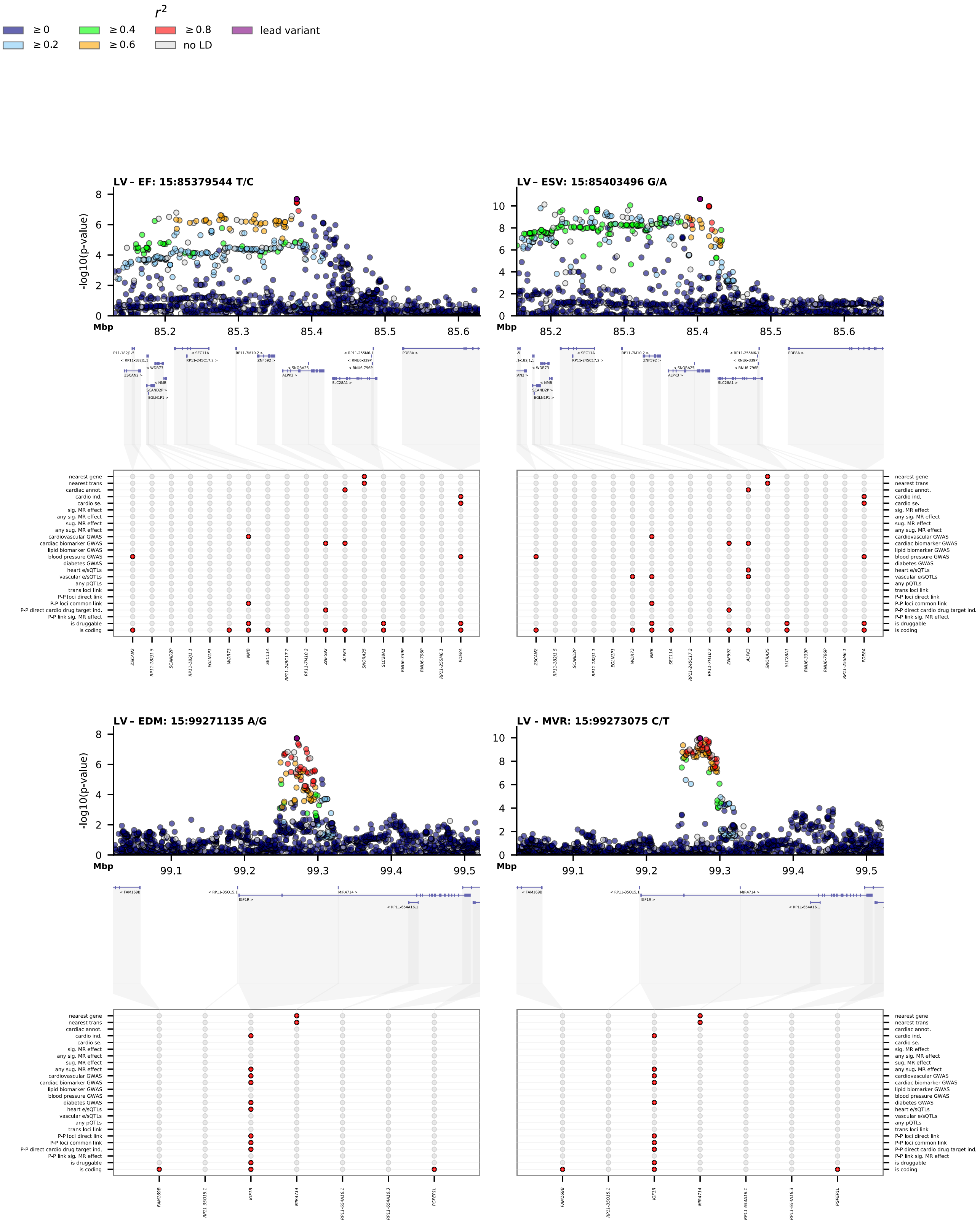

r^2
≥ 0
≥ 0.2
≥ 0.4
≥ 0.6
≥ 0.8
no LD
lead variant
LV – EF: 15:85379544 T/C
LV – ESV: 15:85403496 G/A
LV – EDM: 15:99271135 A/G
LV - MVR: 15:99273075 C/T
-log10(p-value)
Mbp
nearest gene
nearest trans
cardiac annot.
cardio ind.
cardio se.
sig. MR effect
any sig. MR effect
sug. MR effect
any sug. MR effect
cardiovascular GWAS
cardiac biomarker GWAS
lipid biomarker GWAS
blood pressure GWAS
diabetes GWAS
heart e/sQTLs
vascular e/sQTLs
any pQTLs
trans loci link
P-P loci direct link
P-P loci common link
P-P direct cardio drug target ind.
P-P link sig. MR effect
is druggable
is coding
ZSCAN2
RP11-182J1.5
SCAND2P
RP11-182J1.1
EGLN1P1
WDR73
NMB
SEC11A
RP11-245C17.2
RP11-7M10.2
ZNF592
ALPK3
SNORA25
SLC28A1
RNU6-339P
RNU6-796P
RP11-255M6.1
PDE8A
FAM169B
RP11-35O15.1
IGF1R
MIR4714
RP11-654A16.1
RP11-654A16.3
PGPEP1L

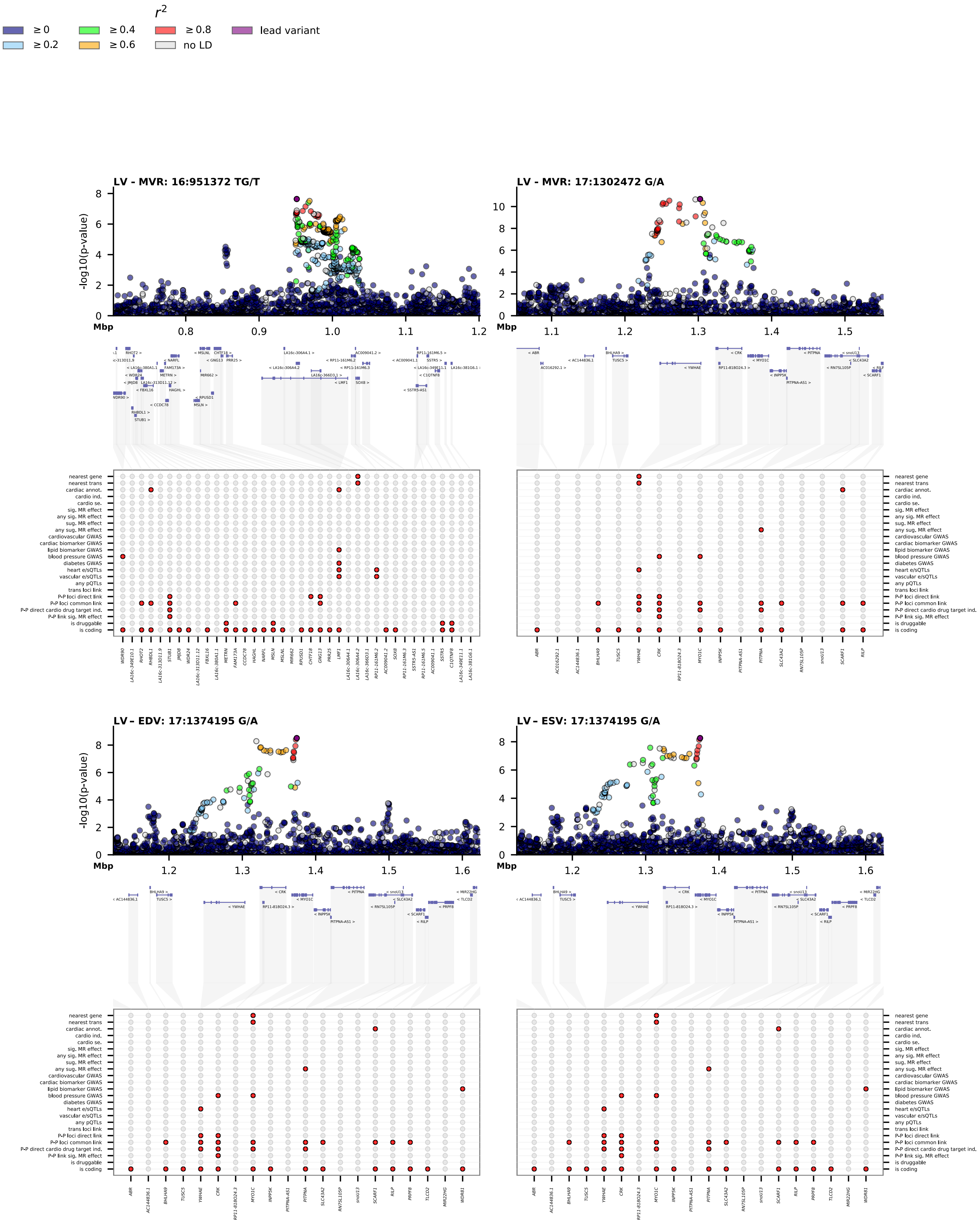

r2
≥ 0
≥ 0.2
≥ 0.4
≥ 0.6
≥ 0.8
no LD
lead variant
LV - MVR: 16:951372 TG/T
LV - MVR: 17:1302472 G/A
LV – EDV: 17:1374195 G/A
LV – ESV: 17:1374195 G/A
-log10(p-value)
Mbp
nearest gene
nearest trans
cardiac annot.
cardio ind.
cardio se.
sig. MR effect
any sig. MR effect
sug. MR effect
any sug. MR effect
cardiovascular GWAS
cardiac biomarker GWAS
lipid biomarker GWAS
blood pressure GWAS
diabetes GWAS
heart e/sQTLs
vascular e/sQTLs
any pQTLs
trans loci link
P-P loci direct link
P-P loci common link
P-P direct cardio drug target ind.
P-P link sig. MR effect
is druggable
is coding

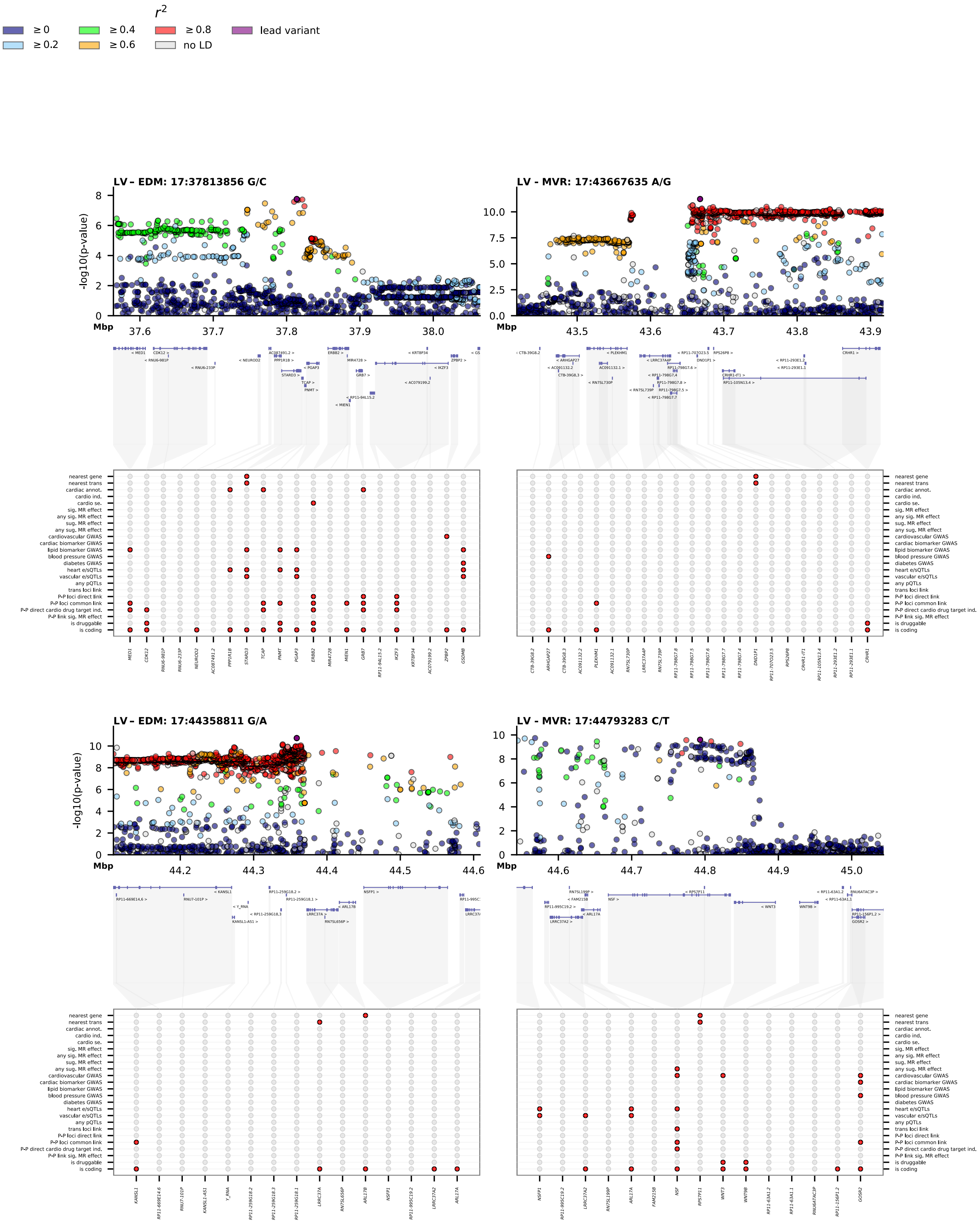

r^2
≥ 0
≥ 0.2
≥ 0.4
≥ 0.6
≥ 0.8
no LD
lead variant
LV – EDM: 17:37813856 G/C
LV - MVR: 17:43667635 A/G
LV – EDM: 17:44358811 G/A
LV - MVR: 17:44793283 C/T
-log10(p-value)
Mbp
nearest gene
nearest trans
cardiac annot.
cardio ind.
cardio se.
sig. MR effect
any sig. MR effect
sug. MR effect
any sug. MR effect
cardiovascular GWAS
cardiac biomarker GWAS
lipid biomarker GWAS
blood pressure GWAS
diabetes GWAS
heart e/sQTLs
vascular e/sQTLs
any pQTLs
trans loci link
P-P loci direct link
P-P loci common link
P-P direct cardio drug target ind.
P-P link sig. MR effect
is druggable
is coding

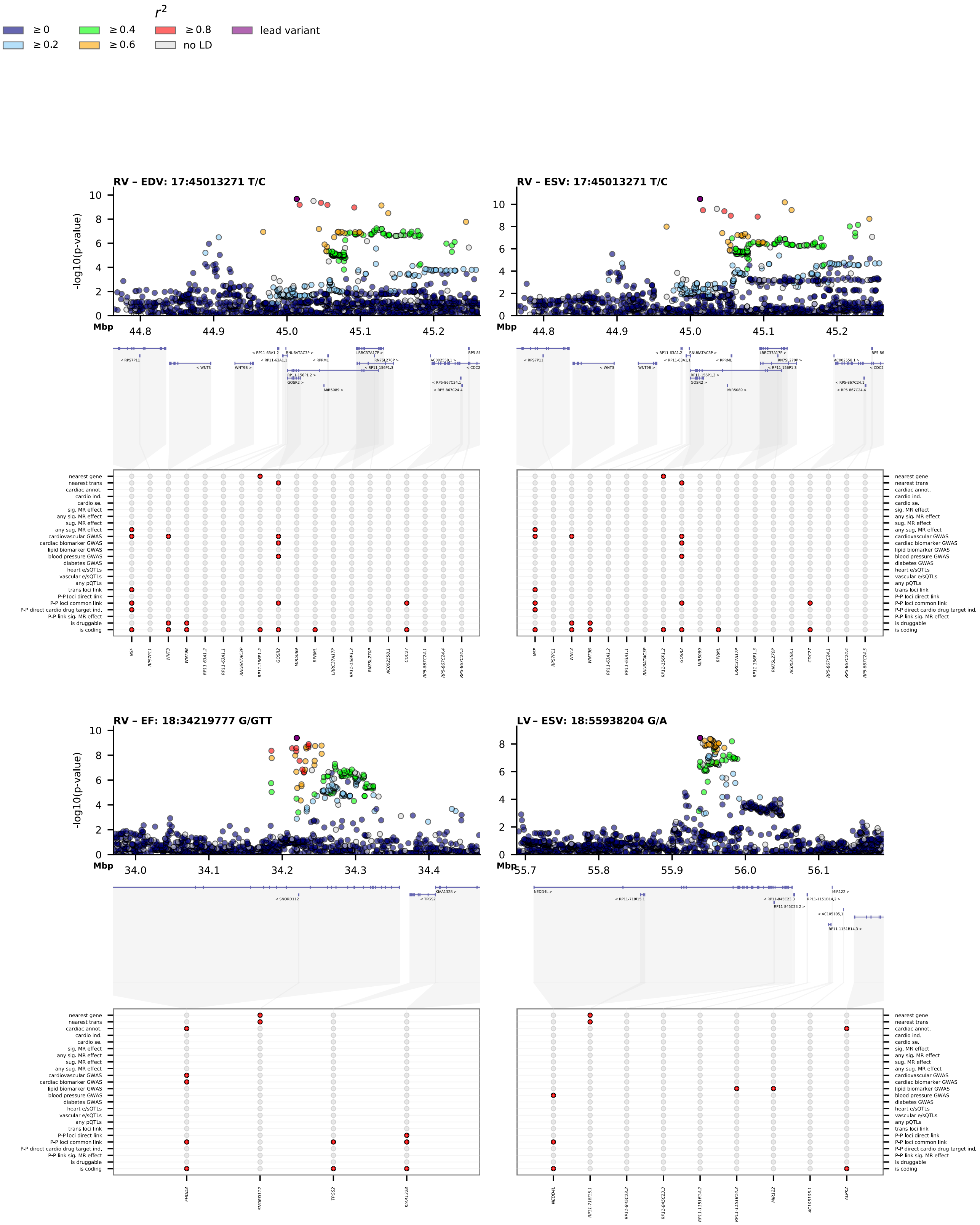

r^2
≥ 0
≥ 0.2
≥ 0.4
≥ 0.6
≥ 0.8
no LD
lead variant
RV – EDV: 17:45013271 T/C
RV – ESV: 17:45013271 T/C
RV – EF: 18:34219777 G/GTT
LV – ESV: 18:55938204 G/A
-log10(p-value)
Mbp
nearest gene
nearest trans
cardiac annot.
cardio ind.
cardio se.
sig. MR effect
any sig. MR effect
sug. MR effect
any sug. MR effect
cardiovascular GWAS
cardiac biomarker GWAS
lipid biomarker GWAS
blood pressure GWAS
diabetes GWAS
heart e/sQTLs
vascular e/sQTLs
any pQTLs
trans loci link
P-P loci direct link
P-P loci common link
P-P direct cardio drug target ind.
P-P link sig. MR effect
is druggable
is coding
NSF
RPS7P11
WNT3
WNT9B
RP11-63A1.2
RP11-63A1.1
RNU6ATAC3P
RP11-156P1.2
GOSR2
MIR5089
RPRML
LRRC37A17P
RP11-156P1.3
RN7SL270P
AC002558.1
CDC27
RP5-867C24.1
RP5-867C24.4
RP5-867C24.5
FHOD3
SNORD112
TPGS2
KIAA1328
NEDD4L
RP11-718I15.1
RP11-845C23.2
RP11-845C23.3
RP11-1151B14.2
RP11-1151B14.3
MIR122
AC105105.1
ALPK2

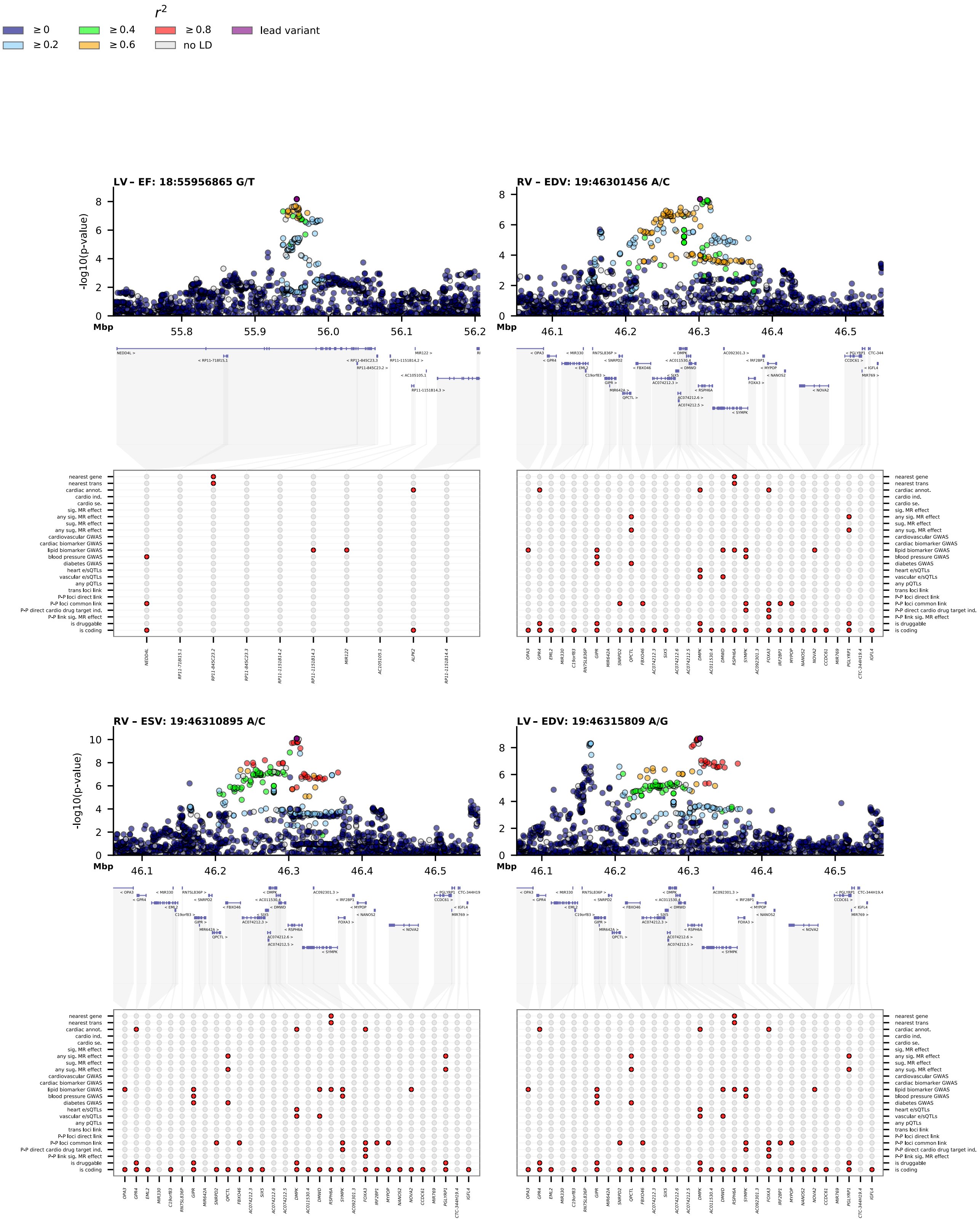

r^2
≥ 0
≥ 0.2
≥ 0.4
≥ 0.6
≥ 0.8
no LD
lead variant
LV – EF: 18:55956865 G/T
RV – EDV: 19:46301456 A/C
RV – ESV: 19:46310895 A/C
LV – EDV: 19:46315809 A/G
-log10(p-value)
Mbp
nearest gene
nearest trans
cardiac annot.
cardio ind.
cardio se.
sig. MR effect
any sig. MR effect
sug. MR effect
any sug. MR effect
cardiovascular GWAS
cardiac biomarker GWAS
lipid biomarker GWAS
blood pressure GWAS
diabetes GWAS
heart e/sQTLs
vascular e/sQTLs
any pQTLs
trans loci link
P-P loci direct link
P-P loci common link
P-P direct cardio drug target ind.
P-P link sig. MR effect
is druggable
is coding

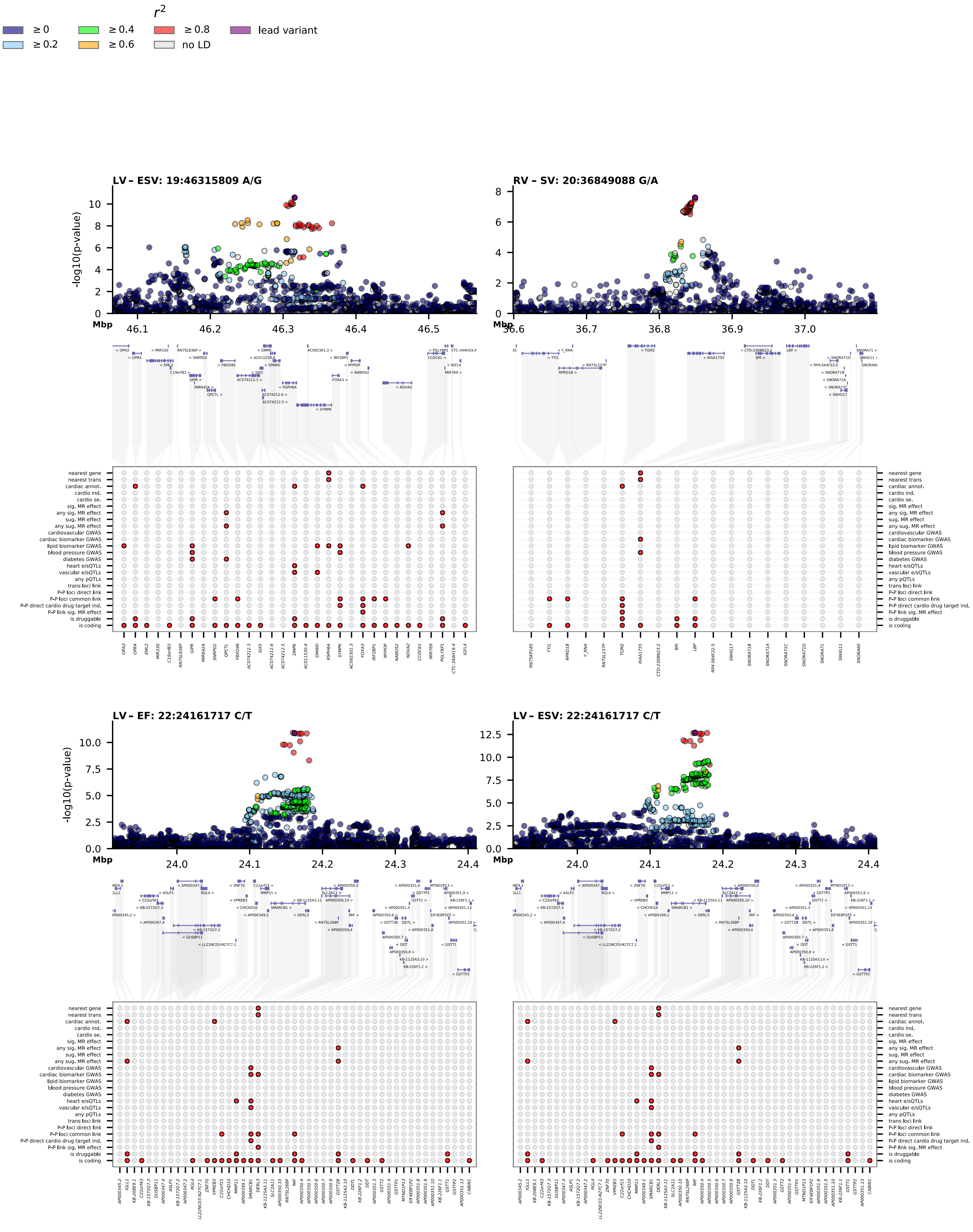

r^2
≥ 0
≥ 0.2
≥ 0.4
≥ 0.6
≥ 0.8
no LD
lead variant
LV – ESV: 19:46315809 A/G
RV – SV: 20:36849088 G/A
LV – EF: 22:24161717 C/T
LV – ESV: 22:24161717 C/T
-log10(p-value)
Mbp
nearest gene
nearest trans
cardiac annot.
cardio ind.
cardio se.
sig. MR effect
any sig. MR effect
sug. MR effect
any sug. MR effect
cardiovascular GWAS
cardiac biomarker GWAS
lipid biomarker GWAS
blood pressure GWAS
diabetes GWAS
heart e/sQTLs
vascular e/sQTLs
any pQTLs
trans loci link
P-P loci direct link
P-P loci common link
P-P direct cardio drug target ind.
P-P link sig. MR effect
is druggable
is coding

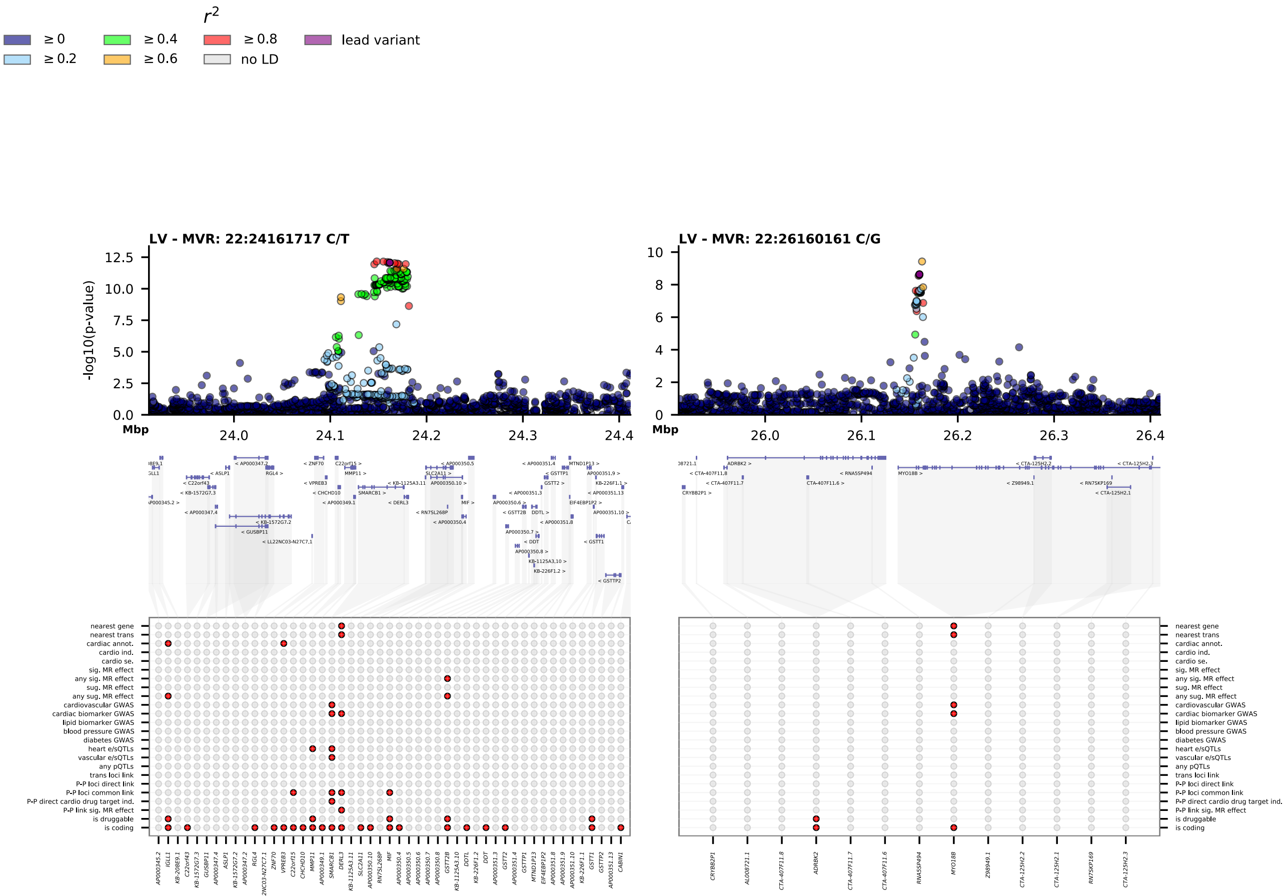

r^2
≥ 0
≥ 0.2
≥ 0.4
≥ 0.6
≥ 0.8
no LD
lead variant
LV - MVR: 22:24161717 C/T
LV - MVR: 22:26160161 C/G
-log10(p-value)
Mbp
nearest gene
nearest trans
cardiac annot.
cardio ind.
cardio se.
sig. MR effect
any sig. MR effect
sug. MR effect
any sug. MR effect
cardiovascular GWAS
cardiac biomarker GWAS
lipid biomarker GWAS
blood pressure GWAS
diabetes GWAS
heart e/sQTLs
vascular e/sQTLs
any pQTLs
trans loci link
P-P loci direct link
P-P loci common link
P-P direct cardio drug target ind.
P-P link sig. MR effect
is druggable
is coding

Gene assignment table

The supplementary file 'GeneAssignment.xlsx' contains the original scoring sheet used to rank the most likely putative causal gene for each CMR locus. The rating was based on the annotated locus view plots presented in Supplementary pdf, including 23 criteria potentially informative for the causal gene assignment. A rank of 1 should be interpreted as the first choice, with any number of subsequent choices possible. The gene with the highest average rank (i.e., the smallest number), was presented as the putative causal gene.

Supplementary note

Keywords of cardiometabolic drug indications and side-effects.

Cardiometabolic indication

- Acute Coronary Syndrome
- Acute exacerbation of bronchiectasis
- Acute exacerbation of chronic obstructive pulmonary disease
- Amyloidosis
- Amyloidosis, Familial
- Anemia
- Anemia, Aplastic
- Anemia, Hemolytic, Autoimmune
- Anemia, Refractory
- Anemia, Refractory, with Excess of Blasts
- Anemia, Sickle Cell
- Aneurysm
- Angioedemas, Hereditary
- Aortic Aneurysm, Abdominal
- Aortic Diseases
- Aortic Valve Stenosis
- Asthma
- Atrial Fibrillation
- Atrial Flutter
- Bronchitis, Chronic
- Cardiomyopathies
- Cardiovascular Diseases
- Coronary Artery Disease
- Coronary Disease
- Emphysema
- Essential Hypertension
- Fibrosis
- Heart Diseases
- Heart Failure
- Heart failure (for patients in sinus rhythm)
- Hypertension
- Hypertension, Pulmonary
- Hypotension, Orthostatic
- Idiopathic Pulmonary Fibrosis
- Ischemia
- Lung Diseases, Interstitial
- Lung Diseases, Obstructive
- Maintenance, for atrial fibrillation or flutter
- Mild to moderate essential hypertension
- Myocardial Infarction
- Myocarditis
- Myositis
- Myositis, Inclusion Body
- Non-ST Elevated Myocardial Infarction
- Pericarditis
- Peripheral Arterial Disease
- Pneumonia

- Pneumonia, Viral
- Pre-Eclampsia
- Prophylaxis of acute rejection in cardiac transplantation (in combination with ciclosporin and corticosteroids) (under expert supervision)
- Prophylaxis of atherothrombotic events following an acute coronary syndrome with elevated cardiac biomarkers (in combination with aspirin alone or aspirin and clopidogrel)
- Prophylaxis of atherothrombotic events in patients with coronary artery disease or symptomatic peripheral artery disease at high risk of ischaemic events (in combination with aspirin)
- Prophylaxis of stroke and systemic embolism in non-valvular atrial fibrillation and at least one risk factor (such as previous stroke or transient ischaemic attack, symptomatic heart failure, diabetes mellitus, hypertension, or age 75 years and over)
- Prophylaxis of stroke and systemic embolism in patients with non-valvular atrial fibrillation and with at least one of the following risk factors: congestive heart failure, hypertension, previous stroke or transient ischaemic attack, age ≥ 75 years, or diabetes mellitus
- Pulmonary Disease, Chronic Obstructive
- Pulmonary Embolism
- Pulmonary Fibrosis
- Pulmonary Veno-Occlusive Disease
- Respiratory Distress Syndrome, Adult
- Rheumatic Heart Disease
- Sarcoidosis
- Stroke
- Tachycardia, Supraventricular
- Thrombocythemia, Essential
- Thrombocytopenia
- Thromboembolism
- Thrombosis
- Vascular Diseases
- Venous Thromboembolism
- Venous Thrombosis
- Severe acute asthma in patients not previously treated with theophylline
- Coronary Restenosis
- Secondary prevention of ischaemic stroke and transient ischaemic attacks
- Severe acute asthma
- Treatment of deep-vein thrombosis
- Adjunct to oral anticoagulation for prophylaxis of thromboembolism associated with prosthetic heart valves
- Severe acute exacerbation of chronic obstructive pulmonary disease in patients not previously treated with theophylline
- Atherosclerosis
- Heart Arrest
- Angina, Unstable
- Secondary prevention of ischaemic stroke (not asso-

ciated with atrial fibrillation) and transient ischaemic attacks (used alone or with aspirin)
- Cerebral Arterial Diseases
- Chronic asthma
- Embolism
- Arrhythmias, Cardiac
- Adjunct to bronchodilators for the maintenance treatment of patients with severe chronic obstructive pulmonary disease associated with chronic bronchitis and a history of frequent exacerbations
- Hypercholesterolemia
- Diabetes Mellitus
- Ischemic Attack, Transient
- Type 2 diabetes mellitus [monotherapy or in combination with other antidiabetic drugs (including insulin)]
- Severe acute exacerbation of chronic obstructive pulmonary disease
- Diabetes Mellitus, Type 1
- Hereditary angioedema
- Type 2 diabetes mellitus [reduction in risk or delay of onset]
- Angina, Stable
- Myocardial Ischemia
- Carcinoid Heart Disease
- Carotid Stenosis
- Diabetes Mellitus, Type 2
- ST Elevation Myocardial Infarction
- Hypotension

Cardiometabolic side-effects

- Acute coronary syndrome
- anaemia
- Anaemia
- aneurysm
- Aneurysm
- angina pectoris
- Angina pectoris
- arrhythmia
- arrhythmias
- Arrhythmias
- Arterial thromboembolism
- artery dissection
- ascites
- asthma
- atrioventricular block
- bradycardia
- cardiac arrest
- Cardiac arrest
- cardiac conduction disorder
- cardiac disorder
- Cardiac disorder
- cardiac inflammation
- cardiomegaly
- cardiomyopathy
- cardiovascular disorders
- cerebral ischaemia
- cerebrovascular insufficiency
- chest discomfort
- chest pain
- Chest pain
- chest tightness
- circulatory collapse
- coagulation disorders
- coma
- congestive heart failure
- Coronary artery insufficiency
- cyanosis
- diabetes mellitus

- dyslipidaemia
- Dyslipidaemia
- dyspnoea
- Dyspnoea
- embolism and thrombosis
- Embolism and thrombosis
- Endocarditis
- haemorrhage
- Haemorrhage
- hypercalcaemia
- hypercholesterolaemia
- hyperglycaemia
- Hyperglycaemia
- hypertension
- hyperthyroidism
- hypocalcaemia
- hypoglycaemia
- hypokalaemia
- hypomagnesaemia
- hyponatraemia
- hypophosphataemia
- hypotension
- hypoxia
- interstitial lung disease
- ischaemia
- ischaemic heart disease
- multi organ failure
- myocardial dysfunction
- myocardial infarction
- Myocardial infarction
- myocardial ischaemia
- myocarditis
- Myocarditis
- myopathy
- myositis
- oedema
- palpitations
- pericardial disorders
- pericardial effusion
- pericarditis
- Pulmonary arterial hypertension
- pulmonary embolism
- pulmonary hypertension
- pulmonary oedema
- QT interval prolongation
- respiratory disorders
- Respiratory disorders
- sarcoidosis
- stroke
- supraventricular tachycardia
- syncope
- tachycardia
- thyroid disorder
- type 1 diabetes mellitus
- ventricular dysfunction
- Asthma
- Prophylaxis of stroke and systemic embolism in patients with non-valvular atrial fibrillation and with at least one of the following risk factors: congestive heart failure, hypertension, previous stroke or transient ischaemic attack, age ≥ 75 years, or diabetes mellitus
- Cerebral Amyloid Angiopathy
- Acute Coronary Syndrome
- Severe acute asthma in patients not previously treated with theophylline
- Coronary Restenosis
- Non-ST Elevated Myocardial Infarction
- Secondary prevention of ischaemic stroke and transient ischaemic attacks
- Pericarditis
- Stroke
- Severe acute asthma
- Treatment of deep-vein thrombosis
- Adjunct to oral anticoagulation for prophylaxis of thromboembolism associ-

ated with prosthetic heart valves
- Severe acute exacerbation of chronic obstructive pulmonary disease in patients not previously treated with theophylline
- Atherosclerosis
- Prophylaxis of atherothrombotic events in patients with coronary artery disease or symptomatic peripheral artery disease at high risk of ischaemic events (in combination with aspirin)
- Anemia
- Peripheral Arterial Disease
- Aortic Valve Stenosis
- Amyloidosis
- Thromboembolism
- Venous Thromboembolism
- Atrial Fibrillation
- Heart Arrest
- Thrombosis
- Angina, Unstable
- Secondary prevention of ischaemic stroke (not associated with atrial fibrillation) and transient ischaemic attacks (used alone or with aspirin)
- Heart Diseases
- Pneumonia
- Coronary Disease
- Prophylaxis of stroke and systemic embolism in nonvalvular atrial fibrillation and at least one risk factor (such as previous stroke or transient ischaemic attack, symptomatic heart failure, diabetes mellitus, hypertension, or age 75 years and over)
- Cerebral Arterial Diseases
- Chronic asthma
- Prophylaxis of recurrent pulmonary embolism
- Pulmonary Embolism
- Embolism
- Hypertension, Pulmonary
- Atrial Flutter
- Arrhythmias, Cardiac
- Rheumatic Heart Disease
- Venous Thrombosis
- Treatment of pulmonary embolism
- Prophylaxis of atherothrombotic events following an acute coronary syndrome with elevated cardiac biomarkers (in combination with aspirin alone or aspirin and clopidogrel)
- Myocardial Infarction
- Heart Failure
- Hypercholesterolemia
- Bronchitis, Chronic
- Diabetes Mellitus
- Sarcoidosis
- Cardiovascular Diseases
- Ischemic Attack, Transient
- Type 2 diabetes mellitus [monotherapy or in combination with other antidiabetic drugs (including insulin)]
- Severe acute exacerbation of chronic obstructive pulmonary disease
- Diabetes Mellitus, Type 1
- Type 2 diabetes mellitus [reduction in risk or delay of onset]
- Coronary Artery Disease
- Angina, Stable
- Myocardial Ischemia
- Hypertension

- Carcinoid Heart Disease
- Carotid Stenosis
- Diabetes Mellitus, Type 2
- ST Elevation Myocardial Infarction
- Hypotension
- Intracranial Aneurysm

Other supplementary materials for this manuscript:

Supplementary File 3 (.xlsx): Scoring sheet identifying the putative causal genes.
